# Building the process-drug–side effect network to discover the relationship between biological Processes and side effects

**DOI:** 10.1186/1471-2105-12-S2-S2

**Published:** 2011-03-29

**Authors:** Sejoon Lee, Kwang H Lee, Min Song, Doheon Lee

**Affiliations:** 1 Bio and Brain Engineering Department, KAIST, Daejeon 305-701, South Korea; 2 Information Systems Department, New Jersey Institute of Technology, University Heights, Newark, USA

## Abstract

**Background:**

Side effects are unwanted responses to drug treatment and are important resources for human phenotype information. The recent development of a database on side effects, the side effect resource (SIDER), is a first step in documenting the relationship between drugs and their side effects. It is, however, insufficient to simply find the association of drugs with biological processes; that relationship is crucial because drugs that influence biological processes can have an impact on phenotype. Therefore, knowing which processes respond to drugs that influence the phenotype will enable more effective and systematic study of the effect of drugs on phenotype. To the best of our knowledge, the relationship between biological processes and side effects of drugs has not yet been systematically researched.

**Methods:**

We propose 3 steps for systematically searching relationships between drugs and biological processes: enrichment scores (ES) calculations, t-score calculation, and threshold-based filtering. Subsequently, the side effect-related biological processes are found by merging the drug-biological process network and the drug-side effect network. Evaluation is conducted in 2 ways: first, by discerning the number of biological processes discovered by our method that co-occur with Gene Ontology (GO) terms in relation to effects extracted from PubMed records using a text-mining technique and second, determining whether there is improvement in performance by limiting response processes by drugs sharing the same side effect to frequent ones alone.

**Results:**

The multi-level network (the process-drug-side effect network) was built by merging the drug-biological process network and the drug-side effect network. We generated a network of 74 drugs-168 side effects-2209 biological process relation resources. The preliminary results showed that the process-drug-side effect network was able to find meaningful relationships between biological processes and side effects in an efficient manner.

**Conclusions:**

We propose a novel process-drug-side effect network for discovering the relationship between biological processes and side effects. By exploring the relationship between drugs and phenotypes through a multi-level network, the mechanisms underlying the effect of specific drugs on the human body may be understood.

## Background

**Figure 1:**
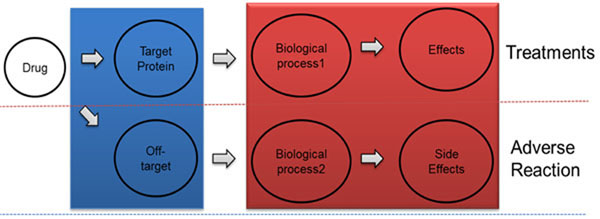
Flow of drug treatments and adverse reaction

Side effects are unwanted responses to drug treatment, and they are important resources of human phenotype information. Drugs bind to target proteins and affect biological processes, and the processes cause phenotype effect. However, drugs may also bind to off-target proteins, which affects other biological processes and causes adverse reactions (Figure 1). Side effects occur mainly when drugs bind to unintended off-targets. These side effects vary from simple symptoms, such as headache, to critical symptoms, such as carcinoma. Most side effects are harmful to humans, but side effects can also be utilized to find new uses for known drugs, such as Viagra. Therefore, it is highly desirable to automatically discover new targets for known drugs and to understand the mechanisms that cause side effects for target-specific treatments.

In their paper published in *Science*, Campellos et al reported finding new targets based on drugs with similar side effects [1]. They used an ABC network model built with (A) drugs developed for new targets, (B) targets, and (C) side effects. Similarly, Keiser used chemical similarity to find new targets for a known drug [2]. Keiser’s approach enabled the discovery of off-targets of a known drug but did not consider the relationship between a drug and its biological process.

Like Keiser’s and Campellos’s studies, most previous research was focused mostly on finding off-target proteins causing the side effects. In addition, the biological processes that are affected by the drug target need to be considered because they cause phenotypical responses in the human biological system. A drug that influences biological processes can also have an impact on phenotype. Therefore, if the biological process that responds to a drug influencing the phenotype is known then drugs pertinent to the phenotype can be studied more effectively and systematically. To date, the relationships between biological processes and side effects have not been systematically researched.

Two databases are available for studying relationships between side effects and biological processes: the connectivity map and side effects resource (SIDER). The connectivity map is developed to generate and analyze a drug-gene-disease network from large-scale experimental gene expression responses to drugs [3]. SIDER is a recently developed database on side effects to document the relationship between drugs and side effects [4]. The connectivity map provides drug-responsive gene expression information, and SIDER provides drug-side effect relationships.

By utilizing the connectivity map and SIDER, we aimed to automatically discover the relationship between biological processes and side effects by building a multi-level network of drug-biological processes influenced by the association of targets with side effects.

**Figure 2:**
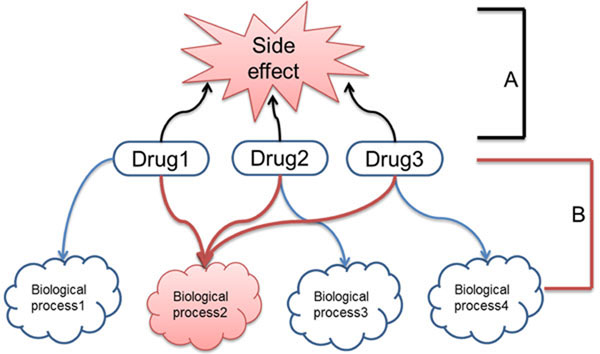
**Concept of discovering side effect-related biological processes.** A: Drug-Side effect network; B: Drug-Biological processes network

Figure 2 is an example of our approach. If drug 1, 2, or 3 induces the same side effect, their common response (biological process2) is potentially related to their side effect. To examine these relationships, SIDER was used to construct the drug-side effect network (Fig. 2A). SIDER provides information on the frequency of connections between drugs and side effects. The drug-side effect relationships are filtered based on the frequency of relevant information to construct a reliable drug-side effect network. The drug responsive biological process network was also constructed using drug responsive gene expression profiles (Fig. 2B).

Gene ontology (GO) terms were used for biological processes, and gene set enrichment scores (ES) were used to find which processes were upregulated or downregulated by the drugs. Subsequently, an ABC network model was built (A, processes; B, drugs; and C, side effects) to find relationships between side effects and biological processes (Fig. 2). The results show that many processes found in the drug-process network were meaningful and were confirmed by previous studies. In addition, a novel network consisting of 168 effects and 2,209 biological processes was constructed, and these relationships based on the ABC model were also confirmed to be significant by support from the literature. Finally, evaluations were conducted in 2 ways: first, by quantifying how many biological processes were found by our method and were concurrently found in GO terms with effects extracted from the PubMed records using a text-mining technique and second, whether there was an improvement in performance by limiting response processes by drugs sharing the same side effect to frequent ones alone. The experimental results showed that our process-drug-side effect network was able to reveal meaningful relationships between biological processes and side effects in an efficient manner.

In addition to comprehensive evaluation, our method contributes to systematically finding relationships between drugs and biological processes using ES scores calculations, t-score calculation, and threshold-based filtering. Second, side effect-related biological processes are revealed by merging the drug-biological process network and the drug-side effect network. Finally, data on 74 drugs, 168 effects, and 2209 biological process relation resources were generated.

## Datasets and methods

To discover the relationships between side effects and biological processes, 2 networks were constructed: the drug-biological process network and the drug-side effect network. Side effect and biological process relationships were automatically revealed by connecting the 2 networks.

### Drug-biological process network construction

**Figure 3:**
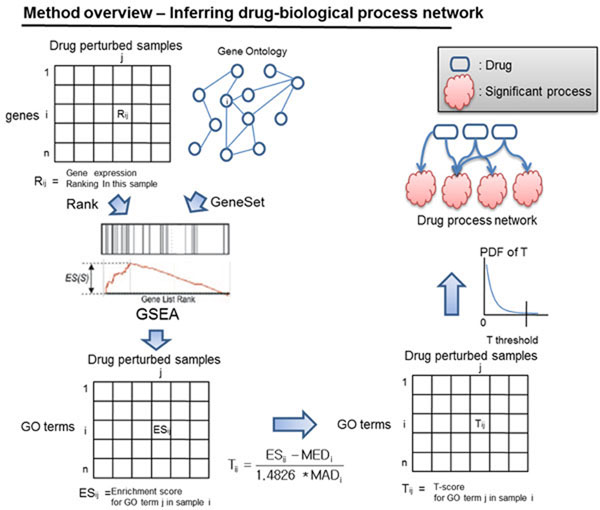
Schematic diagram for inferring relationships between biological processes (GO) and drugs

Figure 3 illustrates an overview of the approach to constructing the drug-process network. To find a drug-responsive biological process, gene rank information from the connectivity map and gene set information available in GO were used. The ES for each GO term was calculated to find significant terms. Subsequently, the t-score was calculated to measure the significance of each process of the drug in question. Finally, a threshold T was applied to remove insignificant data between drugs and biological processes.

#### Connectivity map

A connectivity map was used to construct a drug-responsive process database. The connectivity map is a collection of genome-wide transcriptional expression data from cultured human cells treated with bioactive small molecules[3]. The connectivity map contains 6,100 expression profiles representing 1,309 compounds. The connectivity map provided rank information of probes for each sample. There were 22,283 probes and 6,100 samples in the rank matrix. Probe sets in ranked matrix were ranked in descending order of the ratio of the corresponding treatment-to-control values. Therefore, “top rank” means probes that are more highly upregulated than the control; “bottom rank” means probes that are more highly downregulated than the control. Top rank genes are positively affected by drugs, and bottom rank genes are negatively affected by drugs.

#### Gene Ontology

GO was used as a resource for biological processes. The GO project provided term definitions representing gene product properties in 3 categories [5]: cellular component, molecular function, and biological processes

#### Gene Set Enrichment Analysis

Gene Set Enrichment Analysis (GSEA) was used to show the relationship of processes to drugs. GSEA is a gene expression profile analysis technique used for finding the significance of a function, pathway, or GO category [6].

In this approach, gene sets S i = {1,…,n} are defined by GO terms and ranking information of each gene L j = {1,…,k} from the connectivity map. The ESs of each gene set were calculated in 6,100 samples. ESs of upregulated processes were calculated based on the ranked list; ESs of downregulated processes were calculated using the reversed ranked list.

[6]

C_ij_ is defined as a summing factor of a gene g_j_ that is drawn from L. N is the number of total genes in L, and N_s_ is the number of genes in the gene set S_i_.

Then the running sum Sum_ij_ for each sample against gene j is calculated using the following equation:

The ES for gene set i was calculated as follows:

ES is the maximum deviation from zero of Sum_ij_. For a randomly distributed gene set, S_i_, ES_i_ will be relatively small, but if it is concentrated at the top or the bottom of the list, or otherwise non-randomly distributed, then ES_i_ will be correspondingly high.

#### Process significance calculation

A t-score was used to show the significance of each process. To get a normalized t-score robust to outliers, the ESs were standardized with the median-MAD normalization method for each process [7]. ES_ij_ was used to denote an ES of process i = {1,2,…p} from sample j = {1,2,…,n}.

Both MED_i_ and MAD_i_ were used to represent the median, and the median absolute deviation of enrichment scores for biological process i. The scale factor of 1.4826 in the above equation was used to make MAD_i_ an estimator of σ.

### Drug-side effect network construction

#### Side effect resource (SIDER)

SIDER was developed to discover the relationships between side effects and drugs, and SIDER connects 888 drugs to 1,450 types of side effects [4]. It contains frequency of occurrence information between drugs and side effects for one-third of the drug-side effect pairs. (Table 1)

**Table 1:** Examples of SIDER information

STITCHID	UMLS concept ID	Effect name	Description of frequency	Frequency score
-1003	C0000737	Nausea	26%	0.26
-104741	C0010200	Cough	Postmarketing	0.001
-104865	C0015230	Rash	Rare	0.001
-115237	C0013604	Edema	Infrequent	0.01

A search tool for Interactions of chemicals (STITCH) ID is represented as a compound ID in STITCH databases. A unified medical language system (UMLS) concept ID implies a description of frequency that consists of 4 types: postmarketing, rare, infrequent, and frequent. For frequent cases, a percentage is used instead of the word “frequent.”

#### Drug-side effect network construction

Drug-side effect relationships available in SIDER are incomplete because side effects do not occur in gene expression data every time. Therefore, drug-side effect relationships appearing in SIDER needed to be filtered to find highly occurring relationships of gene expression data. Among the 120,598 common drug-side effect relationships in SIDER, however, only 15,672 relations have a frequency higher than 5%. Most relations had no information about frequency. Twenty percent was set as a threshold of frequency to find drug-side effect relationships (Additional file 1). Finally, 6,197 filtered relations were used to construct the drug-side effect network.

### Biological process-side effect network construction

**Figure 4:**
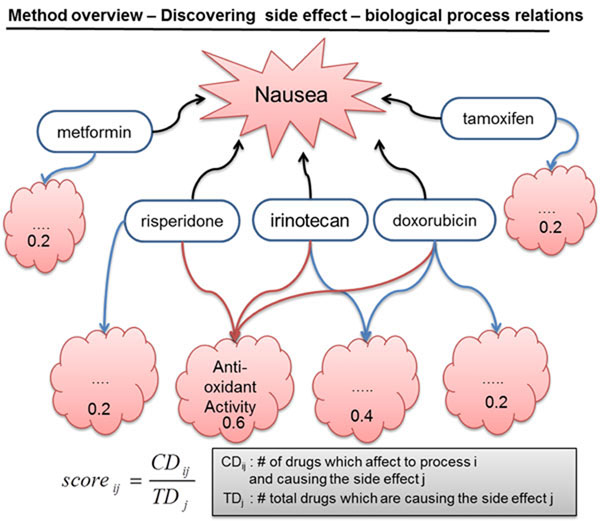
**Schematic diagram for discovering side effect-biological process relationships.** Nausea, which is the sensation of unease and discomfort in the stomach with an urge to vomit, is an example of a side effect. In this example, 3 of 5 drugs known to cause nausea are related to anti-oxidant activity, but the other processes were perturbed by only 1 or 2 drugs. Based on this connectivity, the scores were calculated to find possible processes causing the side effects. Finally, the processes were analyzed to ensure whether the side effect-biological process relationships revealed by this approach were meaningful.

Lastly, the biological process-side effect network was built. Figure 4 shows the method used for finding relationships between side effects and biological processes. The hypothesis used was that frequent responses to drugs causing the same side effect have higher probabilities of correlation with a side effect than less frequent responses.

#### Connecting drug-process and drug-side effect networks

To find relationships between biological processes and side effects, drug information was used as a bridge between the 2 networks, the drug-biological process network and the drug-side effect network. This can be represented as an ABC model consisting of A, biological processes; B, drugs; and C, side-effects. To merge the 2 networks, the drug names needed to be normalized because the connectivity map and SIDER use different drug identification. DrugBank was used to obtain normalized drug information for 1,494 FDA-approved drugs. The file “drugcards.zip” was downloaded from the DrugBank [8]. Three fields, i.e., drug ID, synonym, and brand names, were used to normalize drug names between the AB network and the BC network. Because of the small number of side effects with frequency information, only 74 drugs were included in both the AB and BC networks. Finally, using the 74 drugs with 168 effects and 2,209 processes network, data on 63,878 relationships were generated.

**Figure 5:**
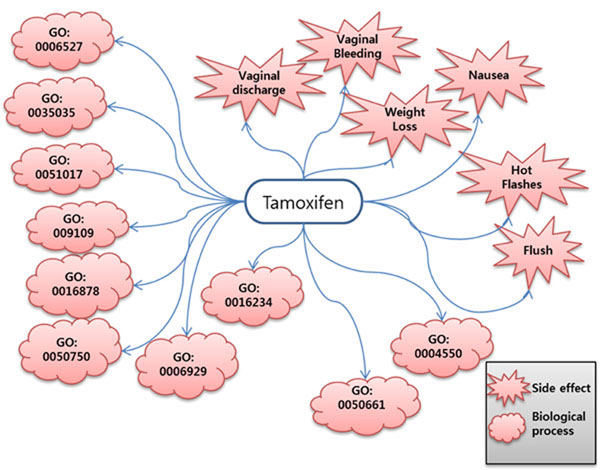
**Tamoxifen-mediated drug-process network and drug-side effect network.** Tamoxifen causes six types of side effects that are reported with a frequency of greater than 20%. We found 10 significant upregulated biological processes associated with tamoxifen (p < 0.001).

To illustrate the construction of the side effect-biological process network, the example of tamoxifen was used. Tamoxifen is one of drugs present in both the drug-process network and the drug-side effect network, and it is used as a mediator to connect the 2 networks (Figure 5).

#### Discovering side effect-related processes from the drug-process-side effect network

Co-occurrence-based scoring was used to determine how many drugs shared the same side effect in each process. A biological process that has a high co-occurrence score implies that the process is closely related to the targeted side effect; therefore, side effect data are only used when at least 2 drugs are related.

Score_ij_ was used to denote the co-occurrence score of a process i = {1,2,…n} in a side effect j = {1,2,…,n}. For each side effect i = {1,2,..n}, CD_ij_ is used to represent the number of drugs that have the co-occurring process i related to a side effect j, and TD_j_ is used to represent the number of total drugs related to the side effect j.

In the drug-process-side effect network, nausea is the most common side effect and is connected to 26 drugs. To investigate how many drugs with the same processes were significant, drug-side effect relations were randomly generated. The processes were determined by randomly selecting 74 drugs (2~26) for each side effect, repeated 1,000 times. The distribution was then determined using the number of related drugs on processes, and the processes with a p-value less than 0.05 were analyzed.

Table 2 shows the total number of drugs causing side effects and how many co-occurring drugs are significant in the total number of drugs. In the case of total drugs ranging from 2 to 5, co-significant processes in more than 2 drugs are significant to side effects.

**Table 2:** Side effect-related process threshold

Number of total drugs causing side effect	Co-occurrences (P < 0.05)
2,3,4,5	2
6,7,8,9,10,11, 12,14,15,16,17	3
19, 24, 25, 26	4

### Evaluation method

The constructed network was evaluated by examining the significance of relationships between biological processes and side effects provided by the network. The significance of relationships was measured by comparing biological processes represented by GO terms with the co-occurrence of GO terms and effect names appearing in PubMed records. The first and second steps were used to calculate the co-occurrence of effect names and GO terms. First, a set of PubMed records with an effect name was used as a query. The “[abstract/title]” qualifier was used in the PubMed search to ensure that effect names appeared in abstracts or titles. Secondly, because it is not easy to extract noun phrases from GO terms by using a simple exact string match, significant phrases were used. To this end, the following text-mining techniques were used: a conditional random field (CRF)-based sentence segmentation technique was used to parse abstracts [9], the sentence was tokenized with the part-of-speech (POS) technique using an extension of the Brill POS tagger [10], and noun phrase groups were extracted with a text chunking technique [11] that specialized in biomedical data collections. Thirdly, the extracted noun phrases were compared with GO terms, and the number of matched phrases was stored along with the phrases. The comparison between extracted phrases and GO terms was based on string similarity between the 2, and the shortest path-based edit distance (SPED) technique [12] was used. The SPED technique is a variation of Markov random field-based edit distance (MRFED) and calculates the shortest path between 2 selected vertices of a graph. Various thresholds were tested for string similarities, and the threshold was set at 0.55 since it gave the best performance. Table 3 shows the number of abstracts found in PubMed and the total GO terms evaluated for rash and urinary tract infection (UTI); 2,209 GO terms were utilized to calculate co-occurrence scores for evaluation.

**Table 3:** Datasets for evaluation

	Urinary tract infection	Rash
Abstracts	12,523	13,287
GO terms	2,209	2,209

## Results and discussion

The goodness of the discovered relations was confirmed using a survey of literature. First, the drug-biological process network was analyzed using the tamoxifen case study to show the significance of our method. Secondly, the ABC network model for A, processes; B, drugs; and C, side-effects was analyzed to find relationships between side effects and biological processes. Two case studies are used as examples to show the meaningfulness of the network. Finally, the performance of the network was evaluated by comparing the number of matched GO terms extracted by a text-mining method that was applied to a large number of PubMed abstracts.

### Drug-biological process network

The network connects 1,309 drugs to 3,629 GO terms with its ES. The GO terms are varied and some GO terms are too broad to interpret the relations; therefore, GO terms with less than 31 genes in human were chosen.  Highly relevant GO terms with a t-score greater than 3.0 (approximately p = 0.001) were also chosen. A positive association is more upregulated than the control; a negative association is more downregulated than the control.

#### Case study—Tamoxifen-related biological processes in the constructed network

For the case study of the drug-process network, tamoxifen was chosen because of its well-known mechanism. Tamoxifen is an antagonist of estrogen receptors in breast tissue [13].  We use 143th instance in connectivity map to find relationships between tamoxifen and its related processes in this case.

Table 4 shows significant processes related to tamoxifen in MCF7 cells (breast cancer cell line) using our method. The most significant GO term is nucleoside diphosphate kinase activity, and Neeman’s experiments support that nucleoside diphosphate is higher in the tamoxifen-treated cells [14]. Tamoxifen also upregulates low-density lipoprotein receptor binding according to Suarez’s study [15]. These results show that biological processes in our drug-biological upregulated process relationships are meaningful in drug response profiles.

**Table 4:** Upregulated tamoxifen-related processes in the drug-process network

GO	T-score	GO term
GO:0004550	4.14835	nucleoside diphosphate kinase activity
GO:0050661	3.38806	NADP or NADPH binding
GO:0016234	3.18592	inclusion body
GO:0006929	3.14893	substrate-bound cell migration
GO:0050750	3.12409	low-density lipoprotein receptor binding
GO:0016878	3.11266	acid-thiol ligase activity
GO:0009109	3.07065	coenzyme catabolic process
GO:0051017	3.04806	actin filament bundle formation
GO:0035035	3.00959	histone acetyltransferase binding
GO:0006527	3.0003	arginine catabolic process

Table 5 shows that there are 6 downregulated processes for tamoxifen. Translation elongation factor activity is highly related to tamoxifen in MCF-7 cells. As reported by Byun [16], translational elongation factor are underwent by tamoxifen. Cilium is known as cellular GPS, and is crucial to wound repair. For cilium, the peripheral loss of cilia function is reported in tamoxifen treats cell [17]. Tamoxifen reduced proteoglycan synthesis in an in vivo study [18]. Finally, Lahoute found that tamoxifen induced a loss of serum response factor (SRF), which induces downregulation of skeletal muscle fiber development [19]. These results confirm that biological processes in the drug-biological downregulated processes relationships are also meaningful in drug response profiles.

**Table 5:** Down regulated tamoxifen-related processes in the drug-process network

GO	T-score	GO term
GO:0003746	3.1047	Translation elongation factor activity
GO:0005929	3.20237	Cilium
GO:0019319	4.57807	Hexose biosynthetic process
GO:0030166	3.25123	Proteoglycan biosynthetic process
GO:0046364	4.97227	Monosaccaride biosyntheticprocess
GO:0048741	3.26341	Skeletal muscle fiber development

### Biological process-side effect network

**Figure 6:**
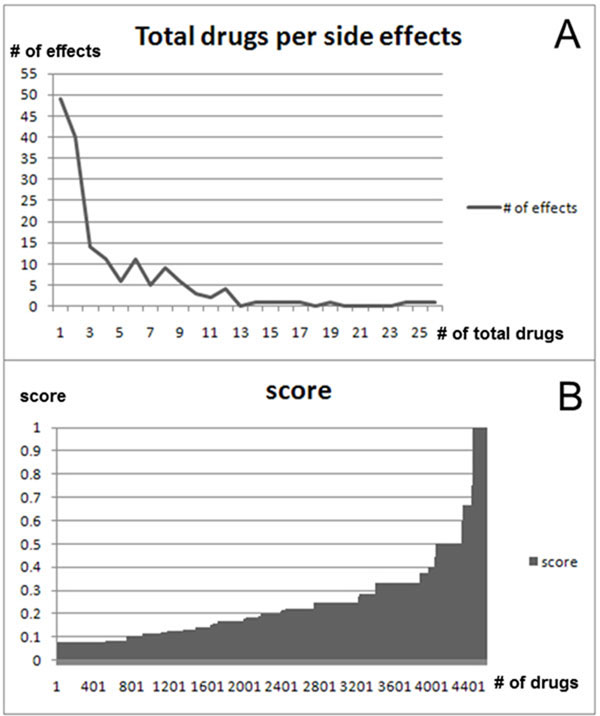
**Network statistics in drug upregulated biological process-side effect network**. Figure 6A shows the relationship between side effects and the total number of connected drugs in upregulated processes. The range of the total number of drugs is 1 to 26. It shows that 49 side effects occurred with only 1 drug, and 26 drugs caused nausea. Figure 6B shows that most scores of relations (about 88%) are less than 0.5. Half of relation scores are less than 0.2. Further, only 543 relation scores are greater than or equal to 0.5. This means that many significant processes are not over-represented among drugs. Therefore, a threshold needs to be determined to show which processes are highly related to which side effect (Table 2)

The biological process-side effect network contains 63,878 biological process-side effect pairs and covers a total of 168 side effects and 2,209 processes. In this network, there are 37,280 upregulated biological process-side effect pairs with a total of 168 side effects and 1,736 processes (Additional file 2). Furthermore, there are 26,598 downregulated biological process-side effect pairs, 168 side effects, and 1,430 processes (Additional file 3). Figure 6A shows the statistics of upregulated processes. To apply our algorithm, the side effects of more than 1 drug need to be considered. We finally used 119 effects and 744 processes with 4581 relations. (Figure 6)

#### Case study—Nausea-related biological processes in the biological processes-side effect network

In the case study of nausea, the most common cause is gastroenteritis or food poisoning, but nausea also frequently occurs as a medication side effect. Nausea is connected to 26 drugs in the drug-side effect network. For random sampling analysis, a score greater than or equal to 0.15 was considered significant (p < 0.05).

Table 6 shows 3 upregulated processes related to nausea. For example, Yoneyama et al found that adenosine deaminase activity (ADA) was related to hyperemesis gravidarum (vomiting and nausea) [20]. Chemotherapeutic agents induce oxidative damage in the gastrointestinal tract, causing nausea and vomiting; therefore, upregulated antioxidant activity is needed to reduce oxidative damages [21]. Also, nausea occurs when blood sugar rises rapidly [22], and the cellular carbohydrate catabolic process is noted for increasing the blood sugar level in the body.

**Table 6:** Nausea-related upregulated processes

GO	Co-occurrence score	GO term
GO:0019239	0.19	Deaminase activity
GO:0016209	0.19	Antioxidant activity
GO:0044275	0.19	Cellular carbohydrate catabolic process

Table 7 shows downregulated processes that are related to nausea. In human studies, treatment with cytokines is often accompanied by nausea [23]. Synaptic vesicle endocytosis may subsequently be used for neurotransmitter storage [5]. Neurotransmitters are also involved in relaying messages of nausea and vomiting [24].

**Table 7:** Nausea-related downregulated processes

GO	Co-occurrences score	GO term
GO:0002718	0.15	Regulation of cytokine production during immune response
GO:0046631	0.15	Alpha-beta T-cell activation
GO:0070410	0.15	Co-SMAD binding
GO:0016863	0.15	Intramolecular oxidoreductase activity
GO:0048488	0.15	Synaptic vesicle endocytosis

#### Case study—Anemia-related biological processes in the biological processes-side effect network

Anemia is known as deficiency of hemoglobin, which is a molecular substance inside red blood cells. As hemoglobin transfers oxygen from lungs to the tissues, anemia makes hypoxia in tissues. Anemia is connected to 10 drugs in the drug-side effect network. A random sampling analysis score greater than or equal to 0.3 was considered significant (p < 0.05).

Table 8 shows anemia-related upregulated processes. Cytochrome b5 reductase is an enzyme in the blood. This enzyme regulates the iron in red blood cells and helps the oxygen transportation. Therefore, cytochrome b5 reductase is highly related to anemia. Antioxidant activity of blood serum is highly related to anemia [25]. Anemia search results are similar to those of nausea (GO:0016209, GO:0044275) because 8 of 10 drugs causing anemia also cause nausea.

**Table 8:** Anemia related up-regulated processes

GO	Co-occurrences based score	GO term
GO:0016209	0.3	Antioxidant activity
GO:0005852	0.5	Eukaryotic translation initiation factor 3 complex
GO:0004128	0.3	Cytochrome-b5 reductase activity
GO:0044275	0.3	Cellular carbohydrate catabolic process

Table 9 shows downregulated processes related to anemia. Regulation of cytokine production during immune response was related to anemia in a previous study [26]. Iron deficiency induces anemia and neurotransmitter deficiency. Synaptic vesicle endocytosis may subsequently be used for neurotransmitter storage [5]. Downregulated activity of synaptic vesicle endocytosis induces neurotransmitter deficiency.

**Table 9:** Anemia related down-regulated processes

GO	Co-occurrences score	GO term
GO:0070410	0.3	Co-SMAD binding
GO:0002718	0.3	Regulation of cytokine production during immune response
GO:0048488	0.3	Synaptic vesicle endocytosis

### Evaluation result

**Figure 7:**
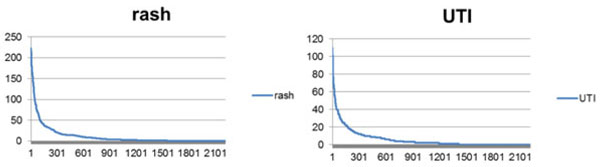
**Literature-based co-occurrence score distribution of 2 side effects.** Top 20 processes are omitted in this graph because of range problem

Two different side effects, i.e., rash, and UTI, were used for evaluation by retrieving PubMed records for each side effect and calculating the co-occurrence scores for each GO term. Figure 7 shows the co-occurrence scores for each GO term for 2 cases. To evaluate the significance of discovered biological processes, the top 10%, 20%, 30%, 40%, and 50% scores in the distribution were selected, as shown in Figure 7. This threshold was used to examine the significance of the processes in each top n%.

**Figure 8:**
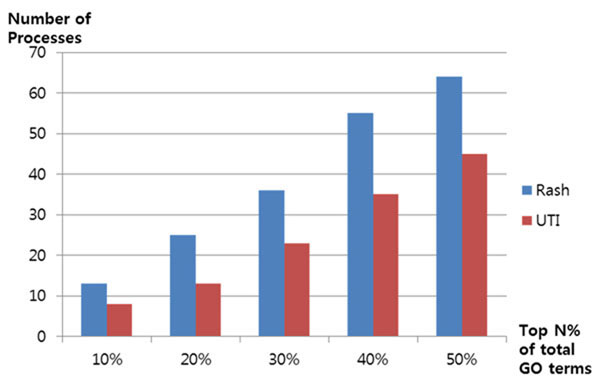
**The number of processes matched with text-mining results for rash, and UTI**. The x axis is the top n% of co-occurred GO terms with biological processes (total 2,209). The y axis is the number of processes with scores greater than the top n% (x axis) threshold of the total process scores

Figure 8 shows the number of matched terms between our approach and the results of the text-mining method for GO terms extracted from PubMed.

For rash, our method showed 116 GO-related terms. Of 116 processes, 13 were found in the top 10% of the text-mining results, 25 processes were found in the top 20% of the results, 36 processes were found in the top 30% of the results, 55 processes were found in the top 40% of the results, and 64 processes were in the top 50% of the results. For UTI, our method shows 76 GO-related terms. Of 76 processes, 8 were found in the top 10% of the results, 13 were found in the top 20% of the results, 23 were found in the top 30% of the text-mining results, 35 were found in the top 40% of the results, and 45 processes were in the top 50% of the results.

**Figure 9:**
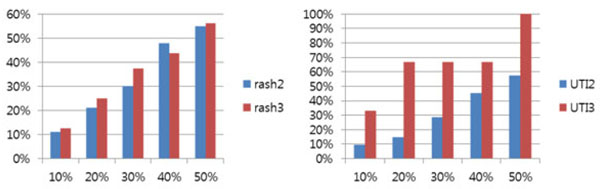
**Evaluation of our hypothesis for rash and UTI**. The x axis is the top n% for the total process scores. The y axis is percentage of processes with scores greater than the top n% (x axis) threshold of the total processes scores.

It was assumed that more frequent responsive processes to drugs causing the same side effect have higher probabilities of correlation with a side effect than less frequent responsive processes. The hypothesis was tested with rash and UTI cases. In Figure 9A, the rash2 bar (blue) includes less frequent response processes, and the rash3 bar (red) includes only significant frequent response processes. For the rash2 bar, we found 100 related processes. Eleven processes (11%) were found in the top 10% of the text-mining results, 21 (21%) were in the top 20% of the results, 30 (30%) were in the top 30% of the results, 48 (48%) were in the top 40% of the results, and 55 processes (55%) were in the top 50% of the results. The rash3 bar shows 16 significant frequent response processes by drugs. Two processes (13%) were in the top 10%, 4 (25%) were in the top 20%, 6 (38%) were in the top 30%, 7 (44%) were in the top 40%, and 9 processes (56%) were in the top 50%. For all results, except 40%, rash3 performs better than rash2 in terms of the proportion of processes discovered over the top n ranked processed (Fig. 9A).

In Figure 9B, the UTI2 bar (blue) includes less frequent response processes, and the UTI3 bar (red) only includes significant frequent response processes. For the UTI2 bar, our method found 73 related processes. Seven processes (10%) were found in the top 10%, 11 (15%) were found in the top 20% of the results, 21 (29%) were found in the top 30%, 33 (45%) were found in the top 40%, and 42 processes (58%) were found in the top 50%. As indicated by the UTI3 bar, our method found 3 frequent response processes by drugs. One process (33%) was found in the top 10%, 2 processes (67%) in the top 20%, 30%, and 40%, and 3 processes (100%) in the top 50% of the results. This shows that UTI3 performed better than UTI2 in all 5 cases (Fig. 9B) and confirms that our method was able to find relationships between biological processes and side effects.

## Conclusions

In this paper, we proposed a new approach for automatically discovering relationships between biological processes and side effects using the co-occurrence based multi-level network. We built the drug-biological process network, and showed that our method can be used to discover drug related significant processes (as shown in the example of tamoxifen). In addition, we built an ABC Model (using A, biological processes; B, drugs; and C, side effect information) for 74 drugs, 168 side effects, and 2,209 biological processes. A literature analysis confirmed that relations between side effects and biological processes found by co-occurrence were meaningful. In addition, our method was evaluated using a text-mining technique to extract co-occurring GO terms with effects. The results showed that our method is efficient and useful for finding relationships between biological processes and side effects.

In a future study, the scoring scheme will be improved because the current scoring algorithm considers all drugs equally regardless of the number of side effects or the number of biological processes associated with them. For example, drug A has only 1 side effect (s-1), whereas drug B has 2 side effects (s1 and s2), with all other settings the same, including association with biological process (p). In this case, drug A provides more reliable information on the association of s1and p than drug B. However, the proposed scoring scheme cannot reflect this, thus causing a loss of information for a more accurate association. We also plan to investigate whether biological processes related to side effects are valuable resources in elucidating the mechanism of drug effects. Instead of using the text-mining technique, a manual evaluation will be conducted to identify undiscovered relationships from process-side effect pairs that are not mentioned in literature. In addition, we are interested in a research on personalized drug responsive expression data by applying multi-level networks for personalized medicine. By exploring the relationship between drugs and phenotypes on the multi-level network, we will be able to understand the mechanisms underlying drug involvement in the human body.

## Competing interests

The authors declare that they have no competing interests.

## Authors' contributions

LS designed the method and drafted the manuscript along with MS. MS also critically revised the manuscript for important intellectual content. KHL and DL supervised the work and gave final approval of the version of the manuscript to be submitted.

## Supplementary Material

Additional file 1 This file contains common drug names and related side effect names which are reported with frequency of greater than 20% from SIDER. First Column: Drug Bank ID Second Column: Drug name Third Column: Effect ID ( UMLS Concept ID) Fourth Column: Effect name Click here for file

Additional file 2 This file contains up_regulated processes (T-score > 3.0) and related effects. First Column: Effect ID ( UMLS Concept ID) Second Column: Process ID ( Gene Ontology ID) Third Column: The number of drugs which affect to process and causing the side effect. Fourth Column: Total drugs which are causing the side effect. Click here for file

Additional file 3 This file contains down_regulated processes (T-score > 3.0) and related effects. First Column: Effect ID ( UMLS Concept ID) Second Column: Process ID ( Gene Ontology ID) Third Column: The number of drugs which affect to process and causing the side effect. Fourth Column: Total drugs which are causing the side effect. Click here for file
