# Supplementary material for: Building the process-drug–side effect network to discover the relationship between biological Processes and side effects
Source: BMC Bioinformatics. 2011 Mar 29;12(Suppl 2):S2. doi: 10.1186/1471-2105-12-S2-S2 (PMC3073182; doi:10.1186/1471-2105-12-S2-S2)
Supplement: Additional file 1 — This file contains common drug names and related side effect names which are reported with frequency of greater than 20% from SIDER. First Column: Drug Bank ID Second Column: Drug name Third Column: Effect ID ( UMLS Concept ID) Fourth Column: Effect name [file 1471-2105-12-S2-S2-S1.doc]

DB00331 metformin C0011991 Diarrhea

DB00331 metformin C0027497 Nausea

DB00331 metformin C0042963 Vomiting

DB00816 metaproterenol C0027769 Nervousness

DB00787 acyclovir C0018681 Headache

DB01043 memantine C0002871 anemia

DB01043 memantine C0004134 ataxia

DB01043 memantine C0007787 transient ischemic attack

DB01043 memantine C0009763 conjunctivitis

DB01043 memantine C0015230 rash

DB01043 memantine C0018801 cardiac failure

DB01043 memantine C0032285 pneumonia

DB01043 memantine C0038454 cerebrovascular accident

DB01043 memantine C0039070 syncope

DB01043 memantine C0042571 vertigo

DB01043 memantine C0086543 cataract

DB00382 THA C0003862 arthralgia

DB00382 THA C0003864 arthritis

DB00382 THA C0006277 bronchitis

DB00382 THA C0009763 Conjunctivitis

DB00382 THA C0009951 Convulsions

DB00382 THA C0013404 dyspnea

DB00382 THA C0015967 fever

DB00382 THA C0020538 hypertension

DB00382 THA C0020649 Hypotension

DB00382 THA C0027769 Nervousness

DB00382 THA C0030554 paresthesia

DB00382 THA C0031350 Pharyngitis

DB00382 THA C0032285 pneumonia

DB00382 THA C0037199 sinusitis

DB00382 THA C0039070 syncope

DB00382 THA C0042571 vertigo

DB00382 THA C0085593 Chill

DB00382 THA C0085649 peripheral edema

DB00382 THA C0231218 malaise

DB00382 THA C0700590 Sweating increased

DB01006 letrozole C0003862 Arthralgia

DB01006 letrozole C0003864 Arthritis

DB01006 letrozole C0016382 Flushes

DB01006 letrozole C0020040 Hot Flashes

DB01006 letrozole C0151825 Bone Pain

DB01006 letrozole C0030552 Weakness

DB01097 leflunomide C0011991 Diarrhea

DB01097 leflunomide C0018681 Headache

DB01097 leflunomide C0021311 Infection

DB00270 isradipine C0018681 Headache

DB00270 isradipine C0013604 Edema

DB01247 isocarboxazid C0012833 Dizziness

DB00762 irinotecan C0002170 Alopecia

DB00762 irinotecan C0002871 Anemia

DB00762 irinotecan C0003123 Anorexia

DB00762 irinotecan C0004093 Asthenia

DB00762 irinotecan C0009806 Constipation

DB00762 irinotecan C0011991 Diarrhea

DB00762 irinotecan C0013404 Dyspnea

DB00762 irinotecan C0015967 Fever

DB00762 irinotecan C0023530 Leukopenia

DB00762 irinotecan C0027497 Nausea

DB00762 irinotecan C0027947 Neutropenia

DB00762 irinotecan C0030193 Pain

DB00762 irinotecan C0042963 Vomiting

DB00997 doxorubicin C0002170 Alopecia

DB00997 doxorubicin C0002453 Amenorrhea

DB00997 doxorubicin C0002871 Anemia

DB00997 doxorubicin C0011991 Diarrhea

DB00997 doxorubicin C0020040 Hot flashes

DB00997 doxorubicin C0021311 Infection

DB00997 doxorubicin C0023530 Leukopenia

DB00997 doxorubicin C0027497 Nausea

DB00997 doxorubicin C0027947 Neutropenia

DB00997 doxorubicin C0040034 Thrombocytopenia

DB00997 doxorubicin C0042963 vomiting

DB00997 doxorubicin C0004093 Asthenia

DB00997 doxorubicin C0015230 Rash

DB00997 doxorubicin C0009763 Conjunctivitis

DB00997 doxorubicin C0022568 keratitis

DB00495 zidovudine C0003123 Anorexia

DB00495 zidovudine C0018681 Headache

DB00495 zidovudine C0027497 Nausea

DB00495 zidovudine C0231218 Malaise

DB00495 zidovudine C0015967 Fever

DB00715 paroxetine C0010200 Cough

DB00715 paroxetine C0020538 Hypertension

DB00715 paroxetine C0027497 Nausea

DB00715 paroxetine C0030193 pain

DB00715 paroxetine C0033774 Pruritus

DB00715 paroxetine C0035455 rhinitis

DB00715 paroxetine C0039070 syncope

DB00715 paroxetine C0039231 tachycardia

DB00715 paroxetine C0042571 vertigo

DB00715 paroxetine C0042963 vomiting

DB00715 paroxetine C0043094 Weight gain

DB00715 paroxetine C0043096 weight loss

DB00715 paroxetine C0231218 Malaise

DB00715 paroxetine C0235198 concentration impaired

DB00715 paroxetine C0003862 arthralgia

DB00715 paroxetine C0040264 tinnitus

DB00715 paroxetine C0085593 chills

DB00715 paroxetine C0002622 amnesia

DB00715 paroxetine C0037199 sinusitis

DB00715 paroxetine C0013144 Somnolence

DB00715 paroxetine C0043352 Dry Mouth

DB00715 paroxetine C0018681 Headache

DB00864 tacrolimus C0003862 Arthralgia

DB00864 tacrolimus C0020538 Hypertension

DB00864 tacrolimus C0021400 Flu

DB00864 tacrolimus C0033774 Pruritus

DB00864 tacrolimus C1527304 Allergic Reaction

DB01018 guanfacine C0013144 Drowsiness

DB01018 guanfacine C0013144 Somnolence

DB01018 guanfacine C0043352 Dry Mouth

DB00273 topiramate C0002871 anemia

DB00273 topiramate C0003862 Arthralgia

DB00273 topiramate C0009763 conjunctivitis

DB00273 topiramate C0018524 hallucination

DB00273 topiramate C0021116 impotence

DB00273 topiramate C0033975 psychosis

DB00273 topiramate C0038663 suicide attempt

DB00273 topiramate C0039070 syncope

DB00273 topiramate C0011175 dehydration

DB00273 topiramate C0011991 diarrhea

DB00273 topiramate C0013428 dysuria

DB00273 topiramate C0015967 fever

DB00273 topiramate C0019112 hemorrhoids

DB00273 topiramate C0022650 renal calculus

DB00273 topiramate C0040264 tinnitus

DB00273 topiramate C0042109 urticaria

DB00273 topiramate C0042963 vomiting

DB00273 topiramate C0151786 muscle weakness

DB00273 topiramate C0702166 acne

DB00273 topiramate C0002170 alopecia

DB00273 topiramate C0002453 amenorrhea

DB00273 topiramate C0006277 bronchitis

DB00273 topiramate C0009951 convulsions

DB00273 topiramate C0010200 coughing

DB00273 topiramate C0011124 libido decreased

DB00273 topiramate C0015672 fatigue

DB00273 topiramate C0025323 menorrhagia

DB00273 topiramate C0025874 intermenstrual bleeding

DB00273 topiramate C0029456 osteoporosis

DB00273 topiramate C0030252 palpitation

DB00273 topiramate C0031212 personality disorder

DB00273 topiramate C0042029 urinary tract infection

DB00273 topiramate C0042267 vaginitis

DB00273 topiramate C0042571 vertigo

DB00273 topiramate C0043094 weight increase

DB00273 topiramate C0231218 malaise

DB00273 topiramate C0677481 micturition frequency

DB00273 topiramate C0917801 insomnia

DB00674 galantamine C0008031 chest pain

DB00674 galantamine C0016204 flatulence

DB00674 galantamine C0042024 incontinence

DB00674 galantamine C0004093 asthenia

DB00674 galantamine C0015967 fever

DB00674 galantamine C0231218 malaise

DB00674 galantamine C0027497 Nausea

DB00674 galantamine C0020538 hypertension

DB00684 tobramycin C0000737 Abdominal Pain

DB00684 tobramycin C0003123 Anorexia

DB00684 tobramycin C0004093 Asthenia

DB00684 tobramycin C0004096 Asthma

DB00684 tobramycin C0008031 Chest Pain

DB00684 tobramycin C0010200 Cough

DB00684 tobramycin C0013404 Dyspnea

DB00684 tobramycin C0015967 Fever

DB00684 tobramycin C0018681 Headache

DB00684 tobramycin C0019079 Hemoptysis

DB00684 tobramycin C0024115 Lung Disorder

DB00684 tobramycin C0031350 Pharyngitis

DB00684 tobramycin C0035455 Rhinitis

DB00684 tobramycin C0042963 Vomiting

DB00996 gabapentin C0003123 anorexia

DB00996 gabapentin C0003467 anxiety

DB00996 gabapentin C0003862 arthralgia

DB00996 gabapentin C0004093 asthenia

DB00996 gabapentin C0013604 edema

DB00996 gabapentin C0016204 flatulence

DB00996 gabapentin C0017574 gingivitis

DB00996 gabapentin C0020538 hypertension

DB00996 gabapentin C0030554 paresthesia

DB00996 gabapentin C0032285 neumonia

DB00996 gabapentin C0034150 purpura

DB00996 gabapentin C0042571 vertigo

DB00996 gabapentin C0042798 abnormal vision

DB00996 gabapentin C0231218 malaise

DB00996 gabapentin C0032285 pneumonia

DB00996 gabapentin C0009676 confusion

DB00996 gabapentin C0012833 Dizziness

DB00996 gabapentin C0013144 Somnolence

DB00176 fluvoxamine C0013144 Somnolence

DB00176 fluvoxamine C0018681 Headache

DB00176 fluvoxamine C0027497 Nausea

DB00176 fluvoxamine C0917801 Insomnia

DB00176 fluvoxamine C0004093 Asthenia

DB00176 fluvoxamine C0002622 amnesia

DB00176 fluvoxamine C0010200 cough

DB00176 fluvoxamine C0013604 edema

DB00176 fluvoxamine C0020538 hypertension

DB00176 fluvoxamine C0020649 hypotension

DB00176 fluvoxamine C0037199 sinusitis

DB00176 fluvoxamine C0039070 syncope

DB00176 fluvoxamine C0039231 tachycardia

DB00176 fluvoxamine C0043094 weight gain

DB00176 fluvoxamine C0043096 weight loss

DB00176 fluvoxamine C0231218 malaise

DB00176 fluvoxamine C0000737 Abdominal pain

DB00176 fluvoxamine C0003123 Anorexia

DB00176 fluvoxamine C0003467 anxiety

DB00176 fluvoxamine C0009806 constipation

DB00176 fluvoxamine C0011991 diarrhea

DB00176 fluvoxamine C0012833 dizziness

DB00176 fluvoxamine C0013395 dyspepsia

DB00176 fluvoxamine C0027769 nervousness

DB00176 fluvoxamine C0030252 Palpitation

DB00176 fluvoxamine C0040822 tremor

DB00176 fluvoxamine C0042571 vertigo

DB00176 fluvoxamine C0042963 vomiting

DB00176 fluvoxamine C0043352 dry mouth

DB00176 fluvoxamine C0085631 Agitation

DB00176 fluvoxamine C0700590 Sweating increased

DB00499 flutamide C0011124 Loss of Libido

DB00499 flutamide C0020040 Hot Flashes

DB00499 flutamide C0021116 Impotence

DB00472 fluoxetine C0002622 amnesia

DB00472 fluoxetine C0009676 confusion

DB00472 fluoxetine C0013456 ear pain

DB00472 fluoxetine C0019080 hemorrhage

DB00472 fluoxetine C0020538 hypertension

DB00472 fluoxetine C0027497 nausea

DB00472 fluoxetine C0040264 tinnitus

DB00472 fluoxetine C0042963 vomiting

DB00472 fluoxetine C0043094 weight gain

DB00472 fluoxetine C0085593 chills

DB00472 fluoxetine C0085631 agitation

DB00472 fluoxetine C0677481 urinary frequency

DB00472 fluoxetine C0851578 sleep disorder

DB00472 fluoxetine C0008031 chest pain

DB00472 fluoxetine C0030252 palpitation

DB00472 fluoxetine C0030554 paresthesia

DB00472 fluoxetine C0004093 Asthenia

DB00472 fluoxetine C0917801 Insomnia

DB00472 fluoxetine C0018681 Headache

DB00472 fluoxetine C0035455 Rhinitis

DB00472 fluoxetine C0003467 anxiety

DB00472 fluoxetine C0003862 joint pain

DB00472 fluoxetine C0004604 back pain

DB00472 fluoxetine C0006277 bronchitis

DB00472 fluoxetine C0011991 diarrhea

DB00472 fluoxetine C0012833 dizziness

DB00472 fluoxetine C0013144 drowsiness

DB00472 fluoxetine C0013390 painful menstruation

DB00472 fluoxetine C0015230 rash

DB00472 fluoxetine C0015672 fatigue

DB00472 fluoxetine C0021116 impotence

DB00472 fluoxetine C0027769 nervousness

DB00472 fluoxetine C0033774 pruritus

DB00472 fluoxetine C0033953 sexual dysfunction

DB00472 fluoxetine C0040822 tremor

DB00472 fluoxetine C0042029 urinary tract infection

DB00472 fluoxetine C0042798 abnormal vision

DB00472 fluoxetine C0043096 weight loss

DB00472 fluoxetine C0220870 lightheadedness

DB00472 fluoxetine C0231528 muscle pain

DB00472 fluoxetine C0700590 excessive sweating

DB00472 fluoxetine C0220870 light headedness

DB00472 fluoxetine C0013144 Somnolence

DB01041 thalidomide C0004093 Asthenia

DB01041 thalidomide C0013144 Somnolence

DB01041 thalidomide C0014130 Endocrine Disorders

DB01041 thalidomide C0015230 Rash

DB01041 thalidomide C0015967 Fever

DB01041 thalidomide C0023530 Leukopenia

DB00624 testosterone C0033774 pruritus

DB00675 tamoxifen C0002453 Amenorrhea

DB00675 tamoxifen C0016382 Flush

DB00675 tamoxifen C0020040 Hot Flashes

DB00675 tamoxifen C0027497 Nausea

DB00675 tamoxifen C0043096 Weight Loss

DB00675 tamoxifen C0151706 Vaginal Bleeding

DB00675 tamoxifen C0227791 Vaginal Discharge

DB00675 tamoxifen C0227791 Vaginal Discharges

DB01117 atovaquone C0000737 Abdominal pain

DB01117 atovaquone C0004093 Asthenia

DB01117 atovaquone C0010200 Cough

DB01117 atovaquone C0011991 Diarrhea

DB01117 atovaquone C0013404 Dyspnea

DB01117 atovaquone C0015230 Rash

DB01117 atovaquone C0015967 Fever

DB01117 atovaquone C0018681 Headache

DB01117 atovaquone C0020625 Hyponatremia

DB01117 atovaquone C0021311 Infection

DB01117 atovaquone C0027497 Nausea

DB01117 atovaquone C0035455 Rhinitis

DB01117 atovaquone C0042963 Vomiting

DB01223 aminophylline C0000737 Abdominal Pain

DB01223 aminophylline C0020456 Hyperglycemia

DB01223 aminophylline C0020621 Hypokalemia

DB01223 aminophylline C0020649 Hypotension

DB01223 aminophylline C0027769 Nervousness

DB01223 aminophylline C0036974 shock

DB01223 aminophylline C0039239 Sinus tachycardia

DB01223 aminophylline C0039240 supraventricular tachycardias

DB01223 aminophylline C0040822 Tremors

DB01223 aminophylline C0042963 Vomiting

DB01223 aminophylline C0151636 Ventricular premature beats

DB01223 aminophylline C0003811 arrhythmias

DB00641 simvastatin C0009806 Constipation

DB00619 imatinib C0000737 Abdominal Pain

DB00619 imatinib C0002871 Anemia

DB00619 imatinib C0003862 Arthralgia

DB00619 imatinib C0004093 Asthenia

DB00619 imatinib C0010200 Cough

DB00619 imatinib C0011991 Diarrhea

DB00619 imatinib C0013395 Dyspepsia

DB00619 imatinib C0013404 Dyspnea

DB00619 imatinib C0013604 Edema

DB00619 imatinib C0015230 Skin Rash

DB00619 imatinib C0015672 Fatigue

DB00619 imatinib C0015967 Pyrexia

DB00619 imatinib C0018681 Headache

DB00619 imatinib C0019080 Hemorrhage

DB00619 imatinib C0026821 Muscle Cramps

DB00619 imatinib C0026858 Musculoskeletal Pain

DB00619 imatinib C0027441 Nasopharyngitis

DB00619 imatinib C0027497 Nausea

DB00619 imatinib C0027947 Neutropenia

DB00619 imatinib C0040034 Thrombocytopenia

DB00619 imatinib C0042963 Vomiting

DB00619 imatinib C0043094 Weight Increased

DB00619 imatinib C0231528 Myalgia

DB00254 doxycycline C0009443 Common Cold

DB00254 doxycycline C0018681 Headache

DB01037 selegiline C0002622 amnesia

DB01037 selegiline C0003123 anorexia

DB01037 selegiline C0006277 bronchitis

DB01037 selegiline C0007859 neck pain

DB01037 selegiline C0008031 Chest pain

DB01037 selegiline C0009806 Constipation

DB01037 selegiline C0010200 Cough

DB01037 selegiline C0013390 dysmenorrhea

DB01037 selegiline C0013491 Ecchymosis

DB01037 selegiline C0016204 flatulence

DB01037 selegiline C0016663 pathological fracture

DB01037 selegiline C0017160 gastroenteritis

DB01037 selegiline C0020538 Hypertension

DB01037 selegiline C0025874 metrorrhagia

DB01037 selegiline C0030554 paresthesia

DB01037 selegiline C0033774 Pruritus

DB01037 selegiline C0038990 sweating

DB01037 selegiline C0040264 tinnitus

DB01037 selegiline C0042029 Urinary tract infection

DB01037 selegiline C0042963 vomiting

DB01037 selegiline C0085631 Agitation

DB01037 selegiline C0085649 Peripheral edema

DB01037 selegiline C0231528 Myalgia

DB01037 selegiline C0677481 urinary frequency

DB01037 selegiline C0702166 acne

DB00773 etoposide C0002170 Alopecia

DB00773 etoposide C0002871 Anemia

DB00773 etoposide C0023530 Leukopenia

DB00773 etoposide C0027497 Nausea

DB00773 etoposide C0040034 Thrombocytopenia

DB00773 etoposide C0042963 vomiting

DB00773 etoposide C0004093 Asthenia

DB00773 etoposide C0015967 Fever

DB00773 etoposide C0027947 Neutropenia

DB00773 etoposide C0085593 Chills

DB00773 etoposide C0231218 Malaise

DB00091 cyclosporine C0019572 Hirsutism

DB00091 cyclosporine C0020538 Hypertension

DB00091 cyclosporine C0040822 Tremor

DB00091 cyclosporine C0042029 Urinary Tract Infections

DB00091 cyclosporine C0021311 Infection

DB00091 cyclosporine C0031117 Peripheral Nervous System Disorders

DB00908 quinidine C0011991 diarrhea

DB00586 diclofenac C0000737 Abdominal pain

DB00734 risperidone C0000737 abdominal pain

DB00734 risperidone C0002453 amenorrhea

DB00734 risperidone C0002871 anemia

DB00734 risperidone C0003467 anxiety

DB00734 risperidone C0003862 arthralgia

DB00734 risperidone C0004093 asthenia

DB00734 risperidone C0004604 back pain

DB00734 risperidone C0008031 chest pain

DB00734 risperidone C0013404 dyspnea

DB00734 risperidone C0015230 rash

DB00734 risperidone C0020649 hypotension

DB00734 risperidone C0027497 nausea

DB00734 risperidone C0027769 nervousness

DB00734 risperidone C0030193 pain

DB00734 risperidone C0033975 psychosis

DB00734 risperidone C0039231 tachycardia

DB00734 risperidone C0042024 urinary incontinence

DB00734 risperidone C0042963 vomiting

DB00734 risperidone C0085631 agitation

DB00734 risperidone C0151825 skeletal pain

DB00734 risperidone C0003123 anorexia

DB00734 risperidone C0015672 fatigue

DB00734 risperidone C0025323 menorrhagia

DB00734 risperidone C0032617 polyuria

DB00734 risperidone C0037036 salivation

DB00734 risperidone C0085602 polydipsia

DB00734 risperidone C0349506 photosensitivity

DB00734 risperidone C0013428 dysuria

DB00734 risperidone C0018965 hematuria

DB00734 risperidone C0004134 ataxia

DB00734 risperidone C0009806 Constipation

DB00734 risperidone C0013144 Somnolence

DB00734 risperidone C0037036 Saliva increased

DB00734 risperidone C0040822 tremor

DB00734 risperidone C0041912 Upper respiratory tract infection

DB00734 risperidone C0575081 abnormal gait

DB00734 risperidone C0917801 Insomnia

DB00734 risperidone C0010200 Coughing

DB00734 risperidone C0035455 Rhinitis

DB00740 riluzole C0040822 tremor

DB00740 riluzole C0085631 Agitation

DB00740 riluzole C0004093 Asthenia

DB00740 riluzole C0027497 Nausea

DB00084 ribavirin C0000737 Abdominal Pain

DB00084 ribavirin C0003123 Anorexia

DB00084 ribavirin C0004093 Asthenia

DB00084 ribavirin C0011991 Diarrhea

DB00084 ribavirin C0012833 Dizziness

DB00084 ribavirin C0013395 Dyspepsia

DB00084 ribavirin C0015672 Fatigue

DB00084 ribavirin C0015967 Fever

DB00084 ribavirin C0016382 Flushing

DB00084 ribavirin C0018681 Headache

DB00084 ribavirin C0027497 Nausea

DB00084 ribavirin C0027947 Neutropenia

DB00084 ribavirin C0042963 Vomiting

DB00084 ribavirin C0043096 Weight Decrease

DB00084 ribavirin C0700590 Sweating Increased

DB00084 ribavirin C0002170 Alopecia

DB00084 ribavirin C0003467 Anxiety

DB00084 ribavirin C0003862 Arthralgia

DB00084 ribavirin C0010200 Coughing

DB00084 ribavirin C0013404 Dyspnea

DB00084 ribavirin C0015230 Rash

DB00084 ribavirin C0021400 Influenza

DB00084 ribavirin C0026858 Musculoskeletal Pain

DB00084 ribavirin C0033774 Pruritus

DB00084 ribavirin C0151908 Skin Dry

DB00084 ribavirin C0231528 Myalgia

DB00084 ribavirin C0235198 Concentration Impaired

DB00084 ribavirin C0917801 Insomnia

DB00481 raloxifene C0020040 Hot Flashes

DB00481 raloxifene C0024902 Breast Pain

DB00481 raloxifene C0151706 Vaginal Bleeding

DB00694 daunorubicin C0000737 Abdominal Pain

DB00694 daunorubicin C0002170 Alopecia

DB00694 daunorubicin C0003123 Anorexia

DB00694 daunorubicin C0010200 Cough

DB00694 daunorubicin C0011991 Diarrhea

DB00694 daunorubicin C0013404 Dyspnea

DB00694 daunorubicin C0015672 Fatigue

DB00694 daunorubicin C0015967 Fever

DB00694 daunorubicin C0018681 Headache

DB00694 daunorubicin C0021311 Infections

DB00694 daunorubicin C0027497 Nausea

DB00694 daunorubicin C0027947 Neutropenia

DB00694 daunorubicin C0042963 Vomiting

DB00694 daunorubicin C0442874 Neuropathy

DB00694 daunorubicin C1527304 Allergic Reactions

DB00924 cyclobenzaprine C0013144 Drowsiness

DB00924 cyclobenzaprine C0043352 Dry Mouth

DB00924 cyclobenzaprine C0012833 dizziness

DB00924 cyclobenzaprine C0013395 dyspepsia

DB00924 cyclobenzaprine C0015672 fatigue

DB00924 cyclobenzaprine C0027497 nausea

DB00924 cyclobenzaprine C0030552 weakness

DB00924 cyclobenzaprine C0030554 paresthesia

DB00924 cyclobenzaprine C0039231 Increased heart rate

DB00924 cyclobenzaprine C0039231 tachycardia

DB00924 cyclobenzaprine C0344232 blurred vision

DB00924 cyclobenzaprine C0917801 insomnia

DB01182 propafenone C0011991 Diarrhea

DB00396 progesterone C0030193 pain

DB00396 progesterone C0012833 Dizziness

DB00396 progesterone C0009806 Constipation

DB00396 progesterone C0013144 Somnolence

DB00396 progesterone C0015672 Fatigue

DB00396 progesterone C0020565 Breast Enlargement

DB00396 progesterone C0027497 Nausea

DB00396 progesterone C0003862 Joint Pain

DB00396 progesterone C0018681 Headache

DB00396 progesterone C0020040 Hot Flashes

DB00396 progesterone C0262397 Breast Tenderness

DB01242 clomipramine C0042571 vertigo

DB00317 gefitinib C0011991 Diarrhea

DB00317 gefitinib C0015230 Rash

DB00317 gefitinib C0702166 Acne

DB00990 exemestane C0016382 flushes

DB01190 clindamycin C0041834 Erythema

DB00215 citalopram C0002622 amnesia

DB00215 citalopram C0009676 confusion

DB00215 citalopram C0015230 rash

DB00215 citalopram C0016204 Flatulence

DB00215 citalopram C0020651 Postural hypotension

DB00215 citalopram C0021400 Influenza

DB00215 citalopram C0030193 pain

DB00215 citalopram C0030554 paraesthesia

DB00215 citalopram C0032617 Polyuria

DB00215 citalopram C0033774 Pruritus

DB00215 citalopram C0038663 suicide attempt

DB00215 citalopram C0039231 tachycardia

DB00215 citalopram C0043094 weight increase

DB00215 citalopram C0043096 weight decrease

DB00215 citalopram C0149931 Migraine

DB00215 citalopram C0851578 sleep disorder

DB00215 citalopram C0002453 amenorrhea

DB00215 citalopram C0010200 coughing

DB00215 citalopram C0020649 hypotension

DB00215 citalopram C0030554 paresthesia

DB00215 citalopram C0037036 saliva increased

DB00215 citalopram C0000729 abdominal cramp

DB00215 citalopram C0003862 arthralgia

DB00215 citalopram C0006277 bronchitis

DB00215 citalopram C0008031 chest pain

DB00215 citalopram C0013390 menstrual cramps

DB00215 citalopram C0015967 fever

DB00215 citalopram C0016382 flushes

DB00215 citalopram C0017160 gastroenteritis

DB00215 citalopram C0018834 heartburn

DB00215 citalopram C0020517 allergy

DB00215 citalopram C0020538 hypertension

DB00215 citalopram C0025345 menstrual disorder

DB00215 citalopram C0027424 nasal congestion

DB00215 citalopram C0030196 pain in limb

DB00215 citalopram C0030252 palpitation

DB00215 citalopram C0037195 sinus headache

DB00215 citalopram C0040264 tinnitus

DB00215 citalopram C0042029 urinary tract infection

DB00215 citalopram C0152029 sinus congestion

DB00215 citalopram C0231528 myalgia

DB00215 citalopram C0235198 concentration impaired

DB00215 citalopram C0344232 vision blurred

DB00215 citalopram C0677481 urinary frequency

DB00215 citalopram C0009450 infec

DB00215 citalopram C0012833 Dizziness

DB00215 citalopram C0033953 Sexual Dysfunction

DB00215 citalopram C1269683 Major Depressive Disorder

DB00215 citalopram C0027497 Nausea

DB00215 citalopram C0018681 Headache

DB00215 citalopram C0013456 ear ache

DB00215 citalopram C0017168 gastroesophageal reflux

DB00215 citalopram C0026821 muscle cramp

DB00215 citalopram C0037011 shoulder pain

DB00215 citalopram C0040460 tooth ache

DB00215 citalopram C0040822 tremor

DB00215 citalopram C0042571 vertigo

DB00215 citalopram C0042963 vomiting

DB01186 pergolide C0000737 abdominal pain

DB01186 pergolide C0002622 amnesia

DB01186 pergolide C0002871 anemia

DB01186 pergolide C0003467 anxiety

DB01186 pergolide C0003862 arthralgia

DB01186 pergolide C0004093 asthenia

DB01186 pergolide C0004604 back pain

DB01186 pergolide C0007859 neck pain

DB01186 pergolide C0008031 chest pain

DB01186 pergolide C0009676 confusion

DB01186 pergolide C0009806 constipation

DB01186 pergolide C0010200 cough

DB01186 pergolide C0011168 dysphagia

DB01186 pergolide C0011991 diarrhea

DB01186 pergolide C0012569 diplopia

DB01186 pergolide C0012833 dizziness

DB01186 pergolide C0013144 somnolence

DB01186 pergolide C0013384 dyskinesia

DB01186 pergolide C0013390 dysmenorrhea

DB01186 pergolide C0013395 dyspepsia

DB01186 pergolide C0013404 dyspnea

DB01186 pergolide C0015230 rash

DB01186 pergolide C0015967 fever

DB01186 pergolide C0018524 hallucinations

DB01186 pergolide C0018681 headache

DB01186 pergolide C0018802 congestive heart failure

DB01186 pergolide C0018965 hematuria

DB01186 pergolide C0020538 hypertension

DB01186 pergolide C0020651 postural hypotension

DB01186 pergolide C0021400 flu syndrome

DB01186 pergolide C0027497 nausea

DB01186 pergolide C0027769 nervousness

DB01186 pergolide C0030193 pain

DB01186 pergolide C0030252 palpitations

DB01186 pergolide C0030554 paresthesia

DB01186 pergolide C0031212 personality disorder

DB01186 pergolide C0031350 pharyngitis

DB01186 pergolide C0032285 pneumonia

DB01186 pergolide C0033975 psychosis

DB01186 pergolide C0035455 rhinitis

DB01186 pergolide C0038990 sweating

DB01186 pergolide C0039070 syncope

DB01186 pergolide C0040822 tremor

DB01186 pergolide C0042024 urinary incontinence

DB01186 pergolide C0042029 urinary tract infection

DB01186 pergolide C0042798 abnormal vision

DB01186 pergolide C0042963 vomiting

DB01186 pergolide C0043094 weight gain

DB01186 pergolide C0043096 weight loss

DB01186 pergolide C0043352 dry mouth

DB01186 pergolide C0085583 choreoathetosis

DB01186 pergolide C0085649 peripheral edema

DB01186 pergolide C0231528 myalgia

DB01186 pergolide C0575081 abnormal gait

DB01186 pergolide C0677481 urinary frequency

DB01186 pergolide C0917801 insomnia

DB00482 celecoxib C0018681 Headache

DB01229 paclitaxel C0002170 Alopecia

DB01229 paclitaxel C0002871 Anemia

DB01229 paclitaxel C0011991 Diarrhea

DB01229 paclitaxel C0021311 Infections

DB01229 paclitaxel C0023530 Leukopenia

DB01229 paclitaxel C0027497 Nausea

DB01229 paclitaxel C0027947 Neutropenia

DB01229 paclitaxel C0042963 vomiting

DB01229 paclitaxel C0019348 Herpes Simplex

DB01229 paclitaxel C0040034 Thrombocytopenia

DB00262 BCNU C0042029 Urinary Tract Infection

DB00363 clozapine C0009806 Constipation

DB00363 clozapine C0012833 Dizziness

DB00363 clozapine C0013144 Drowsiness

DB00363 clozapine C0013144 Somnolence

DB00363 clozapine C0037036 Salivary hypersecretion

DB00363 clozapine C0037036 Salivation

DB00363 clozapine C0039231 Tachycardia

DB00363 clozapine C0042571 vertigo

DB00363 clozapine C0043094 Weight increased

DB00363 clozapine C0917801 Insomnia

DB01142 doxepin C0013144 Drowsiness

DB00564 carbamazepine C0012833 DIZZINESS

DB00564 carbamazepine C0013144 SOMNOLENCE

DB00564 carbamazepine C0018681 Headache

DB00564 carbamazepine C0027497 NAUSEA

DB01083 orlistat C0000737 Abdominal Pain

DB01083 orlistat C0018681 Headache

DB01083 orlistat C0021400 Influenza

DB01083 orlistat C0041912 Upper Respiratory Infection

DB00904 ondansetron C0013144 Drowsiness

DB00904 ondansetron C0018681 Headache

DB00904 ondansetron C0011991 Diarrhea

DB00783 estradiol C0024902 Breast Pain

DB00783 estradiol C0003862 Arthralgia

DB00783 estradiol C0004604 Back Pain

DB00783 estradiol C0013395 Dyspepsia

DB00783 estradiol C0018681 Headache NOS

DB00783 estradiol C0021400 Influenza

DB00783 estradiol C0025874 Intermenstrual Bleeding

DB00783 estradiol C0027441 Nasopharyngitis

DB00783 estradiol C0030196 Pain in Limb

DB00783 estradiol C0037195 Sinus Headache

DB00783 estradiol C0037199 Sinusitis NOS

DB00783 estradiol C0262397 Breast Tenderness

DB00488 HTM C0002871 Anemia

DB00488 HTM C0027497 Nausea

DB00488 HTM C0042963 Vomiting

DB00488 HTM C0442874 Neuropathy

DB01156 bupropion C0018681 Headache

DB01156 bupropion C0043352 Dry mouth

DB01222 budesonide C0021311 Infection

DB00490 buspirone C0008031 chest pain

DB00490 buspirone C0009676 confusion

DB00490 buspirone C0009806 constipation

DB00490 buspirone C0012833 Dizziness

DB00490 buspirone C0013144 drowsiness

DB00490 buspirone C0015230 Skin rash

DB00490 buspirone C0015672 fatigue

DB00490 buspirone C0018681 headache

DB00490 buspirone C0027424 Nasal congestion

DB00490 buspirone C0027497 Nausea

DB00490 buspirone C0027769 nervousness

DB00490 buspirone C0028084 nightmares

DB00490 buspirone C0028643 numbness

DB00490 buspirone C0030552 weakness

DB00490 buspirone C0030554 Paresthesia

DB00490 buspirone C0038990 sweating

DB00490 buspirone C0039231 Tachycardia

DB00490 buspirone C0040264 Tinnitus

DB00490 buspirone C0040822 tremor

DB00490 buspirone C0043352 Dry mouth

DB00490 buspirone C0220870 lightheadedness

DB00490 buspirone C0231528 muscle aches

DB00490 buspirone C0242429 sore throat

DB00490 buspirone C0344232 blurred vision

DB00490 buspirone C0917801 insomnia

DB00490 buspirone C0011991 diarrhea

DB00490 buspirone C0042963 vomiting

DB00665 nilutamide C0016382 flushes

DB00394 beclomethasone C0018681 HEADACHE

DB00394 beclomethasone C0031350 PHARYNGITIS

DB00615 rifabutin C0027947 Neutropenia

DB01085 pilocarpine C0038990 Sweating

DB00181 baclofen C0013144 Somnolence

DB00993 azathioprine C0023530 Leukopenia

DB00714 apomorphine C0013144 Drowsiness

DB00714 apomorphine C0013144 Somnolence

DB00714 apomorphine C0013384 Dyskinesias

DB00714 apomorphine C0027497 Nausea

DB00714 apomorphine C0042963 Vomiting

DB01204 mitoxantrone C0002170 Alopecia

DB01204 mitoxantrone C0002453 Amenorrhea

DB01204 mitoxantrone C0003123 Anorexia

DB01204 mitoxantrone C0004093 Asthenia

DB01204 mitoxantrone C0011991 Diarrhea

DB01204 mitoxantrone C0013604 Edema

DB01204 mitoxantrone C0015672 Fatigue

DB01204 mitoxantrone C0015967 Fever

DB01204 mitoxantrone C0019080 Bleeding

DB01204 mitoxantrone C0020456 Hyperglycemia

DB01204 mitoxantrone C0021311 Infections

DB01204 mitoxantrone C0025345 Menstrual disorder

DB01204 mitoxantrone C0027497 Nausea

DB01204 mitoxantrone C0030193 Pain

DB01204 mitoxantrone C0038362 stomatitis

DB01204 mitoxantrone C0041912 Upper respiratory tract infection

DB01204 mitoxantrone C0042029 Urinary tract infection

DB01204 mitoxantrone C0042963 vomiting

DB01204 mitoxantrone C0231218 Malaise

DB01204 mitoxantrone C0243026 Sepsis

DB01118 amiodarone C0020649 Hypotension

DB00201 theophylline C0000737 Abdominal Pain

DB00201 theophylline C0003811 arrhythmias

DB00201 theophylline C0020456 Hyperglycemia

DB00201 theophylline C0020621 Hypokalemia

DB00201 theophylline C0020649 Hypotension

DB00201 theophylline C0027769 Nervousness

DB00201 theophylline C0036974 shock

DB00201 theophylline C0039239 Sinus tachycardia

DB00201 theophylline C0039240 supraventricular tachycardias

DB00201 theophylline C0040822 Tremors

DB00201 theophylline C0042963 Vomiting

DB00201 theophylline C0151636 Ventricular premature beats

DB00201 theophylline C0039231 tachycardias

DB00201 theophylline C0039231 tachycardia

DB00264 metoprolol C0018801 Heart failure

DB00264 metoprolol C0020649 Hypotension

DB01001 salbutamol C0018681 Headache

DB01001 salbutamol C0040822 Tremor
