# Supplementary material for: Building the process-drug–side effect network to discover the relationship between biological Processes and side effects
Source: BMC Bioinformatics. 2011 Mar 29;12(Suppl 2):S2. doi: 10.1186/1471-2105-12-S2-S2 (PMC3073182; doi:10.1186/1471-2105-12-S2-S2)
Supplement: Additional file 2 — This file contains up_regulated processes (T-score > 3.0) and related effects. First Column: Effect ID ( UMLS Concept ID) Second Column: Process ID ( Gene Ontology ID) Third Column: The number of drugs which affect to process and causing the side effect. Fourth Column: Total drugs which are causing the side effect. [file 1471-2105-12-S2-S2-S2.doc]

C0011991 GO:0005786 1 19

C0011991 GO:0007598 1 19

C0011991 GO:0043954 1 19

C0011991 GO:0043525 1 19

C0011991 GO:0048066 2 19

C0011991 GO:0002706 1 19

C0011991 GO:0002703 1 19

C0011991 GO:0034199 1 19

C0011991 GO:0002700 2 19

C0011991 GO:0016864 1 19

C0011991 GO:0016863 1 19

C0011991 GO:0016862 1 19

C0011991 GO:0009168 1 19

C0011991 GO:0016860 3 19

C0011991 GO:0014031 1 19

C0011991 GO:0000002 1 19

C0011991 GO:0043206 2 19

C0011991 GO:0005834 3 19

C0011991 GO:0006978 1 19

C0011991 GO:0004653 2 19

C0011991 GO:0006309 4 19

C0011991 GO:0030914 1 19

C0011991 GO:0004716 1 19

C0011991 GO:0007080 1 19

C0011991 GO:0030856 1 19

C0011991 GO:0016514 2 19

C0011991 GO:0051294 2 19

C0011991 GO:0002824 1 19

C0011991 GO:0003923 2 19

C0011991 GO:0002822 3 19

C0011991 GO:0031109 2 19

C0011991 GO:0021510 1 19

C0011991 GO:0035270 1 19

C0011991 GO:0002673 1 19

C0011991 GO:0000718 1 19

C0011991 GO:0006278 1 19

C0011991 GO:0016812 2 19

C0011991 GO:0006271 1 19

C0011991 GO:0045259 2 19

C0011991 GO:0016814 4 19

C0011991 GO:0010887 2 19

C0011991 GO:0010885 1 19

C0011991 GO:0010884 1 19

C0011991 GO:0006376 1 19

C0011991 GO:0004622 1 19

C0011991 GO:0006378 1 19

C0011991 GO:0019320 1 19

C0011991 GO:0031958 1 19

C0011991 GO:0032412 1 19

C0011991 GO:0048568 1 19

C0011991 GO:0006458 1 19

C0011991 GO:0000302 3 19

C0011991 GO:0030175 2 19

C0011991 GO:0008483 2 19

C0011991 GO:0030170 1 19

C0011991 GO:0008484 3 19

C0011991 GO:0005487 1 19

C0011991 GO:0010862 1 19

C0011991 GO:0051224 1 19

C0011991 GO:0051225 1 19

C0011991 GO:0003205 2 19

C0011991 GO:0003206 2 19

C0011991 GO:0015992 3 19

C0011991 GO:0015491 1 19

C0011991 GO:0042551 2 19

C0011991 GO:0006885 2 19

C0011991 GO:0042255 1 19

C0011991 GO:0003208 2 19

C0011991 GO:0042558 1 19

C0011991 GO:0042559 1 19

C0011991 GO:0002687 1 19

C0011991 GO:0002685 1 19

C0011991 GO:0051653 1 19

C0011991 GO:0043021 2 19

C0011991 GO:0043022 2 19

C0011991 GO:0042827 1 19

C0011991 GO:0051972 1 19

C0011991 GO:0007622 1 19

C0011991 GO:0009304 3 19

C0011991 GO:0032479 1 19

C0011991 GO:0048709 2 19

C0011991 GO:0000956 1 19

C0011991 GO:0033764 1 19

C0011991 GO:0009260 2 19

C0011991 GO:0007052 1 19

C0011991 GO:0009262 1 19

C0011991 GO:0009264 1 19

C0011991 GO:0009267 2 19

C0011991 GO:0009266 1 19

C0011991 GO:0006303 1 19

C0011991 GO:0035303 2 19

C0011991 GO:0044042 1 19

C0011991 GO:0030675 1 19

C0011991 GO:0031080 1 19

C0011991 GO:0004536 1 19

C0011991 GO:0015781 1 19

C0011991 GO:0015780 1 19

C0011991 GO:0004532 1 19

C0011991 GO:0016776 1 19

C0011991 GO:0006691 1 19

C0011991 GO:0015074 2 19

C0011991 GO:0048365 1 19

C0011991 GO:0016676 3 19

C0011991 GO:0015179 1 19

C0011991 GO:0010553 1 19

C0011991 GO:0016675 3 19

C0011991 GO:0015175 1 19

C0011991 GO:0006026 1 19

C0011991 GO:0006027 1 19

C0011991 GO:0030880 1 19

C0011991 GO:0033613 1 19

C0011991 GO:0008143 1 19

C0011991 GO:0033209 2 19

C0011991 GO:0008144 2 19

C0011991 GO:0042633 1 19

C0011991 GO:0031228 1 19

C0011991 GO:0042177 3 19

C0011991 GO:0010001 1 19

C0011991 GO:0051453 1 19

C0011991 GO:0031498 1 19

C0011991 GO:0042645 1 19

C0011991 GO:0051457 1 19

C0011991 GO:0009395 1 19

C0011991 GO:0009394 1 19

C0011991 GO:0000127 3 19

C0011991 GO:0007127 1 19

C0011991 GO:0042267 1 19

C0011991 GO:0002228 1 19

C0011991 GO:0045621 1 19

C0011991 GO:0005506 1 19

C0011991 GO:0045622 1 19

C0011991 GO:0033108 2 19

C0011991 GO:0055002 1 19

C0011991 GO:0042552 2 19

C0011991 GO:0006879 1 19

C0011991 GO:0006073 1 19

C0011991 GO:0042288 2 19

C0011991 GO:0050879 1 19

C0011991 GO:0055008 1 19

C0011991 GO:0015929 1 19

C0011991 GO:0005913 1 19

C0011991 GO:0001656 1 19

C0011991 GO:0046889 3 19

C0011991 GO:0033293 1 19

C0011991 GO:0044253 1 19

C0011991 GO:0045884 1 19

C0011991 GO:0006337 1 19

C0011991 GO:0006099 1 19

C0011991 GO:0016528 1 19

C0011991 GO:0016529 1 19

C0011991 GO:0004190 1 19

C0011991 GO:0003899 1 19

C0011991 GO:0042797 3 19

C0011991 GO:0042791 3 19

C0011991 GO:0017046 1 19

C0011991 GO:0045923 1 19

C0011991 GO:0019047 1 19

C0011991 GO:0017048 1 19

C0011991 GO:0060021 1 19

C0011991 GO:0008213 1 19

C0011991 GO:0042593 1 19

C0011991 GO:0022404 1 19

C0011991 GO:0008210 1 19

C0011991 GO:0042594 3 19

C0011991 GO:0015184 1 19

C0011991 GO:0007270 1 19

C0011991 GO:0043631 1 19

C0011991 GO:0008603 2 19

C0011991 GO:0006096 2 19

C0011991 GO:0046209 1 19

C0011991 GO:0009820 2 19

C0011991 GO:0033993 1 19

C0011991 GO:0050996 2 19

C0011991 GO:0017080 1 19

C0011991 GO:0032210 1 19

C0011991 GO:0003730 2 19

C0011991 GO:0045814 1 19

C0011991 GO:0019210 1 19

C0011991 GO:0019213 1 19

C0011991 GO:0019212 1 19

C0011991 GO:0045089 1 19

C0011991 GO:0006706 1 19

C0011991 GO:0070822 1 19

C0011991 GO:0048592 1 19

C0011991 GO:0002444 1 19

C0011991 GO:0000272 3 19

C0011991 GO:0051293 1 19

C0011991 GO:0060485 1 19

C0011991 GO:0000445 3 19

C0011991 GO:0040017 2 19

C0011991 GO:0030532 1 19

C0011991 GO:0009913 1 19

C0011991 GO:0035195 1 19

C0011991 GO:0001836 1 19

C0011991 GO:0001837 2 19

C0011991 GO:0005763 2 19

C0011991 GO:0048524 1 19

C0011991 GO:0006413 1 19

C0011991 GO:0048520 1 19

C0011991 GO:0019363 1 19

C0011991 GO:0019362 2 19

C0011991 GO:0006633 1 19

C0011991 GO:0042133 2 19

C0011991 GO:0034446 1 19

C0011991 GO:0000086 1 19

C0011991 GO:0007612 1 19

C0011991 GO:0000080 2 19

C0011991 GO:0009451 1 19

C0011991 GO:0043449 1 19

C0011991 GO:0007163 1 19

C0011991 GO:0003015 1 19

C0011991 GO:0019915 1 19

C0011991 GO:0042765 2 19

C0011991 GO:0016597 3 19

C0011991 GO:0009295 1 19

C0011991 GO:0007004 1 19

C0011991 GO:0042446 1 19

C0011991 GO:0005540 1 19

C0011991 GO:0002263 1 19

C0011991 GO:0004402 1 19

C0011991 GO:0010257 2 19

C0011991 GO:0001909 1 19

C0011991 GO:0032135 2 19

C0011991 GO:0006664 1 19

C0011991 GO:0015002 3 19

C0011991 GO:0033032 2 19

C0011991 GO:0034366 1 19

C0011991 GO:0051119 1 19

C0011991 GO:0043968 1 19

C0011991 GO:0005310 1 19

C0011991 GO:0016209 4 19

C0011991 GO:0004385 1 19

C0011991 GO:0001516 1 19

C0011991 GO:0008170 2 19

C0011991 GO:0000796 2 19

C0011991 GO:0000794 1 19

C0011991 GO:0016895 1 19

C0011991 GO:0009074 2 19

C0011991 GO:0070279 1 19

C0011991 GO:0009070 1 19

C0011991 GO:0030261 2 19

C0011991 GO:0031668 1 19

C0011991 GO:0031669 1 19

C0011991 GO:0000159 1 19

C0011991 GO:0016566 2 19

C0011991 GO:0004707 1 19

C0011991 GO:0007043 1 19

C0011991 GO:0005388 1 19

C0011991 GO:0016444 2 19

C0011991 GO:0004033 1 19

C0011991 GO:0018024 1 19

C0011991 GO:0006297 2 19

C0011991 GO:0017166 2 19

C0011991 GO:0005732 1 19

C0011991 GO:0015851 1 19

C0011991 GO:0006298 3 19

C0011991 GO:0008250 1 19

C0011991 GO:0006805 1 19

C0011991 GO:0048009 2 19

C0011991 GO:0001764 2 19

C0011991 GO:0046496 2 19

C0011991 GO:0005929 1 19

C0011991 GO:0031345 1 19

C0011991 GO:0030280 1 19

C0011991 GO:0051187 1 19

C0011991 GO:0051181 2 19

C0011991 GO:0051180 2 19

C0011991 GO:0016801 1 19

C0011991 GO:0042098 1 19

C0011991 GO:0016896 1 19

C0011991 GO:0000779 1 19

C0011991 GO:0007183 1 19

C0011991 GO:0004551 1 19

C0011991 GO:0030149 1 19

C0011991 GO:0031941 1 19

C0011991 GO:0006221 1 19

C0011991 GO:0000339 1 19

C0011991 GO:0032768 1 19

C0011991 GO:0032769 2 19

C0011991 GO:0017015 1 19

C0011991 GO:0019319 3 19

C0011991 GO:0050795 1 19

C0011991 GO:0003995 2 19

C0011991 GO:0003231 2 19

C0011991 GO:0030069 1 19

C0011991 GO:0006911 1 19

C0011991 GO:0046660 1 19

C0011991 GO:0008652 1 19

C0011991 GO:0043666 1 19

C0011991 GO:0042542 4 19

C0011991 GO:0022898 2 19

C0011991 GO:0010714 1 19

C0011991 GO:0010712 1 19

C0011991 GO:0070198 1 19

C0011991 GO:0046824 1 19

C0011991 GO:0003746 2 19

C0011991 GO:0001942 1 19

C0011991 GO:0019897 1 19

C0011991 GO:0016796 2 19

C0011991 GO:0006637 1 19

C0011991 GO:0006636 1 19

C0011991 GO:0006635 1 19

C0011991 GO:0006733 1 19

C0011991 GO:0009311 1 19

C0011991 GO:0004520 1 19

C0011991 GO:0032967 1 19

C0011991 GO:0004859 1 19

C0011991 GO:0007041 1 19

C0011991 GO:0032963 1 19

C0011991 GO:0000428 1 19

C0011991 GO:0004529 1 19

C0011991 GO:0016709 1 19

C0011991 GO:0016706 1 19

C0011991 GO:0004527 2 19

C0011991 GO:0004526 1 19

C0011991 GO:0031099 1 19

C0011991 GO:0001945 1 19

C0011991 GO:0050768 2 19

C0011991 GO:0050769 3 19

C0011991 GO:0032409 1 19

C0011991 GO:0015165 2 19

C0011991 GO:0031396 1 19

C0011991 GO:0031397 3 19

C0011991 GO:0006744 1 19

C0011991 GO:0006743 1 19

C0011991 GO:0006740 2 19

C0011991 GO:0005355 1 19

C0011991 GO:0005217 2 19

C0011991 GO:0046466 1 19

C0011991 GO:0014065 1 19

C0011991 GO:0007131 1 19

C0011991 GO:0055067 2 19

C0011991 GO:0043473 2 19

C0011991 GO:0031625 2 19

C0011991 GO:0002460 1 19

C0011991 GO:0030174 1 19

C0011991 GO:0000299 1 19

C0011991 GO:0048167 1 19

C0011991 GO:0002366 1 19

C0011991 GO:0016018 2 19

C0011991 GO:0032404 2 19

C0011991 GO:0005104 1 19

C0011991 GO:0004177 2 19

C0011991 GO:0050868 1 19

C0011991 GO:0055010 2 19

C0011991 GO:0046365 2 19

C0011991 GO:0046364 3 19

C0011991 GO:0016234 1 19

C0011991 GO:0030983 2 19

C0011991 GO:0009108 1 19

C0011991 GO:0009109 2 19

C0011991 GO:0034623 1 19

C0011991 GO:0034235 2 19

C0011991 GO:0000175 1 19

C0011991 GO:0016846 1 19

C0011991 GO:0031532 1 19

C0011991 GO:0004180 2 19

C0011991 GO:0000060 1 19

C0011991 GO:0005814 1 19

C0011991 GO:0018279 1 19

C0011991 GO:0016607 1 19

C0011991 GO:0070301 2 19

C0011991 GO:0045939 2 19

C0011991 GO:0031124 1 19

C0011991 GO:0010812 1 19

C0011991 GO:0050690 2 19

C0011991 GO:0022417 2 19

C0011991 GO:0016903 3 19

C0011991 GO:0022410 1 19

C0011991 GO:0043627 1 19

C0011991 GO:0007266 3 19

C0011991 GO:0055088 1 19

C0011991 GO:0001637 1 19

C0011991 GO:0005851 2 19

C0011991 GO:0003706 1 19

C0011991 GO:0032202 1 19

C0011991 GO:0000387 1 19

C0011991 GO:0006390 2 19

C0011991 GO:0006479 1 19

C0011991 GO:0005782 2 19

C0011991 GO:0034440 1 19

C0011991 GO:0032387 1 19

C0011991 GO:0048469 1 19

C0011991 GO:0005788 1 19

C0011991 GO:0004950 1 19

C0011991 GO:0015645 1 19

C0011991 GO:0031058 1 19

C0011991 GO:0030119 2 19

C0011991 GO:0030118 2 19

C0011991 GO:0015718 1 19

C0011991 GO:0005391 1 19

C0011991 GO:0007219 1 19

C0011991 GO:0016628 2 19

C0011991 GO:0016627 2 19

C0011991 GO:0007216 2 19

C0011991 GO:0016620 2 19

C0011991 GO:0070001 1 19

C0011991 GO:0048512 1 19

C0011991 GO:0010720 1 19

C0011991 GO:0010721 2 19

C0011991 GO:0031907 2 19

C0011991 GO:0019359 1 19

C0011991 GO:0005678 1 19

C0011991 GO:0046635 1 19

C0011991 GO:0048762 1 19

C0011991 GO:0000099 1 19

C0011991 GO:0016799 1 19

C0011991 GO:0005092 1 19

C0011991 GO:0005095 2 19

C0011991 GO:0003007 1 19

C0011991 GO:0043536 1 19

C0011991 GO:0043535 1 19

C0011991 GO:0045187 1 19

C0011991 GO:0007076 1 19

C0011991 GO:0055117 1 19

C0011991 GO:0003009 1 19

C0011991 GO:0051096 2 19

C0011991 GO:0051095 1 19

C0011991 GO:0070566 1 19

C0011991 GO:0005527 2 19

C0011991 GO:0043523 2 19

C0011991 GO:0004550 2 19

C0011991 GO:0043027 1 19

C0011991 GO:0033558 1 19

C0011991 GO:0006672 2 19

C0011991 GO:0045494 1 19

C0011991 GO:0050921 2 19

C0011991 GO:0006921 2 19

C0011991 GO:0009142 1 19

C0011991 GO:0009145 1 19

C0011991 GO:0016278 1 19

C0011991 GO:0016279 1 19

C0011991 GO:0009062 1 19

C0011991 GO:0000783 1 19

C0011991 GO:0000782 1 19

C0011991 GO:0016885 2 19

C0011991 GO:0009066 1 19

C0011991 GO:0009065 1 19

C0011991 GO:0048168 1 19

C0011991 GO:0031576 1 19

C0011991 GO:0042625 1 19

C0011991 GO:0031572 1 19

C0011991 GO:0045116 1 19

C0011991 GO:0090100 1 19

C0011991 GO:0031571 1 19

C0011991 GO:0005852 3 19

C0011991 GO:0031672 1 19

C0011991 GO:0007006 1 19

C0011991 GO:0016575 1 19

C0011991 GO:0016574 1 19

C0011991 GO:0005528 2 19

C0011991 GO:0005522 1 19

C0011991 GO:0016471 2 19

C0011991 GO:0004129 3 19

C0011991 GO:0004128 2 19

C0011991 GO:0006818 2 19

C0011991 GO:0055029 1 19

C0011991 GO:0001776 1 19

C0011991 GO:0045334 1 19

C0011991 GO:0006189 1 19

C0011991 GO:0050811 1 19

C0011991 GO:0030275 1 19

C0011991 GO:0042303 1 19

C0011991 GO:0008308 1 19

C0011991 GO:0044275 4 19

C0011991 GO:0000738 2 19

C0011991 GO:0000737 1 19

C0011991 GO:0000731 1 19

C0011991 GO:0006739 2 19

C0011991 GO:0030276 1 19

C0011991 GO:0004601 4 19

C0011991 GO:0004602 3 19

C0011991 GO:0006188 1 19

C0011991 GO:0032770 1 19

C0011991 GO:0010921 2 19

C0011991 GO:0045055 1 19

C0011991 GO:0042772 1 19

C0011991 GO:0017022 1 19

C0011991 GO:0010927 1 19

C0011991 GO:0045580 1 19

C0011991 GO:0045582 2 19

C0011991 GO:0060047 1 19

C0011991 GO:0046677 1 19

C0011991 GO:0003229 2 19

C0011991 GO:0019395 1 19

C0011991 GO:0001953 1 19

C0011991 GO:0060048 1 19

C0011991 GO:0045426 1 19

C0011991 GO:0030018 1 19

C0011991 GO:0050881 1 19

C0011991 GO:0015278 1 19

C0011991 GO:0001707 2 19

C0011991 GO:0001704 2 19

C0011991 GO:0051087 2 19

C0011991 GO:0042375 1 19

C0011991 GO:0015370 1 19

C0011991 GO:0045833 2 19

C0011991 GO:0003756 1 19

C0011991 GO:0070688 1 19

C0011991 GO:0017156 2 19

C0011991 GO:0030669 1 19

C0011991 GO:0043094 1 19

C0011991 GO:0019209 1 19

C0011991 GO:0048332 2 19

C0011991 GO:0031330 1 19

C0011991 GO:0031333 1 19

C0011991 GO:0000217 1 19

C0011991 GO:0004860 2 19

C0011991 GO:0030128 2 19

C0011991 GO:0004864 1 19

C0011991 GO:0030122 2 19

C0011991 GO:0008091 1 19

C0011991 GO:0030121 1 19

C0011991 GO:0030656 1 19

C0011991 GO:0030125 2 19

C0011991 GO:0050770 1 19

C0011991 GO:0001959 4 19

C0011991 GO:0050772 1 19

C0011991 GO:0032182 1 19

C0011991 GO:0001952 1 19

C0011991 GO:0008535 1 19

C0011991 GO:0005742 1 19

C0011991 GO:0070461 1 19

C0011991 GO:0005744 1 19

C0011991 GO:0016653 2 19

C0011991 GO:0046040 1 19

C0011991 GO:0006007 3 19

C0011991 GO:0002039 2 19

C0011991 GO:0034502 1 19

C0011991 GO:0042116 2 19

C0011991 GO:0046890 1 19

C0011991 GO:0034508 1 19

C0011991 GO:0042113 1 19

C0011991 GO:0016831 1 19

C0011991 GO:0005890 1 19

C0011991 GO:0043507 1 19

C0011991 GO:0032986 1 19

C0011991 GO:0007020 1 19

C0011991 GO:0032984 1 19

C0011991 GO:0043467 1 19

C0011991 GO:0032981 2 19

C0011991 GO:0031638 1 19

C0011991 GO:0045766 1 19

C0011991 GO:0004467 1 19

C0011991 GO:0004468 1 19

C0011991 GO:0030165 1 19

C0011991 GO:0016229 1 19

C0011991 GO:0046426 1 19

C0011991 GO:0005007 1 19

C0011991 GO:0046356 1 19

C0011991 GO:0030239 1 19

C0011991 GO:0006769 2 19

C0011991 GO:0007584 1 19

C0011991 GO:0007585 1 19

C0011991 GO:0034341 2 19

C0011991 GO:0008198 1 19

C0011991 GO:0034220 1 19

C0011991 GO:0040001 1 19

C0011991 GO:0042026 1 19

C0011991 GO:0009116 1 19

C0011991 GO:0051646 1 19

C0011991 GO:0009119 1 19

C0011991 GO:0070925 1 19

C0011991 GO:0006941 1 19

C0011991 GO:0016877 1 19

C0011991 GO:0018342 1 19

C0011991 GO:0000070 1 19

C0011991 GO:0006754 1 19

C0011991 GO:0016878 1 19

C0011991 GO:0019842 2 19

C0011991 GO:0031526 1 19

C0011991 GO:0046457 1 19

C0011991 GO:0046456 1 19

C0011991 GO:0043596 1 19

C0011991 GO:0033176 2 19

C0011991 GO:0046519 2 19

C0011991 GO:0016504 1 19

C0011991 GO:0016505 1 19

C0011991 GO:0046638 2 19

C0011991 GO:0046637 1 19

C0011991 GO:0051287 2 19

C0011991 GO:0060558 1 19

C0011991 GO:0046634 1 19

C0011991 GO:0046631 2 19

C0011991 GO:0016646 2 19

C0011991 GO:0055072 1 19

C0011991 GO:0050920 1 19

C0011991 GO:0051262 1 19

C0011991 GO:0045309 2 19

C0011991 GO:0051893 1 19

C0011991 GO:0034765 1 19

C0011991 GO:0008376 1 19

C0011991 GO:0008278 1 19

C0011991 GO:0034762 1 19

C0011991 GO:0008373 1 19

C0011991 GO:0015804 1 19

C0011991 GO:0006983 1 19

C0011991 GO:0005640 1 19

C0011991 GO:0018196 1 19

C0011991 GO:0015238 2 19

C0011991 GO:0006266 1 19

C0011991 GO:0043325 1 19

C0011991 GO:0010894 2 19

C0011991 GO:0010896 3 19

C0011991 GO:0010898 1 19

C0011991 GO:0006383 1 19

C0011991 GO:0032393 2 19

C0011991 GO:0009206 1 19

C0011991 GO:0014003 2 19

C0011991 GO:0009201 1 19

C0011991 GO:0000314 2 19

C0011991 GO:0022405 1 19

C0011991 GO:0006563 1 19

C0011991 GO:0010975 2 19

C0011991 GO:0030041 1 19

C0011991 GO:0030515 1 19

C0011991 GO:0030511 1 19

C0011991 GO:0051354 2 19

C0011991 GO:0007205 1 19

C0011991 GO:0009410 1 19

C0011991 GO:0005663 1 19

C0011991 GO:0043130 1 19

C0011991 GO:0005665 1 19

C0011991 GO:0031123 1 19

C0011991 GO:0019439 1 19

C0011991 GO:0060415 1 19

C0011991 GO:0021782 1 19

C0011991 GO:0005080 1 19

C0011991 GO:0002821 1 19

C0011991 GO:0005086 1 19

C0011991 GO:0042745 1 19

C0011991 GO:0042744 1 19

C0011991 GO:0042743 1 19

C0011991 GO:0005871 2 19

C0011991 GO:0031369 3 19

C0011991 GO:0055102 1 19

C0011991 GO:0042749 1 19

C0011991 GO:0008408 1 19

C0011991 GO:0016049 1 19

C0011991 GO:0008235 1 19

C0011991 GO:0008406 1 19

C0011991 GO:0015149 1 19

C0011991 GO:0010810 1 19

C0011991 GO:0019239 4 19

C0011991 GO:0005662 2 19

C0011991 GO:0006687 1 19

C0011991 GO:0016684 4 19

C0011991 GO:0015145 1 19

C0011991 GO:0016769 2 19

C0011991 GO:0000125 1 19

C0011991 GO:0030894 1 19

C0011991 GO:0042384 1 19

C0011991 GO:0046545 1 19

C0011991 GO:0009156 1 19

C0011991 GO:0048500 1 19

C0011991 GO:0009152 1 19

C0011991 GO:0008154 1 19

C0011991 GO:0048256 2 19

C0011991 GO:0043256 1 19

C0011991 GO:0008159 1 19

C0011991 GO:0000030 1 19

C0011991 GO:0045165 1 19

C0011991 GO:0000132 2 19

C0011991 GO:0007250 1 19

C0011991 GO:0031647 1 19

C0011991 GO:0030867 3 19

C0011991 GO:0031579 1 19

C0011991 GO:0010883 1 19

C0011991 GO:0045736 2 19

C0011991 GO:0016469 2 19

C0011991 GO:0030431 1 19

C0011991 GO:0002819 2 19

C0011991 GO:0051004 2 19

C0011991 GO:0005901 1 19

C0011991 GO:0030262 2 19

C0011991 GO:0051000 1 19

C0011991 GO:0048029 1 19

C0011991 GO:0016909 1 19

C0011991 GO:0009650 1 19

C0011991 GO:0007259 1 19

C0011991 GO:0009127 1 19

C0011991 GO:0044246 1 19

C0011991 GO:0006220 1 19

C0011991 GO:0010165 1 19

C0011991 GO:0070603 2 19

C0011991 GO:0001541 1 19

C0011991 GO:0000725 1 19

C0011991 GO:0000724 1 19

C0011991 GO:0006342 1 19

C0011991 GO:0042921 1 19

C0011991 GO:0046784 3 19

C0011991 GO:0046782 1 19

C0011991 GO:0044452 1 19

C0011991 GO:0008385 1 19

C0011991 GO:0070761 1 19

C0011991 GO:0010939 2 19

C0011991 GO:0003709 2 19

C0011991 GO:0019079 2 19

C0011991 GO:0046165 2 19

C0011991 GO:0015298 1 19

C0011991 GO:0035035 1 19

C0011991 GO:0034062 1 19

C0011991 GO:0051318 1 19

C0011991 GO:0030004 1 19

C0011991 GO:0010832 1 19

C0011991 GO:0010833 2 19

C0011991 GO:0010830 1 19

C0011991 GO:0051310 1 19

C0011991 GO:0055085 1 19

C0011991 GO:0006084 1 19

C0011991 GO:0008634 1 19

C0011991 GO:0008637 1 19

C0011991 GO:0005507 1 19

C0011991 GO:0043601 1 19

C0011991 GO:0006081 3 19

C0011991 GO:0043603 2 19

C0011991 GO:0070776 1 19

C0011991 GO:0070775 1 19

C0011991 GO:0042813 1 19

C0011991 GO:0043370 1 19

C0011991 GO:0045861 1 19

C0011991 GO:0034976 1 19

C0011991 GO:0000184 1 19

C0011991 GO:0019206 1 19

C0011991 GO:0055001 1 19

C0011991 GO:0008630 1 19

C0011991 GO:0019751 1 19

C0011991 GO:0008287 1 19

C0011991 GO:0000347 3 19

C0011991 GO:0000346 3 19

C0011991 GO:0030131 1 19

C0011991 GO:0030132 2 19

C0011991 GO:0030641 1 19

C0011991 GO:0050660 1 19

C0011991 GO:0034381 1 19

C0011991 GO:0070555 2 19

C0011991 GO:0033500 1 19

C0011991 GO:0016645 3 19

C0011991 GO:0008526 2 19

C0011991 GO:0005753 2 19

C0011991 GO:0010466 1 19

C0011991 GO:0070410 1 19

C0011991 GO:0006400 1 19

C0011991 GO:0019104 2 19

C0011991 GO:0005881 2 19

C0011991 GO:0005883 1 19

C0011991 GO:0007159 2 19

C0011991 GO:0051015 1 19

C0011991 GO:0002440 1 19

C0011991 GO:0000381 2 19

C0011991 GO:0007157 2 19

C0011991 GO:0002443 1 19

C0011991 GO:0051896 1 19

C0011991 GO:0002446 1 19

C0011991 GO:0042771 1 19

C0011991 GO:0042063 1 19

C0011991 GO:0002562 2 19

C0011991 GO:0016580 1 19

C0011991 GO:0016581 2 19

C0011991 GO:0001938 1 19

C0011991 GO:0033344 2 19

C0011991 GO:0004576 1 19

C0011991 GO:0002250 1 19

C0011991 GO:0004549 1 19

C0011991 GO:0001654 1 19

C0011991 GO:0001933 1 19

C0011991 GO:0004579 1 19

C0011991 GO:0001937 1 19

C0011991 GO:0001936 1 19

C0027497 GO:0006776 1 26

C0027497 GO:0010149 1 26

C0027497 GO:0007598 2 26

C0027497 GO:0043954 1 26

C0027497 GO:0006906 1 26

C0027497 GO:0048066 2 26

C0027497 GO:0002706 2 26

C0027497 GO:0002703 2 26

C0027497 GO:0034199 1 26

C0027497 GO:0016868 1 26

C0027497 GO:0016866 1 26

C0027497 GO:0016864 1 26

C0027497 GO:0016863 1 26

C0027497 GO:0016862 1 26

C0027497 GO:0009168 2 26

C0027497 GO:0016860 3 26

C0027497 GO:0006306 1 26

C0027497 GO:0006305 1 26

C0027497 GO:0000002 2 26

C0027497 GO:0043206 2 26

C0027497 GO:0005834 3 26

C0027497 GO:0003158 1 26

C0027497 GO:0004653 3 26

C0027497 GO:0019239 5 26

C0027497 GO:0006309 4 26

C0027497 GO:0030914 1 26

C0027497 GO:0004716 2 26

C0027497 GO:0009218 1 26

C0027497 GO:0002366 1 26

C0027497 GO:0030856 1 26

C0027497 GO:0016514 2 26

C0027497 GO:0016101 1 26

C0027497 GO:0030858 1 26

C0027497 GO:0051294 2 26

C0027497 GO:0002824 2 26

C0027497 GO:0003923 2 26

C0027497 GO:0002822 4 26

C0027497 GO:0031109 2 26

C0027497 GO:0021510 2 26

C0027497 GO:0007281 1 26

C0027497 GO:0035270 1 26

C0027497 GO:0007183 2 26

C0027497 GO:0002673 1 26

C0027497 GO:0000718 1 26

C0027497 GO:0006278 1 26

C0027497 GO:0031050 2 26

C0027497 GO:0016812 3 26

C0027497 GO:0006271 2 26

C0027497 GO:0006270 1 26

C0027497 GO:0045259 2 26

C0027497 GO:0016814 4 26

C0027497 GO:0010887 2 26

C0027497 GO:0015884 1 26

C0027497 GO:0010885 1 26

C0027497 GO:0010884 1 26

C0027497 GO:0006376 1 26

C0027497 GO:0070776 1 26

C0027497 GO:0004622 1 26

C0027497 GO:0005161 1 26

C0027497 GO:0006378 1 26

C0027497 GO:0010888 1 26

C0027497 GO:0021537 1 26

C0027497 GO:0019320 1 26

C0027497 GO:0009895 1 26

C0027497 GO:0031958 1 26

C0027497 GO:0032412 1 26

C0027497 GO:0030897 1 26

C0027497 GO:0048568 2 26

C0027497 GO:0006458 1 26

C0027497 GO:0000302 3 26

C0027497 GO:0030175 1 26

C0027497 GO:0008483 2 26

C0027497 GO:0030170 2 26

C0027497 GO:0008484 3 26

C0027497 GO:0030501 1 26

C0027497 GO:0008535 1 26

C0027497 GO:0051224 1 26

C0027497 GO:0051225 1 26

C0027497 GO:0003205 2 26

C0027497 GO:0032091 1 26

C0027497 GO:0016605 1 26

C0027497 GO:0003206 2 26

C0027497 GO:0015992 3 26

C0027497 GO:0015491 1 26

C0027497 GO:0042551 3 26

C0027497 GO:0006885 2 26

C0027497 GO:0042255 1 26

C0027497 GO:0003208 2 26

C0027497 GO:0042558 1 26

C0027497 GO:0042559 1 26

C0027497 GO:0070742 1 26

C0027497 GO:0043028 1 26

C0027497 GO:0005657 1 26

C0027497 GO:0051653 1 26

C0027497 GO:0043021 2 26

C0027497 GO:0043022 2 26

C0027497 GO:0042826 1 26

C0027497 GO:0051972 1 26

C0027497 GO:0006303 1 26

C0027497 GO:0070169 1 26

C0027497 GO:0007622 1 26

C0027497 GO:0016780 1 26

C0027497 GO:0009304 4 26

C0027497 GO:0016653 3 26

C0027497 GO:0048709 2 26

C0027497 GO:0009303 1 26

C0027497 GO:0046466 1 26

C0027497 GO:0060606 1 26

C0027497 GO:0009260 2 26

C0027497 GO:0007052 1 26

C0027497 GO:0009262 1 26

C0027497 GO:0009264 1 26

C0027497 GO:0009267 1 26

C0027497 GO:0009266 2 26

C0027497 GO:0032890 1 26

C0027497 GO:0019395 1 26

C0027497 GO:0035303 1 26

C0027497 GO:0044042 1 26

C0027497 GO:0030675 1 26

C0027497 GO:0030672 1 26

C0027497 GO:0031080 2 26

C0027497 GO:0002889 1 26

C0027497 GO:0004536 1 26

C0027497 GO:0006000 1 26

C0027497 GO:0015781 1 26

C0027497 GO:0015780 1 26

C0027497 GO:0004532 1 26

C0027497 GO:0050654 1 26

C0027497 GO:0042791 3 26

C0027497 GO:0006691 1 26

C0027497 GO:0015074 1 26

C0027497 GO:0005003 1 26

C0027497 GO:0016676 3 26

C0027497 GO:0005007 1 26

C0027497 GO:0016675 3 26

C0027497 GO:0006752 1 26

C0027497 GO:0006026 1 26

C0027497 GO:0006027 1 26

C0027497 GO:0016783 1 26

C0027497 GO:0030880 2 26

C0027497 GO:0033613 1 26

C0027497 GO:0008143 2 26

C0027497 GO:0033209 3 26

C0027497 GO:0008144 3 26

C0027497 GO:0042633 1 26

C0027497 GO:0009410 1 26

C0027497 GO:0042177 2 26

C0027497 GO:0018210 1 26

C0027497 GO:0051453 1 26

C0027497 GO:0031498 1 26

C0027497 GO:0004691 2 26

C0027497 GO:0004690 2 26

C0027497 GO:0004697 1 26

C0027497 GO:0030261 2 26

C0027497 GO:0009395 1 26

C0027497 GO:0009394 1 26

C0027497 GO:0000127 3 26

C0027497 GO:0042267 2 26

C0027497 GO:0045178 2 26

C0027497 GO:0031532 1 26

C0027497 GO:0033275 1 26

C0027497 GO:0002228 2 26

C0027497 GO:0045741 1 26

C0027497 GO:0045621 1 26

C0027497 GO:0045620 1 26

C0027497 GO:0005506 1 26

C0027497 GO:0045622 1 26

C0027497 GO:0002221 1 26

C0027497 GO:0033108 3 26

C0027497 GO:0055002 1 26

C0027497 GO:0008287 1 26

C0027497 GO:0006879 1 26

C0027497 GO:0006073 1 26

C0027497 GO:0042288 2 26

C0027497 GO:0050879 1 26

C0027497 GO:0055008 1 26

C0027497 GO:0051017 1 26

C0027497 GO:0051015 1 26

C0027497 GO:0005913 1 26

C0027497 GO:0001656 2 26

C0027497 GO:0046504 1 26

C0027497 GO:0046889 2 26

C0027497 GO:0033293 1 26

C0027497 GO:0044253 2 26

C0027497 GO:0030201 1 26

C0027497 GO:0043666 3 26

C0027497 GO:0005786 2 26

C0027497 GO:0045884 2 26

C0027497 GO:0006337 1 26

C0027497 GO:0032369 1 26

C0027497 GO:0006099 2 26

C0027497 GO:0016528 1 26

C0027497 GO:0016529 1 26

C0027497 GO:0004190 1 26

C0027497 GO:0003899 2 26

C0027497 GO:0042797 3 26

C0027497 GO:0016254 1 26

C0027497 GO:0016255 1 26

C0027497 GO:0017046 1 26

C0027497 GO:0045923 1 26

C0027497 GO:0019047 1 26

C0027497 GO:0001516 1 26

C0027497 GO:0018065 1 26

C0027497 GO:0017124 1 26

C0027497 GO:0017048 2 26

C0027497 GO:0060021 1 26

C0027497 GO:0008213 1 26

C0027497 GO:0042593 1 26

C0027497 GO:0022404 1 26

C0027497 GO:0008210 1 26

C0027497 GO:0001889 1 26

C0027497 GO:0042594 2 26

C0027497 GO:0043631 1 26

C0027497 GO:0008603 2 26

C0027497 GO:0006096 2 26

C0027497 GO:0022409 1 26

C0027497 GO:0045446 1 26

C0027497 GO:0009820 3 26

C0027497 GO:0033993 1 26

C0027497 GO:0050996 1 26

C0027497 GO:0006527 1 26

C0027497 GO:0017080 1 26

C0027497 GO:0032210 1 26

C0027497 GO:0003730 2 26

C0027497 GO:0045814 2 26

C0027497 GO:0019210 1 26

C0027497 GO:0019213 1 26

C0027497 GO:0045089 2 26

C0027497 GO:0006706 1 26

C0027497 GO:0070120 1 26

C0027497 GO:0070822 1 26

C0027497 GO:0051291 1 26

C0027497 GO:0048592 1 26

C0027497 GO:0048593 2 26

C0027497 GO:0000272 3 26

C0027497 GO:0031047 1 26

C0027497 GO:0030104 1 26

C0027497 GO:0000445 4 26

C0027497 GO:0015662 1 26

C0027497 GO:0050750 2 26

C0027497 GO:0001838 1 26

C0027497 GO:0070566 1 26

C0027497 GO:0040017 2 26

C0027497 GO:0030532 1 26

C0027497 GO:0009913 2 26

C0027497 GO:0003746 2 26

C0027497 GO:0008517 2 26

C0027497 GO:0043535 1 26

C0027497 GO:0001836 1 26

C0027497 GO:0001837 2 26

C0027497 GO:0005765 1 26

C0027497 GO:0043534 1 26

C0027497 GO:0005689 1 26

C0027497 GO:0005763 1 26

C0027497 GO:0048525 1 26

C0027497 GO:0048524 3 26

C0027497 GO:0051806 1 26

C0027497 GO:0006413 1 26

C0027497 GO:0048520 1 26

C0027497 GO:0019363 1 26

C0027497 GO:0019362 2 26

C0027497 GO:0006633 1 26

C0027497 GO:0042133 2 26

C0027497 GO:0034446 1 26

C0027497 GO:0000086 1 26

C0027497 GO:0007612 1 26

C0027497 GO:0000080 2 26

C0027497 GO:0009451 1 26

C0027497 GO:0000956 1 26

C0027497 GO:0031264 1 26

C0027497 GO:0007163 1 26

C0027497 GO:0003015 1 26

C0027497 GO:0019915 1 26

C0027497 GO:0042765 2 26

C0027497 GO:0016597 3 26

C0027497 GO:0009295 1 26

C0027497 GO:0016790 1 26

C0027497 GO:0042446 1 26

C0027497 GO:0004364 1 26

C0027497 GO:0048306 1 26

C0027497 GO:0004402 2 26

C0027497 GO:0010257 3 26

C0027497 GO:0005546 1 26

C0027497 GO:0045667 1 26

C0027497 GO:0001909 1 26

C0027497 GO:0006733 2 26

C0027497 GO:0070513 1 26

C0027497 GO:0032135 1 26

C0027497 GO:0045669 1 26

C0027497 GO:0015002 3 26

C0027497 GO:0033032 3 26

C0027497 GO:0034366 1 26

C0027497 GO:0051119 1 26

C0027497 GO:0043968 1 26

C0027497 GO:0005310 2 26

C0027497 GO:0016209 5 26

C0027497 GO:0016893 2 26

C0027497 GO:0008170 2 26

C0027497 GO:0016891 2 26

C0027497 GO:0000796 2 26

C0027497 GO:0000794 1 26

C0027497 GO:0016895 1 26

C0027497 GO:0009074 2 26

C0027497 GO:0051702 2 26

C0027497 GO:0035148 1 26

C0027497 GO:0042992 1 26

C0027497 GO:0014020 1 26

C0027497 GO:0031669 1 26

C0027497 GO:0018209 1 26

C0027497 GO:0000159 1 26

C0027497 GO:0005828 1 26

C0027497 GO:0016566 3 26

C0027497 GO:0004707 1 26

C0027497 GO:0016291 1 26

C0027497 GO:0007043 1 26

C0027497 GO:0005388 1 26

C0027497 GO:0016444 2 26

C0027497 GO:0004033 1 26

C0027497 GO:0018024 2 26

C0027497 GO:0006297 2 26

C0027497 GO:0017166 2 26

C0027497 GO:0015851 3 26

C0027497 GO:0007040 1 26

C0027497 GO:0034062 2 26

C0027497 GO:0046716 1 26

C0027497 GO:0008250 1 26

C0027497 GO:0052126 1 26

C0027497 GO:0048009 2 26

C0027497 GO:0001764 2 26

C0027497 GO:0046496 2 26

C0027497 GO:0005929 2 26

C0027497 GO:0031345 1 26

C0027497 GO:0045088 1 26

C0027497 GO:0051184 1 26

C0027497 GO:0051187 2 26

C0027497 GO:0051181 3 26

C0027497 GO:0051180 1 26

C0027497 GO:0051183 2 26

C0027497 GO:0042698 1 26

C0027497 GO:0016801 1 26

C0027497 GO:0042098 1 26

C0027497 GO:0031123 1 26

C0027497 GO:0008088 1 26

C0027497 GO:0016896 1 26

C0027497 GO:0043331 1 26

C0027497 GO:0016363 1 26

C0027497 GO:0007172 1 26

C0027497 GO:0030149 1 26

C0027497 GO:0032813 1 26

C0027497 GO:0009881 1 26

C0027497 GO:0006221 1 26

C0027497 GO:0050792 2 26

C0027497 GO:0032768 1 26

C0027497 GO:0032769 2 26

C0027497 GO:0017015 2 26

C0027497 GO:0019319 3 26

C0027497 GO:0050795 1 26

C0027497 GO:0003995 2 26

C0027497 GO:0002263 1 26

C0027497 GO:0008494 2 26

C0027497 GO:0051238 1 26

C0027497 GO:0003231 2 26

C0027497 GO:0030069 1 26

C0027497 GO:0006911 1 26

C0027497 GO:0046660 3 26

C0027497 GO:0046148 1 26

C0027497 GO:0051092 2 26

C0027497 GO:0016944 1 26

C0027497 GO:0008652 1 26

C0027497 GO:0006891 1 26

C0027497 GO:0008656 1 26

C0027497 GO:0006895 1 26

C0027497 GO:0042542 4 26

C0027497 GO:0022898 2 26

C0027497 GO:0010714 2 26

C0027497 GO:0010712 2 26

C0027497 GO:0070198 1 26

C0027497 GO:0043154 2 26

C0027497 GO:0046824 1 26

C0027497 GO:0046823 1 26

C0027497 GO:0043014 1 26

C0027497 GO:0006518 2 26

C0027497 GO:0007031 1 26

C0027497 GO:0001942 1 26

C0027497 GO:0019897 1 26

C0027497 GO:0016796 2 26

C0027497 GO:0006637 1 26

C0027497 GO:0006636 1 26

C0027497 GO:0006635 1 26

C0027497 GO:0019674 1 26

C0027497 GO:0004521 2 26

C0027497 GO:0009311 1 26

C0027497 GO:0004520 1 26

C0027497 GO:0042401 1 26

C0027497 GO:0031307 1 26

C0027497 GO:0032967 2 26

C0027497 GO:0004859 2 26

C0027497 GO:0007041 1 26

C0027497 GO:0032963 1 26

C0027497 GO:0000428 2 26

C0027497 GO:0005545 2 26

C0027497 GO:0004529 1 26

C0027497 GO:0022602 1 26

C0027497 GO:0016706 1 26

C0027497 GO:0004527 2 26

C0027497 GO:0004526 1 26

C0027497 GO:0031099 1 26

C0027497 GO:0001945 2 26

C0027497 GO:0050768 1 26

C0027497 GO:0050769 3 26

C0027497 GO:0032409 2 26

C0027497 GO:0015166 1 26

C0027497 GO:0016667 1 26

C0027497 GO:0009925 1 26

C0027497 GO:0031396 1 26

C0027497 GO:0031397 2 26

C0027497 GO:0000777 1 26

C0027497 GO:0021953 1 26

C0027497 GO:0006740 3 26

C0027497 GO:0005355 1 26

C0027497 GO:0050999 1 26

C0027497 GO:0042169 1 26

C0027497 GO:0015165 2 26

C0027497 GO:0005217 2 26

C0027497 GO:0016578 1 26

C0027497 GO:0006929 1 26

C0027497 GO:0046966 1 26

C0027497 GO:0021954 1 26

C0027497 GO:0014065 1 26

C0027497 GO:0006595 2 26

C0027497 GO:0055067 2 26

C0027497 GO:0007033 1 26

C0027497 GO:0031625 3 26

C0027497 GO:0002460 1 26

C0027497 GO:0030174 1 26

C0027497 GO:0045069 1 26

C0027497 GO:0048167 1 26

C0027497 GO:0009060 1 26

C0027497 GO:0034405 2 26

C0027497 GO:0009084 2 26

C0027497 GO:0016018 3 26

C0027497 GO:0032404 2 26

C0027497 GO:0005104 1 26

C0027497 GO:0004177 2 26

C0027497 GO:0016331 1 26

C0027497 GO:0050868 1 26

C0027497 GO:0055010 2 26

C0027497 GO:0046365 2 26

C0027497 GO:0046364 4 26

C0027497 GO:0016234 2 26

C0027497 GO:0030983 1 26

C0027497 GO:0046040 2 26

C0027497 GO:0009109 4 26

C0027497 GO:0034623 1 26

C0027497 GO:0034235 2 26

C0027497 GO:0051149 1 26

C0027497 GO:0051148 1 26

C0027497 GO:0000175 1 26

C0027497 GO:0001523 1 26

C0027497 GO:0070279 2 26

C0027497 GO:0016846 1 26

C0027497 GO:0016840 1 26

C0027497 GO:0007569 1 26

C0027497 GO:0004180 3 26

C0027497 GO:0000060 2 26

C0027497 GO:0005814 1 26

C0027497 GO:0033628 1 26

C0027497 GO:0016248 1 26

C0027497 GO:0016246 1 26

C0027497 GO:0018279 1 26

C0027497 GO:0031576 1 26

C0027497 GO:0070301 4 26

C0027497 GO:0017119 1 26

C0027497 GO:0045939 2 26

C0027497 GO:0031124 1 26

C0027497 GO:0010812 1 26

C0027497 GO:0050690 2 26

C0027497 GO:0022417 3 26

C0027497 GO:0016903 4 26

C0027497 GO:0022410 1 26

C0027497 GO:0007266 3 26

C0027497 GO:0000146 1 26

C0027497 GO:0055088 1 26

C0027497 GO:0006970 1 26

C0027497 GO:0002791 1 26

C0027497 GO:0070717 2 26

C0027497 GO:0005851 2 26

C0027497 GO:0009112 2 26

C0027497 GO:0052192 1 26

C0027497 GO:0003706 1 26

C0027497 GO:0006144 1 26

C0027497 GO:0044409 1 26

C0027497 GO:0070652 1 26

C0027497 GO:0006390 1 26

C0027497 GO:0009206 1 26

C0027497 GO:0000149 1 26

C0027497 GO:0008209 1 26

C0027497 GO:0006479 1 26

C0027497 GO:0005782 1 26

C0027497 GO:0032434 1 26

C0027497 GO:0034440 1 26

C0027497 GO:0006471 1 26

C0027497 GO:0032387 2 26

C0027497 GO:0048469 2 26

C0027497 GO:0005788 2 26

C0027497 GO:0006779 1 26

C0027497 GO:0030509 1 26

C0027497 GO:0030111 2 26

C0027497 GO:0004955 1 26

C0027497 GO:0031057 1 26

C0027497 GO:0031056 1 26

C0027497 GO:0031058 1 26

C0027497 GO:0030119 2 26

C0027497 GO:0030118 4 26

C0027497 GO:0005391 1 26

C0027497 GO:0005779 1 26

C0027497 GO:0048863 1 26

C0027497 GO:0016628 1 26

C0027497 GO:0002687 1 26

C0027497 GO:0006783 1 26

C0027497 GO:0007213 1 26

C0027497 GO:0016627 3 26

C0027497 GO:0001824 1 26

C0027497 GO:0007216 2 26

C0027497 GO:0016620 3 26

C0027497 GO:0070001 1 26

C0027497 GO:0048512 1 26

C0027497 GO:0002685 1 26

C0027497 GO:0010720 1 26

C0027497 GO:0010721 1 26

C0027497 GO:0031907 1 26

C0027497 GO:0019359 1 26

C0027497 GO:0005678 2 26

C0027497 GO:0043574 1 26

C0027497 GO:0032722 1 26

C0027497 GO:0046635 1 26

C0027497 GO:0031901 1 26

C0027497 GO:0034404 1 26

C0027497 GO:0015718 1 26

C0027497 GO:0016799 2 26

C0027497 GO:0043473 2 26

C0027497 GO:0005092 1 26

C0027497 GO:0032153 1 26

C0027497 GO:0032155 1 26

C0027497 GO:0006607 1 26

C0027497 GO:0005095 3 26

C0027497 GO:0031274 1 26

C0027497 GO:0019903 1 26

C0027497 GO:0045187 1 26

C0027497 GO:0008585 1 26

C0027497 GO:0031272 1 26

C0027497 GO:0007076 1 26

C0027497 GO:0043536 1 26

C0027497 GO:0055117 1 26

C0027497 GO:0003009 1 26

C0027497 GO:0051096 2 26

C0027497 GO:0032479 2 26

C0027497 GO:0051095 2 26

C0027497 GO:0060395 1 26

C0027497 GO:0050732 1 26

C0027497 GO:0043449 1 26

C0027497 GO:0005527 2 26

C0027497 GO:0033135 1 26

C0027497 GO:0043523 1 26

C0027497 GO:0004550 3 26

C0027497 GO:0043027 1 26

C0027497 GO:0033558 1 26

C0027497 GO:0002700 1 26

C0027497 GO:0006672 3 26

C0027497 GO:0045494 1 26

C0027497 GO:0043525 1 26

C0027497 GO:0006921 3 26

C0027497 GO:0042398 1 26

C0027497 GO:0045739 1 26

C0027497 GO:0009142 1 26

C0027497 GO:0045309 2 26

C0027497 GO:0009145 1 26

C0027497 GO:0016278 2 26

C0027497 GO:0016279 2 26

C0027497 GO:0009062 2 26

C0027497 GO:0000783 1 26

C0027497 GO:0000782 1 26

C0027497 GO:0016885 2 26

C0027497 GO:0009066 1 26

C0027497 GO:0009065 2 26

C0027497 GO:0009064 1 26

C0027497 GO:0031334 1 26

C0027497 GO:0048168 1 26

C0027497 GO:0035004 1 26

C0027497 GO:0070918 2 26

C0027497 GO:0031513 1 26

C0027497 GO:0042625 1 26

C0027497 GO:0031572 1 26

C0027497 GO:0045116 1 26

C0027497 GO:0090100 1 26

C0027497 GO:0031571 1 26

C0027497 GO:0005852 4 26

C0027497 GO:0043548 2 26

C0027497 GO:0031672 1 26

C0027497 GO:0051896 1 26

C0027497 GO:0016575 1 26

C0027497 GO:0016574 1 26

C0027497 GO:0005528 2 26

C0027497 GO:0005522 2 26

C0027497 GO:0048641 1 26

C0027497 GO:0007004 1 26

C0027497 GO:0016471 2 26

C0027497 GO:0004129 3 26

C0027497 GO:0004128 3 26

C0027497 GO:0006818 2 26

C0027497 GO:0055029 2 26

C0027497 GO:0016303 1 26

C0027497 GO:0001776 1 26

C0027497 GO:0045334 1 26

C0027497 GO:0006189 2 26

C0027497 GO:0006188 2 26

C0027497 GO:0042308 1 26

C0027497 GO:0006978 1 26

C0027497 GO:0042306 1 26

C0027497 GO:0034656 1 26

C0027497 GO:0042303 1 26

C0027497 GO:0008308 1 26

C0027497 GO:0000578 1 26

C0027497 GO:0044275 5 26

C0027497 GO:0070206 1 26

C0027497 GO:0046902 2 26

C0027497 GO:0000738 3 26

C0027497 GO:0006953 1 26

C0027497 GO:0001654 1 26

C0027497 GO:0000737 1 26

C0027497 GO:0002758 1 26

C0027497 GO:0016831 1 26

C0027497 GO:0006739 2 26

C0027497 GO:0004601 4 26

C0027497 GO:0004602 3 26

C0027497 GO:0032770 2 26

C0027497 GO:0043044 1 26

C0027497 GO:0030159 1 26

C0027497 GO:0010921 1 26

C0027497 GO:0045055 1 26

C0027497 GO:0042772 1 26

C0027497 GO:0051828 1 26

C0027497 GO:0017022 1 26

C0027497 GO:0010927 1 26

C0027497 GO:0045580 2 26

C0027497 GO:0034654 1 26

C0027497 GO:0045582 2 26

C0027497 GO:0005662 2 26

C0027497 GO:0046677 1 26

C0027497 GO:0031231 1 26

C0027497 GO:0003229 2 26

C0027497 GO:0034655 1 26

C0027497 GO:0003950 1 26

C0027497 GO:0042572 1 26

C0027497 GO:0060048 1 26

C0027497 GO:0030018 1 26

C0027497 GO:0034399 1 26

C0027497 GO:0050881 1 26

C0027497 GO:0015278 1 26

C0027497 GO:0001707 2 26

C0027497 GO:0001704 2 26

C0027497 GO:0051087 1 26

C0027497 GO:0015370 1 26

C0027497 GO:0045833 1 26

C0027497 GO:0006505 1 26

C0027497 GO:0003756 1 26

C0027497 GO:0006506 1 26

C0027497 GO:0008641 1 26

C0027497 GO:0017156 2 26

C0027497 GO:0030669 1 26

C0027497 GO:0043094 1 26

C0027497 GO:0048332 2 26

C0027497 GO:0006721 1 26

C0027497 GO:0031333 1 26

C0027497 GO:0000217 1 26

C0027497 GO:0004860 2 26

C0027497 GO:0030128 2 26

C0027497 GO:0030122 2 26

C0027497 GO:0008091 1 26

C0027497 GO:0030121 1 26

C0027497 GO:0030656 1 26

C0027497 GO:0030125 4 26

C0027497 GO:0050770 1 26

C0027497 GO:0001959 4 26

C0027497 GO:0050772 1 26

C0027497 GO:0060047 1 26

C0027497 GO:0048365 2 26

C0027497 GO:0032182 2 26

C0027497 GO:0001952 2 26

C0027497 GO:0001950 1 26

C0027497 GO:0070461 2 26

C0027497 GO:0005744 1 26

C0027497 GO:0032570 1 26

C0027497 GO:0006007 3 26

C0027497 GO:0002039 2 26

C0027497 GO:0050650 1 26

C0027497 GO:0051147 2 26

C0027497 GO:0034502 1 26

C0027497 GO:0005871 1 26

C0027497 GO:0042116 1 26

C0027497 GO:0046890 1 26

C0027497 GO:0034508 2 26

C0027497 GO:0042113 1 26

C0027497 GO:0000731 1 26

C0027497 GO:0046112 1 26

C0027497 GO:0005890 1 26

C0027497 GO:0043506 1 26

C0027497 GO:0043507 1 26

C0027497 GO:0000184 1 26

C0027497 GO:0032986 1 26

C0027497 GO:0007020 1 26

C0027497 GO:0032984 1 26

C0027497 GO:0043467 1 26

C0027497 GO:0032981 3 26

C0027497 GO:0031638 1 26

C0027497 GO:0045766 3 26

C0027497 GO:0030137 1 26

C0027497 GO:0008235 1 26

C0027497 GO:0005005 1 26

C0027497 GO:0004468 2 26

C0027497 GO:0030165 1 26

C0027497 GO:0016229 1 26

C0027497 GO:0046426 2 26

C0027497 GO:0010553 1 26

C0027497 GO:0046356 2 26

C0027497 GO:0030239 1 26

C0027497 GO:0006769 2 26

C0027497 GO:0007585 1 26

C0027497 GO:0034614 2 26

C0027497 GO:0034341 2 26

C0027497 GO:0008198 1 26

C0027497 GO:0000779 3 26

C0027497 GO:0002712 1 26

C0027497 GO:0042026 1 26

C0027497 GO:0009116 2 26

C0027497 GO:0051646 1 26

C0027497 GO:0009119 1 26

C0027497 GO:0070925 1 26

C0027497 GO:0006941 1 26

C0027497 GO:0016877 1 26

C0027497 GO:0000178 2 26

C0027497 GO:0000070 2 26

C0027497 GO:0001936 3 26

C0027497 GO:0006754 1 26

C0027497 GO:0016878 2 26

C0027497 GO:0051293 1 26

C0027497 GO:0019842 2 26

C0027497 GO:0031526 1 26

C0027497 GO:0046457 1 26

C0027497 GO:0046456 1 26

C0027497 GO:0043596 3 26

C0027497 GO:0030900 2 26

C0027497 GO:0033176 2 26

C0027497 GO:0046519 3 26

C0027497 GO:0016504 1 26

C0027497 GO:0016505 1 26

C0027497 GO:0060491 1 26

C0027497 GO:0008589 1 26

C0027497 GO:0015879 1 26

C0027497 GO:0046638 2 26

C0027497 GO:0046637 1 26

C0027497 GO:0051287 2 26

C0027497 GO:0060558 1 26

C0027497 GO:0046634 1 26

C0027497 GO:0046631 1 26

C0027497 GO:0016646 2 26

C0027497 GO:0050926 1 26

C0027497 GO:0055072 1 26

C0027497 GO:0050920 2 26

C0027497 GO:0051262 1 26

C0027497 GO:0006825 1 26

C0027497 GO:0051893 1 26

C0027497 GO:0034765 1 26

C0027497 GO:0008376 1 26

C0027497 GO:0008278 1 26

C0027497 GO:0034762 1 26

C0027497 GO:0031984 1 26

C0027497 GO:0006983 1 26

C0027497 GO:0018196 1 26

C0027497 GO:0015238 2 26

C0027497 GO:0006266 3 26

C0027497 GO:0043325 1 26

C0027497 GO:0010894 2 26

C0027497 GO:0010896 1 26

C0027497 GO:0006383 1 26

C0027497 GO:0032393 3 26

C0027497 GO:0007219 1 26

C0027497 GO:0014003 2 26

C0027497 GO:0009201 1 26

C0027497 GO:0000314 1 26

C0027497 GO:0022405 1 26

C0027497 GO:0030049 1 26

C0027497 GO:0030879 1 26

C0027497 GO:0006563 2 26

C0027497 GO:0010975 2 26

C0027497 GO:0030515 2 26

C0027497 GO:0030511 1 26

C0027497 GO:0045109 1 26

C0027497 GO:0070584 1 26

C0027497 GO:0005066 1 26

C0027497 GO:0005062 1 26

C0027497 GO:0051354 2 26

C0027497 GO:0031228 1 26

C0027497 GO:0031114 1 26

C0027497 GO:0005663 1 26

C0027497 GO:0043130 2 26

C0027497 GO:0005665 2 26

C0027497 GO:0048500 2 26

C0027497 GO:0019439 1 26

C0027497 GO:0019438 1 26

C0027497 GO:0060415 1 26

C0027497 GO:0045930 1 26

C0027497 GO:0005080 1 26

C0027497 GO:0002821 2 26

C0027497 GO:0032648 2 26

C0027497 GO:0005086 1 26

C0027497 GO:0001953 1 26

C0027497 GO:0042745 1 26

C0027497 GO:0042744 3 26

C0027497 GO:0042743 1 26

C0027497 GO:0032947 1 26

C0027497 GO:0015838 1 26

C0027497 GO:0043425 1 26

C0027497 GO:0007064 1 26

C0027497 GO:0031369 3 26

C0027497 GO:0055102 1 26

C0027497 GO:0042749 1 26

C0027497 GO:0008408 2 26

C0027497 GO:0042645 1 26

C0027497 GO:0016049 1 26

C0027497 GO:0045649 1 26

C0027497 GO:0051457 2 26

C0027497 GO:0008406 2 26

C0027497 GO:0035085 1 26

C0027497 GO:0015149 1 26

C0027497 GO:0010810 2 26

C0027497 GO:0001964 1 26

C0027497 GO:0033014 1 26

C0027497 GO:0006687 1 26

C0027497 GO:0006684 1 26

C0027497 GO:0016763 1 26

C0027497 GO:0001841 1 26

C0027497 GO:0001843 1 26

C0027497 GO:0016684 4 26

C0027497 GO:0015145 1 26

C0027497 GO:0016769 2 26

C0027497 GO:0000125 1 26

C0027497 GO:0030894 2 26

C0027497 GO:0042384 1 26

C0027497 GO:0046545 3 26

C0027497 GO:0009156 2 26

C0027497 GO:0005338 1 26

C0027497 GO:0033764 2 26

C0027497 GO:0008013 1 26

C0027497 GO:0007026 1 26

C0027497 GO:0001701 2 26

C0027497 GO:0043393 1 26

C0027497 GO:0048256 3 26

C0027497 GO:0000038 1 26

C0027497 GO:0043256 1 26

C0027497 GO:0043255 1 26

C0027497 GO:0000030 1 26

C0027497 GO:0043550 1 26

C0027497 GO:0045165 2 26

C0027497 GO:0000132 2 26

C0027497 GO:0007250 1 26

C0027497 GO:0004683 3 26

C0027497 GO:0031647 1 26

C0027497 GO:0030867 4 26

C0027497 GO:0031579 1 26

C0027497 GO:0002218 1 26

C0027497 GO:0045730 1 26

C0027497 GO:0045736 3 26

C0027497 GO:0016469 2 26

C0027497 GO:0015665 1 26

C0027497 GO:0030431 1 26

C0027497 GO:0046718 1 26

C0027497 GO:0016607 1 26

C0027497 GO:0002819 3 26

C0027497 GO:0045071 1 26

C0027497 GO:0051004 2 26

C0027497 GO:0030260 1 26

C0027497 GO:0030262 2 26

C0027497 GO:0051000 2 26

C0027497 GO:0048029 1 26

C0027497 GO:0016909 1 26

C0027497 GO:0009650 2 26

C0027497 GO:0043087 1 26

C0027497 GO:0009127 2 26

C0027497 GO:0044246 2 26

C0027497 GO:0090087 1 26

C0027497 GO:0006220 2 26

C0027497 GO:0010883 1 26

C0027497 GO:0070603 2 26

C0027497 GO:0001541 2 26

C0027497 GO:0006342 1 26

C0027497 GO:0042921 1 26

C0027497 GO:0046784 4 26

C0027497 GO:0046782 3 26

C0027497 GO:0044452 2 26

C0027497 GO:0008385 2 26

C0027497 GO:0080010 1 26

C0027497 GO:0022616 1 26

C0027497 GO:0070761 2 26

C0027497 GO:0010939 2 26

C0027497 GO:0003709 3 26

C0027497 GO:0019079 2 26

C0027497 GO:0046165 2 26

C0027497 GO:0070688 1 26

C0027497 GO:0015298 1 26

C0027497 GO:0035035 2 26

C0027497 GO:0006298 1 26

C0027497 GO:0051318 1 26

C0027497 GO:0030004 1 26

C0027497 GO:0010833 2 26

C0027497 GO:0051310 1 26

C0027497 GO:0008206 1 26

C0027497 GO:0008200 1 26

C0027497 GO:0055085 1 26

C0027497 GO:0006084 2 26

C0027497 GO:0008634 4 26

C0027497 GO:0008637 2 26

C0027497 GO:0005507 1 26

C0027497 GO:0043601 2 26

C0027497 GO:0006081 4 26

C0027497 GO:0043603 2 26

C0027497 GO:0005666 1 26

C0027497 GO:0042364 1 26

C0027497 GO:0070775 1 26

C0027497 GO:0042813 1 26

C0027497 GO:0003727 1 26

C0027497 GO:0006536 1 26

C0027497 GO:0043370 1 26

C0027497 GO:0045861 1 26

C0027497 GO:0034976 2 26

C0027497 GO:0000185 1 26

C0027497 GO:0002793 1 26

C0027497 GO:0019206 1 26

C0027497 GO:0055001 1 26

C0027497 GO:0008630 1 26

C0027497 GO:0000347 4 26

C0027497 GO:0000346 4 26

C0027497 GO:0030131 1 26

C0027497 GO:0016328 1 26

C0027497 GO:0030132 2 26

C0027497 GO:0030641 1 26

C0027497 GO:0050661 2 26

C0027497 GO:0040001 1 26

C0027497 GO:0032202 1 26

C0027497 GO:0070555 1 26

C0027497 GO:0033500 1 26

C0027497 GO:0016645 3 26

C0027497 GO:0008526 2 26

C0027497 GO:0050927 1 26

C0027497 GO:0031330 1 26

C0027497 GO:0005753 2 26

C0027497 GO:0010466 1 26

C0027497 GO:0070410 2 26

C0027497 GO:0030280 1 26

C0027497 GO:0010741 1 26

C0027497 GO:0042552 1 26

C0027497 GO:0048662 1 26

C0027497 GO:0070325 1 26

C0027497 GO:0048742 1 26

C0027497 GO:0019104 3 26

C0027497 GO:0042102 1 26

C0027497 GO:0005881 2 26

C0027497 GO:0005883 1 26

C0027497 GO:0006359 1 26

C0027497 GO:0007159 2 26

C0027497 GO:0000381 2 26

C0027497 GO:0007157 2 26

C0027497 GO:0002443 2 26

C0027497 GO:0002444 2 26

C0027497 GO:0070252 1 26

C0027497 GO:0002446 2 26

C0027497 GO:0042771 1 26

C0027497 GO:0042471 1 26

C0027497 GO:0002562 2 26

C0027497 GO:0016580 1 26

C0027497 GO:0016581 2 26

C0027497 GO:0050921 2 26

C0027497 GO:0001938 1 26

C0027497 GO:0034220 1 26

C0027497 GO:0033344 3 26

C0027497 GO:0004576 1 26

C0027497 GO:0002250 1 26

C0027497 GO:0004549 1 26

C0027497 GO:0015030 1 26

C0027497 GO:0001933 2 26

C0027497 GO:0004579 1 26

C0027497 GO:0001937 2 26

C0027497 GO:0015036 1 26

C0042963 GO:0006776 1 24

C0042963 GO:0010149 1 24

C0042963 GO:0007598 1 24

C0042963 GO:0043954 1 24

C0042963 GO:0048066 2 24

C0042963 GO:0002706 2 24

C0042963 GO:0002703 2 24

C0042963 GO:0034199 1 24

C0042963 GO:0002700 1 24

C0042963 GO:0001508 1 24

C0042963 GO:0016864 1 24

C0042963 GO:0016863 1 24

C0042963 GO:0016862 1 24

C0042963 GO:0009168 1 24

C0042963 GO:0016860 3 24

C0042963 GO:0000002 2 24

C0042963 GO:0043206 2 24

C0042963 GO:0005834 2 24

C0042963 GO:0006978 1 24

C0042963 GO:0004653 3 24

C0042963 GO:0019239 6 24

C0042963 GO:0006309 4 24

C0042963 GO:0030914 1 24

C0042963 GO:0004716 2 24

C0042963 GO:0009218 1 24

C0042963 GO:0007080 1 24

C0042963 GO:0030856 1 24

C0042963 GO:0016514 2 24

C0042963 GO:0016101 1 24

C0042963 GO:0030330 1 24

C0042963 GO:0051294 2 24

C0042963 GO:0002824 2 24

C0042963 GO:0003923 2 24

C0042963 GO:0002822 4 24

C0042963 GO:0031109 2 24

C0042963 GO:0021510 2 24

C0042963 GO:0035270 1 24

C0042963 GO:0002673 1 24

C0042963 GO:0000718 1 24

C0042963 GO:0006278 1 24

C0042963 GO:0034284 1 24

C0042963 GO:0016812 2 24

C0042963 GO:0006271 1 24

C0042963 GO:0048500 1 24

C0042963 GO:0045259 2 24

C0042963 GO:0016814 5 24

C0042963 GO:0010887 2 24

C0042963 GO:0010885 1 24

C0042963 GO:0010884 1 24

C0042963 GO:0006376 1 24

C0042963 GO:0004622 1 24

C0042963 GO:0006378 1 24

C0042963 GO:0010888 1 24

C0042963 GO:0021537 1 24

C0042963 GO:0019320 1 24

C0042963 GO:0031958 1 24

C0042963 GO:0032412 2 24

C0042963 GO:0017002 1 24

C0042963 GO:0001953 2 24

C0042963 GO:0006458 1 24

C0042963 GO:0000302 3 24

C0042963 GO:0030175 1 24

C0042963 GO:0008483 2 24

C0042963 GO:0030170 2 24

C0042963 GO:0008484 3 24

C0042963 GO:0005337 1 24

C0042963 GO:0005487 1 24

C0042963 GO:0005024 1 24

C0042963 GO:0051224 1 24

C0042963 GO:0051225 1 24

C0042963 GO:0003205 2 24

C0042963 GO:0016605 2 24

C0042963 GO:0003206 2 24

C0042963 GO:0015992 3 24

C0042963 GO:0015491 2 24

C0042963 GO:0042551 3 24

C0042963 GO:0006885 2 24

C0042963 GO:0004521 2 24

C0042963 GO:0042255 1 24

C0042963 GO:0003208 2 24

C0042963 GO:0042558 2 24

C0042963 GO:0042559 1 24

C0042963 GO:0070742 2 24

C0042963 GO:0002687 1 24

C0042963 GO:0002685 1 24

C0042963 GO:0051653 1 24

C0042963 GO:0050792 1 24

C0042963 GO:0043021 2 24

C0042963 GO:0043022 2 24

C0042963 GO:0051879 1 24

C0042963 GO:0051972 1 24

C0042963 GO:0006303 1 24

C0042963 GO:0007622 1 24

C0042963 GO:0009309 1 24

C0042963 GO:0009304 3 24

C0042963 GO:0032479 2 24

C0042963 GO:0048709 2 24

C0042963 GO:0009303 1 24

C0042963 GO:0033764 2 24

C0042963 GO:0009260 3 24

C0042963 GO:0007052 1 24

C0042963 GO:0009262 2 24

C0042963 GO:0009264 2 24

C0042963 GO:0009267 1 24

C0042963 GO:0008630 2 24

C0042963 GO:0032890 1 24

C0042963 GO:0019395 1 24

C0042963 GO:0035303 1 24

C0042963 GO:0044042 1 24

C0042963 GO:0030675 1 24

C0042963 GO:0050716 1 24

C0042963 GO:0031080 2 24

C0042963 GO:0004536 1 24

C0042963 GO:0006000 1 24

C0042963 GO:0015781 1 24

C0042963 GO:0015780 1 24

C0042963 GO:0004532 1 24

C0042963 GO:0050654 1 24

C0042963 GO:0006691 1 24

C0042963 GO:0070567 1 24

C0042963 GO:0005003 1 24

C0042963 GO:0016676 3 24

C0042963 GO:0005007 1 24

C0042963 GO:0016675 3 24

C0042963 GO:0006026 1 24

C0042963 GO:0006027 1 24

C0042963 GO:0016783 1 24

C0042963 GO:0030880 1 24

C0042963 GO:0033613 1 24

C0042963 GO:0008143 1 24

C0042963 GO:0033209 3 24

C0042963 GO:0008144 2 24

C0042963 GO:0042633 1 24

C0042963 GO:0031228 1 24

C0042963 GO:0042177 2 24

C0042963 GO:0018210 1 24

C0042963 GO:0050718 1 24

C0042963 GO:0051453 1 24

C0042963 GO:0031498 1 24

C0042963 GO:0004691 2 24

C0042963 GO:0004690 2 24

C0042963 GO:0051457 1 24

C0042963 GO:0009395 1 24

C0042963 GO:0009394 2 24

C0042963 GO:0000127 1 24

C0042963 GO:0042267 2 24

C0042963 GO:0030705 1 24

C0042963 GO:0002228 2 24

C0042963 GO:0045741 1 24

C0042963 GO:0045621 1 24

C0042963 GO:0005506 1 24

C0042963 GO:0045622 1 24

C0042963 GO:0033108 1 24

C0042963 GO:0055002 1 24

C0042963 GO:0008287 1 24

C0042963 GO:0006879 1 24

C0042963 GO:0006073 1 24

C0042963 GO:0042288 2 24

C0042963 GO:0050879 1 24

C0042963 GO:0055008 1 24

C0042963 GO:0051015 1 24

C0042963 GO:0002637 1 24

C0042963 GO:0005913 1 24

C0042963 GO:0001656 2 24

C0042963 GO:0046504 1 24

C0042963 GO:0046889 2 24

C0042963 GO:0033293 1 24

C0042963 GO:0044253 2 24

C0042963 GO:0005786 1 24

C0042963 GO:0045884 2 24

C0042963 GO:0006337 1 24

C0042963 GO:0032369 2 24

C0042963 GO:0006099 2 24

C0042963 GO:0016528 1 24

C0042963 GO:0016529 1 24

C0042963 GO:0004190 1 24

C0042963 GO:0003899 2 24

C0042963 GO:0042797 1 24

C0042963 GO:0016254 1 24

C0042963 GO:0042791 1 24

C0042963 GO:0017046 1 24

C0042963 GO:0045923 1 24

C0042963 GO:0019047 1 24

C0042963 GO:0030032 1 24

C0042963 GO:0018065 1 24

C0042963 GO:0017048 1 24

C0042963 GO:0060021 1 24

C0042963 GO:0008213 3 24

C0042963 GO:0042593 1 24

C0042963 GO:0022404 1 24

C0042963 GO:0008210 1 24

C0042963 GO:0001889 1 24

C0042963 GO:0042594 2 24

C0042963 GO:0043631 1 24

C0042963 GO:0008603 2 24

C0042963 GO:0006096 2 24

C0042963 GO:0009820 3 24

C0042963 GO:0033993 1 24

C0042963 GO:0050996 1 24

C0042963 GO:0017080 1 24

C0042963 GO:0032210 1 24

C0042963 GO:0003730 2 24

C0042963 GO:0045814 1 24

C0042963 GO:0019210 1 24

C0042963 GO:0019213 1 24

C0042963 GO:0045089 1 24

C0042963 GO:0006706 1 24

C0042963 GO:0070120 1 24

C0042963 GO:0070822 1 24

C0042963 GO:0019218 1 24

C0042963 GO:0048592 1 24

C0042963 GO:0048593 2 24

C0042963 GO:0002444 2 24

C0042963 GO:0000272 3 24

C0042963 GO:0031047 1 24

C0042963 GO:0000445 3 24

C0042963 GO:0003746 2 24

C0042963 GO:0050999 1 24

C0042963 GO:0040017 1 24

C0042963 GO:0030532 2 24

C0042963 GO:0009913 1 24

C0042963 GO:0008517 1 24

C0042963 GO:0043535 1 24

C0042963 GO:0001836 1 24

C0042963 GO:0001837 1 24

C0042963 GO:0005765 1 24

C0042963 GO:0043534 1 24

C0042963 GO:0005763 1 24

C0042963 GO:0048524 1 24

C0042963 GO:0006413 1 24

C0042963 GO:0006144 1 24

C0042963 GO:0019363 1 24

C0042963 GO:0019362 2 24

C0042963 GO:0006633 1 24

C0042963 GO:0042133 3 24

C0042963 GO:0034446 1 24

C0042963 GO:0000086 1 24

C0042963 GO:0007612 1 24

C0042963 GO:0000080 2 24

C0042963 GO:0009451 1 24

C0042963 GO:0000956 1 24

C0042963 GO:0043449 1 24

C0042963 GO:0007163 1 24

C0042963 GO:0003015 1 24

C0042963 GO:0019915 1 24

C0042963 GO:0042765 2 24

C0042963 GO:0016597 3 24

C0042963 GO:0009295 1 24

C0042963 GO:0016790 1 24

C0042963 GO:0042446 1 24

C0042963 GO:0004364 1 24

C0042963 GO:0002263 1 24

C0042963 GO:0004402 1 24

C0042963 GO:0010257 1 24

C0042963 GO:0005546 2 24

C0042963 GO:0044452 1 24

C0042963 GO:0045667 1 24

C0042963 GO:0001909 1 24

C0042963 GO:0032135 1 24

C0042963 GO:0045669 1 24

C0042963 GO:0015002 3 24

C0042963 GO:0033032 2 24

C0042963 GO:0034366 1 24

C0042963 GO:0051119 1 24

C0042963 GO:0043968 1 24

C0042963 GO:0005310 1 24

C0042963 GO:0016209 5 24

C0042963 GO:0016893 2 24

C0042963 GO:0008170 1 24

C0042963 GO:0016891 2 24

C0042963 GO:0000796 2 24

C0042963 GO:0000794 1 24

C0042963 GO:0016895 1 24

C0042963 GO:0009074 2 24

C0042963 GO:0051702 2 24

C0042963 GO:0070279 2 24

C0042963 GO:0042992 1 24

C0042963 GO:0030261 2 24

C0042963 GO:0043236 1 24

C0042963 GO:0031669 1 24

C0042963 GO:0018209 1 24

C0042963 GO:0000159 1 24

C0042963 GO:0000018 1 24

C0042963 GO:0016566 3 24

C0042963 GO:0004707 1 24

C0042963 GO:0006942 1 24

C0042963 GO:0016291 1 24

C0042963 GO:0007043 1 24

C0042963 GO:0005388 1 24

C0042963 GO:0016444 2 24

C0042963 GO:0004033 1 24

C0042963 GO:0018024 2 24

C0042963 GO:0006297 2 24

C0042963 GO:0017166 2 24

C0042963 GO:0002839 1 24

C0042963 GO:0015851 2 24

C0042963 GO:0002834 1 24

C0042963 GO:0002837 1 24

C0042963 GO:0002836 1 24

C0042963 GO:0034062 2 24

C0042963 GO:0002833 1 24

C0042963 GO:0008250 1 24

C0042963 GO:0050820 2 24

C0042963 GO:0048009 2 24

C0042963 GO:0001764 2 24

C0042963 GO:0046496 2 24

C0042963 GO:0005929 1 24

C0042963 GO:0006707 1 24

C0042963 GO:0031345 1 24

C0042963 GO:0045088 1 24

C0042963 GO:0051184 1 24

C0042963 GO:0051187 2 24

C0042963 GO:0051181 4 24

C0042963 GO:0051180 1 24

C0042963 GO:0051183 1 24

C0042963 GO:0016801 2 24

C0042963 GO:0042098 1 24

C0042963 GO:0016896 1 24

C0042963 GO:0007183 2 24

C0042963 GO:0016363 1 24

C0042963 GO:0030149 1 24

C0042963 GO:0009881 1 24

C0042963 GO:0006221 1 24

C0042963 GO:0015711 1 24

C0042963 GO:0000339 1 24

C0042963 GO:0032768 1 24

C0042963 GO:0032769 2 24

C0042963 GO:0017015 1 24

C0042963 GO:0019319 3 24

C0042963 GO:0050795 1 24

C0042963 GO:0003995 2 24

C0042963 GO:0003231 2 24

C0042963 GO:0030069 1 24

C0042963 GO:0006911 1 24

C0042963 GO:0046660 1 24

C0042963 GO:0046148 1 24

C0042963 GO:0016944 1 24

C0042963 GO:0008652 1 24

C0042963 GO:0043666 2 24

C0042963 GO:0032368 1 24

C0042963 GO:0042542 4 24

C0042963 GO:0022898 3 24

C0042963 GO:0010714 2 24

C0042963 GO:0010712 2 24

C0042963 GO:0070198 1 24

C0042963 GO:0046824 1 24

C0042963 GO:0046823 1 24

C0042963 GO:0006518 1 24

C0042963 GO:0001942 1 24

C0042963 GO:0005544 1 24

C0042963 GO:0019897 1 24

C0042963 GO:0016796 2 24

C0042963 GO:0006637 1 24

C0042963 GO:0006636 1 24

C0042963 GO:0006635 1 24

C0042963 GO:0019674 1 24

C0042963 GO:0006733 1 24

C0042963 GO:0009311 1 24

C0042963 GO:0004520 1 24

C0042963 GO:0042401 1 24

C0042963 GO:0031307 1 24

C0042963 GO:0032967 2 24

C0042963 GO:0004859 1 24

C0042963 GO:0007041 1 24

C0042963 GO:0032963 1 24

C0042963 GO:0000428 1 24

C0042963 GO:0005545 1 24

C0042963 GO:0004529 1 24

C0042963 GO:0030201 1 24

C0042963 GO:0016706 1 24

C0042963 GO:0004527 2 24

C0042963 GO:0004526 1 24

C0042963 GO:0031099 1 24

C0042963 GO:0001945 1 24

C0042963 GO:0050768 1 24

C0042963 GO:0050769 3 24

C0042963 GO:0032409 2 24

C0042963 GO:0015166 1 24

C0042963 GO:0015165 2 24

C0042963 GO:0048568 1 24

C0042963 GO:0031396 1 24

C0042963 GO:0031397 2 24

C0042963 GO:0006744 1 24

C0042963 GO:0006743 1 24

C0042963 GO:0043034 1 24

C0042963 GO:0021953 1 24

C0042963 GO:0006740 2 24

C0042963 GO:0005355 1 24

C0042963 GO:0016127 1 24

C0042963 GO:0042169 1 24

C0042963 GO:0042162 1 24

C0042963 GO:0005217 2 24

C0042963 GO:0046466 1 24

C0042963 GO:0016278 2 24

C0042963 GO:0021954 1 24

C0042963 GO:0014065 1 24

C0042963 GO:0006595 1 24

C0042963 GO:0007033 1 24

C0042963 GO:0031625 2 24

C0042963 GO:0002460 1 24

C0042963 GO:0030174 2 24

C0042963 GO:0048167 1 24

C0042963 GO:0005513 1 24

C0042963 GO:0009060 1 24

C0042963 GO:0002366 1 24

C0042963 GO:0034405 1 24

C0042963 GO:0009084 1 24

C0042963 GO:0016018 2 24

C0042963 GO:0032404 2 24

C0042963 GO:0005104 1 24

C0042963 GO:0004177 2 24

C0042963 GO:0016331 1 24

C0042963 GO:0050868 1 24

C0042963 GO:0055010 2 24

C0042963 GO:0046365 2 24

C0042963 GO:0046364 3 24

C0042963 GO:0016234 1 24

C0042963 GO:0030983 1 24

C0042963 GO:0009108 1 24

C0042963 GO:0009109 3 24

C0042963 GO:0034623 1 24

C0042963 GO:0034235 2 24

C0042963 GO:0051149 1 24

C0042963 GO:0051148 1 24

C0042963 GO:0031526 1 24

C0042963 GO:0001523 1 24

C0042963 GO:0016846 1 24

C0042963 GO:0016840 1 24

C0042963 GO:0031532 1 24

C0042963 GO:0004180 2 24

C0042963 GO:0000060 1 24

C0042963 GO:0005814 1 24

C0042963 GO:0004675 1 24

C0042963 GO:0018279 1 24

C0042963 GO:0016607 1 24

C0042963 GO:0070301 4 24

C0042963 GO:0017119 1 24

C0042963 GO:0045939 4 24

C0042963 GO:0031124 1 24

C0042963 GO:0010812 2 24

C0042963 GO:0050690 2 24

C0042963 GO:0022417 3 24

C0042963 GO:0016903 4 24

C0042963 GO:0022410 1 24

C0042963 GO:0007266 3 24

C0042963 GO:0000146 1 24

C0042963 GO:0009746 1 24

C0042963 GO:0055088 1 24

C0042963 GO:0006970 1 24

C0042963 GO:0002791 1 24

C0042963 GO:0005851 2 24

C0042963 GO:0009112 1 24

C0042963 GO:0003706 1 24

C0042963 GO:0009749 1 24

C0042963 GO:0005662 2 24

C0042963 GO:0070652 1 24

C0042963 GO:0006390 2 24

C0042963 GO:0045806 1 24

C0042963 GO:0006479 3 24

C0042963 GO:0032024 1 24

C0042963 GO:0006775 1 24

C0042963 GO:0005782 1 24

C0042963 GO:0034440 1 24

C0042963 GO:0006471 1 24

C0042963 GO:0031970 1 24

C0042963 GO:0032387 2 24

C0042963 GO:0048469 2 24

C0042963 GO:0005788 1 24

C0042963 GO:0006779 1 24

C0042963 GO:0030509 1 24

C0042963 GO:0030111 1 24

C0042963 GO:0031056 1 24

C0042963 GO:0031058 1 24

C0042963 GO:0030119 2 24

C0042963 GO:0030118 2 24

C0042963 GO:0005391 1 24

C0042963 GO:0007219 1 24

C0042963 GO:0016628 1 24

C0042963 GO:0006783 1 24

C0042963 GO:0007213 2 24

C0042963 GO:0016627 3 24

C0042963 GO:0001824 1 24

C0042963 GO:0007216 2 24

C0042963 GO:0016620 3 24

C0042963 GO:0070001 1 24

C0042963 GO:0048512 1 24

C0042963 GO:0010720 1 24

C0042963 GO:0010721 1 24

C0042963 GO:0031907 1 24

C0042963 GO:0019359 1 24

C0042963 GO:0005678 1 24

C0042963 GO:0046635 1 24

C0042963 GO:0034404 1 24

C0042963 GO:0015718 2 24

C0042963 GO:0016799 2 24

C0042963 GO:0043473 2 24

C0042963 GO:0005092 1 24

C0042963 GO:0000096 1 24

C0042963 GO:0006607 1 24

C0042963 GO:0005095 2 24

C0042963 GO:0043536 1 24

C0042963 GO:0019903 1 24

C0042963 GO:0045187 1 24

C0042963 GO:0007076 1 24

C0042963 GO:0003009 1 24

C0042963 GO:0051096 1 24

C0042963 GO:0051095 1 24

C0042963 GO:0070566 1 24

C0042963 GO:0005527 2 24

C0042963 GO:0055072 1 24

C0042963 GO:0004550 2 24

C0042963 GO:0043027 1 24

C0042963 GO:0033558 1 24

C0042963 GO:0006672 2 24

C0042963 GO:0045494 1 24

C0042963 GO:0043525 1 24

C0042963 GO:0006921 2 24

C0042963 GO:0042398 1 24

C0042963 GO:0009142 1 24

C0042963 GO:0009145 1 24

C0042963 GO:0034379 1 24

C0042963 GO:0016279 2 24

C0042963 GO:0009062 2 24

C0042963 GO:0000783 1 24

C0042963 GO:0000782 1 24

C0042963 GO:0016885 1 24

C0042963 GO:0009066 1 24

C0042963 GO:0009065 2 24

C0042963 GO:0021700 1 24

C0042963 GO:0048168 1 24

C0042963 GO:0035004 1 24

C0042963 GO:0090087 1 24

C0042963 GO:0031576 1 24

C0042963 GO:0042625 1 24

C0042963 GO:0031572 1 24

C0042963 GO:0045116 1 24

C0042963 GO:0090100 1 24

C0042963 GO:0031571 1 24

C0042963 GO:0005852 4 24

C0042963 GO:0043548 1 24

C0042963 GO:0031672 1 24

C0042963 GO:0007006 1 24

C0042963 GO:0016575 1 24

C0042963 GO:0016574 1 24

C0042963 GO:0005528 2 24

C0042963 GO:0005522 1 24

C0042963 GO:0007004 1 24

C0042963 GO:0016471 2 24

C0042963 GO:0004129 3 24

C0042963 GO:0004128 3 24

C0042963 GO:0006818 2 24

C0042963 GO:0055029 1 24

C0042963 GO:0016303 1 24

C0042963 GO:0001776 1 24

C0042963 GO:0045334 1 24

C0042963 GO:0050810 1 24

C0042963 GO:0006188 1 24

C0042963 GO:0042308 1 24

C0042963 GO:0034654 1 24

C0042963 GO:0042306 1 24

C0042963 GO:0034656 1 24

C0042963 GO:0042303 1 24

C0042963 GO:0008308 1 24

C0042963 GO:0044275 5 24

C0042963 GO:0001570 1 24

C0042963 GO:0070206 1 24

C0042963 GO:0046902 1 24

C0042963 GO:0000738 2 24

C0042963 GO:0001654 1 24

C0042963 GO:0000737 1 24

C0042963 GO:0000731 1 24

C0042963 GO:0008535 1 24

C0042963 GO:0006739 2 24

C0042963 GO:0006189 1 24

C0042963 GO:0004601 4 24

C0042963 GO:0004602 3 24

C0042963 GO:0032770 2 24

C0042963 GO:0030159 1 24

C0042963 GO:0010921 1 24

C0042963 GO:0045055 1 24

C0042963 GO:0009266 2 24

C0042963 GO:0042772 1 24

C0042963 GO:0017022 1 24

C0042963 GO:0010927 1 24

C0042963 GO:0045580 1 24

C0042963 GO:0045582 2 24

C0042963 GO:0060047 1 24

C0042963 GO:0046677 1 24

C0042963 GO:0003229 2 24

C0042963 GO:0034655 1 24

C0042963 GO:0003950 1 24

C0042963 GO:0042572 1 24

C0042963 GO:0060048 1 24

C0042963 GO:0045426 1 24

C0042963 GO:0030018 1 24

C0042963 GO:0034399 1 24

C0042963 GO:0050881 1 24

C0042963 GO:0015278 1 24

C0042963 GO:0001707 2 24

C0042963 GO:0001704 2 24

C0042963 GO:0051087 2 24

C0042963 GO:0032370 1 24

C0042963 GO:0032373 1 24

C0042963 GO:0042375 1 24

C0042963 GO:0015370 1 24

C0042963 GO:0045833 1 24

C0042963 GO:0006505 1 24

C0042963 GO:0003756 1 24

C0042963 GO:0033135 2 24

C0042963 GO:0008641 1 24

C0042963 GO:0017156 2 24

C0042963 GO:0043094 1 24

C0042963 GO:0048332 2 24

C0042963 GO:0006721 1 24

C0042963 GO:0031333 1 24

C0042963 GO:0000217 1 24

C0042963 GO:0004860 2 24

C0042963 GO:0030128 2 24

C0042963 GO:0030122 2 24

C0042963 GO:0008091 1 24

C0042963 GO:0030121 1 24

C0042963 GO:0030656 1 24

C0042963 GO:0030125 2 24

C0042963 GO:0070688 1 24

C0042963 GO:0050770 1 24

C0042963 GO:0001959 3 24

C0042963 GO:0050772 1 24

C0042963 GO:0015884 1 24

C0042963 GO:0048365 2 24

C0042963 GO:0032182 2 24

C0042963 GO:0001952 3 24

C0042963 GO:0001950 1 24

C0042963 GO:0005742 1 24

C0042963 GO:0070461 1 24

C0042963 GO:0005744 1 24

C0042963 GO:0016653 3 24

C0042963 GO:0046040 1 24

C0042963 GO:0006007 3 24

C0042963 GO:0002039 2 24

C0042963 GO:0050650 1 24

C0042963 GO:0042054 1 24

C0042963 GO:0051147 1 24

C0042963 GO:0034502 1 24

C0042963 GO:0042116 1 24

C0042963 GO:0002709 1 24

C0042963 GO:0046890 1 24

C0042963 GO:0034508 1 24

C0042963 GO:0042113 1 24

C0042963 GO:0046112 1 24

C0042963 GO:0005890 1 24

C0042963 GO:0043506 1 24

C0042963 GO:0043507 1 24

C0042963 GO:0032986 1 24

C0042963 GO:0007020 1 24

C0042963 GO:0032984 1 24

C0042963 GO:0043467 1 24

C0042963 GO:0032981 1 24

C0042963 GO:0031638 1 24

C0042963 GO:0045766 2 24

C0042963 GO:0051291 1 24

C0042963 GO:0030137 1 24

C0042963 GO:0005005 1 24

C0042963 GO:0004468 1 24

C0042963 GO:0005112 1 24

C0042963 GO:0030165 1 24

C0042963 GO:0016229 1 24

C0042963 GO:0046426 2 24

C0042963 GO:0046356 2 24

C0042963 GO:0030239 1 24

C0042963 GO:0006769 2 24

C0042963 GO:0007585 1 24

C0042963 GO:0034614 2 24

C0042963 GO:0034341 1 24

C0042963 GO:0008198 1 24

C0042963 GO:0000779 2 24

C0042963 GO:0042026 1 24

C0042963 GO:0009116 1 24

C0042963 GO:0051646 1 24

C0042963 GO:0009119 1 24

C0042963 GO:0070925 1 24

C0042963 GO:0043523 1 24

C0042963 GO:0001533 1 24

C0042963 GO:0000178 1 24

C0042963 GO:0000070 2 24

C0042963 GO:0001516 1 24

C0042963 GO:0006754 1 24

C0042963 GO:0016878 1 24

C0042963 GO:0051293 1 24

C0042963 GO:0019842 2 24

C0042963 GO:0006359 1 24

C0042963 GO:0046457 1 24

C0042963 GO:0046456 1 24

C0042963 GO:0043596 1 24

C0042963 GO:0030900 2 24

C0042963 GO:0033176 2 24

C0042963 GO:0046519 2 24

C0042963 GO:0016504 1 24

C0042963 GO:0016505 1 24

C0042963 GO:0046638 2 24

C0042963 GO:0046637 1 24

C0042963 GO:0051287 3 24

C0042963 GO:0060558 1 24

C0042963 GO:0046634 1 24

C0042963 GO:0046631 1 24

C0042963 GO:0016646 2 24

C0042963 GO:0008276 1 24

C0042963 GO:0050920 1 24

C0042963 GO:0051262 1 24

C0042963 GO:0045309 2 24

C0042963 GO:0051893 1 24

C0042963 GO:0034765 1 24

C0042963 GO:0008376 1 24

C0042963 GO:0008278 1 24

C0042963 GO:0034762 2 24

C0042963 GO:0006983 1 24

C0042963 GO:0018196 1 24

C0042963 GO:0015238 2 24

C0042963 GO:0006266 2 24

C0042963 GO:0043325 1 24

C0042963 GO:0010894 4 24

C0042963 GO:0010896 1 24

C0042963 GO:0006383 1 24

C0042963 GO:0032393 3 24

C0042963 GO:0009206 1 24

C0042963 GO:0014003 2 24

C0042963 GO:0009201 1 24

C0042963 GO:0000314 1 24

C0042963 GO:0022405 1 24

C0042963 GO:0030879 1 24

C0042963 GO:0006563 2 24

C0042963 GO:0010975 2 24

C0042963 GO:0030515 1 24

C0042963 GO:0030511 1 24

C0042963 GO:0045109 1 24

C0042963 GO:0005062 2 24

C0042963 GO:0051354 2 24

C0042963 GO:0051219 1 24

C0042963 GO:0005663 1 24

C0042963 GO:0043130 2 24

C0042963 GO:0030669 2 24

C0042963 GO:0031123 1 24

C0042963 GO:0019439 1 24

C0042963 GO:0032731 1 24

C0042963 GO:0060415 1 24

C0042963 GO:0030194 2 24

C0042963 GO:0005088 1 24

C0042963 GO:0045930 1 24

C0042963 GO:0005080 1 24

C0042963 GO:0002821 2 24

C0042963 GO:0032648 1 24

C0042963 GO:0005086 1 24

C0042963 GO:0042745 1 24

C0042963 GO:0042744 3 24

C0042963 GO:0042743 1 24

C0042963 GO:0032947 1 24

C0042963 GO:0005871 1 24

C0042963 GO:0043425 1 24

C0042963 GO:0055067 2 24

C0042963 GO:0055102 1 24

C0042963 GO:0042749 1 24

C0042963 GO:0008408 2 24

C0042963 GO:0042645 1 24

C0042963 GO:0016049 1 24

C0042963 GO:0008235 1 24

C0042963 GO:0008406 1 24

C0042963 GO:0035085 1 24

C0042963 GO:0015149 1 24

C0042963 GO:0010810 3 24

C0042963 GO:0001964 1 24

C0042963 GO:0033014 1 24

C0042963 GO:0006687 1 24

C0042963 GO:0006684 1 24

C0042963 GO:0016763 1 24

C0042963 GO:0005665 1 24

C0042963 GO:0016684 4 24

C0042963 GO:0015145 1 24

C0042963 GO:0016769 2 24

C0042963 GO:0000125 1 24

C0042963 GO:0030894 1 24

C0042963 GO:0042384 1 24

C0042963 GO:0046545 1 24

C0042963 GO:0009156 2 24

C0042963 GO:0005338 1 24

C0042963 GO:0009152 1 24

C0042963 GO:0008013 2 24

C0042963 GO:0001701 1 24

C0042963 GO:0043393 1 24

C0042963 GO:0048256 3 24

C0042963 GO:0043256 1 24

C0042963 GO:0000030 1 24

C0042963 GO:0043550 1 24

C0042963 GO:0045165 1 24

C0042963 GO:0000132 2 24

C0042963 GO:0007250 1 24

C0042963 GO:0006941 1 24

C0042963 GO:0004683 2 24

C0042963 GO:0031647 1 24

C0042963 GO:0030867 4 24

C0042963 GO:0031579 1 24

C0042963 GO:0045730 1 24

C0042963 GO:0045736 2 24

C0042963 GO:0005246 1 24

C0042963 GO:0016469 2 24

C0042963 GO:0015665 1 24

C0042963 GO:0030431 1 24

C0042963 GO:0032376 1 24

C0042963 GO:0015074 1 24

C0042963 GO:0002819 3 24

C0042963 GO:0051004 2 24

C0042963 GO:0030262 2 24

C0042963 GO:0051000 2 24

C0042963 GO:0048029 1 24

C0042963 GO:0016909 1 24

C0042963 GO:0009650 2 24

C0042963 GO:0043087 1 24

C0042963 GO:0009127 1 24

C0042963 GO:0044246 2 24

C0042963 GO:0006220 2 24

C0042963 GO:0010883 1 24

C0042963 GO:0070603 2 24

C0042963 GO:0001541 1 24

C0042963 GO:0006342 1 24

C0042963 GO:0042921 1 24

C0042963 GO:0046784 3 24

C0042963 GO:0046782 2 24

C0042963 GO:0000175 1 24

C0042963 GO:0008385 1 24

C0042963 GO:0022616 2 24

C0042963 GO:0070761 1 24

C0042963 GO:0010939 2 24

C0042963 GO:0003709 3 24

C0042963 GO:0019079 2 24

C0042963 GO:0046165 2 24

C0042963 GO:0006506 1 24

C0042963 GO:0015298 2 24

C0042963 GO:0035035 1 24

C0042963 GO:0006298 1 24

C0042963 GO:0051318 1 24

C0042963 GO:0030004 1 24

C0042963 GO:0010833 2 24

C0042963 GO:0051310 1 24

C0042963 GO:0015858 1 24

C0042963 GO:0055085 1 24

C0042963 GO:0006084 2 24

C0042963 GO:0008634 3 24

C0042963 GO:0008637 2 24

C0042963 GO:0005507 1 24

C0042963 GO:0043601 1 24

C0042963 GO:0006081 4 24

C0042963 GO:0043603 2 24

C0042963 GO:0070776 1 24

C0042963 GO:0070775 1 24

C0042963 GO:0042813 1 24

C0042963 GO:0043370 1 24

C0042963 GO:0045861 1 24

C0042963 GO:0034976 1 24

C0042963 GO:0019200 1 24

C0042963 GO:0000184 1 24

C0042963 GO:0019206 1 24

C0042963 GO:0055001 1 24

C0042963 GO:0000347 3 24

C0042963 GO:0000346 3 24

C0042963 GO:0030131 1 24

C0042963 GO:0030132 2 24

C0042963 GO:0031369 2 24

C0042963 GO:0030641 1 24

C0042963 GO:0050661 1 24

C0042963 GO:0040001 1 24

C0042963 GO:0016725 1 24

C0042963 GO:0032202 1 24

C0042963 GO:0070555 1 24

C0042963 GO:0042516 1 24

C0042963 GO:0033500 1 24

C0042963 GO:0016645 3 24

C0042963 GO:0008526 2 24

C0042963 GO:0031330 1 24

C0042963 GO:0005753 2 24

C0042963 GO:0010466 1 24

C0042963 GO:0070410 1 24

C0042963 GO:0006400 1 24

C0042963 GO:0030280 1 24

C0042963 GO:0010741 1 24

C0042963 GO:0042552 1 24

C0042963 GO:0048662 1 24

C0042963 GO:0031334 1 24

C0042963 GO:0019104 3 24

C0042963 GO:0042102 1 24

C0042963 GO:0005881 2 24

C0042963 GO:0005883 1 24

C0042963 GO:0007159 2 24

C0042963 GO:0000381 2 24

C0042963 GO:0007157 2 24

C0042963 GO:0002443 2 24

C0042963 GO:0051896 1 24

C0042963 GO:0002446 2 24

C0042963 GO:0042771 2 24

C0042963 GO:0002562 2 24

C0042963 GO:0016580 1 24

C0042963 GO:0016581 2 24

C0042963 GO:0050921 1 24

C0042963 GO:0001938 2 24

C0042963 GO:0034220 1 24

C0042963 GO:0033344 2 24

C0042963 GO:0004576 1 24

C0042963 GO:0002250 1 24

C0042963 GO:0004549 1 24

C0042963 GO:0015030 1 24

C0042963 GO:0001933 2 24

C0042963 GO:0004579 1 24

C0042963 GO:0001937 3 24

C0042963 GO:0001936 3 24

C0027769 GO:0005786 1 9

C0027769 GO:0007598 1 9

C0027769 GO:0022624 1 9

C0027769 GO:0048066 1 9

C0027769 GO:0002706 2 9

C0027769 GO:0002703 1 9

C0027769 GO:0002700 1 9

C0027769 GO:0016866 1 9

C0027769 GO:0002709 2 9

C0027769 GO:0005838 1 9

C0027769 GO:0002366 1 9

C0027769 GO:0051294 1 9

C0027769 GO:0002824 2 9

C0027769 GO:0002822 2 9

C0027769 GO:0031109 1 9

C0027769 GO:0002821 2 9

C0027769 GO:0007281 1 9

C0027769 GO:0035270 1 9

C0027769 GO:0003841 1 9

C0027769 GO:0006278 1 9

C0027769 GO:0034284 1 9

C0027769 GO:0006271 1 9

C0027769 GO:0016814 2 9

C0027769 GO:0010887 2 9

C0027769 GO:0010885 1 9

C0027769 GO:0031958 1 9

C0027769 GO:0017002 1 9

C0027769 GO:0000428 1 9

C0027769 GO:0006595 1 9

C0027769 GO:0016607 1 9

C0027769 GO:0016605 1 9

C0027769 GO:0015992 1 9

C0027769 GO:0015491 2 9

C0027769 GO:0006885 1 9

C0027769 GO:0042255 2 9

C0027769 GO:0042558 1 9

C0027769 GO:0070742 1 9

C0027769 GO:0002687 2 9

C0027769 GO:0002685 2 9

C0027769 GO:0009168 2 9

C0027769 GO:0043021 1 9

C0027769 GO:0043022 1 9

C0027769 GO:0007622 1 9

C0027769 GO:0032479 1 9

C0027769 GO:0048709 1 9

C0027769 GO:0033764 1 9

C0027769 GO:0009260 1 9

C0027769 GO:0007052 1 9

C0027769 GO:0009262 1 9

C0027769 GO:0009264 1 9

C0027769 GO:0044042 1 9

C0027769 GO:0050716 1 9

C0027769 GO:0031080 3 9

C0027769 GO:0050714 1 9

C0027769 GO:0004536 1 9

C0027769 GO:0006000 1 9

C0027769 GO:0050718 1 9

C0027769 GO:0015074 1 9

C0027769 GO:0048365 1 9

C0027769 GO:0030880 1 9

C0027769 GO:0008143 1 9

C0027769 GO:0019047 1 9

C0027769 GO:0004693 1 9

C0027769 GO:0009394 1 9

C0027769 GO:0043560 1 9

C0027769 GO:0043484 1 9

C0027769 GO:0002228 2 9

C0027769 GO:0045621 1 9

C0027769 GO:0045622 1 9

C0027769 GO:0045749 1 9

C0027769 GO:0033108 1 9

C0027769 GO:0030669 1 9

C0027769 GO:0001656 1 9

C0027769 GO:0006978 1 9

C0027769 GO:0042542 1 9

C0027769 GO:0003899 2 9

C0027769 GO:0017046 1 9

C0027769 GO:0045923 1 9

C0027769 GO:0018105 1 9

C0027769 GO:0030032 1 9

C0027769 GO:0060021 1 9

C0027769 GO:0008213 1 9

C0027769 GO:0008603 1 9

C0027769 GO:0003730 1 9

C0027769 GO:0070668 1 9

C0027769 GO:0006707 1 9

C0027769 GO:0070120 1 9

C0027769 GO:0000445 2 9

C0027769 GO:0030530 1 9

C0027769 GO:0040017 1 9

C0027769 GO:0030532 1 9

C0027769 GO:0045187 1 9

C0027769 GO:0006413 1 9

C0027769 GO:0019362 1 9

C0027769 GO:0042133 1 9

C0027769 GO:0007612 1 9

C0027769 GO:0000080 1 9

C0027769 GO:0043523 1 9

C0027769 GO:0043525 1 9

C0027769 GO:0007004 1 9

C0027769 GO:0002263 1 9

C0027769 GO:0005544 1 9

C0027769 GO:0010257 1 9

C0027769 GO:0005546 1 9

C0027769 GO:0001909 1 9

C0027769 GO:0032135 2 9

C0027769 GO:0033032 2 9

C0027769 GO:0051119 1 9

C0027769 GO:0005310 1 9

C0027769 GO:0016209 1 9

C0027769 GO:0004385 1 9

C0027769 GO:0016893 2 9

C0027769 GO:0000790 1 9

C0027769 GO:0016891 3 9

C0027769 GO:0000796 1 9

C0027769 GO:0016895 1 9

C0027769 GO:0051702 1 9

C0027769 GO:0009071 1 9

C0027769 GO:0043236 1 9

C0027769 GO:0090079 1 9

C0027769 GO:0018209 1 9

C0027769 GO:0000018 2 9

C0027769 GO:0016445 1 9

C0027769 GO:0018024 1 9

C0027769 GO:0006297 1 9

C0027769 GO:0002839 2 9

C0027769 GO:0002834 2 9

C0027769 GO:0002837 2 9

C0027769 GO:0002836 2 9

C0027769 GO:0034062 2 9

C0027769 GO:0034061 1 9

C0027769 GO:0050820 1 9

C0027769 GO:0048009 2 9

C0027769 GO:0001764 1 9

C0027769 GO:0046496 1 9

C0027769 GO:0006563 1 9

C0027769 GO:0010149 1 9

C0027769 GO:0032813 1 9

C0027769 GO:0017015 1 9

C0027769 GO:0019319 1 9

C0027769 GO:0050795 1 9

C0027769 GO:0030069 1 9

C0027769 GO:0046148 1 9

C0027769 GO:0005720 1 9

C0027769 GO:0008652 1 9

C0027769 GO:0002711 1 9

C0027769 GO:0042267 2 9

C0027769 GO:0006739 1 9

C0027769 GO:0006733 1 9

C0027769 GO:0016799 1 9

C0027769 GO:0031307 1 9

C0027769 GO:0031306 1 9

C0027769 GO:0007043 1 9

C0027769 GO:0007041 1 9

C0027769 GO:0004529 1 9

C0027769 GO:0016706 1 9

C0027769 GO:0004526 1 9

C0027769 GO:0004521 2 9

C0027769 GO:0015165 1 9

C0027769 GO:0048568 1 9

C0027769 GO:0021954 1 9

C0027769 GO:0021953 1 9

C0027769 GO:0032404 2 9

C0027769 GO:0005355 2 9

C0027769 GO:0016127 1 9

C0027769 GO:0008139 1 9

C0027769 GO:0070301 1 9

C0027769 GO:0031397 1 9

C0027769 GO:0014065 1 9

C0027769 GO:0055067 1 9

C0027769 GO:0043473 1 9

C0027769 GO:0002460 1 9

C0027769 GO:0009084 2 9

C0027769 GO:0016018 2 9

C0027769 GO:0004177 2 9

C0027769 GO:0033135 1 9

C0027769 GO:0030983 2 9

C0027769 GO:0046040 2 9

C0027769 GO:0009109 1 9

C0027769 GO:0051148 1 9

C0027769 GO:0016846 1 9

C0027769 GO:0005814 1 9

C0027769 GO:0033628 1 9

C0027769 GO:0045930 1 9

C0027769 GO:0017119 1 9

C0027769 GO:0010810 1 9

C0027769 GO:0010812 1 9

C0027769 GO:0050690 1 9

C0027769 GO:0016903 1 9

C0027769 GO:0030431 1 9

C0027769 GO:0016909 1 9

C0027769 GO:0009746 1 9

C0027769 GO:0003709 2 9

C0027769 GO:0051881 1 9

C0027769 GO:0032200 1 9

C0027769 GO:0009749 1 9

C0027769 GO:0000387 1 9

C0027769 GO:0006390 1 9

C0027769 GO:0010833 1 9

C0027769 GO:0006479 1 9

C0027769 GO:0032024 1 9

C0027769 GO:0000245 1 9

C0027769 GO:0032387 2 9

C0027769 GO:0021510 1 9

C0027769 GO:0006779 1 9

C0027769 GO:0030509 1 9

C0027769 GO:0006783 1 9

C0027769 GO:0007213 3 9

C0027769 GO:0048512 1 9

C0027769 GO:0006073 1 9

C0027769 GO:0005092 1 9

C0027769 GO:0000096 1 9

C0027769 GO:0006607 1 9

C0027769 GO:0043535 1 9

C0027769 GO:0019902 1 9

C0027769 GO:0007172 1 9

C0027769 GO:0007076 1 9

C0027769 GO:0045793 1 9

C0027769 GO:0002274 1 9

C0027769 GO:0006672 1 9

C0027769 GO:0016278 1 9

C0027769 GO:0016279 1 9

C0027769 GO:0016885 1 9

C0027769 GO:0016289 1 9

C0027769 GO:0005852 3 9

C0027769 GO:0090100 1 9

C0027769 GO:0005851 1 9

C0027769 GO:0016574 1 9

C0027769 GO:0002200 1 9

C0027769 GO:0016471 1 9

C0027769 GO:0004128 1 9

C0027769 GO:0006818 1 9

C0027769 GO:0055029 1 9

C0027769 GO:0006189 2 9

C0027769 GO:0006188 2 9

C0027769 GO:0034655 1 9

C0027769 GO:0034656 1 9

C0027769 GO:0001672 1 9

C0027769 GO:0045616 1 9

C0027769 GO:0033176 1 9

C0027769 GO:0045580 1 9

C0027769 GO:0045582 2 9

C0027769 GO:0046677 1 9

C0027769 GO:0030278 1 9

C0027769 GO:0007250 1 9

C0027769 GO:0051087 1 9

C0027769 GO:0046364 1 9

C0027769 GO:0017156 1 9

C0027769 GO:0031330 1 9

C0027769 GO:0031333 1 9

C0027769 GO:0000217 1 9

C0027769 GO:0004860 1 9

C0027769 GO:0001953 1 9

C0027769 GO:0001952 1 9

C0027769 GO:0016653 2 9

C0027769 GO:0002039 1 9

C0027769 GO:0042054 1 9

C0027769 GO:0034508 1 9

C0027769 GO:0048754 1 9

C0027769 GO:0070822 1 9

C0027769 GO:0032981 1 9

C0027769 GO:0045766 1 9

C0027769 GO:0045604 1 9

C0027769 GO:0005114 1 9

C0027769 GO:0005112 1 9

C0027769 GO:0016229 1 9

C0027769 GO:0034614 1 9

C0027769 GO:0006911 1 9

C0027769 GO:0000779 1 9

C0027769 GO:0009116 1 9

C0027769 GO:0051646 1 9

C0027769 GO:0000070 1 9

C0027769 GO:0001516 1 9

C0027769 GO:0046457 1 9

C0027769 GO:0046456 1 9

C0027769 GO:0046519 1 9

C0027769 GO:0016504 1 9

C0027769 GO:0016505 1 9

C0027769 GO:0046638 2 9

C0027769 GO:0046637 1 9

C0027769 GO:0050684 1 9

C0027769 GO:0046635 1 9

C0027769 GO:0046634 1 9

C0027769 GO:0046631 1 9

C0027769 GO:0050920 1 9

C0027769 GO:0050921 1 9

C0027769 GO:0051893 1 9

C0027769 GO:0008376 1 9

C0027769 GO:0006983 1 9

C0027769 GO:0043043 1 9

C0027769 GO:0010894 1 9

C0027769 GO:0006760 2 9

C0027769 GO:0006769 1 9

C0027769 GO:0031668 1 9

C0027769 GO:0030165 1 9

C0027769 GO:0000502 1 9

C0027769 GO:0030511 2 9

C0027769 GO:0005062 1 9

C0027769 GO:0051219 1 9

C0027769 GO:0005663 1 9

C0027769 GO:0005662 1 9

C0027769 GO:0006164 1 9

C0027769 GO:0032731 1 9

C0027769 GO:0043034 1 9

C0027769 GO:0030194 1 9

C0027769 GO:0005088 1 9

C0027769 GO:0005086 1 9

C0027769 GO:0042745 1 9

C0027769 GO:0042744 1 9

C0027769 GO:0007064 1 9

C0027769 GO:0031369 1 9

C0027769 GO:0042749 1 9

C0027769 GO:0008408 1 9

C0027769 GO:0000381 2 9

C0027769 GO:0015149 1 9

C0027769 GO:0045939 1 9

C0027769 GO:0019239 3 9

C0027769 GO:0033014 1 9

C0027769 GO:0006684 1 9

C0027769 GO:0015145 1 9

C0027769 GO:0030894 1 9

C0027769 GO:0030890 1 9

C0027769 GO:0009156 2 9

C0027769 GO:0048500 1 9

C0027769 GO:0009152 1 9

C0027769 GO:0008013 1 9

C0027769 GO:0014003 1 9

C0027769 GO:0005844 1 9

C0027769 GO:0000132 1 9

C0027769 GO:0004683 1 9

C0027769 GO:0031647 1 9

C0027769 GO:0045739 1 9

C0027769 GO:0022410 1 9

C0027769 GO:0030307 1 9

C0027769 GO:0002819 1 9

C0027769 GO:0030261 1 9

C0027769 GO:0048024 1 9

C0027769 GO:0009127 2 9

C0027769 GO:0006220 1 9

C0027769 GO:0006221 1 9

C0027769 GO:0042772 1 9

C0027769 GO:0042921 1 9

C0027769 GO:0046784 2 9

C0027769 GO:0046782 1 9

C0027769 GO:0044452 1 9

C0027769 GO:0008385 1 9

C0027769 GO:0022612 1 9

C0027769 GO:0022616 2 9

C0027769 GO:0070688 1 9

C0027769 GO:0015298 2 9

C0027769 GO:0017134 1 9

C0027769 GO:0002833 2 9

C0027769 GO:0043601 1 9

C0027769 GO:0070776 1 9

C0027769 GO:0070775 1 9

C0027769 GO:0003727 1 9

C0027769 GO:0006536 1 9

C0027769 GO:0001763 1 9

C0027769 GO:0043370 1 9

C0027769 GO:0000347 2 9

C0027769 GO:0000346 2 9

C0027769 GO:0045682 1 9

C0027769 GO:0032202 1 9

C0027769 GO:0016725 1 9

C0027769 GO:0008526 1 9

C0027769 GO:0019104 1 9

C0027769 GO:0005883 1 9

C0027769 GO:0007159 1 9

C0027769 GO:0016049 1 9

C0027769 GO:0002444 1 9

C0027769 GO:0002446 1 9

C0027769 GO:0016580 1 9

C0027769 GO:0016581 1 9

C0027769 GO:0001938 2 9

C0027769 GO:0033344 1 9

C0027769 GO:0002250 1 9

C0027769 GO:0004549 1 9

C0027769 GO:0015030 1 9

C0027769 GO:0001937 2 9

C0027769 GO:0001936 2 9

C0018681 GO:0005786 1 25

C0018681 GO:0007598 2 25

C0018681 GO:0050840 1 25

C0018681 GO:0043954 1 25

C0018681 GO:0006906 1 25

C0018681 GO:0033158 2 25

C0018681 GO:0002706 2 25

C0018681 GO:0002703 2 25

C0018681 GO:0034199 1 25

C0018681 GO:0009161 1 25

C0018681 GO:0001508 1 25

C0018681 GO:0005782 2 25

C0018681 GO:0070937 1 25

C0018681 GO:0048261 2 25

C0018681 GO:0016863 1 25

C0018681 GO:0009168 2 25

C0018681 GO:0016860 1 25

C0018681 GO:0070513 1 25

C0018681 GO:0000002 1 25

C0018681 GO:0042267 1 25

C0018681 GO:0005834 2 25

C0018681 GO:0044253 1 25

C0018681 GO:0051495 1 25

C0018681 GO:0060249 1 25

C0018681 GO:0004653 1 25

C0018681 GO:0001964 1 25

C0018681 GO:0018210 1 25

C0018681 GO:0031513 2 25

C0018681 GO:0002366 1 25

C0018681 GO:0004712 1 25

C0018681 GO:0016514 2 25

C0018681 GO:0016101 1 25

C0018681 GO:0032438 1 25

C0018681 GO:0006284 1 25

C0018681 GO:0045851 1 25

C0018681 GO:0006282 1 25

C0018681 GO:0051298 1 25

C0018681 GO:0051294 2 25

C0018681 GO:0002824 1 25

C0018681 GO:0002822 2 25

C0018681 GO:0031109 1 25

C0018681 GO:0006474 1 25

C0018681 GO:0007281 1 25

C0018681 GO:0035270 1 25

C0018681 GO:0048066 1 25

C0018681 GO:0001754 1 25

C0018681 GO:0030296 1 25

C0018681 GO:0000718 2 25

C0018681 GO:0006278 2 25

C0018681 GO:0030509 1 25

C0018681 GO:0006271 1 25

C0018681 GO:0006270 1 25

C0018681 GO:0080010 1 25

C0018681 GO:0016814 2 25

C0018681 GO:0010887 2 25

C0018681 GO:0042698 1 25

C0018681 GO:0010885 1 25

C0018681 GO:0043331 2 25

C0018681 GO:0003995 1 25

C0018681 GO:0006378 1 25

C0018681 GO:0006379 1 25

C0018681 GO:0021537 2 25

C0018681 GO:0019320 1 25

C0018681 GO:0043206 1 25

C0018681 GO:0009895 2 25

C0018681 GO:0031958 1 25

C0018681 GO:0032412 1 25

C0018681 GO:0005080 1 25

C0018681 GO:0048568 2 25

C0018681 GO:0030681 2 25

C0018681 GO:0002700 1 25

C0018681 GO:0000302 2 25

C0018681 GO:0030175 2 25

C0018681 GO:0030174 2 25

C0018681 GO:0030170 1 25

C0018681 GO:0035004 1 25

C0018681 GO:0070934 1 25

C0018681 GO:0005487 2 25

C0018681 GO:0001950 1 25

C0018681 GO:0051225 1 25

C0018681 GO:0008565 1 25

C0018681 GO:0016605 1 25

C0018681 GO:0003206 1 25

C0018681 GO:0015992 2 25

C0018681 GO:0015491 1 25

C0018681 GO:0042551 2 25

C0018681 GO:0006885 2 25

C0018681 GO:0016126 1 25

C0018681 GO:0042255 2 25

C0018681 GO:0003208 1 25

C0018681 GO:0042558 1 25

C0018681 GO:0042559 1 25

C0018681 GO:0070742 3 25

C0018681 GO:0005655 2 25

C0018681 GO:0002685 1 25

C0018681 GO:0006112 1 25

C0018681 GO:0033014 1 25

C0018681 GO:0050792 1 25

C0018681 GO:0043021 3 25

C0018681 GO:0043022 3 25

C0018681 GO:0051879 2 25

C0018681 GO:0042827 1 25

C0018681 GO:0043027 1 25

C0018681 GO:0006303 1 25

C0018681 GO:0019883 1 25

C0018681 GO:0007623 1 25

C0018681 GO:0007622 1 25

C0018681 GO:0033077 1 25

C0018681 GO:0009306 1 25

C0018681 GO:0006625 1 25

C0018681 GO:0006626 2 25

C0018681 GO:0032570 1 25

C0018681 GO:0048709 1 25

C0018681 GO:0009303 2 25

C0018681 GO:0009152 2 25

C0018681 GO:0060606 1 25

C0018681 GO:0009260 3 25

C0018681 GO:0007052 2 25

C0018681 GO:0005778 1 25

C0018681 GO:0009264 1 25

C0018681 GO:0009267 1 25

C0018681 GO:0009266 1 25

C0018681 GO:0019395 1 25

C0018681 GO:0035303 1 25

C0018681 GO:0004843 1 25

C0018681 GO:0044042 1 25

C0018681 GO:0030677 2 25

C0018681 GO:0030675 2 25

C0018681 GO:0031080 2 25

C0018681 GO:0004536 1 25

C0018681 GO:0006000 1 25

C0018681 GO:0015781 1 25

C0018681 GO:0015780 1 25

C0018681 GO:0004532 2 25

C0018681 GO:0016776 1 25

C0018681 GO:0035097 1 25

C0018681 GO:0006691 1 25

C0018681 GO:0015074 2 25

C0018681 GO:0048365 3 25

C0018681 GO:0005005 1 25

C0018681 GO:0008633 1 25

C0018681 GO:0010553 2 25

C0018681 GO:0015175 1 25

C0018681 GO:0015172 1 25

C0018681 GO:0008023 1 25

C0018681 GO:0030880 2 25

C0018681 GO:0008143 2 25

C0018681 GO:0030914 1 25

C0018681 GO:0033209 4 25

C0018681 GO:0046579 1 25

C0018681 GO:0008144 1 25

C0018681 GO:0008641 1 25

C0018681 GO:0031228 2 25

C0018681 GO:0042177 1 25

C0018681 GO:0006309 1 25

C0018681 GO:0010001 1 25

C0018681 GO:0051453 1 25

C0018681 GO:0051452 1 25

C0018681 GO:0004691 2 25

C0018681 GO:0004690 2 25

C0018681 GO:0043241 1 25

C0018681 GO:0050431 2 25

C0018681 GO:0016783 1 25

C0018681 GO:0000127 3 25

C0018681 GO:0007127 2 25

C0018681 GO:0043560 1 25

C0018681 GO:0043489 1 25

C0018681 GO:0043484 1 25

C0018681 GO:0045178 5 25

C0018681 GO:0030705 1 25

C0018681 GO:0007162 2 25

C0018681 GO:0002228 1 25

C0018681 GO:0045741 1 25

C0018681 GO:0045621 1 25

C0018681 GO:0045620 1 25

C0018681 GO:0045622 1 25

C0018681 GO:0002221 1 25

C0018681 GO:0033108 2 25

C0018681 GO:0008287 2 25

C0018681 GO:0006879 1 25

C0018681 GO:0006878 1 25

C0018681 GO:0070776 1 25

C0018681 GO:0002637 1 25

C0018681 GO:0045211 1 25

C0018681 GO:0005913 1 25

C0018681 GO:0018108 1 25

C0018681 GO:0001656 3 25

C0018681 GO:0046504 2 25

C0018681 GO:0046889 2 25

C0018681 GO:0006978 2 25

C0018681 GO:0034329 1 25

C0018681 GO:0051983 1 25

C0018681 GO:0035267 1 25

C0018681 GO:0032728 1 25

C0018681 GO:0016854 1 25

C0018681 GO:0008535 1 25

C0018681 GO:0046875 1 25

C0018681 GO:0032369 1 25

C0018681 GO:0006749 1 25

C0018681 GO:0004190 1 25

C0018681 GO:0030262 1 25

C0018681 GO:0003899 1 25

C0018681 GO:0042797 3 25

C0018681 GO:0016254 1 25

C0018681 GO:0042791 3 25

C0018681 GO:0017046 2 25

C0018681 GO:0045923 1 25

C0018681 GO:0007416 1 25

C0018681 GO:0019047 2 25

C0018681 GO:0017124 3 25

C0018681 GO:0017048 1 25

C0018681 GO:0060021 1 25

C0018681 GO:0006099 1 25

C0018681 GO:0004601 1 25

C0018681 GO:0001889 1 25

C0018681 GO:0042594 1 25

C0018681 GO:0045445 1 25

C0018681 GO:0043631 1 25

C0018681 GO:0030425 1 25

C0018681 GO:0008603 2 25

C0018681 GO:0006096 1 25

C0018681 GO:0022408 1 25

C0018681 GO:0022409 1 25

C0018681 GO:0009820 1 25

C0018681 GO:0033993 3 25

C0018681 GO:0043189 1 25

C0018681 GO:0010828 1 25

C0018681 GO:0032271 1 25

C0018681 GO:0045954 1 25

C0018681 GO:0003730 2 25

C0018681 GO:0030424 1 25

C0018681 GO:0045814 3 25

C0018681 GO:0005577 1 25

C0018681 GO:0019212 1 25

C0018681 GO:0045089 4 25

C0018681 GO:0045088 1 25

C0018681 GO:0070120 2 25

C0018681 GO:0051291 1 25

C0018681 GO:0048592 1 25

C0018681 GO:0048593 3 25

C0018681 GO:0009223 1 25

C0018681 GO:0015711 2 25

C0018681 GO:0000245 1 25

C0018681 GO:0031047 2 25

C0018681 GO:0000445 3 25

C0018681 GO:0006518 1 25

C0018681 GO:0050750 2 25

C0018681 GO:0001838 1 25

C0018681 GO:0005048 1 25

C0018681 GO:0040017 2 25

C0018681 GO:0030532 2 25

C0018681 GO:0035194 1 25

C0018681 GO:0035195 1 25

C0018681 GO:0008517 3 25

C0018681 GO:0043535 1 25

C0018681 GO:0001837 2 25

C0018681 GO:0008186 1 25

C0018681 GO:0005763 4 25

C0018681 GO:0048524 1 25

C0018681 GO:0051806 1 25

C0018681 GO:0006413 2 25

C0018681 GO:0006144 2 25

C0018681 GO:0019362 3 25

C0018681 GO:0034440 1 25

C0018681 GO:0030122 1 25

C0018681 GO:0042133 1 25

C0018681 GO:0000086 1 25

C0018681 GO:0031272 1 25

C0018681 GO:0007612 1 25

C0018681 GO:0000080 1 25

C0018681 GO:0031638 1 25

C0018681 GO:0043449 1 25

C0018681 GO:0043523 1 25

C0018681 GO:0003015 2 25

C0018681 GO:0031593 2 25

C0018681 GO:0031343 1 25

C0018681 GO:0090004 1 25

C0018681 GO:0016597 1 25

C0018681 GO:0007004 2 25

C0018681 GO:0016180 1 25

C0018681 GO:0042446 1 25

C0018681 GO:0005540 2 25

C0018681 GO:0002263 1 25

C0018681 GO:0005545 3 25

C0018681 GO:0045667 2 25

C0018681 GO:0001909 1 25

C0018681 GO:0070531 1 25

C0018681 GO:0032135 5 25

C0018681 GO:0045669 1 25

C0018681 GO:0033032 2 25

C0018681 GO:0051057 1 25

C0018681 GO:0009167 1 25

C0018681 GO:0051119 1 25

C0018681 GO:0043968 1 25

C0018681 GO:0005310 1 25

C0018681 GO:0016209 1 25

C0018681 GO:0004385 1 25

C0018681 GO:0051059 1 25

C0018681 GO:0016893 3 25

C0018681 GO:0008170 5 25

C0018681 GO:0016891 3 25

C0018681 GO:0016896 2 25

C0018681 GO:0002819 1 25

C0018681 GO:0000794 1 25

C0018681 GO:0006536 1 25

C0018681 GO:0009074 1 25

C0018681 GO:0051702 1 25

C0018681 GO:0070279 1 25

C0018681 GO:0042992 2 25

C0018681 GO:0006941 2 25

C0018681 GO:0031668 1 25

C0018681 GO:0018209 1 25

C0018681 GO:0000159 2 25

C0018681 GO:0034284 2 25

C0018681 GO:0000018 1 25

C0018681 GO:0005112 1 25

C0018681 GO:0046530 1 25

C0018681 GO:0004707 1 25

C0018681 GO:0016565 2 25

C0018681 GO:0016291 3 25

C0018681 GO:0016441 1 25

C0018681 GO:0004859 1 25

C0018681 GO:0004709 1 25

C0018681 GO:0016444 1 25

C0018681 GO:0046718 1 25

C0018681 GO:0018024 2 25

C0018681 GO:0006297 2 25

C0018681 GO:0017166 1 25

C0018681 GO:0005732 2 25

C0018681 GO:0015851 2 25

C0018681 GO:0006298 6 25

C0018681 GO:0046716 1 25

C0018681 GO:0015858 3 25

C0018681 GO:0050821 1 25

C0018681 GO:0050820 2 25

C0018681 GO:0048009 1 25

C0018681 GO:0001764 1 25

C0018681 GO:0046496 3 25

C0018681 GO:0005929 4 25

C0018681 GO:0051028 1 25

C0018681 GO:0033280 2 25

C0018681 GO:0034708 1 25

C0018681 GO:0046427 1 25

C0018681 GO:0051181 3 25

C0018681 GO:0051180 4 25

C0018681 GO:0051183 3 25

C0018681 GO:0009126 1 25

C0018681 GO:0042098 1 25

C0018681 GO:0015114 1 25

C0018681 GO:0009124 1 25

C0018681 GO:0007183 1 25

C0018681 GO:0016363 2 25

C0018681 GO:0006220 3 25

C0018681 GO:0031941 1 25

C0018681 GO:0032813 1 25

C0018681 GO:0009881 1 25

C0018681 GO:0006221 2 25

C0018681 GO:0000339 2 25

C0018681 GO:0032769 2 25

C0018681 GO:0017015 2 25

C0018681 GO:0019319 3 25

C0018681 GO:0050795 1 25

C0018681 GO:0006801 1 25

C0018681 GO:0008494 1 25

C0018681 GO:0051238 1 25

C0018681 GO:0008499 1 25

C0018681 GO:0003231 1 25

C0018681 GO:0030069 2 25

C0018681 GO:0000272 2 25

C0018681 GO:0016944 1 25

C0018681 GO:0008652 1 25

C0018681 GO:0007220 1 25

C0018681 GO:0009953 1 25

C0018681 GO:0032368 1 25

C0018681 GO:0042542 3 25

C0018681 GO:0034382 1 25

C0018681 GO:0022898 2 25

C0018681 GO:0010714 1 25

C0018681 GO:0010712 1 25

C0018681 GO:0034384 1 25

C0018681 GO:0070198 3 25

C0018681 GO:0043154 2 25

C0018681 GO:0046824 2 25

C0018681 GO:0046823 2 25

C0018681 GO:0043014 1 25

C0018681 GO:0003746 2 25

C0018681 GO:0006584 1 25

C0018681 GO:0043010 1 25

C0018681 GO:0005066 1 25

C0018681 GO:0016796 3 25

C0018681 GO:0016790 2 25

C0018681 GO:0006635 1 25

C0018681 GO:0048306 1 25

C0018681 GO:0048469 1 25

C0018681 GO:0006733 2 25

C0018681 GO:0016799 3 25

C0018681 GO:0004520 1 25

C0018681 GO:0043407 1 25

C0018681 GO:0042401 1 25

C0018681 GO:0032967 1 25

C0018681 GO:0007043 1 25

C0018681 GO:0007041 1 25

C0018681 GO:0000428 2 25

C0018681 GO:0043409 1 25

C0018681 GO:0046006 1 25

C0018681 GO:0016706 1 25

C0018681 GO:0004527 3 25

C0018681 GO:0004526 2 25

C0018681 GO:0004521 1 25

C0018681 GO:0001945 2 25

C0018681 GO:0050768 2 25

C0018681 GO:0019674 1 25

C0018681 GO:0016667 2 25

C0018681 GO:0009925 1 25

C0018681 GO:0021954 3 25

C0018681 GO:0016877 2 25

C0018681 GO:0032407 1 25

C0018681 GO:0021953 3 25

C0018681 GO:0032404 6 25

C0018681 GO:0005355 1 25

C0018681 GO:0042169 1 25

C0018681 GO:0015165 2 25

C0018681 GO:0042162 1 25

C0018681 GO:0070301 2 25

C0018681 GO:0018342 1 25

C0018681 GO:0043270 1 25

C0018681 GO:0031397 2 25

C0018681 GO:0014065 2 25

C0018681 GO:0009408 1 25

C0018681 GO:0007131 2 25

C0018681 GO:0043574 2 25

C0018681 GO:0043473 4 25

C0018681 GO:0007031 2 25

C0018681 GO:0002460 1 25

C0018681 GO:0001786 2 25

C0018681 GO:0000299 2 25

C0018681 GO:0005513 1 25

C0018681 GO:0032202 2 25

C0018681 GO:0032405 2 25

C0018681 GO:0034405 1 25

C0018681 GO:0009084 2 25

C0018681 GO:0016018 2 25

C0018681 GO:0009081 1 25

C0018681 GO:0009083 1 25

C0018681 GO:0004177 1 25

C0018681 GO:0016331 2 25

C0018681 GO:0008484 1 25

C0018681 GO:0055010 1 25

C0018681 GO:0046365 1 25

C0018681 GO:0046364 2 25

C0018681 GO:0030983 5 25

C0018681 GO:0046040 2 25

C0018681 GO:0009109 2 25

C0018681 GO:0051147 1 25

C0018681 GO:0034235 1 25

C0018681 GO:0051148 1 25

C0018681 GO:0050996 1 25

C0018681 GO:0001523 1 25

C0018681 GO:0016846 2 25

C0018681 GO:0016840 2 25

C0018681 GO:0004181 1 25

C0018681 GO:0004180 2 25

C0018681 GO:0000060 2 25

C0018681 GO:0032320 1 25

C0018681 GO:0005814 1 25

C0018681 GO:0004675 1 25

C0018681 GO:0042346 1 25

C0018681 GO:0016246 1 25

C0018681 GO:0018279 1 25

C0018681 GO:0003205 1 25

C0018681 GO:0005217 1 25

C0018681 GO:0050699 1 25

C0018681 GO:0045939 1 25

C0018681 GO:0031124 2 25

C0018681 GO:0010812 2 25

C0018681 GO:0031123 2 25

C0018681 GO:0022417 1 25

C0018681 GO:0016903 2 25

C0018681 GO:0030431 1 25

C0018681 GO:0031572 2 25

C0018681 GO:0043624 1 25

C0018681 GO:0045116 1 25

C0018681 GO:0009746 2 25

C0018681 GO:0003709 3 25

C0018681 GO:0006493 1 25

C0018681 GO:0009743 1 25

C0018681 GO:0050690 1 25

C0018681 GO:0002793 1 25

C0018681 GO:0051923 1 25

C0018681 GO:0032200 1 25

C0018681 GO:0009749 2 25

C0018681 GO:0044409 1 25

C0018681 GO:0070652 1 25

C0018681 GO:0000387 2 25

C0018681 GO:0006390 2 25

C0018681 GO:0045806 1 25

C0018681 GO:0032496 1 25

C0018681 GO:0016281 1 25

C0018681 GO:0008209 1 25

C0018681 GO:0006776 1 25

C0018681 GO:0032655 1 25

C0018681 GO:0006775 1 25

C0018681 GO:0032388 2 25

C0018681 GO:0032925 1 25

C0018681 GO:0016864 1 25

C0018681 GO:0009218 1 25

C0018681 GO:0009219 1 25

C0018681 GO:0031970 1 25

C0018681 GO:0032387 3 25

C0018681 GO:0019228 1 25

C0018681 GO:0021510 2 25

C0018681 GO:0005788 2 25

C0018681 GO:0006779 1 25

C0018681 GO:0031050 2 25

C0018681 GO:0030111 1 25

C0018681 GO:0015645 1 25

C0018681 GO:0016411 1 25

C0018681 GO:0030119 1 25

C0018681 GO:0030118 1 25

C0018681 GO:0046131 1 25

C0018681 GO:0005779 1 25

C0018681 GO:0007219 1 25

C0018681 GO:0002687 2 25

C0018681 GO:0006783 1 25

C0018681 GO:0007213 1 25

C0018681 GO:0016627 1 25

C0018681 GO:0001824 1 25

C0018681 GO:0007216 1 25

C0018681 GO:0016620 1 25

C0018681 GO:0070001 1 25

C0018681 GO:0048512 1 25

C0018681 GO:0005657 1 25

C0018681 GO:0006073 1 25

C0018681 GO:0010721 2 25

C0018681 GO:0031907 2 25

C0018681 GO:0005678 2 25

C0018681 GO:0031903 1 25

C0018681 GO:0034404 1 25

C0018681 GO:0015718 2 25

C0018681 GO:0032153 1 25

C0018681 GO:0032155 1 25

C0018681 GO:0006607 2 25

C0018681 GO:0008239 1 25

C0018681 GO:0005095 3 25

C0018681 GO:0003007 1 25

C0018681 GO:0031274 1 25

C0018681 GO:0019903 1 25

C0018681 GO:0045187 1 25

C0018681 GO:0007172 1 25

C0018681 GO:0043531 1 25

C0018681 GO:0007044 1 25

C0018681 GO:0007076 1 25

C0018681 GO:0007568 1 25

C0018681 GO:0009451 1 25

C0018681 GO:0005885 1 25

C0018681 GO:0055117 2 25

C0018681 GO:0008630 1 25

C0018681 GO:0052192 1 25

C0018681 GO:0032479 3 25

C0018681 GO:0043603 1 25

C0018681 GO:0043025 1 25

C0018681 GO:0050732 1 25

C0018681 GO:0060390 2 25

C0018681 GO:0060391 2 25

C0018681 GO:0005527 2 25

C0018681 GO:0033135 1 25

C0018681 GO:0001912 2 25

C0018681 GO:0002224 1 25

C0018681 GO:0055072 1 25

C0018681 GO:0004550 1 25

C0018681 GO:0051972 1 25

C0018681 GO:0006672 2 25

C0018681 GO:0045494 1 25

C0018681 GO:0043525 1 25

C0018681 GO:0022602 1 25

C0018681 GO:0042398 1 25

C0018681 GO:0008272 1 25

C0018681 GO:0016278 2 25

C0018681 GO:0016279 2 25

C0018681 GO:0009062 1 25

C0018681 GO:0000783 1 25

C0018681 GO:0000782 1 25

C0018681 GO:0016885 1 25

C0018681 GO:0000784 1 25

C0018681 GO:0009065 1 25

C0018681 GO:0009712 1 25

C0018681 GO:0070918 2 25

C0018681 GO:0031576 2 25

C0018681 GO:0016289 3 25

C0018681 GO:0005852 4 25

C0018681 GO:0043547 1 25

C0018681 GO:0090100 1 25

C0018681 GO:0005851 3 25

C0018681 GO:0043548 1 25

C0018681 GO:0070717 1 25

C0018681 GO:0045055 2 25

C0018681 GO:0051896 1 25

C0018681 GO:0016574 1 25

C0018681 GO:0005528 2 25

C0018681 GO:0031264 1 25

C0018681 GO:0031256 1 25

C0018681 GO:0005522 2 25

C0018681 GO:0048641 1 25

C0018681 GO:0016471 2 25

C0018681 GO:0006818 2 25

C0018681 GO:0055029 2 25

C0018681 GO:0016303 1 25

C0018681 GO:0035251 1 25

C0018681 GO:0035250 1 25

C0018681 GO:0030374 2 25

C0018681 GO:0001776 1 25

C0018681 GO:0006189 2 25

C0018681 GO:0006188 2 25

C0018681 GO:0042308 2 25

C0018681 GO:0034654 1 25

C0018681 GO:0042306 1 25

C0018681 GO:0045616 1 25

C0018681 GO:0042301 1 25

C0018681 GO:0044275 1 25

C0018681 GO:0001570 1 25

C0018681 GO:0007250 1 25

C0018681 GO:0008091 1 25

C0018681 GO:0006953 1 25

C0018681 GO:0005104 1 25

C0018681 GO:0002758 1 25

C0018681 GO:0016831 2 25

C0018681 GO:0006213 1 25

C0018681 GO:0003684 1 25

C0018681 GO:0009262 1 25

C0018681 GO:0033363 1 25

C0018681 GO:0050671 1 25

C0018681 GO:0006739 2 25

C0018681 GO:0030276 2 25

C0018681 GO:0006359 1 25

C0018681 GO:0033176 2 25

C0018681 GO:0007369 1 25

C0018681 GO:0030159 2 25

C0018681 GO:0010921 1 25

C0018681 GO:0006477 1 25

C0018681 GO:0030275 1 25

C0018681 GO:0010257 2 25

C0018681 GO:0045580 3 25

C0018681 GO:0045582 2 25

C0018681 GO:0060047 2 25

C0018681 GO:0046677 2 25

C0018681 GO:0031231 1 25

C0018681 GO:0003229 1 25

C0018681 GO:0001953 2 25

C0018681 GO:0060048 1 25

C0018681 GO:0030530 1 25

C0018681 GO:0050868 1 25

C0018681 GO:0034399 2 25

C0018681 GO:0051324 2 25

C0018681 GO:0015278 1 25

C0018681 GO:0019783 1 25

C0018681 GO:0001707 2 25

C0018681 GO:0001704 2 25

C0018681 GO:0032376 1 25

C0018681 GO:0034311 1 25

C0018681 GO:0032370 2 25

C0018681 GO:0032373 1 25

C0018681 GO:0042375 1 25

C0018681 GO:0015370 1 25

C0018681 GO:0006505 1 25

C0018681 GO:0070761 2 25

C0018681 GO:0032272 1 25

C0018681 GO:0006506 1 25

C0018681 GO:0017156 2 25

C0018681 GO:0017157 1 25

C0018681 GO:0048332 2 25

C0018681 GO:0031330 2 25

C0018681 GO:0032508 1 25

C0018681 GO:0031333 2 25

C0018681 GO:0000217 1 25

C0018681 GO:0004860 1 25

C0018681 GO:0030128 1 25

C0018681 GO:0016073 1 25

C0018681 GO:0004864 1 25

C0018681 GO:0005593 1 25

C0018681 GO:0004869 1 25

C0018681 GO:0030121 1 25

C0018681 GO:0030125 1 25

C0018681 GO:0051258 1 25

C0018681 GO:0048839 1 25

C0018681 GO:0050770 1 25

C0018681 GO:0001959 1 25

C0018681 GO:0015884 1 25

C0018681 GO:0006268 1 25

C0018681 GO:0046906 1 25

C0018681 GO:0032182 2 25

C0018681 GO:0001952 1 25

C0018681 GO:0005024 1 25

C0018681 GO:0051828 1 25

C0018681 GO:0005744 2 25

C0018681 GO:0016653 1 25

C0018681 GO:0006007 1 25

C0018681 GO:0002039 1 25

C0018681 GO:0005720 1 25

C0018681 GO:0034502 3 25

C0018681 GO:0042116 2 25

C0018681 GO:0048753 1 25

C0018681 GO:0005161 1 25

C0018681 GO:0034508 2 25

C0018681 GO:0042113 1 25

C0018681 GO:0000731 1 25

C0018681 GO:0042417 1 25

C0018681 GO:0046112 1 25

C0018681 GO:0005891 1 25

C0018681 GO:0005436 1 25

C0018681 GO:0043506 1 25

C0018681 GO:0045768 1 25

C0018681 GO:0070822 1 25

C0018681 GO:0032984 1 25

C0018681 GO:0043467 1 25

C0018681 GO:0032981 2 25

C0018681 GO:0005003 2 25

C0018681 GO:0045766 2 25

C0018681 GO:0045767 1 25

C0018681 GO:0033059 1 25

C0018681 GO:0004467 1 25

C0018681 GO:0008235 1 25

C0018681 GO:0045604 1 25

C0018681 GO:0006695 1 25

C0018681 GO:0005114 1 25

C0018681 GO:0005313 1 25

C0018681 GO:0030165 1 25

C0018681 GO:0016229 1 25

C0018681 GO:0005007 2 25

C0018681 GO:0046356 1 25

C0018681 GO:0007584 1 25

C0018681 GO:0030235 1 25

C0018681 GO:0034341 1 25

C0018681 GO:0000779 4 25

C0018681 GO:0009110 1 25

C0018681 GO:0009116 2 25

C0018681 GO:0002717 1 25

C0018681 GO:0051646 1 25

C0018681 GO:0070925 2 25

C0018681 GO:0001530 1 25

C0018681 GO:0001533 1 25

C0018681 GO:0000777 3 25

C0018681 GO:0031529 1 25

C0018681 GO:0000178 2 25

C0018681 GO:0000070 1 25

C0018681 GO:0001516 1 25

C0018681 GO:0000790 1 25

C0018681 GO:0019842 1 25

C0018681 GO:0000175 2 25

C0018681 GO:0046457 1 25

C0018681 GO:0046456 1 25

C0018681 GO:0043596 2 25

C0018681 GO:0030900 1 25

C0018681 GO:0046519 2 25

C0018681 GO:0016504 1 25

C0018681 GO:0016505 1 25

C0018681 GO:0048520 1 25

C0018681 GO:0030856 1 25

C0018681 GO:0008589 1 25

C0018681 GO:0031114 2 25

C0018681 GO:0016894 1 25

C0018681 GO:0046638 2 25

C0018681 GO:0046637 1 25

C0018681 GO:0051287 1 25

C0018681 GO:0046635 1 25

C0018681 GO:0046634 1 25

C0018681 GO:0046631 2 25

C0018681 GO:0008584 1 25

C0018681 GO:0055070 1 25

C0018681 GO:0050926 1 25

C0018681 GO:0008276 1 25

C0018681 GO:0050920 2 25

C0018681 GO:0016645 1 25

C0018681 GO:0045309 1 25

C0018681 GO:0051893 1 25

C0018681 GO:0008375 2 25

C0018681 GO:0008376 1 25

C0018681 GO:0051897 1 25

C0018681 GO:0008278 2 25

C0018681 GO:0034762 1 25

C0018681 GO:0008373 1 25

C0018681 GO:0033628 1 25

C0018681 GO:0031985 1 25

C0018681 GO:0031984 1 25

C0018681 GO:0006983 2 25

C0018681 GO:0018196 1 25

C0018681 GO:0015238 1 25

C0018681 GO:0000796 1 25

C0018681 GO:0006266 2 25

C0018681 GO:0010894 1 25

C0018681 GO:0010896 2 25

C0018681 GO:0010898 1 25

C0018681 GO:0004089 1 25

C0018681 GO:0006383 1 25

C0018681 GO:0032393 1 25

C0018681 GO:0032392 1 25

C0018681 GO:0005791 1 25

C0018681 GO:0043648 1 25

C0018681 GO:0000314 4 25

C0018681 GO:0006769 3 25

C0018681 GO:0006563 3 25

C0018681 GO:0010975 1 25

C0018681 GO:0000502 1 25

C0018681 GO:0030511 2 25

C0018681 GO:0045109 1 25

C0018681 GO:0070584 1 25

C0018681 GO:0070585 2 25

C0018681 GO:0051216 1 25

C0018681 GO:0051354 3 25

C0018681 GO:0051219 1 25

C0018681 GO:0017069 1 25

C0018681 GO:0005663 3 25

C0018681 GO:0043130 2 25

C0018681 GO:0005665 1 25

C0018681 GO:0006911 1 25

C0018681 GO:0048500 1 25

C0018681 GO:0005669 1 25

C0018681 GO:0019438 1 25

C0018681 GO:0030194 2 25

C0018681 GO:0005089 1 25

C0018681 GO:0021782 1 25

C0018681 GO:0006611 1 25

C0018681 GO:0007205 1 25

C0018681 GO:0002821 1 25

C0018681 GO:0032648 1 25

C0018681 GO:0005086 1 25

C0018681 GO:0042745 1 25

C0018681 GO:0042744 1 25

C0018681 GO:0042743 1 25

C0018681 GO:0032947 3 25

C0018681 GO:0005871 2 25

C0018681 GO:0043425 1 25

C0018681 GO:0007064 1 25

C0018681 GO:0055067 2 25

C0018681 GO:0014020 1 25

C0018681 GO:0042749 1 25

C0018681 GO:0004549 2 25

C0018681 GO:0016049 1 25

C0018681 GO:0051457 4 25

C0018681 GO:0035085 2 25

C0018681 GO:0015149 1 25

C0018681 GO:0010810 1 25

C0018681 GO:0019239 4 25

C0018681 GO:0005662 2 25

C0018681 GO:0006687 1 25

C0018681 GO:0016763 2 25

C0018681 GO:0001841 1 25

C0018681 GO:0001843 1 25

C0018681 GO:0016684 1 25

C0018681 GO:0015145 1 25

C0018681 GO:0043168 1 25

C0018681 GO:0030894 4 25

C0018681 GO:0042384 1 25

C0018681 GO:0005337 3 25

C0018681 GO:0009394 1 25

C0018681 GO:0009156 3 25

C0018681 GO:0006164 2 25

C0018681 GO:0033764 1 25

C0018681 GO:0008013 1 25

C0018681 GO:0019104 4 25

C0018681 GO:0007026 2 25

C0018681 GO:0001701 2 25

C0018681 GO:0048256 2 25

C0018681 GO:0048255 1 25

C0018681 GO:0000038 1 25

C0018681 GO:0014003 1 25

C0018681 GO:0000030 3 25

C0018681 GO:0043550 2 25

C0018681 GO:0000132 2 25

C0018681 GO:0003756 1 25

C0018681 GO:0042734 1 25

C0018681 GO:0004683 1 25

C0018681 GO:0004726 1 25

C0018681 GO:0031647 3 25

C0018681 GO:0030867 1 25

C0018681 GO:0031579 1 25

C0018681 GO:0043496 1 25

C0018681 GO:0004708 1 25

C0018681 GO:0002218 1 25

C0018681 GO:0045736 2 25

C0018681 GO:0032481 1 25

C0018681 GO:0005246 1 25

C0018681 GO:0045739 3 25

C0018681 GO:0005537 1 25

C0018681 GO:0022410 1 25

C0018681 GO:0048863 1 25

C0018681 GO:0051087 2 25

C0018681 GO:0016607 2 25

C0018681 GO:0043256 1 25

C0018681 GO:0035148 1 25

C0018681 GO:0030261 1 25

C0018681 GO:0030260 1 25

C0018681 GO:0006942 1 25

C0018681 GO:0048029 1 25

C0018681 GO:0007266 1 25

C0018681 GO:0052126 1 25

C0018681 GO:0009123 1 25

C0018681 GO:0060263 1 25

C0018681 GO:0007259 1 25

C0018681 GO:0043087 1 25

C0018681 GO:0009127 2 25

C0018681 GO:0044246 1 25

C0018681 GO:0000723 1 25

C0018681 GO:0010883 1 25

C0018681 GO:0000049 1 25

C0018681 GO:0070603 2 25

C0018681 GO:0007569 2 25

C0018681 GO:0002444 2 25

C0018681 GO:0042531 1 25

C0018681 GO:0042921 1 25

C0018681 GO:0046784 3 25

C0018681 GO:0046782 1 25

C0018681 GO:0044452 3 25

C0018681 GO:0008385 1 25

C0018681 GO:0045742 1 25

C0018681 GO:0022616 1 25

C0018681 GO:0033160 2 25

C0018681 GO:0015295 1 25

C0018681 GO:0002718 1 25

C0018681 GO:0010939 2 25

C0018681 GO:0043028 1 25

C0018681 GO:0046165 2 25

C0018681 GO:0070688 3 25

C0018681 GO:0015298 1 25

C0018681 GO:0035035 1 25

C0018681 GO:0034062 1 25

C0018681 GO:0015804 1 25

C0018681 GO:0030004 1 25

C0018681 GO:0015807 1 25

C0018681 GO:0010832 1 25

C0018681 GO:0010833 2 25

C0018681 GO:0010830 1 25

C0018681 GO:0018958 1 25

C0018681 GO:0008250 1 25

C0018681 GO:0055085 2 25

C0018681 GO:0045598 1 25

C0018681 GO:0006084 1 25

C0018681 GO:0051092 3 25

C0018681 GO:0043601 4 25

C0018681 GO:0051096 4 25

C0018681 GO:0051095 2 25

C0018681 GO:0042364 2 25

C0018681 GO:0070775 1 25

C0018681 GO:0016909 2 25

C0018681 GO:0042813 1 25

C0018681 GO:0003727 1 25

C0018681 GO:0003724 1 25

C0018681 GO:0019059 1 25

C0018681 GO:0043370 1 25

C0018681 GO:0009304 3 25

C0018681 GO:0034976 1 25

C0018681 GO:0000185 1 25

C0018681 GO:0008625 1 25

C0018681 GO:0046326 1 25

C0018681 GO:0019751 1 25

C0018681 GO:0003705 1 25

C0018681 GO:0000347 3 25

C0018681 GO:0000346 3 25

C0018681 GO:0032869 1 25

C0018681 GO:0016328 1 25

C0018681 GO:0030132 1 25

C0018681 GO:0031369 1 25

C0018681 GO:0030641 1 25

C0018681 GO:0050661 1 25

C0018681 GO:0016725 2 25

C0018681 GO:0034381 1 25

C0018681 GO:0046902 1 25

C0018681 GO:0000940 1 25

C0018681 GO:0042516 1 25

C0018681 GO:0016646 1 25

C0018681 GO:0051262 2 25

C0018681 GO:0008526 1 25

C0018681 GO:0050927 1 25

C0018681 GO:0006721 1 25

C0018681 GO:0070412 1 25

C0018681 GO:0048488 1 25

C0018681 GO:0006406 1 25

C0018681 GO:0048531 1 25

C0018681 GO:0042552 1 25

C0018681 GO:0034451 2 25

C0018681 GO:0070325 2 25

C0018681 GO:0048742 1 25

C0018681 GO:0008408 3 25

C0018681 GO:0042102 1 25

C0018681 GO:0005883 1 25

C0018681 GO:0007159 1 25

C0018681 GO:0005884 1 25

C0018681 GO:0030898 1 25

C0018681 GO:0002440 1 25

C0018681 GO:0000381 2 25

C0018681 GO:0007157 3 25

C0018681 GO:0002443 1 25

C0018681 GO:0042772 2 25

C0018681 GO:0002446 2 25

C0018681 GO:0042771 2 25

C0018681 GO:0042063 1 25

C0018681 GO:0002562 1 25

C0018681 GO:0004579 1 25

C0018681 GO:0016580 1 25

C0018681 GO:0016581 2 25

C0018681 GO:0050921 2 25

C0018681 GO:0045008 1 25

C0018681 GO:0001938 1 25

C0018681 GO:0034220 2 25

C0018681 GO:0033344 2 25

C0018681 GO:0002250 1 25

C0018681 GO:0042104 1 25

C0018681 GO:0030228 1 25

C0018681 GO:0001933 1 25

C0018681 GO:0001936 4 25

C0018681 GO:0001937 3 25

C0018681 GO:0015036 2 25

C0002871 GO:0090087 1 10

C0002871 GO:0002706 1 10

C0002871 GO:0002703 2 10

C0002871 GO:0034199 1 10

C0002871 GO:0016864 1 10

C0002871 GO:0016862 1 10

C0002871 GO:0009168 1 10

C0002871 GO:0016860 2 10

C0002871 GO:0000002 2 10

C0002871 GO:0043206 1 10

C0002871 GO:0005834 1 10

C0002871 GO:0006978 1 10

C0002871 GO:0004653 1 10

C0002871 GO:0006309 1 10

C0002871 GO:0007080 1 10

C0002871 GO:0030856 1 10

C0002871 GO:0008484 1 10

C0002871 GO:0016514 1 10

C0002871 GO:0051294 2 10

C0002871 GO:0003923 1 10

C0002871 GO:0002822 2 10

C0002871 GO:0031109 2 10

C0002871 GO:0021510 1 10

C0002871 GO:0035270 1 10

C0002871 GO:0002673 1 10

C0002871 GO:0006278 1 10

C0002871 GO:0045259 1 10

C0002871 GO:0016814 1 10

C0002871 GO:0010887 1 10

C0002871 GO:0010884 1 10

C0002871 GO:0043331 1 10

C0002871 GO:0003995 1 10

C0002871 GO:0006378 1 10

C0002871 GO:0010888 1 10

C0002871 GO:0060260 1 10

C0002871 GO:0031958 1 10

C0002871 GO:0032412 1 10

C0002871 GO:0048568 1 10

C0002871 GO:0000302 2 10

C0002871 GO:0008483 1 10

C0002871 GO:0035004 1 10

C0002871 GO:0005487 2 10

C0002871 GO:0003205 1 10

C0002871 GO:0003206 1 10

C0002871 GO:0042552 1 10

C0002871 GO:0015491 1 10

C0002871 GO:0042551 1 10

C0002871 GO:0042255 1 10

C0002871 GO:0003208 1 10

C0002871 GO:0042559 1 10

C0002871 GO:0002687 1 10

C0002871 GO:0002685 1 10

C0002871 GO:0051653 1 10

C0002871 GO:0043021 2 10

C0002871 GO:0043022 2 10

C0002871 GO:0043027 1 10

C0002871 GO:0048709 1 10

C0002871 GO:0033764 1 10

C0002871 GO:0009260 1 10

C0002871 GO:0009262 2 10

C0002871 GO:0009264 2 10

C0002871 GO:0009266 1 10

C0002871 GO:0044042 1 10

C0002871 GO:0031080 1 10

C0002871 GO:0004536 1 10

C0002871 GO:0050650 1 10

C0002871 GO:0004532 1 10

C0002871 GO:0050654 1 10

C0002871 GO:0070566 1 10

C0002871 GO:0048365 2 10

C0002871 GO:0016676 2 10

C0002871 GO:0016675 2 10

C0002871 GO:0006026 1 10

C0002871 GO:0006027 1 10

C0002871 GO:0030880 1 10

C0002871 GO:0033613 1 10

C0002871 GO:0050690 1 10

C0002871 GO:0014003 1 10

C0002871 GO:0042177 1 10

C0002871 GO:0031498 1 10

C0002871 GO:0042645 1 10

C0002871 GO:0005876 1 10

C0002871 GO:0009395 1 10

C0002871 GO:0009394 2 10

C0002871 GO:0033683 1 10

C0002871 GO:0002228 2 10

C0002871 GO:0045741 1 10

C0002871 GO:0045621 1 10

C0002871 GO:0005506 1 10

C0002871 GO:0045622 1 10

C0002871 GO:0033108 2 10

C0002871 GO:0055002 1 10

C0002871 GO:0006073 1 10

C0002871 GO:0042288 1 10

C0002871 GO:0050879 1 10

C0002871 GO:0055008 1 10

C0002871 GO:0051015 1 10

C0002871 GO:0005913 1 10

C0002871 GO:0001656 1 10

C0002871 GO:0046889 1 10

C0002871 GO:0033293 1 10

C0002871 GO:0044253 1 10

C0002871 GO:0006337 1 10

C0002871 GO:0042542 1 10

C0002871 GO:0022404 1 10

C0002871 GO:0016528 1 10

C0002871 GO:0016529 1 10

C0002871 GO:0003899 2 10

C0002871 GO:0016254 1 10

C0002871 GO:0045923 1 10

C0002871 GO:0032365 1 10

C0002871 GO:0017048 1 10

C0002871 GO:0060021 1 10

C0002871 GO:0042593 1 10

C0002871 GO:0006099 1 10

C0002871 GO:0022405 1 10

C0002871 GO:0042594 1 10

C0002871 GO:0043631 1 10

C0002871 GO:0008603 2 10

C0002871 GO:0006096 1 10

C0002871 GO:0009820 2 10

C0002871 GO:0017080 1 10

C0002871 GO:0003730 1 10

C0002871 GO:0019210 1 10

C0002871 GO:0019213 1 10

C0002871 GO:0006706 2 10

C0002871 GO:0051291 1 10

C0002871 GO:0048592 1 10

C0002871 GO:0002444 1 10

C0002871 GO:0000272 2 10

C0002871 GO:0031047 1 10

C0002871 GO:0002821 1 10

C0002871 GO:0003746 1 10

C0002871 GO:0040017 1 10

C0002871 GO:0009913 1 10

C0002871 GO:0001836 1 10

C0002871 GO:0005765 1 10

C0002871 GO:0006413 2 10

C0002871 GO:0006144 1 10

C0002871 GO:0019363 1 10

C0002871 GO:0006633 1 10

C0002871 GO:0042133 1 10

C0002871 GO:0007612 1 10

C0002871 GO:0000080 1 10

C0002871 GO:0009451 1 10

C0002871 GO:0043523 1 10

C0002871 GO:0043525 1 10

C0002871 GO:0019915 1 10

C0002871 GO:0042765 1 10

C0002871 GO:0016597 1 10

C0002871 GO:0009295 1 10

C0002871 GO:0007004 1 10

C0002871 GO:0004364 2 10

C0002871 GO:0002263 1 10

C0002871 GO:0010257 2 10

C0002871 GO:0005546 1 10

C0002871 GO:0001909 1 10

C0002871 GO:0032135 1 10

C0002871 GO:0015002 2 10

C0002871 GO:0033032 1 10

C0002871 GO:0034366 1 10

C0002871 GO:0016799 1 10

C0002871 GO:0016209 3 10

C0002871 GO:0004385 1 10

C0002871 GO:0016893 1 10

C0002871 GO:0016891 1 10

C0002871 GO:0016896 1 10

C0002871 GO:0009074 1 10

C0002871 GO:0051702 1 10

C0002871 GO:0030261 1 10

C0002871 GO:0045109 1 10

C0002871 GO:0005828 1 10

C0002871 GO:0016566 2 10

C0002871 GO:0004033 1 10

C0002871 GO:0018024 1 10

C0002871 GO:0006297 1 10

C0002871 GO:0017166 1 10

C0002871 GO:0034062 2 10

C0002871 GO:0008250 1 10

C0002871 GO:0048009 1 10

C0002871 GO:0051187 1 10

C0002871 GO:0006563 1 10

C0002871 GO:0016801 1 10

C0002871 GO:0010149 1 10

C0002871 GO:0007183 1 10

C0002871 GO:0000339 1 10

C0002871 GO:0032768 1 10

C0002871 GO:0017015 1 10

C0002871 GO:0019319 1 10

C0002871 GO:0050795 1 10

C0002871 GO:0003231 1 10

C0002871 GO:0046148 1 10

C0002871 GO:0008652 1 10

C0002871 GO:0043666 1 10

C0002871 GO:0010718 1 10

C0002871 GO:0022898 1 10

C0002871 GO:0042267 2 10

C0002871 GO:0010712 1 10

C0002871 GO:0046824 1 10

C0002871 GO:0006518 1 10

C0002871 GO:0001942 1 10

C0002871 GO:0019897 1 10

C0002871 GO:0016796 1 10

C0002871 GO:0006636 1 10

C0002871 GO:0019674 1 10

C0002871 GO:0031099 1 10

C0002871 GO:0009311 1 10

C0002871 GO:0031307 1 10

C0002871 GO:0032967 1 10

C0002871 GO:0004859 1 10

C0002871 GO:0032963 1 10

C0002871 GO:0000209 1 10

C0002871 GO:0000445 2 10

C0002871 GO:0006921 1 10

C0002871 GO:0016706 1 10

C0002871 GO:0004527 1 10

C0002871 GO:0004526 1 10

C0002871 GO:0004521 1 10

C0002871 GO:0050768 1 10

C0002871 GO:0050769 1 10

C0002871 GO:0032409 1 10

C0002871 GO:0031397 1 10

C0002871 GO:0006744 1 10

C0002871 GO:0006743 1 10

C0002871 GO:0006740 1 10

C0002871 GO:0005217 1 10

C0002871 GO:0014065 1 10

C0002871 GO:0006595 1 10

C0002871 GO:0007033 1 10

C0002871 GO:0031625 1 10

C0002871 GO:0002460 1 10

C0002871 GO:0002366 1 10

C0002871 GO:0009084 1 10

C0002871 GO:0016018 1 10

C0002871 GO:0032404 1 10

C0002871 GO:0004177 2 10

C0002871 GO:0050868 1 10

C0002871 GO:0055010 1 10

C0002871 GO:0046365 1 10

C0002871 GO:0046364 1 10

C0002871 GO:0016234 1 10

C0002871 GO:0030983 1 10

C0002871 GO:0009108 1 10

C0002871 GO:0009109 1 10

C0002871 GO:0034623 1 10

C0002871 GO:0034235 1 10

C0002871 GO:0005814 1 10

C0002871 GO:0018279 1 10

C0002871 GO:0045930 1 10

C0002871 GO:0070301 2 10

C0002871 GO:0017119 1 10

C0002871 GO:0045939 1 10

C0002871 GO:0031124 1 10

C0002871 GO:0010812 1 10

C0002871 GO:0031123 1 10

C0002871 GO:0022417 2 10

C0002871 GO:0016903 2 10

C0002871 GO:0007266 1 10

C0002871 GO:0051318 1 10

C0002871 GO:0006970 1 10

C0002871 GO:0002791 1 10

C0002871 GO:0005851 1 10

C0002871 GO:0003706 1 10

C0002871 GO:0070652 1 10

C0002871 GO:0006390 1 10

C0002871 GO:0005786 1 10

C0002871 GO:0006471 1 10

C0002871 GO:0048469 1 10

C0002871 GO:0005788 1 10

C0002871 GO:0006779 1 10

C0002871 GO:0030111 1 10

C0002871 GO:0031056 1 10

C0002871 GO:0030119 1 10

C0002871 GO:0030118 1 10

C0002871 GO:0005072 1 10

C0002871 GO:0016628 1 10

C0002871 GO:0006783 1 10

C0002871 GO:0007213 1 10

C0002871 GO:0016627 2 10

C0002871 GO:0001824 1 10

C0002871 GO:0007216 1 10

C0002871 GO:0016620 2 10

C0002871 GO:0010720 1 10

C0002871 GO:0010721 1 10

C0002871 GO:0019359 1 10

C0002871 GO:0043473 2 10

C0002871 GO:0005092 1 10

C0002871 GO:0000090 1 10

C0002871 GO:0005095 2 10

C0002871 GO:0043536 1 10

C0002871 GO:0043535 1 10

C0002871 GO:0043534 1 10

C0002871 GO:0007076 1 10

C0002871 GO:0003009 1 10

C0002871 GO:0060393 1 10

C0002871 GO:0015012 1 10

C0002871 GO:0004550 1 10

C0002871 GO:0033558 1 10

C0002871 GO:0006672 1 10

C0002871 GO:0030201 1 10

C0002871 GO:0009142 1 10

C0002871 GO:0016278 1 10

C0002871 GO:0016279 1 10

C0002871 GO:0009062 1 10

C0002871 GO:0009060 1 10

C0002871 GO:0016885 1 10

C0002871 GO:0009065 2 10

C0002871 GO:0005852 5 10

C0002871 GO:0090100 1 10

C0002871 GO:0031571 1 10

C0002871 GO:0031345 1 10

C0002871 GO:0031672 1 10

C0002871 GO:0007006 1 10

C0002871 GO:0016575 1 10

C0002871 GO:0009112 1 10

C0002871 GO:0030879 1 10

C0002871 GO:0004129 2 10

C0002871 GO:0004128 3 10

C0002871 GO:0055029 1 10

C0002871 GO:0016303 1 10

C0002871 GO:0001776 1 10

C0002871 GO:0045334 1 10

C0002871 GO:0006189 1 10

C0002871 GO:0006188 1 10

C0002871 GO:0034404 1 10

C0002871 GO:0034654 1 10

C0002871 GO:0001672 1 10

C0002871 GO:0008308 1 10

C0002871 GO:0044275 3 10

C0002871 GO:0000738 1 10

C0002871 GO:0001654 1 10

C0002871 GO:0000737 1 10

C0002871 GO:0000731 1 10

C0002871 GO:0004601 2 10

C0002871 GO:0004602 1 10

C0002871 GO:0032770 1 10

C0002871 GO:0017022 1 10

C0002871 GO:0010927 1 10

C0002871 GO:0045580 1 10

C0002871 GO:0045582 1 10

C0002871 GO:0046677 1 10

C0002871 GO:0003229 1 10

C0002871 GO:0003950 1 10

C0002871 GO:0042572 1 10

C0002871 GO:0045426 1 10

C0002871 GO:0050881 1 10

C0002871 GO:0001701 1 10

C0002871 GO:0051322 1 10

C0002871 GO:0042303 1 10

C0002871 GO:0051087 2 10

C0002871 GO:0042375 1 10

C0002871 GO:0006505 1 10

C0002871 GO:0003756 1 10

C0002871 GO:0006506 1 10

C0002871 GO:0008641 1 10

C0002871 GO:0017156 1 10

C0002871 GO:0030669 1 10

C0002871 GO:0031333 1 10

C0002871 GO:0000217 1 10

C0002871 GO:0004860 1 10

C0002871 GO:0030128 1 10

C0002871 GO:0030122 1 10

C0002871 GO:0030656 1 10

C0002871 GO:0030125 1 10

C0002871 GO:0050770 1 10

C0002871 GO:0001959 2 10

C0002871 GO:0050772 1 10

C0002871 GO:0015884 1 10

C0002871 GO:0000428 1 10

C0002871 GO:0001952 1 10

C0002871 GO:0008535 1 10

C0002871 GO:0005742 1 10

C0002871 GO:0000956 1 10

C0002871 GO:0005744 1 10

C0002871 GO:0016653 2 10

C0002871 GO:0010770 1 10

C0002871 GO:0006007 2 10

C0002871 GO:0002039 1 10

C0002871 GO:0046112 1 10

C0002871 GO:0043506 1 10

C0002871 GO:0032986 1 10

C0002871 GO:0070822 1 10

C0002871 GO:0032984 1 10

C0002871 GO:0032981 2 10

C0002871 GO:0031638 1 10

C0002871 GO:0045766 1 10

C0002871 GO:0005310 1 10

C0002871 GO:0046040 1 10

C0002871 GO:0046426 2 10

C0002871 GO:0046356 1 10

C0002871 GO:0030239 1 10

C0002871 GO:0007585 1 10

C0002871 GO:0034614 2 10

C0002871 GO:0016878 1 10

C0002871 GO:0000779 2 10

C0002871 GO:0042026 1 10

C0002871 GO:0009116 1 10

C0002871 GO:0009119 1 10

C0002871 GO:0000777 1 10

C0002871 GO:0000070 1 10

C0002871 GO:0001516 1 10

C0002871 GO:0051293 1 10

C0002871 GO:0019842 1 10

C0002871 GO:0000175 1 10

C0002871 GO:0046457 1 10

C0002871 GO:0046456 1 10

C0002871 GO:0043596 1 10

C0002871 GO:0046519 1 10

C0002871 GO:0000796 1 10

C0002871 GO:0046638 1 10

C0002871 GO:0046637 1 10

C0002871 GO:0051287 1 10

C0002871 GO:0046635 1 10

C0002871 GO:0046634 1 10

C0002871 GO:0016646 1 10

C0002871 GO:0050920 1 10

C0002871 GO:0050921 1 10

C0002871 GO:0045309 1 10

C0002871 GO:0051893 1 10

C0002871 GO:0034765 1 10

C0002871 GO:0034762 1 10

C0002871 GO:0018196 1 10

C0002871 GO:0015238 1 10

C0002871 GO:0008143 1 10

C0002871 GO:0010894 1 10

C0002871 GO:0032393 2 10

C0002871 GO:0030165 1 10

C0002871 GO:0010975 1 10

C0002871 GO:0030511 1 10

C0002871 GO:0005062 1 10

C0002871 GO:0005665 1 10

C0002871 GO:0048500 1 10

C0002871 GO:0019439 1 10

C0002871 GO:0060415 1 10

C0002871 GO:0001953 1 10

C0002871 GO:0042744 2 10

C0002871 GO:0005871 1 10

C0002871 GO:0031369 2 10

C0002871 GO:0055102 1 10

C0002871 GO:0008408 2 10

C0002871 GO:0000381 1 10

C0002871 GO:0008406 1 10

C0002871 GO:0010810 1 10

C0002871 GO:0033014 1 10

C0002871 GO:0006684 1 10

C0002871 GO:0016684 2 10

C0002871 GO:0016769 1 10

C0002871 GO:0009156 1 10

C0002871 GO:0005338 1 10

C0002871 GO:0009152 1 10

C0002871 GO:0048256 2 10

C0002871 GO:0042633 1 10

C0002871 GO:0045165 1 10

C0002871 GO:0000132 2 10

C0002871 GO:0004683 1 10

C0002871 GO:0031647 2 10

C0002871 GO:0030867 2 10

C0002871 GO:0045730 1 10

C0002871 GO:0016469 1 10

C0002871 GO:0002819 1 10

C0002871 GO:0051004 2 10

C0002871 GO:0051000 1 10

C0002871 GO:0009650 2 10

C0002871 GO:0009127 1 10

C0002871 GO:0044246 1 10

C0002871 GO:0006220 1 10

C0002871 GO:0006221 1 10

C0002871 GO:0070603 1 10

C0002871 GO:0001541 1 10

C0002871 GO:0006342 1 10

C0002871 GO:0042921 1 10

C0002871 GO:0046784 2 10

C0002871 GO:0046782 1 10

C0002871 GO:0044452 1 10

C0002871 GO:0010939 1 10

C0002871 GO:0003709 1 10

C0002871 GO:0046165 1 10

C0002871 GO:0015298 1 10

C0002871 GO:0035035 1 10

C0002871 GO:0055088 1 10

C0002871 GO:0010833 1 10

C0002871 GO:0051310 1 10

C0002871 GO:0055085 1 10

C0002871 GO:0006084 1 10

C0002871 GO:0008634 2 10

C0002871 GO:0008637 2 10

C0002871 GO:0006081 2 10

C0002871 GO:0043603 1 10

C0002871 GO:0070776 1 10

C0002871 GO:0070775 1 10

C0002871 GO:0043370 1 10

C0002871 GO:0045861 1 10

C0002871 GO:0034976 1 10

C0002871 GO:0000184 1 10

C0002871 GO:0055001 1 10

C0002871 GO:0000347 2 10

C0002871 GO:0000346 2 10

C0002871 GO:0030131 1 10

C0002871 GO:0030132 1 10

C0002871 GO:0030137 1 10

C0002871 GO:0050661 1 10

C0002871 GO:0040001 1 10

C0002871 GO:0033500 1 10

C0002871 GO:0016645 1 10

C0002871 GO:0008526 1 10

C0002871 GO:0005753 1 10

C0002871 GO:0010466 1 10

C0002871 GO:0006400 1 10

C0002871 GO:0031334 1 10

C0002871 GO:0008210 1 10

C0002871 GO:0019104 2 10

C0002871 GO:0005881 1 10

C0002871 GO:0005883 1 10

C0002871 GO:0007159 1 10

C0002871 GO:0007157 1 10

C0002871 GO:0002443 1 10

C0002871 GO:0042772 1 10

C0002871 GO:0002446 1 10

C0002871 GO:0016580 1 10

C0002871 GO:0016581 1 10

C0002871 GO:0010714 1 10

C0002871 GO:0001938 1 10

C0002871 GO:0004576 1 10

C0002871 GO:0002250 1 10

C0002871 GO:0004549 1 10

C0002871 GO:0015030 1 10

C0002871 GO:0001933 2 10

C0002871 GO:0004579 1 10

C0002871 GO:0001937 1 10

C0002871 GO:0001936 1 10

C0004134 GO:0060260 1 2

C0004134 GO:0018024 1 2

C0004134 GO:0006297 1 2

C0004134 GO:0000737 1 2

C0004134 GO:0030201 1 2

C0004134 GO:0004128 1 2

C0004134 GO:0009142 1 2

C0004134 GO:0016799 1 2

C0004134 GO:0034062 1 2

C0004134 GO:0016278 1 2

C0004134 GO:0033683 1 2

C0004134 GO:0031307 1 2

C0004134 GO:0042744 1 2

C0004134 GO:0005828 1 2

C0004134 GO:0005487 1 2

C0004134 GO:0060393 1 2

C0004134 GO:0000209 1 2

C0004134 GO:0005876 1 2

C0004134 GO:0017119 1 2

C0004134 GO:0006220 1 2

C0004134 GO:0009394 1 2

C0004134 GO:0005072 1 2

C0004134 GO:0004364 1 2

C0004134 GO:0005852 2 2

C0004134 GO:0032981 1 2

C0004134 GO:0006783 1 2

C0004134 GO:0007213 1 2

C0004134 GO:0010149 1 2

C0004134 GO:0004521 1 2

C0004134 GO:0010257 1 2

C0004134 GO:0055085 1 2

C0004134 GO:0033014 1 2

C0004134 GO:0006684 1 2

C0004134 GO:0008408 1 2

C0004134 GO:0004683 1 2

C0004134 GO:0006779 1 2

C0004134 GO:0003899 1 2

C0004134 GO:0033108 1 2

C0004134 GO:0006706 1 2

C0004134 GO:0009262 1 2

C0004134 GO:0034614 1 2

C0004134 GO:0016209 1 2

C0004134 GO:0070301 1 2

C0004134 GO:0004385 1 2

C0004134 GO:0000090 1 2

C0004134 GO:0019104 1 2

C0004134 GO:0000779 1 2

C0004134 GO:0005883 1 2

C0004134 GO:0016891 1 2

C0004134 GO:0005913 1 2

C0004134 GO:0031047 1 2

C0004134 GO:0016893 1 2

C0004134 GO:0015030 1 2

C0004134 GO:0009084 1 2

C0004134 GO:0016279 1 2

C0004134 GO:0001672 1 2

C0004134 GO:0051322 1 2

C0004134 GO:0000777 1 2

C0004134 GO:0016653 1 2

C0004134 GO:0032365 1 2

C0004134 GO:0015884 1 2

C0004134 GO:0031647 1 2

C0004134 GO:0000731 1 2

C0004134 GO:0010718 1 2

C0004134 GO:0015012 1 2

C0004134 GO:0048365 1 2

C0004134 GO:0043596 1 2

C0004134 GO:0043331 1 2

C0004134 GO:0046148 1 2

C0004134 GO:0010770 1 2

C0004134 GO:0006413 1 2

C0004134 GO:0006595 1 2

C0004134 GO:0009264 1 2

C0007787 GO:0060260 1 1

C0007787 GO:0018024 1 1

C0007787 GO:0006297 1 1

C0007787 GO:0000737 1 1

C0007787 GO:0030201 1 1

C0007787 GO:0009142 1 1

C0007787 GO:0016278 1 1

C0007787 GO:0033683 1 1

C0007787 GO:0005828 1 1

C0007787 GO:0005487 1 1

C0007787 GO:0060393 1 1

C0007787 GO:0000209 1 1

C0007787 GO:0005876 1 1

C0007787 GO:0005072 1 1

C0007787 GO:0004364 1 1

C0007787 GO:0005852 1 1

C0007787 GO:0032981 1 1

C0007787 GO:0010257 1 1

C0007787 GO:0055085 1 1

C0007787 GO:0033108 1 1

C0007787 GO:0006706 1 1

C0007787 GO:0004385 1 1

C0007787 GO:0000090 1 1

C0007787 GO:0000779 1 1

C0007787 GO:0005883 1 1

C0007787 GO:0005913 1 1

C0007787 GO:0031047 1 1

C0007787 GO:0016279 1 1

C0007787 GO:0001672 1 1

C0007787 GO:0051322 1 1

C0007787 GO:0000777 1 1

C0007787 GO:0032365 1 1

C0007787 GO:0015884 1 1

C0007787 GO:0031647 1 1

C0007787 GO:0000731 1 1

C0007787 GO:0010718 1 1

C0007787 GO:0015012 1 1

C0007787 GO:0043596 1 1

C0007787 GO:0043331 1 1

C0007787 GO:0010770 1 1

C0007787 GO:0006413 1 1

C0009763 GO:0042133 1 4

C0009763 GO:0000245 1 4

C0009763 GO:0022624 1 4

C0009763 GO:0032770 1 4

C0009763 GO:0050684 1 4

C0009763 GO:0005788 1 4

C0009763 GO:0045259 1 4

C0009763 GO:0002706 1 4

C0009763 GO:0007266 1 4

C0009763 GO:0009108 1 4

C0009763 GO:0034199 1 4

C0009763 GO:0019915 1 4

C0009763 GO:0034765 1 4

C0009763 GO:0016864 1 4

C0009763 GO:0016862 1 4

C0009763 GO:0002709 1 4

C0009763 GO:0007004 1 4

C0009763 GO:0005072 1 4

C0009763 GO:0004364 1 4

C0009763 GO:0005838 1 4

C0009763 GO:0016628 1 4

C0009763 GO:0042267 1 4

C0009763 GO:0005834 1 4

C0009763 GO:0000302 1 4

C0009763 GO:0016627 1 4

C0009763 GO:0018196 1 4

C0009763 GO:0043043 1 4

C0009763 GO:0010257 3 4

C0009763 GO:0007080 1 4

C0009763 GO:0010894 1 4

C0009763 GO:0010720 1 4

C0009763 GO:0051287 1 4

C0009763 GO:0032135 1 4

C0009763 GO:0015002 1 4

C0009763 GO:0032393 1 4

C0009763 GO:0006760 1 4

C0009763 GO:0034366 1 4

C0009763 GO:0005092 2 4

C0009763 GO:0033500 1 4

C0009763 GO:0002824 1 4

C0009763 GO:0004385 2 4

C0009763 GO:0000090 1 4

C0009763 GO:0006740 1 4

C0009763 GO:0002821 1 4

C0009763 GO:0003841 1 4

C0009763 GO:0016891 1 4

C0009763 GO:0000796 1 4

C0009763 GO:0007172 1 4

C0009763 GO:0010975 1 4

C0009763 GO:0007076 1 4

C0009763 GO:0000502 1 4

C0009763 GO:0009451 1 4

C0009763 GO:0009071 1 4

C0009763 GO:0003009 1 4

C0009763 GO:0006081 1 4

C0009763 GO:0051004 1 4

C0009763 GO:0045793 1 4

C0009763 GO:0006278 1 4

C0009763 GO:0018209 1 4

C0009763 GO:0001959 1 4

C0009763 GO:0006271 1 4

C0009763 GO:0005828 1 4

C0009763 GO:0000018 1 4

C0009763 GO:0016814 1 4

C0009763 GO:0060393 1 4

C0009763 GO:0015012 1 4

C0009763 GO:0005663 1 4

C0009763 GO:0010884 1 4

C0009763 GO:0043331 1 4

C0009763 GO:0006164 1 4

C0009763 GO:0019439 1 4

C0009763 GO:0004859 1 4

C0009763 GO:0016445 1 4

C0009763 GO:0048256 1 4

C0009763 GO:0060260 1 4

C0009763 GO:0018024 1 4

C0009763 GO:0006297 2 4

C0009763 GO:0017166 1 4

C0009763 GO:0002839 1 4

C0009763 GO:0030201 1 4

C0009763 GO:0031647 1 4

C0009763 GO:0002834 1 4

C0009763 GO:0009142 1 4

C0009763 GO:0002836 1 4

C0009763 GO:0042559 1 4

C0009763 GO:0016278 1 4

C0009763 GO:0016279 1 4

C0009763 GO:0004693 1 4

C0009763 GO:0001763 1 4

C0009763 GO:0016885 1 4

C0009763 GO:0009065 1 4

C0009763 GO:0005487 2 4

C0009763 GO:0055102 1 4

C0009763 GO:0016607 1 4

C0009763 GO:0016289 1 4

C0009763 GO:0005852 3 4

C0009763 GO:0005851 1 4

C0009763 GO:0016801 1 4

C0009763 GO:0031672 1 4

C0009763 GO:0016528 1 4

C0009763 GO:0009127 1 4

C0009763 GO:0009168 1 4

C0009763 GO:0000339 1 4

C0009763 GO:0002200 1 4

C0009763 GO:0016860 1 4

C0009763 GO:0016769 1 4

C0009763 GO:0007006 1 4

C0009763 GO:0004129 1 4

C0009763 GO:0004128 1 4

C0009763 GO:0032813 1 4

C0009763 GO:0009156 1 4

C0009763 GO:0032768 1 4

C0009763 GO:0009152 2 4

C0009763 GO:0019319 2 4

C0009763 GO:0009260 2 4

C0009763 GO:0009262 1 4

C0009763 GO:0006189 1 4

C0009763 GO:0006188 1 4

C0009763 GO:0002711 1 4

C0009763 GO:0009084 1 4

C0009763 GO:0042633 1 4

C0009763 GO:0030069 1 4

C0009763 GO:0000272 1 4

C0009763 GO:0001672 2 4

C0009763 GO:0005720 1 4

C0009763 GO:0005844 1 4

C0009763 GO:0006007 1 4

C0009763 GO:0000738 1 4

C0009763 GO:0031080 1 4

C0009763 GO:0010718 1 4

C0009763 GO:0022898 1 4

C0009763 GO:0001776 1 4

C0009763 GO:0000737 1 4

C0009763 GO:0030867 1 4

C0009763 GO:0000731 1 4

C0009763 GO:0015074 1 4

C0009763 GO:0016676 1 4

C0009763 GO:0051087 1 4

C0009763 GO:0016675 1 4

C0009763 GO:0007159 1 4

C0009763 GO:0045739 1 4

C0009763 GO:0006026 1 4

C0009763 GO:0006027 1 4

C0009763 GO:0004602 1 4

C0009763 GO:0009264 1 4

C0009763 GO:0019897 1 4

C0009763 GO:0004033 1 4

C0009763 GO:0004526 1 4

C0009763 GO:0030307 1 4

C0009763 GO:0009311 1 4

C0009763 GO:0017022 1 4

C0009763 GO:0010927 1 4

C0009763 GO:0030261 1 4

C0009763 GO:0031306 1 4

C0009763 GO:0051000 1 4

C0009763 GO:0032963 1 4

C0009763 GO:0048024 1 4

C0009763 GO:0043206 1 4

C0009763 GO:0042375 1 4

C0009763 GO:0000209 1 4

C0009763 GO:0005876 1 4

C0009763 GO:0050868 1 4

C0009763 GO:0009395 1 4

C0009763 GO:0009394 1 4

C0009763 GO:0050881 1 4

C0009763 GO:0070603 1 4

C0009763 GO:0032412 1 4

C0009763 GO:0051322 1 4

C0009763 GO:0009074 1 4

C0009763 GO:0042303 1 4

C0009763 GO:0001942 1 4

C0009763 GO:0043484 1 4

C0009763 GO:0031099 1 4

C0009763 GO:0033683 1 4

C0009763 GO:0050769 1 4

C0009763 GO:0032409 1 4

C0009763 GO:0002228 1 4

C0009763 GO:0003756 1 4

C0009763 GO:0051310 1 4

C0009763 GO:0021954 1 4

C0009763 GO:0005506 1 4

C0009763 GO:0006744 1 4

C0009763 GO:0006743 1 4

C0009763 GO:0002837 1 4

C0009763 GO:0021953 1 4

C0009763 GO:0045749 1 4

C0009763 GO:0019902 1 4

C0009763 GO:0033108 3 4

C0009763 GO:0055002 1 4

C0009763 GO:0042288 1 4

C0009763 GO:0008139 1 4

C0009763 GO:0046165 1 4

C0009763 GO:0016514 1 4

C0009763 GO:0007216 1 4

C0009763 GO:0055088 1 4

C0009763 GO:0005913 1 4

C0009763 GO:0034061 1 4

C0009763 GO:0044275 1 4

C0009763 GO:0002673 1 4

C0009763 GO:0003995 1 4

C0009763 GO:0008250 1 4

C0009763 GO:0055085 1 4

C0009763 GO:0030656 1 4

C0009763 GO:0030530 1 4

C0009763 GO:0045426 1 4

C0009763 GO:0050770 1 4

C0009763 GO:0043603 1 4

C0009763 GO:0008483 1 4

C0009763 GO:0015884 1 4

C0009763 GO:0042542 1 4

C0009763 GO:0018105 1 4

C0009763 GO:0022616 1 4

C0009763 GO:0005742 1 4

C0009763 GO:0003727 1 4

C0009763 GO:0006536 1 4

C0009763 GO:0016529 1 4

C0009763 GO:0016653 1 4

C0009763 GO:0010770 1 4

C0009763 GO:0034762 1 4

C0009763 GO:0032404 1 4

C0009763 GO:0006000 1 4

C0009763 GO:0018279 1 4

C0009763 GO:0046889 1 4

C0009763 GO:0004177 2 4

C0009763 GO:0032365 1 4

C0009763 GO:0008484 1 4

C0009763 GO:0055001 1 4

C0009763 GO:0046365 1 4

C0009763 GO:0046364 2 4

C0009763 GO:0048754 1 4

C0009763 GO:0030983 1 4

C0009763 GO:0042593 1 4

C0009763 GO:0022404 1 4

C0009763 GO:0022405 1 4

C0009763 GO:0046040 1 4

C0009763 GO:0008603 1 4

C0009763 GO:0004653 1 4

C0009763 GO:0016684 1 4

C0009763 GO:0032981 3 4

C0009763 GO:0050879 1 4

C0009763 GO:0031638 1 4

C0009763 GO:0016645 1 4

C0009763 GO:0005753 1 4

C0009763 GO:0003730 1 4

C0009763 GO:0005310 1 4

C0009763 GO:0006400 1 4

C0009763 GO:0004576 1 4

C0009763 GO:0016209 1 4

C0009763 GO:0022417 1 4

C0009763 GO:0045930 1 4

C0009763 GO:0050772 1 4

C0009763 GO:0006706 2 4

C0009763 GO:0070120 1 4

C0009763 GO:0030239 1 4

C0009763 GO:0045939 1 4

C0009763 GO:0007585 1 4

C0009763 GO:0008210 1 4

C0009763 GO:0004549 1 4

C0009763 GO:0050690 1 4

C0009763 GO:0006607 1 4

C0009763 GO:0016878 1 4

C0009763 GO:0000779 1 4

C0009763 GO:0005883 1 4

C0009763 GO:0016903 1 4

C0009763 GO:0042026 1 4

C0009763 GO:0031047 1 4

C0009763 GO:0051646 1 4

C0009763 GO:0000381 1 4

C0009763 GO:0007157 1 4

C0009763 GO:0016469 1 4

C0009763 GO:0000777 1 4

C0009763 GO:0051881 1 4

C0009763 GO:0030532 1 4

C0009763 GO:0009913 1 4

C0009763 GO:0005217 1 4

C0009763 GO:0009820 1 4

C0009763 GO:0019047 1 4

C0009763 GO:0032200 1 4

C0009763 GO:0019842 1 4

C0009763 GO:0004601 1 4

C0009763 GO:0043596 1 4

C0009763 GO:0000790 1 4

C0009763 GO:0000387 1 4

C0009763 GO:0006390 1 4

C0009763 GO:0004579 1 4

C0009763 GO:0006413 1 4

C0009763 GO:0002833 1 4

C0009763 GO:0010833 1 4

C0015230 GO:0010149 1 12

C0015230 GO:0007598 1 12

C0015230 GO:0043954 2 12

C0015230 GO:0048066 1 12

C0015230 GO:0002706 1 12

C0015230 GO:0002703 1 12

C0015230 GO:0034199 1 12

C0015230 GO:0002700 1 12

C0015230 GO:0016864 1 12

C0015230 GO:0016862 1 12

C0015230 GO:0009168 1 12

C0015230 GO:0016860 1 12

C0015230 GO:0000002 1 12

C0015230 GO:0043206 1 12

C0015230 GO:0005834 2 12

C0015230 GO:0004653 1 12

C0015230 GO:0002366 1 12

C0015230 GO:0016514 1 12

C0015230 GO:0046504 1 12

C0015230 GO:0051294 1 12

C0015230 GO:0002822 2 12

C0015230 GO:0031109 1 12

C0015230 GO:0021510 1 12

C0015230 GO:0035270 1 12

C0015230 GO:0002673 1 12

C0015230 GO:0000718 1 12

C0015230 GO:0045259 1 12

C0015230 GO:0016814 2 12

C0015230 GO:0010887 2 12

C0015230 GO:0010885 1 12

C0015230 GO:0010884 2 12

C0015230 GO:0043331 1 12

C0015230 GO:0003995 2 12

C0015230 GO:0006378 1 12

C0015230 GO:0060260 1 12

C0015230 GO:0031958 1 12

C0015230 GO:0032412 1 12

C0015230 GO:0048568 1 12

C0015230 GO:0000302 1 12

C0015230 GO:0030174 1 12

C0015230 GO:0008484 1 12

C0015230 GO:0046651 1 12

C0015230 GO:0005487 1 12

C0015230 GO:0016607 1 12

C0015230 GO:0015992 1 12

C0015230 GO:0015491 1 12

C0015230 GO:0042551 2 12

C0015230 GO:0006885 1 12

C0015230 GO:0042255 1 12

C0015230 GO:0042558 1 12

C0015230 GO:0070742 1 12

C0015230 GO:0002687 1 12

C0015230 GO:0002685 1 12

C0015230 GO:0043021 2 12

C0015230 GO:0043022 2 12

C0015230 GO:0043027 1 12

C0015230 GO:0009304 3 12

C0015230 GO:0032479 2 12

C0015230 GO:0048709 1 12

C0015230 GO:0009260 2 12

C0015230 GO:0009262 1 12

C0015230 GO:0009264 1 12

C0015230 GO:0009266 1 12

C0015230 GO:0006303 1 12

C0015230 GO:0044042 1 12

C0015230 GO:0030675 1 12

C0015230 GO:0031080 1 12

C0015230 GO:0004536 1 12

C0015230 GO:0015781 1 12

C0015230 GO:0015780 1 12

C0015230 GO:0035095 1 12

C0015230 GO:0048365 1 12

C0015230 GO:0016676 1 12

C0015230 GO:0015179 1 12

C0015230 GO:0016675 1 12

C0015230 GO:0015175 1 12

C0015230 GO:0006026 1 12

C0015230 GO:0006027 1 12

C0015230 GO:0030880 1 12

C0015230 GO:0050690 2 12

C0015230 GO:0033209 1 12

C0015230 GO:0008144 1 12

C0015230 GO:0014003 1 12

C0015230 GO:0005876 1 12

C0015230 GO:0009395 1 12

C0015230 GO:0009394 1 12

C0015230 GO:0033683 1 12

C0015230 GO:0002228 1 12

C0015230 GO:0045621 1 12

C0015230 GO:0005506 1 12

C0015230 GO:0045622 1 12

C0015230 GO:0045749 1 12

C0015230 GO:0033108 2 12

C0015230 GO:0055002 1 12

C0015230 GO:0006073 1 12

C0015230 GO:0042288 1 12

C0015230 GO:0050879 1 12

C0015230 GO:0005913 1 12

C0015230 GO:0001656 1 12

C0015230 GO:0046889 1 12

C0015230 GO:0006978 1 12

C0015230 GO:0042542 3 12

C0015230 GO:0022404 1 12

C0015230 GO:0016528 1 12

C0015230 GO:0016529 1 12

C0015230 GO:0003899 3 12

C0015230 GO:0017046 1 12

C0015230 GO:0045923 1 12

C0015230 GO:0042267 1 12

C0015230 GO:0060021 1 12

C0015230 GO:0042593 1 12

C0015230 GO:0006099 1 12

C0015230 GO:0022405 1 12

C0015230 GO:0007271 1 12

C0015230 GO:0007270 1 12

C0015230 GO:0043631 1 12

C0015230 GO:0008603 2 12

C0015230 GO:0009820 1 12

C0015230 GO:0033993 1 12

C0015230 GO:0003730 2 12

C0015230 GO:0045089 1 12

C0015230 GO:0006706 2 12

C0015230 GO:0048593 1 12

C0015230 GO:0000272 1 12

C0015230 GO:0031047 1 12

C0015230 GO:0000445 2 12

C0015230 GO:0040017 1 12

C0015230 GO:0009913 1 12

C0015230 GO:0001837 1 12

C0015230 GO:0005763 1 12

C0015230 GO:0048524 1 12

C0015230 GO:0006413 2 12

C0015230 GO:0015184 1 12

C0015230 GO:0042133 1 12

C0015230 GO:0009593 1 12

C0015230 GO:0009311 1 12

C0015230 GO:0007612 1 12

C0015230 GO:0000080 1 12

C0015230 GO:0007163 1 12

C0015230 GO:0043525 1 12

C0015230 GO:0019915 1 12

C0015230 GO:0004364 1 12

C0015230 GO:0002263 1 12

C0015230 GO:0010257 2 12

C0015230 GO:0001909 1 12

C0015230 GO:0032135 1 12

C0015230 GO:0015002 1 12

C0015230 GO:0033032 2 12

C0015230 GO:0034366 1 12

C0015230 GO:0005310 1 12

C0015230 GO:0016209 2 12

C0015230 GO:0004385 1 12

C0015230 GO:0016893 1 12

C0015230 GO:0016891 1 12

C0015230 GO:0000796 1 12

C0015230 GO:0009074 2 12

C0015230 GO:0051004 1 12

C0015230 GO:0005828 1 12

C0015230 GO:0004707 1 12

C0015230 GO:0016444 1 12

C0015230 GO:0004033 1 12

C0015230 GO:0018024 2 12

C0015230 GO:0006297 2 12

C0015230 GO:0017166 1 12

C0015230 GO:0034062 3 12

C0015230 GO:0008250 1 12

C0015230 GO:0048009 1 12

C0015230 GO:0001764 1 12

C0015230 GO:0005929 1 12

C0015230 GO:0045088 1 12

C0015230 GO:0051184 1 12

C0015230 GO:0006563 2 12

C0015230 GO:0002377 1 12

C0015230 GO:0016801 1 12

C0015230 GO:0042098 1 12

C0015230 GO:0007183 1 12

C0015230 GO:0016363 1 12

C0015230 GO:0032768 1 12

C0015230 GO:0032769 1 12

C0015230 GO:0017015 1 12

C0015230 GO:0019319 1 12

C0015230 GO:0050795 1 12

C0015230 GO:0046148 1 12

C0015230 GO:0016944 1 12

C0015230 GO:0010718 1 12

C0015230 GO:0022898 1 12

C0015230 GO:0032365 1 12

C0015230 GO:0004527 1 12

C0015230 GO:0019897 2 12

C0015230 GO:0016796 1 12

C0015230 GO:0031099 1 12

C0015230 GO:0016799 2 12

C0015230 GO:0001945 1 12

C0015230 GO:0031307 1 12

C0015230 GO:0004859 1 12

C0015230 GO:0007041 1 12

C0015230 GO:0032963 1 12

C0015230 GO:0000428 1 12

C0015230 GO:0000209 1 12

C0015230 GO:0016706 1 12

C0015230 GO:0001942 1 12

C0015230 GO:0004521 1 12

C0015230 GO:0004520 1 12

C0015230 GO:0050769 1 12

C0015230 GO:0032409 1 12

C0015230 GO:0015165 2 12

C0015230 GO:0031397 1 12

C0015230 GO:0032405 1 12

C0015230 GO:0006740 1 12

C0015230 GO:0005217 1 12

C0015230 GO:0014065 1 12

C0015230 GO:0006595 1 12

C0015230 GO:0043473 1 12

C0015230 GO:0002460 1 12

C0015230 GO:0008483 1 12

C0015230 GO:0045619 1 12

C0015230 GO:0009084 1 12

C0015230 GO:0016018 2 12

C0015230 GO:0032404 3 12

C0015230 GO:0004177 2 12

C0015230 GO:0050868 1 12

C0015230 GO:0046365 1 12

C0015230 GO:0046364 1 12

C0015230 GO:0030983 1 12

C0015230 GO:0046040 1 12

C0015230 GO:0009109 1 12

C0015230 GO:0016840 1 12

C0015230 GO:0000060 1 12

C0015230 GO:0005814 1 12

C0015230 GO:0018279 1 12

C0015230 GO:0045930 1 12

C0015230 GO:0070301 2 12

C0015230 GO:0017119 1 12

C0015230 GO:0045939 2 12

C0015230 GO:0031124 1 12

C0015230 GO:0010812 1 12

C0015230 GO:0031123 1 12

C0015230 GO:0022417 1 12

C0015230 GO:0016903 2 12

C0015230 GO:0055067 1 12

C0015230 GO:0016505 1 12

C0015230 GO:0007266 1 12

C0015230 GO:0003709 2 12

C0015230 GO:0042346 1 12

C0015230 GO:0042345 1 12

C0015230 GO:0002793 1 12

C0015230 GO:0032202 1 12

C0015230 GO:0005662 2 12

C0015230 GO:0043548 1 12

C0015230 GO:0005786 1 12

C0015230 GO:0048469 1 12

C0015230 GO:0005788 1 12

C0015230 GO:0006779 1 12

C0015230 GO:0005072 1 12

C0015230 GO:0016628 1 12

C0015230 GO:0006783 1 12

C0015230 GO:0007213 1 12

C0015230 GO:0016627 1 12

C0015230 GO:0007216 1 12

C0015230 GO:0010720 1 12

C0015230 GO:0005678 1 12

C0015230 GO:0000099 1 12

C0015230 GO:0005092 1 12

C0015230 GO:0000090 1 12

C0015230 GO:0005095 1 12

C0015230 GO:0043535 1 12

C0015230 GO:0019905 1 12

C0015230 GO:0007076 1 12

C0015230 GO:0055117 1 12

C0015230 GO:0003009 1 12

C0015230 GO:0060393 1 12

C0015230 GO:0015012 1 12

C0015230 GO:0043523 1 12

C0015230 GO:0006672 2 12

C0015230 GO:0045494 2 12

C0015230 GO:0050921 1 12

C0015230 GO:0030201 1 12

C0015230 GO:0009142 1 12

C0015230 GO:0016278 2 12

C0015230 GO:0016279 2 12

C0015230 GO:0000783 1 12

C0015230 GO:0000782 1 12

C0015230 GO:0009065 1 12

C0015230 GO:0031576 1 12

C0015230 GO:0005852 5 12

C0015230 GO:0090100 1 12

C0015230 GO:0005851 1 12

C0015230 GO:0000149 1 12

C0015230 GO:0031672 1 12

C0015230 GO:0051896 1 12

C0015230 GO:0016571 1 12

C0015230 GO:0005522 1 12

C0015230 GO:0016471 1 12

C0015230 GO:0004129 1 12

C0015230 GO:0004128 2 12

C0015230 GO:0006818 1 12

C0015230 GO:0015908 1 12

C0015230 GO:0055029 1 12

C0015230 GO:0001776 1 12

C0015230 GO:0006189 1 12

C0015230 GO:0006188 1 12

C0015230 GO:0001672 1 12

C0015230 GO:0044275 2 12

C0015230 GO:0008091 1 12

C0015230 GO:0000737 1 12

C0015230 GO:0000731 1 12

C0015230 GO:0006739 1 12

C0015230 GO:0006359 1 12

C0015230 GO:0033176 1 12

C0015230 GO:0032770 1 12

C0015230 GO:0017022 1 12

C0015230 GO:0010927 1 12

C0015230 GO:0045580 2 12

C0015230 GO:0045582 2 12

C0015230 GO:0046677 1 12

C0015230 GO:0001953 1 12

C0015230 GO:0034399 1 12

C0015230 GO:0050881 1 12

C0015230 GO:0001707 1 12

C0015230 GO:0001704 1 12

C0015230 GO:0051322 1 12

C0015230 GO:0042303 1 12

C0015230 GO:0051087 1 12

C0015230 GO:0070761 1 12

C0015230 GO:0033135 1 12

C0015230 GO:0017156 1 12

C0015230 GO:0048332 1 12

C0015230 GO:0031333 1 12

C0015230 GO:0000217 2 12

C0015230 GO:0000738 1 12

C0015230 GO:0030656 1 12

C0015230 GO:0050770 1 12

C0015230 GO:0001959 1 12

C0015230 GO:0050772 1 12

C0015230 GO:0015884 1 12

C0015230 GO:0032182 1 12

C0015230 GO:0001952 1 12

C0015230 GO:0016653 3 12

C0015230 GO:0010770 1 12

C0015230 GO:0006007 1 12

C0015230 GO:0002039 1 12

C0015230 GO:0042116 1 12

C0015230 GO:0004532 1 12

C0015230 GO:0042113 1 12

C0015230 GO:0070822 2 12

C0015230 GO:0043467 1 12

C0015230 GO:0032981 2 12

C0015230 GO:0031638 1 12

C0015230 GO:0045766 1 12

C0015230 GO:0006695 1 12

C0015230 GO:0016229 1 12

C0015230 GO:0046356 1 12

C0015230 GO:0030239 1 12

C0015230 GO:0007585 2 12

C0015230 GO:0034614 1 12

C0015230 GO:0006911 1 12

C0015230 GO:0016878 1 12

C0015230 GO:0000779 2 12

C0015230 GO:0009110 1 12

C0015230 GO:0042026 1 12

C0015230 GO:0009116 1 12

C0015230 GO:0051646 1 12

C0015230 GO:0070925 1 12

C0015230 GO:0000777 1 12

C0015230 GO:0000178 1 12

C0015230 GO:0001516 1 12

C0015230 GO:0019842 1 12

C0015230 GO:0000175 1 12

C0015230 GO:0046457 1 12

C0015230 GO:0046456 1 12

C0015230 GO:0043596 2 12

C0015230 GO:0004602 1 12

C0015230 GO:0046519 2 12

C0015230 GO:0016504 1 12

C0015230 GO:0016896 1 12

C0015230 GO:0046638 2 12

C0015230 GO:0046637 1 12

C0015230 GO:0051287 1 12

C0015230 GO:0046635 1 12

C0015230 GO:0046634 1 12

C0015230 GO:0046631 1 12

C0015230 GO:0016646 1 12

C0015230 GO:0050920 1 12

C0015230 GO:0016645 2 12

C0015230 GO:0051893 1 12

C0015230 GO:0034765 1 12

C0015230 GO:0008278 1 12

C0015230 GO:0034762 1 12

C0015230 GO:0015804 1 12

C0015230 GO:0006983 1 12

C0015230 GO:0018196 1 12

C0015230 GO:0008143 1 12

C0015230 GO:0006266 2 12

C0015230 GO:0010894 2 12

C0015230 GO:0006383 1 12

C0015230 GO:0032393 1 12

C0015230 GO:0000314 1 12

C0015230 GO:0030165 1 12

C0015230 GO:0010975 1 12

C0015230 GO:0030511 1 12

C0015230 GO:0051354 1 12

C0015230 GO:0005663 1 12

C0015230 GO:0043130 1 12

C0015230 GO:0048500 1 12

C0015230 GO:0019439 1 12

C0015230 GO:0002821 1 12

C0015230 GO:0005086 1 12

C0015230 GO:0042744 1 12

C0015230 GO:0031369 2 12

C0015230 GO:0055102 1 12

C0015230 GO:0008408 2 12

C0015230 GO:0016049 1 12

C0015230 GO:0035085 1 12

C0015230 GO:0010810 1 12

C0015230 GO:0019239 3 12

C0015230 GO:0033014 1 12

C0015230 GO:0006684 1 12

C0015230 GO:0016684 1 12

C0015230 GO:0016769 1 12

C0015230 GO:0030894 1 12

C0015230 GO:0009156 2 12

C0015230 GO:0033764 1 12

C0015230 GO:0004004 1 12

C0015230 GO:0042633 1 12

C0015230 GO:0000030 1 12

C0015230 GO:0000132 1 12

C0015230 GO:0007250 1 12

C0015230 GO:0004683 2 12

C0015230 GO:0031647 2 12

C0015230 GO:0030867 1 12

C0015230 GO:0045736 2 12

C0015230 GO:0016469 1 12

C0015230 GO:0002819 1 12

C0015230 GO:0030261 2 12

C0015230 GO:0051000 1 12

C0015230 GO:0016909 1 12

C0015230 GO:0043087 1 12

C0015230 GO:0009127 1 12

C0015230 GO:0006220 2 12

C0015230 GO:0006221 1 12

C0015230 GO:0070603 1 12

C0015230 GO:0042772 1 12

C0015230 GO:0042921 1 12

C0015230 GO:0046784 2 12

C0015230 GO:0046782 1 12

C0015230 GO:0044452 1 12

C0015230 GO:0008385 1 12

C0015230 GO:0022616 1 12

C0015230 GO:0003756 1 12

C0015230 GO:0010939 1 12

C0015230 GO:0046165 1 12

C0015230 GO:0070688 1 12

C0015230 GO:0015298 1 12

C0015230 GO:0035035 1 12

C0015230 GO:0006298 1 12

C0015230 GO:0055088 1 12

C0015230 GO:0055085 1 12

C0015230 GO:0006084 1 12

C0015230 GO:0043601 1 12

C0015230 GO:0006081 2 12

C0015230 GO:0043603 1 12

C0015230 GO:0004601 1 12

C0015230 GO:0070776 1 12

C0015230 GO:0070775 1 12

C0015230 GO:0043370 1 12

C0015230 GO:0055001 1 12

C0015230 GO:0010948 1 12

C0015230 GO:0000347 2 12

C0015230 GO:0000346 2 12

C0015230 GO:0033500 1 12

C0015230 GO:0051262 1 12

C0015230 GO:0008526 1 12

C0015230 GO:0005753 1 12

C0015230 GO:0048531 1 12

C0015230 GO:0034451 1 12

C0015230 GO:0008210 1 12

C0015230 GO:0019104 3 12

C0015230 GO:0005883 1 12

C0015230 GO:0007159 1 12

C0015230 GO:0002440 1 12

C0015230 GO:0000381 1 12

C0015230 GO:0007157 1 12

C0015230 GO:0002444 3 12

C0015230 GO:0002446 3 12

C0015230 GO:0002562 1 12

C0015230 GO:0016580 2 12

C0015230 GO:0016581 2 12

C0015230 GO:0001938 1 12

C0015230 GO:0033344 1 12

C0015230 GO:0004576 1 12

C0015230 GO:0002250 1 12

C0015230 GO:0015030 1 12

C0015230 GO:0004579 1 12

C0015230 GO:0001937 1 12

C0015230 GO:0001936 1 12

C0018801 GO:0060260 1 2

C0018801 GO:0018024 1 2

C0018801 GO:0006297 1 2

C0018801 GO:0000737 1 2

C0018801 GO:0035194 1 2

C0018801 GO:0030201 1 2

C0018801 GO:0000790 1 2

C0018801 GO:0009142 1 2

C0018801 GO:0034508 1 2

C0018801 GO:0043982 1 2

C0018801 GO:0016278 1 2

C0018801 GO:0033683 1 2

C0018801 GO:0031050 1 2

C0018801 GO:0005828 1 2

C0018801 GO:0051148 1 2

C0018801 GO:0005487 1 2

C0018801 GO:0060393 1 2

C0018801 GO:0000209 1 2

C0018801 GO:0005876 1 2

C0018801 GO:0070918 1 2

C0018801 GO:0005548 1 2

C0018801 GO:0005072 1 2

C0018801 GO:0004364 1 2

C0018801 GO:0005852 1 2

C0018801 GO:0032981 1 2

C0018801 GO:0045785 1 2

C0018801 GO:0010257 1 2

C0018801 GO:0055085 1 2

C0018801 GO:0044452 1 2

C0018801 GO:0016246 1 2

C0018801 GO:0042771 1 2

C0018801 GO:0005795 1 2

C0018801 GO:0033108 1 2

C0018801 GO:0030894 1 2

C0018801 GO:0032813 1 2

C0018801 GO:0006706 1 2

C0018801 GO:0030330 1 2

C0018801 GO:0042162 1 2

C0018801 GO:0004385 1 2

C0018801 GO:0000090 1 2

C0018801 GO:0006474 1 2

C0018801 GO:0043410 1 2

C0018801 GO:0000779 1 2

C0018801 GO:0005883 1 2

C0018801 GO:0016811 1 2

C0018801 GO:0005913 1 2

C0018801 GO:0031047 2 2

C0018801 GO:0016279 1 2

C0018801 GO:0001672 1 2

C0018801 GO:0051322 1 2

C0018801 GO:0000777 1 2

C0018801 GO:0043601 1 2

C0018801 GO:0008630 1 2

C0018801 GO:0032365 1 2

C0018801 GO:0015884 1 2

C0018801 GO:0043983 1 2

C0018801 GO:0035196 1 2

C0018801 GO:0031647 1 2

C0018801 GO:0000731 1 2

C0018801 GO:0043984 1 2

C0018801 GO:0010718 1 2

C0018801 GO:0010887 1 2

C0018801 GO:0015012 1 2

C0018801 GO:0043596 1 2

C0018801 GO:0043331 1 2

C0018801 GO:0006164 1 2

C0018801 GO:0016441 1 2

C0018801 GO:0010770 1 2

C0018801 GO:0031985 1 2

C0018801 GO:0006413 1 2

C0018801 GO:0043981 1 2

C0018801 GO:0006004 1 2

C0032285 GO:0005786 1 4

C0032285 GO:0051043 1 4

C0032285 GO:0000245 1 4

C0032285 GO:0022624 1 4

C0032285 GO:0046638 1 4

C0032285 GO:0009218 1 4

C0032285 GO:0050684 1 4

C0032285 GO:0046635 1 4

C0032285 GO:0046634 1 4

C0032285 GO:0007612 1 4

C0032285 GO:0021510 1 4

C0032285 GO:0002706 1 4

C0032285 GO:0043523 1 4

C0032285 GO:0002703 1 4

C0032285 GO:0043525 2 4

C0032285 GO:0051893 1 4

C0032285 GO:0007006 1 4

C0032285 GO:0002709 1 4

C0032285 GO:0007004 1 4

C0032285 GO:0005072 1 4

C0032285 GO:0004364 1 4

C0032285 GO:0005838 1 4

C0032285 GO:0042267 2 4

C0032285 GO:0002263 1 4

C0032285 GO:0043043 1 4

C0032285 GO:0010257 2 4

C0032285 GO:0046637 1 4

C0032285 GO:0030165 1 4

C0032285 GO:0002685 1 4

C0032285 GO:0001909 1 4

C0032285 GO:0006073 1 4

C0032285 GO:0032135 2 4

C0032285 GO:0006268 1 4

C0032285 GO:0004536 1 4

C0032285 GO:0016455 1 4

C0032285 GO:0050795 1 4

C0032285 GO:0035272 1 4

C0032285 GO:0006760 1 4

C0032285 GO:0006563 1 4

C0032285 GO:0005092 1 4

C0032285 GO:0051294 1 4

C0032285 GO:0002824 1 4

C0032285 GO:0004385 2 4

C0032285 GO:0000090 1 4

C0032285 GO:0031109 1 4

C0032285 GO:0002821 1 4

C0032285 GO:0031274 1 4

C0032285 GO:0003841 2 4

C0032285 GO:0016891 2 4

C0032285 GO:0000796 1 4

C0032285 GO:0007172 1 4

C0032285 GO:0016893 1 4

C0032285 GO:0031272 1 4

C0032285 GO:0007076 1 4

C0032285 GO:0000502 1 4

C0032285 GO:0016581 1 4

C0032285 GO:0009071 1 4

C0032285 GO:0030511 1 4

C0032285 GO:0045580 1 4

C0032285 GO:0045793 1 4

C0032285 GO:0006278 1 4

C0032285 GO:0018209 1 4

C0032285 GO:0006271 1 4

C0032285 GO:0005828 1 4

C0032285 GO:0000018 1 4

C0032285 GO:0060393 1 4

C0032285 GO:0010887 1 4

C0032285 GO:0015012 1 4

C0032285 GO:0005663 1 4

C0032285 GO:0043331 1 4

C0032285 GO:0006164 1 4

C0032285 GO:0050920 1 4

C0032285 GO:0016445 1 4

C0032285 GO:0006672 1 4

C0032285 GO:0050921 1 4

C0032285 GO:0060260 1 4

C0032285 GO:0018024 1 4

C0032285 GO:0006297 2 4

C0032285 GO:0002839 1 4

C0032285 GO:0030201 1 4

C0032285 GO:0031647 2 4

C0032285 GO:0002834 1 4

C0032285 GO:0009142 1 4

C0032285 GO:0002836 1 4

C0032285 GO:0003899 1 4

C0032285 GO:0034062 1 4

C0032285 GO:0016278 1 4

C0032285 GO:0016279 1 4

C0032285 GO:0004693 1 4

C0032285 GO:0001763 1 4

C0032285 GO:0016885 1 4

C0032285 GO:0007064 1 4

C0032285 GO:0005487 1 4

C0032285 GO:0030073 1 4

C0032285 GO:0016607 1 4

C0032285 GO:0016289 1 4

C0032285 GO:0005852 3 4

C0032285 GO:0015491 1 4

C0032285 GO:0005851 1 4

C0032285 GO:0042255 1 4

C0032285 GO:0009127 2 4

C0032285 GO:0002687 1 4

C0032285 GO:0002366 1 4

C0032285 GO:0009168 2 4

C0032285 GO:0000339 1 4

C0032285 GO:0043021 1 4

C0032285 GO:0002200 1 4

C0032285 GO:0043022 1 4

C0032285 GO:0032813 1 4

C0032285 GO:0055029 1 4

C0032285 GO:0009156 2 4

C0032285 GO:0048500 1 4

C0032285 GO:0017015 1 4

C0032285 GO:0009152 1 4

C0032285 GO:0030898 1 4

C0032285 GO:0019319 1 4

C0032285 GO:0009260 1 4

C0032285 GO:0006189 2 4

C0032285 GO:0006188 2 4

C0032285 GO:0002711 1 4

C0032285 GO:0009084 1 4

C0032285 GO:0030069 1 4

C0032285 GO:0001672 2 4

C0032285 GO:0005720 1 4

C0032285 GO:0005844 1 4

C0032285 GO:0000132 1 4

C0032285 GO:0031080 2 4

C0032285 GO:0010718 2 4

C0032285 GO:0010717 1 4

C0032285 GO:0002039 1 4

C0032285 GO:0000737 1 4

C0032285 GO:0000731 1 4

C0032285 GO:0015074 1 4

C0032285 GO:0043370 1 4

C0032285 GO:0046519 1 4

C0032285 GO:0033032 1 4

C0032285 GO:0045739 1 4

C0032285 GO:0015665 1 4

C0032285 GO:0000428 1 4

C0032285 GO:0009116 1 4

C0032285 GO:0004526 1 4

C0032285 GO:0030880 1 4

C0032285 GO:0045923 1 4

C0032285 GO:0008143 1 4

C0032285 GO:0030307 1 4

C0032285 GO:0044042 1 4

C0032285 GO:0045178 1 4

C0032285 GO:0030261 1 4

C0032285 GO:0002718 1 4

C0032285 GO:0045582 1 4

C0032285 GO:0031306 1 4

C0032285 GO:0046677 1 4

C0032285 GO:0048024 1 4

C0032285 GO:0031958 1 4

C0032285 GO:0000209 1 4

C0032285 GO:0005876 1 4

C0032285 GO:0006221 1 4

C0032285 GO:0051322 1 4

C0032285 GO:0016706 1 4

C0032285 GO:0002444 1 4

C0032285 GO:0043484 1 4

C0032285 GO:0004521 1 4

C0032285 GO:0033683 1 4

C0032285 GO:0042921 1 4

C0032285 GO:0046784 1 4

C0032285 GO:0002228 2 4

C0032285 GO:0015166 1 4

C0032285 GO:0048568 1 4

C0032285 GO:0045621 1 4

C0032285 GO:0021954 1 4

C0032285 GO:0045622 1 4

C0032285 GO:0002837 1 4

C0032285 GO:0021953 1 4

C0032285 GO:0045749 1 4

C0032285 GO:0019902 1 4

C0032285 GO:0033108 2 4

C0032285 GO:0001937 1 4

C0032285 GO:0043535 1 4

C0032285 GO:0046456 1 4

C0032285 GO:0008329 1 4

C0032285 GO:0008139 1 4

C0032285 GO:0044452 1 4

C0032285 GO:0000217 1 4

C0032285 GO:0015298 1 4

C0032285 GO:0005913 2 4

C0032285 GO:0034061 1 4

C0032285 GO:0001656 1 4

C0032285 GO:0022612 1 4

C0032285 GO:0014065 2 4

C0032285 GO:0035270 1 4

C0032285 GO:0060021 1 4

C0032285 GO:0006978 1 4

C0032285 GO:0055085 1 4

C0032285 GO:0030530 1 4

C0032285 GO:0043473 1 4

C0032285 GO:0002460 1 4

C0032285 GO:0015884 1 4

C0032285 GO:0001953 1 4

C0032285 GO:0007567 1 4

C0032285 GO:0018105 1 4

C0032285 GO:0070776 1 4

C0032285 GO:0070775 1 4

C0032285 GO:0022616 1 4

C0032285 GO:0003727 1 4

C0032285 GO:0006536 1 4

C0032285 GO:0010770 2 4

C0032285 GO:0016018 1 4

C0032285 GO:0032404 2 4

C0032285 GO:0006000 1 4

C0032285 GO:0004177 2 4

C0032285 GO:0032365 1 4

C0032285 GO:0001516 1 4

C0032285 GO:0046364 1 4

C0032285 GO:0048754 1 4

C0032285 GO:0030983 2 4

C0032285 GO:0046040 2 4

C0032285 GO:0000347 1 4

C0032285 GO:0000346 1 4

C0032285 GO:0007270 1 4

C0032285 GO:0006090 1 4

C0032285 GO:0031369 1 4

C0032285 GO:0070822 1 4

C0032285 GO:0008603 1 4

C0032285 GO:0032981 2 4

C0032285 GO:0051087 1 4

C0032285 GO:0008526 1 4

C0032285 GO:0001952 1 4

C0032285 GO:0003730 1 4

C0032285 GO:0045930 1 4

C0032285 GO:0019213 1 4

C0032285 GO:0031333 1 4

C0032285 GO:0006706 1 4

C0032285 GO:0070120 1 4

C0032285 GO:0010810 1 4

C0032285 GO:0002822 1 4

C0032285 GO:0010812 1 4

C0032285 GO:0090100 1 4

C0032285 GO:0004549 1 4

C0032285 GO:0006607 1 4

C0032285 GO:0000779 2 4

C0032285 GO:0005883 1 4

C0032285 GO:0031397 1 4

C0032285 GO:0031047 1 4

C0032285 GO:0045766 1 4

C0032285 GO:0051646 1 4

C0032285 GO:0000381 2 4

C0032285 GO:0005814 1 4

C0032285 GO:0000445 1 4

C0032285 GO:0042772 1 4

C0032285 GO:0002446 1 4

C0032285 GO:0000777 1 4

C0032285 GO:0016580 1 4

C0032285 GO:0051881 1 4

C0032285 GO:0040017 1 4

C0032285 GO:0030532 1 4

C0032285 GO:0019047 1 4

C0032285 GO:0032200 1 4

C0032285 GO:0046457 1 4

C0032285 GO:0001938 1 4

C0032285 GO:0043596 1 4

C0032285 GO:0000790 1 4

C0032285 GO:0002250 1 4

C0032285 GO:0000387 1 4

C0032285 GO:0001936 1 4

C0032285 GO:0006413 2 4

C0032285 GO:0002833 1 4

C0032285 GO:0010833 1 4

C0038454 GO:0060260 1 1

C0038454 GO:0018024 1 1

C0038454 GO:0006297 1 1

C0038454 GO:0000737 1 1

C0038454 GO:0030201 1 1

C0038454 GO:0009142 1 1

C0038454 GO:0016278 1 1

C0038454 GO:0033683 1 1

C0038454 GO:0005828 1 1

C0038454 GO:0005487 1 1

C0038454 GO:0060393 1 1

C0038454 GO:0000209 1 1

C0038454 GO:0005876 1 1

C0038454 GO:0005072 1 1

C0038454 GO:0004364 1 1

C0038454 GO:0005852 1 1

C0038454 GO:0032981 1 1

C0038454 GO:0010257 1 1

C0038454 GO:0055085 1 1

C0038454 GO:0033108 1 1

C0038454 GO:0006706 1 1

C0038454 GO:0004385 1 1

C0038454 GO:0000090 1 1

C0038454 GO:0000779 1 1

C0038454 GO:0005883 1 1

C0038454 GO:0005913 1 1

C0038454 GO:0031047 1 1

C0038454 GO:0016279 1 1

C0038454 GO:0001672 1 1

C0038454 GO:0051322 1 1

C0038454 GO:0000777 1 1

C0038454 GO:0032365 1 1

C0038454 GO:0015884 1 1

C0038454 GO:0031647 1 1

C0038454 GO:0000731 1 1

C0038454 GO:0010718 1 1

C0038454 GO:0015012 1 1

C0038454 GO:0043596 1 1

C0038454 GO:0043331 1 1

C0038454 GO:0010770 1 1

C0038454 GO:0006413 1 1

C0039070 GO:0006776 1 6

C0039070 GO:0005786 1 6

C0039070 GO:0000245 1 6

C0039070 GO:0022624 1 6

C0039070 GO:0046638 1 6

C0039070 GO:0046637 1 6

C0039070 GO:0050684 1 6

C0039070 GO:0046635 1 6

C0039070 GO:0032387 2 6

C0039070 GO:0007612 1 6

C0039070 GO:0021510 2 6

C0039070 GO:0009451 1 6

C0039070 GO:0002706 1 6

C0039070 GO:0043523 1 6

C0039070 GO:0002703 1 6

C0039070 GO:0043525 1 6

C0039070 GO:0051893 1 6

C0039070 GO:0032182 1 6

C0039070 GO:0008376 1 6

C0039070 GO:0042559 1 6

C0039070 GO:0007006 1 6

C0039070 GO:0002709 1 6

C0039070 GO:0007004 1 6

C0039070 GO:0005355 1 6

C0039070 GO:0005072 1 6

C0039070 GO:0004364 1 6

C0039070 GO:0005838 1 6

C0039070 GO:0042267 2 6

C0039070 GO:0002263 1 6

C0039070 GO:0043043 1 6

C0039070 GO:0010257 2 6

C0039070 GO:0018210 1 6

C0039070 GO:0060021 1 6

C0039070 GO:0002822 1 6

C0039070 GO:0045667 1 6

C0039070 GO:0007080 1 6

C0039070 GO:0001909 1 6

C0039070 GO:0006073 1 6

C0039070 GO:0006733 1 6

C0039070 GO:0016101 1 6

C0039070 GO:0032135 2 6

C0039070 GO:0045669 1 6

C0039070 GO:0033032 1 6

C0039070 GO:0050795 1 6

C0039070 GO:0048512 1 6

C0039070 GO:0019903 1 6

C0039070 GO:0006760 1 6

C0039070 GO:0006563 1 6

C0039070 GO:0051119 1 6

C0039070 GO:0005092 1 6

C0039070 GO:0051294 1 6

C0039070 GO:0002824 1 6

C0039070 GO:0004385 2 6

C0039070 GO:0000090 1 6

C0039070 GO:0031109 1 6

C0039070 GO:0002821 1 6

C0039070 GO:0035270 1 6

C0039070 GO:0003841 1 6

C0039070 GO:0016891 1 6

C0039070 GO:0000796 1 6

C0039070 GO:0007172 1 6

C0039070 GO:0045923 1 6

C0039070 GO:0007076 1 6

C0039070 GO:0000502 1 6

C0039070 GO:0016581 1 6

C0039070 GO:0042992 1 6

C0039070 GO:0009071 1 6

C0039070 GO:0030511 1 6

C0039070 GO:0045580 1 6

C0039070 GO:0045793 1 6

C0039070 GO:0006278 1 6

C0039070 GO:0018209 2 6

C0039070 GO:0000159 1 6

C0039070 GO:0030509 1 6

C0039070 GO:0006271 1 6

C0039070 GO:0005828 1 6

C0039070 GO:0000018 1 6

C0039070 GO:0060393 1 6

C0039070 GO:0010887 1 6

C0039070 GO:0015012 1 6

C0039070 GO:0005663 1 6

C0039070 GO:0043130 1 6

C0039070 GO:0043331 1 6

C0039070 GO:0016291 1 6

C0039070 GO:0006164 1 6

C0039070 GO:0051087 2 6

C0039070 GO:0016445 1 6

C0039070 GO:0006672 1 6

C0039070 GO:0048256 1 6

C0039070 GO:0060260 1 6

C0039070 GO:0018024 1 6

C0039070 GO:0006297 2 6

C0039070 GO:0002839 1 6

C0039070 GO:0030201 1 6

C0039070 GO:0031647 2 6

C0039070 GO:0002834 1 6

C0039070 GO:0009142 1 6

C0039070 GO:0002836 1 6

C0039070 GO:0003899 1 6

C0039070 GO:0034062 1 6

C0039070 GO:0016278 1 6

C0039070 GO:0016279 1 6

C0039070 GO:0004693 1 6

C0039070 GO:0042745 1 6

C0039070 GO:0001763 1 6

C0039070 GO:0016885 1 6

C0039070 GO:0032947 1 6

C0039070 GO:0001516 1 6

C0039070 GO:0005487 2 6

C0039070 GO:0001950 1 6

C0039070 GO:0042749 1 6

C0039070 GO:0016607 1 6

C0039070 GO:0016289 1 6

C0039070 GO:0005852 3 6

C0039070 GO:0015491 1 6

C0039070 GO:0005851 1 6

C0039070 GO:0043370 1 6

C0039070 GO:0042255 1 6

C0039070 GO:0009127 2 6

C0039070 GO:0015149 1 6

C0039070 GO:0016574 1 6

C0039070 GO:0001964 1 6

C0039070 GO:0002366 1 6

C0039070 GO:0009168 2 6

C0039070 GO:0016763 1 6

C0039070 GO:0043021 1 6

C0039070 GO:0090100 1 6

C0039070 GO:0002200 1 6

C0039070 GO:0043022 1 6

C0039070 GO:0015145 1 6

C0039070 GO:0032813 1 6

C0039070 GO:0007622 1 6

C0039070 GO:0016605 1 6

C0039070 GO:0055029 1 6

C0039070 GO:0009156 2 6

C0039070 GO:0048500 1 6

C0039070 GO:0017015 1 6

C0039070 GO:0009152 2 6

C0039070 GO:0008013 1 6

C0039070 GO:0019319 1 6

C0039070 GO:0009260 2 6

C0039070 GO:0007052 1 6

C0039070 GO:0009262 1 6

C0039070 GO:0006189 2 6

C0039070 GO:0006188 2 6

C0039070 GO:0002711 1 6

C0039070 GO:0009084 1 6

C0039070 GO:0042306 1 6

C0039070 GO:0030069 1 6

C0039070 GO:0001672 2 6

C0039070 GO:0016018 1 6

C0039070 GO:0005720 1 6

C0039070 GO:0005844 1 6

C0039070 GO:0008603 1 6

C0039070 GO:0000132 1 6

C0039070 GO:0031080 2 6

C0039070 GO:0010718 1 6

C0039070 GO:0004536 1 6

C0039070 GO:0002039 1 6

C0039070 GO:0001889 1 6

C0039070 GO:0000737 1 6

C0039070 GO:0000731 1 6

C0039070 GO:0015074 1 6

C0039070 GO:0046823 1 6

C0039070 GO:0046519 1 6

C0039070 GO:0045739 1 6

C0039070 GO:0006359 1 6

C0039070 GO:0000428 1 6

C0039070 GO:0009264 1 6

C0039070 GO:0021537 1 6

C0039070 GO:0004526 1 6

C0039070 GO:0016790 1 6

C0039070 GO:0030159 1 6

C0039070 GO:0050921 1 6

C0039070 GO:0008143 1 6

C0039070 GO:0030307 1 6

C0039070 GO:0042308 1 6

C0039070 GO:0044042 1 6

C0039070 GO:0046677 1 6

C0039070 GO:0005545 1 6

C0039070 GO:0030261 1 6

C0039070 GO:0045582 1 6

C0039070 GO:0031306 1 6

C0039070 GO:0007043 1 6

C0039070 GO:0048024 1 6

C0039070 GO:0031958 1 6

C0039070 GO:0004691 1 6

C0039070 GO:0004690 1 6

C0039070 GO:0000209 1 6

C0039070 GO:0005876 1 6

C0039070 GO:0009394 1 6

C0039070 GO:0000445 1 6

C0039070 GO:0045426 1 6

C0039070 GO:0015851 1 6

C0039070 GO:0051322 1 6

C0039070 GO:0016706 1 6

C0039070 GO:0002444 1 6

C0039070 GO:0000339 1 6

C0039070 GO:0043484 1 6

C0039070 GO:0033683 1 6

C0039070 GO:0042921 1 6

C0039070 GO:0042375 1 6

C0039070 GO:0046784 1 6

C0039070 GO:0002228 2 6

C0039070 GO:0048568 1 6

C0039070 GO:0045621 1 6

C0039070 GO:0021954 2 6

C0039070 GO:0030431 1 6

C0039070 GO:0006744 1 6

C0039070 GO:0006743 1 6

C0039070 GO:0002837 1 6

C0039070 GO:0021953 2 6

C0039070 GO:0045749 1 6

C0039070 GO:0019902 1 6

C0039070 GO:0033108 2 6

C0039070 GO:0042169 1 6

C0039070 GO:0008287 1 6

C0039070 GO:0043535 1 6

C0039070 GO:0046456 1 6

C0039070 GO:0008139 1 6

C0039070 GO:0006721 1 6

C0039070 GO:0002687 1 6

C0039070 GO:0044452 1 6

C0039070 GO:0000217 1 6

C0039070 GO:0015298 1 6

C0039070 GO:0004860 1 6

C0039070 GO:0005913 1 6

C0039070 GO:0034061 1 6

C0039070 GO:0001656 2 6

C0039070 GO:0031397 1 6

C0039070 GO:0051310 1 6

C0039070 GO:0005086 1 6

C0039070 GO:0006978 1 6

C0039070 GO:0055085 1 6

C0039070 GO:0030530 1 6

C0039070 GO:0043473 1 6

C0039070 GO:0045622 1 6

C0039070 GO:0002460 1 6

C0039070 GO:0015884 1 6

C0039070 GO:0001953 1 6

C0039070 GO:0018105 1 6

C0039070 GO:0070776 1 6

C0039070 GO:0070775 1 6

C0039070 GO:0022616 1 6

C0039070 GO:0005742 1 6

C0039070 GO:0003727 1 6

C0039070 GO:0006536 1 6

C0039070 GO:0006221 1 6

C0039070 GO:0009116 1 6

C0039070 GO:0010770 1 6

C0039070 GO:0009108 1 6

C0039070 GO:0032404 2 6

C0039070 GO:0006000 1 6

C0039070 GO:0046496 1 6

C0039070 GO:0004177 2 6

C0039070 GO:0016331 1 6

C0039070 GO:0032365 1 6

C0039070 GO:0002685 1 6

C0039070 GO:0031369 1 6

C0039070 GO:0022410 1 6

C0039070 GO:0046364 1 6

C0039070 GO:0034508 1 6

C0039070 GO:0048754 1 6

C0039070 GO:0030983 2 6

C0039070 GO:0046040 2 6

C0039070 GO:0009109 1 6

C0039070 GO:0000347 1 6

C0039070 GO:0000346 1 6

C0039070 GO:0014065 1 6

C0039070 GO:0043550 1 6

C0039070 GO:0001523 1 6

C0039070 GO:0004653 1 6

C0039070 GO:0070822 1 6

C0039070 GO:0045187 1 6

C0039070 GO:0016846 1 6

C0039070 GO:0032981 2 6

C0039070 GO:0005814 1 6

C0039070 GO:0008526 1 6

C0039070 GO:0001952 1 6

C0039070 GO:0001936 1 6

C0039070 GO:0003730 1 6

C0039070 GO:0006400 1 6

C0039070 GO:0030900 1 6

C0039070 GO:0045814 1 6

C0039070 GO:0030165 1 6

C0039070 GO:0002250 1 6

C0039070 GO:0046634 1 6

C0039070 GO:0045930 1 6

C0039070 GO:0031333 1 6

C0039070 GO:0006706 1 6

C0039070 GO:0070120 2 6

C0039070 GO:0010810 1 6

C0039070 GO:0006769 1 6

C0039070 GO:0010812 1 6

C0039070 GO:0048593 1 6

C0039070 GO:0004549 1 6

C0039070 GO:0050920 1 6

C0039070 GO:0006607 2 6

C0039070 GO:0000779 3 6

C0039070 GO:0005883 2 6

C0039070 GO:0031047 2 6

C0039070 GO:0045766 1 6

C0039070 GO:0051646 1 6

C0039070 GO:0000381 2 6

C0039070 GO:0002443 1 6

C0039070 GO:0042772 1 6

C0039070 GO:0002446 1 6

C0039070 GO:0000777 1 6

C0039070 GO:0030880 1 6

C0039070 GO:0016580 1 6

C0039070 GO:0051881 1 6

C0039070 GO:0040017 1 6

C0039070 GO:0030532 1 6

C0039070 GO:0019047 1 6

C0039070 GO:0032200 1 6

C0039070 GO:0046457 1 6

C0039070 GO:0001938 1 6

C0039070 GO:0043596 1 6

C0039070 GO:0000790 1 6

C0039070 GO:0001937 2 6

C0039070 GO:0000387 1 6

C0039070 GO:0006390 1 6

C0039070 GO:0019362 1 6

C0039070 GO:0006413 2 6

C0039070 GO:0002833 1 6

C0039070 GO:0010833 1 6

C0042571 GO:0051043 1 9

C0042571 GO:0022624 1 9

C0042571 GO:0043954 1 9

C0042571 GO:0002706 1 9

C0042571 GO:0002709 1 9

C0042571 GO:0032925 1 9

C0042571 GO:0015149 1 9

C0042571 GO:0005838 1 9

C0042571 GO:0005834 1 9

C0042571 GO:0004653 1 9

C0042571 GO:0001964 1 9

C0042571 GO:0018210 1 9

C0042571 GO:0007080 1 9

C0042571 GO:0046027 1 9

C0042571 GO:0016101 2 9

C0042571 GO:0016455 1 9

C0042571 GO:0030595 1 9

C0042571 GO:0010390 1 9

C0042571 GO:0030593 1 9

C0042571 GO:0002824 1 9

C0042571 GO:0002822 1 9

C0042571 GO:0002821 1 9

C0042571 GO:0003841 2 9

C0042571 GO:0035272 2 9

C0042571 GO:0050770 1 9

C0042571 GO:0006278 1 9

C0042571 GO:0032365 1 9

C0042571 GO:0015145 1 9

C0042571 GO:0006271 1 9

C0042571 GO:0010887 1 9

C0042571 GO:0010885 1 9

C0042571 GO:0043331 1 9

C0042571 GO:0006379 1 9

C0042571 GO:0010889 1 9

C0042571 GO:0010888 1 9

C0042571 GO:0060260 2 9

C0042571 GO:0032412 1 9

C0042571 GO:0001953 1 9

C0042571 GO:0005487 2 9

C0042571 GO:0050994 1 9

C0042571 GO:0030073 1 9

C0042571 GO:0016607 2 9

C0042571 GO:0016605 1 9

C0042571 GO:0042551 2 9

C0042571 GO:0042559 1 9

C0042571 GO:0070742 1 9

C0042571 GO:0002687 1 9

C0042571 GO:0002685 1 9

C0042571 GO:0009168 1 9

C0042571 GO:0002688 1 9

C0042571 GO:0007622 1 9

C0042571 GO:0009304 1 9

C0042571 GO:0032570 1 9

C0042571 GO:0005779 1 9

C0042571 GO:0009260 3 9

C0042571 GO:0007052 1 9

C0042571 GO:0009262 1 9

C0042571 GO:0009264 1 9

C0042571 GO:0009267 1 9

C0042571 GO:0006303 1 9

C0042571 GO:0031080 1 9

C0042571 GO:0004532 1 9

C0042571 GO:0031231 1 9

C0042571 GO:0015074 1 9

C0042571 GO:0006029 1 9

C0042571 GO:0006752 1 9

C0042571 GO:0008360 1 9

C0042571 GO:0018212 1 9

C0042571 GO:0033209 2 9

C0042571 GO:0005227 1 9

C0042571 GO:0019047 1 9

C0042571 GO:0004693 1 9

C0042571 GO:0004691 1 9

C0042571 GO:0004690 1 9

C0042571 GO:0004697 1 9

C0042571 GO:0005876 1 9

C0042571 GO:0009394 1 9

C0042571 GO:0042326 1 9

C0042571 GO:0043484 1 9

C0042571 GO:0045178 1 9

C0042571 GO:0002228 2 9

C0042571 GO:0002224 1 9

C0042571 GO:0002221 1 9

C0042571 GO:0045749 1 9

C0042571 GO:0016944 1 9

C0042571 GO:0008287 1 9

C0042571 GO:0008329 1 9

C0042571 GO:0043550 1 9

C0042571 GO:0005913 2 9

C0042571 GO:0001656 1 9

C0042571 GO:0046504 1 9

C0042571 GO:0010718 3 9

C0042571 GO:0004190 1 9

C0042571 GO:0018107 1 9

C0042571 GO:0018105 1 9

C0042571 GO:0018108 1 9

C0042571 GO:0045884 1 9

C0042571 GO:0001889 1 9

C0042571 GO:0042594 1 9

C0042571 GO:0015184 1 9

C0042571 GO:0007270 1 9

C0042571 GO:0008601 1 9

C0042571 GO:0006090 1 9

C0042571 GO:0006094 1 9

C0042571 GO:0003730 1 9

C0042571 GO:0045814 1 9

C0042571 GO:0002690 1 9

C0042571 GO:0019213 1 9

C0042571 GO:0006706 1 9

C0042571 GO:0070120 2 9

C0042571 GO:0048593 2 9

C0042571 GO:0006607 2 9

C0042571 GO:0031047 2 9

C0042571 GO:0000445 1 9

C0042571 GO:0042509 1 9

C0042571 GO:0010595 1 9

C0042571 GO:0030530 1 9

C0042571 GO:0040017 1 9

C0042571 GO:0043536 1 9

C0042571 GO:0009913 1 9

C0042571 GO:0043535 1 9

C0042571 GO:0050996 1 9

C0042571 GO:0045187 1 9

C0042571 GO:0006413 2 9

C0042571 GO:0048520 1 9

C0042571 GO:0019362 1 9

C0042571 GO:0009451 1 9

C0042571 GO:0043525 1 9

C0042571 GO:0031345 1 9

C0042571 GO:0007006 2 9

C0042571 GO:0007004 1 9

C0042571 GO:0004364 1 9

C0042571 GO:0045785 1 9

C0042571 GO:0010257 2 9

C0042571 GO:0045667 1 9

C0042571 GO:0032135 1 9

C0042571 GO:0045669 1 9

C0042571 GO:0051119 1 9

C0042571 GO:0004385 2 9

C0042571 GO:0016893 2 9

C0042571 GO:0000790 1 9

C0042571 GO:0016891 3 9

C0042571 GO:0000796 1 9

C0042571 GO:0009074 2 9

C0042571 GO:0051702 1 9

C0042571 GO:0042992 1 9

C0042571 GO:0042993 1 9

C0042571 GO:0090079 1 9

C0042571 GO:0018209 2 9

C0042571 GO:0000159 1 9

C0042571 GO:0005828 1 9

C0042571 GO:0000018 1 9

C0042571 GO:0016291 1 9

C0042571 GO:0016445 1 9

C0042571 GO:0018024 1 9

C0042571 GO:0006297 2 9

C0042571 GO:0002839 1 9

C0042571 GO:0015851 1 9

C0042571 GO:0002834 1 9

C0042571 GO:0002837 1 9

C0042571 GO:0002836 1 9

C0042571 GO:0006298 1 9

C0042571 GO:0034061 1 9

C0042571 GO:0046321 1 9

C0042571 GO:0050820 2 9

C0042571 GO:0001763 1 9

C0042571 GO:0046496 1 9

C0042571 GO:0050829 1 9

C0042571 GO:0051028 1 9

C0042571 GO:0045088 1 9

C0042571 GO:0006563 1 9

C0042571 GO:0000779 2 9

C0042571 GO:0010883 1 9

C0042571 GO:0016363 1 9

C0042571 GO:0001948 1 9

C0042571 GO:0032813 2 9

C0042571 GO:0009880 1 9

C0042571 GO:0000339 2 9

C0042571 GO:0019319 1 9

C0042571 GO:0050795 1 9

C0042571 GO:0030069 1 9

C0042571 GO:0005720 1 9

C0042571 GO:0032369 1 9

C0042571 GO:0010717 2 9

C0042571 GO:0042267 2 9

C0042571 GO:0046823 1 9

C0042571 GO:0015385 1 9

C0042571 GO:0021537 1 9

C0042571 GO:0016796 1 9

C0042571 GO:0016790 1 9

C0042571 GO:0006733 1 9

C0042571 GO:0051646 1 9

C0042571 GO:0031306 1 9

C0042571 GO:0007043 1 9

C0042571 GO:0032963 1 9

C0042571 GO:0032886 1 9

C0042571 GO:0000209 1 9

C0042571 GO:0050766 1 9

C0042571 GO:0016709 1 9

C0042571 GO:0004527 1 9

C0042571 GO:0004526 1 9

C0042571 GO:0004521 1 9

C0042571 GO:0010769 2 9

C0042571 GO:0015166 1 9

C0042571 GO:0009925 1 9

C0042571 GO:0021954 3 9

C0042571 GO:0006744 1 9

C0042571 GO:0006743 1 9

C0042571 GO:0021953 3 9

C0042571 GO:0032404 1 9

C0042571 GO:0005355 1 9

C0042571 GO:0042169 1 9

C0042571 GO:0008139 1 9

C0042571 GO:0042162 1 9

C0042571 GO:0014065 1 9

C0042571 GO:0010596 1 9

C0042571 GO:0048730 1 9

C0042571 GO:0009084 1 9

C0042571 GO:0005451 1 9

C0042571 GO:0004177 1 9

C0042571 GO:0016331 1 9

C0042571 GO:0050868 1 9

C0042571 GO:0033135 2 9

C0042571 GO:0030983 1 9

C0042571 GO:0009108 1 9

C0042571 GO:0009109 1 9

C0042571 GO:0001523 2 9

C0042571 GO:0006376 1 9

C0042571 GO:0016846 2 9

C0042571 GO:0016840 1 9

C0042571 GO:0033628 1 9

C0042571 GO:0042787 1 9

C0042571 GO:0016246 1 9

C0042571 GO:0045931 1 9

C0042571 GO:0045930 1 9

C0042571 GO:0030183 1 9

C0042571 GO:0010812 1 9

C0042571 GO:0030431 1 9

C0042571 GO:0035295 1 9

C0042571 GO:0051881 2 9

C0042571 GO:0031998 1 9

C0042571 GO:0032200 1 9

C0042571 GO:0006390 2 9

C0042571 GO:0044403 1 9

C0042571 GO:0010833 1 9

C0042571 GO:0006776 1 9

C0042571 GO:0000245 2 9

C0042571 GO:0009218 1 9

C0042571 GO:0032387 2 9

C0042571 GO:0021510 1 9

C0042571 GO:0005788 1 9

C0042571 GO:0006476 1 9

C0042571 GO:0030509 1 9

C0042571 GO:0030506 1 9

C0042571 GO:0005072 1 9

C0042571 GO:0016627 1 9

C0042571 GO:0070001 1 9

C0042571 GO:0048512 1 9

C0042571 GO:0010720 1 9

C0042571 GO:0051287 1 9

C0042571 GO:0032655 1 9

C0042571 GO:0000099 1 9

C0042571 GO:0005092 1 9

C0042571 GO:0005159 1 9

C0042571 GO:0000090 1 9

C0042571 GO:0043537 1 9

C0042571 GO:0031274 1 9

C0042571 GO:0019903 1 9

C0042571 GO:0019902 1 9

C0042571 GO:0007172 2 9

C0042571 GO:0031272 1 9

C0042571 GO:0007076 1 9

C0042571 GO:0045793 1 9

C0042571 GO:0030618 1 9

C0042571 GO:0060393 1 9

C0042571 GO:0015012 2 9

C0042571 GO:0070507 1 9

C0042571 GO:0030201 2 9

C0042571 GO:0009142 1 9

C0042571 GO:0070567 1 9

C0042571 GO:0016278 1 9

C0042571 GO:0016279 1 9

C0042571 GO:0016885 1 9

C0042571 GO:0016289 1 9

C0042571 GO:0005852 2 9

C0042571 GO:0000146 1 9

C0042571 GO:0005851 1 9

C0042571 GO:0043548 1 9

C0042571 GO:0031674 1 9

C0042571 GO:0045055 1 9

C0042571 GO:0016575 1 9

C0042571 GO:0016574 1 9

C0042571 GO:0005522 1 9

C0042571 GO:0002200 1 9

C0042571 GO:0045727 1 9

C0042571 GO:0035251 1 9

C0042571 GO:0009303 1 9

C0042571 GO:0006189 1 9

C0042571 GO:0006188 1 9

C0042571 GO:0034713 1 9

C0042571 GO:0042308 1 9

C0042571 GO:0042307 1 9

C0042571 GO:0042306 2 9

C0042571 GO:0030279 1 9

C0042571 GO:0001672 2 9

C0042571 GO:0000737 1 9

C0042571 GO:0002758 1 9

C0042571 GO:0000731 1 9

C0042571 GO:0006359 1 9

C0042571 GO:0030159 1 9

C0042571 GO:0005545 1 9

C0042571 GO:0045426 1 9

C0042571 GO:0030018 1 9

C0042571 GO:0034399 1 9

C0042571 GO:0051322 1 9

C0042571 GO:0050818 1 9

C0042571 GO:0051087 1 9

C0042571 GO:0042375 1 9

C0042571 GO:0046364 1 9

C0042571 GO:0006721 2 9

C0042571 GO:0009247 1 9

C0042571 GO:0004860 1 9

C0042571 GO:0004864 1 9

C0042571 GO:0030121 1 9

C0042571 GO:0030656 1 9

C0042571 GO:0050771 1 9

C0042571 GO:0050715 1 9

C0042571 GO:0015884 1 9

C0042571 GO:0032182 1 9

C0042571 GO:0001950 1 9

C0042571 GO:0005742 1 9

C0042571 GO:0032479 1 9

C0042571 GO:0010770 3 9

C0042571 GO:0006000 1 9

C0042571 GO:0001818 1 9

C0042571 GO:0042054 1 9

C0042571 GO:0034508 1 9

C0042571 GO:0048754 1 9

C0042571 GO:0042312 1 9

C0042571 GO:0032981 2 9

C0042571 GO:0046040 1 9

C0042571 GO:0046425 1 9

C0042571 GO:0006911 1 9

C0042571 GO:0002711 1 9

C0042571 GO:0002718 1 9

C0042571 GO:0000777 1 9

C0042571 GO:0000178 1 9

C0042571 GO:0000175 1 9

C0042571 GO:0043596 1 9

C0042571 GO:0030900 1 9

C0042571 GO:0030902 1 9

C0042571 GO:0016896 1 9

C0042571 GO:0050684 1 9

C0042571 GO:0008276 1 9

C0042571 GO:0050920 1 9

C0042571 GO:0050921 1 9

C0042571 GO:0034765 1 9

C0042571 GO:0008376 1 9

C0042571 GO:0008278 1 9

C0042571 GO:0034762 1 9

C0042571 GO:0006983 1 9

C0042571 GO:0043043 1 9

C0042571 GO:0009071 1 9

C0042571 GO:0044419 1 9

C0042571 GO:0006266 1 9

C0042571 GO:0010896 1 9

C0042571 GO:0010898 1 9

C0042571 GO:0006268 1 9

C0042571 GO:0006760 1 9

C0042571 GO:0019722 1 9

C0042571 GO:0030532 1 9

C0042571 GO:0006769 1 9

C0042571 GO:0030166 1 9

C0042571 GO:0010975 1 9

C0042571 GO:0000502 1 9

C0042571 GO:0005663 1 9

C0042571 GO:0043130 1 9

C0042571 GO:0006164 1 9

C0042571 GO:0030193 1 9

C0042571 GO:0030194 2 9

C0042571 GO:0005086 1 9

C0042571 GO:0042745 1 9

C0042571 GO:0032947 1 9

C0042571 GO:0007064 1 9

C0042571 GO:0042749 1 9

C0042571 GO:0004549 1 9

C0042571 GO:0000381 1 9

C0042571 GO:0035085 1 9

C0042571 GO:0070513 1 9

C0042571 GO:0019239 2 9

C0042571 GO:0006687 1 9

C0042571 GO:0006684 1 9

C0042571 GO:0016763 1 9

C0042571 GO:0006688 1 9

C0042571 GO:0002040 1 9

C0042571 GO:0009156 2 9

C0042571 GO:0009152 2 9

C0042571 GO:0008013 1 9

C0042571 GO:0048256 1 9

C0042571 GO:0005844 1 9

C0042571 GO:0004683 1 9

C0042571 GO:0031647 1 9

C0042571 GO:0048469 1 9

C0042571 GO:0002218 1 9

C0042571 GO:0045739 1 9

C0042571 GO:0015665 1 9

C0042571 GO:0022410 1 9

C0042571 GO:0030307 1 9

C0042571 GO:0002819 1 9

C0042571 GO:0033683 1 9

C0042571 GO:0030261 1 9

C0042571 GO:0048024 1 9

C0042571 GO:0008629 1 9

C0042571 GO:0043087 1 9

C0042571 GO:0009127 1 9

C0042571 GO:0046784 1 9

C0042571 GO:0022612 1 9

C0042571 GO:0022616 2 9

C0042571 GO:0032273 1 9

C0042571 GO:0010832 1 9

C0042571 GO:0002833 1 9

C0042571 GO:0010830 1 9

C0042571 GO:0051310 1 9

C0042571 GO:0008206 1 9

C0042571 GO:0055085 1 9

C0042571 GO:0008630 2 9

C0042571 GO:0007567 1 9

C0042571 GO:0030665 1 9

C0042571 GO:0003727 2 9

C0042571 GO:0006536 1 9

C0042571 GO:0048009 1 9

C0042571 GO:0033108 2 9

C0042571 GO:0000347 1 9

C0042571 GO:0000346 1 9

C0042571 GO:0030134 2 9

C0042571 GO:0042516 1 9

C0042571 GO:0042517 1 9

C0042571 GO:0006400 1 9

C0042571 GO:0006406 1 9

C0042571 GO:0000387 1 9

C0042571 GO:0005881 1 9

C0042571 GO:0042974 1 9

C0042571 GO:0005883 2 9

C0042571 GO:0015030 1 9

C0042571 GO:0002443 1 9

C0042571 GO:0002444 1 9

C0042571 GO:0002446 1 9

C0042571 GO:0042771 1 9

C0042571 GO:0030898 1 9

C0042571 GO:0030658 1 9

C0042571 GO:0042475 1 9

C0042571 GO:0001654 1 9

C0042571 GO:0001933 1 9

C0042571 GO:0001937 1 9

C0086543 GO:0060260 1 1

C0086543 GO:0018024 1 1

C0086543 GO:0006297 1 1

C0086543 GO:0000737 1 1

C0086543 GO:0030201 1 1

C0086543 GO:0009142 1 1

C0086543 GO:0016278 1 1

C0086543 GO:0033683 1 1

C0086543 GO:0005828 1 1

C0086543 GO:0005487 1 1

C0086543 GO:0060393 1 1

C0086543 GO:0000209 1 1

C0086543 GO:0005876 1 1

C0086543 GO:0005072 1 1

C0086543 GO:0004364 1 1

C0086543 GO:0005852 1 1

C0086543 GO:0032981 1 1

C0086543 GO:0010257 1 1

C0086543 GO:0055085 1 1

C0086543 GO:0033108 1 1

C0086543 GO:0006706 1 1

C0086543 GO:0004385 1 1

C0086543 GO:0000090 1 1

C0086543 GO:0000779 1 1

C0086543 GO:0005883 1 1

C0086543 GO:0005913 1 1

C0086543 GO:0031047 1 1

C0086543 GO:0016279 1 1

C0086543 GO:0001672 1 1

C0086543 GO:0051322 1 1

C0086543 GO:0000777 1 1

C0086543 GO:0032365 1 1

C0086543 GO:0015884 1 1

C0086543 GO:0031647 1 1

C0086543 GO:0000731 1 1

C0086543 GO:0010718 1 1

C0086543 GO:0015012 1 1

C0086543 GO:0043596 1 1

C0086543 GO:0043331 1 1

C0086543 GO:0010770 1 1

C0086543 GO:0006413 1 1

C0003862 GO:0032432 1 14

C0003862 GO:0051043 1 14

C0003862 GO:0010676 1 14

C0003862 GO:0022624 1 14

C0003862 GO:0048066 1 14

C0003862 GO:0002706 2 14

C0003862 GO:0002705 1 14

C0003862 GO:0002703 2 14

C0003862 GO:0034199 1 14

C0003862 GO:0009161 1 14

C0003862 GO:0007044 1 14

C0003862 GO:0070934 1 14

C0003862 GO:0070937 1 14

C0003862 GO:0043021 2 14

C0003862 GO:0002709 1 14

C0003862 GO:0002708 1 14

C0003862 GO:0005838 1 14

C0003862 GO:0043206 1 14

C0003862 GO:0005834 1 14

C0003862 GO:0003158 1 14

C0003862 GO:0051495 1 14

C0003862 GO:0060249 1 14

C0003862 GO:0004653 1 14

C0003862 GO:0001964 1 14

C0003862 GO:0018210 1 14

C0003862 GO:0030914 1 14

C0003862 GO:0007080 2 14

C0003862 GO:0004712 2 14

C0003862 GO:0016514 1 14

C0003862 GO:0016101 1 14

C0003862 GO:0030125 1 14

C0003862 GO:0016455 1 14

C0003862 GO:0006284 1 14

C0003862 GO:0006282 1 14

C0003862 GO:0051294 1 14

C0003862 GO:0002824 1 14

C0003862 GO:0002822 1 14

C0003862 GO:0031109 1 14

C0003862 GO:0002821 1 14

C0003862 GO:0035270 1 14

C0003862 GO:0003841 2 14

C0003862 GO:0035272 1 14

C0003862 GO:0005788 1 14

C0003862 GO:0001754 1 14

C0003862 GO:0006779 1 14

C0003862 GO:0000718 2 14

C0003862 GO:0006278 2 14

C0003862 GO:0031050 2 14

C0003862 GO:0006271 2 14

C0003862 GO:0006270 2 14

C0003862 GO:0080010 1 14

C0003862 GO:0010887 2 14

C0003862 GO:0042698 1 14

C0003862 GO:0010885 1 14

C0003862 GO:0006376 1 14

C0003862 GO:0070776 1 14

C0003862 GO:0006378 1 14

C0003862 GO:0006379 1 14

C0003862 GO:0021537 2 14

C0003862 GO:0018149 1 14

C0003862 GO:0031958 2 14

C0003862 GO:0048568 1 14

C0003862 GO:0006595 1 14

C0003862 GO:0030175 1 14

C0003862 GO:0010907 1 14

C0003862 GO:0030170 1 14

C0003862 GO:0008186 1 14

C0003862 GO:0006626 1 14

C0003862 GO:0005487 1 14

C0003862 GO:0008535 1 14

C0003862 GO:0016944 1 14

C0003862 GO:0030073 1 14

C0003862 GO:0016607 2 14

C0003862 GO:0016605 1 14

C0003862 GO:0048261 1 14

C0003862 GO:0015491 1 14

C0003862 GO:0042551 2 14

C0003862 GO:0042255 1 14

C0003862 GO:0008641 1 14

C0003862 GO:0042558 1 14

C0003862 GO:0042559 1 14

C0003862 GO:0070742 2 14

C0003862 GO:0002687 2 14

C0003862 GO:0002685 1 14

C0003862 GO:0009168 3 14

C0003862 GO:0050792 1 14

C0003862 GO:0045913 1 14

C0003862 GO:0043022 3 14

C0003862 GO:0043027 1 14

C0003862 GO:0006303 2 14

C0003862 GO:0019883 1 14

C0003862 GO:0016783 1 14

C0003862 GO:0009306 1 14

C0003862 GO:0031256 1 14

C0003862 GO:0009304 2 14

C0003862 GO:0032479 2 14

C0003862 GO:0048709 1 14

C0003862 GO:0009303 1 14

C0003862 GO:0033764 1 14

C0003862 GO:0043410 1 14

C0003862 GO:0009260 5 14

C0003862 GO:0032481 1 14

C0003862 GO:0009262 3 14

C0003862 GO:0009264 3 14

C0003862 GO:0009266 1 14

C0003862 GO:0032890 1 14

C0003862 GO:0000002 1 14

C0003862 GO:0004843 1 14

C0003862 GO:0044042 1 14

C0003862 GO:0030675 2 14

C0003862 GO:0031080 2 14

C0003862 GO:0004536 1 14

C0003862 GO:0002039 1 14

C0003862 GO:0015781 1 14

C0003862 GO:0015780 1 14

C0003862 GO:0004532 1 14

C0003862 GO:0015074 1 14

C0003862 GO:0048365 2 14

C0003862 GO:0010553 1 14

C0003862 GO:0015175 1 14

C0003862 GO:0015172 1 14

C0003862 GO:0008023 1 14

C0003862 GO:0030880 2 14

C0003862 GO:0008143 2 14

C0003862 GO:0033209 2 14

C0003862 GO:0008144 1 14

C0003862 GO:0043256 1 14

C0003862 GO:0019047 2 14

C0003862 GO:0004693 1 14

C0003862 GO:0005871 1 14

C0003862 GO:0004691 1 14

C0003862 GO:0004690 1 14

C0003862 GO:0051457 1 14

C0003862 GO:0009394 3 14

C0003862 GO:0000127 1 14

C0003862 GO:0007127 2 14

C0003862 GO:0046330 1 14

C0003862 GO:0043484 1 14

C0003862 GO:0045178 3 14

C0003862 GO:0002228 2 14

C0003862 GO:0045741 1 14

C0003862 GO:0045621 1 14

C0003862 GO:0005507 1 14

C0003862 GO:0002221 1 14

C0003862 GO:0045749 2 14

C0003862 GO:0033108 2 14

C0003862 GO:0008287 1 14

C0003862 GO:0032728 1 14

C0003862 GO:0042288 1 14

C0003862 GO:0043550 2 14

C0003862 GO:0005913 2 14

C0003862 GO:0001656 3 14

C0003862 GO:0046504 1 14

C0003862 GO:0006978 1 14

C0003862 GO:0034329 1 14

C0003862 GO:0035267 1 14

C0003862 GO:0005786 1 14

C0003862 GO:0045742 1 14

C0003862 GO:0010718 1 14

C0003862 GO:0016528 1 14

C0003862 GO:0016529 1 14

C0003862 GO:0003899 2 14

C0003862 GO:0042797 1 14

C0003862 GO:0016254 1 14

C0003862 GO:0042791 1 14

C0003862 GO:0017046 1 14

C0003862 GO:0045923 1 14

C0003862 GO:0018105 1 14

C0003862 GO:0017124 2 14

C0003862 GO:0018108 1 14

C0003862 GO:0060021 1 14

C0003862 GO:0001889 1 14

C0003862 GO:0007270 1 14

C0003862 GO:0043631 1 14

C0003862 GO:0006090 1 14

C0003862 GO:0010828 1 14

C0003862 GO:0022409 1 14

C0003862 GO:0001725 1 14

C0003862 GO:0045446 1 14

C0003862 GO:0033993 1 14

C0003862 GO:0043189 1 14

C0003862 GO:0018409 1 14

C0003862 GO:0045954 1 14

C0003862 GO:0003730 4 14

C0003862 GO:0045814 1 14

C0003862 GO:0019213 1 14

C0003862 GO:0045089 2 14

C0003862 GO:0045088 1 14

C0003862 GO:0070120 3 14

C0003862 GO:0051291 1 14

C0003862 GO:0048592 1 14

C0003862 GO:0048593 3 14

C0003862 GO:0000272 1 14

C0003862 GO:0031047 1 14

C0003862 GO:0000445 2 14

C0003862 GO:0003746 1 14

C0003862 GO:0050750 1 14

C0003862 GO:0001838 1 14

C0003862 GO:0030530 2 14

C0003862 GO:0040017 1 14

C0003862 GO:0030532 2 14

C0003862 GO:0035194 1 14

C0003862 GO:0008517 1 14

C0003862 GO:0042752 1 14

C0003862 GO:0032320 1 14

C0003862 GO:0005763 2 14

C0003862 GO:0048524 1 14

C0003862 GO:0051806 1 14

C0003862 GO:0006413 1 14

C0003862 GO:0006144 1 14

C0003862 GO:0043531 1 14

C0003862 GO:0034446 1 14

C0003862 GO:0000086 1 14

C0003862 GO:0007612 1 14

C0003862 GO:0000080 1 14

C0003862 GO:0009451 1 14

C0003862 GO:0007162 2 14

C0003862 GO:0043523 1 14

C0003862 GO:0043525 2 14

C0003862 GO:0031593 1 14

C0003862 GO:0007006 2 14

C0003862 GO:0007004 2 14

C0003862 GO:0016180 1 14

C0003862 GO:0002263 1 14

C0003862 GO:0010257 2 14

C0003862 GO:0045667 1 14

C0003862 GO:0001909 1 14

C0003862 GO:0070531 1 14

C0003862 GO:0032135 3 14

C0003862 GO:0045669 1 14

C0003862 GO:0033032 3 14

C0003862 GO:0009167 1 14

C0003862 GO:0043967 1 14

C0003862 GO:0043968 1 14

C0003862 GO:0005310 1 14

C0003862 GO:0016209 1 14

C0003862 GO:0004385 2 14

C0003862 GO:0051059 1 14

C0003862 GO:0000792 1 14

C0003862 GO:0016893 3 14

C0003862 GO:0000790 2 14

C0003862 GO:0016891 4 14

C0003862 GO:0000796 2 14

C0003862 GO:0000794 2 14

C0003862 GO:0042992 2 14

C0003862 GO:0042993 1 14

C0003862 GO:0042990 1 14

C0003862 GO:0014020 1 14

C0003862 GO:0018209 2 14

C0003862 GO:0000159 1 14

C0003862 GO:0042401 1 14

C0003862 GO:0034284 1 14

C0003862 GO:0000018 3 14

C0003862 GO:0031513 1 14

C0003862 GO:0005112 1 14

C0003862 GO:0046530 1 14

C0003862 GO:0004707 1 14

C0003862 GO:0016565 1 14

C0003862 GO:0016291 1 14

C0003862 GO:0016441 1 14

C0003862 GO:0005388 1 14

C0003862 GO:0016445 1 14

C0003862 GO:0004708 2 14

C0003862 GO:0046718 1 14

C0003862 GO:0018024 2 14

C0003862 GO:0006297 4 14

C0003862 GO:0002839 1 14

C0003862 GO:0005732 1 14

C0003862 GO:0015851 1 14

C0003862 GO:0002834 1 14

C0003862 GO:0002837 1 14

C0003862 GO:0002836 1 14

C0003862 GO:0034062 2 14

C0003862 GO:0034061 1 14

C0003862 GO:0008250 1 14

C0003862 GO:0050821 1 14

C0003862 GO:0050820 1 14

C0003862 GO:0001763 1 14

C0003862 GO:0006801 1 14

C0003862 GO:0005929 3 14

C0003862 GO:0051028 1 14

C0003862 GO:0046427 1 14

C0003862 GO:0006563 3 14

C0003862 GO:0051180 1 14

C0003862 GO:0051183 2 14

C0003862 GO:0009126 1 14

C0003862 GO:0010149 1 14

C0003862 GO:0007416 1 14

C0003862 GO:0007183 1 14

C0003862 GO:0016363 2 14

C0003862 GO:0000723 1 14

C0003862 GO:0032813 1 14

C0003862 GO:0009881 1 14

C0003862 GO:0000339 3 14

C0003862 GO:0032769 1 14

C0003862 GO:0017015 1 14

C0003862 GO:0019319 2 14

C0003862 GO:0050795 1 14

C0003862 GO:0031163 1 14

C0003862 GO:0008499 1 14

C0003862 GO:0030069 2 14

C0003862 GO:0046148 1 14

C0003862 GO:0005720 2 14

C0003862 GO:0008652 1 14

C0003862 GO:0007220 1 14

C0003862 GO:0009953 1 14

C0003862 GO:0002711 2 14

C0003862 GO:0010717 1 14

C0003862 GO:0042267 2 14

C0003862 GO:0032088 1 14

C0003862 GO:0043154 1 14

C0003862 GO:0046823 2 14

C0003862 GO:0043014 1 14

C0003862 GO:0006518 1 14

C0003862 GO:0043010 1 14

C0003862 GO:0042834 1 14

C0003862 GO:0016796 2 14

C0003862 GO:0016790 1 14

C0003862 GO:0048306 1 14

C0003862 GO:0016799 3 14

C0003862 GO:0070279 1 14

C0003862 GO:0051646 1 14

C0003862 GO:0031307 1 14

C0003862 GO:0031306 1 14

C0003862 GO:0004859 1 14

C0003862 GO:0007041 1 14

C0003862 GO:0000428 2 14

C0003862 GO:0043409 1 14

C0003862 GO:0016706 1 14

C0003862 GO:0004527 2 14

C0003862 GO:0004526 2 14

C0003862 GO:0004521 3 14

C0003862 GO:0004520 1 14

C0003862 GO:0050768 1 14

C0003862 GO:0015166 1 14

C0003862 GO:0015165 1 14

C0003862 GO:0021954 3 14

C0003862 GO:0006744 1 14

C0003862 GO:0006743 1 14

C0003862 GO:0021953 3 14

C0003862 GO:0032404 4 14

C0003862 GO:0016126 1 14

C0003862 GO:0042169 1 14

C0003862 GO:0008139 1 14

C0003862 GO:0042162 1 14

C0003862 GO:0070301 1 14

C0003862 GO:0070304 1 14

C0003862 GO:0043270 1 14

C0003862 GO:0031397 1 14

C0003862 GO:0014065 3 14

C0003862 GO:0009408 2 14

C0003862 GO:0007131 1 14

C0003862 GO:0005048 1 14

C0003862 GO:0043473 2 14

C0003862 GO:0002460 1 14

C0003862 GO:0030174 2 14

C0003862 GO:0000299 1 14

C0003862 GO:0002366 1 14

C0003862 GO:0009084 4 14

C0003862 GO:0016018 2 14

C0003862 GO:0004177 2 14

C0003862 GO:0016331 2 14

C0003862 GO:0050868 1 14

C0003862 GO:0046364 3 14

C0003862 GO:0030983 3 14

C0003862 GO:0009108 1 14

C0003862 GO:0034235 1 14

C0003862 GO:0001523 1 14

C0003862 GO:0016846 1 14

C0003862 GO:0016840 1 14

C0003862 GO:0000060 1 14

C0003862 GO:0005814 1 14

C0003862 GO:0042787 1 14

C0003862 GO:0018279 1 14

C0003862 GO:0045930 2 14

C0003862 GO:0017119 1 14

C0003862 GO:0010810 1 14

C0003862 GO:0031124 2 14

C0003862 GO:0010812 2 14

C0003862 GO:0031123 2 14

C0003862 GO:0016505 1 14

C0003862 GO:0031572 1 14

C0003862 GO:0043547 1 14

C0003862 GO:0009746 1 14

C0003862 GO:0003709 1 14

C0003862 GO:0051881 1 14

C0003862 GO:0042345 1 14

C0003862 GO:0002793 1 14

C0003862 GO:0051923 1 14

C0003862 GO:0032200 1 14

C0003862 GO:0009749 1 14

C0003862 GO:0005662 1 14

C0003862 GO:0070652 1 14

C0003862 GO:0000387 2 14

C0003862 GO:0006390 2 14

C0003862 GO:0043548 1 14

C0003862 GO:0002833 1 14

C0003862 GO:0006776 2 14

C0003862 GO:0052192 1 14

C0003862 GO:0000245 2 14

C0003862 GO:0016864 1 14

C0003862 GO:0009218 2 14

C0003862 GO:0031970 1 14

C0003862 GO:0032387 2 14

C0003862 GO:0019228 1 14

C0003862 GO:0006474 1 14

C0003862 GO:0006477 1 14

C0003862 GO:0006476 1 14

C0003862 GO:0030509 1 14

C0003862 GO:0030111 1 14

C0003862 GO:0030119 1 14

C0003862 GO:0030118 1 14

C0003862 GO:0046131 1 14

C0003862 GO:0008375 1 14

C0003862 GO:0043028 1 14

C0003862 GO:0006783 1 14

C0003862 GO:0007213 1 14

C0003862 GO:0046638 2 14

C0003862 GO:0042531 1 14

C0003862 GO:0005657 1 14

C0003862 GO:0006073 1 14

C0003862 GO:0010721 1 14

C0003862 GO:0005678 3 14

C0003862 GO:0032655 1 14

C0003862 GO:0015718 1 14

C0003862 GO:0005092 1 14

C0003862 GO:0006607 2 14

C0003862 GO:0008239 1 14

C0003862 GO:0005095 3 14

C0003862 GO:0031274 2 14

C0003862 GO:0019903 1 14

C0003862 GO:0019902 1 14

C0003862 GO:0007172 1 14

C0003862 GO:0000777 1 14

C0003862 GO:0031272 2 14

C0003862 GO:0007076 2 14

C0003862 GO:0007568 1 14

C0003862 GO:0007598 2 14

C0003862 GO:0007569 1 14

C0003862 GO:0045793 1 14

C0003862 GO:0051262 1 14

C0003862 GO:0050732 1 14

C0003862 GO:0031264 1 14

C0003862 GO:0005527 2 14

C0003862 GO:0033135 1 14

C0003862 GO:0001912 1 14

C0003862 GO:0004550 1 14

C0003862 GO:0051972 1 14

C0003862 GO:0006672 2 14

C0003862 GO:0003015 1 14

C0003862 GO:0022602 1 14

C0003862 GO:0042398 1 14

C0003862 GO:0016278 2 14

C0003862 GO:0016279 2 14

C0003862 GO:0034404 1 14

C0003862 GO:0043331 1 14

C0003862 GO:0016885 2 14

C0003862 GO:0000784 1 14

C0003862 GO:0009065 1 14

C0003862 GO:0009064 1 14

C0003862 GO:0070918 2 14

C0003862 GO:0031576 2 14

C0003862 GO:0042625 1 14

C0003862 GO:0016289 2 14

C0003862 GO:0005852 5 14

C0003862 GO:0045116 2 14

C0003862 GO:0090100 1 14

C0003862 GO:0005851 3 14

C0003862 GO:0016281 1 14

C0003862 GO:0003724 1 14

C0003862 GO:0031579 2 14

C0003862 GO:0005528 2 14

C0003862 GO:0016571 1 14

C0003862 GO:0005522 2 14

C0003862 GO:0002200 1 14

C0003862 GO:0032925 1 14

C0003862 GO:0016471 1 14

C0003862 GO:0004128 2 14

C0003862 GO:0055029 2 14

C0003862 GO:0035251 1 14

C0003862 GO:0035250 1 14

C0003862 GO:0030374 1 14

C0003862 GO:0006953 1 14

C0003862 GO:0006189 3 14

C0003862 GO:0006188 3 14

C0003862 GO:0042308 2 14

C0003862 GO:0042307 1 14

C0003862 GO:0042306 2 14

C0003862 GO:0001672 1 14

C0003862 GO:0042301 1 14

C0003862 GO:0060606 1 14

C0003862 GO:0002758 1 14

C0003862 GO:0016831 1 14

C0003862 GO:0006213 1 14

C0003862 GO:0006739 1 14

C0003862 GO:0006359 1 14

C0003862 GO:0033176 1 14

C0003862 GO:0018196 1 14

C0003862 GO:0030159 1 14

C0003862 GO:0045055 1 14

C0003862 GO:0042772 1 14

C0003862 GO:0005545 2 14

C0003862 GO:0045580 2 14

C0003862 GO:0034654 1 14

C0003862 GO:0045582 2 14

C0003862 GO:0060047 1 14

C0003862 GO:0046677 2 14

C0003862 GO:0001953 2 14

C0003862 GO:0015238 1 14

C0003862 GO:0008235 1 14

C0003862 GO:0045426 1 14

C0003862 GO:0034399 2 14

C0003862 GO:0051324 1 14

C0003862 GO:0001701 2 14

C0003862 GO:0019783 2 14

C0003862 GO:0001707 2 14

C0003862 GO:0001704 2 14

C0003862 GO:0051087 3 14

C0003862 GO:0015711 1 14

C0003862 GO:0042375 1 14

C0003862 GO:0070761 2 14

C0003862 GO:0032272 2 14

C0003862 GO:0070688 2 14

C0003862 GO:0008603 1 14

C0003862 GO:0017156 2 14

C0003862 GO:0017157 1 14

C0003862 GO:0043535 1 14

C0003862 GO:0048332 2 14

C0003862 GO:0006721 1 14

C0003862 GO:0032508 1 14

C0003862 GO:0031333 1 14

C0003862 GO:0000217 1 14

C0003862 GO:0030128 1 14

C0003862 GO:0016073 1 14

C0003862 GO:0030122 1 14

C0003862 GO:0004869 1 14

C0003862 GO:0070198 1 14

C0003862 GO:0051258 1 14

C0003862 GO:0048839 1 14

C0003862 GO:0001959 1 14

C0003862 GO:0015884 1 14

C0003862 GO:0006383 1 14

C0003862 GO:0032182 2 14

C0003862 GO:0001952 1 14

C0003862 GO:0001950 1 14

C0003862 GO:0005742 2 14

C0003862 GO:0051828 1 14

C0003862 GO:0016653 3 14

C0003862 GO:0010770 1 14

C0003862 GO:0006000 1 14

C0003862 GO:0034502 1 14

C0003862 GO:0042116 1 14

C0003862 GO:0048754 1 14

C0003862 GO:0005891 1 14

C0003862 GO:0043506 1 14

C0003862 GO:0045768 1 14

C0003862 GO:0070822 1 14

C0003862 GO:0032981 2 14

C0003862 GO:0051287 1 14

C0003862 GO:0005003 1 14

C0003862 GO:0045766 2 14

C0003862 GO:0045767 1 14

C0003862 GO:0046040 3 14

C0003862 GO:0005313 1 14

C0003862 GO:0016229 1 14

C0003862 GO:0005007 1 14

C0003862 GO:0034614 1 14

C0003862 GO:0016226 1 14

C0003862 GO:0016878 1 14

C0003862 GO:0000779 3 14

C0003862 GO:0009110 1 14

C0003862 GO:0009116 2 14

C0003862 GO:0002717 1 14

C0003862 GO:0002718 2 14

C0003862 GO:0070925 2 14

C0003862 GO:0016877 1 14

C0003862 GO:0031529 1 14

C0003862 GO:0000178 1 14

C0003862 GO:0001516 1 14

C0003862 GO:0008170 1 14

C0003862 GO:0000175 1 14

C0003862 GO:0046457 1 14

C0003862 GO:0046456 1 14

C0003862 GO:0043596 1 14

C0003862 GO:0030900 1 14

C0003862 GO:0046519 2 14

C0003862 GO:0016504 1 14

C0003862 GO:0016896 1 14

C0003862 GO:0007090 1 14

C0003862 GO:0008589 1 14

C0003862 GO:0031114 1 14

C0003862 GO:0016894 1 14

C0003862 GO:0051289 1 14

C0003862 GO:0046637 1 14

C0003862 GO:0050684 1 14

C0003862 GO:0046635 1 14

C0003862 GO:0046634 1 14

C0003862 GO:0046631 1 14

C0003862 GO:0050920 1 14

C0003862 GO:0050921 1 14

C0003862 GO:0008272 1 14

C0003862 GO:0051893 1 14

C0003862 GO:0016725 1 14

C0003862 GO:0051897 1 14

C0003862 GO:0008278 2 14

C0003862 GO:0031985 1 14

C0003862 GO:0031984 1 14

C0003862 GO:0006983 1 14

C0003862 GO:0043044 1 14

C0003862 GO:0043043 1 14

C0003862 GO:0009071 1 14

C0003862 GO:0006266 1 14

C0003862 GO:0004089 1 14

C0003862 GO:0006268 1 14

C0003862 GO:0032392 1 14

C0003862 GO:0005791 1 14

C0003862 GO:0006760 1 14

C0003862 GO:0070585 1 14

C0003862 GO:0000314 2 14

C0003862 GO:0030165 1 14

C0003862 GO:0000502 2 14

C0003862 GO:0030511 1 14

C0003862 GO:0005066 1 14

C0003862 GO:0051354 1 14

C0003862 GO:0017069 1 14

C0003862 GO:0005663 3 14

C0003862 GO:0043130 2 14

C0003862 GO:0005665 1 14

C0003862 GO:0006164 2 14

C0003862 GO:0005669 1 14

C0003862 GO:0019438 1 14

C0003862 GO:0030194 1 14

C0003862 GO:0005089 1 14

C0003862 GO:0005123 1 14

C0003862 GO:0032648 1 14

C0003862 GO:0042744 1 14

C0003862 GO:0032947 2 14

C0003862 GO:0031365 1 14

C0003862 GO:0043425 1 14

C0003862 GO:0007064 1 14

C0003862 GO:0031369 1 14

C0003862 GO:0008408 3 14

C0003862 GO:0000381 2 14

C0003862 GO:0016045 1 14

C0003862 GO:0035085 2 14

C0003862 GO:0019239 2 14

C0003862 GO:0033014 1 14

C0003862 GO:0006687 1 14

C0003862 GO:0006684 2 14

C0003862 GO:0016763 3 14

C0003862 GO:0001841 1 14

C0003862 GO:0001843 1 14

C0003862 GO:0043168 1 14

C0003862 GO:0030894 2 14

C0003862 GO:0042384 1 14

C0003862 GO:0009156 4 14

C0003862 GO:0048500 1 14

C0003862 GO:0009152 3 14

C0003862 GO:0008013 1 14

C0003862 GO:0019104 3 14

C0003862 GO:0007026 1 14

C0003862 GO:0008625 1 14

C0003862 GO:0048256 2 14

C0003862 GO:0014003 1 14

C0003862 GO:0000030 2 14

C0003862 GO:0043498 1 14

C0003862 GO:0005844 1 14

C0003862 GO:0000132 1 14

C0003862 GO:0004683 2 14

C0003862 GO:0031647 1 14

C0003862 GO:0048469 1 14

C0003862 GO:0002218 1 14

C0003862 GO:0045736 2 14

C0003862 GO:0045739 2 14

C0003862 GO:0015665 1 14

C0003862 GO:0005537 1 14

C0003862 GO:0055038 1 14

C0003862 GO:0030307 1 14

C0003862 GO:0055037 1 14

C0003862 GO:0052126 1 14

C0003862 GO:0035148 1 14

C0003862 GO:0030261 2 14

C0003862 GO:0030260 1 14

C0003862 GO:0032271 1 14

C0003862 GO:0048024 1 14

C0003862 GO:0009123 1 14

C0003862 GO:0043087 1 14

C0003862 GO:0009127 3 14

C0003862 GO:0009124 1 14

C0003862 GO:0006220 4 14

C0003862 GO:0006221 2 14

C0003862 GO:0000049 1 14

C0003862 GO:0070603 1 14

C0003862 GO:0000726 1 14

C0003862 GO:0002444 2 14

C0003862 GO:0042921 2 14

C0003862 GO:0046784 2 14

C0003862 GO:0046782 1 14

C0003862 GO:0044452 2 14

C0003862 GO:0022612 1 14

C0003862 GO:0022616 2 14

C0003862 GO:0003756 1 14

C0003862 GO:0015295 1 14

C0003862 GO:0010939 1 14

C0003862 GO:0046165 2 14

C0003862 GO:0016411 1 14

C0003862 GO:0015298 1 14

C0003862 GO:0035035 1 14

C0003862 GO:0006298 2 14

C0003862 GO:0015804 1 14

C0003862 GO:0002224 1 14

C0003862 GO:0010833 2 14

C0003862 GO:0051310 2 14

C0003862 GO:0009743 1 14

C0003862 GO:0051092 1 14

C0003862 GO:0045622 1 14

C0003862 GO:0043601 2 14

C0003862 GO:0051096 1 14

C0003862 GO:0008633 1 14

C0003862 GO:0007567 1 14

C0003862 GO:0042364 1 14

C0003862 GO:0070775 1 14

C0003862 GO:0003727 3 14

C0003862 GO:0006536 2 14

C0003862 GO:0048009 1 14

C0003862 GO:0030099 1 14

C0003862 GO:0043370 1 14

C0003862 GO:0003684 1 14

C0003862 GO:0046326 1 14

C0003862 GO:0003705 1 14

C0003862 GO:0000347 2 14

C0003862 GO:0000346 2 14

C0003862 GO:0046112 1 14

C0003862 GO:0030132 1 14

C0003862 GO:0032496 1 14

C0003862 GO:0050661 1 14

C0003862 GO:0032405 1 14

C0003862 GO:0008329 1 14

C0003862 GO:0032202 1 14

C0003862 GO:0021510 2 14

C0003862 GO:0016646 1 14

C0003862 GO:0016645 1 14

C0003862 GO:0008526 2 14

C0003862 GO:0044409 1 14

C0003862 GO:0070412 1 14

C0003862 GO:0006400 1 14

C0003862 GO:0006406 1 14

C0003862 GO:0048531 1 14

C0003862 GO:0070328 1 14

C0003862 GO:0070325 2 14

C0003862 GO:0004549 2 14

C0003862 GO:0005885 1 14

C0003862 GO:0005884 1 14

C0003862 GO:0016049 1 14

C0003862 GO:0007157 1 14

C0003862 GO:0002443 1 14

C0003862 GO:0051896 1 14

C0003862 GO:0002446 2 14

C0003862 GO:0042771 1 14

C0003862 GO:0030898 2 14

C0003862 GO:0016580 1 14

C0003862 GO:0016581 2 14

C0003862 GO:0001938 1 14

C0003862 GO:0005577 2 14

C0003862 GO:0002250 1 14

C0003862 GO:0015030 1 14

C0003862 GO:0001933 1 14

C0003862 GO:0004579 1 14

C0003862 GO:0001937 3 14

C0003862 GO:0001936 2 14

C0003864 GO:0006776 1 2

C0003864 GO:0000245 1 2

C0003864 GO:0022624 1 2

C0003864 GO:0034446 1 2

C0003864 GO:0050684 1 2

C0003864 GO:0006474 1 2

C0003864 GO:0006476 1 2

C0003864 GO:0002706 1 2

C0003864 GO:0002705 1 2

C0003864 GO:0051897 1 2

C0003864 GO:0002709 1 2

C0003864 GO:0007004 2 2

C0003864 GO:0005838 1 2

C0003864 GO:0051289 1 2

C0003864 GO:0043043 1 2

C0003864 GO:0010257 1 2

C0003864 GO:0007080 1 2

C0003864 GO:0032135 1 2

C0003864 GO:0006760 1 2

C0003864 GO:0043967 1 2

C0003864 GO:0005092 1 2

C0003864 GO:0002824 1 2

C0003864 GO:0004385 1 2

C0003864 GO:0006607 1 2

C0003864 GO:0002821 1 2

C0003864 GO:0003841 1 2

C0003864 GO:0016891 1 2

C0003864 GO:0000796 1 2

C0003864 GO:0007172 1 2

C0003864 GO:0000794 1 2

C0003864 GO:0007076 1 2

C0003864 GO:0000502 1 2

C0003864 GO:0009071 1 2

C0003864 GO:0045793 1 2

C0003864 GO:0006278 2 2

C0003864 GO:0018209 1 2

C0003864 GO:0006271 1 2

C0003864 GO:0006270 1 2

C0003864 GO:0000018 1 2

C0003864 GO:0005663 1 2

C0003864 GO:0031306 1 2

C0003864 GO:0004550 1 2

C0003864 GO:0006164 1 2

C0003864 GO:0016445 1 2

C0003864 GO:0006297 2 2

C0003864 GO:0018149 1 2

C0003864 GO:0002839 1 2

C0003864 GO:0002834 1 2

C0003864 GO:0002837 1 2

C0003864 GO:0002836 1 2

C0003864 GO:0034061 1 2

C0003864 GO:0004693 1 2

C0003864 GO:0001763 1 2

C0003864 GO:0016885 1 2

C0003864 GO:0031365 1 2

C0003864 GO:0009064 1 2

C0003864 GO:0016607 1 2

C0003864 GO:0016289 1 2

C0003864 GO:0000381 1 2

C0003864 GO:0045116 1 2

C0003864 GO:0005851 1 2

C0003864 GO:0016045 1 2

C0003864 GO:0016281 1 2

C0003864 GO:0009168 1 2

C0003864 GO:0016763 1 2

C0003864 GO:0045913 1 2

C0003864 GO:0002200 1 2

C0003864 GO:0002708 1 2

C0003864 GO:0032813 1 2

C0003864 GO:0009156 1 2

C0003864 GO:0019319 1 2

C0003864 GO:0009152 1 2

C0003864 GO:0043410 1 2

C0003864 GO:0009260 1 2

C0003864 GO:0031163 1 2

C0003864 GO:0007127 1 2

C0003864 GO:0006189 1 2

C0003864 GO:0006188 1 2

C0003864 GO:0032890 1 2

C0003864 GO:0006303 1 2

C0003864 GO:0030069 1 2

C0003864 GO:0001672 1 2

C0003864 GO:0005720 1 2

C0003864 GO:0005844 1 2

C0003864 GO:0031080 1 2

C0003864 GO:0016254 1 2

C0003864 GO:0042267 1 2

C0003864 GO:0032088 1 2

C0003864 GO:0015074 1 2

C0003864 GO:0045739 1 2

C0003864 GO:0042834 1 2

C0003864 GO:0043484 1 2

C0003864 GO:0042752 1 2

C0003864 GO:0030307 1 2

C0003864 GO:0030261 1 2

C0003864 GO:0018105 1 2

C0003864 GO:0048024 1 2

C0003864 GO:0009127 1 2

C0003864 GO:0000723 1 2

C0003864 GO:0001701 1 2

C0003864 GO:0019783 1 2

C0003864 GO:0000726 1 2

C0003864 GO:0004526 1 2

C0003864 GO:0046330 1 2

C0003864 GO:0002228 1 2

C0003864 GO:0021954 1 2

C0003864 GO:0022616 1 2

C0003864 GO:0021953 1 2

C0003864 GO:0045749 1 2

C0003864 GO:0033108 1 2

C0003864 GO:0008139 1 2

C0003864 GO:0070304 1 2

C0003864 GO:0010833 2 2

C0003864 GO:0010676 1 2

C0003864 GO:0051310 1 2

C0003864 GO:0009408 1 2

C0003864 GO:0007131 1 2

C0003864 GO:0030530 1 2

C0003864 GO:0051096 1 2

C0003864 GO:0010907 1 2

C0003864 GO:0005742 1 2

C0003864 GO:0003727 1 2

C0003864 GO:0006536 1 2

C0003864 GO:0030099 1 2

C0003864 GO:0009084 2 2

C0003864 GO:0032404 1 2

C0003864 GO:0006000 1 2

C0003864 GO:0004177 1 2

C0003864 GO:0019047 1 2

C0003864 GO:0009065 1 2

C0003864 GO:0046364 1 2

C0003864 GO:0048754 1 2

C0003864 GO:0030983 1 2

C0003864 GO:0046040 1 2

C0003864 GO:0032981 1 2

C0003864 GO:0018409 1 2

C0003864 GO:0042787 1 2

C0003864 GO:0003730 1 2

C0003864 GO:0045930 1 2

C0003864 GO:0070328 1 2

C0003864 GO:0070120 1 2

C0003864 GO:0004549 1 2

C0003864 GO:0016226 1 2

C0003864 GO:0016878 1 2

C0003864 GO:0002711 2 2

C0003864 GO:0051646 1 2

C0003864 GO:0005852 1 2

C0003864 GO:0051881 1 2

C0003864 GO:0030532 1 2

C0003864 GO:0032200 1 2

C0003864 GO:0005577 1 2

C0003864 GO:0019902 1 2

C0003864 GO:0000790 1 2

C0003864 GO:0000387 1 2

C0003864 GO:0043498 1 2

C0003864 GO:0002833 1 2

C0006277 GO:0006479 1 5

C0006277 GO:0000245 1 5

C0006277 GO:0022624 1 5

C0006277 GO:0046638 1 5

C0006277 GO:0050684 1 5

C0006277 GO:0031970 1 5

C0006277 GO:0048469 1 5

C0006277 GO:0000080 1 5

C0006277 GO:0009451 1 5

C0006277 GO:0002706 1 5

C0006277 GO:0008276 1 5

C0006277 GO:0046631 1 5

C0006277 GO:0035085 1 5

C0006277 GO:0007006 1 5

C0006277 GO:0002709 1 5

C0006277 GO:0007004 1 5

C0006277 GO:0005838 1 5

C0006277 GO:0006983 1 5

C0006277 GO:0050820 1 5

C0006277 GO:0043043 1 5

C0006277 GO:0010257 1 5

C0006277 GO:0006266 1 5

C0006277 GO:0007080 1 5

C0006277 GO:0010894 1 5

C0006277 GO:0051287 1 5

C0006277 GO:0046504 1 5

C0006277 GO:0032135 1 5

C0006277 GO:0033032 1 5

C0006277 GO:0006760 1 5

C0006277 GO:0006563 1 5

C0006277 GO:0005092 1 5

C0006277 GO:0002824 1 5

C0006277 GO:0004385 1 5

C0006277 GO:0006607 1 5

C0006277 GO:0002821 1 5

C0006277 GO:0003841 1 5

C0006277 GO:0016891 1 5

C0006277 GO:0000796 1 5

C0006277 GO:0007172 1 5

C0006277 GO:0007076 1 5

C0006277 GO:0000502 1 5

C0006277 GO:0007598 1 5

C0006277 GO:0009071 1 5

C0006277 GO:0045793 1 5

C0006277 GO:0006278 1 5

C0006277 GO:0018209 1 5

C0006277 GO:0006271 1 5

C0006277 GO:0000018 1 5

C0006277 GO:0010887 1 5

C0006277 GO:0005663 1 5

C0006277 GO:0031306 1 5

C0006277 GO:0006164 1 5

C0006277 GO:0051087 1 5

C0006277 GO:0070567 1 5

C0006277 GO:0016445 1 5

C0006277 GO:0030194 1 5

C0006277 GO:0006297 1 5

C0006277 GO:0002839 1 5

C0006277 GO:0048256 1 5

C0006277 GO:0042398 1 5

C0006277 GO:0002834 1 5

C0006277 GO:0002837 1 5

C0006277 GO:0002836 1 5

C0006277 GO:0034061 1 5

C0006277 GO:0004693 1 5

C0006277 GO:0001763 1 5

C0006277 GO:0016885 1 5

C0006277 GO:0043425 1 5

C0006277 GO:0005487 1 5

C0006277 GO:0021700 1 5

C0006277 GO:0016944 1 5

C0006277 GO:0016607 2 5

C0006277 GO:0016289 1 5

C0006277 GO:0000381 1 5

C0006277 GO:0005851 1 5

C0006277 GO:0016801 1 5

C0006277 GO:0043548 1 5

C0006277 GO:0010885 1 5

C0006277 GO:0042558 1 5

C0006277 GO:0042559 1 5

C0006277 GO:0070742 1 5

C0006277 GO:0045939 1 5

C0006277 GO:0019239 2 5

C0006277 GO:0009168 1 5

C0006277 GO:0000339 1 5

C0006277 GO:0002200 1 5

C0006277 GO:0016363 1 5

C0006277 GO:0016471 1 5

C0006277 GO:0032813 1 5

C0006277 GO:0009309 1 5

C0006277 GO:0009156 2 5

C0006277 GO:0009304 1 5

C0006277 GO:0032479 1 5

C0006277 GO:0048709 1 5

C0006277 GO:0019319 1 5

C0006277 GO:0009152 2 5

C0006277 GO:0009260 3 5

C0006277 GO:0009262 1 5

C0006277 GO:0006189 1 5

C0006277 GO:0006188 1 5

C0006277 GO:0014003 1 5

C0006277 GO:0006303 1 5

C0006277 GO:0030069 1 5

C0006277 GO:0001672 1 5

C0006277 GO:0016018 1 5

C0006277 GO:0005720 1 5

C0006277 GO:0005844 1 5

C0006277 GO:0031080 1 5

C0006277 GO:0004683 1 5

C0006277 GO:0042267 1 5

C0006277 GO:0015074 1 5

C0006277 GO:0050810 1 5

C0006277 GO:0045739 1 5

C0006277 GO:0033176 1 5

C0006277 GO:0009264 1 5

C0006277 GO:0043484 1 5

C0006277 GO:0030307 1 5

C0006277 GO:0033209 1 5

C0006277 GO:0030261 1 5

C0006277 GO:0042401 1 5

C0006277 GO:0045582 1 5

C0006277 GO:0018105 1 5

C0006277 GO:0016049 1 5

C0006277 GO:0007041 1 5

C0006277 GO:0048024 1 5

C0006277 GO:0030705 1 5

C0006277 GO:0043087 1 5

C0006277 GO:0009127 1 5

C0006277 GO:0034399 1 5

C0006277 GO:0009394 1 5

C0006277 GO:0004526 1 5

C0006277 GO:0042375 1 5

C0006277 GO:0046784 1 5

C0006277 GO:0002228 1 5

C0006277 GO:0046782 1 5

C0006277 GO:0033135 1 5

C0006277 GO:0021954 1 5

C0006277 GO:0006744 1 5

C0006277 GO:0006743 1 5

C0006277 GO:0022616 2 5

C0006277 GO:0021953 1 5

C0006277 GO:0045749 1 5

C0006277 GO:0033108 1 5

C0006277 GO:0008139 1 5

C0006277 GO:0042162 1 5

C0006277 GO:0045426 1 5

C0006277 GO:0010833 1 5

C0006277 GO:0051310 1 5

C0006277 GO:0016504 1 5

C0006277 GO:0030530 1 5

C0006277 GO:0008630 1 5

C0006277 GO:0030174 1 5

C0006277 GO:0015884 1 5

C0006277 GO:0001953 1 5

C0006277 GO:0001952 1 5

C0006277 GO:0005742 1 5

C0006277 GO:0003727 1 5

C0006277 GO:0006536 1 5

C0006277 GO:0048009 1 5

C0006277 GO:0017156 1 5

C0006277 GO:0016653 1 5

C0006277 GO:0009084 1 5

C0006277 GO:0009108 1 5

C0006277 GO:0032404 1 5

C0006277 GO:0006000 1 5

C0006277 GO:0017046 1 5

C0006277 GO:0004177 1 5

C0006277 GO:0019200 1 5

C0006277 GO:0019047 1 5

C0006277 GO:0046364 1 5

C0006277 GO:0048754 1 5

C0006277 GO:0030983 1 5

C0006277 GO:0008213 1 5

C0006277 GO:0046040 1 5

C0006277 GO:0000347 1 5

C0006277 GO:0000346 1 5

C0006277 GO:0048593 1 5

C0006277 GO:0016840 1 5

C0006277 GO:0032981 1 5

C0006277 GO:0003730 1 5

C0006277 GO:0006400 1 5

C0006277 GO:0016229 1 5

C0006277 GO:0045930 1 5

C0006277 GO:0033764 1 5

C0006277 GO:0045088 1 5

C0006277 GO:0070120 1 5

C0006277 GO:0030330 1 5

C0006277 GO:0010810 1 5

C0006277 GO:0019218 1 5

C0006277 GO:0010812 1 5

C0006277 GO:0001570 1 5

C0006277 GO:0004549 1 5

C0006277 GO:0034379 1 5

C0006277 GO:0002711 1 5

C0006277 GO:0051646 1 5

C0006277 GO:0005852 1 5

C0006277 GO:0000445 1 5

C0006277 GO:0002444 1 5

C0006277 GO:0002446 1 5

C0006277 GO:0042771 1 5

C0006277 GO:0000178 1 5

C0006277 GO:0051881 1 5

C0006277 GO:0030532 2 5

C0006277 GO:0048066 1 5

C0006277 GO:0042551 1 5

C0006277 GO:0032200 1 5

C0006277 GO:0019902 1 5

C0006277 GO:0000790 1 5

C0006277 GO:0000387 1 5

C0006277 GO:0006390 1 5

C0006277 GO:0016505 1 5

C0006277 GO:0002833 1 5

C0009951 GO:0000245 1 2

C0009951 GO:0022624 1 2

C0009951 GO:0050684 1 2

C0009951 GO:0009451 1 2

C0009951 GO:0002706 1 2

C0009951 GO:0007006 1 2

C0009951 GO:0002709 1 2

C0009951 GO:0007004 1 2

C0009951 GO:0005838 1 2

C0009951 GO:0043043 1 2

C0009951 GO:0010257 1 2

C0009951 GO:0007080 1 2

C0009951 GO:0032135 1 2

C0009951 GO:0006760 1 2

C0009951 GO:0005092 1 2

C0009951 GO:0002824 1 2

C0009951 GO:0004385 1 2

C0009951 GO:0006607 1 2

C0009951 GO:0002821 1 2

C0009951 GO:0003841 1 2

C0009951 GO:0016891 1 2

C0009951 GO:0000796 1 2

C0009951 GO:0007172 1 2

C0009951 GO:0007076 1 2

C0009951 GO:0000502 1 2

C0009951 GO:0009071 1 2

C0009951 GO:0045793 1 2

C0009951 GO:0006278 1 2

C0009951 GO:0018209 1 2

C0009951 GO:0006271 1 2

C0009951 GO:0000018 1 2

C0009951 GO:0005663 1 2

C0009951 GO:0031306 1 2

C0009951 GO:0006164 1 2

C0009951 GO:0051087 1 2

C0009951 GO:0016445 1 2

C0009951 GO:0006297 1 2

C0009951 GO:0002839 1 2

C0009951 GO:0048256 1 2

C0009951 GO:0002834 1 2

C0009951 GO:0002837 1 2

C0009951 GO:0002836 1 2

C0009951 GO:0034061 1 2

C0009951 GO:0004693 1 2

C0009951 GO:0001763 1 2

C0009951 GO:0016885 1 2

C0009951 GO:0005487 1 2

C0009951 GO:0016607 1 2

C0009951 GO:0016289 1 2

C0009951 GO:0000381 1 2

C0009951 GO:0005851 1 2

C0009951 GO:0042559 1 2

C0009951 GO:0009168 1 2

C0009951 GO:0000339 1 2

C0009951 GO:0002200 1 2

C0009951 GO:0032813 1 2

C0009951 GO:0009156 1 2

C0009951 GO:0019319 1 2

C0009951 GO:0009152 2 2

C0009951 GO:0009260 2 2

C0009951 GO:0009262 1 2

C0009951 GO:0006189 1 2

C0009951 GO:0006188 1 2

C0009951 GO:0030069 1 2

C0009951 GO:0001672 1 2

C0009951 GO:0005720 1 2

C0009951 GO:0005844 1 2

C0009951 GO:0031080 1 2

C0009951 GO:0042267 1 2

C0009951 GO:0015074 1 2

C0009951 GO:0045739 1 2

C0009951 GO:0009264 1 2

C0009951 GO:0043484 1 2

C0009951 GO:0030307 1 2

C0009951 GO:0030261 1 2

C0009951 GO:0018105 1 2

C0009951 GO:0048024 1 2

C0009951 GO:0009127 1 2

C0009951 GO:0009394 1 2

C0009951 GO:0004526 1 2

C0009951 GO:0042375 1 2

C0009951 GO:0002228 1 2

C0009951 GO:0021954 1 2

C0009951 GO:0006744 1 2

C0009951 GO:0006743 1 2

C0009951 GO:0022616 1 2

C0009951 GO:0021953 1 2

C0009951 GO:0045749 1 2

C0009951 GO:0033108 1 2

C0009951 GO:0008139 1 2

C0009951 GO:0045426 1 2

C0009951 GO:0010833 1 2

C0009951 GO:0051310 1 2

C0009951 GO:0030530 1 2

C0009951 GO:0005742 1 2

C0009951 GO:0003727 1 2

C0009951 GO:0006536 1 2

C0009951 GO:0009084 1 2

C0009951 GO:0009108 1 2

C0009951 GO:0032404 1 2

C0009951 GO:0006000 1 2

C0009951 GO:0004177 1 2

C0009951 GO:0019047 1 2

C0009951 GO:0046364 1 2

C0009951 GO:0048754 1 2

C0009951 GO:0030983 1 2

C0009951 GO:0046040 1 2

C0009951 GO:0032981 1 2

C0009951 GO:0003730 1 2

C0009951 GO:0006400 1 2

C0009951 GO:0045930 1 2

C0009951 GO:0070120 1 2

C0009951 GO:0004549 1 2

C0009951 GO:0002711 1 2

C0009951 GO:0051646 1 2

C0009951 GO:0005852 1 2

C0009951 GO:0051881 1 2

C0009951 GO:0030532 1 2

C0009951 GO:0032200 1 2

C0009951 GO:0019902 1 2

C0009951 GO:0000790 1 2

C0009951 GO:0000387 1 2

C0009951 GO:0006390 1 2

C0009951 GO:0002833 1 2

C0013404 GO:0010149 1 9

C0013404 GO:0022624 1 9

C0013404 GO:0043954 1 9

C0013404 GO:0002706 1 9

C0013404 GO:0002703 1 9

C0013404 GO:0001508 1 9

C0013404 GO:0002709 1 9

C0013404 GO:0016860 2 9

C0013404 GO:0005838 1 9

C0013404 GO:0005834 1 9

C0013404 GO:0006978 1 9

C0013404 GO:0006309 2 9

C0013404 GO:0002366 1 9

C0013404 GO:0030856 1 9

C0013404 GO:0051294 1 9

C0013404 GO:0002824 1 9

C0013404 GO:0003923 1 9

C0013404 GO:0002822 1 9

C0013404 GO:0031109 1 9

C0013404 GO:0002821 1 9

C0013404 GO:0035270 1 9

C0013404 GO:0003841 1 9

C0013404 GO:0000718 1 9

C0013404 GO:0006278 1 9

C0013404 GO:0006271 1 9

C0013404 GO:0016814 1 9

C0013404 GO:0010887 1 9

C0013404 GO:0010883 1 9

C0013404 GO:0006378 1 9

C0013404 GO:0019320 1 9

C0013404 GO:0031958 1 9

C0013404 GO:0032412 1 9

C0013404 GO:0048568 1 9

C0013404 GO:0000302 2 9

C0013404 GO:0030174 1 9

C0013404 GO:0008484 1 9

C0013404 GO:0016607 1 9

C0013404 GO:0003206 2 9

C0013404 GO:0015992 1 9

C0013404 GO:0015491 1 9

C0013404 GO:0042551 2 9

C0013404 GO:0006885 1 9

C0013404 GO:0042255 1 9

C0013404 GO:0003208 1 9

C0013404 GO:0042558 1 9

C0013404 GO:0002687 1 9

C0013404 GO:0002685 1 9

C0013404 GO:0009168 2 9

C0013404 GO:0043021 2 9

C0013404 GO:0043022 2 9

C0013404 GO:0051879 1 9

C0013404 GO:0043027 1 9

C0013404 GO:0009304 1 9

C0013404 GO:0009303 1 9

C0013404 GO:0009260 2 9

C0013404 GO:0009262 1 9

C0013404 GO:0009264 1 9

C0013404 GO:0009266 1 9

C0013404 GO:0000002 1 9

C0013404 GO:0044042 1 9

C0013404 GO:0030675 1 9

C0013404 GO:0031080 2 9

C0013404 GO:0004536 1 9

C0013404 GO:0002039 1 9

C0013404 GO:0015781 1 9

C0013404 GO:0015780 1 9

C0013404 GO:0004532 1 9

C0013404 GO:0006691 1 9

C0013404 GO:0015074 2 9

C0013404 GO:0005003 1 9

C0013404 GO:0016676 1 9

C0013404 GO:0005007 1 9

C0013404 GO:0016675 1 9

C0013404 GO:0030880 1 9

C0013404 GO:0008143 1 9

C0013404 GO:0033209 1 9

C0013404 GO:0008144 1 9

C0013404 GO:0031228 1 9

C0013404 GO:0018105 1 9

C0013404 GO:0004693 1 9

C0013404 GO:0031498 1 9

C0013404 GO:0042645 1 9

C0013404 GO:0051457 1 9

C0013404 GO:0009394 1 9

C0013404 GO:0043484 1 9

C0013404 GO:0002228 2 9

C0013404 GO:0045621 1 9

C0013404 GO:0045622 1 9

C0013404 GO:0045749 1 9

C0013404 GO:0033108 1 9

C0013404 GO:0006879 1 9

C0013404 GO:0055008 1 9

C0013404 GO:0002637 1 9

C0013404 GO:0001656 1 9

C0013404 GO:0046889 1 9

C0013404 GO:0044253 1 9

C0013404 GO:0006337 1 9

C0013404 GO:0042542 2 9

C0013404 GO:0004190 1 9

C0013404 GO:0030262 1 9

C0013404 GO:0003899 2 9

C0013404 GO:0045923 1 9

C0013404 GO:0042267 2 9

C0013404 GO:0060021 1 9

C0013404 GO:0006099 1 9

C0013404 GO:0043631 1 9

C0013404 GO:0008603 1 9

C0013404 GO:0006096 1 9

C0013404 GO:0009820 1 9

C0013404 GO:0033993 1 9

C0013404 GO:0003730 2 9

C0013404 GO:0019210 1 9

C0013404 GO:0019213 1 9

C0013404 GO:0045089 1 9

C0013404 GO:0070120 1 9

C0013404 GO:0015711 1 9

C0013404 GO:0000445 1 9

C0013404 GO:0030530 1 9

C0013404 GO:0040017 1 9

C0013404 GO:0030532 1 9

C0013404 GO:0001836 1 9

C0013404 GO:0001837 1 9

C0013404 GO:0005763 1 9

C0013404 GO:0048524 1 9

C0013404 GO:0006413 1 9

C0013404 GO:0019362 1 9

C0013404 GO:0034440 1 9

C0013404 GO:0042133 1 9

C0013404 GO:0007612 1 9

C0013404 GO:0000080 1 9

C0013404 GO:0043449 1 9

C0013404 GO:0043523 1 9

C0013404 GO:0003015 1 9

C0013404 GO:0042765 1 9

C0013404 GO:0016597 2 9

C0013404 GO:0009295 1 9

C0013404 GO:0007004 1 9

C0013404 GO:0042446 1 9

C0013404 GO:0002263 1 9

C0013404 GO:0010257 1 9

C0013404 GO:0001909 1 9

C0013404 GO:0032135 2 9

C0013404 GO:0015002 1 9

C0013404 GO:0033032 1 9

C0013404 GO:0016209 3 9

C0013404 GO:0004385 1 9

C0013404 GO:0016893 1 9

C0013404 GO:0000790 1 9

C0013404 GO:0016891 2 9

C0013404 GO:0000796 1 9

C0013404 GO:0009074 1 9

C0013404 GO:0009071 1 9

C0013404 GO:0030261 2 9

C0013404 GO:0018209 1 9

C0013404 GO:0000018 1 9

C0013404 GO:0016566 1 9

C0013404 GO:0004707 1 9

C0013404 GO:0032967 1 9

C0013404 GO:0016445 1 9

C0013404 GO:0016444 1 9

C0013404 GO:0018024 1 9

C0013404 GO:0006297 2 9

C0013404 GO:0017166 1 9

C0013404 GO:0002839 1 9

C0013404 GO:0015851 1 9

C0013404 GO:0002834 1 9

C0013404 GO:0002837 1 9

C0013404 GO:0002836 1 9

C0013404 GO:0034062 2 9

C0013404 GO:0034061 1 9

C0013404 GO:0015858 1 9

C0013404 GO:0048024 1 9

C0013404 GO:0001763 1 9

C0013404 GO:0046496 1 9

C0013404 GO:0051181 2 9

C0013404 GO:0051180 1 9

C0013404 GO:0042098 1 9

C0013404 GO:0000779 1 9

C0013404 GO:0007183 1 9

C0013404 GO:0032813 1 9

C0013404 GO:0032769 2 9

C0013404 GO:0017015 1 9

C0013404 GO:0019319 2 9

C0013404 GO:0050795 1 9

C0013404 GO:0003231 2 9

C0013404 GO:0030069 2 9

C0013404 GO:0000272 2 9

C0013404 GO:0046148 1 9

C0013404 GO:0005720 1 9

C0013404 GO:0008652 1 9

C0013404 GO:0032368 1 9

C0013404 GO:0032369 1 9

C0013404 GO:0022898 2 9

C0013404 GO:0010714 1 9

C0013404 GO:0010712 1 9

C0013404 GO:0070198 1 9

C0013404 GO:0003746 1 9

C0013404 GO:0006633 1 9

C0013404 GO:0016796 1 9

C0013404 GO:0006636 1 9

C0013404 GO:0006635 1 9

C0013404 GO:0016799 2 9

C0013404 GO:0021510 1 9

C0013404 GO:0001945 1 9

C0013404 GO:0031307 1 9

C0013404 GO:0031306 1 9

C0013404 GO:0000428 1 9

C0013404 GO:0016706 1 9

C0013404 GO:0004527 1 9

C0013404 GO:0004526 1 9

C0013404 GO:0004521 1 9

C0013404 GO:0004520 1 9

C0013404 GO:0015165 1 9

C0013404 GO:0021954 1 9

C0013404 GO:0021953 1 9

C0013404 GO:0032404 3 9

C0013404 GO:0008139 1 9

C0013404 GO:0070301 3 9

C0013404 GO:0031397 1 9

C0013404 GO:0014065 1 9

C0013404 GO:0006595 1 9

C0013404 GO:0043473 1 9

C0013404 GO:0002460 1 9

C0013404 GO:0005513 1 9

C0013404 GO:0009084 2 9

C0013404 GO:0016018 1 9

C0013404 GO:0005104 1 9

C0013404 GO:0004177 2 9

C0013404 GO:0055010 1 9

C0013404 GO:0046365 1 9

C0013404 GO:0046364 2 9

C0013404 GO:0030983 2 9

C0013404 GO:0046040 2 9

C0013404 GO:0009109 1 9

C0013404 GO:0034623 1 9

C0013404 GO:0034235 1 9

C0013404 GO:0050996 1 9

C0013404 GO:0004180 1 9

C0013404 GO:0000060 1 9

C0013404 GO:0005814 1 9

C0013404 GO:0004675 1 9

C0013404 GO:0003205 2 9

C0013404 GO:0005217 1 9

C0013404 GO:0017119 1 9

C0013404 GO:0045939 1 9

C0013404 GO:0031124 1 9

C0013404 GO:0010812 1 9

C0013404 GO:0031123 1 9

C0013404 GO:0022417 1 9

C0013404 GO:0016903 1 9

C0013404 GO:0055067 1 9

C0013404 GO:0007266 1 9

C0013404 GO:0051881 1 9

C0013404 GO:0031571 1 9

C0013404 GO:0032200 1 9

C0013404 GO:0005662 1 9

C0013404 GO:0000387 1 9

C0013404 GO:0045806 1 9

C0013404 GO:0002833 1 9

C0013404 GO:0005786 1 9

C0013404 GO:0006775 1 9

C0013404 GO:0000245 1 9

C0013404 GO:0048469 1 9

C0013404 GO:0006779 1 9

C0013404 GO:0007219 1 9

C0013404 GO:0006783 1 9

C0013404 GO:0007213 1 9

C0013404 GO:0016627 1 9

C0013404 GO:0007216 1 9

C0013404 GO:0016620 2 9

C0013404 GO:0070001 1 9

C0013404 GO:0006073 1 9

C0013404 GO:0005678 1 9

C0013404 GO:0015718 1 9

C0013404 GO:0005092 1 9

C0013404 GO:0006607 1 9

C0013404 GO:0005095 1 9

C0013404 GO:0043535 1 9

C0013404 GO:0019902 1 9

C0013404 GO:0007172 1 9

C0013404 GO:0007076 1 9

C0013404 GO:0008630 1 9

C0013404 GO:0045793 1 9

C0013404 GO:0016471 1 9

C0013404 GO:0004550 2 9

C0013404 GO:0033558 1 9

C0013404 GO:0006672 2 9

C0013404 GO:0045494 1 9

C0013404 GO:0043525 1 9

C0013404 GO:0006921 1 9

C0013404 GO:0016278 1 9

C0013404 GO:0016279 1 9

C0013404 GO:0009062 1 9

C0013404 GO:0016885 1 9

C0013404 GO:0031576 1 9

C0013404 GO:0016289 1 9

C0013404 GO:0005852 4 9

C0013404 GO:0090100 1 9

C0013404 GO:0005851 2 9

C0013404 GO:0051896 1 9

C0013404 GO:0016575 1 9

C0013404 GO:0005528 1 9

C0013404 GO:0005522 1 9

C0013404 GO:0002200 1 9

C0013404 GO:0005527 1 9

C0013404 GO:0004129 1 9

C0013404 GO:0004128 2 9

C0013404 GO:0006818 1 9

C0013404 GO:0055029 1 9

C0013404 GO:0006189 2 9

C0013404 GO:0006188 2 9

C0013404 GO:0001672 1 9

C0013404 GO:0044275 1 9

C0013404 GO:0008091 1 9

C0013404 GO:0004601 2 9

C0013404 GO:0033176 1 9

C0013404 GO:0045580 1 9

C0013404 GO:0045582 1 9

C0013404 GO:0060047 1 9

C0013404 GO:0046677 1 9

C0013404 GO:0003229 1 9

C0013404 GO:0019395 1 9

C0013404 GO:0001953 1 9

C0013404 GO:0060048 1 9

C0013404 GO:0015278 1 9

C0013404 GO:0001707 1 9

C0013404 GO:0001704 1 9

C0013404 GO:0032376 1 9

C0013404 GO:0032370 1 9

C0013404 GO:0032373 1 9

C0013404 GO:0015370 1 9

C0013404 GO:0070761 1 9

C0013404 GO:0017156 1 9

C0013404 GO:0048332 1 9

C0013404 GO:0031330 1 9

C0013404 GO:0031333 1 9

C0013404 GO:0000217 1 9

C0013404 GO:0004860 1 9

C0013404 GO:0030121 1 9

C0013404 GO:0001959 1 9

C0013404 GO:0032182 1 9

C0013404 GO:0001952 1 9

C0013404 GO:0005024 1 9

C0013404 GO:0005744 1 9

C0013404 GO:0016653 1 9

C0013404 GO:0006007 1 9

C0013404 GO:0006000 1 9

C0013404 GO:0034502 1 9

C0013404 GO:0042116 1 9

C0013404 GO:0042113 1 9

C0013404 GO:0048754 1 9

C0013404 GO:0032986 1 9

C0013404 GO:0070822 1 9

C0013404 GO:0032984 1 9

C0013404 GO:0043467 1 9

C0013404 GO:0032981 1 9

C0013404 GO:0048365 1 9

C0013404 GO:0045766 1 9

C0013404 GO:0005005 1 9

C0013404 GO:0046426 1 9

C0013404 GO:0046356 1 9

C0013404 GO:0034614 1 9

C0013404 GO:0002711 1 9

C0013404 GO:0009116 1 9

C0013404 GO:0051646 2 9

C0013404 GO:0070925 1 9

C0013404 GO:0001533 1 9

C0013404 GO:0001516 1 9

C0013404 GO:0019842 1 9

C0013404 GO:0000175 1 9

C0013404 GO:0046457 1 9

C0013404 GO:0046456 1 9

C0013404 GO:0043596 1 9

C0013404 GO:0046519 2 9

C0013404 GO:0016896 1 9

C0013404 GO:0046638 1 9

C0013404 GO:0046637 1 9

C0013404 GO:0050684 1 9

C0013404 GO:0046635 1 9

C0013404 GO:0046634 1 9

C0013404 GO:0055072 1 9

C0013404 GO:0050920 1 9

C0013404 GO:0051262 1 9

C0013404 GO:0051893 1 9

C0013404 GO:0008278 1 9

C0013404 GO:0034762 1 9

C0013404 GO:0043043 1 9

C0013404 GO:0006266 1 9

C0013404 GO:0010894 1 9

C0013404 GO:0006383 1 9

C0013404 GO:0032393 1 9

C0013404 GO:0006760 1 9

C0013404 GO:0000314 1 9

C0013404 GO:0006769 1 9

C0013404 GO:0030165 1 9

C0013404 GO:0000502 1 9

C0013404 GO:0030511 1 9

C0013404 GO:0051354 2 9

C0013404 GO:0005663 2 9

C0013404 GO:0043130 1 9

C0013404 GO:0006164 1 9

C0013404 GO:0060415 1 9

C0013404 GO:0045930 1 9

C0013404 GO:0051453 1 9

C0013404 GO:0042744 2 9

C0013404 GO:0042743 1 9

C0013404 GO:0031369 1 9

C0013404 GO:0008408 2 9

C0013404 GO:0000381 3 9

C0013404 GO:0010810 1 9

C0013404 GO:0019239 1 9

C0013404 GO:0033014 1 9

C0013404 GO:0006684 1 9

C0013404 GO:0016684 2 9

C0013404 GO:0030894 1 9

C0013404 GO:0005337 1 9

C0013404 GO:0009156 2 9

C0013404 GO:0048500 1 9

C0013404 GO:0009152 1 9

C0013404 GO:0042177 1 9

C0013404 GO:0000030 1 9

C0013404 GO:0005844 1 9

C0013404 GO:0000132 1 9

C0013404 GO:0006941 1 9

C0013404 GO:0004683 1 9

C0013404 GO:0031647 1 9

C0013404 GO:0030867 1 9

C0013404 GO:0045736 1 9

C0013404 GO:0005246 1 9

C0013404 GO:0045739 1 9

C0013404 GO:0051087 1 9

C0013404 GO:0030307 1 9

C0013404 GO:0051004 1 9

C0013404 GO:0006942 1 9

C0013404 GO:0048029 1 9

C0013404 GO:0009650 1 9

C0013404 GO:0009127 2 9

C0013404 GO:0044246 1 9

C0013404 GO:0006220 2 9

C0013404 GO:0006221 1 9

C0013404 GO:0002444 1 9

C0013404 GO:0042921 1 9

C0013404 GO:0046784 1 9

C0013404 GO:0044452 1 9

C0013404 GO:0022616 1 9

C0013404 GO:0010939 1 9

C0013404 GO:0046165 1 9

C0013404 GO:0015298 1 9

C0013404 GO:0035035 1 9

C0013404 GO:0006298 1 9

C0013404 GO:0051318 1 9

C0013404 GO:0030004 1 9

C0013404 GO:0010833 1 9

C0013404 GO:0055085 1 9

C0013404 GO:0006084 1 9

C0013404 GO:0008634 1 9

C0013404 GO:0008637 1 9

C0013404 GO:0043601 1 9

C0013404 GO:0006081 1 9

C0013404 GO:0043603 1 9

C0013404 GO:0050921 1 9

C0013404 GO:0070776 1 9

C0013404 GO:0070775 1 9

C0013404 GO:0042813 1 9

C0013404 GO:0003727 1 9

C0013404 GO:0006536 1 9

C0013404 GO:0043370 1 9

C0013404 GO:0000347 1 9

C0013404 GO:0000346 1 9

C0013404 GO:0030641 1 9

C0013404 GO:0042516 1 9

C0013404 GO:0016646 2 9

C0013404 GO:0016645 1 9

C0013404 GO:0008526 1 9

C0013404 GO:0019104 2 9

C0013404 GO:0007159 1 9

C0013404 GO:0006563 1 9

C0013404 GO:0007157 1 9

C0013404 GO:0042772 1 9

C0013404 GO:0002446 1 9

C0013404 GO:0042771 1 9

C0013404 GO:0002562 1 9

C0013404 GO:0016580 1 9

C0013404 GO:0016581 2 9

C0013404 GO:0019047 2 9

C0013404 GO:0001938 1 9

C0013404 GO:0034220 1 9

C0013404 GO:0033344 1 9

C0013404 GO:0002250 1 9

C0013404 GO:0004549 1 9

C0013404 GO:0015030 1 9

C0013404 GO:0001933 1 9

C0013404 GO:0001937 1 9

C0013404 GO:0001936 1 9

C0015967 GO:0022624 1 15

C0015967 GO:0043954 2 15

C0015967 GO:0006906 1 15

C0015967 GO:0048066 1 15

C0015967 GO:0002706 2 15

C0015967 GO:0002703 2 15

C0015967 GO:0001508 1 15

C0015967 GO:0002709 1 15

C0015967 GO:0016860 2 15

C0015967 GO:0005838 1 15

C0015967 GO:0043206 1 15

C0015967 GO:0005834 2 15

C0015967 GO:0006978 1 15

C0015967 GO:0004653 1 15

C0015967 GO:0006309 3 15

C0015967 GO:0007080 1 15

C0015967 GO:0030856 1 15

C0015967 GO:0046504 1 15

C0015967 GO:0051294 1 15

C0015967 GO:0002824 1 15

C0015967 GO:0003923 2 15

C0015967 GO:0002822 2 15

C0015967 GO:0031109 1 15

C0015967 GO:0002821 2 15

C0015967 GO:0035270 1 15

C0015967 GO:0003841 1 15

C0015967 GO:0000718 1 15

C0015967 GO:0006278 1 15

C0015967 GO:0016812 1 15

C0015967 GO:0006271 1 15

C0015967 GO:0045259 1 15

C0015967 GO:0016814 2 15

C0015967 GO:0010887 1 15

C0015967 GO:0010884 1 15

C0015967 GO:0043331 1 15

C0015967 GO:0003995 2 15

C0015967 GO:0006378 1 15

C0015967 GO:0010888 1 15

C0015967 GO:0019320 1 15

C0015967 GO:0009895 1 15

C0015967 GO:0031958 1 15

C0015967 GO:0032412 1 15

C0015967 GO:0048568 2 15

C0015967 GO:0000302 2 15

C0015967 GO:0030174 1 15

C0015967 GO:0030170 1 15

C0015967 GO:0035004 1 15

C0015967 GO:0046651 1 15

C0015967 GO:0005487 1 15

C0015967 GO:0051224 1 15

C0015967 GO:0016944 1 15

C0015967 GO:0016607 2 15

C0015967 GO:0003206 2 15

C0015967 GO:0015992 2 15

C0015967 GO:0015491 1 15

C0015967 GO:0042551 3 15

C0015967 GO:0006885 1 15

C0015967 GO:0042255 1 15

C0015967 GO:0003208 2 15

C0015967 GO:0042558 1 15

C0015967 GO:0042559 1 15

C0015967 GO:0070742 1 15

C0015967 GO:0043028 1 15

C0015967 GO:0005657 1 15

C0015967 GO:0009168 2 15

C0015967 GO:0050792 1 15

C0015967 GO:0043021 2 15

C0015967 GO:0043022 2 15

C0015967 GO:0051879 1 15

C0015967 GO:0043027 1 15

C0015967 GO:0006303 1 15

C0015967 GO:0005338 1 15

C0015967 GO:0009304 3 15

C0015967 GO:0032479 1 15

C0015967 GO:0009303 1 15

C0015967 GO:0033764 1 15

C0015967 GO:0009260 4 15

C0015967 GO:0009262 1 15

C0015967 GO:0009264 1 15

C0015967 GO:0009266 1 15

C0015967 GO:0000002 2 15

C0015967 GO:0035303 1 15

C0015967 GO:0044042 1 15

C0015967 GO:0030675 1 15

C0015967 GO:0031080 2 15

C0015967 GO:0004536 1 15

C0015967 GO:0002039 1 15

C0015967 GO:0015781 1 15

C0015967 GO:0015780 1 15

C0015967 GO:0004532 1 15

C0015967 GO:0006691 1 15

C0015967 GO:0015074 2 15

C0015967 GO:0005003 2 15

C0015967 GO:0005005 2 15

C0015967 GO:0005007 1 15

C0015967 GO:0016675 2 15

C0015967 GO:0006752 1 15

C0015967 GO:0006754 1 15

C0015967 GO:0030880 1 15

C0015967 GO:0008143 1 15

C0015967 GO:0016469 1 15

C0015967 GO:0033209 2 15

C0015967 GO:0008144 2 15

C0015967 GO:0031228 1 15

C0015967 GO:0019047 2 15

C0015967 GO:0004693 1 15

C0015967 GO:0031498 1 15

C0015967 GO:0042645 1 15

C0015967 GO:0051457 1 15

C0015967 GO:0009394 1 15

C0015967 GO:0043484 1 15

C0015967 GO:0045071 1 15

C0015967 GO:0002228 2 15

C0015967 GO:0045621 1 15

C0015967 GO:0045622 1 15

C0015967 GO:0045749 2 15

C0015967 GO:0033108 1 15

C0015967 GO:0006879 1 15

C0015967 GO:0042288 1 15

C0015967 GO:0055008 1 15

C0015967 GO:0002637 1 15

C0015967 GO:0001656 1 15

C0015967 GO:0046889 1 15

C0015967 GO:0044253 2 15

C0015967 GO:0043666 2 15

C0015967 GO:0006337 1 15

C0015967 GO:0042542 2 15

C0015967 GO:0004190 1 15

C0015967 GO:0030262 1 15

C0015967 GO:0003899 2 15

C0015967 GO:0016254 1 15

C0015967 GO:0045923 1 15

C0015967 GO:0018105 1 15

C0015967 GO:0060021 1 15

C0015967 GO:0006099 3 15

C0015967 GO:0043631 1 15

C0015967 GO:0007271 1 15

C0015967 GO:0008603 1 15

C0015967 GO:0006096 1 15

C0015967 GO:0022409 1 15

C0015967 GO:0009820 2 15

C0015967 GO:0033993 1 15

C0015967 GO:0003730 3 15

C0015967 GO:0045814 1 15

C0015967 GO:0019210 1 15

C0015967 GO:0019213 1 15

C0015967 GO:0045089 1 15

C0015967 GO:0045088 1 15

C0015967 GO:0070120 1 15

C0015967 GO:0048593 1 15

C0015967 GO:0015711 1 15

C0015967 GO:0000445 1 15

C0015967 GO:0003746 1 15

C0015967 GO:0030530 1 15

C0015967 GO:0040017 1 15

C0015967 GO:0030532 1 15

C0015967 GO:0001836 1 15

C0015967 GO:0001837 1 15

C0015967 GO:0005765 1 15

C0015967 GO:0005763 1 15

C0015967 GO:0048525 1 15

C0015967 GO:0048524 2 15

C0015967 GO:0006413 1 15

C0015967 GO:0019362 1 15

C0015967 GO:0006633 1 15

C0015967 GO:0042133 1 15

C0015967 GO:0009593 1 15

C0015967 GO:0007612 1 15

C0015967 GO:0000080 1 15

C0015967 GO:0009451 1 15

C0015967 GO:0031264 1 15

C0015967 GO:0007163 1 15

C0015967 GO:0003015 1 15

C0015967 GO:0042765 2 15

C0015967 GO:0016597 3 15

C0015967 GO:0009295 1 15

C0015967 GO:0007004 1 15

C0015967 GO:0042446 1 15

C0015967 GO:0004364 1 15

C0015967 GO:0002263 1 15

C0015967 GO:0010257 1 15

C0015967 GO:0005546 1 15

C0015967 GO:0001909 1 15

C0015967 GO:0032135 2 15

C0015967 GO:0015002 2 15

C0015967 GO:0033032 1 15

C0015967 GO:0016209 3 15

C0015967 GO:0004385 1 15

C0015967 GO:0016893 1 15

C0015967 GO:0000790 1 15

C0015967 GO:0016891 2 15

C0015967 GO:0000796 2 15

C0015967 GO:0009074 1 15

C0015967 GO:0051702 2 15

C0015967 GO:0070279 1 15

C0015967 GO:0009071 1 15

C0015967 GO:0030261 3 15

C0015967 GO:0018209 1 15

C0015967 GO:0016676 2 15

C0015967 GO:0000018 1 15

C0015967 GO:0016566 1 15

C0015967 GO:0004707 1 15

C0015967 GO:0032967 2 15

C0015967 GO:0016445 1 15

C0015967 GO:0016444 1 15

C0015967 GO:0018024 1 15

C0015967 GO:0006297 2 15

C0015967 GO:0017166 1 15

C0015967 GO:0002839 1 15

C0015967 GO:0015851 2 15

C0015967 GO:0002834 1 15

C0015967 GO:0002837 1 15

C0015967 GO:0002836 1 15

C0015967 GO:0034062 2 15

C0015967 GO:0034061 1 15

C0015967 GO:0015858 1 15

C0015967 GO:0048024 1 15

C0015967 GO:0001763 1 15

C0015967 GO:0046496 1 15

C0015967 GO:0005929 1 15

C0015967 GO:0002366 1 15

C0015967 GO:0030280 1 15

C0015967 GO:0051184 1 15

C0015967 GO:0051187 1 15

C0015967 GO:0051181 3 15

C0015967 GO:0051180 1 15

C0015967 GO:0042098 1 15

C0015967 GO:0000779 1 15

C0015967 GO:0007183 1 15

C0015967 GO:0016363 1 15

C0015967 GO:0030149 1 15

C0015967 GO:0032813 1 15

C0015967 GO:0006221 1 15

C0015967 GO:0004004 1 15

C0015967 GO:0000339 1 15

C0015967 GO:0032769 2 15

C0015967 GO:0017015 1 15

C0015967 GO:0019319 3 15

C0015967 GO:0050795 1 15

C0015967 GO:0051896 1 15

C0015967 GO:0051238 1 15

C0015967 GO:0003231 2 15

C0015967 GO:0030069 2 15

C0015967 GO:0000272 2 15

C0015967 GO:0005720 1 15

C0015967 GO:0008652 1 15

C0015967 GO:0008656 1 15

C0015967 GO:0032368 1 15

C0015967 GO:0032369 1 15

C0015967 GO:0022898 2 15

C0015967 GO:0042267 2 15

C0015967 GO:0010712 2 15

C0015967 GO:0070198 1 15

C0015967 GO:0046824 1 15

C0015967 GO:0006518 1 15

C0015967 GO:0019897 1 15

C0015967 GO:0016796 1 15

C0015967 GO:0006636 1 15

C0015967 GO:0006635 1 15

C0015967 GO:0019674 1 15

C0015967 GO:0016799 1 15

C0015967 GO:0021510 1 15

C0015967 GO:0004520 1 15

C0015967 GO:0031306 1 15

C0015967 GO:0000428 1 15

C0015967 GO:0016706 1 15

C0015967 GO:0004527 1 15

C0015967 GO:0004526 1 15

C0015967 GO:0004521 1 15

C0015967 GO:0001945 1 15

C0015967 GO:0050769 1 15

C0015967 GO:0016667 1 15

C0015967 GO:0009925 1 15

C0015967 GO:0021954 1 15

C0015967 GO:0006744 1 15

C0015967 GO:0006743 1 15

C0015967 GO:0021953 1 15

C0015967 GO:0032404 4 15

C0015967 GO:0015165 1 15

C0015967 GO:0008139 1 15

C0015967 GO:0070301 2 15

C0015967 GO:0031397 1 15

C0015967 GO:0014065 1 15

C0015967 GO:0007033 1 15

C0015967 GO:0002460 1 15

C0015967 GO:0008483 1 15

C0015967 GO:0045069 1 15

C0015967 GO:0048167 1 15

C0015967 GO:0005513 1 15

C0015967 GO:0009060 1 15

C0015967 GO:0045619 1 15

C0015967 GO:0009084 1 15

C0015967 GO:0016018 2 15

C0015967 GO:0006740 1 15

C0015967 GO:0005104 1 15

C0015967 GO:0004177 2 15

C0015967 GO:0008484 2 15

C0015967 GO:0055010 2 15

C0015967 GO:0046365 1 15

C0015967 GO:0046364 3 15

C0015967 GO:0030983 2 15

C0015967 GO:0009108 1 15

C0015967 GO:0009109 3 15

C0015967 GO:0034623 1 15

C0015967 GO:0034235 2 15

C0015967 GO:0050996 1 15

C0015967 GO:0016840 1 15

C0015967 GO:0031532 1 15

C0015967 GO:0004180 1 15

C0015967 GO:0000060 2 15

C0015967 GO:0005814 1 15

C0015967 GO:0004675 1 15

C0015967 GO:0016248 1 15

C0015967 GO:0016246 1 15

C0015967 GO:0003205 2 15

C0015967 GO:0005217 1 15

C0015967 GO:0045939 1 15

C0015967 GO:0031124 1 15

C0015967 GO:0010812 1 15

C0015967 GO:0031123 1 15

C0015967 GO:0022417 2 15

C0015967 GO:0016903 2 15

C0015967 GO:0055067 1 15

C0015967 GO:0007266 2 15

C0015967 GO:0003709 1 15

C0015967 GO:0042346 1 15

C0015967 GO:0042345 1 15

C0015967 GO:0002793 1 15

C0015967 GO:0032200 1 15

C0015967 GO:0070652 1 15

C0015967 GO:0000387 1 15

C0015967 GO:0006390 1 15

C0015967 GO:0045806 1 15

C0015967 GO:0000149 1 15

C0015967 GO:0002833 1 15

C0015967 GO:0005786 2 15

C0015967 GO:0006775 1 15

C0015967 GO:0000245 1 15

C0015967 GO:0034440 1 15

C0015967 GO:0006471 1 15

C0015967 GO:0048469 2 15

C0015967 GO:0005788 1 15

C0015967 GO:0031050 1 15

C0015967 GO:0031579 1 15

C0015967 GO:0005391 1 15

C0015967 GO:0048863 1 15

C0015967 GO:0002687 1 15

C0015967 GO:0016627 2 15

C0015967 GO:0007216 1 15

C0015967 GO:0016620 3 15

C0015967 GO:0070001 1 15

C0015967 GO:0002685 1 15

C0015967 GO:0006073 1 15

C0015967 GO:0005678 1 15

C0015967 GO:0015718 2 15

C0015967 GO:0043473 2 15

C0015967 GO:0005092 1 15

C0015967 GO:0006607 1 15

C0015967 GO:0005095 2 15

C0015967 GO:0043535 1 15

C0015967 GO:0019902 1 15

C0015967 GO:0019905 1 15

C0015967 GO:0007172 1 15

C0015967 GO:0007076 2 15

C0015967 GO:0055117 1 15

C0015967 GO:0006081 3 15

C0015967 GO:0045793 1 15

C0015967 GO:0043603 1 15

C0015967 GO:0050732 1 15

C0015967 GO:0043449 1 15

C0015967 GO:0005527 2 15

C0015967 GO:0033135 1 15

C0015967 GO:0002377 1 15

C0015967 GO:0043523 1 15

C0015967 GO:0004550 2 15

C0015967 GO:0033558 1 15

C0015967 GO:0006672 2 15

C0015967 GO:0045494 2 15

C0015967 GO:0043525 1 15

C0015967 GO:0006921 2 15

C0015967 GO:0009142 1 15

C0015967 GO:0009145 1 15

C0015967 GO:0016278 1 15

C0015967 GO:0016279 1 15

C0015967 GO:0009062 2 15

C0015967 GO:0000783 1 15

C0015967 GO:0000782 1 15

C0015967 GO:0016885 1 15

C0015967 GO:0009066 1 15

C0015967 GO:0009065 1 15

C0015967 GO:0048168 1 15

C0015967 GO:0070918 1 15

C0015967 GO:0031576 1 15

C0015967 GO:0042625 1 15

C0015967 GO:0016289 1 15

C0015967 GO:0005852 3 15

C0015967 GO:0090100 1 15

C0015967 GO:0005851 2 15

C0015967 GO:0043548 2 15

C0015967 GO:0007006 1 15

C0015967 GO:0016575 1 15

C0015967 GO:0005528 2 15

C0015967 GO:0016571 1 15

C0015967 GO:0005522 1 15

C0015967 GO:0002200 1 15

C0015967 GO:0016471 1 15

C0015967 GO:0004129 2 15

C0015967 GO:0004128 1 15

C0015967 GO:0006818 1 15

C0015967 GO:0015908 1 15

C0015967 GO:0055029 1 15

C0015967 GO:0016303 1 15

C0015967 GO:0006189 2 15

C0015967 GO:0006188 2 15

C0015967 GO:0001672 1 15

C0015967 GO:0044275 3 15

C0015967 GO:0008091 1 15

C0015967 GO:0000737 1 15

C0015967 GO:0016831 1 15

C0015967 GO:0006739 1 15

C0015967 GO:0004601 3 15

C0015967 GO:0033176 1 15

C0015967 GO:0010921 1 15

C0015967 GO:0045055 1 15

C0015967 GO:0042772 1 15

C0015967 GO:0045580 3 15

C0015967 GO:0045582 1 15

C0015967 GO:0060047 1 15

C0015967 GO:0046677 1 15

C0015967 GO:0003229 2 15

C0015967 GO:0019395 1 15

C0015967 GO:0003950 1 15

C0015967 GO:0042572 1 15

C0015967 GO:0060048 1 15

C0015967 GO:0045426 1 15

C0015967 GO:0030018 1 15

C0015967 GO:0034399 1 15

C0015967 GO:0015278 1 15

C0015967 GO:0001707 1 15

C0015967 GO:0001704 1 15

C0015967 GO:0032376 1 15

C0015967 GO:0032370 1 15

C0015967 GO:0032373 1 15

C0015967 GO:0042375 1 15

C0015967 GO:0015370 1 15

C0015967 GO:0006505 1 15

C0015967 GO:0070761 1 15

C0015967 GO:0006506 1 15

C0015967 GO:0017156 1 15

C0015967 GO:0043094 1 15

C0015967 GO:0048332 1 15

C0015967 GO:0031330 1 15

C0015967 GO:0031333 1 15

C0015967 GO:0000217 2 15

C0015967 GO:0004860 1 15

C0015967 GO:0000738 1 15

C0015967 GO:0030121 1 15

C0015967 GO:0001959 2 15

C0015967 GO:0032182 1 15

C0015967 GO:0001952 1 15

C0015967 GO:0005024 1 15

C0015967 GO:0005742 1 15

C0015967 GO:0005744 1 15

C0015967 GO:0032570 1 15

C0015967 GO:0046040 2 15

C0015967 GO:0006007 1 15

C0015967 GO:0006000 1 15

C0015967 GO:0034502 1 15

C0015967 GO:0009206 1 15

C0015967 GO:0042116 1 15

C0015967 GO:0035095 1 15

C0015967 GO:0042113 1 15

C0015967 GO:0048754 1 15

C0015967 GO:0005890 1 15

C0015967 GO:0043506 1 15

C0015967 GO:0032986 1 15

C0015967 GO:0070822 2 15

C0015967 GO:0032984 1 15

C0015967 GO:0043467 1 15

C0015967 GO:0032981 1 15

C0015967 GO:0045766 1 15

C0015967 GO:0006695 1 15

C0015967 GO:0046426 2 15

C0015967 GO:0046356 3 15

C0015967 GO:0007585 1 15

C0015967 GO:0002711 1 15

C0015967 GO:0009110 1 15

C0015967 GO:0009116 1 15

C0015967 GO:0051646 2 15

C0015967 GO:0070925 1 15

C0015967 GO:0001533 1 15

C0015967 GO:0000178 2 15

C0015967 GO:0001516 1 15

C0015967 GO:0019842 1 15

C0015967 GO:0000175 1 15

C0015967 GO:0046457 1 15

C0015967 GO:0046456 1 15

C0015967 GO:0043596 2 15

C0015967 GO:0004602 1 15

C0015967 GO:0046519 2 15

C0015967 GO:0016896 1 15

C0015967 GO:0031571 1 15

C0015967 GO:0046638 1 15

C0015967 GO:0046637 1 15

C0015967 GO:0050684 1 15

C0015967 GO:0046635 1 15

C0015967 GO:0046634 1 15

C0015967 GO:0055072 1 15

C0015967 GO:0050920 1 15

C0015967 GO:0016645 1 15

C0015967 GO:0045309 1 15

C0015967 GO:0051893 1 15

C0015967 GO:0008278 1 15

C0015967 GO:0034762 1 15

C0015967 GO:0033628 1 15

C0015967 GO:0043043 1 15

C0015967 GO:0015238 1 15

C0015967 GO:0006266 3 15

C0015967 GO:0010894 1 15

C0015967 GO:0010896 1 15

C0015967 GO:0006383 1 15

C0015967 GO:0032393 2 15

C0015967 GO:0007219 1 15

C0015967 GO:0006760 1 15

C0015967 GO:0009201 1 15

C0015967 GO:0000314 1 15

C0015967 GO:0006769 1 15

C0015967 GO:0030165 1 15

C0015967 GO:0010975 1 15

C0015967 GO:0000502 1 15

C0015967 GO:0030515 1 15

C0015967 GO:0030511 1 15

C0015967 GO:0045109 1 15

C0015967 GO:0005062 1 15

C0015967 GO:0051354 2 15

C0015967 GO:0005663 2 15

C0015967 GO:0043130 1 15

C0015967 GO:0006164 1 15

C0015967 GO:0060415 1 15

C0015967 GO:0045930 2 15

C0015967 GO:0005086 1 15

C0015967 GO:0051453 1 15

C0015967 GO:0042744 1 15

C0015967 GO:0042743 1 15

C0015967 GO:0031369 2 15

C0015967 GO:0008408 1 15

C0015967 GO:0000381 3 15

C0015967 GO:0008235 1 15

C0015967 GO:0035085 1 15

C0015967 GO:0010810 1 15

C0015967 GO:0019239 3 15

C0015967 GO:0005662 1 15

C0015967 GO:0016684 3 15

C0015967 GO:0016769 1 15

C0015967 GO:0030894 1 15

C0015967 GO:0005337 1 15

C0015967 GO:0009156 3 15

C0015967 GO:0048500 2 15

C0015967 GO:0009152 2 15

C0015967 GO:0042177 1 15

C0015967 GO:0048256 2 15

C0015967 GO:0043255 1 15

C0015967 GO:0000030 1 15

C0015967 GO:0005844 1 15

C0015967 GO:0000132 1 15

C0015967 GO:0006941 1 15

C0015967 GO:0004683 1 15

C0015967 GO:0031647 1 15

C0015967 GO:0030867 3 15

C0015967 GO:0045730 1 15

C0015967 GO:0045736 2 15

C0015967 GO:0005246 1 15

C0015967 GO:0045739 1 15

C0015967 GO:0051087 2 15

C0015967 GO:0030307 1 15

C0015967 GO:0002819 1 15

C0015967 GO:0045178 1 15

C0015967 GO:0051004 1 15

C0015967 GO:0006942 1 15

C0015967 GO:0048029 1 15

C0015967 GO:0009650 2 15

C0015967 GO:0043087 1 15

C0015967 GO:0009127 2 15

C0015967 GO:0044246 2 15

C0015967 GO:0006220 1 15

C0015967 GO:0010883 1 15

C0015967 GO:0006342 1 15

C0015967 GO:0042921 1 15

C0015967 GO:0046784 1 15

C0015967 GO:0046782 1 15

C0015967 GO:0044452 2 15

C0015967 GO:0046466 1 15

C0015967 GO:0022616 2 15

C0015967 GO:0001953 1 15

C0015967 GO:0010939 1 15

C0015967 GO:0019079 1 15

C0015967 GO:0046165 1 15

C0015967 GO:0015298 1 15

C0015967 GO:0035035 1 15

C0015967 GO:0006298 1 15

C0015967 GO:0051318 1 15

C0015967 GO:0030004 1 15

C0015967 GO:0010833 2 15

C0015967 GO:0051310 1 15

C0015967 GO:0008200 1 15

C0015967 GO:0055085 1 15

C0015967 GO:0051881 1 15

C0015967 GO:0006084 3 15

C0015967 GO:0008634 2 15

C0015967 GO:0008637 2 15

C0015967 GO:0005507 1 15

C0015967 GO:0043601 1 15

C0015967 GO:0051096 1 15

C0015967 GO:0051095 1 15

C0015967 GO:0070776 1 15

C0015967 GO:0070775 1 15

C0015967 GO:0042813 1 15

C0015967 GO:0003727 1 15

C0015967 GO:0006536 1 15

C0015967 GO:0043370 1 15

C0015967 GO:0034976 1 15

C0015967 GO:0019206 1 15

C0015967 GO:0008630 1 15

C0015967 GO:0010948 1 15

C0015967 GO:0000347 1 15

C0015967 GO:0000346 1 15

C0015967 GO:0030641 1 15

C0015967 GO:0032405 1 15

C0015967 GO:0042516 1 15

C0015967 GO:0016646 2 15

C0015967 GO:0051262 1 15

C0015967 GO:0008526 2 15

C0015967 GO:0005753 1 15

C0015967 GO:0006400 1 15

C0015967 GO:0048531 1 15

C0015967 GO:0034451 1 15

C0015967 GO:0019104 2 15

C0015967 GO:0006359 1 15

C0015967 GO:0007159 1 15

C0015967 GO:0002440 1 15

C0015967 GO:0006563 2 15

C0015967 GO:0007157 1 15

C0015967 GO:0002444 3 15

C0015967 GO:0002446 3 15

C0015967 GO:0042771 1 15

C0015967 GO:0002562 1 15

C0015967 GO:0016580 2 15

C0015967 GO:0016581 2 15

C0015967 GO:0010714 2 15

C0015967 GO:0050921 1 15

C0015967 GO:0001938 1 15

C0015967 GO:0034220 1 15

C0015967 GO:0033344 1 15

C0015967 GO:0002250 1 15

C0015967 GO:0004549 1 15

C0015967 GO:0001933 2 15

C0015967 GO:0001936 2 15

C0015967 GO:0001937 1 15

C0015967 GO:0015036 1 15

C0020538 GO:0032432 1 11

C0020538 GO:0051043 1 11

C0020538 GO:0007598 1 11

C0020538 GO:0022624 1 11

C0020538 GO:0006901 1 11

C0020538 GO:0006900 1 11

C0020538 GO:0048066 1 11

C0020538 GO:0002706 1 11

C0020538 GO:0002703 1 11

C0020538 GO:0009161 1 11

C0020538 GO:0016864 1 11

C0020538 GO:0016862 1 11

C0020538 GO:0002709 1 11

C0020538 GO:0016860 1 11

C0020538 GO:0005838 1 11

C0020538 GO:0003158 1 11

C0020538 GO:0004653 1 11

C0020538 GO:0001964 1 11

C0020538 GO:0018210 1 11

C0020538 GO:0004712 1 11

C0020538 GO:0016514 1 11

C0020538 GO:0016101 1 11

C0020538 GO:0016455 1 11

C0020538 GO:0051294 1 11

C0020538 GO:0002824 1 11

C0020538 GO:0002822 1 11

C0020538 GO:0031109 1 11

C0020538 GO:0002821 1 11

C0020538 GO:0035270 1 11

C0020538 GO:0003841 2 11

C0020538 GO:0035272 1 11

C0020538 GO:0006903 1 11

C0020538 GO:0006278 2 11

C0020538 GO:0031050 1 11

C0020538 GO:0006271 1 11

C0020538 GO:0010887 2 11

C0020538 GO:0010885 1 11

C0020538 GO:0006376 1 11

C0020538 GO:0003995 1 11

C0020538 GO:0021537 1 11

C0020538 GO:0031958 2 11

C0020538 GO:0032182 1 11

C0020538 GO:0030174 1 11

C0020538 GO:0016944 1 11

C0020538 GO:0030073 1 11

C0020538 GO:0016607 2 11

C0020538 GO:0016605 1 11

C0020538 GO:0015491 1 11

C0020538 GO:0042551 1 11

C0020538 GO:0016126 1 11

C0020538 GO:0042255 2 11

C0020538 GO:0042558 1 11

C0020538 GO:0051650 1 11

C0020538 GO:0070742 1 11

C0020538 GO:0043028 1 11

C0020538 GO:0005657 1 11

C0020538 GO:0009168 3 11

C0020538 GO:0000339 1 11

C0020538 GO:0043021 1 11

C0020538 GO:0043022 1 11

C0020538 GO:0007622 1 11

C0020538 GO:0009309 1 11

C0020538 GO:0006626 1 11

C0020538 GO:0032479 1 11

C0020538 GO:0048709 1 11

C0020538 GO:0009303 1 11

C0020538 GO:0033764 1 11

C0020538 GO:0009260 2 11

C0020538 GO:0007052 1 11

C0020538 GO:0030898 1 11

C0020538 GO:0006303 1 11

C0020538 GO:0044042 1 11

C0020538 GO:0031080 2 11

C0020538 GO:0004536 1 11

C0020538 GO:0002039 1 11

C0020538 GO:0015074 1 11

C0020538 GO:0005003 1 11

C0020538 GO:0005005 1 11

C0020538 GO:0015179 1 11

C0020538 GO:0015175 1 11

C0020538 GO:0006752 1 11

C0020538 GO:0005343 1 11

C0020538 GO:0008143 1 11

C0020538 GO:0033209 1 11

C0020538 GO:0019047 1 11

C0020538 GO:0004693 1 11

C0020538 GO:0004691 1 11

C0020538 GO:0004690 1 11

C0020538 GO:0043484 1 11

C0020538 GO:0045178 1 11

C0020538 GO:0030705 2 11

C0020538 GO:0016417 1 11

C0020538 GO:0002228 2 11

C0020538 GO:0045621 1 11

C0020538 GO:0005507 1 11

C0020538 GO:0045749 2 11

C0020538 GO:0033108 2 11

C0020538 GO:0008287 1 11

C0020538 GO:0042288 1 11

C0020538 GO:0043550 1 11

C0020538 GO:0034637 1 11

C0020538 GO:0005913 1 11

C0020538 GO:0001656 2 11

C0020538 GO:0046504 1 11

C0020538 GO:0006978 1 11

C0020538 GO:0005786 2 11

C0020538 GO:0010718 1 11

C0020538 GO:0043982 1 11

C0020538 GO:0016528 1 11

C0020538 GO:0016529 1 11

C0020538 GO:0003899 1 11

C0020538 GO:0017046 1 11

C0020538 GO:0045923 1 11

C0020538 GO:0018105 1 11

C0020538 GO:0060021 1 11

C0020538 GO:0008213 1 11

C0020538 GO:0001889 1 11

C0020538 GO:0015184 1 11

C0020538 GO:0007270 1 11

C0020538 GO:0045444 1 11

C0020538 GO:0006090 1 11

C0020538 GO:0022409 1 11

C0020538 GO:0001725 1 11

C0020538 GO:0045446 1 11

C0020538 GO:0009820 1 11

C0020538 GO:0043189 1 11

C0020538 GO:0003730 2 11

C0020538 GO:0045814 1 11

C0020538 GO:0019213 1 11

C0020538 GO:0045088 1 11

C0020538 GO:0070120 2 11

C0020538 GO:0019218 1 11

C0020538 GO:0048593 2 11

C0020538 GO:0000090 1 11

C0020538 GO:0031047 1 11

C0020538 GO:0000445 2 11

C0020538 GO:0030530 1 11

C0020538 GO:0040017 1 11

C0020538 GO:0030532 2 11

C0020538 GO:0035194 1 11

C0020538 GO:0032981 2 11

C0020538 GO:0043535 1 11

C0020538 GO:0019902 1 11

C0020538 GO:0048525 1 11

C0020538 GO:0048524 1 11

C0020538 GO:0006413 1 11

C0020538 GO:0006144 1 11

C0020538 GO:0019363 1 11

C0020538 GO:0019362 2 11

C0020538 GO:0031397 1 11

C0020538 GO:0000086 1 11

C0020538 GO:0007612 1 11

C0020538 GO:0000080 1 11

C0020538 GO:0007162 1 11

C0020538 GO:0043523 1 11

C0020538 GO:0043525 2 11

C0020538 GO:0031593 1 11

C0020538 GO:0007006 1 11

C0020538 GO:0007004 2 11

C0020538 GO:0002263 1 11

C0020538 GO:0010257 2 11

C0020538 GO:0045667 1 11

C0020538 GO:0001909 1 11

C0020538 GO:0032135 2 11

C0020538 GO:0045669 1 11

C0020538 GO:0033032 3 11

C0020538 GO:0009167 1 11

C0020538 GO:0051119 1 11

C0020538 GO:0004385 2 11

C0020538 GO:0000792 1 11

C0020538 GO:0016893 2 11

C0020538 GO:0000790 1 11

C0020538 GO:0016891 3 11

C0020538 GO:0000796 1 11

C0020538 GO:0042992 1 11

C0020538 GO:0042993 1 11

C0020538 GO:0042990 1 11

C0020538 GO:0018209 2 11

C0020538 GO:0000159 1 11

C0020538 GO:0000018 2 11

C0020538 GO:0016291 1 11

C0020538 GO:0016441 1 11

C0020538 GO:0005388 1 11

C0020538 GO:0016445 1 11

C0020538 GO:0004708 1 11

C0020538 GO:0006297 1 11

C0020538 GO:0002839 1 11

C0020538 GO:0015851 2 11

C0020538 GO:0002834 1 11

C0020538 GO:0002837 1 11

C0020538 GO:0002836 1 11

C0020538 GO:0034062 1 11

C0020538 GO:0034061 1 11

C0020538 GO:0008250 1 11

C0020538 GO:0050820 1 11

C0020538 GO:0001763 1 11

C0020538 GO:0046496 2 11

C0020538 GO:0005929 1 11

C0020538 GO:0006563 2 11

C0020538 GO:0016801 1 11

C0020538 GO:0043087 1 11

C0020538 GO:0016363 2 11

C0020538 GO:0030148 1 11

C0020538 GO:0032813 1 11

C0020538 GO:0050792 1 11

C0020538 GO:0017015 1 11

C0020538 GO:0019319 1 11

C0020538 GO:0050795 1 11

C0020538 GO:0031163 1 11

C0020538 GO:0030069 1 11

C0020538 GO:0046660 1 11

C0020538 GO:0005720 1 11

C0020538 GO:0006890 1 11

C0020538 GO:0008656 1 11

C0020538 GO:0002711 1 11

C0020538 GO:0010717 1 11

C0020538 GO:0043983 1 11

C0020538 GO:0042267 2 11

C0020538 GO:0043984 1 11

C0020538 GO:0046823 1 11

C0020538 GO:0010984 1 11

C0020538 GO:0016790 1 11

C0020538 GO:0048469 1 11

C0020538 GO:0006733 2 11

C0020538 GO:0042401 1 11

C0020538 GO:0031306 1 11

C0020538 GO:0007043 1 11

C0020538 GO:0007041 1 11

C0020538 GO:0000428 1 11

C0020538 GO:0030665 1 11

C0020538 GO:0030660 1 11

C0020538 GO:0030663 1 11

C0020538 GO:0016706 1 11

C0020538 GO:0004526 1 11

C0020538 GO:0004521 2 11

C0020538 GO:0015166 1 11

C0020538 GO:0048568 1 11

C0020538 GO:0021954 2 11

C0020538 GO:0021953 2 11

C0020538 GO:0032404 2 11

C0020538 GO:0005355 1 11

C0020538 GO:0042169 1 11

C0020538 GO:0008034 1 11

C0020538 GO:0008139 1 11

C0020538 GO:0042162 1 11

C0020538 GO:0048199 1 11

C0020538 GO:0046467 1 11

C0020538 GO:0014065 2 11

C0020538 GO:0043473 1 11

C0020538 GO:0006479 1 11

C0020538 GO:0045069 1 11

C0020538 GO:0009084 1 11

C0020538 GO:0016018 4 11

C0020538 GO:0006740 1 11

C0020538 GO:0004177 2 11

C0020538 GO:0016331 1 11

C0020538 GO:0046364 2 11

C0020538 GO:0030983 2 11

C0020538 GO:0046040 3 11

C0020538 GO:0009109 1 11

C0020538 GO:0001523 1 11

C0020538 GO:0016846 1 11

C0020538 GO:0016840 1 11

C0020538 GO:0005814 1 11

C0020538 GO:0004683 1 11

C0020538 GO:0016248 1 11

C0020538 GO:0016246 1 11

C0020538 GO:0018279 1 11

C0020538 GO:0045930 2 11

C0020538 GO:0010810 2 11

C0020538 GO:0010812 2 11

C0020538 GO:0022410 1 11

C0020538 GO:0003709 1 11

C0020538 GO:0051881 1 11

C0020538 GO:0042345 1 11

C0020538 GO:0032200 1 11

C0020538 GO:0008290 1 11

C0020538 GO:0002833 1 11

C0020538 GO:0006776 1 11

C0020538 GO:0000245 1 11

C0020538 GO:0009218 1 11

C0020538 GO:0031970 1 11

C0020538 GO:0032387 2 11

C0020538 GO:0021510 2 11

C0020538 GO:0005788 1 11

C0020538 GO:0030509 1 11

C0020538 GO:0048863 1 11

C0020538 GO:0002687 2 11

C0020538 GO:0048512 1 11

C0020538 GO:0002685 2 11

C0020538 GO:0006073 1 11

C0020538 GO:0051287 1 11

C0020538 GO:0009304 1 11

C0020538 GO:0019359 1 11

C0020538 GO:0005678 1 11

C0020538 GO:0000099 1 11

C0020538 GO:0005092 1 11

C0020538 GO:0006607 2 11

C0020538 GO:0005095 1 11

C0020538 GO:0031274 1 11

C0020538 GO:0019903 1 11

C0020538 GO:0045187 1 11

C0020538 GO:0007172 1 11

C0020538 GO:0031272 1 11

C0020538 GO:0007076 1 11

C0020538 GO:0055117 1 11

C0020538 GO:0045793 1 11

C0020538 GO:0002460 1 11

C0020538 GO:0050732 1 11

C0020538 GO:0031264 1 11

C0020538 GO:0005527 1 11

C0020538 GO:0033135 1 11

C0020538 GO:0006672 1 11

C0020538 GO:0050921 1 11

C0020538 GO:0031647 1 11

C0020538 GO:0042398 1 11

C0020538 GO:0034379 1 11

C0020538 GO:0070776 1 11

C0020538 GO:0016885 2 11

C0020538 GO:0021700 1 11

C0020538 GO:0070918 1 11

C0020538 GO:0042625 1 11

C0020538 GO:0016289 1 11

C0020538 GO:0005852 3 11

C0020538 GO:0090100 1 11

C0020538 GO:0005851 1 11

C0020538 GO:0043548 2 11

C0020538 GO:0031579 1 11

C0020538 GO:0016574 1 11

C0020538 GO:0005528 1 11

C0020538 GO:0016571 1 11

C0020538 GO:0046520 1 11

C0020538 GO:0002200 1 11

C0020538 GO:0016471 1 11

C0020538 GO:0004128 1 11

C0020538 GO:0055029 1 11

C0020538 GO:0006189 3 11

C0020538 GO:0006188 3 11

C0020538 GO:0042308 1 11

C0020538 GO:0042307 1 11

C0020538 GO:0042306 2 11

C0020538 GO:0001672 1 11

C0020538 GO:0001570 1 11

C0020538 GO:0010155 1 11

C0020538 GO:0050810 1 11

C0020538 GO:0006359 1 11

C0020538 GO:0033176 1 11

C0020538 GO:0018196 1 11

C0020538 GO:0030159 1 11

C0020538 GO:0005545 1 11

C0020538 GO:0045580 1 11

C0020538 GO:0045582 2 11

C0020538 GO:0046677 1 11

C0020538 GO:0034399 2 11

C0020538 GO:0042274 1 11

C0020538 GO:0051087 1 11

C0020538 GO:0003756 1 11

C0020538 GO:0032272 1 11

C0020538 GO:0070688 1 11

C0020538 GO:0008603 1 11

C0020538 GO:0017156 2 11

C0020538 GO:0006721 1 11

C0020538 GO:0031333 1 11

C0020538 GO:0000217 1 11

C0020538 GO:0004860 1 11

C0020538 GO:0030126 1 11

C0020538 GO:0015884 1 11

C0020538 GO:0001953 2 11

C0020538 GO:0001952 2 11

C0020538 GO:0001950 1 11

C0020538 GO:0005744 1 11

C0020538 GO:0016653 2 11

C0020538 GO:0010770 1 11

C0020538 GO:0006000 1 11

C0020538 GO:0048200 1 11

C0020538 GO:0034508 1 11

C0020538 GO:0048205 1 11

C0020538 GO:0048754 1 11

C0020538 GO:0070822 1 11

C0020538 GO:0070567 2 11

C0020538 GO:0007029 1 11

C0020538 GO:0043967 1 11

C0020538 GO:0045766 1 11

C0020538 GO:0016229 1 11

C0020538 GO:0030330 1 11

C0020538 GO:0016226 1 11

C0020538 GO:0000779 2 11

C0020538 GO:0009110 1 11

C0020538 GO:0009116 1 11

C0020538 GO:0002718 1 11

C0020538 GO:0000178 1 11

C0020538 GO:0001516 1 11

C0020538 GO:0046457 1 11

C0020538 GO:0046456 1 11

C0020538 GO:0043596 1 11

C0020538 GO:0030900 1 11

C0020538 GO:0046519 1 11

C0020538 GO:0016504 1 11

C0020538 GO:0016505 1 11

C0020538 GO:0007090 1 11

C0020538 GO:0046638 2 11

C0020538 GO:0046637 1 11

C0020538 GO:0050684 1 11

C0020538 GO:0046635 1 11

C0020538 GO:0046634 1 11

C0020538 GO:0046631 1 11

C0020538 GO:0008276 1 11

C0020538 GO:0050920 1 11

C0020538 GO:0035267 1 11

C0020538 GO:0051893 1 11

C0020538 GO:0008375 1 11

C0020538 GO:0008376 1 11

C0020538 GO:0030880 1 11

C0020538 GO:0006984 1 11

C0020538 GO:0006983 1 11

C0020538 GO:0005640 1 11

C0020538 GO:0043044 1 11

C0020538 GO:0043043 1 11

C0020538 GO:0009071 1 11

C0020538 GO:0006266 2 11

C0020538 GO:0043325 1 11

C0020538 GO:0010894 1 11

C0020538 GO:0006268 1 11

C0020538 GO:0006760 1 11

C0020538 GO:0005793 1 11

C0020538 GO:0006769 2 11

C0020538 GO:0030165 1 11

C0020538 GO:0000502 1 11

C0020538 GO:0030511 1 11

C0020538 GO:0070585 1 11

C0020538 GO:0005663 1 11

C0020538 GO:0043130 1 11

C0020538 GO:0006164 1 11

C0020538 GO:0005669 1 11

C0020538 GO:0030194 1 11

C0020538 GO:0005123 1 11

C0020538 GO:0005086 1 11

C0020538 GO:0042745 1 11

C0020538 GO:0032947 1 11

C0020538 GO:0043425 1 11

C0020538 GO:0007064 1 11

C0020538 GO:0031369 1 11

C0020538 GO:0042749 1 11

C0020538 GO:0004549 1 11

C0020538 GO:0000381 2 11

C0020538 GO:0035085 1 11

C0020538 GO:0015149 1 11

C0020538 GO:0045939 1 11

C0020538 GO:0019239 2 11

C0020538 GO:0002366 1 11

C0020538 GO:0006684 1 11

C0020538 GO:0016763 1 11

C0020538 GO:0015145 1 11

C0020538 GO:0046545 1 11

C0020538 GO:0009156 3 11

C0020538 GO:0048500 2 11

C0020538 GO:0009152 2 11

C0020538 GO:0008013 1 11

C0020538 GO:0009150 1 11

C0020538 GO:0014003 1 11

C0020538 GO:0043255 1 11

C0020538 GO:0005844 1 11

C0020538 GO:0000132 1 11

C0020538 GO:0043981 1 11

C0020538 GO:0051322 1 11

C0020538 GO:0030867 1 11

C0020538 GO:0045736 1 11

C0020538 GO:0045739 1 11

C0020538 GO:0015665 1 11

C0020538 GO:0030431 1 11

C0020538 GO:0055038 1 11

C0020538 GO:0030307 1 11

C0020538 GO:0055037 1 11

C0020538 GO:0045071 1 11

C0020538 GO:0030261 1 11

C0020538 GO:0048024 1 11

C0020538 GO:0009126 1 11

C0020538 GO:0009127 3 11

C0020538 GO:0006220 1 11

C0020538 GO:0006221 1 11

C0020538 GO:0070603 1 11

C0020538 GO:0042772 1 11

C0020538 GO:0042921 2 11

C0020538 GO:0046784 2 11

C0020538 GO:0046782 1 11

C0020538 GO:0044452 1 11

C0020538 GO:0022612 1 11

C0020538 GO:0022616 2 11

C0020538 GO:0070761 1 11

C0020538 GO:0051646 1 11

C0020538 GO:0046165 1 11

C0020538 GO:0015298 1 11

C0020538 GO:0015804 1 11

C0020538 GO:0010833 2 11

C0020538 GO:0045454 1 11

C0020538 GO:0008200 1 11

C0020538 GO:0045622 1 11

C0020538 GO:0008630 1 11

C0020538 GO:0043603 1 11

C0020538 GO:0007567 1 11

C0020538 GO:0042364 1 11

C0020538 GO:0070775 1 11

C0020538 GO:0003727 3 11

C0020538 GO:0006536 1 11

C0020538 GO:0048009 1 11

C0020538 GO:0045947 1 11

C0020538 GO:0043370 1 11

C0020538 GO:0034976 1 11

C0020538 GO:0019200 1 11

C0020538 GO:0000347 2 11

C0020538 GO:0000346 2 11

C0020538 GO:0030137 1 11

C0020538 GO:0016722 1 11

C0020538 GO:0008329 1 11

C0020538 GO:0008526 2 11

C0020538 GO:0048194 1 11

C0020538 GO:0070325 1 11

C0020538 GO:0000387 1 11

C0020538 GO:0005883 1 11

C0020538 GO:0016049 1 11

C0020538 GO:0002443 1 11

C0020538 GO:0002444 2 11

C0020538 GO:0002446 2 11

C0020538 GO:0042771 1 11

C0020538 GO:0016580 1 11

C0020538 GO:0016581 1 11

C0020538 GO:0001938 1 11

C0020538 GO:0004576 1 11

C0020538 GO:0002250 1 11

C0020538 GO:0004579 1 11

C0020538 GO:0001937 2 11

C0020538 GO:0001936 1 11

C0020649 GO:0006479 1 8

C0020649 GO:0032024 1 8

C0020649 GO:0009264 1 8

C0020649 GO:0042133 1 8

C0020649 GO:0000245 1 8

C0020649 GO:0022624 1 8

C0020649 GO:0060249 1 8

C0020649 GO:0050684 1 8

C0020649 GO:0032387 1 8

C0020649 GO:0048469 1 8

C0020649 GO:0006779 1 8

C0020649 GO:0002706 1 8

C0020649 GO:0043236 1 8

C0020649 GO:0051702 1 8

C0020649 GO:0008376 1 8

C0020649 GO:0002709 2 8

C0020649 GO:0007004 1 8

C0020649 GO:0005548 1 8

C0020649 GO:0070742 2 8

C0020649 GO:0031985 1 8

C0020649 GO:0005838 1 8

C0020649 GO:0045785 1 8

C0020649 GO:0006783 1 8

C0020649 GO:0007213 2 8

C0020649 GO:0007369 1 8

C0020649 GO:0043043 1 8

C0020649 GO:0010257 1 8

C0020649 GO:0005546 1 8

C0020649 GO:0044452 1 8

C0020649 GO:0048512 1 8

C0020649 GO:0004715 1 8

C0020649 GO:0010894 1 8

C0020649 GO:0006733 1 8

C0020649 GO:0034656 1 8

C0020649 GO:0046504 1 8

C0020649 GO:0032135 1 8

C0020649 GO:0005795 1 8

C0020649 GO:0006760 1 8

C0020649 GO:0030330 1 8

C0020649 GO:0051119 1 8

C0020649 GO:0005092 1 8

C0020649 GO:0005310 1 8

C0020649 GO:0002824 2 8

C0020649 GO:0000096 1 8

C0020649 GO:0004385 1 8

C0020649 GO:0006607 1 8

C0020649 GO:0002821 1 8

C0020649 GO:0016893 2 8

C0020649 GO:0003841 1 8

C0020649 GO:0016891 3 8

C0020649 GO:0000796 1 8

C0020649 GO:0007172 1 8

C0020649 GO:0007076 1 8

C0020649 GO:0000502 1 8

C0020649 GO:0005544 1 8

C0020649 GO:0009071 1 8

C0020649 GO:0045793 1 8

C0020649 GO:0006278 1 8

C0020649 GO:0018209 1 8

C0020649 GO:0005062 1 8

C0020649 GO:0034284 1 8

C0020649 GO:0004143 1 8

C0020649 GO:0006271 1 8

C0020649 GO:0016811 1 8

C0020649 GO:0051219 1 8

C0020649 GO:0000018 2 8

C0020649 GO:0016814 1 8

C0020649 GO:0005112 1 8

C0020649 GO:0010887 1 8

C0020649 GO:0005663 1 8

C0020649 GO:0031306 1 8

C0020649 GO:0046849 1 8

C0020649 GO:0006164 2 8

C0020649 GO:0016441 1 8

C0020649 GO:0016445 1 8

C0020649 GO:0043034 1 8

C0020649 GO:0030194 1 8

C0020649 GO:0050811 1 8

C0020649 GO:0006297 1 8

C0020649 GO:0002839 2 8

C0020649 GO:0030299 1 8

C0020649 GO:0017002 1 8

C0020649 GO:0002834 2 8

C0020649 GO:0002837 2 8

C0020649 GO:0002836 2 8

C0020649 GO:0034062 1 8

C0020649 GO:0034061 1 8

C0020649 GO:0005086 1 8

C0020649 GO:0004693 1 8

C0020649 GO:0042745 1 8

C0020649 GO:0001763 1 8

C0020649 GO:0016885 2 8

C0020649 GO:0046496 1 8

C0020649 GO:0016944 1 8

C0020649 GO:0042749 1 8

C0020649 GO:0045088 1 8

C0020649 GO:0016607 2 8

C0020649 GO:0016289 1 8

C0020649 GO:0000381 1 8

C0020649 GO:0015491 1 8

C0020649 GO:0005851 1 8

C0020649 GO:0004521 2 8

C0020649 GO:0010149 1 8

C0020649 GO:0035085 1 8

C0020649 GO:0015149 1 8

C0020649 GO:0016574 1 8

C0020649 GO:0019239 2 8

C0020649 GO:0033014 1 8

C0020649 GO:0009168 1 8

C0020649 GO:0006684 1 8

C0020649 GO:0002200 1 8

C0020649 GO:0016363 1 8

C0020649 GO:0015145 1 8

C0020649 GO:0006303 1 8

C0020649 GO:0004128 1 8

C0020649 GO:0030894 1 8

C0020649 GO:0032813 2 8

C0020649 GO:0007622 1 8

C0020649 GO:0016605 1 8

C0020649 GO:0009156 2 8

C0020649 GO:0009304 1 8

C0020649 GO:0032479 1 8

C0020649 GO:0019319 1 8

C0020649 GO:0008013 1 8

C0020649 GO:0009152 1 8

C0020649 GO:0043410 1 8

C0020649 GO:0009260 2 8

C0020649 GO:0007052 1 8

C0020649 GO:0009262 1 8

C0020649 GO:0006189 1 8

C0020649 GO:0006188 1 8

C0020649 GO:0003899 1 8

C0020649 GO:0034655 1 8

C0020649 GO:0030069 1 8

C0020649 GO:0034341 1 8

C0020649 GO:0001672 1 8

C0020649 GO:0046148 1 8

C0020649 GO:0005720 1 8

C0020649 GO:0005844 1 8

C0020649 GO:0031050 1 8

C0020649 GO:0050885 1 8

C0020649 GO:0031080 1 8

C0020649 GO:0004683 2 8

C0020649 GO:0043982 1 8

C0020649 GO:0032731 1 8

C0020649 GO:0042267 1 8

C0020649 GO:0006474 1 8

C0020649 GO:0050892 1 8

C0020649 GO:0032088 1 8

C0020649 GO:0015074 1 8

C0020649 GO:0048365 1 8

C0020649 GO:0006029 1 8

C0020649 GO:0015175 1 8

C0020649 GO:0045739 1 8

C0020649 GO:0006024 1 8

C0020649 GO:0030431 1 8

C0020649 GO:0032107 1 8

C0020649 GO:0032104 1 8

C0020649 GO:0043484 1 8

C0020649 GO:0042054 1 8

C0020649 GO:0005088 1 8

C0020649 GO:0030307 1 8

C0020649 GO:0033209 1 8

C0020649 GO:0016799 1 8

C0020649 GO:0030261 1 8

C0020649 GO:0031307 1 8

C0020649 GO:0018105 1 8

C0020649 GO:0007043 1 8

C0020649 GO:0050718 1 8

C0020649 GO:0048024 1 8

C0020649 GO:0044241 1 8

C0020649 GO:0043087 1 8

C0020649 GO:0009127 1 8

C0020649 GO:0006220 1 8

C0020649 GO:0009394 1 8

C0020649 GO:0030203 1 8

C0020649 GO:0045058 1 8

C0020649 GO:0034399 1 8

C0020649 GO:0004526 1 8

C0020649 GO:0030669 1 8

C0020649 GO:0006266 1 8

C0020649 GO:0002228 1 8

C0020649 GO:0033135 2 8

C0020649 GO:0021954 1 8

C0020649 GO:0042771 1 8

C0020649 GO:0022616 3 8

C0020649 GO:0021953 1 8

C0020649 GO:0045749 1 8

C0020649 GO:0005355 1 8

C0020649 GO:0033108 1 8

C0020649 GO:0008139 1 8

C0020649 GO:0006721 1 8

C0020649 GO:0019048 1 8

C0020649 GO:0070301 1 8

C0020649 GO:0015298 1 8

C0020649 GO:0016278 1 8

C0020649 GO:0004860 1 8

C0020649 GO:0010833 1 8

C0020649 GO:0016101 1 8

C0020649 GO:0006563 1 8

C0020649 GO:0006595 1 8

C0020649 GO:0030530 1 8

C0020649 GO:0043601 1 8

C0020649 GO:0008630 1 8

C0020649 GO:0050820 1 8

C0020649 GO:0016279 1 8

C0020649 GO:0003727 1 8

C0020649 GO:0006536 1 8

C0020649 GO:0042744 1 8

C0020649 GO:0016653 1 8

C0020649 GO:0006004 1 8

C0020649 GO:0009084 2 8

C0020649 GO:0019104 1 8

C0020649 GO:0032404 1 8

C0020649 GO:0006000 1 8

C0020649 GO:0030032 1 8

C0020649 GO:0004177 1 8

C0020649 GO:0019047 1 8

C0020649 GO:0016127 1 8

C0020649 GO:0046364 1 8

C0020649 GO:0008408 1 8

C0020649 GO:0034508 2 8

C0020649 GO:0048754 1 8

C0020649 GO:0030983 1 8

C0020649 GO:0008213 1 8

C0020649 GO:0046040 1 8

C0020649 GO:0009109 1 8

C0020649 GO:0051148 2 8

C0020649 GO:0050716 1 8

C0020649 GO:0001523 1 8

C0020649 GO:0045187 1 8

C0020649 GO:0016846 1 8

C0020649 GO:0016840 1 8

C0020649 GO:0032981 1 8

C0020649 GO:0033993 1 8

C0020649 GO:0043981 1 8

C0020649 GO:0003730 1 8

C0020649 GO:0042162 1 8

C0020649 GO:0016246 1 8

C0020649 GO:0070918 1 8

C0020649 GO:0016209 2 8

C0020649 GO:0043984 1 8

C0020649 GO:0045930 1 8

C0020649 GO:0006707 1 8

C0020649 GO:0017119 1 8

C0020649 GO:0070120 1 8

C0020649 GO:0045936 1 8

C0020649 GO:0045939 1 8

C0020649 GO:0006769 1 8

C0020649 GO:0031047 1 8

C0020649 GO:0048593 1 8

C0020649 GO:0034614 1 8

C0020649 GO:0004549 1 8

C0020649 GO:0000272 1 8

C0020649 GO:0002711 1 8

C0020649 GO:0005883 1 8

C0020649 GO:0022410 1 8

C0020649 GO:0043983 1 8

C0020649 GO:0051646 1 8

C0020649 GO:0005852 2 8

C0020649 GO:0002444 1 8

C0020649 GO:0002446 1 8

C0020649 GO:0016725 1 8

C0020649 GO:0009746 1 8

C0020649 GO:0000178 1 8

C0020649 GO:0000070 1 8

C0020649 GO:0051881 1 8

C0020649 GO:0030532 1 8

C0020649 GO:0035194 1 8

C0020649 GO:0010563 1 8

C0020649 GO:0035196 1 8

C0020649 GO:0042551 1 8

C0020649 GO:0032200 1 8

C0020649 GO:0009749 1 8

C0020649 GO:0001938 1 8

C0020649 GO:0022600 1 8

C0020649 GO:0019902 1 8

C0020649 GO:0000790 2 8

C0020649 GO:0001937 1 8

C0020649 GO:0000387 1 8

C0020649 GO:0018024 1 8

C0020649 GO:0015030 1 8

C0020649 GO:0001933 1 8

C0020649 GO:0001936 1 8

C0020649 GO:0043548 1 8

C0020649 GO:0002833 2 8

C0020649 GO:0019362 1 8

C0030554 GO:0005786 1 8

C0030554 GO:0051043 1 8

C0030554 GO:0007598 1 8

C0030554 GO:0022624 1 8

C0030554 GO:0048066 1 8

C0030554 GO:0002706 2 8

C0030554 GO:0002703 1 8

C0030554 GO:0016868 1 8

C0030554 GO:0016866 1 8

C0030554 GO:0002709 1 8

C0030554 GO:0005838 1 8

C0030554 GO:0003158 1 8

C0030554 GO:0046504 1 8

C0030554 GO:0030858 1 8

C0030554 GO:0016455 1 8

C0030554 GO:0051294 1 8

C0030554 GO:0002824 1 8

C0030554 GO:0002822 2 8

C0030554 GO:0031109 1 8

C0030554 GO:0002821 2 8

C0030554 GO:0035270 1 8

C0030554 GO:0003841 2 8

C0030554 GO:0035272 1 8

C0030554 GO:0006278 1 8

C0030554 GO:0006271 2 8

C0030554 GO:0080010 1 8

C0030554 GO:0016814 1 8

C0030554 GO:0010887 2 8

C0030554 GO:0010885 1 8

C0030554 GO:0031958 1 8

C0030554 GO:0001953 2 8

C0030554 GO:0002700 1 8

C0030554 GO:0030174 1 8

C0030554 GO:0030073 1 8

C0030554 GO:0016607 2 8

C0030554 GO:0015992 1 8

C0030554 GO:0015491 1 8

C0030554 GO:0042551 1 8

C0030554 GO:0006885 1 8

C0030554 GO:0042255 1 8

C0030554 GO:0042558 1 8

C0030554 GO:0042559 1 8

C0030554 GO:0070742 1 8

C0030554 GO:0002687 1 8

C0030554 GO:0002685 1 8

C0030554 GO:0009168 3 8

C0030554 GO:0043021 1 8

C0030554 GO:0043022 1 8

C0030554 GO:0009309 1 8

C0030554 GO:0009304 1 8

C0030554 GO:0016653 1 8

C0030554 GO:0048709 1 8

C0030554 GO:0009303 1 8

C0030554 GO:0033764 1 8

C0030554 GO:0009260 2 8

C0030554 GO:0006303 1 8

C0030554 GO:0044042 1 8

C0030554 GO:0030672 1 8

C0030554 GO:0031080 2 8

C0030554 GO:0004536 1 8

C0030554 GO:0006000 1 8

C0030554 GO:0070567 1 8

C0030554 GO:0030880 1 8

C0030554 GO:0008143 2 8

C0030554 GO:0033209 1 8

C0030554 GO:0019047 1 8

C0030554 GO:0004693 1 8

C0030554 GO:0043484 1 8

C0030554 GO:0045178 1 8

C0030554 GO:0030705 1 8

C0030554 GO:0002228 2 8

C0030554 GO:0045621 1 8

C0030554 GO:0045622 1 8

C0030554 GO:0045749 1 8

C0030554 GO:0005720 1 8

C0030554 GO:0008329 1 8

C0030554 GO:0005913 1 8

C0030554 GO:0001656 1 8

C0030554 GO:0006978 1 8

C0030554 GO:0042542 1 8

C0030554 GO:0003899 1 8

C0030554 GO:0017046 1 8

C0030554 GO:0045923 1 8

C0030554 GO:0018105 1 8

C0030554 GO:0017048 1 8

C0030554 GO:0060021 1 8

C0030554 GO:0008213 1 8

C0030554 GO:0007270 1 8

C0030554 GO:0008603 1 8

C0030554 GO:0006090 1 8

C0030554 GO:0003730 1 8

C0030554 GO:0019213 1 8

C0030554 GO:0045088 1 8

C0030554 GO:0070120 1 8

C0030554 GO:0019218 1 8

C0030554 GO:0048593 1 8

C0030554 GO:0000445 3 8

C0030554 GO:0030530 1 8

C0030554 GO:0040017 1 8

C0030554 GO:0030532 2 8

C0030554 GO:0032981 1 8

C0030554 GO:0006413 1 8

C0030554 GO:0007612 1 8

C0030554 GO:0000080 1 8

C0030554 GO:0009451 1 8

C0030554 GO:0043523 1 8

C0030554 GO:0043525 2 8

C0030554 GO:0007006 1 8

C0030554 GO:0007004 1 8

C0030554 GO:0002263 1 8

C0030554 GO:0010257 1 8

C0030554 GO:0001909 1 8

C0030554 GO:0032135 2 8

C0030554 GO:0033032 2 8

C0030554 GO:0004385 1 8

C0030554 GO:0016893 1 8

C0030554 GO:0000790 1 8

C0030554 GO:0016891 2 8

C0030554 GO:0000796 1 8

C0030554 GO:0009071 1 8

C0030554 GO:0018209 1 8

C0030554 GO:0000018 1 8

C0030554 GO:0016445 1 8

C0030554 GO:0006297 1 8

C0030554 GO:0002839 1 8

C0030554 GO:0002834 1 8

C0030554 GO:0002837 1 8

C0030554 GO:0002836 1 8

C0030554 GO:0034062 1 8

C0030554 GO:0034061 1 8

C0030554 GO:0050820 1 8

C0030554 GO:0001763 1 8

C0030554 GO:0001764 1 8

C0030554 GO:0006563 2 8

C0030554 GO:0016801 1 8

C0030554 GO:0016363 1 8

C0030554 GO:0032813 1 8

C0030554 GO:0000339 1 8

C0030554 GO:0017015 2 8

C0030554 GO:0019319 1 8

C0030554 GO:0050795 1 8

C0030554 GO:0008494 1 8

C0030554 GO:0030069 1 8

C0030554 GO:0046660 1 8

C0030554 GO:0016944 1 8

C0030554 GO:0043666 1 8

C0030554 GO:0006895 1 8

C0030554 GO:0010718 1 8

C0030554 GO:0010717 1 8

C0030554 GO:0042267 2 8

C0030554 GO:0006739 1 8

C0030554 GO:0048306 1 8

C0030554 GO:0042401 1 8

C0030554 GO:0031306 1 8

C0030554 GO:0007041 1 8

C0030554 GO:0016706 1 8

C0030554 GO:0004526 1 8

C0030554 GO:0004521 1 8

C0030554 GO:0015166 1 8

C0030554 GO:0015165 1 8

C0030554 GO:0048568 1 8

C0030554 GO:0021954 1 8

C0030554 GO:0021953 1 8

C0030554 GO:0032404 2 8

C0030554 GO:0008139 1 8

C0030554 GO:0042162 1 8

C0030554 GO:0031397 1 8

C0030554 GO:0014065 2 8

C0030554 GO:0043473 1 8

C0030554 GO:0002460 1 8

C0030554 GO:0009084 1 8

C0030554 GO:0016018 2 8

C0030554 GO:0004177 2 8

C0030554 GO:0033135 1 8

C0030554 GO:0030983 2 8

C0030554 GO:0046040 3 8

C0030554 GO:0016840 1 8

C0030554 GO:0005814 1 8

C0030554 GO:0045930 1 8

C0030554 GO:0045939 1 8

C0030554 GO:0010812 2 8

C0030554 GO:0050690 1 8

C0030554 GO:0016903 1 8

C0030554 GO:0055067 1 8

C0030554 GO:0016909 1 8

C0030554 GO:0003709 1 8

C0030554 GO:0051881 1 8

C0030554 GO:0070717 1 8

C0030554 GO:0032200 1 8

C0030554 GO:0032202 1 8

C0030554 GO:0002833 1 8

C0030554 GO:0006479 1 8

C0030554 GO:0000245 1 8

C0030554 GO:0009218 1 8

C0030554 GO:0031970 1 8

C0030554 GO:0048469 1 8

C0030554 GO:0060491 1 8

C0030554 GO:0006073 1 8

C0030554 GO:0051287 1 8

C0030554 GO:0045446 1 8

C0030554 GO:0008634 1 8

C0030554 GO:0005092 1 8

C0030554 GO:0006607 1 8

C0030554 GO:0031274 2 8

C0030554 GO:0043535 1 8

C0030554 GO:0019902 1 8

C0030554 GO:0007172 1 8

C0030554 GO:0031272 2 8

C0030554 GO:0007076 1 8

C0030554 GO:0045793 1 8

C0030554 GO:0005666 1 8

C0030554 GO:0016471 1 8

C0030554 GO:0006672 1 8

C0030554 GO:0042398 1 8

C0030554 GO:0034379 1 8

C0030554 GO:0016885 1 8

C0030554 GO:0021700 1 8

C0030554 GO:0016289 1 8

C0030554 GO:0005852 2 8

C0030554 GO:0090100 1 8

C0030554 GO:0005851 1 8

C0030554 GO:0043548 1 8

C0030554 GO:0009112 1 8

C0030554 GO:0002200 1 8

C0030554 GO:0016578 1 8

C0030554 GO:0006818 1 8

C0030554 GO:0055029 1 8

C0030554 GO:0006189 3 8

C0030554 GO:0006188 3 8

C0030554 GO:0001672 1 8

C0030554 GO:0001570 1 8

C0030554 GO:0000738 1 8

C0030554 GO:0050810 1 8

C0030554 GO:0033176 1 8

C0030554 GO:0045580 1 8

C0030554 GO:0045582 2 8

C0030554 GO:0046677 1 8

C0030554 GO:0034399 1 8

C0030554 GO:0007250 1 8

C0030554 GO:0051087 1 8

C0030554 GO:0070761 1 8

C0030554 GO:0046364 2 8

C0030554 GO:0017156 1 8

C0030554 GO:0031333 1 8

C0030554 GO:0000217 1 8

C0030554 GO:0015884 1 8

C0030554 GO:0000428 1 8

C0030554 GO:0001952 2 8

C0030554 GO:0032479 2 8

C0030554 GO:0010770 1 8

C0030554 GO:0002039 1 8

C0030554 GO:0048754 1 8

C0030554 GO:0070822 1 8

C0030554 GO:0015074 1 8

C0030554 GO:0045766 1 8

C0030554 GO:0016229 1 8

C0030554 GO:0030330 1 8

C0030554 GO:0006911 1 8

C0030554 GO:0000779 1 8

C0030554 GO:0009116 2 8

C0030554 GO:0002718 1 8

C0030554 GO:0000178 1 8

C0030554 GO:0001516 1 8

C0030554 GO:0046457 1 8

C0030554 GO:0046456 1 8

C0030554 GO:0046519 1 8

C0030554 GO:0016504 1 8

C0030554 GO:0016505 1 8

C0030554 GO:0046638 2 8

C0030554 GO:0046637 1 8

C0030554 GO:0050684 1 8

C0030554 GO:0046635 1 8

C0030554 GO:0046634 1 8

C0030554 GO:0046631 1 8

C0030554 GO:0008276 1 8

C0030554 GO:0050920 1 8

C0030554 GO:0050921 1 8

C0030554 GO:0051893 1 8

C0030554 GO:0031984 1 8

C0030554 GO:0006983 1 8

C0030554 GO:0043043 1 8

C0030554 GO:0006266 1 8

C0030554 GO:0010894 1 8

C0030554 GO:0006268 1 8

C0030554 GO:0006760 1 8

C0030554 GO:0030165 1 8

C0030554 GO:0000502 1 8

C0030554 GO:0030515 1 8

C0030554 GO:0030511 1 8

C0030554 GO:0005663 1 8

C0030554 GO:0005662 1 8

C0030554 GO:0048500 1 8

C0030554 GO:0019438 1 8

C0030554 GO:0030194 1 8

C0030554 GO:0032648 1 8

C0030554 GO:0043425 1 8

C0030554 GO:0007064 1 8

C0030554 GO:0031369 1 8

C0030554 GO:0004549 1 8

C0030554 GO:0000381 2 8

C0030554 GO:0035085 1 8

C0030554 GO:0010810 2 8

C0030554 GO:0019239 3 8

C0030554 GO:0002366 1 8

C0030554 GO:0002711 1 8

C0030554 GO:0046545 1 8

C0030554 GO:0009156 3 8

C0030554 GO:0006164 1 8

C0030554 GO:0009152 1 8

C0030554 GO:0030898 1 8

C0030554 GO:0048256 1 8

C0030554 GO:0014003 1 8

C0030554 GO:0005844 1 8

C0030554 GO:0045165 1 8

C0030554 GO:0000132 1 8

C0030554 GO:0004683 1 8

C0030554 GO:0031647 1 8

C0030554 GO:0045739 1 8

C0030554 GO:0015665 1 8

C0030554 GO:0030307 1 8

C0030554 GO:0002819 1 8

C0030554 GO:0030261 1 8

C0030554 GO:0048024 1 8

C0030554 GO:0043087 1 8

C0030554 GO:0009127 3 8

C0030554 GO:0006221 1 8

C0030554 GO:0002444 2 8

C0030554 GO:0042921 1 8

C0030554 GO:0046784 3 8

C0030554 GO:0046782 1 8

C0030554 GO:0044452 1 8

C0030554 GO:0008385 2 8

C0030554 GO:0022612 1 8

C0030554 GO:0022616 2 8

C0030554 GO:0051646 1 8

C0030554 GO:0070688 1 8

C0030554 GO:0015298 1 8

C0030554 GO:0010833 1 8

C0030554 GO:0051092 1 8

C0030554 GO:0008630 1 8

C0030554 GO:0007567 1 8

C0030554 GO:0070776 1 8

C0030554 GO:0070775 1 8

C0030554 GO:0003727 1 8

C0030554 GO:0006536 1 8

C0030554 GO:0048009 1 8

C0030554 GO:0043370 1 8

C0030554 GO:0019200 1 8

C0030554 GO:0033108 1 8

C0030554 GO:0000347 3 8

C0030554 GO:0000346 3 8

C0030554 GO:0021510 1 8

C0030554 GO:0008526 1 8

C0030554 GO:0070410 1 8

C0030554 GO:0000387 1 8

C0030554 GO:0016049 1 8

C0030554 GO:0042772 1 8

C0030554 GO:0002446 2 8

C0030554 GO:0042771 1 8

C0030554 GO:0042471 1 8

C0030554 GO:0016580 1 8

C0030554 GO:0016581 1 8

C0030554 GO:0001938 1 8

C0030554 GO:0033344 1 8

C0030554 GO:0002250 1 8

C0030554 GO:0001937 1 8

C0030554 GO:0001936 1 8

C0031350 GO:0005786 1 4

C0031350 GO:0006775 1 4

C0031350 GO:0000245 1 4

C0031350 GO:0022624 1 4

C0031350 GO:0046638 1 4

C0031350 GO:0046637 1 4

C0031350 GO:0050684 1 4

C0031350 GO:0046635 1 4

C0031350 GO:0046634 1 4

C0031350 GO:0007612 1 4

C0031350 GO:0021510 1 4

C0031350 GO:0002706 1 4

C0031350 GO:0043523 1 4

C0031350 GO:0002703 1 4

C0031350 GO:0043525 1 4

C0031350 GO:0001508 1 4

C0031350 GO:0002709 1 4

C0031350 GO:0007004 1 4

C0031350 GO:0005838 1 4

C0031350 GO:0002263 1 4

C0031350 GO:0043043 1 4

C0031350 GO:0010257 1 4

C0031350 GO:0030165 1 4

C0031350 GO:0002685 1 4

C0031350 GO:0001909 1 4

C0031350 GO:0006073 1 4

C0031350 GO:0006942 1 4

C0031350 GO:0046504 1 4

C0031350 GO:0032135 2 4

C0031350 GO:0046875 1 4

C0031350 GO:0033032 1 4

C0031350 GO:0034382 1 4

C0031350 GO:0015718 1 4

C0031350 GO:0006760 1 4

C0031350 GO:0005092 1 4

C0031350 GO:0051294 2 4

C0031350 GO:0002824 1 4

C0031350 GO:0004385 1 4

C0031350 GO:0006607 1 4

C0031350 GO:0031109 1 4

C0031350 GO:0002821 1 4

C0031350 GO:0035270 1 4

C0031350 GO:0003841 1 4

C0031350 GO:0016891 1 4

C0031350 GO:0000796 1 4

C0031350 GO:0007172 1 4

C0031350 GO:0007076 1 4

C0031350 GO:0000502 1 4

C0031350 GO:0016581 1 4

C0031350 GO:0009071 1 4

C0031350 GO:0030511 1 4

C0031350 GO:0045580 1 4

C0031350 GO:0045793 1 4

C0031350 GO:0006278 1 4

C0031350 GO:0018209 1 4

C0031350 GO:0051354 1 4

C0031350 GO:0006271 1 4

C0031350 GO:0000018 1 4

C0031350 GO:0010887 1 4

C0031350 GO:0005663 1 4

C0031350 GO:0031306 1 4

C0031350 GO:0001953 1 4

C0031350 GO:0006164 1 4

C0031350 GO:0003995 1 4

C0031350 GO:0050920 1 4

C0031350 GO:0016445 1 4

C0031350 GO:0006672 1 4

C0031350 GO:0050921 1 4

C0031350 GO:0006297 1 4

C0031350 GO:0002839 1 4

C0031350 GO:0032412 1 4

C0031350 GO:0031647 1 4

C0031350 GO:0002834 1 4

C0031350 GO:0002837 1 4

C0031350 GO:0002836 1 4

C0031350 GO:0034062 1 4

C0031350 GO:0034061 1 4

C0031350 GO:0015858 1 4

C0031350 GO:0004693 1 4

C0031350 GO:0009303 1 4

C0031350 GO:0001763 1 4

C0031350 GO:0016885 1 4

C0031350 GO:0031369 1 4

C0031350 GO:0016607 1 4

C0031350 GO:0016289 1 4

C0031350 GO:0000381 2 4

C0031350 GO:0015491 1 4

C0031350 GO:0005851 1 4

C0031350 GO:0042255 1 4

C0031350 GO:0002687 1 4

C0031350 GO:0000779 1 4

C0031350 GO:0002366 1 4

C0031350 GO:0009168 2 4

C0031350 GO:0043021 1 4

C0031350 GO:0002200 1 4

C0031350 GO:0043022 1 4

C0031350 GO:0051879 1 4

C0031350 GO:0032813 1 4

C0031350 GO:0005337 1 4

C0031350 GO:0055029 1 4

C0031350 GO:0009156 2 4

C0031350 GO:0048500 1 4

C0031350 GO:0017015 1 4

C0031350 GO:0019319 1 4

C0031350 GO:0050795 1 4

C0031350 GO:0009152 1 4

C0031350 GO:0009260 1 4

C0031350 GO:0006189 2 4

C0031350 GO:0006188 2 4

C0031350 GO:0003899 1 4

C0031350 GO:0051324 1 4

C0031350 GO:0030069 1 4

C0031350 GO:0001672 1 4

C0031350 GO:0016018 1 4

C0031350 GO:0005720 1 4

C0031350 GO:0005844 1 4

C0031350 GO:0000132 2 4

C0031350 GO:0031080 2 4

C0031350 GO:0032369 1 4

C0031350 GO:0004536 1 4

C0031350 GO:0022898 1 4

C0031350 GO:0042267 2 4

C0031350 GO:0015074 1 4

C0031350 GO:0005003 1 4

C0031350 GO:0005005 1 4

C0031350 GO:0043370 1 4

C0031350 GO:0046519 1 4

C0031350 GO:0005246 1 4

C0031350 GO:0045739 1 4

C0031350 GO:0030194 1 4

C0031350 GO:0043484 1 4

C0031350 GO:0030880 1 4

C0031350 GO:0045923 1 4

C0031350 GO:0008143 1 4

C0031350 GO:0030307 1 4

C0031350 GO:0044042 1 4

C0031350 GO:0030261 1 4

C0031350 GO:0045582 1 4

C0031350 GO:0018105 1 4

C0031350 GO:0046677 1 4

C0031350 GO:0042921 1 4

C0031350 GO:0016909 1 4

C0031350 GO:0048024 1 4

C0031350 GO:0031958 1 4

C0031350 GO:0033280 1 4

C0031350 GO:0009127 2 4

C0031350 GO:0050431 1 4

C0031350 GO:0006221 1 4

C0031350 GO:0001952 1 4

C0031350 GO:0016706 1 4

C0031350 GO:0002444 1 4

C0031350 GO:0004526 1 4

C0031350 GO:0032370 2 4

C0031350 GO:0032373 1 4

C0031350 GO:0046784 1 4

C0031350 GO:0002228 2 4

C0031350 GO:0002446 1 4

C0031350 GO:0048568 1 4

C0031350 GO:0045621 1 4

C0031350 GO:0021954 1 4

C0031350 GO:0045622 1 4

C0031350 GO:0022616 1 4

C0031350 GO:0021953 1 4

C0031350 GO:0045749 1 4

C0031350 GO:0033108 1 4

C0031350 GO:0001937 1 4

C0031350 GO:0043535 1 4

C0031350 GO:0046456 1 4

C0031350 GO:0051893 1 4

C0031350 GO:0008139 1 4

C0031350 GO:0044452 1 4

C0031350 GO:0000217 1 4

C0031350 GO:0015298 1 4

C0031350 GO:0006298 1 4

C0031350 GO:0002637 1 4

C0031350 GO:0010833 1 4

C0031350 GO:0001656 1 4

C0031350 GO:0031397 1 4

C0031350 GO:0006563 1 4

C0031350 GO:0060021 1 4

C0031350 GO:0006978 1 4

C0031350 GO:0030530 1 4

C0031350 GO:0043473 1 4

C0031350 GO:0032368 1 4

C0031350 GO:0002460 1 4

C0031350 GO:0050820 1 4

C0031350 GO:0000428 1 4

C0031350 GO:0070776 1 4

C0031350 GO:0070775 1 4

C0031350 GO:0005024 1 4

C0031350 GO:0005513 1 4

C0031350 GO:0003727 1 4

C0031350 GO:0006536 1 4

C0031350 GO:0004726 1 4

C0031350 GO:0009084 1 4

C0031350 GO:0034762 1 4

C0031350 GO:0032404 3 4

C0031350 GO:0006000 1 4

C0031350 GO:0004177 2 4

C0031350 GO:0019047 1 4

C0031350 GO:0001516 1 4

C0031350 GO:0046364 1 4

C0031350 GO:0048754 1 4

C0031350 GO:0030983 2 4

C0031350 GO:0051181 1 4

C0031350 GO:0046040 2 4

C0031350 GO:0000347 1 4

C0031350 GO:0000346 1 4

C0031350 GO:0014065 1 4

C0031350 GO:0008603 1 4

C0031350 GO:0051298 1 4

C0031350 GO:0070822 1 4

C0031350 GO:0032981 1 4

C0031350 GO:0004181 1 4

C0031350 GO:0004180 1 4

C0031350 GO:0042516 1 4

C0031350 GO:0051087 1 4

C0031350 GO:0008526 1 4

C0031350 GO:0004675 1 4

C0031350 GO:0003730 1 4

C0031350 GO:0002039 1 4

C0031350 GO:0050840 1 4

C0031350 GO:0045930 1 4

C0031350 GO:0031333 1 4

C0031350 GO:0070120 1 4

C0031350 GO:0010810 1 4

C0031350 GO:0002822 1 4

C0031350 GO:0010812 1 4

C0031350 GO:0090100 1 4

C0031350 GO:0004549 1 4

C0031350 GO:0015711 1 4

C0031350 GO:0002711 1 4

C0031350 GO:0009116 1 4

C0031350 GO:0045766 1 4

C0031350 GO:0051646 1 4

C0031350 GO:0005852 2 4

C0031350 GO:0005814 1 4

C0031350 GO:0000445 1 4

C0031350 GO:0042772 1 4

C0031350 GO:0001533 1 4

C0031350 GO:0016580 1 4

C0031350 GO:0051881 1 4

C0031350 GO:0040017 1 4

C0031350 GO:0030532 1 4

C0031350 GO:0070717 1 4

C0031350 GO:0032200 1 4

C0031350 GO:0046457 1 4

C0031350 GO:0001938 1 4

C0031350 GO:0019902 1 4

C0031350 GO:0000790 1 4

C0031350 GO:0002250 1 4

C0031350 GO:0000387 1 4

C0031350 GO:0032376 1 4

C0031350 GO:0045806 1 4

C0031350 GO:0006413 1 4

C0031350 GO:0002833 1 4

C0031350 GO:0001936 1 4

C0037199 GO:0022624 1 4

C0037199 GO:0002706 2 4

C0037199 GO:0002703 1 4

C0037199 GO:0034199 1 4

C0037199 GO:0009161 1 4

C0037199 GO:0070934 1 4

C0037199 GO:0070937 1 4

C0037199 GO:0048261 1 4

C0037199 GO:0002709 1 4

C0037199 GO:0032925 1 4

C0037199 GO:0005838 1 4

C0037199 GO:0043206 1 4

C0037199 GO:0051495 1 4

C0037199 GO:0004653 1 4

C0037199 GO:0018210 1 4

C0037199 GO:0030914 1 4

C0037199 GO:0004712 1 4

C0037199 GO:0016101 1 4

C0037199 GO:0006284 1 4

C0037199 GO:0006282 1 4

C0037199 GO:0002824 1 4

C0037199 GO:0051291 1 4

C0037199 GO:0002821 1 4

C0037199 GO:0003841 1 4

C0037199 GO:0005788 1 4

C0037199 GO:0001754 1 4

C0037199 GO:0000718 1 4

C0037199 GO:0006278 1 4

C0037199 GO:0034284 1 4

C0037199 GO:0006271 2 4

C0037199 GO:0006270 1 4

C0037199 GO:0080010 1 4

C0037199 GO:0043331 1 4

C0037199 GO:0006379 1 4

C0037199 GO:0021537 2 4

C0037199 GO:0032182 1 4

C0037199 GO:0030175 1 4

C0037199 GO:0030174 1 4

C0037199 GO:0030170 1 4

C0037199 GO:0008186 1 4

C0037199 GO:0008535 1 4

C0037199 GO:0016607 1 4

C0037199 GO:0016605 1 4

C0037199 GO:0016864 1 4

C0037199 GO:0008641 1 4

C0037199 GO:0070742 1 4

C0037199 GO:0043028 1 4

C0037199 GO:0005657 1 4

C0037199 GO:0009168 2 4

C0037199 GO:0050792 1 4

C0037199 GO:0043022 1 4

C0037199 GO:0051972 1 4

C0037199 GO:0019883 1 4

C0037199 GO:0016783 1 4

C0037199 GO:0009306 1 4

C0037199 GO:0031256 1 4

C0037199 GO:0006626 1 4

C0037199 GO:0009303 1 4

C0037199 GO:0060606 1 4

C0037199 GO:0009260 2 4

C0037199 GO:0007052 1 4

C0037199 GO:0009262 1 4

C0037199 GO:0009264 1 4

C0037199 GO:0004843 1 4

C0037199 GO:0016073 1 4

C0037199 GO:0031080 1 4

C0037199 GO:0015074 1 4

C0037199 GO:0048365 1 4

C0037199 GO:0005007 1 4

C0037199 GO:0015175 1 4

C0037199 GO:0031050 1 4

C0037199 GO:0015172 1 4

C0037199 GO:0008023 1 4

C0037199 GO:0030880 1 4

C0037199 GO:0008143 1 4

C0037199 GO:0033209 1 4

C0037199 GO:0019047 2 4

C0037199 GO:0004693 1 4

C0037199 GO:0005871 1 4

C0037199 GO:0004691 1 4

C0037199 GO:0031513 1 4

C0037199 GO:0051457 1 4

C0037199 GO:0009394 1 4

C0037199 GO:0007127 1 4

C0037199 GO:0043484 1 4

C0037199 GO:0045178 1 4

C0037199 GO:0045742 1 4

C0037199 GO:0045741 1 4

C0037199 GO:0002224 1 4

C0037199 GO:0002221 1 4

C0037199 GO:0045749 1 4

C0037199 GO:0033108 1 4

C0037199 GO:0008287 1 4

C0037199 GO:0043550 2 4

C0037199 GO:0005913 1 4

C0037199 GO:0001656 2 4

C0037199 GO:0034329 1 4

C0037199 GO:0018105 1 4

C0037199 GO:0017124 1 4

C0037199 GO:0018108 1 4

C0037199 GO:0001889 1 4

C0037199 GO:0010828 1 4

C0037199 GO:0022409 1 4

C0037199 GO:0043189 1 4

C0037199 GO:0045954 1 4

C0037199 GO:0003730 2 4

C0037199 GO:0045814 1 4

C0037199 GO:0045089 1 4

C0037199 GO:0070120 3 4

C0037199 GO:0048592 1 4

C0037199 GO:0048593 2 4

C0037199 GO:0009084 2 4

C0037199 GO:0031047 1 4

C0037199 GO:0003746 1 4

C0037199 GO:0001838 1 4

C0037199 GO:0005048 1 4

C0037199 GO:0030532 2 4

C0037199 GO:0007026 1 4

C0037199 GO:0032320 1 4

C0037199 GO:0019902 1 4

C0037199 GO:0005763 1 4

C0037199 GO:0051806 1 4

C0037199 GO:0006144 1 4

C0037199 GO:0019362 1 4

C0037199 GO:0007162 1 4

C0037199 GO:0016796 1 4

C0037199 GO:0003015 1 4

C0037199 GO:0031593 1 4

C0037199 GO:0007004 1 4

C0037199 GO:0016180 1 4

C0037199 GO:0010257 1 4

C0037199 GO:0045667 1 4

C0037199 GO:0008408 1 4

C0037199 GO:0070531 1 4

C0037199 GO:0032135 2 4

C0037199 GO:0045669 1 4

C0037199 GO:0009167 1 4

C0037199 GO:0051119 1 4

C0037199 GO:0043968 1 4

C0037199 GO:0005310 1 4

C0037199 GO:0005313 1 4

C0037199 GO:0004385 1 4

C0037199 GO:0051059 1 4

C0037199 GO:0016893 1 4

C0037199 GO:0008170 1 4

C0037199 GO:0016891 2 4

C0037199 GO:0000796 2 4

C0037199 GO:0000794 1 4

C0037199 GO:0042992 2 4

C0037199 GO:0009071 1 4

C0037199 GO:0014020 1 4

C0037199 GO:0018209 2 4

C0037199 GO:0000159 1 4

C0037199 GO:0000018 2 4

C0037199 GO:0046530 1 4

C0037199 GO:0031306 1 4

C0037199 GO:0016291 1 4

C0037199 GO:0016445 1 4

C0037199 GO:0004708 1 4

C0037199 GO:0046718 1 4

C0037199 GO:0018024 1 4

C0037199 GO:0006297 2 4

C0037199 GO:0002839 1 4

C0037199 GO:0005732 1 4

C0037199 GO:0015851 1 4

C0037199 GO:0002834 1 4

C0037199 GO:0002837 1 4

C0037199 GO:0002836 1 4

C0037199 GO:0006298 1 4

C0037199 GO:0034061 1 4

C0037199 GO:0008250 1 4

C0037199 GO:0050821 1 4

C0037199 GO:0050820 1 4

C0037199 GO:0001763 1 4

C0037199 GO:0006801 1 4

C0037199 GO:0005929 1 4

C0037199 GO:0051028 1 4

C0037199 GO:0006563 1 4

C0037199 GO:0051180 1 4

C0037199 GO:0051183 1 4

C0037199 GO:0007416 1 4

C0037199 GO:0016363 1 4

C0037199 GO:0032813 1 4

C0037199 GO:0009881 1 4

C0037199 GO:0000339 1 4

C0037199 GO:0019319 2 4

C0037199 GO:0008499 1 4

C0037199 GO:0030069 2 4

C0037199 GO:0000272 1 4

C0037199 GO:0005720 2 4

C0037199 GO:0008652 1 4

C0037199 GO:0007220 1 4

C0037199 GO:0009953 1 4

C0037199 GO:0002711 1 4

C0037199 GO:0042267 1 4

C0037199 GO:0070198 1 4

C0037199 GO:0046823 2 4

C0037199 GO:0043014 1 4

C0037199 GO:0006518 1 4

C0037199 GO:0043010 1 4

C0037199 GO:0006739 1 4

C0037199 GO:0016790 1 4

C0037199 GO:0048306 1 4

C0037199 GO:0006733 1 4

C0037199 GO:0016799 1 4

C0037199 GO:0070279 1 4

C0037199 GO:0002718 1 4

C0037199 GO:0007044 1 4

C0037199 GO:0007043 1 4

C0037199 GO:0000428 1 4

C0037199 GO:0043409 1 4

C0037199 GO:0004527 1 4

C0037199 GO:0004526 2 4

C0037199 GO:0004521 1 4

C0037199 GO:0050768 1 4

C0037199 GO:0021954 3 4

C0037199 GO:0016877 1 4

C0037199 GO:0021953 3 4

C0037199 GO:0032404 2 4

C0037199 GO:0016126 1 4

C0037199 GO:0042169 1 4

C0037199 GO:0008139 1 4

C0037199 GO:0042162 1 4

C0037199 GO:0043270 1 4

C0037199 GO:0014065 1 4

C0037199 GO:0009408 1 4

C0037199 GO:0030530 2 4

C0037199 GO:0043473 1 4

C0037199 GO:0000299 1 4

C0037199 GO:0032405 1 4

C0037199 GO:0034404 1 4

C0037199 GO:0005355 1 4

C0037199 GO:0016331 2 4

C0037199 GO:0050868 1 4

C0037199 GO:0046364 2 4

C0037199 GO:0030983 2 4

C0037199 GO:0046040 2 4

C0037199 GO:0009109 1 4

C0037199 GO:0034235 1 4

C0037199 GO:0001523 1 4

C0037199 GO:0016846 2 4

C0037199 GO:0070918 1 4

C0037199 GO:0018279 1 4

C0037199 GO:0045930 1 4

C0037199 GO:0031124 1 4

C0037199 GO:0010812 1 4

C0037199 GO:0031123 1 4

C0037199 GO:0022410 1 4

C0037199 GO:0005852 2 4

C0037199 GO:0045116 1 4

C0037199 GO:0009746 1 4

C0037199 GO:0003709 1 4

C0037199 GO:0051881 1 4

C0037199 GO:0051923 1 4

C0037199 GO:0003705 1 4

C0037199 GO:0009749 1 4

C0037199 GO:0044409 1 4

C0037199 GO:0070652 1 4

C0037199 GO:0006390 1 4

C0037199 GO:0002833 1 4

C0037199 GO:0006776 1 4

C0037199 GO:0052192 1 4

C0037199 GO:0000245 2 4

C0037199 GO:0060249 1 4

C0037199 GO:0009218 1 4

C0037199 GO:0031970 1 4

C0037199 GO:0032387 3 4

C0037199 GO:0019228 1 4

C0037199 GO:0021510 1 4

C0037199 GO:0006477 1 4

C0037199 GO:0030509 1 4

C0037199 GO:0030111 1 4

C0037199 GO:0030119 1 4

C0037199 GO:0030118 1 4

C0037199 GO:0046131 1 4

C0037199 GO:0002687 1 4

C0037199 GO:0042531 1 4

C0037199 GO:0048512 1 4

C0037199 GO:0032728 1 4

C0037199 GO:0010721 1 4

C0037199 GO:0005678 1 4

C0037199 GO:0032655 1 4

C0037199 GO:0015718 1 4

C0037199 GO:0005092 1 4

C0037199 GO:0006607 2 4

C0037199 GO:0005095 1 4

C0037199 GO:0031274 1 4

C0037199 GO:0019903 1 4

C0037199 GO:0045187 1 4

C0037199 GO:0007172 1 4

C0037199 GO:0043531 1 4

C0037199 GO:0031272 1 4

C0037199 GO:0007076 2 4

C0037199 GO:0007568 1 4

C0037199 GO:0045793 1 4

C0037199 GO:0050732 1 4

C0037199 GO:0031264 1 4

C0037199 GO:0001912 1 4

C0037199 GO:0016278 1 4

C0037199 GO:0016279 1 4

C0037199 GO:0016885 1 4

C0037199 GO:0000784 1 4

C0037199 GO:0007622 1 4

C0037199 GO:0031576 1 4

C0037199 GO:0016289 2 4

C0037199 GO:0031572 1 4

C0037199 GO:0043547 1 4

C0037199 GO:0005851 2 4

C0037199 GO:0003724 1 4

C0037199 GO:0045055 1 4

C0037199 GO:0016574 1 4

C0037199 GO:0005528 1 4

C0037199 GO:0002200 1 4

C0037199 GO:0005527 1 4

C0037199 GO:0055029 1 4

C0037199 GO:0035251 1 4

C0037199 GO:0035250 1 4

C0037199 GO:0030374 1 4

C0037199 GO:0006953 1 4

C0037199 GO:0006189 2 4

C0037199 GO:0006188 2 4

C0037199 GO:0042308 2 4

C0037199 GO:0034654 1 4

C0037199 GO:0042306 1 4

C0037199 GO:0001672 1 4

C0037199 GO:0042301 1 4

C0037199 GO:0002758 1 4

C0037199 GO:0032481 1 4

C0037199 GO:0006213 1 4

C0037199 GO:0006359 1 4

C0037199 GO:0030159 1 4

C0037199 GO:0004177 1 4

C0037199 GO:0005545 2 4

C0037199 GO:0008239 1 4

C0037199 GO:0060047 1 4

C0037199 GO:0046677 1 4

C0037199 GO:0008235 1 4

C0037199 GO:0034399 1 4

C0037199 GO:0051324 1 4

C0037199 GO:0001701 1 4

C0037199 GO:0019783 1 4

C0037199 GO:0001707 1 4

C0037199 GO:0001704 1 4

C0037199 GO:0051087 1 4

C0037199 GO:0015711 1 4

C0037199 GO:0070761 1 4

C0037199 GO:0032272 1 4

C0037199 GO:0070688 1 4

C0037199 GO:0017156 1 4

C0037199 GO:0017157 1 4

C0037199 GO:0048332 1 4

C0037199 GO:0006721 1 4

C0037199 GO:0032508 1 4

C0037199 GO:0004860 1 4

C0037199 GO:0030128 1 4

C0037199 GO:0030675 1 4

C0037199 GO:0030122 1 4

C0037199 GO:0004869 1 4

C0037199 GO:0030125 1 4

C0037199 GO:0051258 1 4

C0037199 GO:0048839 1 4

C0037199 GO:0001959 1 4

C0037199 GO:0001953 1 4

C0037199 GO:0001950 1 4

C0037199 GO:0051828 1 4

C0037199 GO:0032479 1 4

C0037199 GO:0006000 1 4

C0037199 GO:0034502 1 4

C0037199 GO:0034508 1 4

C0037199 GO:0048754 1 4

C0037199 GO:0016831 1 4

C0037199 GO:0005891 1 4

C0037199 GO:0043506 1 4

C0037199 GO:0045768 1 4

C0037199 GO:0032981 1 4

C0037199 GO:0005003 1 4

C0037199 GO:0045767 1 4

C0037199 GO:0005112 1 4

C0037199 GO:0046427 1 4

C0037199 GO:0000779 2 4

C0037199 GO:0009110 1 4

C0037199 GO:0009116 1 4

C0037199 GO:0002717 1 4

C0037199 GO:0051646 1 4

C0037199 GO:0070925 1 4

C0037199 GO:0000777 1 4

C0037199 GO:0031529 1 4

C0037199 GO:0000790 2 4

C0037199 GO:0030900 1 4

C0037199 GO:0031114 1 4

C0037199 GO:0016894 1 4

C0037199 GO:0050684 1 4

C0037199 GO:0035267 1 4

C0037199 GO:0008272 1 4

C0037199 GO:0008375 1 4

C0037199 GO:0008376 1 4

C0037199 GO:0008278 1 4

C0037199 GO:0031985 1 4

C0037199 GO:0031984 1 4

C0037199 GO:0018196 1 4

C0037199 GO:0043043 1 4

C0037199 GO:0015238 1 4

C0037199 GO:0004089 1 4

C0037199 GO:0032392 1 4

C0037199 GO:0005791 1 4

C0037199 GO:0006760 1 4

C0037199 GO:0000314 1 4

C0037199 GO:0006769 1 4

C0037199 GO:0000502 2 4

C0037199 GO:0070585 1 4

C0037199 GO:0017069 1 4

C0037199 GO:0005663 2 4

C0037199 GO:0043130 1 4

C0037199 GO:0005665 1 4

C0037199 GO:0006164 2 4

C0037199 GO:0005669 1 4

C0037199 GO:0019438 1 4

C0037199 GO:0030194 1 4

C0037199 GO:0005089 1 4

C0037199 GO:0032648 1 4

C0037199 GO:0005086 1 4

C0037199 GO:0042745 1 4

C0037199 GO:0032947 2 4

C0037199 GO:0032496 1 4

C0037199 GO:0042749 1 4

C0037199 GO:0004549 2 4

C0037199 GO:0004690 1 4

C0037199 GO:0000381 1 4

C0037199 GO:0035085 1 4

C0037199 GO:0015149 1 4

C0037199 GO:0001964 1 4

C0037199 GO:0016565 1 4

C0037199 GO:0006687 1 4

C0037199 GO:0016763 2 4

C0037199 GO:0001841 1 4

C0037199 GO:0001843 1 4

C0037199 GO:0015145 1 4

C0037199 GO:0043168 1 4

C0037199 GO:0030894 1 4

C0037199 GO:0042384 1 4

C0037199 GO:0009156 2 4

C0037199 GO:0009152 2 4

C0037199 GO:0008013 1 4

C0037199 GO:0019104 1 4

C0037199 GO:0008625 1 4

C0037199 GO:0048256 1 4

C0037199 GO:0043256 1 4

C0037199 GO:0000030 1 4

C0037199 GO:0005844 1 4

C0037199 GO:0031579 1 4

C0037199 GO:0002218 1 4

C0037199 GO:0045739 2 4

C0037199 GO:0005537 1 4

C0037199 GO:0030431 1 4

C0037199 GO:0030307 1 4

C0037199 GO:0052126 1 4

C0037199 GO:0035148 1 4

C0037199 GO:0030261 1 4

C0037199 GO:0030260 1 4

C0037199 GO:0032271 1 4

C0037199 GO:0048024 1 4

C0037199 GO:0009123 1 4

C0037199 GO:0009126 1 4

C0037199 GO:0009127 2 4

C0037199 GO:0009124 1 4

C0037199 GO:0006220 1 4

C0037199 GO:0006221 1 4

C0037199 GO:0000049 1 4

C0037199 GO:0044452 1 4

C0037199 GO:0002228 1 4

C0037199 GO:0022616 1 4

C0037199 GO:0003756 1 4

C0037199 GO:0015295 1 4

C0037199 GO:0010939 1 4

C0037199 GO:0046165 1 4

C0037199 GO:0016411 1 4

C0037199 GO:0045580 1 4

C0037199 GO:0015804 1 4

C0037199 GO:0010833 1 4

C0037199 GO:0009743 1 4

C0037199 GO:0051092 1 4

C0037199 GO:0043601 1 4

C0037199 GO:0007569 1 4

C0037199 GO:0008633 1 4

C0037199 GO:0003727 2 4

C0037199 GO:0006536 2 4

C0037199 GO:0003684 1 4

C0037199 GO:0046496 1 4

C0037199 GO:0046326 1 4

C0037199 GO:0032200 1 4

C0037199 GO:0046112 1 4

C0037199 GO:0030132 1 4

C0037199 GO:0050661 1 4

C0037199 GO:0016725 1 4

C0037199 GO:0032202 1 4

C0037199 GO:0051262 1 4

C0037199 GO:0070412 1 4

C0037199 GO:0006406 1 4

C0037199 GO:0048531 1 4

C0037199 GO:0051287 1 4

C0037199 GO:0000387 2 4

C0037199 GO:0005883 1 4

C0037199 GO:0005885 1 4

C0037199 GO:0005884 1 4

C0037199 GO:0007157 1 4

C0037199 GO:0002443 1 4

C0037199 GO:0042771 1 4

C0037199 GO:0030898 1 4

C0037199 GO:0005577 1 4

C0037199 GO:0001933 1 4

C0037199 GO:0004579 1 4

C0037199 GO:0001937 2 4

C0037199 GO:0001936 1 4

C0085593 GO:0006776 1 4

C0085593 GO:0000245 1 4

C0085593 GO:0022624 1 4

C0085593 GO:0046638 1 4

C0085593 GO:0006471 1 4

C0085593 GO:0050684 1 4

C0085593 GO:0032387 1 4

C0085593 GO:0021510 1 4

C0085593 GO:0000080 1 4

C0085593 GO:0042572 1 4

C0085593 GO:0002706 2 4

C0085593 GO:0002703 1 4

C0085593 GO:0019239 1 4

C0085593 GO:0032967 1 4

C0085593 GO:0046631 1 4

C0085593 GO:0002709 1 4

C0085593 GO:0007004 1 4

C0085593 GO:0004364 1 4

C0085593 GO:0005838 1 4

C0085593 GO:0006983 1 4

C0085593 GO:0016627 1 4

C0085593 GO:0043043 1 4

C0085593 GO:0010257 1 4

C0085593 GO:0018210 1 4

C0085593 GO:0045667 1 4

C0085593 GO:0016101 1 4

C0085593 GO:0032135 1 4

C0085593 GO:0045669 1 4

C0085593 GO:0033032 1 4

C0085593 GO:0032393 1 4

C0085593 GO:0019903 1 4

C0085593 GO:0006760 1 4

C0085593 GO:0010714 1 4

C0085593 GO:0043473 1 4

C0085593 GO:0005092 1 4

C0085593 GO:0002824 1 4

C0085593 GO:0004385 1 4

C0085593 GO:0006607 2 4

C0085593 GO:0002821 2 4

C0085593 GO:0003841 1 4

C0085593 GO:0016891 1 4

C0085593 GO:0000796 1 4

C0085593 GO:0007172 1 4

C0085593 GO:0007076 1 4

C0085593 GO:0000502 1 4

C0085593 GO:0042992 1 4

C0085593 GO:0009071 1 4

C0085593 GO:0005062 1 4

C0085593 GO:0045793 1 4

C0085593 GO:0006278 1 4

C0085593 GO:0018209 2 4

C0085593 GO:0000159 1 4

C0085593 GO:0030509 1 4

C0085593 GO:0006271 1 4

C0085593 GO:0000018 1 4

C0085593 GO:0010887 1 4

C0085593 GO:0005663 1 4

C0085593 GO:0031306 1 4

C0085593 GO:0016291 1 4

C0085593 GO:0006164 1 4

C0085593 GO:0016445 1 4

C0085593 GO:0010888 1 4

C0085593 GO:0021537 1 4

C0085593 GO:0006297 1 4

C0085593 GO:0002839 1 4

C0085593 GO:0015851 1 4

C0085593 GO:0002834 1 4

C0085593 GO:0002837 1 4

C0085593 GO:0002836 1 4

C0085593 GO:0034061 1 4

C0085593 GO:0004693 1 4

C0085593 GO:0001763 1 4

C0085593 GO:0016885 1 4

C0085593 GO:0032947 1 4

C0085593 GO:0009065 1 4

C0085593 GO:0001950 1 4

C0085593 GO:0051702 1 4

C0085593 GO:0016607 1 4

C0085593 GO:0016289 1 4

C0085593 GO:0000381 1 4

C0085593 GO:0046356 1 4

C0085593 GO:0005851 1 4

C0085593 GO:0010885 1 4

C0085593 GO:0001964 1 4

C0085593 GO:0043130 1 4

C0085593 GO:0009168 1 4

C0085593 GO:0016763 1 4

C0085593 GO:0002200 1 4

C0085593 GO:0016471 1 4

C0085593 GO:0032813 1 4

C0085593 GO:0016605 1 4

C0085593 GO:0006505 1 4

C0085593 GO:0009156 1 4

C0085593 GO:0005338 1 4

C0085593 GO:0048709 1 4

C0085593 GO:0019319 1 4

C0085593 GO:0008013 1 4

C0085593 GO:0009152 1 4

C0085593 GO:0009260 1 4

C0085593 GO:0006189 1 4

C0085593 GO:0006188 1 4

C0085593 GO:0042308 1 4

C0085593 GO:0014003 1 4

C0085593 GO:0042306 1 4

C0085593 GO:0030069 1 4

C0085593 GO:0001672 1 4

C0085593 GO:0005720 1 4

C0085593 GO:0005844 1 4

C0085593 GO:0043666 1 4

C0085593 GO:0031080 1 4

C0085593 GO:0000779 1 4

C0085593 GO:0048256 1 4

C0085593 GO:0001889 1 4

C0085593 GO:0042267 1 4

C0085593 GO:0030867 1 4

C0085593 GO:0010712 1 4

C0085593 GO:0015074 1 4

C0085593 GO:0046824 1 4

C0085593 GO:0045730 1 4

C0085593 GO:0046823 1 4

C0085593 GO:0045739 1 4

C0085593 GO:0006359 1 4

C0085593 GO:0033176 1 4

C0085593 GO:0000002 1 4

C0085593 GO:0043484 1 4

C0085593 GO:0016790 1 4

C0085593 GO:0030159 1 4

C0085593 GO:0019674 1 4

C0085593 GO:0030307 1 4

C0085593 GO:0002819 1 4

C0085593 GO:0005545 1 4

C0085593 GO:0030261 1 4

C0085593 GO:0045582 1 4

C0085593 GO:0018105 1 4

C0085593 GO:0016049 1 4

C0085593 GO:0007041 1 4

C0085593 GO:0048024 1 4

C0085593 GO:0003950 1 4

C0085593 GO:0004691 1 4

C0085593 GO:0004690 1 4

C0085593 GO:0009127 1 4

C0085593 GO:0044246 1 4

C0085593 GO:0000445 1 4

C0085593 GO:0006342 1 4

C0085593 GO:0004526 1 4

C0085593 GO:0045109 1 4

C0085593 GO:0046784 1 4

C0085593 GO:0002228 1 4

C0085593 GO:0046782 2 4

C0085593 GO:0006506 1 4

C0085593 GO:0021954 2 4

C0085593 GO:0016303 1 4

C0085593 GO:0005546 1 4

C0085593 GO:0022616 1 4

C0085593 GO:0021953 2 4

C0085593 GO:0045749 1 4

C0085593 GO:0033108 1 4

C0085593 GO:0042169 1 4

C0085593 GO:0008287 1 4

C0085593 GO:0008139 1 4

C0085593 GO:0006721 1 4

C0085593 GO:0010833 1 4

C0085593 GO:0001656 1 4

C0085593 GO:0051187 1 4

C0085593 GO:0016254 1 4

C0085593 GO:0044253 1 4

C0085593 GO:0030530 1 4

C0085593 GO:0006084 1 4

C0085593 GO:0007033 1 4

C0085593 GO:0008637 1 4

C0085593 GO:0009650 1 4

C0085593 GO:0009062 1 4

C0085593 GO:0007598 1 4

C0085593 GO:0032182 1 4

C0085593 GO:0016620 1 4

C0085593 GO:0004653 1 4

C0085593 GO:0003727 1 4

C0085593 GO:0006536 1 4

C0085593 GO:0048009 1 4

C0085593 GO:0017156 1 4

C0085593 GO:0016653 1 4

C0085593 GO:0009084 1 4

C0085593 GO:0016018 1 4

C0085593 GO:0032404 1 4

C0085593 GO:0006000 1 4

C0085593 GO:0017046 1 4

C0085593 GO:0004177 1 4

C0085593 GO:0016331 1 4

C0085593 GO:0019047 1 4

C0085593 GO:0035004 1 4

C0085593 GO:0046364 1 4

C0085593 GO:0048754 1 4

C0085593 GO:0030983 1 4

C0085593 GO:0008634 1 4

C0085593 GO:0006099 1 4

C0085593 GO:0046040 1 4

C0085593 GO:0009109 1 4

C0085593 GO:0000347 1 4

C0085593 GO:0000346 1 4

C0085593 GO:0043506 1 4

C0085593 GO:0043550 1 4

C0085593 GO:0001523 1 4

C0085593 GO:0032981 1 4

C0085593 GO:0005095 1 4

C0085593 GO:0003730 1 4

C0085593 GO:0030900 1 4

C0085593 GO:0045814 1 4

C0085593 GO:0016229 1 4

C0085593 GO:0045930 1 4

C0085593 GO:0033764 2 4

C0085593 GO:0046426 1 4

C0085593 GO:0070120 2 4

C0085593 GO:0002822 1 4

C0085593 GO:0048593 1 4

C0085593 GO:0004549 1 4

C0085593 GO:0002711 1 4

C0085593 GO:0022417 1 4

C0085593 GO:0016903 1 4

C0085593 GO:0031047 1 4

C0085593 GO:0051646 1 4

C0085593 GO:0005852 1 4

C0085593 GO:0002443 1 4

C0085593 GO:0009060 1 4

C0085593 GO:0006518 1 4

C0085593 GO:0051881 1 4

C0085593 GO:0030532 1 4

C0085593 GO:0048066 1 4

C0085593 GO:0009820 1 4

C0085593 GO:0032200 1 4

C0085593 GO:0005765 1 4

C0085593 GO:0019902 1 4

C0085593 GO:0000790 1 4

C0085593 GO:0001937 1 4

C0085593 GO:0070652 1 4

C0085593 GO:0000387 1 4

C0085593 GO:0016504 1 4

C0085593 GO:0016505 1 4

C0085593 GO:0001933 1 4

C0085593 GO:0002833 1 4

C0085649 GO:0006479 1 3

C0085649 GO:0005786 1 3

C0085649 GO:0000245 1 3

C0085649 GO:0022624 1 3

C0085649 GO:0046638 1 3

C0085649 GO:0046637 1 3

C0085649 GO:0050684 1 3

C0085649 GO:0031970 1 3

C0085649 GO:0046634 1 3

C0085649 GO:0007612 1 3

C0085649 GO:0021510 1 3

C0085649 GO:0002706 1 3

C0085649 GO:0008276 1 3

C0085649 GO:0002703 1 3

C0085649 GO:0043525 1 3

C0085649 GO:0051893 1 3

C0085649 GO:0002709 1 3

C0085649 GO:0007004 1 3

C0085649 GO:0005838 1 3

C0085649 GO:0002687 1 3

C0085649 GO:0050820 1 3

C0085649 GO:0002263 1 3

C0085649 GO:0043043 1 3

C0085649 GO:0010257 1 3

C0085649 GO:0002822 1 3

C0085649 GO:0030165 1 3

C0085649 GO:0002685 1 3

C0085649 GO:0010894 1 3

C0085649 GO:0001909 1 3

C0085649 GO:0006073 1 3

C0085649 GO:0051287 1 3

C0085649 GO:0032135 2 3

C0085649 GO:0046635 1 3

C0085649 GO:0033032 1 3

C0085649 GO:0006760 1 3

C0085649 GO:0005092 1 3

C0085649 GO:0051294 1 3

C0085649 GO:0002824 1 3

C0085649 GO:0004385 1 3

C0085649 GO:0006607 1 3

C0085649 GO:0031109 1 3

C0085649 GO:0002821 1 3

C0085649 GO:0035270 1 3

C0085649 GO:0003841 1 3

C0085649 GO:0016891 1 3

C0085649 GO:0000796 1 3

C0085649 GO:0007172 1 3

C0085649 GO:0007076 1 3

C0085649 GO:0000502 1 3

C0085649 GO:0016581 1 3

C0085649 GO:0009071 1 3

C0085649 GO:0030511 1 3

C0085649 GO:0045580 1 3

C0085649 GO:0045793 1 3

C0085649 GO:0006278 1 3

C0085649 GO:0018209 1 3

C0085649 GO:0006271 1 3

C0085649 GO:0000018 1 3

C0085649 GO:0010887 1 3

C0085649 GO:0005663 1 3

C0085649 GO:0031306 1 3

C0085649 GO:0006164 1 3

C0085649 GO:0050920 1 3

C0085649 GO:0070567 1 3

C0085649 GO:0016445 1 3

C0085649 GO:0006672 1 3

C0085649 GO:0030194 1 3

C0085649 GO:0000428 1 3

C0085649 GO:0006297 1 3

C0085649 GO:0002839 1 3

C0085649 GO:0031647 1 3

C0085649 GO:0042398 1 3

C0085649 GO:0002834 1 3

C0085649 GO:0002837 1 3

C0085649 GO:0002836 1 3

C0085649 GO:0034062 1 3

C0085649 GO:0034061 1 3

C0085649 GO:0070776 1 3

C0085649 GO:0004693 1 3

C0085649 GO:0001763 1 3

C0085649 GO:0016885 1 3

C0085649 GO:0043425 1 3

C0085649 GO:0031369 1 3

C0085649 GO:0021700 1 3

C0085649 GO:0016607 1 3

C0085649 GO:0016289 1 3

C0085649 GO:0000381 2 3

C0085649 GO:0015491 1 3

C0085649 GO:0005851 1 3

C0085649 GO:0016801 1 3

C0085649 GO:0055029 1 3

C0085649 GO:0042255 1 3

C0085649 GO:0042558 1 3

C0085649 GO:0090100 1 3

C0085649 GO:0045939 1 3

C0085649 GO:0002366 1 3

C0085649 GO:0009168 2 3

C0085649 GO:0043021 1 3

C0085649 GO:0002200 1 3

C0085649 GO:0043022 1 3

C0085649 GO:0032813 1 3

C0085649 GO:0009309 1 3

C0085649 GO:0009156 2 3

C0085649 GO:0048500 1 3

C0085649 GO:0017015 1 3

C0085649 GO:0019319 1 3

C0085649 GO:0050795 1 3

C0085649 GO:0009152 1 3

C0085649 GO:0009260 1 3

C0085649 GO:0006189 2 3

C0085649 GO:0006188 2 3

C0085649 GO:0003899 1 3

C0085649 GO:0030069 1 3

C0085649 GO:0001672 1 3

C0085649 GO:0005720 1 3

C0085649 GO:0005844 1 3

C0085649 GO:0000132 1 3

C0085649 GO:0031080 2 3

C0085649 GO:0000779 1 3

C0085649 GO:0004536 1 3

C0085649 GO:0002039 1 3

C0085649 GO:0042267 2 3

C0085649 GO:0015074 1 3

C0085649 GO:0046519 1 3

C0085649 GO:0050810 1 3

C0085649 GO:0045739 1 3

C0085649 GO:0043484 1 3

C0085649 GO:0030880 1 3

C0085649 GO:0045923 1 3

C0085649 GO:0008143 1 3

C0085649 GO:0030307 1 3

C0085649 GO:0044042 1 3

C0085649 GO:0030261 1 3

C0085649 GO:0042401 1 3

C0085649 GO:0045582 1 3

C0085649 GO:0018105 1 3

C0085649 GO:0046677 1 3

C0085649 GO:0048024 1 3

C0085649 GO:0031958 1 3

C0085649 GO:0009127 2 3

C0085649 GO:0006221 1 3

C0085649 GO:0016706 1 3

C0085649 GO:0002444 1 3

C0085649 GO:0004526 1 3

C0085649 GO:0042921 1 3

C0085649 GO:0030705 1 3

C0085649 GO:0046784 1 3

C0085649 GO:0002228 2 3

C0085649 GO:0048568 1 3

C0085649 GO:0045621 1 3

C0085649 GO:0021954 1 3

C0085649 GO:0045622 1 3

C0085649 GO:0022616 1 3

C0085649 GO:0021953 1 3

C0085649 GO:0045749 1 3

C0085649 GO:0033108 1 3

C0085649 GO:0001937 1 3

C0085649 GO:0043535 1 3

C0085649 GO:0046456 1 3

C0085649 GO:0008139 1 3

C0085649 GO:0042162 1 3

C0085649 GO:0044452 1 3

C0085649 GO:0000217 1 3

C0085649 GO:0015298 1 3

C0085649 GO:0010833 1 3

C0085649 GO:0001656 1 3

C0085649 GO:0031397 1 3

C0085649 GO:0006563 1 3

C0085649 GO:0060021 1 3

C0085649 GO:0006978 1 3

C0085649 GO:0030530 1 3

C0085649 GO:0043473 1 3

C0085649 GO:0008630 1 3

C0085649 GO:0002460 1 3

C0085649 GO:0030174 1 3

C0085649 GO:0015884 1 3

C0085649 GO:0001953 2 3

C0085649 GO:0001952 2 3

C0085649 GO:0070775 1 3

C0085649 GO:0043370 1 3

C0085649 GO:0014065 1 3

C0085649 GO:0003727 1 3

C0085649 GO:0006536 1 3

C0085649 GO:0009084 1 3

C0085649 GO:0016018 1 3

C0085649 GO:0032404 2 3

C0085649 GO:0006000 1 3

C0085649 GO:0004177 2 3

C0085649 GO:0019200 1 3

C0085649 GO:0019047 1 3

C0085649 GO:0001516 1 3

C0085649 GO:0046364 1 3

C0085649 GO:0048754 1 3

C0085649 GO:0030983 2 3

C0085649 GO:0008213 1 3

C0085649 GO:0046040 2 3

C0085649 GO:0000347 1 3

C0085649 GO:0000346 1 3

C0085649 GO:0008603 1 3

C0085649 GO:0070822 1 3

C0085649 GO:0032981 1 3

C0085649 GO:0051087 1 3

C0085649 GO:0008526 1 3

C0085649 GO:0003730 1 3

C0085649 GO:0045930 1 3

C0085649 GO:0031333 1 3

C0085649 GO:0070120 1 3

C0085649 GO:0030330 1 3

C0085649 GO:0010810 2 3

C0085649 GO:0019218 1 3

C0085649 GO:0010812 2 3

C0085649 GO:0001570 1 3

C0085649 GO:0004549 1 3

C0085649 GO:0034379 1 3

C0085649 GO:0043523 1 3

C0085649 GO:0002711 1 3

C0085649 GO:0009116 1 3

C0085649 GO:0045766 1 3

C0085649 GO:0051646 1 3

C0085649 GO:0005852 2 3

C0085649 GO:0005814 1 3

C0085649 GO:0000445 1 3

C0085649 GO:0042772 1 3

C0085649 GO:0002446 1 3

C0085649 GO:0042771 1 3

C0085649 GO:0016580 1 3

C0085649 GO:0051881 1 3

C0085649 GO:0040017 1 3

C0085649 GO:0030532 2 3

C0085649 GO:0050921 1 3

C0085649 GO:0032200 1 3

C0085649 GO:0046457 1 3

C0085649 GO:0001938 1 3

C0085649 GO:0019902 1 3

C0085649 GO:0000790 1 3

C0085649 GO:0002250 1 3

C0085649 GO:0000387 1 3

C0085649 GO:0006413 1 3

C0085649 GO:0002833 1 3

C0085649 GO:0001936 1 3

C0231218 GO:0005786 1 9

C0231218 GO:0051043 1 9

C0231218 GO:0022624 1 9

C0231218 GO:0006906 1 9

C0231218 GO:0048066 1 9

C0231218 GO:0002706 2 9

C0231218 GO:0002703 1 9

C0231218 GO:0002709 1 9

C0231218 GO:0005838 1 9

C0231218 GO:0043206 1 9

C0231218 GO:0004653 2 9

C0231218 GO:0001964 1 9

C0231218 GO:0006309 1 9

C0231218 GO:0006471 1 9

C0231218 GO:0007080 1 9

C0231218 GO:0016101 1 9

C0231218 GO:0016455 1 9

C0231218 GO:0002824 1 9

C0231218 GO:0003923 1 9

C0231218 GO:0002822 1 9

C0231218 GO:0002821 2 9

C0231218 GO:0003841 2 9

C0231218 GO:0035272 1 9

C0231218 GO:0006278 1 9

C0231218 GO:0030509 1 9

C0231218 GO:0016812 1 9

C0231218 GO:0006271 1 9

C0231218 GO:0048500 1 9

C0231218 GO:0045259 1 9

C0231218 GO:0016814 1 9

C0231218 GO:0043331 1 9

C0231218 GO:0003995 1 9

C0231218 GO:0010888 1 9

C0231218 GO:0021537 1 9

C0231218 GO:0009895 1 9

C0231218 GO:0048568 1 9

C0231218 GO:0008483 1 9

C0231218 GO:0030170 1 9

C0231218 GO:0035004 1 9

C0231218 GO:0005487 1 9

C0231218 GO:0051224 1 9

C0231218 GO:0030073 1 9

C0231218 GO:0016607 1 9

C0231218 GO:0016605 1 9

C0231218 GO:0015992 1 9

C0231218 GO:0003208 1 9

C0231218 GO:0042559 1 9

C0231218 GO:0043028 1 9

C0231218 GO:0005657 1 9

C0231218 GO:0009168 1 9

C0231218 GO:0050792 1 9

C0231218 GO:0007622 1 9

C0231218 GO:0033764 1 9

C0231218 GO:0009260 2 9

C0231218 GO:0007052 1 9

C0231218 GO:0009262 1 9

C0231218 GO:0009264 1 9

C0231218 GO:0000002 1 9

C0231218 GO:0035303 1 9

C0231218 GO:0031080 1 9

C0231218 GO:0015074 1 9

C0231218 GO:0005003 1 9

C0231218 GO:0005005 1 9

C0231218 GO:0016675 1 9

C0231218 GO:0006752 1 9

C0231218 GO:0006754 1 9

C0231218 GO:0008144 1 9

C0231218 GO:0019047 1 9

C0231218 GO:0018210 1 9

C0231218 GO:0004693 1 9

C0231218 GO:0004691 1 9

C0231218 GO:0004690 1 9

C0231218 GO:0009394 1 9

C0231218 GO:0043484 1 9

C0231218 GO:0045071 1 9

C0231218 GO:0002228 1 9

C0231218 GO:0005507 1 9

C0231218 GO:0045749 1 9

C0231218 GO:0033108 1 9

C0231218 GO:0008287 1 9

C0231218 GO:0008329 1 9

C0231218 GO:0043550 1 9

C0231218 GO:0005913 1 9

C0231218 GO:0001656 1 9

C0231218 GO:0044253 1 9

C0231218 GO:0008656 1 9

C0231218 GO:0010718 1 9

C0231218 GO:0016254 1 9

C0231218 GO:0018105 1 9

C0231218 GO:0006099 1 9

C0231218 GO:0001889 1 9

C0231218 GO:0007270 1 9

C0231218 GO:0006090 1 9

C0231218 GO:0022409 1 9

C0231218 GO:0009820 1 9

C0231218 GO:0003730 1 9

C0231218 GO:0045814 2 9

C0231218 GO:0019213 1 9

C0231218 GO:0070120 2 9

C0231218 GO:0048593 1 9

C0231218 GO:0031047 1 9

C0231218 GO:0016469 1 9

C0231218 GO:0030530 1 9

C0231218 GO:0030532 1 9

C0231218 GO:0005765 1 9

C0231218 GO:0019902 1 9

C0231218 GO:0048525 1 9

C0231218 GO:0048524 1 9

C0231218 GO:0019362 1 9

C0231218 GO:0009451 1 9

C0231218 GO:0031264 1 9

C0231218 GO:0043525 1 9

C0231218 GO:0042765 1 9

C0231218 GO:0016597 1 9

C0231218 GO:0007006 2 9

C0231218 GO:0007004 1 9

C0231218 GO:0004364 1 9

C0231218 GO:0010257 1 9

C0231218 GO:0005546 1 9

C0231218 GO:0045667 1 9

C0231218 GO:0032135 1 9

C0231218 GO:0045669 1 9

C0231218 GO:0015002 1 9

C0231218 GO:0051119 1 9

C0231218 GO:0016209 1 9

C0231218 GO:0004385 1 9

C0231218 GO:0016893 2 9

C0231218 GO:0000790 1 9

C0231218 GO:0016891 3 9

C0231218 GO:0000796 1 9

C0231218 GO:0051702 2 9

C0231218 GO:0042992 1 9

C0231218 GO:0009071 1 9

C0231218 GO:0018209 2 9

C0231218 GO:0000159 1 9

C0231218 GO:0000018 1 9

C0231218 GO:0031306 1 9

C0231218 GO:0016291 1 9

C0231218 GO:0016445 1 9

C0231218 GO:0006297 1 9

C0231218 GO:0002839 1 9

C0231218 GO:0015851 2 9

C0231218 GO:0002834 1 9

C0231218 GO:0002837 1 9

C0231218 GO:0002836 1 9

C0231218 GO:0034061 1 9

C0231218 GO:0009650 1 9

C0231218 GO:0001763 1 9

C0231218 GO:0046496 1 9

C0231218 GO:0030280 1 9

C0231218 GO:0051187 1 9

C0231218 GO:0051181 1 9

C0231218 GO:0030149 1 9

C0231218 GO:0032813 1 9

C0231218 GO:0000339 2 9

C0231218 GO:0019319 2 9

C0231218 GO:0051238 1 9

C0231218 GO:0030069 1 9

C0231218 GO:0005720 1 9

C0231218 GO:0043666 2 9

C0231218 GO:0002711 1 9

C0231218 GO:0010717 1 9

C0231218 GO:0042267 1 9

C0231218 GO:0010712 1 9

C0231218 GO:0046824 1 9

C0231218 GO:0046823 1 9

C0231218 GO:0006518 1 9

C0231218 GO:0006739 1 9

C0231218 GO:0016790 1 9

C0231218 GO:0019674 1 9

C0231218 GO:0006733 1 9

C0231218 GO:0070279 1 9

C0231218 GO:0051646 1 9

C0231218 GO:0032967 1 9

C0231218 GO:0007043 1 9

C0231218 GO:0004526 1 9

C0231218 GO:0004521 2 9

C0231218 GO:0050769 1 9

C0231218 GO:0015166 1 9

C0231218 GO:0016667 1 9

C0231218 GO:0009925 1 9

C0231218 GO:0021954 2 9

C0231218 GO:0006744 1 9

C0231218 GO:0006743 1 9

C0231218 GO:0021953 2 9

C0231218 GO:0032404 1 9

C0231218 GO:0005355 1 9

C0231218 GO:0042169 1 9

C0231218 GO:0008139 1 9

C0231218 GO:0014065 1 9

C0231218 GO:0043473 1 9

C0231218 GO:0045069 1 9

C0231218 GO:0009084 1 9

C0231218 GO:0016018 1 9

C0231218 GO:0006740 1 9

C0231218 GO:0004177 1 9

C0231218 GO:0016331 1 9

C0231218 GO:0008484 1 9

C0231218 GO:0055010 1 9

C0231218 GO:0046364 2 9

C0231218 GO:0030983 1 9

C0231218 GO:0009108 1 9

C0231218 GO:0009109 2 9

C0231218 GO:0034235 1 9

C0231218 GO:0001523 1 9

C0231218 GO:0016846 1 9

C0231218 GO:0031532 1 9

C0231218 GO:0000060 1 9

C0231218 GO:0033628 1 9

C0231218 GO:0016248 1 9

C0231218 GO:0016246 1 9

C0231218 GO:0045930 1 9

C0231218 GO:0022417 1 9

C0231218 GO:0016903 1 9

C0231218 GO:0030431 1 9

C0231218 GO:0007266 1 9

C0231218 GO:0051881 1 9

C0231218 GO:0032200 1 9

C0231218 GO:0070652 1 9

C0231218 GO:0006390 1 9

C0231218 GO:0002833 1 9

C0231218 GO:0006776 1 9

C0231218 GO:0000245 1 9

C0231218 GO:0009218 1 9

C0231218 GO:0032387 2 9

C0231218 GO:0021510 1 9

C0231218 GO:0005788 1 9

C0231218 GO:0031050 1 9

C0231218 GO:0045055 1 9

C0231218 GO:0048863 1 9

C0231218 GO:0016627 1 9

C0231218 GO:0016620 1 9

C0231218 GO:0048512 1 9

C0231218 GO:0015718 1 9

C0231218 GO:0007033 1 9

C0231218 GO:0005092 1 9

C0231218 GO:0006607 2 9

C0231218 GO:0005095 1 9

C0231218 GO:0031274 1 9

C0231218 GO:0019903 1 9

C0231218 GO:0045187 1 9

C0231218 GO:0007172 1 9

C0231218 GO:0031272 1 9

C0231218 GO:0007076 1 9

C0231218 GO:0006081 1 9

C0231218 GO:0045793 1 9

C0231218 GO:0050732 1 9

C0231218 GO:0006921 1 9

C0231218 GO:0009142 1 9

C0231218 GO:0009145 1 9

C0231218 GO:0009062 1 9

C0231218 GO:0048167 1 9

C0231218 GO:0009060 1 9

C0231218 GO:0016885 1 9

C0231218 GO:0009066 1 9

C0231218 GO:0009065 1 9

C0231218 GO:0048168 1 9

C0231218 GO:0070918 1 9

C0231218 GO:0042625 1 9

C0231218 GO:0016289 1 9

C0231218 GO:0005852 1 9

C0231218 GO:0005851 1 9

C0231218 GO:0043548 1 9

C0231218 GO:0031579 1 9

C0231218 GO:0005391 1 9

C0231218 GO:0016574 1 9

C0231218 GO:0005528 1 9

C0231218 GO:0002200 1 9

C0231218 GO:0005527 1 9

C0231218 GO:0004129 1 9

C0231218 GO:0016303 1 9

C0231218 GO:0006189 1 9

C0231218 GO:0006188 1 9

C0231218 GO:0042308 1 9

C0231218 GO:0042306 1 9

C0231218 GO:0001672 1 9

C0231218 GO:0044275 1 9

C0231218 GO:0000738 1 9

C0231218 GO:0000737 1 9

C0231218 GO:0016831 1 9

C0231218 GO:0006359 1 9

C0231218 GO:0004602 1 9

C0231218 GO:0030159 1 9

C0231218 GO:0010921 1 9

C0231218 GO:0005545 1 9

C0231218 GO:0045580 1 9

C0231218 GO:0003229 1 9

C0231218 GO:0003950 1 9

C0231218 GO:0042572 1 9

C0231218 GO:0008235 1 9

C0231218 GO:0045426 1 9

C0231218 GO:0030018 1 9

C0231218 GO:0051087 1 9

C0231218 GO:0042375 1 9

C0231218 GO:0006505 1 9

C0231218 GO:0006506 1 9

C0231218 GO:0043094 1 9

C0231218 GO:0006721 1 9

C0231218 GO:0004860 1 9

C0231218 GO:0001959 1 9

C0231218 GO:0032182 1 9

C0231218 GO:0001950 1 9

C0231218 GO:0005742 1 9

C0231218 GO:0032570 1 9

C0231218 GO:0010770 1 9

C0231218 GO:0006000 1 9

C0231218 GO:0034508 1 9

C0231218 GO:0048754 1 9

C0231218 GO:0005890 1 9

C0231218 GO:0043506 1 9

C0231218 GO:0032981 1 9

C0231218 GO:0016676 1 9

C0231218 GO:0046040 1 9

C0231218 GO:0046426 1 9

C0231218 GO:0046356 1 9

C0231218 GO:0000779 1 9

C0231218 GO:0002718 1 9

C0231218 GO:0000178 1 9

C0231218 GO:0004601 1 9

C0231218 GO:0043596 1 9

C0231218 GO:0030900 1 9

C0231218 GO:0050684 1 9

C0231218 GO:0045309 1 9

C0231218 GO:0008376 1 9

C0231218 GO:0043043 1 9

C0231218 GO:0015238 1 9

C0231218 GO:0006266 1 9

C0231218 GO:0010896 1 9

C0231218 GO:0006268 1 9

C0231218 GO:0032393 1 9

C0231218 GO:0009206 1 9

C0231218 GO:0006760 1 9

C0231218 GO:0009201 1 9

C0231218 GO:0006769 1 9

C0231218 GO:0010975 1 9

C0231218 GO:0000502 1 9

C0231218 GO:0030515 1 9

C0231218 GO:0045109 1 9

C0231218 GO:0005062 1 9

C0231218 GO:0005663 1 9

C0231218 GO:0043130 1 9

C0231218 GO:0006164 1 9

C0231218 GO:0005086 1 9

C0231218 GO:0042745 1 9

C0231218 GO:0032947 1 9

C0231218 GO:0007064 1 9

C0231218 GO:0042749 1 9

C0231218 GO:0004549 1 9

C0231218 GO:0000381 1 9

C0231218 GO:0015149 1 9

C0231218 GO:0019239 1 9

C0231218 GO:0016763 1 9

C0231218 GO:0016684 1 9

C0231218 GO:0015145 1 9

C0231218 GO:0016769 1 9

C0231218 GO:0009156 1 9

C0231218 GO:0005338 1 9

C0231218 GO:0009152 2 9

C0231218 GO:0008013 1 9

C0231218 GO:0048256 2 9

C0231218 GO:0043255 1 9

C0231218 GO:0005844 1 9

C0231218 GO:0030867 2 9

C0231218 GO:0045730 1 9

C0231218 GO:0045739 1 9

C0231218 GO:0015665 1 9

C0231218 GO:0022410 1 9

C0231218 GO:0030307 1 9

C0231218 GO:0002819 1 9

C0231218 GO:0045178 2 9

C0231218 GO:0030261 1 9

C0231218 GO:0048024 1 9

C0231218 GO:0009127 1 9

C0231218 GO:0044246 1 9

C0231218 GO:0006342 1 9

C0231218 GO:0046782 1 9

C0231218 GO:0044452 1 9

C0231218 GO:0046466 1 9

C0231218 GO:0022612 1 9

C0231218 GO:0022616 1 9

C0231218 GO:0019079 1 9

C0231218 GO:0010833 2 9

C0231218 GO:0051310 1 9

C0231218 GO:0008200 1 9

C0231218 GO:0006084 1 9

C0231218 GO:0008634 1 9

C0231218 GO:0008637 1 9

C0231218 GO:0051096 1 9

C0231218 GO:0051095 1 9

C0231218 GO:0007567 1 9

C0231218 GO:0003727 1 9

C0231218 GO:0006536 1 9

C0231218 GO:0034976 1 9

C0231218 GO:0019206 1 9

C0231218 GO:0042288 1 9

C0231218 GO:0008526 1 9

C0231218 GO:0005753 1 9

C0231218 GO:0006400 1 9

C0231218 GO:0000387 1 9

C0231218 GO:0005883 1 9

C0231218 GO:0002443 1 9

C0231218 GO:0030898 1 9

C0231218 GO:0010714 1 9

C0231218 GO:0001933 1 9

C0231218 GO:0001936 1 9

C0231218 GO:0001937 1 9

C0231218 GO:0015036 1 9

C0700590 GO:0000245 1 4

C0700590 GO:0022624 1 4

C0700590 GO:0046638 1 4

C0700590 GO:0050684 1 4

C0700590 GO:0032387 1 4

C0700590 GO:0000080 1 4

C0700590 GO:0002706 1 4

C0700590 GO:0008376 1 4

C0700590 GO:0008278 1 4

C0700590 GO:0002709 1 4

C0700590 GO:0007004 1 4

C0700590 GO:0005838 1 4

C0700590 GO:0006983 1 4

C0700590 GO:0043043 1 4

C0700590 GO:0010257 1 4

C0700590 GO:0048512 1 4

C0700590 GO:0004707 1 4

C0700590 GO:0006733 1 4

C0700590 GO:0032135 1 4

C0700590 GO:0006383 1 4

C0700590 GO:0033032 1 4

C0700590 GO:0006760 1 4

C0700590 GO:0051119 1 4

C0700590 GO:0005092 1 4

C0700590 GO:0002824 1 4

C0700590 GO:0004385 1 4

C0700590 GO:0006607 1 4

C0700590 GO:0002821 1 4

C0700590 GO:0003841 1 4

C0700590 GO:0016891 1 4

C0700590 GO:0000796 1 4

C0700590 GO:0007172 1 4

C0700590 GO:0015780 1 4

C0700590 GO:0007076 1 4

C0700590 GO:0000502 1 4

C0700590 GO:0016581 1 4

C0700590 GO:0007598 1 4

C0700590 GO:0009071 1 4

C0700590 GO:0045793 1 4

C0700590 GO:0006278 1 4

C0700590 GO:0018209 1 4

C0700590 GO:0051354 1 4

C0700590 GO:0006271 1 4

C0700590 GO:0000018 1 4

C0700590 GO:0010887 1 4

C0700590 GO:0005663 2 4

C0700590 GO:0031306 1 4

C0700590 GO:0006164 1 4

C0700590 GO:0016445 1 4

C0700590 GO:0006672 1 4

C0700590 GO:0018024 1 4

C0700590 GO:0006297 2 4

C0700590 GO:0002839 1 4

C0700590 GO:0002834 1 4

C0700590 GO:0002837 1 4

C0700590 GO:0002836 1 4

C0700590 GO:0006298 1 4

C0700590 GO:0034061 1 4

C0700590 GO:0005086 1 4

C0700590 GO:0004693 1 4

C0700590 GO:0042745 1 4

C0700590 GO:0001763 1 4

C0700590 GO:0016885 1 4

C0700590 GO:0046496 1 4

C0700590 GO:0042749 1 4

C0700590 GO:0016607 1 4

C0700590 GO:0016289 1 4

C0700590 GO:0000381 1 4

C0700590 GO:0070761 1 4

C0700590 GO:0005851 2 4

C0700590 GO:0010885 1 4

C0700590 GO:0042558 1 4

C0700590 GO:0051896 1 4

C0700590 GO:0015149 1 4

C0700590 GO:0016574 1 4

C0700590 GO:0019239 1 4

C0700590 GO:0043130 1 4

C0700590 GO:0009168 1 4

C0700590 GO:0005662 1 4

C0700590 GO:0005522 1 4

C0700590 GO:0007183 1 4

C0700590 GO:0002200 1 4

C0700590 GO:0015145 1 4

C0700590 GO:0016471 1 4

C0700590 GO:0030894 1 4

C0700590 GO:0032813 1 4

C0700590 GO:0007622 1 4

C0700590 GO:0009156 1 4

C0700590 GO:0009304 1 4

C0700590 GO:0032769 1 4

C0700590 GO:0048709 1 4

C0700590 GO:0019319 1 4

C0700590 GO:0009152 1 4

C0700590 GO:0009260 2 4

C0700590 GO:0007052 1 4

C0700590 GO:0006189 1 4

C0700590 GO:0006188 1 4

C0700590 GO:0009266 1 4

C0700590 GO:0014003 1 4

C0700590 GO:0030069 1 4

C0700590 GO:0001672 1 4

C0700590 GO:0000030 1 4

C0700590 GO:0005720 1 4

C0700590 GO:0005844 1 4

C0700590 GO:0030675 1 4

C0700590 GO:0031080 1 4

C0700590 GO:0015781 1 4

C0700590 GO:0042267 1 4

C0700590 GO:0015074 1 4

C0700590 GO:0045736 1 4

C0700590 GO:0046519 1 4

C0700590 GO:0045739 1 4

C0700590 GO:0033176 1 4

C0700590 GO:0030431 1 4

C0700590 GO:0043484 1 4

C0700590 GO:0030307 1 4

C0700590 GO:0016799 1 4

C0700590 GO:0008144 1 4

C0700590 GO:0030261 2 4

C0700590 GO:0045582 1 4

C0700590 GO:0018105 1 4

C0700590 GO:0007043 1 4

C0700590 GO:0016049 1 4

C0700590 GO:0007041 1 4

C0700590 GO:0048024 1 4

C0700590 GO:0009127 1 4

C0700590 GO:0006220 1 4

C0700590 GO:0070925 1 4

C0700590 GO:0001707 1 4

C0700590 GO:0001704 1 4

C0700590 GO:0004526 1 4

C0700590 GO:0004520 1 4

C0700590 GO:0046784 1 4

C0700590 GO:0002228 1 4

C0700590 GO:0046782 1 4

C0700590 GO:0015165 1 4

C0700590 GO:0021954 1 4

C0700590 GO:0022616 1 4

C0700590 GO:0021953 1 4

C0700590 GO:0045749 1 4

C0700590 GO:0005355 1 4

C0700590 GO:0033108 1 4

C0700590 GO:0000718 1 4

C0700590 GO:0046631 1 4

C0700590 GO:0008139 1 4

C0700590 GO:0016278 1 4

C0700590 GO:0004860 1 4

C0700590 GO:0010833 1 4

C0700590 GO:0016279 1 4

C0700590 GO:0005678 1 4

C0700590 GO:0030530 1 4

C0700590 GO:0031576 1 4

C0700590 GO:0043601 1 4

C0700590 GO:0030174 1 4

C0700590 GO:0032182 1 4

C0700590 GO:0003727 1 4

C0700590 GO:0006536 1 4

C0700590 GO:0048009 1 4

C0700590 GO:0017156 1 4

C0700590 GO:0016653 1 4

C0700590 GO:0009084 1 4

C0700590 GO:0016018 1 4

C0700590 GO:0032404 2 4

C0700590 GO:0006000 1 4

C0700590 GO:0017046 1 4

C0700590 GO:0004177 1 4

C0700590 GO:0019047 1 4

C0700590 GO:0042116 1 4

C0700590 GO:0046364 1 4

C0700590 GO:0019104 1 4

C0700590 GO:0034508 1 4

C0700590 GO:0048754 1 4

C0700590 GO:0030983 1 4

C0700590 GO:0046040 1 4

C0700590 GO:0009109 1 4

C0700590 GO:0000347 1 4

C0700590 GO:0000346 1 4

C0700590 GO:0045187 1 4

C0700590 GO:0016846 1 4

C0700590 GO:0048332 1 4

C0700590 GO:0032981 1 4

C0700590 GO:0033993 1 4

C0700590 GO:0000060 1 4

C0700590 GO:0016646 1 4

C0700590 GO:0016645 1 4

C0700590 GO:0043596 1 4

C0700590 GO:0003730 1 4

C0700590 GO:0000314 1 4

C0700590 GO:0016229 1 4

C0700590 GO:0045930 1 4

C0700590 GO:0033764 1 4

C0700590 GO:0045089 1 4

C0700590 GO:0070120 1 4

C0700590 GO:0006769 1 4

C0700590 GO:0005763 1 4

C0700590 GO:0004549 1 4

C0700590 GO:0002711 1 4

C0700590 GO:0005883 1 4

C0700590 GO:0022410 1 4

C0700590 GO:0048524 1 4

C0700590 GO:0051646 1 4

C0700590 GO:0005852 1 4

C0700590 GO:0000445 1 4

C0700590 GO:0051881 1 4

C0700590 GO:0030532 1 4

C0700590 GO:0048066 1 4

C0700590 GO:0042551 1 4

C0700590 GO:0032200 1 4

C0700590 GO:0019902 1 4

C0700590 GO:0000790 1 4

C0700590 GO:0000387 1 4

C0700590 GO:0016504 1 4

C0700590 GO:0016505 1 4

C0700590 GO:0002833 1 4

C0700590 GO:0019362 1 4

C0016382 GO:0006776 1 6

C0016382 GO:0000339 1 6

C0016382 GO:0005782 1 6

C0016382 GO:0016363 1 6

C0016382 GO:0008374 1 6

C0016382 GO:0034446 1 6

C0016382 GO:0000060 1 6

C0016382 GO:0006474 1 6

C0016382 GO:0008585 1 6

C0016382 GO:0006476 1 6

C0016382 GO:0031050 1 6

C0016382 GO:0002705 1 6

C0016382 GO:0008276 1 6

C0016382 GO:0031057 1 6

C0016382 GO:0006825 1 6

C0016382 GO:0001912 1 6

C0016382 GO:0030118 1 6

C0016382 GO:0051897 1 6

C0016382 GO:0008206 1 6

C0016382 GO:0002708 1 6

C0016382 GO:0006306 1 6

C0016382 GO:0006305 1 6

C0016382 GO:0006303 2 6

C0016382 GO:0022616 1 6

C0016382 GO:0043044 1 6

C0016382 GO:0004402 1 6

C0016382 GO:0006266 1 6

C0016382 GO:0007080 1 6

C0016382 GO:0004707 1 6

C0016382 GO:0031907 1 6

C0016382 GO:0046504 1 6

C0016382 GO:0005678 2 6

C0016382 GO:0045598 1 6

C0016382 GO:0032722 1 6

C0016382 GO:0033032 1 6

C0016382 GO:0005796 1 6

C0016382 GO:0042440 1 6

C0016382 GO:0001707 1 6

C0016382 GO:0043967 1 6

C0016382 GO:0005310 1 6

C0016382 GO:0000314 1 6

C0016382 GO:0001841 1 6

C0016382 GO:0005095 1 6

C0016382 GO:0046966 1 6

C0016382 GO:0042752 1 6

C0016382 GO:0016891 1 6

C0016382 GO:0000794 1 6

C0016382 GO:0043601 1 6

C0016382 GO:0008144 1 6

C0016382 GO:0014020 1 6

C0016382 GO:0000718 1 6

C0016382 GO:0006278 1 6

C0016382 GO:0016812 1 6

C0016382 GO:0060395 1 6

C0016382 GO:0006270 1 6

C0016382 GO:0030111 1 6

C0016382 GO:0051354 1 6

C0016382 GO:0005663 1 6

C0016382 GO:0043130 1 6

C0016382 GO:0004550 2 6

C0016382 GO:0006672 2 6

C0016382 GO:0016667 1 6

C0016382 GO:0046718 1 6

C0016382 GO:0018024 2 6

C0016382 GO:0006297 2 6

C0016382 GO:0018149 1 6

C0016382 GO:0006298 1 6

C0016382 GO:0016278 2 6

C0016382 GO:0016279 2 6

C0016382 GO:0010907 1 6

C0016382 GO:0032813 1 6

C0016382 GO:0016885 1 6

C0016382 GO:0032947 1 6

C0016382 GO:0009065 1 6

C0016382 GO:0009064 1 6

C0016382 GO:0007064 1 6

C0016382 GO:0031369 1 6

C0016382 GO:0030178 1 6

C0016382 GO:0070918 1 6

C0016382 GO:0031576 1 6

C0016382 GO:0032091 1 6

C0016382 GO:0006563 1 6

C0016382 GO:0045116 1 6

C0016382 GO:0045649 1 6

C0016382 GO:0042551 2 6

C0016382 GO:0016045 1 6

C0016382 GO:0043087 1 6

C0016382 GO:0016281 1 6

C0016382 GO:0042098 1 6

C0016382 GO:0008406 1 6

C0016382 GO:0070461 1 6

C0016382 GO:0042558 1 6

C0016382 GO:0008278 1 6

C0016382 GO:0070742 1 6

C0016382 GO:0019239 1 6

C0016382 GO:0005662 1 6

C0016382 GO:0016763 1 6

C0016382 GO:0045913 1 6

C0016382 GO:0007183 1 6

C0016382 GO:0001843 1 6

C0016382 GO:0007004 1 6

C0016382 GO:0042826 1 6

C0016382 GO:0030897 1 6

C0016382 GO:0030894 1 6

C0016382 GO:0048593 1 6

C0016382 GO:0046545 1 6

C0016382 GO:0016780 1 6

C0016382 GO:0009156 1 6

C0016382 GO:0009304 3 6

C0016382 GO:0032769 1 6

C0016382 GO:0043410 1 6

C0016382 GO:0006953 1 6

C0016382 GO:0031163 1 6

C0016382 GO:0007127 1 6

C0016382 GO:0043393 1 6

C0016382 GO:0008494 1 6

C0016382 GO:0009266 1 6

C0016382 GO:0032890 1 6

C0016382 GO:0034655 1 6

C0016382 GO:0034656 1 6

C0016382 GO:0034341 1 6

C0016382 GO:0046660 1 6

C0016382 GO:0000578 1 6

C0016382 GO:0001541 1 6

C0016382 GO:0043498 1 6

C0016382 GO:0060606 1 6

C0016382 GO:0006891 1 6

C0016382 GO:0030675 1 6

C0016382 GO:0004683 2 6

C0016382 GO:0015781 1 6

C0016382 GO:0015780 1 6

C0016382 GO:0048469 1 6

C0016382 GO:0032088 1 6

C0016382 GO:0045736 1 6

C0016382 GO:0043014 1 6

C0016382 GO:0015662 1 6

C0016382 GO:0005522 1 6

C0016382 GO:0042834 1 6

C0016382 GO:0016796 1 6

C0016382 GO:0033209 1 6

C0016382 GO:0016799 1 6

C0016382 GO:0006929 1 6

C0016382 GO:0035148 1 6

C0016382 GO:0030261 1 6

C0016382 GO:0030260 1 6

C0016382 GO:0009410 1 6

C0016382 GO:0007040 1 6

C0016382 GO:0052126 1 6

C0016382 GO:0004697 1 6

C0016382 GO:0000030 1 6

C0016382 GO:0000723 1 6

C0016382 GO:0000127 1 6

C0016382 GO:0001701 1 6

C0016382 GO:0019783 1 6

C0016382 GO:0000726 1 6

C0016382 GO:0001704 1 6

C0016382 GO:0002444 1 6

C0016382 GO:0004527 1 6

C0016382 GO:0004520 1 6

C0016382 GO:0042116 1 6

C0016382 GO:0032479 1 6

C0016382 GO:0046782 1 6

C0016382 GO:0015165 1 6

C0016382 GO:0042791 1 6

C0016382 GO:0008385 1 6

C0016382 GO:0016877 1 6

C0016382 GO:0034399 1 6

C0016382 GO:0006740 1 6

C0016382 GO:0016944 1 6

C0016382 GO:0042168 1 6

C0016382 GO:0006749 1 6

C0016382 GO:0048332 1 6

C0016382 GO:0051017 1 6

C0016382 GO:0070304 1 6

C0016382 GO:0005913 1 6

C0016382 GO:0010833 1 6

C0016382 GO:0010676 1 6

C0016382 GO:0051310 1 6

C0016382 GO:0009408 1 6

C0016382 GO:0007131 1 6

C0016382 GO:0030125 1 6

C0016382 GO:0031625 2 6

C0016382 GO:0070761 1 6

C0016382 GO:0051096 1 6

C0016382 GO:0001959 1 6

C0016382 GO:0030174 1 6

C0016382 GO:0006383 1 6

C0016382 GO:0032182 1 6

C0016382 GO:0043596 1 6

C0016382 GO:0005742 1 6

C0016382 GO:0003727 1 6

C0016382 GO:0051828 1 6

C0016382 GO:0030099 1 6

C0016382 GO:0009084 1 6

C0016382 GO:0042797 1 6

C0016382 GO:0016254 1 6

C0016382 GO:0016255 1 6

C0016382 GO:0006220 1 6

C0016382 GO:0008517 1 6

C0016382 GO:0031365 1 6

C0016382 GO:0006144 1 6

C0016382 GO:0051289 1 6

C0016382 GO:0033135 1 6

C0016382 GO:0009260 2 6

C0016382 GO:0016234 1 6

C0016382 GO:0052192 1 6

C0016382 GO:0009109 1 6

C0016382 GO:0016607 1 6

C0016382 GO:0050661 1 6

C0016382 GO:0016840 1 6

C0016382 GO:0033993 1 6

C0016382 GO:0006527 1 6

C0016382 GO:0016646 1 6

C0016382 GO:0016645 1 6

C0016382 GO:0018409 1 6

C0016382 GO:0033344 1 6

C0016382 GO:0042787 1 6

C0016382 GO:0004468 1 6

C0016382 GO:0032404 1 6

C0016382 GO:0070328 1 6

C0016382 GO:0035085 1 6

C0016382 GO:0045088 1 6

C0016382 GO:0005763 1 6

C0016382 GO:0045089 1 6

C0016382 GO:0019104 1 6

C0016382 GO:0016226 1 6

C0016382 GO:0016878 2 6

C0016382 GO:0002711 1 6

C0016382 GO:0022417 1 6

C0016382 GO:0035035 1 6

C0016382 GO:0015030 1 6

C0016382 GO:0030104 1 6

C0016382 GO:0051183 1 6

C0016382 GO:0070925 1 6

C0016382 GO:0051896 1 6

C0016382 GO:0006518 1 6

C0016382 GO:0002446 1 6

C0016382 GO:0050750 1 6

C0016382 GO:0001838 1 6

C0016382 GO:0000178 1 6

C0016382 GO:0016581 2 6

C0016382 GO:0016893 1 6

C0016382 GO:0009913 1 6

C0016382 GO:0070717 1 6

C0016382 GO:0005851 1 6

C0016382 GO:0046330 1 6

C0016382 GO:0001837 1 6

C0016382 GO:0005577 1 6

C0016382 GO:0044409 1 6

C0016382 GO:0046519 2 6

C0016382 GO:0048524 2 6

C0016382 GO:0051806 1 6

C0016382 GO:0043548 1 6

C0020040 GO:0007598 1 6

C0020040 GO:0002705 1 6

C0020040 GO:0034199 1 6

C0020040 GO:0008186 1 6

C0020040 GO:0070937 1 6

C0020040 GO:0016864 2 6

C0020040 GO:0016862 2 6

C0020040 GO:0002708 1 6

C0020040 GO:0006306 1 6

C0020040 GO:0006305 1 6

C0020040 GO:0006303 1 6

C0020040 GO:0006301 1 6

C0020040 GO:0043206 1 6

C0020040 GO:0005834 2 6

C0020040 GO:0004653 1 6

C0020040 GO:0018210 1 6

C0020040 GO:0004716 1 6

C0020040 GO:0009199 1 6

C0020040 GO:0007080 2 6

C0020040 GO:0008484 1 6

C0020040 GO:0016514 2 6

C0020040 GO:0045851 1 6

C0020040 GO:0002821 1 6

C0020040 GO:0046330 1 6

C0020040 GO:0002673 1 6

C0020040 GO:0006278 2 6

C0020040 GO:0016812 1 6

C0020040 GO:0006271 1 6

C0020040 GO:0006270 1 6

C0020040 GO:0045259 1 6

C0020040 GO:0016814 1 6

C0020040 GO:0042698 1 6

C0020040 GO:0010885 1 6

C0020040 GO:0010884 1 6

C0020040 GO:0003995 1 6

C0020040 GO:0006378 1 6

C0020040 GO:0010889 1 6

C0020040 GO:0018149 1 6

C0020040 GO:0032412 1 6

C0020040 GO:0048568 1 6

C0020040 GO:0000302 1 6

C0020040 GO:0010907 1 6

C0020040 GO:0000578 1 6

C0020040 GO:0070934 1 6

C0020040 GO:0005484 1 6

C0020040 GO:0030178 1 6

C0020040 GO:0042551 1 6

C0020040 GO:0008641 1 6

C0020040 GO:0045913 1 6

C0020040 GO:0016860 1 6

C0020040 GO:0042826 1 6

C0020040 GO:0051972 1 6

C0020040 GO:0016780 2 6

C0020040 GO:0009304 2 6

C0020040 GO:0005138 1 6

C0020040 GO:0043410 1 6

C0020040 GO:0032890 1 6

C0020040 GO:0004843 1 6

C0020040 GO:0031080 1 6

C0020040 GO:0016255 1 6

C0020040 GO:0070566 1 6

C0020040 GO:0070567 1 6

C0020040 GO:0016676 1 6

C0020040 GO:0010553 2 6

C0020040 GO:0016675 1 6

C0020040 GO:0006752 1 6

C0020040 GO:0006026 1 6

C0020040 GO:0006027 1 6

C0020040 GO:0008143 1 6

C0020040 GO:0005227 1 6

C0020040 GO:0009410 1 6

C0020040 GO:0051452 1 6

C0020040 GO:0004691 1 6

C0020040 GO:0004690 1 6

C0020040 GO:0004697 1 6

C0020040 GO:0042641 1 6

C0020040 GO:0009395 1 6

C0020040 GO:0000127 3 6

C0020040 GO:0007127 1 6

C0020040 GO:0045178 1 6

C0020040 GO:0045742 1 6

C0020040 GO:0045741 1 6

C0020040 GO:0005506 1 6

C0020040 GO:0055001 1 6

C0020040 GO:0055002 1 6

C0020040 GO:0008287 1 6

C0020040 GO:0015923 1 6

C0020040 GO:0042288 1 6

C0020040 GO:0050879 1 6

C0020040 GO:0051017 1 6

C0020040 GO:0010676 1 6

C0020040 GO:0046889 1 6

C0020040 GO:0042593 1 6

C0020040 GO:0016528 1 6

C0020040 GO:0016529 1 6

C0020040 GO:0042797 3 6

C0020040 GO:0016254 1 6

C0020040 GO:0042791 3 6

C0020040 GO:0045923 1 6

C0020040 GO:0017124 1 6

C0020040 GO:0022407 1 6

C0020040 GO:0022404 1 6

C0020040 GO:0022405 1 6

C0020040 GO:0043631 1 6

C0020040 GO:0015184 1 6

C0020040 GO:0035282 1 6

C0020040 GO:0008603 2 6

C0020040 GO:0006090 1 6

C0020040 GO:0009820 1 6

C0020040 GO:0042354 1 6

C0020040 GO:0043189 1 6

C0020040 GO:0006527 1 6

C0020040 GO:0018409 1 6

C0020040 GO:0006706 1 6

C0020040 GO:0000272 1 6

C0020040 GO:0030104 1 6

C0020040 GO:0015662 1 6

C0020040 GO:0050750 2 6

C0020040 GO:0001838 1 6

C0020040 GO:0009913 2 6

C0020040 GO:0008517 1 6

C0020040 GO:0019903 1 6

C0020040 GO:0001837 1 6

C0020040 GO:0009855 1 6

C0020040 GO:0048524 1 6

C0020040 GO:0051806 1 6

C0020040 GO:0042133 2 6

C0020040 GO:0034446 1 6

C0020040 GO:0007162 1 6

C0020040 GO:0019915 1 6

C0020040 GO:0007004 2 6

C0020040 GO:0005548 1 6

C0020040 GO:0030742 1 6

C0020040 GO:0007368 1 6

C0020040 GO:0004402 2 6

C0020040 GO:0010257 2 6

C0020040 GO:0015002 1 6

C0020040 GO:0033032 1 6

C0020040 GO:0034366 1 6

C0020040 GO:0043967 2 6

C0020040 GO:0043968 1 6

C0020040 GO:0005310 2 6

C0020040 GO:0016209 1 6

C0020040 GO:0046966 2 6

C0020040 GO:0008170 1 6

C0020040 GO:0000794 1 6

C0020040 GO:0009074 1 6

C0020040 GO:0042993 1 6

C0020040 GO:0042990 1 6

C0020040 GO:0014020 1 6

C0020040 GO:0000152 1 6

C0020040 GO:0016441 1 6

C0020040 GO:0046718 1 6

C0020040 GO:0018024 3 6

C0020040 GO:0006297 1 6

C0020040 GO:0017166 1 6

C0020040 GO:0032963 1 6

C0020040 GO:0008250 1 6

C0020040 GO:0009799 1 6

C0020040 GO:0005929 1 6

C0020040 GO:0051184 1 6

C0020040 GO:0051183 2 6

C0020040 GO:0016801 1 6

C0020040 GO:0000779 1 6

C0020040 GO:0004004 1 6

C0020040 GO:0032768 1 6

C0020040 GO:0019319 1 6

C0020040 GO:0031163 2 6

C0020040 GO:0008494 1 6

C0020040 GO:0016226 2 6

C0020040 GO:0046660 1 6

C0020040 GO:0000726 2 6

C0020040 GO:0006891 1 6

C0020040 GO:0006890 1 6

C0020040 GO:0009952 1 6

C0020040 GO:0042542 1 6

C0020040 GO:0022898 1 6

C0020040 GO:0032088 1 6

C0020040 GO:0043154 1 6

C0020040 GO:0043014 1 6

C0020040 GO:0006518 1 6

C0020040 GO:0042834 1 6

C0020040 GO:0019897 1 6

C0020040 GO:0009311 1 6

C0020040 GO:0042401 1 6

C0020040 GO:0004859 2 6

C0020040 GO:0007040 1 6

C0020040 GO:0022602 1 6

C0020040 GO:0001942 1 6

C0020040 GO:0031099 1 6

C0020040 GO:0050769 1 6

C0020040 GO:0032409 1 6

C0020040 GO:0006749 1 6

C0020040 GO:0000777 1 6

C0020040 GO:0006740 3 6

C0020040 GO:0016126 1 6

C0020040 GO:0005217 1 6

C0020040 GO:0070304 1 6

C0020040 GO:0006929 1 6

C0020040 GO:0009408 1 6

C0020040 GO:0007131 1 6

C0020040 GO:0006595 1 6

C0020040 GO:0031625 1 6

C0020040 GO:0008483 1 6

C0020040 GO:0035194 1 6

C0020040 GO:0009084 1 6

C0020040 GO:0032404 1 6

C0020040 GO:0009083 1 6

C0020040 GO:0004177 1 6

C0020040 GO:0050868 1 6

C0020040 GO:0046365 1 6

C0020040 GO:0046364 1 6

C0020040 GO:0016234 1 6

C0020040 GO:0009109 1 6

C0020040 GO:0004675 1 6

C0020040 GO:0042787 1 6

C0020040 GO:0018279 1 6

C0020040 GO:0045939 1 6

C0020040 GO:0050690 1 6

C0020040 GO:0022417 1 6

C0020040 GO:0016903 1 6

C0020040 GO:0030433 1 6

C0020040 GO:0007266 1 6

C0020040 GO:0070717 2 6

C0020040 GO:0002793 1 6

C0020040 GO:0052192 1 6

C0020040 GO:0044409 1 6

C0020040 GO:0006776 1 6

C0020040 GO:0032925 1 6

C0020040 GO:0009218 1 6

C0020040 GO:0006474 1 6

C0020040 GO:0005788 1 6

C0020040 GO:0006476 1 6

C0020040 GO:0031050 2 6

C0020040 GO:0030111 2 6

C0020040 GO:0015645 1 6

C0020040 GO:0031057 1 6

C0020040 GO:0030118 1 6

C0020040 GO:0046131 1 6

C0020040 GO:0016628 1 6

C0020040 GO:0016627 1 6

C0020040 GO:0005770 2 6

C0020040 GO:0007216 1 6

C0020040 GO:0010720 1 6

C0020040 GO:0005678 1 6

C0020040 GO:0032722 2 6

C0020040 GO:0000099 1 6

C0020040 GO:0005092 2 6

C0020040 GO:0000096 1 6

C0020040 GO:0005095 1 6

C0020040 GO:0007176 1 6

C0020040 GO:0042752 1 6

C0020040 GO:0043534 1 6

C0020040 GO:0003002 1 6

C0020040 GO:0003009 1 6

C0020040 GO:0006081 1 6

C0020040 GO:0051262 1 6

C0020040 GO:0060395 1 6

C0020040 GO:0004550 2 6

C0020040 GO:0006672 1 6

C0020040 GO:0046006 1 6

C0020040 GO:0042398 1 6

C0020040 GO:0009141 1 6

C0020040 GO:0009144 1 6

C0020040 GO:0016174 1 6

C0020040 GO:0016278 3 6

C0020040 GO:0016279 3 6

C0020040 GO:0016885 1 6

C0020040 GO:0009065 2 6

C0020040 GO:0009064 1 6

C0020040 GO:0070918 2 6

C0020040 GO:0042625 1 6

C0020040 GO:0005852 2 6

C0020040 GO:0045116 1 6

C0020040 GO:0016281 1 6

C0020040 GO:0031672 1 6

C0020040 GO:0031579 1 6

C0020040 GO:0016571 1 6

C0020040 GO:0005522 1 6

C0020040 GO:0016579 1 6

C0020040 GO:0016578 1 6

C0020040 GO:0004129 1 6

C0020040 GO:0004128 1 6

C0020040 GO:0030099 1 6

C0020040 GO:0030374 1 6

C0020040 GO:0001776 1 6

C0020040 GO:0042307 1 6

C0020040 GO:0034655 1 6

C0020040 GO:0034656 1 6

C0020040 GO:0042303 1 6

C0020040 GO:0044275 1 6

C0020040 GO:0060606 1 6

C0020040 GO:0000738 1 6

C0020040 GO:0006953 1 6

C0020040 GO:0006213 1 6

C0020040 GO:0004601 1 6

C0020040 GO:0004602 2 6

C0020040 GO:0032770 1 6

C0020040 GO:0018196 1 6

C0020040 GO:0015280 1 6

C0020040 GO:0051828 1 6

C0020040 GO:0017022 1 6

C0020040 GO:0010927 1 6

C0020040 GO:0042306 1 6

C0020040 GO:0050881 1 6

C0020040 GO:0001701 2 6

C0020040 GO:0019783 1 6

C0020040 GO:0007259 1 6

C0020040 GO:0003756 2 6

C0020040 GO:0030656 1 6

C0020040 GO:0030125 1 6

C0020040 GO:0050770 1 6

C0020040 GO:0001959 3 6

C0020040 GO:0050772 2 6

C0020040 GO:0015884 1 6

C0020040 GO:0044272 1 6

C0020040 GO:0005024 1 6

C0020040 GO:0005742 1 6

C0020040 GO:0070461 2 6

C0020040 GO:0016653 1 6

C0020040 GO:0006004 1 6

C0020040 GO:0006007 1 6

C0020040 GO:0042054 1 6

C0020040 GO:0042119 1 6

C0020040 GO:0005161 1 6

C0020040 GO:0030833 1 6

C0020040 GO:0032981 2 6

C0020040 GO:0031638 1 6

C0020040 GO:0045766 1 6

C0020040 GO:0004467 1 6

C0020040 GO:0046034 1 6

C0020040 GO:0006695 1 6

C0020040 GO:0004468 2 6

C0020040 GO:0005112 1 6

C0020040 GO:0030239 1 6

C0020040 GO:0007585 1 6

C0020040 GO:0034341 2 6

C0020040 GO:0016878 3 6

C0020040 GO:0002711 1 6

C0020040 GO:0042026 1 6

C0020040 GO:0016877 2 6

C0020040 GO:0031529 1 6

C0020040 GO:0019842 1 6

C0020040 GO:0046519 2 6

C0020040 GO:0016504 1 6

C0020040 GO:0007090 1 6

C0020040 GO:0008589 1 6

C0020040 GO:0051289 1 6

C0020040 GO:0051287 1 6

C0020040 GO:0050681 1 6

C0020040 GO:0008585 1 6

C0020040 GO:0035267 1 6

C0020040 GO:0006825 2 6

C0020040 GO:0008374 1 6

C0020040 GO:0034765 1 6

C0020040 GO:0051897 2 6

C0020040 GO:0034762 1 6

C0020040 GO:0043044 1 6

C0020040 GO:0070646 1 6

C0020040 GO:0010894 1 6

C0020040 GO:0032393 1 6

C0020040 GO:0009205 1 6

C0020040 GO:0005791 1 6

C0020040 GO:0030165 1 6

C0020040 GO:0010975 1 6

C0020040 GO:0005066 2 6

C0020040 GO:0006044 1 6

C0020040 GO:0019439 1 6

C0020040 GO:0070325 2 6

C0020040 GO:0031365 1 6

C0020040 GO:0043425 1 6

C0020040 GO:0007064 1 6

C0020040 GO:0031369 2 6

C0020040 GO:0055102 1 6

C0020040 GO:0045649 1 6

C0020040 GO:0016045 1 6

C0020040 GO:0008406 1 6

C0020040 GO:0016763 1 6

C0020040 GO:0001841 1 6

C0020040 GO:0001843 1 6

C0020040 GO:0016684 1 6

C0020040 GO:0016769 1 6

C0020040 GO:0030897 1 6

C0020040 GO:0046545 1 6

C0020040 GO:0043393 1 6

C0020040 GO:0048256 1 6

C0020040 GO:0042633 1 6

C0020040 GO:0043498 2 6

C0020040 GO:0004683 1 6

C0020040 GO:0030867 2 6

C0020040 GO:0033261 1 6

C0020040 GO:0016469 1 6

C0020040 GO:0004033 1 6

C0020040 GO:0035148 1 6

C0020040 GO:0051004 1 6

C0020040 GO:0030260 1 6

C0020040 GO:0051000 1 6

C0020040 GO:0052126 1 6

C0020040 GO:0009798 1 6

C0020040 GO:0000723 1 6

C0020040 GO:0000049 1 6

C0020040 GO:0070603 2 6

C0020040 GO:0001541 1 6

C0020040 GO:0046782 2 6

C0020040 GO:0033273 1 6

C0020040 GO:0019079 1 6

C0020040 GO:0046165 1 6

C0020040 GO:0035035 1 6

C0020040 GO:0055088 1 6

C0020040 GO:0010833 1 6

C0020040 GO:0051310 1 6

C0020040 GO:0008206 1 6

C0020040 GO:0051096 1 6

C0020040 GO:0043603 1 6

C0020040 GO:0042364 1 6

C0020040 GO:0003727 2 6

C0020040 GO:0003724 1 6

C0020040 GO:0017148 1 6

C0020040 GO:0034976 1 6

C0020040 GO:0033108 2 6

C0020040 GO:0030134 1 6

C0020040 GO:0050661 1 6

C0020040 GO:0016725 1 6

C0020040 GO:0033500 1 6

C0020040 GO:0016645 1 6

C0020040 GO:0005753 1 6

C0020040 GO:0010741 1 6

C0020040 GO:0070328 1 6

C0020040 GO:0042104 1 6

C0020040 GO:0008210 1 6

C0020040 GO:0007159 2 6

C0020040 GO:0007157 1 6

C0020040 GO:0007018 1 6

C0020040 GO:0005577 1 6

C0020040 GO:0033344 1 6

C0020040 GO:0004576 1 6

C0020040 GO:0004579 1 6

C0151825 GO:0006776 1 2

C0151825 GO:0034446 1 2

C0151825 GO:0006474 1 2

C0151825 GO:0006476 1 2

C0151825 GO:0002705 1 2

C0151825 GO:0051897 1 2

C0151825 GO:0002708 1 2

C0151825 GO:0006303 1 2

C0151825 GO:0006783 1 2

C0151825 GO:0007213 1 2

C0151825 GO:0051289 1 2

C0151825 GO:0007080 1 2

C0151825 GO:0043967 1 2

C0151825 GO:0016209 1 2

C0151825 GO:0016893 1 2

C0151825 GO:0042752 1 2

C0151825 GO:0016891 1 2

C0151825 GO:0000794 1 2

C0151825 GO:0006779 1 2

C0151825 GO:0006278 1 2

C0151825 GO:0006270 1 2

C0151825 GO:0004550 1 2

C0151825 GO:0006297 1 2

C0151825 GO:0018149 1 2

C0151825 GO:0034062 1 2

C0151825 GO:0006595 1 2

C0151825 GO:0010907 1 2

C0151825 GO:0042744 1 2

C0151825 GO:0009065 1 2

C0151825 GO:0009064 1 2

C0151825 GO:0008408 1 2

C0151825 GO:0005852 1 2

C0151825 GO:0045116 1 2

C0151825 GO:0016045 1 2

C0151825 GO:0016281 1 2

C0151825 GO:0010149 1 2

C0151825 GO:0033014 1 2

C0151825 GO:0006684 1 2

C0151825 GO:0016763 1 2

C0151825 GO:0045913 1 2

C0151825 GO:0007004 1 2

C0151825 GO:0004128 1 2

C0151825 GO:0016653 1 2

C0151825 GO:0043410 1 2

C0151825 GO:0031163 1 2

C0151825 GO:0009262 1 2

C0151825 GO:0009264 1 2

C0151825 GO:0003899 1 2

C0151825 GO:0032890 1 2

C0151825 GO:0046148 1 2

C0151825 GO:0043498 1 2

C0151825 GO:0004683 1 2

C0151825 GO:0007127 1 2

C0151825 GO:0032088 1 2

C0151825 GO:0048365 1 2

C0151825 GO:0042834 1 2

C0151825 GO:0016799 1 2

C0151825 GO:0031307 1 2

C0151825 GO:0000723 1 2

C0151825 GO:0009394 1 2

C0151825 GO:0001701 1 2

C0151825 GO:0019783 1 2

C0151825 GO:0000726 1 2

C0151825 GO:0004521 1 2

C0151825 GO:0070301 1 2

C0151825 GO:0070304 1 2

C0151825 GO:0010833 1 2

C0151825 GO:0010676 1 2

C0151825 GO:0051310 1 2

C0151825 GO:0009408 1 2

C0151825 GO:0007131 1 2

C0151825 GO:0051096 1 2

C0151825 GO:0005742 1 2

C0151825 GO:0030099 1 2

C0151825 GO:0009084 2 2

C0151825 GO:0016254 1 2

C0151825 GO:0006220 1 2

C0151825 GO:0031365 1 2

C0151825 GO:0018409 1 2

C0151825 GO:0042787 1 2

C0151825 GO:0070328 1 2

C0151825 GO:0017119 1 2

C0151825 GO:0034614 1 2

C0151825 GO:0019104 1 2

C0151825 GO:0016226 1 2

C0151825 GO:0016878 1 2

C0151825 GO:0002711 1 2

C0151825 GO:0046330 1 2

C0151825 GO:0005577 1 2

C0151825 GO:0015030 1 2

C0030552 GO:0006776 1 3

C0030552 GO:0034446 1 3

C0030552 GO:0006474 1 3

C0030552 GO:0006476 1 3

C0030552 GO:0002706 1 3

C0030552 GO:0060491 1 3

C0030552 GO:0016868 1 3

C0030552 GO:0016866 1 3

C0030552 GO:0051897 1 3

C0030552 GO:0009168 1 3

C0030552 GO:0002708 1 3

C0030552 GO:0031984 1 3

C0030552 GO:0006303 1 3

C0030552 GO:0003158 1 3

C0030552 GO:0051289 1 3

C0030552 GO:0007080 1 3

C0030552 GO:0030858 1 3

C0030552 GO:0043967 1 3

C0030552 GO:0002822 1 3

C0030552 GO:0002821 1 3

C0030552 GO:0031274 1 3

C0030552 GO:0042752 1 3

C0030552 GO:0000794 1 3

C0030552 GO:0031272 1 3

C0030552 GO:0030515 1 3

C0030552 GO:0009451 1 3

C0030552 GO:0006278 1 3

C0030552 GO:0006271 1 3

C0030552 GO:0006270 1 3

C0030552 GO:0002705 1 3

C0030552 GO:0016814 1 3

C0030552 GO:0048256 1 3

C0030552 GO:0005662 1 3

C0030552 GO:0004550 1 3

C0030552 GO:0005666 1 3

C0030552 GO:0019438 1 3

C0030552 GO:0006297 1 3

C0030552 GO:0018149 1 3

C0030552 GO:0002700 1 3

C0030552 GO:0032648 1 3

C0030552 GO:0080010 1 3

C0030552 GO:0010907 1 3

C0030552 GO:0001764 1 3

C0030552 GO:0009065 1 3

C0030552 GO:0009064 1 3

C0030552 GO:0055067 1 3

C0030552 GO:0015992 1 3

C0030552 GO:0045116 1 3

C0030552 GO:0016045 1 3

C0030552 GO:0006885 1 3

C0030552 GO:0016281 1 3

C0030552 GO:0042559 1 3

C0030552 GO:0019239 1 3

C0030552 GO:0016763 1 3

C0030552 GO:0045913 1 3

C0030552 GO:0007004 1 3

C0030552 GO:0016578 1 3

C0030552 GO:0006818 1 3

C0030552 GO:0046545 1 3

C0030552 GO:0032479 1 3

C0030552 GO:0017015 1 3

C0030552 GO:0009303 1 3

C0030552 GO:0043410 1 3

C0030552 GO:0031163 1 3

C0030552 GO:0007127 1 3

C0030552 GO:0006189 1 3

C0030552 GO:0008494 1 3

C0030552 GO:0032890 1 3

C0030552 GO:0006911 1 3

C0030552 GO:0046660 1 3

C0030552 GO:0051092 1 3

C0030552 GO:0043498 1 3

C0030552 GO:0045165 1 3

C0030552 GO:0030672 1 3

C0030552 GO:0043666 1 3

C0030552 GO:0006895 1 3

C0030552 GO:0009112 1 3

C0030552 GO:0032088 1 3

C0030552 GO:0042834 1 3

C0030552 GO:0006188 1 3

C0030552 GO:0006739 1 3

C0030552 GO:0048306 1 3

C0030552 GO:0008143 1 3

C0030552 GO:0002819 1 3

C0030552 GO:0000738 1 3

C0030552 GO:0009127 1 3

C0030552 GO:0000723 1 3

C0030552 GO:0001701 1 3

C0030552 GO:0019783 1 3

C0030552 GO:0000726 1 3

C0030552 GO:0007250 1 3

C0030552 GO:0046784 1 3

C0030552 GO:0070761 1 3

C0030552 GO:0015165 1 3

C0030552 GO:0070688 1 3

C0030552 GO:0008385 2 3

C0030552 GO:0070304 1 3

C0030552 GO:0010833 1 3

C0030552 GO:0010676 1 3

C0030552 GO:0051310 1 3

C0030552 GO:0009408 1 3

C0030552 GO:0007131 1 3

C0030552 GO:0008634 1 3

C0030552 GO:0051096 1 3

C0030552 GO:0042542 1 3

C0030552 GO:0005742 1 3

C0030552 GO:0030099 1 3

C0030552 GO:0009084 1 3

C0030552 GO:0016254 1 3

C0030552 GO:0031365 1 3

C0030552 GO:0046364 1 3

C0030552 GO:0017048 1 3

C0030552 GO:0046040 1 3

C0030552 GO:0000347 1 3

C0030552 GO:0000346 1 3

C0030552 GO:0045446 1 3

C0030552 GO:0018409 1 3

C0030552 GO:0042787 1 3

C0030552 GO:0070410 1 3

C0030552 GO:0070328 1 3

C0030552 GO:0050690 1 3

C0030552 GO:0016226 1 3

C0030552 GO:0016878 1 3

C0030552 GO:0002711 1 3

C0030552 GO:0016903 1 3

C0030552 GO:0009116 1 3

C0030552 GO:0016909 1 3

C0030552 GO:0000445 1 3

C0030552 GO:0042471 1 3

C0030552 GO:0003709 1 3

C0030552 GO:0070717 1 3

C0030552 GO:0046330 1 3

C0030552 GO:0032202 1 3

C0030552 GO:0005577 1 3

C0030552 GO:0033344 1 3

C0021311 GO:0043954 1 8

C0021311 GO:0006901 1 8

C0021311 GO:0006900 1 8

C0021311 GO:0006903 1 8

C0021311 GO:0009167 1 8

C0021311 GO:0034199 1 8

C0021311 GO:0009161 1 8

C0021311 GO:0016864 2 8

C0021311 GO:0016862 2 8

C0021311 GO:0009168 1 8

C0021311 GO:0016860 3 8

C0021311 GO:0043206 2 8

C0021311 GO:0005834 2 8

C0021311 GO:0004653 2 8

C0021311 GO:0006309 2 8

C0021311 GO:0030914 1 8

C0021311 GO:0016514 1 8

C0021311 GO:0051294 1 8

C0021311 GO:0003923 1 8

C0021311 GO:0051293 1 8

C0021311 GO:0048066 1 8

C0021311 GO:0002673 1 8

C0021311 GO:0006278 2 8

C0021311 GO:0016812 1 8

C0021311 GO:0045259 2 8

C0021311 GO:0016814 3 8

C0021311 GO:0010884 1 8

C0021311 GO:0010883 1 8

C0021311 GO:0003995 3 8

C0021311 GO:0019320 1 8

C0021311 GO:0032412 1 8

C0021311 GO:0000302 2 8

C0021311 GO:0008483 2 8

C0021311 GO:0030170 1 8

C0021311 GO:0008484 3 8

C0021311 GO:0051224 1 8

C0021311 GO:0003205 1 8

C0021311 GO:0003206 1 8

C0021311 GO:0015992 2 8

C0021311 GO:0006885 1 8

C0021311 GO:0042255 1 8

C0021311 GO:0003208 1 8

C0021311 GO:0051650 1 8

C0021311 GO:0002687 1 8

C0021311 GO:0002685 1 8

C0021311 GO:0051653 1 8

C0021311 GO:0006626 1 8

C0021311 GO:0048709 1 8

C0021311 GO:0009303 1 8

C0021311 GO:0009267 1 8

C0021311 GO:0019395 1 8

C0021311 GO:0035303 1 8

C0021311 GO:0006691 1 8

C0021311 GO:0070567 1 8

C0021311 GO:0048365 1 8

C0021311 GO:0016676 2 8

C0021311 GO:0015179 1 8

C0021311 GO:0010553 1 8

C0021311 GO:0016675 2 8

C0021311 GO:0015175 2 8

C0021311 GO:0006026 1 8

C0021311 GO:0006027 1 8

C0021311 GO:0005343 1 8

C0021311 GO:0033613 1 8

C0021311 GO:0033209 1 8

C0021311 GO:0008144 1 8

C0021311 GO:0014003 1 8

C0021311 GO:0031228 1 8

C0021311 GO:0019047 1 8

C0021311 GO:0051453 1 8

C0021311 GO:0005871 2 8

C0021311 GO:0051457 1 8

C0021311 GO:0030261 1 8

C0021311 GO:0009395 1 8

C0021311 GO:0000127 1 8

C0021311 GO:0030705 1 8

C0021311 GO:0016417 1 8

C0021311 GO:0005506 1 8

C0021311 GO:0005507 1 8

C0021311 GO:0055001 1 8

C0021311 GO:0055002 1 8

C0021311 GO:0042552 1 8

C0021311 GO:0006879 1 8

C0021311 GO:0042288 2 8

C0021311 GO:0050879 1 8

C0021311 GO:0051015 1 8

C0021311 GO:0042113 1 8

C0021311 GO:0034637 1 8

C0021311 GO:0001654 1 8

C0021311 GO:0046889 3 8

C0021311 GO:0033293 1 8

C0021311 GO:0044253 1 8

C0021311 GO:0006099 1 8

C0021311 GO:0016528 1 8

C0021311 GO:0016529 1 8

C0021311 GO:0004190 1 8

C0021311 GO:0043983 1 8

C0021311 GO:0042797 1 8

C0021311 GO:0042791 1 8

C0021311 GO:0010714 1 8

C0021311 GO:0017048 1 8

C0021311 GO:0042593 1 8

C0021311 GO:0022404 1 8

C0021311 GO:0022405 1 8

C0021311 GO:0042594 2 8

C0021311 GO:0045444 1 8

C0021311 GO:0015184 1 8

C0021311 GO:0008603 1 8

C0021311 GO:0006096 2 8

C0021311 GO:0009820 3 8

C0021311 GO:0043189 1 8

C0021311 GO:0017080 1 8

C0021311 GO:0019212 2 8

C0021311 GO:0006706 1 8

C0021311 GO:0048592 1 8

C0021311 GO:0000272 2 8

C0021311 GO:0031109 1 8

C0021311 GO:0000445 1 8

C0021311 GO:0010984 1 8

C0021311 GO:0009913 1 8

C0021311 GO:0035195 1 8

C0021311 GO:0032981 2 8

C0021311 GO:0001837 1 8

C0021311 GO:0006144 1 8

C0021311 GO:0019363 2 8

C0021311 GO:0019362 2 8

C0021311 GO:0034440 1 8

C0021311 GO:0042133 2 8

C0021311 GO:0043449 1 8

C0021311 GO:0003015 1 8

C0021311 GO:0019915 1 8

C0021311 GO:0042765 1 8

C0021311 GO:0016597 2 8

C0021311 GO:0007004 2 8

C0021311 GO:0042446 1 8

C0021311 GO:0005540 1 8

C0021311 GO:0010257 2 8

C0021311 GO:0015002 2 8

C0021311 GO:0034366 1 8

C0021311 GO:0043967 1 8

C0021311 GO:0005310 1 8

C0021311 GO:0016209 3 8

C0021311 GO:0000796 1 8

C0021311 GO:0009074 2 8

C0021311 GO:0006941 1 8

C0021311 GO:0031668 1 8

C0021311 GO:0016645 1 8

C0021311 GO:0016444 1 8

C0021311 GO:0004033 1 8

C0021311 GO:0017166 2 8

C0021311 GO:0015851 1 8

C0021311 GO:0006298 1 8

C0021311 GO:0008250 2 8

C0021311 GO:0048009 1 8

C0021311 GO:0046496 2 8

C0021311 GO:0030280 1 8

C0021311 GO:0051181 2 8

C0021311 GO:0051180 1 8

C0021311 GO:0016801 1 8

C0021311 GO:0042098 1 8

C0021311 GO:0016363 1 8

C0021311 GO:0030148 1 8

C0021311 GO:0030149 1 8

C0021311 GO:0031941 1 8

C0021311 GO:0032768 1 8

C0021311 GO:0032769 1 8

C0021311 GO:0019319 3 8

C0021311 GO:0031163 1 8

C0021311 GO:0008499 1 8

C0021311 GO:0003231 1 8

C0021311 GO:0030069 1 8

C0021311 GO:0034341 1 8

C0021311 GO:0046660 1 8

C0021311 GO:0055088 1 8

C0021311 GO:0006890 1 8

C0021311 GO:0043666 1 8

C0021311 GO:0042542 3 8

C0021311 GO:0043982 1 8

C0021311 GO:0022898 2 8

C0021311 GO:0005640 1 8

C0021311 GO:0010712 1 8

C0021311 GO:0043984 1 8

C0021311 GO:0070198 1 8

C0021311 GO:0046824 1 8

C0021311 GO:0003746 2 8

C0021311 GO:0019897 1 8

C0021311 GO:0006739 1 8

C0021311 GO:0006635 1 8

C0021311 GO:0006733 1 8

C0021311 GO:0009311 1 8

C0021311 GO:0070279 1 8

C0021311 GO:0032967 1 8

C0021311 GO:0004859 1 8

C0021311 GO:0032963 1 8

C0021311 GO:0030665 1 8

C0021311 GO:0030660 1 8

C0021311 GO:0030663 1 8

C0021311 GO:0001942 1 8

C0021311 GO:0004526 1 8

C0021311 GO:0031099 1 8

C0021311 GO:0001945 1 8

C0021311 GO:0050768 1 8

C0021311 GO:0050769 2 8

C0021311 GO:0032409 1 8

C0021311 GO:0006740 3 8

C0021311 GO:0016126 1 8

C0021311 GO:0008034 1 8

C0021311 GO:0005217 2 8

C0021311 GO:0031625 1 8

C0021311 GO:0016018 1 8

C0021311 GO:0005104 1 8

C0021311 GO:0004177 1 8

C0021311 GO:0050868 1 8

C0021311 GO:0055010 1 8

C0021311 GO:0046365 2 8

C0021311 GO:0046364 3 8

C0021311 GO:0016234 1 8

C0021311 GO:0046040 1 8

C0021311 GO:0009109 1 8

C0021311 GO:0034235 1 8

C0021311 GO:0050996 1 8

C0021311 GO:0031532 1 8

C0021311 GO:0004180 1 8

C0021311 GO:0018279 2 8

C0021311 GO:0070301 2 8

C0021311 GO:0045939 3 8

C0021311 GO:0050690 1 8

C0021311 GO:0022417 2 8

C0021311 GO:0016903 2 8

C0021311 GO:0055067 1 8

C0021311 GO:0007266 3 8

C0021311 GO:0003709 2 8

C0021311 GO:0003706 1 8

C0021311 GO:0046697 1 8

C0021311 GO:0008290 1 8

C0021311 GO:0005788 2 8

C0021311 GO:0030119 1 8

C0021311 GO:0030118 1 8

C0021311 GO:0015718 1 8

C0021311 GO:0007219 1 8

C0021311 GO:0016628 1 8

C0021311 GO:0016627 2 8

C0021311 GO:0007216 2 8

C0021311 GO:0016620 1 8

C0021311 GO:0001893 1 8

C0021311 GO:0070001 1 8

C0021311 GO:0010720 1 8

C0021311 GO:0010721 1 8

C0021311 GO:0019359 2 8

C0021311 GO:0000099 1 8

C0021311 GO:0005092 1 8

C0021311 GO:0000090 1 8

C0021311 GO:0043536 1 8

C0021311 GO:0007076 1 8

C0021311 GO:0055117 2 8

C0021311 GO:0003009 1 8

C0021311 GO:0008630 1 8

C0021311 GO:0005527 2 8

C0021311 GO:0004550 1 8

C0021311 GO:0033559 1 8

C0021311 GO:0008091 1 8

C0021311 GO:0045494 1 8

C0021311 GO:0006921 1 8

C0021311 GO:0009142 1 8

C0021311 GO:0031593 1 8

C0021311 GO:0009145 1 8

C0021311 GO:0009062 1 8

C0021311 GO:0048167 1 8

C0021311 GO:0016885 1 8

C0021311 GO:0009066 1 8

C0021311 GO:0009065 1 8

C0021311 GO:0008375 1 8

C0021311 GO:0009068 1 8

C0021311 GO:0048168 1 8

C0021311 GO:0042625 1 8

C0021311 GO:0005852 2 8

C0021311 GO:0005851 1 8

C0021311 GO:0031345 1 8

C0021311 GO:0031672 1 8

C0021311 GO:0031579 1 8

C0021311 GO:0005391 1 8

C0021311 GO:0005528 2 8

C0021311 GO:0046520 1 8

C0021311 GO:0016471 1 8

C0021311 GO:0004129 2 8

C0021311 GO:0004128 1 8

C0021311 GO:0006818 1 8

C0021311 GO:0001776 1 8

C0021311 GO:0045334 1 8

C0021311 GO:0006189 1 8

C0021311 GO:0006188 1 8

C0021311 GO:0042303 1 8

C0021311 GO:0008308 1 8

C0021311 GO:0044275 4 8

C0021311 GO:0010155 1 8

C0021311 GO:0000737 1 8

C0021311 GO:0004601 3 8

C0021311 GO:0033176 1 8

C0021311 GO:0032770 1 8

C0021311 GO:0010921 1 8

C0021311 GO:0045055 1 8

C0021311 GO:0017022 1 8

C0021311 GO:0010927 1 8

C0021311 GO:0060047 1 8

C0021311 GO:0003229 1 8

C0021311 GO:0060048 1 8

C0021311 GO:0030018 1 8

C0021311 GO:0034399 1 8

C0021311 GO:0050881 1 8

C0021311 GO:0015278 1 8

C0021311 GO:0042274 1 8

C0021311 GO:0015370 1 8

C0021311 GO:0003756 2 8

C0021311 GO:0017156 1 8

C0021311 GO:0030669 1 8

C0021311 GO:0043094 1 8

C0021311 GO:0031330 1 8

C0021311 GO:0030128 1 8

C0021311 GO:0004864 2 8

C0021311 GO:0030122 1 8

C0021311 GO:0000738 2 8

C0021311 GO:0030121 1 8

C0021311 GO:0030126 1 8

C0021311 GO:0030656 1 8

C0021311 GO:0030125 1 8

C0021311 GO:0050770 1 8

C0021311 GO:0001959 2 8

C0021311 GO:0050772 1 8

C0021311 GO:0008535 1 8

C0021311 GO:0000956 1 8

C0021311 GO:0005744 1 8

C0021311 GO:0016653 1 8

C0021311 GO:0006007 3 8

C0021311 GO:0034502 1 8

C0021311 GO:0042116 1 8

C0021311 GO:0048205 1 8

C0021311 GO:0005890 1 8

C0021311 GO:0070566 1 8

C0021311 GO:0048200 1 8

C0021311 GO:0009201 1 8

C0021311 GO:0043467 1 8

C0021311 GO:0015074 2 8

C0021311 GO:0007029 1 8

C0021311 GO:0031638 1 8

C0021311 GO:0030641 1 8

C0021311 GO:0005007 1 8

C0021311 GO:0046356 1 8

C0021311 GO:0030239 1 8

C0021311 GO:0007584 1 8

C0021311 GO:0007585 1 8

C0021311 GO:0016226 1 8

C0021311 GO:0016878 1 8

C0021311 GO:0034220 1 8

C0021311 GO:0009110 1 8

C0021311 GO:0042026 1 8

C0021311 GO:0051646 1 8

C0021311 GO:0009119 1 8

C0021311 GO:0018342 1 8

C0021311 GO:0000070 1 8

C0021311 GO:0006754 1 8

C0021311 GO:0019842 2 8

C0021311 GO:0004602 2 8

C0021311 GO:0051287 1 8

C0021311 GO:0046631 1 8

C0021311 GO:0055072 1 8

C0021311 GO:0035267 1 8

C0021311 GO:0045309 2 8

C0021311 GO:0034765 1 8

C0021311 GO:0034762 1 8

C0021311 GO:0008373 1 8

C0021311 GO:0006984 1 8

C0021311 GO:0018196 2 8

C0021311 GO:0015238 2 8

C0021311 GO:0006266 1 8

C0021311 GO:0043325 1 8

C0021311 GO:0010894 3 8

C0021311 GO:0010896 2 8

C0021311 GO:0010898 1 8

C0021311 GO:0032393 2 8

C0021311 GO:0009206 1 8

C0021311 GO:0005793 1 8

C0021311 GO:0006769 2 8

C0021311 GO:0010975 2 8

C0021311 GO:0030515 1 8

C0021311 GO:0070585 1 8

C0021311 GO:0051354 1 8

C0021311 GO:0042177 2 8

C0021311 GO:0005665 1 8

C0021311 GO:0019439 1 8

C0021311 GO:0042744 1 8

C0021311 GO:0042743 1 8

C0021311 GO:0031369 1 8

C0021311 GO:0055102 1 8

C0021311 GO:0004549 1 8

C0021311 GO:0000381 1 8

C0021311 GO:0008235 1 8

C0021311 GO:0008406 1 8

C0021311 GO:0019239 2 8

C0021311 GO:0016684 3 8

C0021311 GO:0016769 2 8

C0021311 GO:0046545 1 8

C0021311 GO:0009152 1 8

C0021311 GO:0009150 1 8

C0021311 GO:0005669 1 8

C0021311 GO:0042633 1 8

C0021311 GO:0045165 1 8

C0021311 GO:0000132 1 8

C0021311 GO:0043981 1 8

C0021311 GO:0051322 1 8

C0021311 GO:0030867 4 8

C0021311 GO:0016469 2 8

C0021311 GO:0051004 1 8

C0021311 GO:0030262 1 8

C0021311 GO:0051000 1 8

C0021311 GO:0048029 1 8

C0021311 GO:0009126 1 8

C0021311 GO:0009127 1 8

C0021311 GO:0044246 1 8

C0021311 GO:0070603 1 8

C0021311 GO:0001541 1 8

C0021311 GO:0046784 1 8

C0021311 GO:0046466 1 8

C0021311 GO:0046467 1 8

C0021311 GO:0070761 1 8

C0021311 GO:0010939 1 8

C0021311 GO:0019079 1 8

C0021311 GO:0046165 2 8

C0021311 GO:0015804 2 8

C0021311 GO:0030004 1 8

C0021311 GO:0010832 1 8

C0021311 GO:0010833 3 8

C0021311 GO:0010830 1 8

C0021311 GO:0045454 1 8

C0021311 GO:0055085 1 8

C0021311 GO:0006084 1 8

C0021311 GO:0006081 2 8

C0021311 GO:0043603 3 8

C0021311 GO:0042364 1 8

C0021311 GO:0042813 1 8

C0021311 GO:0003727 1 8

C0021311 GO:0045947 1 8

C0021311 GO:0045861 1 8

C0021311 GO:0034976 2 8

C0021311 GO:0000184 1 8

C0021311 GO:0019206 1 8

C0021311 GO:0033108 2 8

C0021311 GO:0019104 1 8

C0021311 GO:0000347 1 8

C0021311 GO:0000346 1 8

C0021311 GO:0030131 1 8

C0021311 GO:0030132 1 8

C0021311 GO:0030137 1 8

C0021311 GO:0016722 1 8

C0021311 GO:0040001 1 8

C0021311 GO:0008329 1 8

C0021311 GO:0033500 1 8

C0021311 GO:0051262 1 8

C0021311 GO:0008526 1 8

C0021311 GO:0005753 2 8

C0021311 GO:0010466 1 8

C0021311 GO:0048194 1 8

C0021311 GO:0008210 1 8

C0021311 GO:0048199 1 8

C0021311 GO:0005881 1 8

C0021311 GO:0007159 2 8

C0021311 GO:0002440 1 8

C0021311 GO:0007157 2 8

C0021311 GO:0002443 1 8

C0021311 GO:0042771 1 8

C0021311 GO:0002562 1 8

C0021311 GO:0033344 1 8

C0021311 GO:0004576 2 8

C0021311 GO:0004579 2 8

C0013604 GO:0051043 1 5

C0013604 GO:0032388 1 5

C0013604 GO:0009218 1 5

C0013604 GO:0003730 1 5

C0013604 GO:0032387 1 5

C0013604 GO:0048066 1 5

C0013604 GO:0014065 1 5

C0013604 GO:0006739 1 5

C0013604 GO:0043525 1 5

C0013604 GO:0004601 1 5

C0013604 GO:0045309 1 5

C0013604 GO:0042765 1 5

C0013604 GO:0008376 1 5

C0013604 GO:0045055 1 5

C0013604 GO:0016597 1 5

C0013604 GO:0007006 1 5

C0013604 GO:0070742 1 5

C0013604 GO:0000002 1 5

C0013604 GO:0005655 1 5

C0013604 GO:0004653 1 5

C0013604 GO:0005528 1 5

C0013604 GO:0006309 1 5

C0013604 GO:0015238 1 5

C0013604 GO:0048512 1 5

C0013604 GO:0010896 1 5

C0013604 GO:0010921 1 5

C0013604 GO:0006268 1 5

C0013604 GO:0015002 1 5

C0013604 GO:0016455 1 5

C0013604 GO:0009206 1 5

C0013604 GO:0035272 1 5

C0013604 GO:0015718 1 5

C0013604 GO:0009201 1 5

C0013604 GO:0051119 1 5

C0013604 GO:0016209 1 5

C0013604 GO:0003923 1 5

C0013604 GO:0006769 1 5

C0013604 GO:0006607 1 5

C0013604 GO:0005095 1 5

C0013604 GO:0016893 1 5

C0013604 GO:0008170 1 5

C0013604 GO:0045187 1 5

C0013604 GO:0016896 1 5

C0013604 GO:0010975 1 5

C0013604 GO:0031272 1 5

C0013604 GO:0030515 1 5

C0013604 GO:0008144 1 5

C0013604 GO:0009066 1 5

C0013604 GO:0016684 1 5

C0013604 GO:0016812 1 5

C0013604 GO:0019206 1 5

C0013604 GO:0045259 1 5

C0013604 GO:0016814 1 5

C0013604 GO:0005527 1 5

C0013604 GO:0003995 1 5

C0013604 GO:0006378 1 5

C0013604 GO:0009895 1 5

C0013604 GO:0006921 1 5

C0013604 GO:0030681 1 5

C0013604 GO:0009145 1 5

C0013604 GO:0005086 1 5

C0013604 GO:0008483 1 5

C0013604 GO:0042745 1 5

C0013604 GO:0046496 1 5

C0013604 GO:0009065 1 5

C0013604 GO:0005929 1 5

C0013604 GO:0007064 1 5

C0013604 GO:0005487 1 5

C0013604 GO:0051224 1 5

C0013604 GO:0048168 1 5

C0013604 GO:0034708 1 5

C0013604 GO:0030073 1 5

C0013604 GO:0042625 1 5

C0013604 GO:0008408 1 5

C0013604 GO:0005852 1 5

C0013604 GO:0008235 1 5

C0013604 GO:0042255 1 5

C0013604 GO:0003208 1 5

C0013604 GO:0031579 1 5

C0013604 GO:0015149 1 5

C0013604 GO:0016574 1 5

C0013604 GO:0019239 1 5

C0013604 GO:0043021 2 5

C0013604 GO:0043022 1 5

C0013604 GO:0015145 1 5

C0013604 GO:0016769 1 5

C0013604 GO:0043027 1 5

C0013604 GO:0004129 1 5

C0013604 GO:0030149 1 5

C0013604 GO:0007622 1 5

C0013604 GO:0033077 1 5

C0013604 GO:0000339 1 5

C0013604 GO:0019319 1 5

C0013604 GO:0030898 1 5

C0013604 GO:0007052 1 5

C0013604 GO:0035303 1 5

C0013604 GO:0044275 1 5

C0013604 GO:0030677 1 5

C0013604 GO:0000738 1 5

C0013604 GO:0043666 1 5

C0013604 GO:0010718 1 5

C0013604 GO:0010717 1 5

C0013604 GO:0031647 1 5

C0013604 GO:0004532 1 5

C0013604 GO:0035097 1 5

C0013604 GO:0016676 1 5

C0013604 GO:0016675 1 5

C0013604 GO:0016469 1 5

C0013604 GO:0006754 1 5

C0013604 GO:0015665 1 5

C0013604 GO:0004602 1 5

C0013604 GO:0030431 1 5

C0013604 GO:0016796 1 5

C0013604 GO:0006733 1 5

C0013604 GO:0070279 1 5

C0013604 GO:0007043 1 5

C0013604 GO:0003229 1 5

C0013604 GO:0007266 1 5

C0013604 GO:0043206 1 5

C0013604 GO:0051457 1 5

C0013604 GO:0030018 1 5

C0013604 GO:0004527 1 5

C0013604 GO:0004521 1 5

C0013604 GO:0045178 1 5

C0013604 GO:0050769 1 5

C0013604 GO:0015166 1 5

C0013604 GO:0070688 1 5

C0013604 GO:0046466 1 5

C0013604 GO:0022612 1 5

C0013604 GO:0005507 1 5

C0013604 GO:0009142 1 5

C0013604 GO:0006740 1 5

C0013604 GO:0005355 1 5

C0013604 GO:0043094 1 5

C0013604 GO:0008329 1 5

C0013604 GO:0031330 1 5

C0013604 GO:0035035 1 5

C0013604 GO:0004860 1 5

C0013604 GO:0005913 1 5

C0013604 GO:0010833 1 5

C0013604 GO:0005593 1 5

C0013604 GO:0006081 1 5

C0013604 GO:0001959 1 5

C0013604 GO:0007567 1 5

C0013604 GO:0048167 1 5

C0013604 GO:0010770 1 5

C0013604 GO:0030170 1 5

C0013604 GO:0000737 1 5

C0013604 GO:0042288 1 5

C0013604 GO:0008484 1 5

C0013604 GO:0055010 1 5

C0013604 GO:0046364 1 5

C0013604 GO:0005391 1 5

C0013604 GO:0034508 1 5

C0013604 GO:0005890 1 5

C0013604 GO:0051181 1 5

C0013604 GO:0009109 1 5

C0013604 GO:0034235 1 5

C0013604 GO:0007270 1 5

C0013604 GO:0043631 1 5

C0013604 GO:0006090 1 5

C0013604 GO:0016846 1 5

C0013604 GO:0016840 1 5

C0013604 GO:0031532 1 5

C0013604 GO:0008526 1 5

C0013604 GO:0019079 1 5

C0013604 GO:0005753 1 5

C0013604 GO:0048488 1 5

C0013604 GO:0030280 1 5

C0013604 GO:0019213 1 5

C0013604 GO:0045089 1 5

C0013604 GO:0031124 1 5

C0013604 GO:0031123 1 5

C0013604 GO:0005883 1 5

C0013604 GO:0022410 1 5

C0013604 GO:0002718 1 5

C0013604 GO:0015992 1 5

C0013604 GO:0030867 1 5

C0013604 GO:0031274 1 5

C0013604 GO:0003841 1 5

C0013604 GO:0000175 1 5

C0013604 GO:0016891 1 5

C0013604 GO:0042749 1 5

C0013604 GO:0019362 1 5

C0012833 GO:0051043 1 12

C0012833 GO:0007598 2 12

C0012833 GO:0043954 2 12

C0012833 GO:0048066 1 12

C0012833 GO:0002706 1 12

C0012833 GO:0002703 1 12

C0012833 GO:0016868 1 12

C0012833 GO:0009247 1 12

C0012833 GO:0016866 1 12

C0012833 GO:0009168 2 12

C0012833 GO:0032925 1 12

C0012833 GO:0070513 1 12

C0012833 GO:0006303 1 12

C0012833 GO:0005834 1 12

C0012833 GO:0003158 1 12

C0012833 GO:0018212 1 12

C0012833 GO:0046027 1 12

C0012833 GO:0046504 1 12

C0012833 GO:0030858 1 12

C0012833 GO:0016455 1 12

C0012833 GO:0010390 1 12

C0012833 GO:0006282 1 12

C0012833 GO:0051294 1 12

C0012833 GO:0002824 1 12

C0012833 GO:0002822 3 12

C0012833 GO:0031109 1 12

C0012833 GO:0021510 1 12

C0012833 GO:0007281 1 12

C0012833 GO:0035270 1 12

C0012833 GO:0003841 1 12

C0012833 GO:0035272 1 12

C0012833 GO:0050771 1 12

C0012833 GO:0050770 1 12

C0012833 GO:0000718 1 12

C0012833 GO:0006271 1 12

C0012833 GO:0006270 1 12

C0012833 GO:0080010 1 12

C0012833 GO:0016814 1 12

C0012833 GO:0010887 2 12

C0012833 GO:0042698 1 12

C0012833 GO:0010885 2 12

C0012833 GO:0010883 1 12

C0012833 GO:0006379 1 12

C0012833 GO:0010889 1 12

C0012833 GO:0060260 1 12

C0012833 GO:0031958 1 12

C0012833 GO:0032412 1 12

C0012833 GO:0001953 3 12

C0012833 GO:0002700 1 12

C0012833 GO:0030174 1 12

C0012833 GO:0005487 1 12

C0012833 GO:0030073 1 12

C0012833 GO:0016607 2 12

C0012833 GO:0015992 1 12

C0012833 GO:0015491 1 12

C0012833 GO:0042551 3 12

C0012833 GO:0006885 1 12

C0012833 GO:0042255 1 12

C0012833 GO:0042558 1 12

C0012833 GO:0042559 1 12

C0012833 GO:0070742 1 12

C0012833 GO:0002687 1 12

C0012833 GO:0005657 1 12

C0012833 GO:0043021 1 12

C0012833 GO:0043022 1 12

C0012833 GO:0007622 1 12

C0012833 GO:0009304 2 12

C0012833 GO:0016653 1 12

C0012833 GO:0048709 1 12

C0012833 GO:0009303 2 12

C0012833 GO:0009260 2 12

C0012833 GO:0007052 1 12

C0012833 GO:0009267 1 12

C0012833 GO:0009266 1 12

C0012833 GO:0044042 1 12

C0012833 GO:0030675 1 12

C0012833 GO:0030672 1 12

C0012833 GO:0031080 2 12

C0012833 GO:0004536 1 12

C0012833 GO:0015781 1 12

C0012833 GO:0015780 1 12

C0012833 GO:0004532 1 12

C0012833 GO:0070567 1 12

C0012833 GO:0006029 1 12

C0012833 GO:0010553 1 12

C0012833 GO:0006752 1 12

C0012833 GO:0030880 2 12

C0012833 GO:0018107 1 12

C0012833 GO:0008143 2 12

C0012833 GO:0033209 2 12

C0012833 GO:0005227 1 12

C0012833 GO:0008144 2 12

C0012833 GO:0051457 1 12

C0012833 GO:0000127 1 12

C0012833 GO:0045178 2 12

C0012833 GO:0002228 2 12

C0012833 GO:0045621 1 12

C0012833 GO:0045620 1 12

C0012833 GO:0045622 1 12

C0012833 GO:0002221 1 12

C0012833 GO:0045749 1 12

C0012833 GO:0033108 1 12

C0012833 GO:0016328 1 12

C0012833 GO:0008329 1 12

C0012833 GO:0032886 1 12

C0012833 GO:0005913 1 12

C0012833 GO:0010675 1 12

C0012833 GO:0001656 1 12

C0012833 GO:0016101 1 12

C0012833 GO:0006978 1 12

C0012833 GO:0042542 1 12

C0012833 GO:0043130 1 12

C0012833 GO:0004190 1 12

C0012833 GO:0003899 1 12

C0012833 GO:0042797 1 12

C0012833 GO:0042791 1 12

C0012833 GO:0017046 1 12

C0012833 GO:0045923 1 12

C0012833 GO:0018105 1 12

C0012833 GO:0017124 1 12

C0012833 GO:0017048 1 12

C0012833 GO:0060021 1 12

C0012833 GO:0045884 1 12

C0012833 GO:0042594 1 12

C0012833 GO:0015184 1 12

C0012833 GO:0007270 1 12

C0012833 GO:0008601 1 12

C0012833 GO:0045446 1 12

C0012833 GO:0006090 1 12

C0012833 GO:0002218 1 12

C0012833 GO:0033993 1 12

C0012833 GO:0032210 1 12

C0012833 GO:0019213 1 12

C0012833 GO:0045089 2 12

C0012833 GO:0045088 1 12

C0012833 GO:0048593 1 12

C0012833 GO:0000445 4 12

C0012833 GO:0050750 1 12

C0012833 GO:0050999 1 12

C0012833 GO:0040017 2 12

C0012833 GO:0008517 1 12

C0012833 GO:0050994 1 12

C0012833 GO:0050996 1 12

C0012833 GO:0008360 1 12

C0012833 GO:0005763 1 12

C0012833 GO:0048524 1 12

C0012833 GO:0006413 2 12

C0012833 GO:0048520 1 12

C0012833 GO:0019362 1 12

C0012833 GO:0007612 1 12

C0012833 GO:0000080 1 12

C0012833 GO:0009451 1 12

C0012833 GO:0043523 1 12

C0012833 GO:0043525 2 12

C0012833 GO:0031345 1 12

C0012833 GO:0007006 1 12

C0012833 GO:0002263 1 12

C0012833 GO:0010257 1 12

C0012833 GO:0001909 1 12

C0012833 GO:0032135 2 12

C0012833 GO:0033032 2 12

C0012833 GO:0051119 1 12

C0012833 GO:0016893 2 12

C0012833 GO:0008170 1 12

C0012833 GO:0016891 2 12

C0012833 GO:0016896 1 12

C0012833 GO:0009074 1 12

C0012833 GO:0090079 1 12

C0012833 GO:0004707 1 12

C0012833 GO:0007043 1 12

C0012833 GO:0018024 1 12

C0012833 GO:0006297 1 12

C0012833 GO:0034062 1 12

C0012833 GO:0046716 1 12

C0012833 GO:0050820 1 12

C0012833 GO:0048009 2 12

C0012833 GO:0001764 1 12

C0012833 GO:0046496 1 12

C0012833 GO:0005929 1 12

C0012833 GO:0051028 1 12

C0012833 GO:0018108 1 12

C0012833 GO:0006563 2 12

C0012833 GO:0051183 1 12

C0012833 GO:0055067 1 12

C0012833 GO:0000779 1 12

C0012833 GO:0007183 1 12

C0012833 GO:0016363 2 12

C0012833 GO:0005851 1 12

C0012833 GO:0032813 2 12

C0012833 GO:0009880 1 12

C0012833 GO:0000339 1 12

C0012833 GO:0032769 1 12

C0012833 GO:0017015 2 12

C0012833 GO:0050795 1 12

C0012833 GO:0008494 1 12

C0012833 GO:0046660 1 12

C0012833 GO:0016944 1 12

C0012833 GO:0043666 1 12

C0012833 GO:0006895 1 12

C0012833 GO:0010718 1 12

C0012833 GO:0010717 1 12

C0012833 GO:0042267 2 12

C0012833 GO:0043154 2 12

C0012833 GO:0015385 1 12

C0012833 GO:0006109 1 12

C0012833 GO:0016796 1 12

C0012833 GO:0048306 1 12

C0012833 GO:0006733 2 12

C0012833 GO:0016799 1 12

C0012833 GO:0001945 1 12

C0012833 GO:0042401 1 12

C0012833 GO:0004859 1 12

C0012833 GO:0007041 1 12

C0012833 GO:0032963 1 12

C0012833 GO:0005523 1 12

C0012833 GO:0030665 1 12

C0012833 GO:0030201 1 12

C0012833 GO:0016709 1 12

C0012833 GO:0016706 1 12

C0012833 GO:0004527 1 12

C0012833 GO:0004521 1 12

C0012833 GO:0004520 1 12

C0012833 GO:0010769 1 12

C0012833 GO:0015166 1 12

C0012833 GO:0015165 2 12

C0012833 GO:0048568 1 12

C0012833 GO:0031397 1 12

C0012833 GO:0021953 1 12

C0012833 GO:0032404 2 12

C0012833 GO:0005355 1 12

C0012833 GO:0042162 1 12

C0012833 GO:0009925 1 12

C0012833 GO:0021954 1 12

C0012833 GO:0014065 2 12

C0012833 GO:0043574 1 12

C0012833 GO:0043473 1 12

C0012833 GO:0007031 1 12

C0012833 GO:0002460 1 12

C0012833 GO:0034405 1 12

C0012833 GO:0009084 1 12

C0012833 GO:0016018 2 12

C0012833 GO:0005451 1 12

C0012833 GO:0009081 1 12

C0012833 GO:0009083 1 12

C0012833 GO:0004177 1 12

C0012833 GO:0051384 1 12

C0012833 GO:0050868 1 12

C0012833 GO:0033135 1 12

C0012833 GO:0030983 2 12

C0012833 GO:0046040 2 12

C0012833 GO:0009109 1 12

C0012833 GO:0051147 1 12

C0012833 GO:0051148 1 12

C0012833 GO:0001523 1 12

C0012833 GO:0006376 1 12

C0012833 GO:0016846 2 12

C0012833 GO:0016840 1 12

C0012833 GO:0000060 1 12

C0012833 GO:0005814 1 12

C0012833 GO:0042787 1 12

C0012833 GO:0016246 1 12

C0012833 GO:0010810 1 12

C0012833 GO:0010812 3 12

C0012833 GO:0050690 1 12

C0012833 GO:0016903 1 12

C0012833 GO:0030431 1 12

C0012833 GO:0016909 1 12

C0012833 GO:0035295 1 12

C0012833 GO:0003709 1 12

C0012833 GO:0031994 1 12

C0012833 GO:0051881 1 12

C0012833 GO:0070717 1 12

C0012833 GO:0002793 1 12

C0012833 GO:0032202 1 12

C0012833 GO:0006390 1 12

C0012833 GO:0044403 1 12

C0012833 GO:0008290 1 12

C0012833 GO:0043548 1 12

C0012833 GO:0005786 1 12

C0012833 GO:0000245 1 12

C0012833 GO:0042364 1 12

C0012833 GO:0009218 1 12

C0012833 GO:0032387 1 12

C0012833 GO:0048469 1 12

C0012833 GO:0006476 1 12

C0012833 GO:0031231 2 12

C0012833 GO:0060491 1 12

C0012833 GO:0030119 1 12

C0012833 GO:0045055 1 12

C0012833 GO:0005779 2 12

C0012833 GO:0016627 1 12

C0012833 GO:0070001 1 12

C0012833 GO:0048512 1 12

C0012833 GO:0002685 1 12

C0012833 GO:0006073 1 12

C0012833 GO:0005678 1 12

C0012833 GO:0000099 1 12

C0012833 GO:0008634 2 12

C0012833 GO:0032153 1 12

C0012833 GO:0032155 1 12

C0012833 GO:0031274 2 12

C0012833 GO:0043535 1 12

C0012833 GO:0045187 1 12

C0012833 GO:0019905 1 12

C0012833 GO:0007172 2 12

C0012833 GO:0031272 2 12

C0012833 GO:0055117 1 12

C0012833 GO:0007569 1 12

C0012833 GO:0030618 1 12

C0012833 GO:0050732 1 12

C0012833 GO:0016471 1 12

C0012833 GO:0015012 1 12

C0012833 GO:0050927 1 12

C0012833 GO:0070507 1 12

C0012833 GO:0006672 2 12

C0012833 GO:0045494 1 12

C0012833 GO:0022602 1 12

C0012833 GO:0042398 1 12

C0012833 GO:0016278 1 12

C0012833 GO:0016279 1 12

C0012833 GO:0016885 1 12

C0012833 GO:0031576 1 12

C0012833 GO:0005852 1 12

C0012833 GO:0090100 1 12

C0012833 GO:0090101 1 12

C0012833 GO:0000149 1 12

C0012833 GO:0031674 1 12

C0012833 GO:0051896 1 12

C0012833 GO:0016575 1 12

C0012833 GO:0016574 1 12

C0012833 GO:0005522 3 12

C0012833 GO:0048641 2 12

C0012833 GO:0016578 1 12

C0012833 GO:0006818 1 12

C0012833 GO:0055029 2 12

C0012833 GO:0035251 1 12

C0012833 GO:0030276 1 12

C0012833 GO:0006188 2 12

C0012833 GO:0034713 1 12

C0012833 GO:0042301 1 12

C0012833 GO:0000738 1 12

C0012833 GO:0000737 1 12

C0012833 GO:0016830 1 12

C0012833 GO:0016831 1 12

C0012833 GO:0006739 1 12

C0012833 GO:0006189 2 12

C0012833 GO:0033176 1 12

C0012833 GO:0005545 1 12

C0012833 GO:0045580 1 12

C0012833 GO:0045582 2 12

C0012833 GO:0046677 1 12

C0012833 GO:0030018 1 12

C0012833 GO:0034399 2 12

C0012833 GO:0001701 1 12

C0012833 GO:0001707 1 12

C0012833 GO:0001704 1 12

C0012833 GO:0051087 1 12

C0012833 GO:0008629 1 12

C0012833 GO:0070761 2 12

C0012833 GO:0046364 1 12

C0012833 GO:0008603 1 12

C0012833 GO:0017156 1 12

C0012833 GO:0048332 1 12

C0012833 GO:0006721 1 12

C0012833 GO:0031333 1 12

C0012833 GO:0000217 1 12

C0012833 GO:0004860 1 12

C0012833 GO:0004864 1 12

C0012833 GO:0030121 1 12

C0012833 GO:0030656 1 12

C0012833 GO:0070688 1 12

C0012833 GO:0050678 1 12

C0012833 GO:0050715 1 12

C0012833 GO:0015884 1 12

C0012833 GO:0006383 1 12

C0012833 GO:0032182 1 12

C0012833 GO:0001952 1 12

C0012833 GO:0032479 2 12

C0012833 GO:0010770 1 12

C0012833 GO:0002039 1 12

C0012833 GO:0042054 1 12

C0012833 GO:0042116 1 12

C0012833 GO:0002758 1 12

C0012833 GO:0005161 1 12

C0012833 GO:0034508 1 12

C0012833 GO:0070822 1 12

C0012833 GO:0032981 1 12

C0012833 GO:0045766 2 12

C0012833 GO:0030134 1 12

C0012833 GO:0016229 1 12

C0012833 GO:0006911 2 12

C0012833 GO:0009112 1 12

C0012833 GO:0009116 2 12

C0012833 GO:0002718 1 12

C0012833 GO:0070925 1 12

C0012833 GO:0000777 1 12

C0012833 GO:0030166 1 12

C0012833 GO:0000178 1 12

C0012833 GO:0001516 1 12

C0012833 GO:0000175 1 12

C0012833 GO:0046457 1 12

C0012833 GO:0046456 1 12

C0012833 GO:0043596 2 12

C0012833 GO:0030902 1 12

C0012833 GO:0046519 2 12

C0012833 GO:0016504 1 12

C0012833 GO:0016505 1 12

C0012833 GO:0007090 1 12

C0012833 GO:0008589 1 12

C0012833 GO:0031114 1 12

C0012833 GO:0046638 2 12

C0012833 GO:0046637 1 12

C0012833 GO:0051287 1 12

C0012833 GO:0046635 1 12

C0012833 GO:0046634 1 12

C0012833 GO:0046631 1 12

C0012833 GO:0050926 1 12

C0012833 GO:0008276 2 12

C0012833 GO:0050920 2 12

C0012833 GO:0050921 2 12

C0012833 GO:0051893 1 12

C0012833 GO:0034765 1 12

C0012833 GO:0008376 1 12

C0012833 GO:0008278 2 12

C0012833 GO:0034762 1 12

C0012833 GO:0031984 1 12

C0012833 GO:0006983 2 12

C0012833 GO:0044419 1 12

C0012833 GO:0006266 1 12

C0012833 GO:0006268 2 12

C0012833 GO:0019722 1 12

C0012833 GO:0031960 1 12

C0012833 GO:0000314 1 12

C0012833 GO:0006769 1 12

C0012833 GO:0051592 1 12

C0012833 GO:0030165 1 12

C0012833 GO:0010975 1 12

C0012833 GO:0030515 1 12

C0012833 GO:0030511 1 12

C0012833 GO:0070584 1 12

C0012833 GO:0005066 1 12

C0012833 GO:0051354 1 12

C0012833 GO:0005663 2 12

C0012833 GO:0005662 2 12

C0012833 GO:0005665 1 12

C0012833 GO:0048500 1 12

C0012833 GO:0005669 1 12

C0012833 GO:0019438 1 12

C0012833 GO:0030194 1 12

C0012833 GO:0000428 2 12

C0012833 GO:0002821 1 12

C0012833 GO:0032648 1 12

C0012833 GO:0005086 1 12

C0012833 GO:0042745 1 12

C0012833 GO:0043425 1 12

C0012833 GO:0007064 1 12

C0012833 GO:0031369 1 12

C0012833 GO:0042749 1 12

C0012833 GO:0000381 1 12

C0012833 GO:0035085 1 12

C0012833 GO:0015149 1 12

C0012833 GO:0019239 4 12

C0012833 GO:0002366 1 12

C0012833 GO:0006687 1 12

C0012833 GO:0006684 1 12

C0012833 GO:0006688 1 12

C0012833 GO:0015145 1 12

C0012833 GO:0043168 1 12

C0012833 GO:0030894 2 12

C0012833 GO:0046545 1 12

C0012833 GO:0009156 2 12

C0012833 GO:0005666 2 12

C0012833 GO:0033764 1 12

C0012833 GO:0030898 1 12

C0012833 GO:0007026 1 12

C0012833 GO:0048256 1 12

C0012833 GO:0000038 1 12

C0012833 GO:0014003 1 12

C0012833 GO:0000030 1 12

C0012833 GO:0045165 1 12

C0012833 GO:0000132 1 12

C0012833 GO:0007250 1 12

C0012833 GO:0004683 1 12

C0012833 GO:0031647 1 12

C0012833 GO:0033261 1 12

C0012833 GO:0045736 2 12

C0012833 GO:0045739 2 12

C0012833 GO:0015665 1 12

C0012833 GO:0022410 1 12

C0012833 GO:0002819 2 12

C0012833 GO:0030261 1 12

C0012833 GO:0005905 1 12

C0012833 GO:0043087 1 12

C0012833 GO:0009127 2 12

C0012833 GO:0006220 1 12

C0012833 GO:0006221 1 12

C0012833 GO:0000725 1 12

C0012833 GO:0000724 1 12

C0012833 GO:0042772 1 12

C0012833 GO:0042921 1 12

C0012833 GO:0046784 4 12

C0012833 GO:0046782 1 12

C0012833 GO:0044452 1 12

C0012833 GO:0008385 2 12

C0012833 GO:0022612 1 12

C0012833 GO:0022616 1 12

C0012833 GO:0010939 1 12

C0012833 GO:0032273 1 12

C0012833 GO:0015298 1 12

C0012833 GO:0006298 2 12

C0012833 GO:0002224 1 12

C0012833 GO:0010832 2 12

C0012833 GO:0008209 1 12

C0012833 GO:0010830 2 12

C0012833 GO:0042475 1 12

C0012833 GO:0008206 1 12

C0012833 GO:0051092 2 12

C0012833 GO:0043601 2 12

C0012833 GO:0008630 1 12

C0012833 GO:0007567 1 12

C0012833 GO:0070776 1 12

C0012833 GO:0070775 1 12

C0012833 GO:0003727 1 12

C0012833 GO:0043370 1 12

C0012833 GO:0000185 1 12

C0012833 GO:0000347 4 12

C0012833 GO:0000346 4 12

C0012833 GO:0042312 1 12

C0012833 GO:0016646 1 12

C0012833 GO:0016645 1 12

C0012833 GO:0008526 1 12

C0012833 GO:0070412 1 12

C0012833 GO:0070410 1 12

C0012833 GO:0006406 1 12

C0012833 GO:0070325 1 12

C0012833 GO:0048742 2 12

C0012833 GO:0019104 1 12

C0012833 GO:0005881 1 12

C0012833 GO:0042974 1 12

C0012833 GO:0005883 1 12

C0012833 GO:0016049 1 12

C0012833 GO:0002444 2 12

C0012833 GO:0002446 2 12

C0012833 GO:0042471 1 12

C0012833 GO:0030658 1 12

C0012833 GO:0016580 1 12

C0012833 GO:0016581 2 12

C0012833 GO:0001938 1 12

C0012833 GO:0033344 1 12

C0012833 GO:0002250 1 12

C0012833 GO:0015030 1 12

C0012833 GO:0001937 1 12

C0012833 GO:0001936 1 12

C0002170 GO:0048066 1 8

C0002170 GO:0002706 1 8

C0002170 GO:0002703 1 8

C0002170 GO:0034199 1 8

C0002170 GO:0016864 1 8

C0002170 GO:0016862 1 8

C0002170 GO:0051653 1 8

C0002170 GO:0016860 3 8

C0002170 GO:0000002 1 8

C0002170 GO:0043206 2 8

C0002170 GO:0005834 2 8

C0002170 GO:0004653 2 8

C0002170 GO:0006309 3 8

C0002170 GO:0007080 1 8

C0002170 GO:0030856 1 8

C0002170 GO:0008484 3 8

C0002170 GO:0016514 1 8

C0002170 GO:0051294 1 8

C0002170 GO:0003923 2 8

C0002170 GO:0002822 1 8

C0002170 GO:0051293 1 8

C0002170 GO:0002821 1 8

C0002170 GO:0002673 1 8

C0002170 GO:0000718 1 8

C0002170 GO:0006278 1 8

C0002170 GO:0016812 1 8

C0002170 GO:0045259 2 8

C0002170 GO:0016814 3 8

C0002170 GO:0010884 1 8

C0002170 GO:0010883 1 8

C0002170 GO:0003995 2 8

C0002170 GO:0010888 1 8

C0002170 GO:0019320 1 8

C0002170 GO:0032412 1 8

C0002170 GO:0000302 3 8

C0002170 GO:0030174 1 8

C0002170 GO:0030170 1 8

C0002170 GO:0035004 1 8

C0002170 GO:0005487 1 8

C0002170 GO:0051224 1 8

C0002170 GO:0003205 2 8

C0002170 GO:0003206 2 8

C0002170 GO:0015992 2 8

C0002170 GO:0042551 2 8

C0002170 GO:0006885 1 8

C0002170 GO:0003208 2 8

C0002170 GO:0042558 1 8

C0002170 GO:0042559 1 8

C0002170 GO:0009304 1 8

C0002170 GO:0048709 1 8

C0002170 GO:0033764 1 8

C0002170 GO:0009260 2 8

C0002170 GO:0009262 1 8

C0002170 GO:0009264 1 8

C0002170 GO:0009266 1 8

C0002170 GO:0019395 1 8

C0002170 GO:0035303 1 8

C0002170 GO:0030675 1 8

C0002170 GO:0015781 1 8

C0002170 GO:0015780 1 8

C0002170 GO:0006691 1 8

C0002170 GO:0015074 1 8

C0002170 GO:0048365 1 8

C0002170 GO:0016676 3 8

C0002170 GO:0005007 1 8

C0002170 GO:0016675 3 8

C0002170 GO:0006026 1 8

C0002170 GO:0006027 1 8

C0002170 GO:0033613 1 8

C0002170 GO:0033209 1 8

C0002170 GO:0008144 2 8

C0002170 GO:0014003 1 8

C0002170 GO:0031228 1 8

C0002170 GO:0042177 2 8

C0002170 GO:0051453 1 8

C0002170 GO:0031498 1 8

C0002170 GO:0042645 1 8

C0002170 GO:0051457 1 8

C0002170 GO:0009395 1 8

C0002170 GO:0009394 1 8

C0002170 GO:0005506 1 8

C0002170 GO:0005507 1 8

C0002170 GO:0033108 1 8

C0002170 GO:0055002 1 8

C0002170 GO:0042552 1 8

C0002170 GO:0006879 1 8

C0002170 GO:0042288 2 8

C0002170 GO:0050879 1 8

C0002170 GO:0055008 1 8

C0002170 GO:0051015 1 8

C0002170 GO:0001654 1 8

C0002170 GO:0046889 2 8

C0002170 GO:0033293 1 8

C0002170 GO:0044253 2 8

C0002170 GO:0006337 1 8

C0002170 GO:0022404 1 8

C0002170 GO:0016528 1 8

C0002170 GO:0016529 1 8

C0002170 GO:0004190 1 8

C0002170 GO:0016254 1 8

C0002170 GO:0019047 1 8

C0002170 GO:0017048 1 8

C0002170 GO:0042593 1 8

C0002170 GO:0006099 2 8

C0002170 GO:0022405 1 8

C0002170 GO:0042594 1 8

C0002170 GO:0008603 1 8

C0002170 GO:0006096 2 8

C0002170 GO:0009820 3 8

C0002170 GO:0033993 1 8

C0002170 GO:0017080 1 8

C0002170 GO:0019210 1 8

C0002170 GO:0019213 1 8

C0002170 GO:0045089 1 8

C0002170 GO:0006706 1 8

C0002170 GO:0048592 1 8

C0002170 GO:0000272 3 8

C0002170 GO:0031109 1 8

C0002170 GO:0000445 1 8

C0002170 GO:0003746 2 8

C0002170 GO:0009913 1 8

C0002170 GO:0001836 1 8

C0002170 GO:0050996 1 8

C0002170 GO:0005765 1 8

C0002170 GO:0005763 1 8

C0002170 GO:0048524 1 8

C0002170 GO:0019363 1 8

C0002170 GO:0019362 1 8

C0002170 GO:0034440 1 8

C0002170 GO:0042133 2 8

C0002170 GO:0000080 1 8

C0002170 GO:0009451 1 8

C0002170 GO:0043449 1 8

C0002170 GO:0003015 1 8

C0002170 GO:0019915 1 8

C0002170 GO:0042765 2 8

C0002170 GO:0016597 3 8

C0002170 GO:0009295 1 8

C0002170 GO:0007004 1 8

C0002170 GO:0042446 1 8

C0002170 GO:0004364 1 8

C0002170 GO:0010257 1 8

C0002170 GO:0005546 1 8

C0002170 GO:0015002 3 8

C0002170 GO:0034366 1 8

C0002170 GO:0016799 1 8

C0002170 GO:0016209 4 8

C0002170 GO:0000796 1 8

C0002170 GO:0009074 1 8

C0002170 GO:0051702 1 8

C0002170 GO:0051004 2 8

C0002170 GO:0045109 1 8

C0002170 GO:0016566 1 8

C0002170 GO:0004707 1 8

C0002170 GO:0004033 1 8

C0002170 GO:0018024 1 8

C0002170 GO:0006297 1 8

C0002170 GO:0017166 2 8

C0002170 GO:0015851 1 8

C0002170 GO:0006298 1 8

C0002170 GO:0008250 1 8

C0002170 GO:0048009 1 8

C0002170 GO:0046496 1 8

C0002170 GO:0030280 1 8

C0002170 GO:0051187 1 8

C0002170 GO:0051181 2 8

C0002170 GO:0051180 1 8

C0002170 GO:0016801 1 8

C0002170 GO:0007183 1 8

C0002170 GO:0030149 1 8

C0002170 GO:0000339 1 8

C0002170 GO:0032768 1 8

C0002170 GO:0032769 2 8

C0002170 GO:0019319 3 8

C0002170 GO:0003231 2 8

C0002170 GO:0030069 1 8

C0002170 GO:0008652 1 8

C0002170 GO:0043666 2 8

C0002170 GO:0042542 2 8

C0002170 GO:0022898 2 8

C0002170 GO:0010714 2 8

C0002170 GO:0010712 2 8

C0002170 GO:0070198 1 8

C0002170 GO:0046824 1 8

C0002170 GO:0006518 1 8

C0002170 GO:0019897 1 8

C0002170 GO:0006739 1 8

C0002170 GO:0006636 1 8

C0002170 GO:0006635 1 8

C0002170 GO:0019674 1 8

C0002170 GO:0030669 1 8

C0002170 GO:0009311 1 8

C0002170 GO:0070279 1 8

C0002170 GO:0032967 2 8

C0002170 GO:0004859 1 8

C0002170 GO:0032963 1 8

C0002170 GO:0001942 1 8

C0002170 GO:0004526 1 8

C0002170 GO:0031099 1 8

C0002170 GO:0004520 1 8

C0002170 GO:0050768 1 8

C0002170 GO:0050769 2 8

C0002170 GO:0032409 1 8

C0002170 GO:0015165 1 8

C0002170 GO:0006744 1 8

C0002170 GO:0006743 1 8

C0002170 GO:0006740 2 8

C0002170 GO:0005217 2 8

C0002170 GO:0007033 1 8

C0002170 GO:0031625 1 8

C0002170 GO:0008483 2 8

C0002170 GO:0032404 1 8

C0002170 GO:0005104 1 8

C0002170 GO:0004177 1 8

C0002170 GO:0050868 1 8

C0002170 GO:0055010 2 8

C0002170 GO:0046365 2 8

C0002170 GO:0046364 3 8

C0002170 GO:0016234 1 8

C0002170 GO:0009108 1 8

C0002170 GO:0009109 2 8

C0002170 GO:0034623 1 8

C0002170 GO:0034235 2 8

C0002170 GO:0031532 1 8

C0002170 GO:0004180 1 8

C0002170 GO:0000060 1 8

C0002170 GO:0018279 1 8

C0002170 GO:0070301 1 8

C0002170 GO:0045939 1 8

C0002170 GO:0050690 1 8

C0002170 GO:0022417 3 8

C0002170 GO:0016903 3 8

C0002170 GO:0055067 1 8

C0002170 GO:0007266 3 8

C0002170 GO:0051318 1 8

C0002170 GO:0031571 1 8

C0002170 GO:0003706 1 8

C0002170 GO:0070652 1 8

C0002170 GO:0006390 1 8

C0002170 GO:0006471 1 8

C0002170 GO:0048469 1 8

C0002170 GO:0005788 1 8

C0002170 GO:0030119 1 8

C0002170 GO:0030118 1 8

C0002170 GO:0006633 1 8

C0002170 GO:0005391 1 8

C0002170 GO:0007219 1 8

C0002170 GO:0016628 1 8

C0002170 GO:0016627 3 8

C0002170 GO:0007216 2 8

C0002170 GO:0016620 3 8

C0002170 GO:0070001 1 8

C0002170 GO:0010720 1 8

C0002170 GO:0010721 1 8

C0002170 GO:0019359 1 8

C0002170 GO:0005678 1 8

C0002170 GO:0015718 1 8

C0002170 GO:0043473 1 8

C0002170 GO:0005092 1 8

C0002170 GO:0005095 1 8

C0002170 GO:0043536 1 8

C0002170 GO:0007076 1 8

C0002170 GO:0003009 1 8

C0002170 GO:0008630 1 8

C0002170 GO:0005527 2 8

C0002170 GO:0004550 2 8

C0002170 GO:0033558 1 8

C0002170 GO:0006672 1 8

C0002170 GO:0006921 2 8

C0002170 GO:0009142 1 8

C0002170 GO:0009145 1 8

C0002170 GO:0016278 1 8

C0002170 GO:0016279 1 8

C0002170 GO:0009062 2 8

C0002170 GO:0048167 1 8

C0002170 GO:0009060 1 8

C0002170 GO:0016885 1 8

C0002170 GO:0009066 1 8

C0002170 GO:0009065 2 8

C0002170 GO:0048168 1 8

C0002170 GO:0031576 1 8

C0002170 GO:0042625 1 8

C0002170 GO:0005852 1 8

C0002170 GO:0005851 2 8

C0002170 GO:0031345 1 8

C0002170 GO:0031672 1 8

C0002170 GO:0007006 1 8

C0002170 GO:0016575 1 8

C0002170 GO:0005528 2 8

C0002170 GO:0005522 1 8

C0002170 GO:0016471 1 8

C0002170 GO:0004129 3 8

C0002170 GO:0004128 2 8

C0002170 GO:0006818 1 8

C0002170 GO:0016303 1 8

C0002170 GO:0001776 1 8

C0002170 GO:0045334 1 8

C0002170 GO:0042303 1 8

C0002170 GO:0008308 1 8

C0002170 GO:0044275 4 8

C0002170 GO:0000738 2 8

C0002170 GO:0000737 1 8

C0002170 GO:0004601 4 8

C0002170 GO:0004602 2 8

C0002170 GO:0032770 1 8

C0002170 GO:0010921 1 8

C0002170 GO:0045055 1 8

C0002170 GO:0017022 1 8

C0002170 GO:0010927 1 8

C0002170 GO:0060047 1 8

C0002170 GO:0003229 2 8

C0002170 GO:0003950 1 8

C0002170 GO:0042572 1 8

C0002170 GO:0060048 1 8

C0002170 GO:0045426 1 8

C0002170 GO:0030018 1 8

C0002170 GO:0050881 1 8

C0002170 GO:0015278 1 8

C0002170 GO:0001707 1 8

C0002170 GO:0001704 1 8

C0002170 GO:0051087 1 8

C0002170 GO:0042375 1 8

C0002170 GO:0015370 1 8

C0002170 GO:0006505 1 8

C0002170 GO:0070761 1 8

C0002170 GO:0006506 1 8

C0002170 GO:0017156 1 8

C0002170 GO:0043094 1 8

C0002170 GO:0048332 1 8

C0002170 GO:0031330 1 8

C0002170 GO:0004860 1 8

C0002170 GO:0030128 1 8

C0002170 GO:0030122 1 8

C0002170 GO:0030121 1 8

C0002170 GO:0030656 1 8

C0002170 GO:0030125 1 8

C0002170 GO:0050770 1 8

C0002170 GO:0001959 3 8

C0002170 GO:0050772 1 8

C0002170 GO:0032182 1 8

C0002170 GO:0008535 1 8

C0002170 GO:0005742 1 8

C0002170 GO:0000956 1 8

C0002170 GO:0005744 1 8

C0002170 GO:0016653 1 8

C0002170 GO:0006007 3 8

C0002170 GO:0034502 1 8

C0002170 GO:0042116 1 8

C0002170 GO:0005890 1 8

C0002170 GO:0043506 1 8

C0002170 GO:0032986 1 8

C0002170 GO:0070566 1 8

C0002170 GO:0032984 1 8

C0002170 GO:0032981 1 8

C0002170 GO:0031638 1 8

C0002170 GO:0005310 1 8

C0002170 GO:0046426 2 8

C0002170 GO:0046356 2 8

C0002170 GO:0030239 1 8

C0002170 GO:0007585 1 8

C0002170 GO:0016878 1 8

C0002170 GO:0034220 1 8

C0002170 GO:0042026 1 8

C0002170 GO:0009119 1 8

C0002170 GO:0070925 1 8

C0002170 GO:0006941 1 8

C0002170 GO:0000070 1 8

C0002170 GO:0006754 1 8

C0002170 GO:0019842 2 8

C0002170 GO:0043596 1 8

C0002170 GO:0033176 1 8

C0002170 GO:0046519 1 8

C0002170 GO:0051287 1 8

C0002170 GO:0016646 2 8

C0002170 GO:0055072 1 8

C0002170 GO:0045309 2 8

C0002170 GO:0034765 1 8

C0002170 GO:0008278 1 8

C0002170 GO:0034762 1 8

C0002170 GO:0018196 1 8

C0002170 GO:0015238 2 8

C0002170 GO:0010894 1 8

C0002170 GO:0010896 1 8

C0002170 GO:0006383 1 8

C0002170 GO:0032393 3 8

C0002170 GO:0009206 1 8

C0002170 GO:0009201 1 8

C0002170 GO:0000314 1 8

C0002170 GO:0006769 1 8

C0002170 GO:0010975 2 8

C0002170 GO:0030515 1 8

C0002170 GO:0005062 1 8

C0002170 GO:0051354 2 8

C0002170 GO:0005663 1 8

C0002170 GO:0043130 1 8

C0002170 GO:0005665 1 8

C0002170 GO:0019439 1 8

C0002170 GO:0060415 1 8

C0002170 GO:0042744 1 8

C0002170 GO:0042743 1 8

C0002170 GO:0005871 1 8

C0002170 GO:0031369 1 8

C0002170 GO:0055102 1 8

C0002170 GO:0004549 1 8

C0002170 GO:0000381 1 8

C0002170 GO:0008235 1 8

C0002170 GO:0008406 1 8

C0002170 GO:0019239 2 8

C0002170 GO:0005662 1 8

C0002170 GO:0016684 4 8

C0002170 GO:0016769 2 8

C0002170 GO:0030894 1 8

C0002170 GO:0005338 1 8

C0002170 GO:0009152 1 8

C0002170 GO:0048256 2 8

C0002170 GO:0042633 1 8

C0002170 GO:0000030 1 8

C0002170 GO:0045165 1 8

C0002170 GO:0000132 1 8

C0002170 GO:0030867 4 8

C0002170 GO:0031579 1 8

C0002170 GO:0045730 1 8

C0002170 GO:0045736 1 8

C0002170 GO:0016469 2 8

C0002170 GO:0002819 1 8

C0002170 GO:0030261 2 8

C0002170 GO:0030262 1 8

C0002170 GO:0051000 1 8

C0002170 GO:0048029 1 8

C0002170 GO:0009650 2 8

C0002170 GO:0044246 2 8

C0002170 GO:0006220 1 8

C0002170 GO:0070603 1 8

C0002170 GO:0001541 1 8

C0002170 GO:0006342 1 8

C0002170 GO:0046784 1 8

C0002170 GO:0046782 1 8

C0002170 GO:0046466 1 8

C0002170 GO:0003756 1 8

C0002170 GO:0010939 1 8

C0002170 GO:0003709 1 8

C0002170 GO:0019079 1 8

C0002170 GO:0046165 2 8

C0002170 GO:0055088 1 8

C0002170 GO:0030004 1 8

C0002170 GO:0010833 2 8

C0002170 GO:0051310 1 8

C0002170 GO:0055085 1 8

C0002170 GO:0006084 2 8

C0002170 GO:0008634 2 8

C0002170 GO:0008637 2 8

C0002170 GO:0043601 1 8

C0002170 GO:0006081 3 8

C0002170 GO:0043603 2 8

C0002170 GO:0042813 1 8

C0002170 GO:0045861 1 8

C0002170 GO:0034976 1 8

C0002170 GO:0000184 1 8

C0002170 GO:0019206 1 8

C0002170 GO:0055001 1 8

C0002170 GO:0000347 1 8

C0002170 GO:0000346 1 8

C0002170 GO:0030131 1 8

C0002170 GO:0030132 1 8

C0002170 GO:0030641 1 8

C0002170 GO:0040001 1 8

C0002170 GO:0033500 1 8

C0002170 GO:0016645 2 8

C0002170 GO:0008526 1 8

C0002170 GO:0005753 2 8

C0002170 GO:0010466 1 8

C0002170 GO:0006400 1 8

C0002170 GO:0008210 1 8

C0002170 GO:0019104 1 8

C0002170 GO:0005881 1 8

C0002170 GO:0007159 2 8

C0002170 GO:0007157 2 8

C0002170 GO:0002443 1 8

C0002170 GO:0051896 1 8

C0002170 GO:0042771 1 8

C0002170 GO:0016581 1 8

C0002170 GO:0033344 1 8

C0002170 GO:0004576 1 8

C0002170 GO:0001933 2 8

C0002170 GO:0004579 1 8

C0003123 GO:0051043 1 10

C0003123 GO:0006906 1 10

C0003123 GO:0048066 1 10

C0003123 GO:0001508 1 10

C0003123 GO:0016860 2 10

C0003123 GO:0043206 1 10

C0003123 GO:0005834 1 10

C0003123 GO:0004653 1 10

C0003123 GO:0006309 3 10

C0003123 GO:0030856 1 10

C0003123 GO:0016455 1 10

C0003123 GO:0003923 2 10

C0003123 GO:0003841 1 10

C0003123 GO:0035272 1 10

C0003123 GO:0000718 1 10

C0003123 GO:0016812 1 10

C0003123 GO:0045259 1 10

C0003123 GO:0016814 2 10

C0003123 GO:0043331 1 10

C0003123 GO:0003995 1 10

C0003123 GO:0019320 1 10

C0003123 GO:0009895 1 10

C0003123 GO:0032412 1 10

C0003123 GO:0048568 1 10

C0003123 GO:0000302 2 10

C0003123 GO:0030174 2 10

C0003123 GO:0030170 1 10

C0003123 GO:0008484 2 10

C0003123 GO:0051224 1 10

C0003123 GO:0030073 1 10

C0003123 GO:0003205 2 10

C0003123 GO:0003206 2 10

C0003123 GO:0015992 2 10

C0003123 GO:0042551 2 10

C0003123 GO:0006885 1 10

C0003123 GO:0003208 2 10

C0003123 GO:0042558 2 10

C0003123 GO:0051879 1 10

C0003123 GO:0007622 1 10

C0003123 GO:0009309 1 10

C0003123 GO:0009304 1 10

C0003123 GO:0032570 1 10

C0003123 GO:0009303 1 10

C0003123 GO:0009260 1 10

C0003123 GO:0007052 1 10

C0003123 GO:0009262 1 10

C0003123 GO:0009264 1 10

C0003123 GO:0009266 1 10

C0003123 GO:0019395 1 10

C0003123 GO:0035303 1 10

C0003123 GO:0030675 1 10

C0003123 GO:0015781 1 10

C0003123 GO:0015780 1 10

C0003123 GO:0006691 1 10

C0003123 GO:0070567 1 10

C0003123 GO:0005003 1 10

C0003123 GO:0016676 2 10

C0003123 GO:0005007 1 10

C0003123 GO:0016675 2 10

C0003123 GO:0006754 1 10

C0003123 GO:0033209 1 10

C0003123 GO:0008144 2 10

C0003123 GO:0031228 1 10

C0003123 GO:0042177 1 10

C0003123 GO:0051453 1 10

C0003123 GO:0031498 1 10

C0003123 GO:0042645 1 10

C0003123 GO:0051457 1 10

C0003123 GO:0030261 1 10

C0003123 GO:0009394 1 10

C0003123 GO:0045178 2 10

C0003123 GO:0030705 1 10

C0003123 GO:0005507 1 10

C0003123 GO:0006879 1 10

C0003123 GO:0008329 1 10

C0003123 GO:0055008 1 10

C0003123 GO:0002637 1 10

C0003123 GO:0005913 1 10

C0003123 GO:0046889 1 10

C0003123 GO:0044253 1 10

C0003123 GO:0006337 1 10

C0003123 GO:0032369 1 10

C0003123 GO:0004190 1 10

C0003123 GO:0003899 1 10

C0003123 GO:0019047 1 10

C0003123 GO:0008213 1 10

C0003123 GO:0006099 1 10

C0003123 GO:0007270 1 10

C0003123 GO:0006090 1 10

C0003123 GO:0006096 1 10

C0003123 GO:0009820 1 10

C0003123 GO:0033993 1 10

C0003123 GO:0045814 1 10

C0003123 GO:0019210 1 10

C0003123 GO:0019213 2 10

C0003123 GO:0045089 1 10

C0003123 GO:0019218 1 10

C0003123 GO:0000272 2 10

C0003123 GO:0030532 1 10

C0003123 GO:0001836 1 10

C0003123 GO:0050996 1 10

C0003123 GO:0005763 1 10

C0003123 GO:0048524 1 10

C0003123 GO:0019362 2 10

C0003123 GO:0034440 1 10

C0003123 GO:0042133 1 10

C0003123 GO:0000080 1 10

C0003123 GO:0043449 1 10

C0003123 GO:0003015 1 10

C0003123 GO:0042765 2 10

C0003123 GO:0016597 3 10

C0003123 GO:0009295 1 10

C0003123 GO:0042446 1 10

C0003123 GO:0015002 2 10

C0003123 GO:0051119 1 10

C0003123 GO:0016209 4 10

C0003123 GO:0016893 2 10

C0003123 GO:0016891 2 10

C0003123 GO:0051702 1 10

C0003123 GO:0051004 1 10

C0003123 GO:0016566 1 10

C0003123 GO:0004707 1 10

C0003123 GO:0030262 1 10

C0003123 GO:0018024 1 10

C0003123 GO:0006297 1 10

C0003123 GO:0017166 1 10

C0003123 GO:0015851 1 10

C0003123 GO:0034062 1 10

C0003123 GO:0015858 1 10

C0003123 GO:0050820 1 10

C0003123 GO:0046496 2 10

C0003123 GO:0030280 1 10

C0003123 GO:0051181 3 10

C0003123 GO:0051180 1 10

C0003123 GO:0016801 1 10

C0003123 GO:0010149 1 10

C0003123 GO:0007183 1 10

C0003123 GO:0030149 1 10

C0003123 GO:0000339 1 10

C0003123 GO:0032769 2 10

C0003123 GO:0019319 2 10

C0003123 GO:0051238 1 10

C0003123 GO:0003231 2 10

C0003123 GO:0030069 1 10

C0003123 GO:0046148 1 10

C0003123 GO:0008652 1 10

C0003123 GO:0043666 1 10

C0003123 GO:0032368 1 10

C0003123 GO:0042542 1 10

C0003123 GO:0010717 1 10

C0003123 GO:0022898 2 10

C0003123 GO:0010714 1 10

C0003123 GO:0010712 1 10

C0003123 GO:0070198 1 10

C0003123 GO:0003746 1 10

C0003123 GO:0006633 1 10

C0003123 GO:0006739 1 10

C0003123 GO:0006636 1 10

C0003123 GO:0006635 1 10

C0003123 GO:0006733 1 10

C0003123 GO:0016799 2 10

C0003123 GO:0070279 1 10

C0003123 GO:0042401 1 10

C0003123 GO:0031307 1 10

C0003123 GO:0032967 1 10

C0003123 GO:0007043 1 10

C0003123 GO:0004521 2 10

C0003123 GO:0004520 1 10

C0003123 GO:0050769 1 10

C0003123 GO:0015166 1 10

C0003123 GO:0016667 1 10

C0003123 GO:0009925 1 10

C0003123 GO:0032404 1 10

C0003123 GO:0005355 1 10

C0003123 GO:0015165 1 10

C0003123 GO:0042162 1 10

C0003123 GO:0070301 2 10

C0003123 GO:0016278 1 10

C0003123 GO:0014065 1 10

C0003123 GO:0006595 1 10

C0003123 GO:0008483 1 10

C0003123 GO:0005513 1 10

C0003123 GO:0009084 1 10

C0003123 GO:0006740 1 10

C0003123 GO:0005104 1 10

C0003123 GO:0055010 2 10

C0003123 GO:0046365 1 10

C0003123 GO:0046364 2 10

C0003123 GO:0009109 2 10

C0003123 GO:0034623 1 10

C0003123 GO:0034235 2 10

C0003123 GO:0016846 1 10

C0003123 GO:0031532 1 10

C0003123 GO:0004180 1 10

C0003123 GO:0000060 2 10

C0003123 GO:0004675 1 10

C0003123 GO:0005217 1 10

C0003123 GO:0017119 1 10

C0003123 GO:0010810 1 10

C0003123 GO:0010812 1 10

C0003123 GO:0022417 1 10

C0003123 GO:0016903 1 10

C0003123 GO:0030431 1 10

C0003123 GO:0007266 2 10

C0003123 GO:0005851 1 10

C0003123 GO:0005662 1 10

C0003123 GO:0045806 1 10

C0003123 GO:0006479 1 10

C0003123 GO:0006775 1 10

C0003123 GO:0009218 1 10

C0003123 GO:0031970 1 10

C0003123 GO:0032387 1 10

C0003123 GO:0048469 1 10

C0003123 GO:0005788 1 10

C0003123 GO:0006779 1 10

C0003123 GO:0031050 1 10

C0003123 GO:0031579 1 10

C0003123 GO:0005391 1 10

C0003123 GO:0007219 1 10

C0003123 GO:0006783 1 10

C0003123 GO:0007213 1 10

C0003123 GO:0016627 1 10

C0003123 GO:0007216 1 10

C0003123 GO:0016620 2 10

C0003123 GO:0070001 1 10

C0003123 GO:0048512 1 10

C0003123 GO:0005678 1 10

C0003123 GO:0015718 2 10

C0003123 GO:0015711 1 10

C0003123 GO:0031274 1 10

C0003123 GO:0045187 1 10

C0003123 GO:0031272 1 10

C0003123 GO:0051096 1 10

C0003123 GO:0043603 1 10

C0003123 GO:0005527 2 10

C0003123 GO:0055072 1 10

C0003123 GO:0004550 2 10

C0003123 GO:0033558 1 10

C0003123 GO:0006672 1 10

C0003123 GO:0043525 1 10

C0003123 GO:0006921 2 10

C0003123 GO:0042398 1 10

C0003123 GO:0009142 1 10

C0003123 GO:0009145 1 10

C0003123 GO:0034379 1 10

C0003123 GO:0016279 1 10

C0003123 GO:0009062 1 10

C0003123 GO:0048167 1 10

C0003123 GO:0009066 1 10

C0003123 GO:0021700 1 10

C0003123 GO:0048168 1 10

C0003123 GO:0070918 1 10

C0003123 GO:0031576 1 10

C0003123 GO:0042625 1 10

C0003123 GO:0005852 1 10

C0003123 GO:0031571 1 10

C0003123 GO:0007006 1 10

C0003123 GO:0016575 1 10

C0003123 GO:0016574 1 10

C0003123 GO:0005528 2 10

C0003123 GO:0005522 1 10

C0003123 GO:0016471 1 10

C0003123 GO:0004129 2 10

C0003123 GO:0004128 2 10

C0003123 GO:0006818 1 10

C0003123 GO:0050810 1 10

C0003123 GO:0044275 2 10

C0003123 GO:0001570 1 10

C0003123 GO:0000738 1 10

C0003123 GO:0000737 1 10

C0003123 GO:0016831 1 10

C0003123 GO:0004601 3 10

C0003123 GO:0033176 1 10

C0003123 GO:0010921 1 10

C0003123 GO:0045055 1 10

C0003123 GO:0045580 1 10

C0003123 GO:0060047 1 10

C0003123 GO:0003229 2 10

C0003123 GO:0001953 1 10

C0003123 GO:0060048 1 10

C0003123 GO:0030018 1 10

C0003123 GO:0015278 1 10

C0003123 GO:0001707 1 10

C0003123 GO:0001704 1 10

C0003123 GO:0032376 1 10

C0003123 GO:0032370 1 10

C0003123 GO:0032373 1 10

C0003123 GO:0015370 1 10

C0003123 GO:0070761 1 10

C0003123 GO:0017156 1 10

C0003123 GO:0043094 1 10

C0003123 GO:0048332 1 10

C0003123 GO:0031330 1 10

C0003123 GO:0004860 2 10

C0003123 GO:0030121 1 10

C0003123 GO:0001959 2 10

C0003123 GO:0015884 1 10

C0003123 GO:0006383 1 10

C0003123 GO:0032182 1 10

C0003123 GO:0001952 1 10

C0003123 GO:0005024 1 10

C0003123 GO:0005744 1 10

C0003123 GO:0016653 1 10

C0003123 GO:0010770 1 10

C0003123 GO:0006007 1 10

C0003123 GO:0034502 1 10

C0003123 GO:0042116 1 10

C0003123 GO:0034508 1 10

C0003123 GO:0005890 1 10

C0003123 GO:0032986 1 10

C0003123 GO:0032984 1 10

C0003123 GO:0015074 1 10

C0003123 GO:0048365 1 10

C0003123 GO:0005005 1 10

C0003123 GO:0046426 1 10

C0003123 GO:0046356 1 10

C0003123 GO:0030330 1 10

C0003123 GO:0034614 1 10

C0003123 GO:0034220 1 10

C0003123 GO:0002718 1 10

C0003123 GO:0070925 1 10

C0003123 GO:0001533 1 10

C0003123 GO:0000178 1 10

C0003123 GO:0019842 1 10

C0003123 GO:0043596 1 10

C0003123 GO:0004602 1 10

C0003123 GO:0046519 1 10

C0003123 GO:0051287 1 10

C0003123 GO:0008276 1 10

C0003123 GO:0045309 1 10

C0003123 GO:0008376 1 10

C0003123 GO:0008278 1 10

C0003123 GO:0034762 1 10

C0003123 GO:0033628 1 10

C0003123 GO:0015238 1 10

C0003123 GO:0010894 1 10

C0003123 GO:0010896 1 10

C0003123 GO:0006268 1 10

C0003123 GO:0032393 1 10

C0003123 GO:0009206 1 10

C0003123 GO:0009201 1 10

C0003123 GO:0000314 1 10

C0003123 GO:0006769 2 10

C0003123 GO:0010975 1 10

C0003123 GO:0030515 1 10

C0003123 GO:0051354 2 10

C0003123 GO:0005663 1 10

C0003123 GO:0043130 1 10

C0003123 GO:0060415 1 10

C0003123 GO:0030194 1 10

C0003123 GO:0005086 1 10

C0003123 GO:0042745 1 10

C0003123 GO:0042744 2 10

C0003123 GO:0042743 1 10

C0003123 GO:0043425 1 10

C0003123 GO:0007064 1 10

C0003123 GO:0055067 1 10

C0003123 GO:0042749 1 10

C0003123 GO:0008408 1 10

C0003123 GO:0000381 1 10

C0003123 GO:0008235 1 10

C0003123 GO:0015149 1 10

C0003123 GO:0045939 1 10

C0003123 GO:0019239 2 10

C0003123 GO:0033014 1 10

C0003123 GO:0006684 1 10

C0003123 GO:0016684 3 10

C0003123 GO:0015145 1 10

C0003123 GO:0016769 1 10

C0003123 GO:0030894 1 10

C0003123 GO:0005337 1 10

C0003123 GO:0030898 1 10

C0003123 GO:0000030 1 10

C0003123 GO:0004683 1 10

C0003123 GO:0030867 2 10

C0003123 GO:0045736 1 10

C0003123 GO:0005246 1 10

C0003123 GO:0016469 1 10

C0003123 GO:0015665 1 10

C0003123 GO:0022410 1 10

C0003123 GO:0006941 1 10

C0003123 GO:0006942 1 10

C0003123 GO:0048029 1 10

C0003123 GO:0009650 1 10

C0003123 GO:0044246 1 10

C0003123 GO:0006220 2 10

C0003123 GO:0010883 1 10

C0003123 GO:0044452 1 10

C0003123 GO:0046466 1 10

C0003123 GO:0022612 1 10

C0003123 GO:0010939 1 10

C0003123 GO:0019079 1 10

C0003123 GO:0046165 1 10

C0003123 GO:0006298 1 10

C0003123 GO:0051318 1 10

C0003123 GO:0030004 1 10

C0003123 GO:0010833 1 10

C0003123 GO:0055085 1 10

C0003123 GO:0006084 1 10

C0003123 GO:0008634 1 10

C0003123 GO:0008637 1 10

C0003123 GO:0043601 1 10

C0003123 GO:0006081 2 10

C0003123 GO:0051095 1 10

C0003123 GO:0007567 1 10

C0003123 GO:0010718 1 10

C0003123 GO:0042813 1 10

C0003123 GO:0034976 1 10

C0003123 GO:0019200 1 10

C0003123 GO:0019206 1 10

C0003123 GO:0008630 2 10

C0003123 GO:0030641 1 10

C0003123 GO:0042288 1 10

C0003123 GO:0042516 1 10

C0003123 GO:0016646 2 10

C0003123 GO:0016645 1 10

C0003123 GO:0008526 1 10

C0003123 GO:0005753 1 10

C0003123 GO:0019104 2 10

C0003123 GO:0005883 1 10

C0003123 GO:0007159 1 10

C0003123 GO:0007157 1 10

C0003123 GO:0051896 1 10

C0003123 GO:0042771 2 10

C0003123 GO:0016581 1 10

C0003123 GO:0033344 1 10

C0003123 GO:0015030 1 10

C0003123 GO:0001933 1 10

C0003123 GO:0015036 1 10

C0003123 GO:0001936 1 10

C0004093 GO:0051043 1 16

C0004093 GO:0007598 1 16

C0004093 GO:0043954 2 16

C0004093 GO:0048066 2 16

C0004093 GO:0002706 1 16

C0004093 GO:0002703 2 16

C0004093 GO:0034199 1 16

C0004093 GO:0001508 1 16

C0004093 GO:0016864 1 16

C0004093 GO:0016862 1 16

C0004093 GO:0009168 1 16

C0004093 GO:0016860 2 16

C0004093 GO:0000002 2 16

C0004093 GO:0043206 2 16

C0004093 GO:0005834 2 16

C0004093 GO:0006978 1 16

C0004093 GO:0004653 2 16

C0004093 GO:0006309 2 16

C0004093 GO:0031513 1 16

C0004093 GO:0006471 1 16

C0004093 GO:0002366 1 16

C0004093 GO:0030856 1 16

C0004093 GO:0016514 1 16

C0004093 GO:0016455 1 16

C0004093 GO:0051294 1 16

C0004093 GO:0003923 2 16

C0004093 GO:0002822 2 16

C0004093 GO:0031109 1 16

C0004093 GO:0002821 1 16

C0004093 GO:0035270 1 16

C0004093 GO:0003841 1 16

C0004093 GO:0035272 1 16

C0004093 GO:0002673 1 16

C0004093 GO:0000718 1 16

C0004093 GO:0016812 1 16

C0004093 GO:0006270 1 16

C0004093 GO:0045259 2 16

C0004093 GO:0016814 2 16

C0004093 GO:0010887 2 16

C0004093 GO:0010885 1 16

C0004093 GO:0010884 2 16

C0004093 GO:0003995 3 16

C0004093 GO:0006378 1 16

C0004093 GO:0010888 1 16

C0004093 GO:0031958 1 16

C0004093 GO:0032412 2 16

C0004093 GO:0032182 1 16

C0004093 GO:0000302 2 16

C0004093 GO:0008483 2 16

C0004093 GO:0030170 1 16

C0004093 GO:0008484 2 16

C0004093 GO:0046651 1 16

C0004093 GO:0051224 1 16

C0004093 GO:0030073 1 16

C0004093 GO:0003205 1 16

C0004093 GO:0032091 1 16

C0004093 GO:0003206 1 16

C0004093 GO:0015992 1 16

C0004093 GO:0015491 1 16

C0004093 GO:0042551 2 16

C0004093 GO:0042255 1 16

C0004093 GO:0003208 2 16

C0004093 GO:0042558 1 16

C0004093 GO:0043028 1 16

C0004093 GO:0005657 1 16

C0004093 GO:0050792 1 16

C0004093 GO:0043021 2 16

C0004093 GO:0043022 2 16

C0004093 GO:0051879 1 16

C0004093 GO:0043027 1 16

C0004093 GO:0070169 1 16

C0004093 GO:0007622 1 16

C0004093 GO:0009304 2 16

C0004093 GO:0048709 1 16

C0004093 GO:0009303 1 16

C0004093 GO:0009260 1 16

C0004093 GO:0007052 1 16

C0004093 GO:0009262 1 16

C0004093 GO:0009264 1 16

C0004093 GO:0009266 1 16

C0004093 GO:0035303 1 16

C0004093 GO:0044042 1 16

C0004093 GO:0030675 1 16

C0004093 GO:0031080 1 16

C0004093 GO:0004536 1 16

C0004093 GO:0015781 1 16

C0004093 GO:0015780 1 16

C0004093 GO:0035095 1 16

C0004093 GO:0005003 2 16

C0004093 GO:0016676 3 16

C0004093 GO:0016675 3 16

C0004093 GO:0006752 1 16

C0004093 GO:0006026 1 16

C0004093 GO:0006027 1 16

C0004093 GO:0048256 1 16

C0004093 GO:0030880 2 16

C0004093 GO:0008143 1 16

C0004093 GO:0008144 3 16

C0004093 GO:0014003 1 16

C0004093 GO:0031498 1 16

C0004093 GO:0042645 1 16

C0004093 GO:0009395 1 16

C0004093 GO:0009394 1 16

C0004093 GO:0045178 1 16

C0004093 GO:0033275 1 16

C0004093 GO:0002228 1 16

C0004093 GO:0045621 1 16

C0004093 GO:0005506 1 16

C0004093 GO:0045622 1 16

C0004093 GO:0002221 1 16

C0004093 GO:0045749 1 16

C0004093 GO:0033108 2 16

C0004093 GO:0055002 1 16

C0004093 GO:0006073 1 16

C0004093 GO:0008329 1 16

C0004093 GO:0050879 1 16

C0004093 GO:0055008 1 16

C0004093 GO:0002637 1 16

C0004093 GO:0005913 1 16

C0004093 GO:0001656 1 16

C0004093 GO:0046889 1 16

C0004093 GO:0044253 1 16

C0004093 GO:0043666 2 16

C0004093 GO:0006337 1 16

C0004093 GO:0042542 2 16

C0004093 GO:0006099 2 16

C0004093 GO:0016528 1 16

C0004093 GO:0016529 1 16

C0004093 GO:0003899 3 16

C0004093 GO:0016254 1 16

C0004093 GO:0017046 1 16

C0004093 GO:0045923 1 16

C0004093 GO:0042267 1 16

C0004093 GO:0002685 1 16

C0004093 GO:0060021 1 16

C0004093 GO:0042593 1 16

C0004093 GO:0022404 1 16

C0004093 GO:0022405 1 16

C0004093 GO:0043631 1 16

C0004093 GO:0007270 1 16

C0004093 GO:0007271 1 16

C0004093 GO:0008603 2 16

C0004093 GO:0022409 1 16

C0004093 GO:0006090 1 16

C0004093 GO:0009820 2 16

C0004093 GO:0033993 1 16

C0004093 GO:0003730 2 16

C0004093 GO:0019210 1 16

C0004093 GO:0019213 2 16

C0004093 GO:0045089 1 16

C0004093 GO:0006706 1 16

C0004093 GO:0000272 2 16

C0004093 GO:0000445 2 16

C0004093 GO:0040017 1 16

C0004093 GO:0009913 1 16

C0004093 GO:0001836 1 16

C0004093 GO:0001837 1 16

C0004093 GO:0005765 1 16

C0004093 GO:0005689 1 16

C0004093 GO:0005763 1 16

C0004093 GO:0048525 1 16

C0004093 GO:0048524 2 16

C0004093 GO:0006413 1 16

C0004093 GO:0019362 1 16

C0004093 GO:0006633 1 16

C0004093 GO:0042133 1 16

C0004093 GO:0009593 1 16

C0004093 GO:0031123 1 16

C0004093 GO:0007612 1 16

C0004093 GO:0000080 2 16

C0004093 GO:0031264 1 16

C0004093 GO:0007163 1 16

C0004093 GO:0043525 2 16

C0004093 GO:0019915 1 16

C0004093 GO:0042765 2 16

C0004093 GO:0016597 2 16

C0004093 GO:0009295 1 16

C0004093 GO:0004364 1 16

C0004093 GO:0002263 1 16

C0004093 GO:0010257 2 16

C0004093 GO:0005546 1 16

C0004093 GO:0001909 1 16

C0004093 GO:0006733 1 16

C0004093 GO:0032135 1 16

C0004093 GO:0015002 3 16

C0004093 GO:0033032 2 16

C0004093 GO:0034366 1 16

C0004093 GO:0051119 1 16

C0004093 GO:0005310 1 16

C0004093 GO:0016209 4 16

C0004093 GO:0016893 3 16

C0004093 GO:0016891 3 16

C0004093 GO:0000796 1 16

C0004093 GO:0009074 2 16

C0004093 GO:0051702 1 16

C0004093 GO:0070279 1 16

C0004093 GO:0051004 2 16

C0004093 GO:0045109 1 16

C0004093 GO:0005828 1 16

C0004093 GO:0016566 1 16

C0004093 GO:0004707 1 16

C0004093 GO:0007043 1 16

C0004093 GO:0016444 1 16

C0004093 GO:0004033 1 16

C0004093 GO:0018024 1 16

C0004093 GO:0006297 1 16

C0004093 GO:0017166 1 16

C0004093 GO:0015851 1 16

C0004093 GO:0034062 3 16

C0004093 GO:0008250 1 16

C0004093 GO:0048009 1 16

C0004093 GO:0046496 1 16

C0004093 GO:0005929 1 16

C0004093 GO:0030280 1 16

C0004093 GO:0051184 1 16

C0004093 GO:0051187 1 16

C0004093 GO:0051181 2 16

C0004093 GO:0016801 1 16

C0004093 GO:0042098 1 16

C0004093 GO:0008088 1 16

C0004093 GO:0016896 1 16

C0004093 GO:0007183 1 16

C0004093 GO:0005851 1 16

C0004093 GO:0030149 1 16

C0004093 GO:0000339 1 16

C0004093 GO:0032768 1 16

C0004093 GO:0032769 1 16

C0004093 GO:0017015 1 16

C0004093 GO:0019319 2 16

C0004093 GO:0050795 1 16

C0004093 GO:0051896 1 16

C0004093 GO:0002889 1 16

C0004093 GO:0003231 1 16

C0004093 GO:0046148 1 16

C0004093 GO:0008652 1 16

C0004093 GO:0008656 1 16

C0004093 GO:0032368 1 16

C0004093 GO:0010718 1 16

C0004093 GO:0010717 1 16

C0004093 GO:0022898 2 16

C0004093 GO:0010714 1 16

C0004093 GO:0010712 1 16

C0004093 GO:0046824 1 16

C0004093 GO:0006518 1 16

C0004093 GO:0004527 1 16

C0004093 GO:0019897 2 16

C0004093 GO:0016796 1 16

C0004093 GO:0006636 1 16

C0004093 GO:0019674 1 16

C0004093 GO:0031099 1 16

C0004093 GO:0009311 1 16

C0004093 GO:0021510 1 16

C0004093 GO:0004520 1 16

C0004093 GO:0051646 1 16

C0004093 GO:0031307 1 16

C0004093 GO:0032967 1 16

C0004093 GO:0004859 1 16

C0004093 GO:0007041 1 16

C0004093 GO:0032963 1 16

C0004093 GO:0006695 1 16

C0004093 GO:0016706 1 16

C0004093 GO:0001942 1 16

C0004093 GO:0004521 3 16

C0004093 GO:0001945 1 16

C0004093 GO:0050769 2 16

C0004093 GO:0032409 1 16

C0004093 GO:0015166 1 16

C0004093 GO:0015165 1 16

C0004093 GO:0048568 1 16

C0004093 GO:0031397 1 16

C0004093 GO:0032405 1 16

C0004093 GO:0032404 3 16

C0004093 GO:0005355 1 16

C0004093 GO:0005217 1 16

C0004093 GO:0014065 2 16

C0004093 GO:0006595 2 16

C0004093 GO:0007033 1 16

C0004093 GO:0002460 1 16

C0004093 GO:0030174 1 16

C0004093 GO:0045069 1 16

C0004093 GO:0048167 1 16

C0004093 GO:0005513 1 16

C0004093 GO:0009060 1 16

C0004093 GO:0045619 1 16

C0004093 GO:0009084 1 16

C0004093 GO:0016018 3 16

C0004093 GO:0006740 2 16

C0004093 GO:0004177 2 16

C0004093 GO:0050868 1 16

C0004093 GO:0055010 2 16

C0004093 GO:0046365 1 16

C0004093 GO:0046364 2 16

C0004093 GO:0030983 1 16

C0004093 GO:0046040 1 16

C0004093 GO:0009109 3 16

C0004093 GO:0034623 1 16

C0004093 GO:0034235 2 16

C0004093 GO:0000175 1 16

C0004093 GO:0016846 1 16

C0004093 GO:0031532 1 16

C0004093 GO:0004180 1 16

C0004093 GO:0000060 1 16

C0004093 GO:0005814 1 16

C0004093 GO:0004675 1 16

C0004093 GO:0016248 1 16

C0004093 GO:0016246 1 16

C0004093 GO:0018279 1 16

C0004093 GO:0045930 1 16

C0004093 GO:0070301 2 16

C0004093 GO:0017119 1 16

C0004093 GO:0045939 2 16

C0004093 GO:0031124 1 16

C0004093 GO:0010812 1 16

C0004093 GO:0050690 1 16

C0004093 GO:0022417 2 16

C0004093 GO:0016903 2 16

C0004093 GO:0030431 1 16

C0004093 GO:0007266 2 16

C0004093 GO:0051318 1 16

C0004093 GO:0042346 1 16

C0004093 GO:0042345 1 16

C0004093 GO:0002793 1 16

C0004093 GO:0005662 1 16

C0004093 GO:0070652 1 16

C0004093 GO:0045806 1 16

C0004093 GO:0000149 2 16

C0004093 GO:0005786 2 16

C0004093 GO:0006775 1 16

C0004093 GO:0032434 1 16

C0004093 GO:0009218 1 16

C0004093 GO:0032387 1 16

C0004093 GO:0048469 1 16

C0004093 GO:0005788 1 16

C0004093 GO:0006779 1 16

C0004093 GO:0004955 1 16

C0004093 GO:0030501 1 16

C0004093 GO:0030118 1 16

C0004093 GO:0005391 1 16

C0004093 GO:0048863 1 16

C0004093 GO:0016628 1 16

C0004093 GO:0002687 1 16

C0004093 GO:0006783 1 16

C0004093 GO:0007213 1 16

C0004093 GO:0016627 2 16

C0004093 GO:0007216 1 16

C0004093 GO:0016620 2 16

C0004093 GO:0048512 1 16

C0004093 GO:0000381 1 16

C0004093 GO:0010720 1 16

C0004093 GO:0005678 1 16

C0004093 GO:0031901 1 16

C0004093 GO:0015718 2 16

C0004093 GO:0016799 2 16

C0004093 GO:0043473 2 16

C0004093 GO:0005092 1 16

C0004093 GO:0015711 1 16

C0004093 GO:0005095 2 16

C0004093 GO:0031274 1 16

C0004093 GO:0043535 1 16

C0004093 GO:0045187 1 16

C0004093 GO:0019905 1 16

C0004093 GO:0031272 1 16

C0004093 GO:0007076 1 16

C0004093 GO:0055117 1 16

C0004093 GO:0003009 1 16

C0004093 GO:0050732 1 16

C0004093 GO:0005527 1 16

C0004093 GO:0002377 1 16

C0004093 GO:0043523 1 16

C0004093 GO:0004550 1 16

C0004093 GO:0033558 1 16

C0004093 GO:0006672 2 16

C0004093 GO:0045494 2 16

C0004093 GO:0050921 1 16

C0004093 GO:0006921 3 16

C0004093 GO:0009142 1 16

C0004093 GO:0009145 1 16

C0004093 GO:0016278 1 16

C0004093 GO:0016279 1 16

C0004093 GO:0009062 1 16

C0004093 GO:0000783 1 16

C0004093 GO:0000782 1 16

C0004093 GO:0009066 1 16

C0004093 GO:0009065 2 16

C0004093 GO:0009064 1 16

C0004093 GO:0048168 1 16

C0004093 GO:0035004 1 16

C0004093 GO:0031576 1 16

C0004093 GO:0042625 1 16

C0004093 GO:0005852 4 16

C0004093 GO:0090100 1 16

C0004093 GO:0031571 1 16

C0004093 GO:0043548 1 16

C0004093 GO:0031672 1 16

C0004093 GO:0007006 1 16

C0004093 GO:0016575 1 16

C0004093 GO:0016574 1 16

C0004093 GO:0005528 1 16

C0004093 GO:0016571 1 16

C0004093 GO:0005522 1 16

C0004093 GO:0016471 1 16

C0004093 GO:0004129 3 16

C0004093 GO:0004128 3 16

C0004093 GO:0015908 1 16

C0004093 GO:0055029 2 16

C0004093 GO:0016303 1 16

C0004093 GO:0001776 1 16

C0004093 GO:0006189 1 16

C0004093 GO:0006188 1 16

C0004093 GO:0042303 1 16

C0004093 GO:0044275 3 16

C0004093 GO:0046902 1 16

C0004093 GO:0000738 2 16

C0004093 GO:0000737 1 16

C0004093 GO:0002758 1 16

C0004093 GO:0006739 1 16

C0004093 GO:0004601 3 16

C0004093 GO:0033176 1 16

C0004093 GO:0032770 1 16

C0004093 GO:0010921 1 16

C0004093 GO:0045055 1 16

C0004093 GO:0042772 1 16

C0004093 GO:0017022 1 16

C0004093 GO:0010927 1 16

C0004093 GO:0045580 2 16

C0004093 GO:0045582 2 16

C0004093 GO:0046677 1 16

C0004093 GO:0003229 2 16

C0004093 GO:0003950 1 16

C0004093 GO:0042572 1 16

C0004093 GO:0008235 1 16

C0004093 GO:0030018 1 16

C0004093 GO:0050881 1 16

C0004093 GO:0001707 1 16

C0004093 GO:0001704 1 16

C0004093 GO:0032376 1 16

C0004093 GO:0032370 1 16

C0004093 GO:0032373 1 16

C0004093 GO:0006505 1 16

C0004093 GO:0003756 1 16

C0004093 GO:0006506 1 16

C0004093 GO:0017156 2 16

C0004093 GO:0043094 1 16

C0004093 GO:0048332 1 16

C0004093 GO:0031333 1 16

C0004093 GO:0000217 2 16

C0004093 GO:0004860 2 16

C0004093 GO:0008091 1 16

C0004093 GO:0030656 1 16

C0004093 GO:0030125 1 16

C0004093 GO:0050770 1 16

C0004093 GO:0001959 3 16

C0004093 GO:0050772 1 16

C0004093 GO:0048365 1 16

C0004093 GO:0000428 2 16

C0004093 GO:0001952 1 16

C0004093 GO:0005024 1 16

C0004093 GO:0005744 1 16

C0004093 GO:0016653 3 16

C0004093 GO:0010770 1 16

C0004093 GO:0006007 1 16

C0004093 GO:0002039 1 16

C0004093 GO:0042116 1 16

C0004093 GO:0004532 1 16

C0004093 GO:0034508 2 16

C0004093 GO:0042113 1 16

C0004093 GO:0005890 1 16

C0004093 GO:0043506 1 16

C0004093 GO:0032986 1 16

C0004093 GO:0070822 2 16

C0004093 GO:0032984 1 16
[truncated: 542,858 more chars]
