# Supplementary material for: Building the process-drug–side effect network to discover the relationship between biological Processes and side effects
Source: BMC Bioinformatics. 2011 Mar 29;12(Suppl 2):S2. doi: 10.1186/1471-2105-12-S2-S2 (PMC3073182; doi:10.1186/1471-2105-12-S2-S2)
Supplement: Additional file 3 — This file contains down_regulated processes (T-score > 3.0) and related effects. First Column: Effect ID ( UMLS Concept ID) Second Column: Process ID ( Gene Ontology ID) Third Column: The number of drugs which affect to process and causing the side effect. Fourth Column: Total drugs which are causing the side effect. [file 1471-2105-12-S2-S2-S3.doc]

C0011991 GO:0051043 1 19

C0011991 GO:0051044 1 19

C0011991 GO:0030228 1 19

C0011991 GO:0001503 2 19

C0011991 GO:0002703 1 19

C0011991 GO:0002702 2 19

C0011991 GO:0046718 1 19

C0011991 GO:0016863 1 19

C0011991 GO:0016860 1 19

C0011991 GO:0006306 1 19

C0011991 GO:0006305 1 19

C0011991 GO:0006304 2 19

C0011991 GO:0000002 2 19

C0011991 GO:0018212 1 19

C0011991 GO:0018210 2 19

C0011991 GO:0031513 1 19

C0011991 GO:0004715 1 19

C0011991 GO:0016101 1 19

C0011991 GO:0009190 1 19

C0011991 GO:0045851 1 19

C0011991 GO:0002822 1 19

C0011991 GO:0008360 1 19

C0011991 GO:0005788 1 19

C0011991 GO:0042088 1 19

C0011991 GO:0016812 2 19

C0011991 GO:0051784 3 19

C0011991 GO:0004622 1 19

C0011991 GO:0021537 2 19

C0011991 GO:0009895 3 19

C0011991 GO:0032411 1 19

C0011991 GO:0000302 1 19

C0011991 GO:0010906 1 19

C0011991 GO:0046658 1 19

C0011991 GO:0000578 1 19

C0011991 GO:0051224 1 19

C0011991 GO:0030071 1 19

C0011991 GO:0003205 1 19

C0011991 GO:0003206 1 19

C0011991 GO:0043028 2 19

C0011991 GO:0046850 1 19

C0011991 GO:0006110 2 19

C0011991 GO:0045912 2 19

C0011991 GO:0043025 1 19

C0011991 GO:0043027 1 19

C0011991 GO:0019882 1 19

C0011991 GO:0019883 1 19

C0011991 GO:0007622 1 19

C0011991 GO:0005071 1 19

C0011991 GO:0048709 1 19

C0011991 GO:0005138 1 19

C0011991 GO:0043205 1 19

C0011991 GO:0035303 1 19

C0011991 GO:0004437 1 19

C0011991 GO:0030675 2 19

C0011991 GO:0050715 1 19

C0011991 GO:0050714 1 19

C0011991 GO:0050650 2 19

C0011991 GO:0050654 2 19

C0011991 GO:0070567 1 19

C0011991 GO:0006693 1 19

C0011991 GO:0006692 1 19

C0011991 GO:0015179 1 19

C0011991 GO:0006754 1 19

C0011991 GO:0048638 1 19

C0011991 GO:0033613 1 19

C0011991 GO:0008028 1 19

C0011991 GO:0043256 1 19

C0011991 GO:0042177 1 19

C0011991 GO:0004693 1 19

C0011991 GO:0005871 1 19

C0011991 GO:0051457 1 19

C0011991 GO:0031225 1 19

C0011991 GO:0007127 1 19

C0011991 GO:0045078 1 19

C0011991 GO:0043489 2 19

C0011991 GO:0045072 1 19

C0011991 GO:0030705 1 19

C0011991 GO:0045621 1 19

C0011991 GO:0005506 1 19

C0011991 GO:0006879 1 19

C0011991 GO:0008329 1 19

C0011991 GO:0045211 1 19

C0011991 GO:0001654 1 19

C0011991 GO:0017048 1 19

C0011991 GO:0010677 2 19

C0011991 GO:0060021 1 19

C0011991 GO:0051983 2 19

C0011991 GO:0042269 1 19

C0011991 GO:0004190 2 19

C0011991 GO:0004745 1 19

C0011991 GO:0017046 1 19

C0011991 GO:0018107 1 19

C0011991 GO:0019047 1 19

C0011991 GO:0018065 1 19

C0011991 GO:0018108 1 19

C0011991 GO:0060348 2 19

C0011991 GO:0045884 1 19

C0011991 GO:0008210 1 19

C0011991 GO:0007270 1 19

C0011991 GO:0006096 1 19

C0011991 GO:0070491 1 19

C0011991 GO:0032355 2 19

C0011991 GO:0001727 2 19

C0011991 GO:0032350 1 19

C0011991 GO:0042354 1 19

C0011991 GO:0032210 1 19

C0011991 GO:0010828 1 19

C0011991 GO:0070665 1 19

C0011991 GO:0070668 1 19

C0011991 GO:0019213 1 19

C0011991 GO:0021915 2 19

C0011991 GO:0070120 1 19

C0011991 GO:0048593 1 19

C0011991 GO:0000271 1 19

C0011991 GO:0010595 1 19

C0011991 GO:0010594 2 19

C0011991 GO:0005044 1 19

C0011991 GO:0008517 2 19

C0011991 GO:0050995 1 19

C0011991 GO:0050994 1 19

C0011991 GO:0043534 1 19

C0011991 GO:0051806 1 19

C0011991 GO:0006413 1 19

C0011991 GO:0019363 1 19

C0011991 GO:0032148 1 19

C0011991 GO:0015645 1 19

C0011991 GO:0005149 1 19

C0011991 GO:0007612 1 19

C0011991 GO:0007162 1 19

C0011991 GO:0007163 1 19

C0011991 GO:0043524 1 19

C0011991 GO:0019915 1 19

C0011991 GO:0031343 1 19

C0011991 GO:0045767 1 19

C0011991 GO:0016597 1 19

C0011991 GO:0004364 1 19

C0011991 GO:0030742 1 19

C0011991 GO:0004407 1 19

C0011991 GO:0002263 1 19

C0011991 GO:0045667 1 19

C0011991 GO:0045661 1 19

C0011991 GO:0045669 1 19

C0011991 GO:0033032 2 19

C0011991 GO:0043966 1 19

C0011991 GO:0030218 1 19

C0011991 GO:0051059 1 19

C0011991 GO:0001516 1 19

C0011991 GO:0046965 2 19

C0011991 GO:0051701 1 19

C0011991 GO:0009071 1 19

C0011991 GO:0019827 1 19

C0011991 GO:0005901 1 19

C0011991 GO:0004709 3 19

C0011991 GO:0045841 3 19

C0011991 GO:0050820 1 19

C0011991 GO:0001764 2 19

C0011991 GO:0005929 1 19

C0011991 GO:0048002 1 19

C0011991 GO:0030286 1 19

C0011991 GO:0007530 1 19

C0011991 GO:0006563 1 19

C0011991 GO:0046915 1 19

C0011991 GO:0016801 1 19

C0011991 GO:0008088 1 19

C0011991 GO:0001910 1 19

C0011991 GO:0044246 1 19

C0011991 GO:0004143 1 19

C0011991 GO:0009880 1 19

C0011991 GO:0020037 1 19

C0011991 GO:0032768 1 19

C0011991 GO:0017015 1 19

C0011991 GO:0050796 1 19

C0011991 GO:0060070 3 19

C0011991 GO:0030069 1 19

C0011991 GO:0046660 2 19

C0011991 GO:0009953 1 19

C0011991 GO:0009952 1 19

C0011991 GO:0006895 1 19

C0011991 GO:0010712 1 19

C0011991 GO:0043154 1 19

C0011991 GO:0017080 2 19

C0011991 GO:0006518 1 19

C0011991 GO:0043010 1 19

C0011991 GO:0042834 1 19

C0011991 GO:0002715 1 19

C0011991 GO:0016790 1 19

C0011991 GO:0019956 1 19

C0011991 GO:0002718 3 19

C0011991 GO:0016709 2 19

C0011991 GO:0010563 1 19

C0011991 GO:0015166 1 19

C0011991 GO:0002020 1 19

C0011991 GO:0031397 1 19

C0011991 GO:0005355 1 19

C0011991 GO:0042169 1 19

C0011991 GO:0007131 2 19

C0011991 GO:0043470 1 19

C0011991 GO:0043471 1 19

C0011991 GO:0002237 2 19

C0011991 GO:0034405 1 19

C0011991 GO:0016409 1 19

C0011991 GO:0016331 1 19

C0011991 GO:0030323 1 19

C0011991 GO:0030326 1 19

C0011991 GO:0005041 1 19

C0011991 GO:0016338 1 19

C0011991 GO:0009108 1 19

C0011991 GO:0002724 3 19

C0011991 GO:0002720 2 19

C0011991 GO:0007098 1 19

C0011991 GO:0045936 1 19

C0011991 GO:0045089 1 19

C0011991 GO:0050690 1 19

C0011991 GO:0022410 1 19

C0011991 GO:0035113 1 19

C0011991 GO:0007266 1 19

C0011991 GO:0045445 1 19

C0011991 GO:0006493 1 19

C0011991 GO:0051881 1 19

C0011991 GO:0005977 1 19

C0011991 GO:0051923 1 19

C0011991 GO:0003705 2 19

C0011991 GO:0003707 1 19

C0011991 GO:0044409 1 19

C0011991 GO:0060538 1 19

C0011991 GO:0044403 1 19

C0011991 GO:0046697 1 19

C0011991 GO:0005786 1 19

C0011991 GO:0000242 1 19

C0011991 GO:0006775 1 19

C0011991 GO:0001523 1 19

C0011991 GO:0031970 1 19

C0011991 GO:0021510 1 19

C0011991 GO:0006477 1 19

C0011991 GO:0030509 1 19

C0011991 GO:0031058 1 19

C0011991 GO:0045454 1 19

C0011991 GO:0015085 2 19

C0011991 GO:0005072 1 19

C0011991 GO:0048864 1 19

C0011991 GO:0015082 1 19

C0011991 GO:0016627 1 19

C0011991 GO:0005770 1 19

C0011991 GO:0007217 1 19

C0011991 GO:0042562 1 19

C0011991 GO:0070001 2 19

C0011991 GO:0048512 1 19

C0011991 GO:0019359 1 19

C0011991 GO:0043120 1 19

C0011991 GO:0006171 1 19

C0011991 GO:0070228 1 19

C0011991 GO:0005159 2 19

C0011991 GO:0005158 1 19

C0011991 GO:0043536 1 19

C0011991 GO:0042752 1 19

C0011991 GO:0045187 1 19

C0011991 GO:0007173 2 19

C0011991 GO:0046631 1 19

C0011991 GO:0048641 1 19

C0011991 GO:0070875 2 19

C0011991 GO:0008630 1 19

C0011991 GO:0060395 1 19

C0011991 GO:0016055 1 19

C0011991 GO:0060393 2 19

C0011991 GO:0002377 1 19

C0011991 GO:0055072 1 19

C0011991 GO:0033558 1 19

C0011991 GO:0009142 1 19

C0011991 GO:0009145 1 19

C0011991 GO:0006929 1 19

C0011991 GO:0000786 1 19

C0011991 GO:0009068 1 19

C0011991 GO:0021700 1 19

C0011991 GO:0031577 3 19

C0011991 GO:0045058 1 19

C0011991 GO:0031670 1 19

C0011991 GO:0043548 1 19

C0011991 GO:0031672 1 19

C0011991 GO:0005391 1 19

C0011991 GO:0045725 2 19

C0011991 GO:0030879 1 19

C0011991 GO:0015909 1 19

C0011991 GO:0030371 2 19

C0011991 GO:0035250 1 19

C0011991 GO:0030276 1 19

C0011991 GO:0050819 1 19

C0011991 GO:0042301 1 19

C0011991 GO:0044275 1 19

C0011991 GO:0008091 2 19

C0011991 GO:0046906 1 19

C0011991 GO:0050810 1 19

C0011991 GO:0032770 1 19

C0011991 GO:0010921 1 19

C0011991 GO:0017022 1 19

C0011991 GO:0045582 1 19

C0011991 GO:0046677 1 19

C0011991 GO:0042572 1 19

C0011991 GO:0008543 1 19

C0011991 GO:0050885 1 19

C0011991 GO:0032374 1 19

C0011991 GO:0001709 3 19

C0011991 GO:0032371 1 19

C0011991 GO:0032370 3 19

C0011991 GO:0032373 3 19

C0011991 GO:0008308 1 19

C0011991 GO:0032273 2 19

C0011991 GO:0043535 2 19

C0011991 GO:0006721 1 19

C0011991 GO:0006720 1 19

C0011991 GO:0031333 1 19

C0011991 GO:0000217 1 19

C0011991 GO:0048385 1 19

C0011991 GO:0005593 1 19

C0011991 GO:0000188 1 19

C0011991 GO:0050771 1 19

C0011991 GO:0050679 1 19

C0011991 GO:0015884 1 19

C0011991 GO:0044272 1 19

C0011991 GO:0050671 1 19

C0011991 GO:0051828 1 19

C0011991 GO:0006000 1 19

C0011991 GO:0005160 1 19

C0011991 GO:0042113 1 19

C0011991 GO:0043506 1 19

C0011991 GO:0045768 1 19

C0011991 GO:0032984 1 19

C0011991 GO:0043467 1 19

C0011991 GO:0015074 1 19

C0011991 GO:0002573 1 19

C0011991 GO:0004467 1 19

C0011991 GO:0015695 1 19

C0011991 GO:0007584 1 19

C0011991 GO:0003785 1 19

C0011991 GO:0051646 1 19

C0011991 GO:0001530 1 19

C0011991 GO:0016877 1 19

C0011991 GO:0045123 1 19

C0011991 GO:0045124 1 19

C0011991 GO:0046457 1 19

C0011991 GO:0046456 2 19

C0011991 GO:0030900 1 19

C0011991 GO:0007094 3 19

C0011991 GO:0050681 1 19

C0011991 GO:0008585 1 19

C0011991 GO:0050926 2 19

C0011991 GO:0050927 2 19

C0011991 GO:0051893 1 19

C0011991 GO:0008375 1 19

C0011991 GO:0051897 1 19

C0011991 GO:0051896 1 19

C0011991 GO:0007519 1 19

C0011991 GO:0044419 1 19

C0011991 GO:0043325 1 19

C0011991 GO:0010896 1 19

C0011991 GO:0010898 1 19

C0011991 GO:0045839 3 19

C0011991 GO:0030510 1 19

C0011991 GO:0009206 1 19

C0011991 GO:0009201 1 19

C0011991 GO:0070411 1 19

C0011991 GO:0030048 1 19

C0011991 GO:0030165 1 19

C0011991 GO:0030514 1 19

C0011991 GO:0010874 2 19

C0011991 GO:0010875 3 19

C0011991 GO:0016615 1 19

C0011991 GO:0045429 2 19

C0011991 GO:0051353 2 19

C0011991 GO:0005665 1 19

C0011991 GO:0046849 1 19

C0011991 GO:0006164 1 19

C0011991 GO:0019439 1 19

C0011991 GO:0043034 1 19

C0011991 GO:0030194 1 19

C0011991 GO:0007632 1 19

C0011991 GO:0019934 1 19

C0011991 GO:0051453 1 19

C0011991 GO:0042745 1 19

C0011991 GO:0034451 1 19

C0011991 GO:0032946 1 19

C0011991 GO:0051452 1 19

C0011991 GO:0032496 1 19

C0011991 GO:0042749 1 19

C0011991 GO:0007062 3 19

C0011991 GO:0002286 1 19

C0011991 GO:0002285 1 19

C0011991 GO:0004428 2 19

C0011991 GO:0045646 1 19

C0011991 GO:0030183 1 19

C0011991 GO:0050700 1 19

C0011991 GO:0015149 1 19

C0011991 GO:0002366 1 19

C0011991 GO:0006687 1 19

C0011991 GO:0015145 1 19

C0011991 GO:0071174 3 19

C0011991 GO:0046545 2 19

C0011991 GO:0071173 3 19

C0011991 GO:0030890 1 19

C0011991 GO:0048500 1 19

C0011991 GO:0008013 2 19

C0011991 GO:0043393 1 19

C0011991 GO:0048255 2 19

C0011991 GO:0014003 1 19

C0011991 GO:0043560 1 19

C0011991 GO:0045165 1 19

C0011991 GO:0007250 2 19

C0011991 GO:0030863 1 19

C0011991 GO:0031645 1 19

C0011991 GO:0030864 1 19

C0011991 GO:0033261 1 19

C0011991 GO:0045730 1 19

C0011991 GO:0045737 1 19

C0011991 GO:0030195 1 19

C0011991 GO:0015665 1 19

C0011991 GO:0051879 1 19

C0011991 GO:0004033 1 19

C0011991 GO:0032376 3 19

C0011991 GO:0055038 1 19

C0011991 GO:0055037 1 19

C0011991 GO:0006941 1 19

C0011991 GO:0030260 1 19

C0011991 GO:0005903 1 19

C0011991 GO:0052126 1 19

C0011991 GO:0033280 3 19

C0011991 GO:0043087 1 19

C0011991 GO:0009124 2 19

C0011991 GO:0006342 1 19

C0011991 GO:0046782 1 19

C0011991 GO:0005605 1 19

C0011991 GO:0015807 3 19

C0011991 GO:0031994 1 19

C0011991 GO:0001894 2 19

C0011991 GO:0001893 1 19

C0011991 GO:0008635 1 19

C0011991 GO:0006081 1 19

C0011991 GO:0043603 1 19

C0011991 GO:0045861 1 19

C0011991 GO:0048488 3 19

C0011991 GO:0019200 2 19

C0011991 GO:0046326 1 19

C0011991 GO:0052192 1 19

C0011991 GO:0010948 1 19

C0011991 GO:0045682 1 19

C0011991 GO:0030641 1 19

C0011991 GO:0045684 1 19

C0011991 GO:0050660 1 19

C0011991 GO:0008443 1 19

C0011991 GO:0030520 1 19

C0011991 GO:0031330 2 19

C0011991 GO:0010466 1 19

C0011991 GO:0070412 2 19

C0011991 GO:0046504 1 19

C0011991 GO:0070410 3 19

C0011991 GO:0031331 1 19

C0011991 GO:0010741 1 19

C0011991 GO:0034103 1 19

C0011991 GO:0048662 1 19

C0011991 GO:0048660 1 19

C0011991 GO:0048742 1 19

C0011991 GO:0042974 1 19

C0011991 GO:0031214 2 19

C0011991 GO:0002440 1 19

C0011991 GO:0007157 1 19

C0011991 GO:0001937 1 19

C0027497 GO:0051043 2 26

C0027497 GO:0051044 1 26

C0027497 GO:0030228 2 26

C0027497 GO:0050840 1 26

C0027497 GO:0046887 1 26

C0027497 GO:0001503 1 26

C0027497 GO:0002703 1 26

C0027497 GO:0001505 1 26

C0027497 GO:0034199 1 26

C0027497 GO:0002700 1 26

C0027497 GO:0009247 1 26

C0027497 GO:0048260 1 26

C0027497 GO:0016863 4 26

C0027497 GO:0016860 1 26

C0027497 GO:0006306 1 26

C0027497 GO:0006305 1 26

C0027497 GO:0006304 2 26

C0027497 GO:0000002 2 26

C0027497 GO:0042605 1 26

C0027497 GO:0018212 1 26

C0027497 GO:0018210 2 26

C0027497 GO:0031513 1 26

C0027497 GO:0004715 1 26

C0027497 GO:0046504 1 26

C0027497 GO:0060541 1 26

C0027497 GO:0006284 1 26

C0027497 GO:0045851 1 26

C0027497 GO:0005041 1 26

C0027497 GO:0002824 1 26

C0027497 GO:0002822 2 26

C0027497 GO:0002821 1 26

C0027497 GO:0007281 1 26

C0027497 GO:0035270 1 26

C0027497 GO:0046330 1 26

C0027497 GO:0005788 1 26

C0027497 GO:0007286 1 26

C0027497 GO:0030291 1 26

C0027497 GO:0030295 2 26

C0027497 GO:0030296 1 26

C0027497 GO:0042326 1 26

C0027497 GO:0034284 2 26

C0027497 GO:0016812 1 26

C0027497 GO:0070633 1 26

C0027497 GO:0016814 1 26

C0027497 GO:0050864 1 26

C0027497 GO:0051784 3 26

C0027497 GO:0004622 2 26

C0027497 GO:0002702 2 26

C0027497 GO:0021537 3 26

C0027497 GO:0019320 1 26

C0027497 GO:0060263 2 26

C0027497 GO:0032411 1 26

C0027497 GO:0050670 1 26

C0027497 GO:0006458 1 26

C0027497 GO:0009898 1 26

C0027497 GO:0010906 1 26

C0027497 GO:0046658 1 26

C0027497 GO:0043025 1 26

C0027497 GO:0000578 1 26

C0027497 GO:0010862 1 26

C0027497 GO:0051224 1 26

C0027497 GO:0030071 1 26

C0027497 GO:0002687 1 26

C0027497 GO:0002685 1 26

C0027497 GO:0006112 1 26

C0027497 GO:0006110 2 26

C0027497 GO:0045912 2 26

C0027497 GO:0051879 1 26

C0027497 GO:0043027 1 26

C0027497 GO:0048705 1 26

C0027497 GO:0019882 1 26

C0027497 GO:0019883 1 26

C0027497 GO:0007622 1 26

C0027497 GO:0005071 2 26

C0027497 GO:0005138 1 26

C0027497 GO:0050796 1 26

C0027497 GO:0060606 1 26

C0027497 GO:0042439 1 26

C0027497 GO:0046870 1 26

C0027497 GO:0043205 1 26

C0027497 GO:0035303 2 26

C0027497 GO:0004437 1 26

C0027497 GO:0030675 2 26

C0027497 GO:0050716 1 26

C0027497 GO:0050715 2 26

C0027497 GO:0050714 1 26

C0027497 GO:0050650 1 26

C0027497 GO:0050718 1 26

C0027497 GO:0050654 1 26

C0027497 GO:0015074 2 26

C0027497 GO:0006693 1 26

C0027497 GO:0006692 1 26

C0027497 GO:0015179 1 26

C0027497 GO:0006750 1 26

C0027497 GO:0015175 1 26

C0027497 GO:0015172 1 26

C0027497 GO:0048638 1 26

C0027497 GO:0050810 2 26

C0027497 GO:0030880 1 26

C0027497 GO:0033613 1 26

C0027497 GO:0008028 1 26

C0027497 GO:0048145 1 26

C0027497 GO:0033619 1 26

C0027497 GO:0046579 2 26

C0027497 GO:0031228 2 26

C0027497 GO:0042177 2 26

C0027497 GO:0042632 2 26

C0027497 GO:0004693 1 26

C0027497 GO:0005871 1 26

C0027497 GO:0043241 1 26

C0027497 GO:0031225 1 26

C0027497 GO:0007127 1 26

C0027497 GO:0045078 1 26

C0027497 GO:0043489 2 26

C0027497 GO:0045072 1 26

C0027497 GO:0030705 2 26

C0027497 GO:0033238 1 26

C0027497 GO:0033273 1 26

C0027497 GO:0045740 1 26

C0027497 GO:0045621 1 26

C0027497 GO:0005506 1 26

C0027497 GO:0045622 1 26

C0027497 GO:0030260 2 26

C0027497 GO:0002221 1 26

C0027497 GO:0006879 1 26

C0027497 GO:0008329 1 26

C0027497 GO:0042625 1 26

C0027497 GO:0008517 1 26

C0027497 GO:0015929 1 26

C0027497 GO:0045211 1 26

C0027497 GO:0046928 1 26

C0027497 GO:0001654 1 26

C0027497 GO:0018108 1 26

C0027497 GO:0045216 1 26

C0027497 GO:0010677 2 26

C0027497 GO:0046888 1 26

C0027497 GO:0060021 3 26

C0027497 GO:0034329 1 26

C0027497 GO:0051983 2 26

C0027497 GO:0042269 1 26

C0027497 GO:0006337 1 26

C0027497 GO:0004190 3 26

C0027497 GO:0007229 1 26

C0027497 GO:0017046 1 26

C0027497 GO:0018107 1 26

C0027497 GO:0019047 2 26

C0027497 GO:0030031 1 26

C0027497 GO:0018065 1 26

C0027497 GO:0030035 1 26

C0027497 GO:0017048 2 26

C0027497 GO:0060348 1 26

C0027497 GO:0022406 1 26

C0027497 GO:0045884 1 26

C0027497 GO:0045445 1 26

C0027497 GO:0007272 1 26

C0027497 GO:0010828 1 26

C0027497 GO:0070491 1 26

C0027497 GO:0032355 2 26

C0027497 GO:0001727 1 26

C0027497 GO:0033261 2 26

C0027497 GO:0042354 1 26

C0027497 GO:0006527 1 26

C0027497 GO:0006525 1 26

C0027497 GO:0006096 1 26

C0027497 GO:0070668 1 26

C0027497 GO:0005159 2 26

C0027497 GO:0046823 1 26

C0027497 GO:0045768 2 26

C0027497 GO:0019213 1 26

C0027497 GO:0006700 1 26

C0027497 GO:0021915 2 26

C0027497 GO:0070120 1 26

C0027497 GO:0048593 1 26

C0027497 GO:0032856 1 26

C0027497 GO:0006518 1 26

C0027497 GO:0001838 1 26

C0027497 GO:0010595 2 26

C0027497 GO:0010594 3 26

C0027497 GO:0040017 1 26

C0027497 GO:0005044 1 26

C0027497 GO:0032350 1 26

C0027497 GO:0050995 1 26

C0027497 GO:0050994 2 26

C0027497 GO:0016986 1 26

C0027497 GO:0032321 1 26

C0027497 GO:0055092 2 26

C0027497 GO:0051806 2 26

C0027497 GO:0043112 1 26

C0027497 GO:0006413 2 26

C0027497 GO:0048520 1 26

C0027497 GO:0019363 1 26

C0027497 GO:0008366 1 26

C0027497 GO:0032148 1 26

C0027497 GO:0042133 1 26

C0027497 GO:0009593 1 26

C0027497 GO:0005149 2 26

C0027497 GO:0007612 1 26

C0027497 GO:0048771 1 26

C0027497 GO:0007162 1 26

C0027497 GO:0050808 1 26

C0027497 GO:0043524 1 26

C0027497 GO:0032210 1 26

C0027497 GO:0019915 1 26

C0027497 GO:0031343 3 26

C0027497 GO:0045767 2 26

C0027497 GO:0016597 2 26

C0027497 GO:0042446 1 26

C0027497 GO:0004364 1 26

C0027497 GO:0045785 1 26

C0027497 GO:0004407 1 26

C0027497 GO:0002263 2 26

C0027497 GO:0005544 1 26

C0027497 GO:0055117 1 26

C0027497 GO:0045667 1 26

C0027497 GO:0045661 1 26

C0027497 GO:0045669 1 26

C0027497 GO:0010522 1 26

C0027497 GO:0033032 2 26

C0027497 GO:0051057 2 26

C0027497 GO:0016202 1 26

C0027497 GO:0043966 1 26

C0027497 GO:0030218 1 26

C0027497 GO:0005313 1 26

C0027497 GO:0051059 3 26

C0027497 GO:0051058 2 26

C0027497 GO:0046965 1 26

C0027497 GO:0009074 1 26

C0027497 GO:0051702 1 26

C0027497 GO:0051701 1 26

C0027497 GO:0042992 1 26

C0027497 GO:0019827 1 26

C0027497 GO:0002429 1 26

C0027497 GO:0042994 1 26

C0027497 GO:0045109 1 26

C0027497 GO:0006940 1 26

C0027497 GO:0006942 1 26

C0027497 GO:0016291 1 26

C0027497 GO:0004709 2 26

C0027497 GO:0045841 3 26

C0027497 GO:0004035 1 26

C0027497 GO:0008252 1 26

C0027497 GO:0050820 1 26

C0027497 GO:0001764 2 26

C0027497 GO:0005929 2 26

C0027497 GO:0048002 1 26

C0027497 GO:0030286 2 26

C0027497 GO:0007530 1 26

C0027497 GO:0051181 1 26

C0027497 GO:0051180 1 26

C0027497 GO:0046915 1 26

C0027497 GO:0008088 1 26

C0027497 GO:0001910 1 26

C0027497 GO:0004143 1 26

C0027497 GO:0007584 2 26

C0027497 GO:0030148 1 26

C0027497 GO:0020037 1 26

C0027497 GO:0032768 1 26

C0027497 GO:0032769 1 26

C0027497 GO:0019319 1 26

C0027497 GO:0050795 1 26

C0027497 GO:0002688 1 26

C0027497 GO:0060070 3 26

C0027497 GO:0030069 2 26

C0027497 GO:0046660 2 26

C0027497 GO:0009953 1 26

C0027497 GO:0009952 1 26

C0027497 GO:0006895 1 26

C0027497 GO:0032364 1 26

C0027497 GO:0006513 1 26

C0027497 GO:0017080 2 26

C0027497 GO:0002690 1 26

C0027497 GO:0003746 1 26

C0027497 GO:0043010 1 26

C0027497 GO:0042834 1 26

C0027497 GO:0003785 1 26

C0027497 GO:0019894 1 26

C0027497 GO:0016790 1 26

C0027497 GO:0019956 1 26

C0027497 GO:0009312 1 26

C0027497 GO:0002718 4 26

C0027497 GO:0031307 1 26

C0027497 GO:0009895 3 26

C0027497 GO:0007043 1 26

C0027497 GO:0001948 1 26

C0027497 GO:0050764 1 26

C0027497 GO:0046006 1 26

C0027497 GO:0005583 1 26

C0027497 GO:0010563 2 26

C0027497 GO:0050768 1 26

C0027497 GO:0001530 2 26

C0027497 GO:0010769 1 26

C0027497 GO:0015166 1 26

C0027497 GO:0006749 1 26

C0027497 GO:0031397 1 26

C0027497 GO:0022829 1 26

C0027497 GO:0005355 2 26

C0027497 GO:0042169 1 26

C0027497 GO:0008034 3 26

C0027497 GO:0043270 1 26

C0027497 GO:0007131 2 26

C0027497 GO:0043470 1 26

C0027497 GO:0043471 1 26

C0027497 GO:0009062 1 26

C0027497 GO:0002237 3 26

C0027497 GO:0045619 1 26

C0027497 GO:0034405 1 26

C0027497 GO:0045616 1 26

C0027497 GO:0005518 1 26

C0027497 GO:0016409 1 26

C0027497 GO:0004177 1 26

C0027497 GO:0016331 1 26

C0027497 GO:0030323 2 26

C0027497 GO:0030324 1 26

C0027497 GO:0050868 1 26

C0027497 GO:0030326 1 26

C0027497 GO:0051452 1 26

C0027497 GO:0046365 1 26

C0027497 GO:0046364 1 26

C0027497 GO:0050866 1 26

C0027497 GO:0016234 1 26

C0027497 GO:0009108 1 26

C0027497 GO:0034623 1 26

C0027497 GO:0034235 1 26

C0027497 GO:0002724 3 26

C0027497 GO:0002720 2 26

C0027497 GO:0004181 1 26

C0027497 GO:0016538 1 26

C0027497 GO:0042787 1 26

C0027497 GO:0030934 1 26

C0027497 GO:0007098 1 26

C0027497 GO:0045936 2 26

C0027497 GO:0010812 2 26

C0027497 GO:0045089 1 26

C0027497 GO:0030021 1 26

C0027497 GO:0016903 1 26

C0027497 GO:0022410 1 26

C0027497 GO:0035113 1 26

C0027497 GO:0043627 1 26

C0027497 GO:0007266 1 26

C0027497 GO:0035295 1 26

C0027497 GO:0043624 1 26

C0027497 GO:0009746 2 26

C0027497 GO:0043190 1 26

C0027497 GO:0005605 2 26

C0027497 GO:0006493 1 26

C0027497 GO:0051881 2 26

C0027497 GO:0042345 1 26

C0027497 GO:0051928 1 26

C0027497 GO:0002793 1 26

C0027497 GO:0051923 2 26

C0027497 GO:0003705 2 26

C0027497 GO:0009749 2 26

C0027497 GO:0044409 2 26

C0027497 GO:0060538 2 26

C0027497 GO:0044403 1 26

C0027497 GO:0046697 2 26

C0027497 GO:0008290 1 26

C0027497 GO:0005786 2 26

C0027497 GO:0000242 2 26

C0027497 GO:0005782 1 26

C0027497 GO:0031970 1 26

C0027497 GO:0032387 1 26

C0027497 GO:0021510 2 26

C0027497 GO:0006477 2 26

C0027497 GO:0030509 1 26

C0027497 GO:0031057 1 26

C0027497 GO:0031058 1 26

C0027497 GO:0015085 2 26

C0027497 GO:0005072 1 26

C0027497 GO:0048864 1 26

C0027497 GO:0043028 1 26

C0027497 GO:0007213 1 26

C0027497 GO:0005770 1 26

C0027497 GO:0016338 2 26

C0027497 GO:0042562 1 26

C0027497 GO:0070001 3 26

C0027497 GO:0048512 1 26

C0027497 GO:0046850 1 26

C0027497 GO:0006073 1 26

C0027497 GO:0010721 1 26

C0027497 GO:0031907 1 26

C0027497 GO:0019359 1 26

C0027497 GO:0032722 1 26

C0027497 GO:0070228 1 26

C0027497 GO:0032655 2 26

C0027497 GO:0030427 1 26

C0027497 GO:0032153 1 26

C0027497 GO:0005158 1 26

C0027497 GO:0032155 1 26

C0027497 GO:0042129 1 26

C0027497 GO:0043536 2 26

C0027497 GO:0042752 1 26

C0027497 GO:0045187 1 26

C0027497 GO:0007173 2 26

C0027497 GO:0007172 1 26

C0027497 GO:0048641 1 26

C0027497 GO:0070875 2 26

C0027497 GO:0060395 1 26

C0027497 GO:0016055 1 26

C0027497 GO:0060393 1 26

C0027497 GO:0002377 2 26

C0027497 GO:0007163 2 26

C0027497 GO:0004551 2 26

C0027497 GO:0033558 1 26

C0027497 GO:0030201 1 26

C0027497 GO:0006929 2 26

C0027497 GO:0008374 3 26

C0027497 GO:0000786 2 26

C0027497 GO:0031334 1 26

C0027497 GO:0021700 1 26

C0027497 GO:0031577 3 26

C0027497 GO:0090101 1 26

C0027497 GO:0031670 1 26

C0027497 GO:0043548 2 26

C0027497 GO:0005858 1 26

C0027497 GO:0005391 1 26

C0027497 GO:0046520 1 26

C0027497 GO:0005522 1 26

C0027497 GO:0045725 2 26

C0027497 GO:0045727 2 26

C0027497 GO:0030879 2 26

C0027497 GO:0055029 1 26

C0027497 GO:0030371 1 26

C0027497 GO:0035250 2 26

C0027497 GO:0030276 1 26

C0027497 GO:0030274 1 26

C0027497 GO:0042308 1 26

C0027497 GO:0030278 1 26

C0027497 GO:0050819 1 26

C0027497 GO:0042301 2 26

C0027497 GO:0044275 2 26

C0027497 GO:0002757 1 26

C0027497 GO:0008091 2 26

C0027497 GO:0002758 1 26

C0027497 GO:0043120 1 26

C0027497 GO:0044447 1 26

C0027497 GO:0008301 1 26

C0027497 GO:0032770 1 26

C0027497 GO:0015280 1 26

C0027497 GO:0010921 1 26

C0027497 GO:0031016 1 26

C0027497 GO:0019200 2 26

C0027497 GO:0017022 1 26

C0027497 GO:0045580 1 26

C0027497 GO:0045582 1 26

C0027497 GO:0046677 1 26

C0027497 GO:0001953 2 26

C0027497 GO:0042572 1 26

C0027497 GO:0008543 1 26

C0027497 GO:0043168 2 26

C0027497 GO:0007250 2 26

C0027497 GO:0032374 1 26

C0027497 GO:0009116 1 26

C0027497 GO:0001709 3 26

C0027497 GO:0032371 1 26

C0027497 GO:0032370 3 26

C0027497 GO:0032373 3 26

C0027497 GO:0005930 1 26

C0027497 GO:0032273 2 26

C0027497 GO:0060562 1 26

C0027497 GO:0017157 1 26

C0027497 GO:0044042 1 26

C0027497 GO:0019209 2 26

C0027497 GO:0043535 2 26

C0027497 GO:0048332 1 26

C0027497 GO:0031330 2 26

C0027497 GO:0031333 1 26

C0027497 GO:0000217 1 26

C0027497 GO:0004861 1 26

C0027497 GO:0008308 1 26

C0027497 GO:0048385 2 26

C0027497 GO:0005593 1 26

C0027497 GO:0043534 1 26

C0027497 GO:0050771 1 26

C0027497 GO:0050770 3 26

C0027497 GO:0000428 1 26

C0027497 GO:0050671 1 26

C0027497 GO:0051828 2 26

C0027497 GO:0032479 1 26

C0027497 GO:0006007 1 26

C0027497 GO:0006000 1 26

C0027497 GO:0042119 1 26

C0027497 GO:0016830 1 26

C0027497 GO:0005160 1 26

C0027497 GO:0005161 1 26

C0027497 GO:0042113 1 26

C0027497 GO:0043506 2 26

C0027497 GO:0032986 1 26

C0027497 GO:0032984 2 26

C0027497 GO:0043467 1 26

C0027497 GO:0070567 1 26

C0027497 GO:0045766 3 26

C0027497 GO:0002573 2 26

C0027497 GO:0015695 2 26

C0027497 GO:0045604 1 26

C0027497 GO:0015026 1 26

C0027497 GO:0016229 1 26

C0027497 GO:0050851 1 26

C0027497 GO:0050852 1 26

C0027497 GO:0034612 1 26

C0027497 GO:0007585 1 26

C0027497 GO:0009112 2 26

C0027497 GO:0042026 1 26

C0027497 GO:0046580 2 26

C0027497 GO:0051646 1 26

C0027497 GO:0034599 1 26

C0027497 GO:0034185 1 26

C0027497 GO:0016877 2 26

C0027497 GO:0000178 1 26

C0027497 GO:0045123 1 26

C0027497 GO:0008170 1 26

C0027497 GO:0045124 1 26

C0027497 GO:0031526 1 26

C0027497 GO:0030900 1 26

C0027497 GO:0007094 3 26

C0027497 GO:0016505 1 26

C0027497 GO:0035065 1 26

C0027497 GO:0046637 1 26

C0027497 GO:0046634 1 26

C0027497 GO:0050681 1 26

C0027497 GO:0046631 4 26

C0027497 GO:0050926 2 26

C0027497 GO:0055072 1 26

C0027497 GO:0050920 1 26

C0027497 GO:0050921 1 26

C0027497 GO:0030345 1 26

C0027497 GO:0051893 1 26

C0027497 GO:0008375 1 26

C0027497 GO:0051897 1 26

C0027497 GO:0051896 1 26

C0027497 GO:0007519 2 26

C0027497 GO:0044419 1 26

C0027497 GO:0043325 1 26

C0027497 GO:0010896 2 26

C0027497 GO:0010898 2 26

C0027497 GO:0045839 3 26

C0027497 GO:0010875 3 26

C0027497 GO:0032393 2 26

C0027497 GO:0032934 2 26

C0027497 GO:0014020 1 26

C0027497 GO:0032781 1 26

C0027497 GO:0048634 1 26

C0027497 GO:0030166 1 26

C0027497 GO:0030048 1 26

C0027497 GO:0010975 3 26

C0027497 GO:0030041 1 26

C0027497 GO:0008585 1 26

C0027497 GO:0030514 1 26

C0027497 GO:0030512 1 26

C0027497 GO:0010874 2 26

C0027497 GO:0030510 1 26

C0027497 GO:0043648 1 26

C0027497 GO:0007270 1 26

C0027497 GO:0016615 1 26

C0027497 GO:0048871 1 26

C0027497 GO:0006044 1 26

C0027497 GO:0045429 1 26

C0027497 GO:0051353 3 26

C0027497 GO:0048500 2 26

C0027497 GO:0019439 1 26

C0027497 GO:0043030 1 26

C0027497 GO:0032731 1 26

C0027497 GO:0043034 2 26

C0027497 GO:0030194 1 26

C0027497 GO:0005089 1 26

C0027497 GO:0005088 1 26

C0027497 GO:0007632 1 26

C0027497 GO:0032642 1 26

C0027497 GO:0032648 1 26

C0027497 GO:0005086 1 26

C0027497 GO:0019934 1 26

C0027497 GO:0051453 1 26

C0027497 GO:0042745 1 26

C0027497 GO:0034103 1 26

C0027497 GO:0001843 1 26

C0027497 GO:0031498 1 26

C0027497 GO:0048488 4 26

C0027497 GO:0007062 3 26

C0027497 GO:0002286 1 26

C0027497 GO:0002285 1 26

C0027497 GO:0004428 2 26

C0027497 GO:0051457 1 26

C0027497 GO:0030183 1 26

C0027497 GO:0050700 2 26

C0027497 GO:0015149 1 26

C0027497 GO:0002366 2 26

C0027497 GO:0001841 1 26

C0027497 GO:0006688 1 26

C0027497 GO:0015145 1 26

C0027497 GO:0071174 3 26

C0027497 GO:0046545 2 26

C0027497 GO:0071173 3 26

C0027497 GO:0030890 1 26

C0027497 GO:0033764 1 26

C0027497 GO:0008013 2 26

C0027497 GO:0008154 1 26

C0027497 GO:0043393 1 26

C0027497 GO:0050690 1 26

C0027497 GO:0048255 2 26

C0027497 GO:0043560 1 26

C0027497 GO:0043254 1 26

C0027497 GO:0042102 1 26

C0027497 GO:0045165 2 26

C0027497 GO:0050885 1 26

C0027497 GO:0042734 1 26

C0027497 GO:0002449 1 26

C0027497 GO:0030863 1 26

C0027497 GO:0031645 1 26

C0027497 GO:0002715 1 26

C0027497 GO:0030864 1 26

C0027497 GO:0002218 1 26

C0027497 GO:0016461 1 26

C0027497 GO:0045730 2 26

C0027497 GO:0045737 1 26

C0027497 GO:0030195 1 26

C0027497 GO:0015662 1 26

C0027497 GO:0015665 1 26

C0027497 GO:0046718 2 26

C0027497 GO:0032376 3 26

C0027497 GO:0002819 1 26

C0027497 GO:0035148 1 26

C0027497 GO:0006941 2 26

C0027497 GO:0005901 1 26

C0027497 GO:0005902 1 26

C0027497 GO:0005903 1 26

C0027497 GO:0048029 1 26

C0027497 GO:0052126 2 26

C0027497 GO:0033280 3 26

C0027497 GO:0043087 1 26

C0027497 GO:0009124 2 26

C0027497 GO:0006342 1 26

C0027497 GO:0000041 1 26

C0027497 GO:0046782 1 26

C0027497 GO:0046467 1 26

C0027497 GO:0015296 1 26

C0027497 GO:0002224 1 26

C0027497 GO:0015807 3 26

C0027497 GO:0031994 1 26

C0027497 GO:0001894 2 26

C0027497 GO:0001893 2 26

C0027497 GO:0051092 1 26

C0027497 GO:0006081 1 26

C0027497 GO:0043603 1 26

C0027497 GO:0045862 1 26

C0027497 GO:0043370 1 26

C0027497 GO:0045861 2 26

C0027497 GO:0016459 1 26

C0027497 GO:0000185 1 26

C0027497 GO:0046326 1 26

C0027497 GO:0052192 2 26

C0027497 GO:0010948 1 26

C0027497 GO:0032862 1 26

C0027497 GO:0046112 1 26

C0027497 GO:0045682 1 26

C0027497 GO:0030169 2 26

C0027497 GO:0003707 1 26

C0027497 GO:0030641 1 26

C0027497 GO:0045684 1 26

C0027497 GO:0050661 1 26

C0027497 GO:0050660 1 26

C0027497 GO:0008443 1 26

C0027497 GO:0030520 1 26

C0027497 GO:0042516 1 26

C0027497 GO:0050927 2 26

C0027497 GO:0070412 2 26

C0027497 GO:0042749 1 26

C0027497 GO:0070410 4 26

C0027497 GO:0010741 1 26

C0027497 GO:0005697 1 26

C0027497 GO:0034451 1 26

C0027497 GO:0048662 1 26

C0027497 GO:0048660 1 26

C0027497 GO:0048741 1 26

C0027497 GO:0042104 1 26

C0027497 GO:0048742 1 26

C0027497 GO:0042100 1 26

C0027497 GO:0033483 1 26

C0027497 GO:0042974 1 26

C0027497 GO:0005884 1 26

C0027497 GO:0031214 2 26

C0027497 GO:0002440 2 26

C0027497 GO:0005977 1 26

C0027497 GO:0007157 1 26

C0027497 GO:0051004 1 26

C0027497 GO:0002764 1 26

C0027497 GO:0004806 1 26

C0027497 GO:0034637 1 26

C0027497 GO:0002250 1 26

C0027497 GO:0045646 1 26

C0027497 GO:0001937 1 26

C0027497 GO:0015036 1 26

C0042963 GO:0051043 2 24

C0042963 GO:0051044 1 24

C0042963 GO:0030228 2 24

C0042963 GO:0046887 1 24

C0042963 GO:0001503 1 24

C0042963 GO:0002703 1 24

C0042963 GO:0002702 2 24

C0042963 GO:0002700 1 24

C0042963 GO:0048260 1 24

C0042963 GO:0016864 1 24

C0042963 GO:0016863 2 24

C0042963 GO:0016862 1 24

C0042963 GO:0016860 2 24

C0042963 GO:0006306 1 24

C0042963 GO:0006305 1 24

C0042963 GO:0006304 2 24

C0042963 GO:0000002 2 24

C0042963 GO:0043206 1 24

C0042963 GO:0018212 2 24

C0042963 GO:0018210 2 24

C0042963 GO:0031513 1 24

C0042963 GO:0004715 1 24

C0042963 GO:0004712 1 24

C0042963 GO:0046504 1 24

C0042963 GO:0060541 1 24

C0042963 GO:0045851 1 24

C0042963 GO:0002824 1 24

C0042963 GO:0002822 2 24

C0042963 GO:0005788 1 24

C0042963 GO:0030291 1 24

C0042963 GO:0030295 1 24

C0042963 GO:0050770 1 24

C0042963 GO:0034284 1 24

C0042963 GO:0016812 1 24

C0042963 GO:0016814 1 24

C0042963 GO:0051784 3 24

C0042963 GO:0004622 1 24

C0042963 GO:0021537 3 24

C0042963 GO:0060263 2 24

C0042963 GO:0032411 1 24

C0042963 GO:0006458 1 24

C0042963 GO:0009898 1 24

C0042963 GO:0010906 1 24

C0042963 GO:0046658 1 24

C0042963 GO:0043025 1 24

C0042963 GO:0000578 1 24

C0042963 GO:0051224 1 24

C0042963 GO:0030071 1 24

C0042963 GO:0003205 1 24

C0042963 GO:0003206 1 24

C0042963 GO:0043028 1 24

C0042963 GO:0046850 1 24

C0042963 GO:0006112 1 24

C0042963 GO:0006110 2 24

C0042963 GO:0045912 2 24

C0042963 GO:0051879 1 24

C0042963 GO:0043027 1 24

C0042963 GO:0048705 1 24

C0042963 GO:0019882 1 24

C0042963 GO:0019883 1 24

C0042963 GO:0007622 1 24

C0042963 GO:0005071 2 24

C0042963 GO:0048709 1 24

C0042963 GO:0005138 2 24

C0042963 GO:0060606 2 24

C0042963 GO:0042439 1 24

C0042963 GO:0046870 1 24

C0042963 GO:0043205 1 24

C0042963 GO:0035303 1 24

C0042963 GO:0004437 1 24

C0042963 GO:0030675 2 24

C0042963 GO:0050715 1 24

C0042963 GO:0050714 1 24

C0042963 GO:0050650 1 24

C0042963 GO:0042605 1 24

C0042963 GO:0050654 1 24

C0042963 GO:0070567 1 24

C0042963 GO:0006693 1 24

C0042963 GO:0006692 1 24

C0042963 GO:0015179 1 24

C0042963 GO:0006750 1 24

C0042963 GO:0015175 1 24

C0042963 GO:0006752 1 24

C0042963 GO:0048638 1 24

C0042963 GO:0050810 1 24

C0042963 GO:0033613 1 24

C0042963 GO:0008028 1 24

C0042963 GO:0048145 1 24

C0042963 GO:0046579 1 24

C0042963 GO:0043256 1 24

C0042963 GO:0031228 1 24

C0042963 GO:0042177 2 24

C0042963 GO:0042632 1 24

C0042963 GO:0004693 1 24

C0042963 GO:0005871 1 24

C0042963 GO:0051457 1 24

C0042963 GO:0031225 1 24

C0042963 GO:0007127 1 24

C0042963 GO:0045078 1 24

C0042963 GO:0043489 2 24

C0042963 GO:0045072 1 24

C0042963 GO:0030705 2 24

C0042963 GO:0016459 1 24

C0042963 GO:0045621 1 24

C0042963 GO:0005506 1 24

C0042963 GO:0045622 1 24

C0042963 GO:0008286 2 24

C0042963 GO:0006879 1 24

C0042963 GO:0008329 1 24

C0042963 GO:0015929 1 24

C0042963 GO:0045211 1 24

C0042963 GO:0001654 2 24

C0042963 GO:0017048 2 24

C0042963 GO:0001656 1 24

C0042963 GO:0010677 2 24

C0042963 GO:0046889 1 24

C0042963 GO:0060021 4 24

C0042963 GO:0051983 2 24

C0042963 GO:0042269 1 24

C0042963 GO:0004190 2 24

C0042963 GO:0007229 1 24

C0042963 GO:0017046 1 24

C0042963 GO:0018107 1 24

C0042963 GO:0018105 2 24

C0042963 GO:0003995 1 24

C0042963 GO:0030031 1 24

C0042963 GO:0018065 1 24

C0042963 GO:0018108 2 24

C0042963 GO:0060348 1 24

C0042963 GO:0022406 1 24

C0042963 GO:0045884 1 24

C0042963 GO:0008210 1 24

C0042963 GO:0045445 1 24

C0042963 GO:0007272 1 24

C0042963 GO:0010828 1 24

C0042963 GO:0070491 1 24

C0042963 GO:0032355 2 24

C0042963 GO:0001727 1 24

C0042963 GO:0032350 1 24

C0042963 GO:0042354 1 24

C0042963 GO:0032210 1 24

C0042963 GO:0006096 1 24

C0042963 GO:0070665 1 24

C0042963 GO:0070668 1 24

C0042963 GO:0019213 1 24

C0042963 GO:0006700 1 24

C0042963 GO:0021915 2 24

C0042963 GO:0045088 1 24

C0042963 GO:0070120 1 24

C0042963 GO:0048592 1 24

C0042963 GO:0048593 2 24

C0042963 GO:0032856 1 24

C0042963 GO:0001838 2 24

C0042963 GO:0010595 1 24

C0042963 GO:0010594 2 24

C0042963 GO:0005044 1 24

C0042963 GO:0008517 1 24

C0042963 GO:0050995 1 24

C0042963 GO:0050994 1 24

C0042963 GO:0032320 1 24

C0042963 GO:0032321 1 24

C0042963 GO:0055092 1 24

C0042963 GO:0051806 2 24

C0042963 GO:0006413 2 24

C0042963 GO:0019363 1 24

C0042963 GO:0008366 1 24

C0042963 GO:0032148 1 24

C0042963 GO:0005149 1 24

C0042963 GO:0007612 1 24

C0042963 GO:0048771 1 24

C0042963 GO:0007162 1 24

C0042963 GO:0007163 2 24

C0042963 GO:0043524 1 24

C0042963 GO:0043525 1 24

C0042963 GO:0019915 1 24

C0042963 GO:0031343 1 24

C0042963 GO:0045767 2 24

C0042963 GO:0016597 1 24

C0042963 GO:0004364 1 24

C0042963 GO:0004407 1 24

C0042963 GO:0002263 1 24

C0042963 GO:0055117 1 24

C0042963 GO:0045667 1 24

C0042963 GO:0045661 1 24

C0042963 GO:0045669 1 24

C0042963 GO:0010522 1 24

C0042963 GO:0033032 2 24

C0042963 GO:0051057 1 24

C0042963 GO:0016202 1 24

C0042963 GO:0043966 1 24

C0042963 GO:0051059 2 24

C0042963 GO:0046965 1 24

C0042963 GO:0051701 1 24

C0042963 GO:0042992 1 24

C0042963 GO:0019827 1 24

C0042963 GO:0002429 1 24

C0042963 GO:0018209 1 24

C0042963 GO:0030260 2 24

C0042963 GO:0006942 2 24

C0042963 GO:0004709 2 24

C0042963 GO:0004708 1 24

C0042963 GO:0045841 3 24

C0042963 GO:0008252 1 24

C0042963 GO:0050820 1 24

C0042963 GO:0001764 2 24

C0042963 GO:0005929 1 24

C0042963 GO:0048002 1 24

C0042963 GO:0030286 1 24

C0042963 GO:0007530 1 24

C0042963 GO:0002764 1 24

C0042963 GO:0046915 1 24

C0042963 GO:0008088 1 24

C0042963 GO:0004143 1 24

C0042963 GO:0009880 1 24

C0042963 GO:0020037 1 24

C0042963 GO:0032768 1 24

C0042963 GO:0050796 1 24

C0042963 GO:0060070 3 24

C0042963 GO:0030069 1 24

C0042963 GO:0046660 2 24

C0042963 GO:0009953 1 24

C0042963 GO:0009952 1 24

C0042963 GO:0006895 1 24

C0042963 GO:0032364 1 24

C0042963 GO:0006513 1 24

C0042963 GO:0017080 2 24

C0042963 GO:0046823 1 24

C0042963 GO:0006518 1 24

C0042963 GO:0043010 2 24

C0042963 GO:0042834 1 24

C0042963 GO:0002715 1 24

C0042963 GO:0032107 1 24

C0042963 GO:0032104 1 24

C0042963 GO:0019894 1 24

C0042963 GO:0016790 1 24

C0042963 GO:0019956 1 24

C0042963 GO:0009312 1 24

C0042963 GO:0002718 4 24

C0042963 GO:0009895 3 24

C0042963 GO:0007043 1 24

C0042963 GO:0001948 1 24

C0042963 GO:0010563 1 24

C0042963 GO:0050768 1 24

C0042963 GO:0001530 1 24

C0042963 GO:0015166 1 24

C0042963 GO:0006749 1 24

C0042963 GO:0031397 1 24

C0042963 GO:0005355 1 24

C0042963 GO:0042169 3 24

C0042963 GO:0008034 2 24

C0042963 GO:0007131 2 24

C0042963 GO:0008093 1 24

C0042963 GO:0043470 1 24

C0042963 GO:0043471 1 24

C0042963 GO:0002460 2 24

C0042963 GO:0002237 2 24

C0042963 GO:0045619 1 24

C0042963 GO:0034405 1 24

C0042963 GO:0045616 1 24

C0042963 GO:0005518 1 24

C0042963 GO:0016409 1 24

C0042963 GO:0016331 1 24

C0042963 GO:0051384 1 24

C0042963 GO:0030324 1 24

C0042963 GO:0030326 1 24

C0042963 GO:0005041 1 24

C0042963 GO:0016338 2 24

C0042963 GO:0016234 1 24

C0042963 GO:0009108 1 24

C0042963 GO:0034623 1 24

C0042963 GO:0002724 3 24

C0042963 GO:0002720 2 24

C0042963 GO:0045216 1 24

C0042963 GO:0004181 1 24

C0042963 GO:0004675 1 24

C0042963 GO:0016538 1 24

C0042963 GO:0007098 1 24

C0042963 GO:0043588 1 24

C0042963 GO:0045936 1 24

C0042963 GO:0010812 1 24

C0042963 GO:0045089 1 24

C0042963 GO:0050690 1 24

C0042963 GO:0032318 1 24

C0042963 GO:0022410 1 24

C0042963 GO:0035113 1 24

C0042963 GO:0043627 1 24

C0042963 GO:0007266 1 24

C0042963 GO:0035295 1 24

C0042963 GO:0009746 1 24

C0042963 GO:0043190 1 24

C0042963 GO:0005605 2 24

C0042963 GO:0006493 1 24

C0042963 GO:0051881 1 24

C0042963 GO:0043197 1 24

C0042963 GO:0002793 1 24

C0042963 GO:0051923 1 24

C0042963 GO:0003705 2 24

C0042963 GO:0003707 1 24

C0042963 GO:0044409 2 24

C0042963 GO:0060538 2 24

C0042963 GO:0044403 1 24

C0042963 GO:0046697 1 24

C0042963 GO:0005786 2 24

C0042963 GO:0000242 2 24

C0042963 GO:0031970 1 24

C0042963 GO:0032387 1 24

C0042963 GO:0021510 2 24

C0042963 GO:0006477 1 24

C0042963 GO:0030509 1 24

C0042963 GO:0031057 1 24

C0042963 GO:0031058 1 24

C0042963 GO:0015085 2 24

C0042963 GO:0005072 1 24

C0042963 GO:0048864 1 24

C0042963 GO:0007213 1 24

C0042963 GO:0005770 1 24

C0042963 GO:0042562 1 24

C0042963 GO:0070001 2 24

C0042963 GO:0048512 1 24

C0042963 GO:0010721 1 24

C0042963 GO:0019359 1 24

C0042963 GO:0043120 1 24

C0042963 GO:0070228 1 24

C0042963 GO:0032655 1 24

C0042963 GO:0030427 2 24

C0042963 GO:0005159 2 24

C0042963 GO:0005158 1 24

C0042963 GO:0043536 1 24

C0042963 GO:0042752 1 24

C0042963 GO:0045187 1 24

C0042963 GO:0007173 2 24

C0042963 GO:0046631 2 24

C0042963 GO:0048641 1 24

C0042963 GO:0070875 2 24

C0042963 GO:0060395 1 24

C0042963 GO:0016055 2 24

C0042963 GO:0060393 1 24

C0042963 GO:0002377 1 24

C0042963 GO:0001910 1 24

C0042963 GO:0004551 1 24

C0042963 GO:0033558 1 24

C0042963 GO:0003015 1 24

C0042963 GO:0006929 2 24

C0042963 GO:0008374 1 24

C0042963 GO:0000786 2 24

C0042963 GO:0031334 2 24

C0042963 GO:0048246 1 24

C0042963 GO:0009068 2 24

C0042963 GO:0021700 1 24

C0042963 GO:0031577 3 24

C0042963 GO:0031575 1 24

C0042963 GO:0043547 1 24

C0042963 GO:0031670 1 24

C0042963 GO:0043548 2 24

C0042963 GO:0031672 1 24

C0042963 GO:0005391 1 24

C0042963 GO:0005522 1 24

C0042963 GO:0045725 2 24

C0042963 GO:0045727 1 24

C0042963 GO:0030879 1 24

C0042963 GO:0030371 1 24

C0042963 GO:0035250 1 24

C0042963 GO:0008301 1 24

C0042963 GO:0042308 1 24

C0042963 GO:0050819 1 24

C0042963 GO:0042301 1 24

C0042963 GO:0044275 1 24

C0042963 GO:0002757 1 24

C0042963 GO:0008091 2 24

C0042963 GO:0007423 1 24

C0042963 GO:0046906 1 24

C0042963 GO:0016830 1 24

C0042963 GO:0030276 1 24

C0042963 GO:0032770 1 24

C0042963 GO:0010921 1 24

C0042963 GO:0017022 1 24

C0042963 GO:0045580 1 24

C0042963 GO:0045582 1 24

C0042963 GO:0030323 2 24

C0042963 GO:0046677 1 24

C0042963 GO:0042572 1 24

C0042963 GO:0043168 1 24

C0042963 GO:0050885 2 24

C0042963 GO:0032374 1 24

C0042963 GO:0001709 3 24

C0042963 GO:0032371 1 24

C0042963 GO:0032370 4 24

C0042963 GO:0032373 4 24

C0042963 GO:0008308 1 24

C0042963 GO:0003756 1 24

C0042963 GO:0032273 2 24

C0042963 GO:0060562 1 24

C0042963 GO:0017157 1 24

C0042963 GO:0019209 1 24

C0042963 GO:0043535 2 24

C0042963 GO:0031330 2 24

C0042963 GO:0015459 1 24

C0042963 GO:0031333 1 24

C0042963 GO:0000217 1 24

C0042963 GO:0004861 1 24

C0042963 GO:0048385 1 24

C0042963 GO:0005593 1 24

C0042963 GO:0043534 1 24

C0042963 GO:0050679 1 24

C0042963 GO:0001953 1 24

C0042963 GO:0050671 1 24

C0042963 GO:0005024 1 24

C0042963 GO:0051828 2 24

C0042963 GO:0006004 1 24

C0042963 GO:0006000 1 24

C0042963 GO:0005160 1 24

C0042963 GO:0005161 1 24

C0042963 GO:0042113 2 24

C0042963 GO:0043506 3 24

C0042963 GO:0045768 2 24

C0042963 GO:0032984 2 24

C0042963 GO:0043467 1 24

C0042963 GO:0015074 1 24

C0042963 GO:0045766 1 24

C0042963 GO:0002573 1 24

C0042963 GO:0015695 1 24

C0042963 GO:0045604 1 24

C0042963 GO:0050851 1 24

C0042963 GO:0050852 1 24

C0042963 GO:0007584 1 24

C0042963 GO:0034341 1 24

C0042963 GO:0009112 2 24

C0042963 GO:0042026 1 24

C0042963 GO:0003785 2 24

C0042963 GO:0051646 1 24

C0042963 GO:0034185 1 24

C0042963 GO:0016877 1 24

C0042963 GO:0045123 1 24

C0042963 GO:0045124 1 24

C0042963 GO:0030900 2 24

C0042963 GO:0007094 3 24

C0042963 GO:0032376 4 24

C0042963 GO:0035065 1 24

C0042963 GO:0046637 1 24

C0042963 GO:0046634 1 24

C0042963 GO:0050681 1 24

C0042963 GO:0030426 1 24

C0042963 GO:0008585 1 24

C0042963 GO:0050926 2 24

C0042963 GO:0050927 2 24

C0042963 GO:0051893 1 24

C0042963 GO:0008375 1 24

C0042963 GO:0051897 1 24

C0042963 GO:0051896 1 24

C0042963 GO:0007519 2 24

C0042963 GO:0044419 1 24

C0042963 GO:0043325 1 24

C0042963 GO:0010896 1 24

C0042963 GO:0010898 1 24

C0042963 GO:0045839 3 24

C0042963 GO:0030510 1 24

C0042963 GO:0032393 1 24

C0042963 GO:0032934 1 24

C0042963 GO:0014020 2 24

C0042963 GO:0031960 1 24

C0042963 GO:0048634 1 24

C0042963 GO:0030048 1 24

C0042963 GO:0010975 1 24

C0042963 GO:0030041 1 24

C0042963 GO:0042345 1 24

C0042963 GO:0030514 1 24

C0042963 GO:0010874 2 24

C0042963 GO:0010875 3 24

C0042963 GO:0043648 1 24

C0042963 GO:0007270 1 24

C0042963 GO:0016615 1 24

C0042963 GO:0006041 1 24

C0042963 GO:0006044 1 24

C0042963 GO:0045429 1 24

C0042963 GO:0051353 2 24

C0042963 GO:0060047 1 24

C0042963 GO:0046849 1 24

C0042963 GO:0048500 2 24

C0042963 GO:0048531 1 24

C0042963 GO:0043034 2 24

C0042963 GO:0030194 1 24

C0042963 GO:0005089 1 24

C0042963 GO:0005088 1 24

C0042963 GO:0007632 1 24

C0042963 GO:0001843 2 24

C0042963 GO:0005086 1 24

C0042963 GO:0019934 1 24

C0042963 GO:0051453 1 24

C0042963 GO:0042745 1 24

C0042963 GO:0034103 1 24

C0042963 GO:0032946 1 24

C0042963 GO:0051452 1 24

C0042963 GO:0048488 3 24

C0042963 GO:0007062 3 24

C0042963 GO:0002286 1 24

C0042963 GO:0002285 1 24

C0042963 GO:0004428 2 24

C0042963 GO:0045646 1 24

C0042963 GO:0030183 1 24

C0042963 GO:0050700 2 24

C0042963 GO:0015149 1 24

C0042963 GO:0002366 1 24

C0042963 GO:0006687 1 24

C0042963 GO:0001841 2 24

C0042963 GO:0006688 1 24

C0042963 GO:0015145 1 24

C0042963 GO:0071174 3 24

C0042963 GO:0046545 2 24

C0042963 GO:0071173 3 24

C0042963 GO:0030890 1 24

C0042963 GO:0008013 2 24

C0042963 GO:0008154 1 24

C0042963 GO:0043393 1 24

C0042963 GO:0048255 2 24

C0042963 GO:0014003 1 24

C0042963 GO:0043560 1 24

C0042963 GO:0043254 1 24

C0042963 GO:0045165 2 24

C0042963 GO:0007250 2 24

C0042963 GO:0042734 1 24

C0042963 GO:0030863 1 24

C0042963 GO:0031645 2 24

C0042963 GO:0030864 1 24

C0042963 GO:0033261 2 24

C0042963 GO:0016461 1 24

C0042963 GO:0045730 1 24

C0042963 GO:0045737 1 24

C0042963 GO:0030195 1 24

C0042963 GO:0015665 1 24

C0042963 GO:0046718 2 24

C0042963 GO:0050808 1 24

C0042963 GO:0002819 1 24

C0042963 GO:0035148 2 24

C0042963 GO:0006941 2 24

C0042963 GO:0005901 1 24

C0042963 GO:0005903 1 24

C0042963 GO:0048029 1 24

C0042963 GO:0052126 2 24

C0042963 GO:0033280 3 24

C0042963 GO:0043087 2 24

C0042963 GO:0009124 2 24

C0042963 GO:0006342 1 24

C0042963 GO:0046782 1 24

C0042963 GO:0015296 2 24

C0042963 GO:0015807 3 24

C0042963 GO:0031994 1 24

C0042963 GO:0001894 2 24

C0042963 GO:0001893 1 24

C0042963 GO:0006081 1 24

C0042963 GO:0043603 1 24

C0042963 GO:0045862 1 24

C0042963 GO:0043370 1 24

C0042963 GO:0045861 1 24

C0042963 GO:0019200 2 24

C0042963 GO:0046326 1 24

C0042963 GO:0052192 2 24

C0042963 GO:0010948 1 24

C0042963 GO:0032862 1 24

C0042963 GO:0046112 1 24

C0042963 GO:0045682 1 24

C0042963 GO:0030169 1 24

C0042963 GO:0009749 1 24

C0042963 GO:0030641 1 24

C0042963 GO:0045684 1 24

C0042963 GO:0050660 1 24

C0042963 GO:0008443 1 24

C0042963 GO:0030520 1 24

C0042963 GO:0070412 2 24

C0042963 GO:0042749 1 24

C0042963 GO:0070410 4 24

C0042963 GO:0010741 1 24

C0042963 GO:0005697 1 24

C0042963 GO:0034451 1 24

C0042963 GO:0048662 1 24

C0042963 GO:0048660 1 24

C0042963 GO:0009247 1 24

C0042963 GO:0048742 1 24

C0042963 GO:0033483 1 24

C0042963 GO:0042974 1 24

C0042963 GO:0007159 1 24

C0042963 GO:0031214 2 24

C0042963 GO:0002440 2 24

C0042963 GO:0007157 1 24

C0042963 GO:0004806 1 24

C0042963 GO:0034637 1 24

C0042963 GO:0019047 1 24

C0042963 GO:0002250 2 24

C0042963 GO:0001937 1 24

C0042963 GO:0015036 1 24

C0027769 GO:0051043 1 9

C0027769 GO:0051044 1 9

C0027769 GO:0030228 1 9

C0027769 GO:0043954 1 9

C0027769 GO:0031970 1 9

C0027769 GO:0046887 1 9

C0027769 GO:0008585 1 9

C0027769 GO:0050926 1 9

C0027769 GO:0050927 1 9

C0027769 GO:0002703 1 9

C0027769 GO:0003015 1 9

C0027769 GO:0019915 1 9

C0027769 GO:0051893 1 9

C0027769 GO:0031058 1 9

C0027769 GO:0046631 1 9

C0027769 GO:0016864 1 9

C0027769 GO:0031345 1 9

C0027769 GO:0016862 1 9

C0027769 GO:0016860 2 9

C0027769 GO:0015085 1 9

C0027769 GO:0043205 1 9

C0027769 GO:0015082 1 9

C0027769 GO:0002263 1 9

C0027769 GO:0007519 1 9

C0027769 GO:0070001 1 9

C0027769 GO:0006266 1 9

C0027769 GO:0010894 1 9

C0027769 GO:0010896 1 9

C0027769 GO:0010898 1 9

C0027769 GO:0046504 1 9

C0027769 GO:0010875 1 9

C0027769 GO:0043120 1 9

C0027769 GO:0060541 1 9

C0027769 GO:0070228 1 9

C0027769 GO:0004029 1 9

C0027769 GO:0030216 1 9

C0027769 GO:0005159 1 9

C0027769 GO:0005158 1 9

C0027769 GO:0002822 1 9

C0027769 GO:0046030 1 9

C0027769 GO:0007176 1 9

C0027769 GO:0043535 1 9

C0027769 GO:0019905 1 9

C0027769 GO:0006477 1 9

C0027769 GO:0048641 1 9

C0027769 GO:0043536 1 9

C0027769 GO:0010874 1 9

C0027769 GO:0030169 1 9

C0027769 GO:0014020 1 9

C0027769 GO:0031343 1 9

C0027769 GO:0034284 1 9

C0027769 GO:0016812 1 9

C0027769 GO:0005254 1 9

C0027769 GO:0003785 1 9

C0027769 GO:0002274 1 9

C0027769 GO:0051219 1 9

C0027769 GO:0016814 1 9

C0027769 GO:0051353 1 9

C0027769 GO:0001910 2 9

C0027769 GO:0003995 1 9

C0027769 GO:0005388 1 9

C0027769 GO:0045494 1 9

C0027769 GO:0030194 1 9

C0027769 GO:0021537 2 9

C0027769 GO:0009895 1 9

C0027769 GO:0007635 1 9

C0027769 GO:0051103 1 9

C0027769 GO:0019934 1 9

C0027769 GO:0050820 1 9

C0027769 GO:0046326 1 9

C0027769 GO:0048002 1 9

C0027769 GO:0048246 1 9

C0027769 GO:0009068 1 9

C0027769 GO:0021700 1 9

C0027769 GO:0016863 2 9

C0027769 GO:0007530 1 9

C0027769 GO:0002286 1 9

C0027769 GO:0046915 1 9

C0027769 GO:0016801 1 9

C0027769 GO:0006885 1 9

C0027769 GO:0000149 1 9

C0027769 GO:0031672 1 9

C0027769 GO:0050700 1 9

C0027769 GO:0008088 1 9

C0027769 GO:0002366 1 9

C0027769 GO:0001841 1 9

C0027769 GO:0045912 2 9

C0027769 GO:0001843 1 9

C0027769 GO:0045727 1 9

C0027769 GO:0051879 1 9

C0027769 GO:0005788 1 9

C0027769 GO:0043168 1 9

C0027769 GO:0019882 1 9

C0027769 GO:0019883 1 9

C0027769 GO:0046545 1 9

C0027769 GO:0033483 1 9

C0027769 GO:0005138 1 9

C0027769 GO:0060606 1 9

C0027769 GO:0050810 1 9

C0027769 GO:0048255 1 9

C0027769 GO:0030069 1 9

C0027769 GO:0034341 1 9

C0027769 GO:0046660 1 9

C0027769 GO:0008308 1 9

C0027769 GO:0042301 1 9

C0027769 GO:0004437 1 9

C0027769 GO:0005451 1 9

C0027769 GO:0007250 1 9

C0027769 GO:0050715 1 9

C0027769 GO:0050714 1 9

C0027769 GO:0031645 2 9

C0027769 GO:0032364 1 9

C0027769 GO:0016830 1 9

C0027769 GO:0070567 1 9

C0027769 GO:0045730 1 9

C0027769 GO:0005247 1 9

C0027769 GO:0015385 1 9

C0027769 GO:0042834 1 9

C0027769 GO:0002715 1 9

C0027769 GO:0048638 1 9

C0027769 GO:0050808 1 9

C0027769 GO:0048145 1 9

C0027769 GO:0035148 1 9

C0027769 GO:0002440 1 9

C0027769 GO:0045429 1 9

C0027769 GO:0005903 1 9

C0027769 GO:0046677 1 9

C0027769 GO:0032373 2 9

C0027769 GO:0051453 1 9

C0027769 GO:0051457 1 9

C0027769 GO:0009124 1 9

C0027769 GO:0001948 1 9

C0027769 GO:0045078 1 9

C0027769 GO:0043489 1 9

C0027769 GO:0032376 2 9

C0027769 GO:0032370 2 9

C0027769 GO:0045072 1 9

C0027769 GO:0001530 1 9

C0027769 GO:0046782 1 9

C0027769 GO:0004445 1 9

C0027769 GO:0002224 1 9

C0027769 GO:0031397 1 9

C0027769 GO:0005506 1 9

C0027769 GO:0017157 1 9

C0027769 GO:0001838 1 9

C0027769 GO:0008034 1 9

C0027769 GO:0015923 1 9

C0027769 GO:0005518 1 9

C0027769 GO:0031330 1 9

C0027769 GO:0000217 1 9

C0027769 GO:0015929 1 9

C0027769 GO:0030004 1 9

C0027769 GO:0015807 1 9

C0027769 GO:0001654 1 9

C0027769 GO:0006942 1 9

C0027769 GO:0010677 2 9

C0027769 GO:0005593 1 9

C0027769 GO:0046889 1 9

C0027769 GO:0060021 1 9

C0027769 GO:0004693 1 9

C0027769 GO:0042269 1 9

C0027769 GO:0050771 1 9

C0027769 GO:0008093 1 9

C0027769 GO:0060047 1 9

C0027769 GO:0005024 1 9

C0027769 GO:0004190 1 9

C0027769 GO:0006004 1 9

C0027769 GO:0045861 1 9

C0027769 GO:0017144 1 9

C0027769 GO:0007229 1 9

C0027769 GO:0019200 1 9

C0027769 GO:0019047 1 9

C0027769 GO:0030324 1 9

C0027769 GO:0042177 1 9

C0027769 GO:0051923 1 9

C0027769 GO:0005929 1 9

C0027769 GO:0002285 1 9

C0027769 GO:0017048 1 9

C0027769 GO:0042058 1 9

C0027769 GO:0003756 1 9

C0027769 GO:0046112 1 9

C0027769 GO:0045682 1 9

C0027769 GO:0010828 1 9

C0027769 GO:0030641 1 9

C0027769 GO:0045165 1 9

C0027769 GO:0008329 1 9

C0027769 GO:0015074 1 9

C0027769 GO:0070555 1 9

C0027769 GO:0016645 1 9

C0027769 GO:0004675 1 9

C0027769 GO:0015695 1 9

C0027769 GO:0010469 1 9

C0027769 GO:0010741 1 9

C0027769 GO:0051897 1 9

C0027769 GO:0048531 1 9

C0027769 GO:0048662 1 9

C0027769 GO:0048660 1 9

C0027769 GO:0045939 1 9

C0027769 GO:0031334 1 9

C0027769 GO:0048742 1 9

C0027769 GO:0042102 2 9

C0027769 GO:0009112 1 9

C0027769 GO:0007159 1 9

C0027769 GO:0055067 1 9

C0027769 GO:0031214 1 9

C0027769 GO:0051646 1 9

C0027769 GO:0007266 1 9

C0027769 GO:0006752 1 9

C0027769 GO:0009746 1 9

C0027769 GO:0010595 1 9

C0027769 GO:0010594 1 9

C0027769 GO:0016338 1 9

C0027769 GO:0045211 1 9

C0027769 GO:0002793 1 9

C0027769 GO:0050995 1 9

C0027769 GO:0050994 1 9

C0027769 GO:0009749 1 9

C0027769 GO:0030900 1 9

C0027769 GO:0060538 1 9

C0027769 GO:0006413 1 9

C0027769 GO:0001937 1 9

C0018681 GO:0051043 1 25

C0018681 GO:0006909 2 25

C0018681 GO:0030228 1 25

C0018681 GO:0030122 1 25

C0018681 GO:0001503 1 25

C0018681 GO:0002703 3 25

C0018681 GO:0001505 1 25

C0018681 GO:0042036 3 25

C0018681 GO:0009247 1 25

C0018681 GO:0051044 2 25

C0018681 GO:0048261 1 25

C0018681 GO:0016863 4 25

C0018681 GO:0016860 3 25

C0018681 GO:0015149 2 25

C0018681 GO:0006305 2 25

C0018681 GO:0006304 2 25

C0018681 GO:0043205 1 25

C0018681 GO:0019992 2 25

C0018681 GO:0018212 1 25

C0018681 GO:0018210 2 25

C0018681 GO:0031513 1 25

C0018681 GO:0004716 1 25

C0018681 GO:0004715 1 25

C0018681 GO:0008656 1 25

C0018681 GO:0016101 2 25

C0018681 GO:0009190 1 25

C0018681 GO:0006687 1 25

C0018681 GO:0060541 2 25

C0018681 GO:0006284 1 25

C0018681 GO:0045851 1 25

C0018681 GO:0004029 1 25

C0018681 GO:0005583 2 25

C0018681 GO:0006688 1 25

C0018681 GO:0035176 1 25

C0018681 GO:0034311 1 25

C0018681 GO:0002822 3 25

C0018681 GO:0019229 1 25

C0018681 GO:0007281 2 25

C0018681 GO:0046330 1 25

C0018681 GO:0008360 1 25

C0018681 GO:0005788 1 25

C0018681 GO:0030291 2 25

C0018681 GO:0050839 2 25

C0018681 GO:0030295 2 25

C0018681 GO:0042088 1 25

C0018681 GO:0032365 1 25

C0018681 GO:0016812 1 25

C0018681 GO:0048477 1 25

C0018681 GO:0006953 1 25

C0018681 GO:0016811 1 25

C0018681 GO:0070633 2 25

C0018681 GO:0016814 1 25

C0018681 GO:0010887 1 25

C0018681 GO:0035097 1 25

C0018681 GO:0030198 1 25

C0018681 GO:0004622 1 25

C0018681 GO:0002702 1 25

C0018681 GO:0021537 1 25

C0018681 GO:0060263 2 25

C0018681 GO:0032412 1 25

C0018681 GO:0017002 1 25

C0018681 GO:0001953 1 25

C0018681 GO:0009898 1 25

C0018681 GO:0000302 2 25

C0018681 GO:0010906 2 25

C0018681 GO:0030170 1 25

C0018681 GO:0000578 2 25

C0018681 GO:0045778 2 25

C0018681 GO:0010862 1 25

C0018681 GO:0051224 1 25

C0018681 GO:0030073 1 25

C0018681 GO:0003205 1 25

C0018681 GO:0003206 1 25

C0018681 GO:0042551 2 25

C0018681 GO:0006885 1 25

C0018681 GO:0050866 1 25

C0018681 GO:0003208 1 25

C0018681 GO:0008643 1 25

C0018681 GO:0043028 3 25

C0018681 GO:0046850 2 25

C0018681 GO:0006112 1 25

C0018681 GO:0006110 3 25

C0018681 GO:0045912 2 25

C0018681 GO:0051879 1 25

C0018681 GO:0043027 1 25

C0018681 GO:0048705 2 25

C0018681 GO:0019882 3 25

C0018681 GO:0019883 1 25

C0018681 GO:0007623 1 25

C0018681 GO:0006306 2 25

C0018681 GO:0009306 1 25

C0018681 GO:0031256 1 25

C0018681 GO:0070167 2 25

C0018681 GO:0006584 1 25

C0018681 GO:0060606 2 25

C0018681 GO:0006303 1 25

C0018681 GO:0035303 1 25

C0018681 GO:0004437 1 25

C0018681 GO:0050716 1 25

C0018681 GO:0050715 2 25

C0018681 GO:0050714 3 25

C0018681 GO:0050650 1 25

C0018681 GO:0050718 1 25

C0018681 GO:0005436 2 25

C0018681 GO:0070567 3 25

C0018681 GO:0006029 1 25

C0018681 GO:0016676 1 25

C0018681 GO:0015179 2 25

C0018681 GO:0019827 2 25

C0018681 GO:0015175 1 25

C0018681 GO:0006026 2 25

C0018681 GO:0006027 1 25

C0018681 GO:0015172 1 25

C0018681 GO:0043392 1 25

C0018681 GO:0048638 1 25

C0018681 GO:0033613 2 25

C0018681 GO:0045923 2 25

C0018681 GO:0048634 1 25

C0018681 GO:0042531 1 25

C0018681 GO:0009410 2 25

C0018681 GO:0019047 2 25

C0018681 GO:0004693 1 25

C0018681 GO:0051452 1 25

C0018681 GO:0005833 1 25

C0018681 GO:0043241 1 25

C0018681 GO:0050431 2 25

C0018681 GO:0007127 1 25

C0018681 GO:0045078 1 25

C0018681 GO:0043489 1 25

C0018681 GO:0045072 1 25

C0018681 GO:0030705 1 25

C0018681 GO:0033238 1 25

C0018681 GO:0033273 2 25

C0018681 GO:0045740 1 25

C0018681 GO:0005504 1 25

C0018681 GO:0005506 1 25

C0018681 GO:0005507 1 25

C0018681 GO:0032677 1 25

C0018681 GO:0045732 1 25

C0018681 GO:0008286 1 25

C0018681 GO:0035239 1 25

C0018681 GO:0008329 1 25

C0018681 GO:0055008 1 25

C0018681 GO:0045445 1 25

C0018681 GO:0045211 1 25

C0018681 GO:0001654 1 25

C0018681 GO:0017048 2 25

C0018681 GO:0045216 1 25

C0018681 GO:0010677 2 25

C0018681 GO:0033293 1 25

C0018681 GO:0060348 1 25

C0018681 GO:0044253 1 25

C0018681 GO:0034329 1 25

C0018681 GO:0043666 1 25

C0018681 GO:0042593 1 25

C0018681 GO:0032722 1 25

C0018681 GO:0004190 4 25

C0018681 GO:0031645 2 25

C0018681 GO:0001889 1 25

C0018681 GO:0004745 1 25

C0018681 GO:0016525 1 25

C0018681 GO:0017046 1 25

C0018681 GO:0018107 1 25

C0018681 GO:0007416 1 25

C0018681 GO:0018105 1 25

C0018681 GO:0015813 1 25

C0018681 GO:0002720 1 25

C0018681 GO:0015810 2 25

C0018681 GO:0019048 1 25

C0018681 GO:0030035 2 25

C0018681 GO:0018108 2 25

C0018681 GO:0060021 1 25

C0018681 GO:0045884 1 25

C0018681 GO:0008210 1 25

C0018681 GO:0050906 1 25

C0018681 GO:0050905 1 25

C0018681 GO:0007270 3 25

C0018681 GO:0015184 1 25

C0018681 GO:0046209 1 25

C0018681 GO:0010828 1 25

C0018681 GO:0006094 2 25

C0018681 GO:0032355 1 25

C0018681 GO:0001727 1 25

C0018681 GO:0042359 1 25

C0018681 GO:0032350 1 25

C0018681 GO:0042354 1 25

C0018681 GO:0006527 1 25

C0018681 GO:0006525 1 25

C0018681 GO:0006096 2 25

C0018681 GO:0045639 1 25

C0018681 GO:0070668 1 25

C0018681 GO:0070491 1 25

C0018681 GO:0005159 1 25

C0018681 GO:0045736 1 25

C0018681 GO:0019213 1 25

C0018681 GO:0006700 1 25

C0018681 GO:0045089 3 25

C0018681 GO:0045088 1 25

C0018681 GO:0070120 1 25

C0018681 GO:0051291 1 25

C0018681 GO:0048593 2 25

C0018681 GO:0032856 1 25

C0018681 GO:0004969 1 25

C0018681 GO:0005095 1 25

C0018681 GO:0006518 1 25

C0018681 GO:0001838 2 25

C0018681 GO:0010595 1 25

C0018681 GO:0010594 3 25

C0018681 GO:0010596 1 25

C0018681 GO:0005044 1 25

C0018681 GO:0008517 3 25

C0018681 GO:0050995 1 25

C0018681 GO:0050994 1 25

C0018681 GO:0016986 2 25

C0018681 GO:0001837 1 25

C0018681 GO:0032320 1 25

C0018681 GO:0032321 1 25

C0018681 GO:0055092 3 25

C0018681 GO:0051806 1 25

C0018681 GO:0043112 1 25

C0018681 GO:0006413 1 25

C0018681 GO:0007172 1 25

C0018681 GO:0042133 2 25

C0018681 GO:0009593 2 25

C0018681 GO:0005149 1 25

C0018681 GO:0007618 1 25

C0018681 GO:0000086 1 25

C0018681 GO:0007612 2 25

C0018681 GO:0048771 1 25

C0018681 GO:0000080 1 25

C0018681 GO:0007611 1 25

C0018681 GO:0007162 1 25

C0018681 GO:0043523 2 25

C0018681 GO:0043524 2 25

C0018681 GO:0019915 2 25

C0018681 GO:0031343 2 25

C0018681 GO:0045767 3 25

C0018681 GO:0016597 1 25

C0018681 GO:0005548 1 25

C0018681 GO:0042446 1 25

C0018681 GO:0004407 1 25

C0018681 GO:0002263 1 25

C0018681 GO:0045667 2 25

C0018681 GO:0045661 1 25

C0018681 GO:0042562 1 25

C0018681 GO:0045669 2 25

C0018681 GO:0015002 1 25

C0018681 GO:0033032 2 25

C0018681 GO:0051057 1 25

C0018681 GO:0016202 1 25

C0018681 GO:0051119 2 25

C0018681 GO:0030218 5 25

C0018681 GO:0051117 1 25

C0018681 GO:0051059 1 25

C0018681 GO:0051058 1 25

C0018681 GO:0001516 1 25

C0018681 GO:0046965 1 25

C0018681 GO:0004112 1 25

C0018681 GO:0016894 1 25

C0018681 GO:0009074 1 25

C0018681 GO:0051702 1 25

C0018681 GO:0042994 1 25

C0018681 GO:0042992 3 25

C0018681 GO:0009072 1 25

C0018681 GO:0014020 2 25

C0018681 GO:0002429 1 25

C0018681 GO:0018209 1 25

C0018681 GO:0045109 1 25

C0018681 GO:0042401 1 25

C0018681 GO:0046530 1 25

C0018681 GO:0004707 1 25

C0018681 GO:0032967 1 25

C0018681 GO:0016291 2 25

C0018681 GO:0005388 1 25

C0018681 GO:0004709 1 25

C0018681 GO:0005905 1 25

C0018681 GO:0004033 1 25

C0018681 GO:0015269 1 25

C0018681 GO:0044403 1 25

C0018681 GO:0050820 1 25

C0018681 GO:0001764 1 25

C0018681 GO:0005929 1 25

C0018681 GO:0048002 2 25

C0018681 GO:0030286 2 25

C0018681 GO:0034708 1 25

C0018681 GO:0007530 1 25

C0018681 GO:0002761 2 25

C0018681 GO:0051184 1 25

C0018681 GO:0051187 1 25

C0018681 GO:0051181 1 25

C0018681 GO:0002764 1 25

C0018681 GO:0051183 1 25

C0018681 GO:0046915 2 25

C0018681 GO:0016801 1 25

C0018681 GO:0046425 1 25

C0018681 GO:0008088 1 25

C0018681 GO:0001910 1 25

C0018681 GO:0044246 1 25

C0018681 GO:0019894 1 25

C0018681 GO:0016755 1 25

C0018681 GO:0004143 3 25

C0018681 GO:0007185 1 25

C0018681 GO:0030148 1 25

C0018681 GO:0001948 1 25

C0018681 GO:0031941 1 25

C0018681 GO:0030235 1 25

C0018681 GO:0009880 2 25

C0018681 GO:0020037 3 25

C0018681 GO:0032768 1 25

C0018681 GO:0006690 1 25

C0018681 GO:0017015 1 25

C0018681 GO:0050796 1 25

C0018681 GO:0050795 1 25

C0018681 GO:0060070 1 25

C0018681 GO:0007569 1 25

C0018681 GO:0003231 1 25

C0018681 GO:0030069 2 25

C0018681 GO:0046660 1 25

C0018681 GO:0009953 1 25

C0018681 GO:0009952 1 25

C0018681 GO:0042542 2 25

C0018681 GO:0034382 1 25

C0018681 GO:0022898 1 25

C0018681 GO:0010714 1 25

C0018681 GO:0010712 1 25

C0018681 GO:0002695 1 25

C0018681 GO:0043154 2 25

C0018681 GO:0017080 1 25

C0018681 GO:0046823 3 25

C0018681 GO:0003746 2 25

C0018681 GO:0010888 1 25

C0018681 GO:0043010 1 25

C0018681 GO:0042834 1 25

C0018681 GO:0046580 1 25

C0018681 GO:0006633 1 25

C0018681 GO:0006739 1 25

C0018681 GO:0006637 1 25

C0018681 GO:0016790 1 25

C0018681 GO:0019956 1 25

C0018681 GO:0009311 1 25

C0018681 GO:0070279 1 25

C0018681 GO:0002718 2 25

C0018681 GO:0009895 2 25

C0018681 GO:0007043 1 25

C0018681 GO:0032963 1 25

C0018681 GO:0030203 1 25

C0018681 GO:0043409 1 25

C0018681 GO:0050766 1 25

C0018681 GO:0050764 1 25

C0018681 GO:0051701 1 25

C0018681 GO:0030201 1 25

C0018681 GO:0016709 2 25

C0018681 GO:0010563 1 25

C0018681 GO:0050769 1 25

C0018681 GO:0005035 1 25

C0018681 GO:0032409 2 25

C0018681 GO:0005031 1 25

C0018681 GO:0002020 1 25

C0018681 GO:0048568 1 25

C0018681 GO:0031397 1 25

C0018681 GO:0005355 2 25

C0018681 GO:0042509 1 25

C0018681 GO:0042169 1 25

C0018681 GO:0070303 1 25

C0018681 GO:0005217 1 25

C0018681 GO:0002706 1 25

C0018681 GO:0070304 2 25

C0018681 GO:0005868 2 25

C0018681 GO:0007131 1 25

C0018681 GO:0032587 1 25

C0018681 GO:0007032 1 25

C0018681 GO:0010869 1 25

C0018681 GO:0043470 3 25

C0018681 GO:0043471 3 25

C0018681 GO:0002460 1 25

C0018681 GO:0030175 1 25

C0018681 GO:0002237 4 25

C0018681 GO:0045619 2 25

C0018681 GO:0034405 2 25

C0018681 GO:0045616 1 25

C0018681 GO:0005518 1 25

C0018681 GO:0016409 1 25

C0018681 GO:0009083 1 25

C0018681 GO:0016331 2 25

C0018681 GO:0030323 2 25

C0018681 GO:0030324 2 25

C0018681 GO:0050868 2 25

C0018681 GO:0000096 1 25

C0018681 GO:0055010 1 25

C0018681 GO:0016338 2 25

C0018681 GO:0015485 1 25

C0018681 GO:0019842 1 25

C0018681 GO:0051147 3 25

C0018681 GO:0034235 1 25

C0018681 GO:0051149 1 25

C0018681 GO:0002724 1 25

C0018681 GO:0050996 1 25

C0018681 GO:0001523 2 25

C0018681 GO:0016846 1 25

C0018681 GO:0031532 1 25

C0018681 GO:0004181 1 25

C0018681 GO:0004675 2 25

C0018681 GO:0016538 2 25

C0018681 GO:0042787 1 25

C0018681 GO:0030934 2 25

C0018681 GO:0060415 1 25

C0018681 GO:0043588 2 25

C0018681 GO:0045931 1 25

C0018681 GO:0045930 1 25

C0018681 GO:0045936 1 25

C0018681 GO:0010812 1 25

C0018681 GO:0050690 2 25

C0018681 GO:0046627 1 25

C0018681 GO:0055062 1 25

C0018681 GO:0045471 1 25

C0018681 GO:0055061 1 25

C0018681 GO:0016505 1 25

C0018681 GO:0043627 1 25

C0018681 GO:0016909 2 25

C0018681 GO:0035295 2 25

C0018681 GO:0043624 1 25

C0018681 GO:0043190 1 25

C0018681 GO:0005605 1 25

C0018681 GO:0042345 1 25

C0018681 GO:0032205 1 25

C0018681 GO:0002793 1 25

C0018681 GO:0051923 1 25

C0018681 GO:0003705 1 25

C0018681 GO:0055081 1 25

C0018681 GO:0044236 1 25

C0018681 GO:0055072 2 25

C0018681 GO:0044409 1 25

C0018681 GO:0060538 3 25

C0018681 GO:0010832 1 25

C0018681 GO:0046697 2 25

C0018681 GO:0006775 2 25

C0018681 GO:0005782 1 25

C0018681 GO:0032925 1 25

C0018681 GO:0008374 1 25

C0018681 GO:0060249 2 25

C0018681 GO:0031970 1 25

C0018681 GO:0032387 2 25

C0018681 GO:0021510 1 25

C0018681 GO:0006477 1 25

C0018681 GO:0006476 1 25

C0018681 GO:0015645 1 25

C0018681 GO:0018958 1 25

C0018681 GO:0031058 1 25

C0018681 GO:0030510 1 25

C0018681 GO:0045454 1 25

C0018681 GO:0032652 1 25

C0018681 GO:0015085 2 25

C0018681 GO:0048864 2 25

C0018681 GO:0015082 1 25

C0018681 GO:0007217 1 25

C0018681 GO:0055085 1 25

C0018681 GO:0070001 4 25

C0018681 GO:0043124 2 25

C0018681 GO:0046870 1 25

C0018681 GO:0043120 2 25

C0018681 GO:0006171 1 25

C0018681 GO:0070228 3 25

C0018681 GO:0035272 1 25

C0018681 GO:0032655 2 25

C0018681 GO:0000099 1 25

C0018681 GO:0005154 1 25

C0018681 GO:0032153 1 25

C0018681 GO:0005158 2 25

C0018681 GO:0032155 1 25

C0018681 GO:0042129 1 25

C0018681 GO:0007158 1 25

C0018681 GO:0003007 2 25

C0018681 GO:0043536 1 25

C0018681 GO:0043535 3 25

C0018681 GO:0002698 1 25

C0018681 GO:0008585 1 25

C0018681 GO:0043531 1 25

C0018681 GO:0007044 1 25

C0018681 GO:0007598 1 25

C0018681 GO:0030275 1 25

C0018681 GO:0008630 1 25

C0018681 GO:0048732 1 25

C0018681 GO:0008633 1 25

C0018681 GO:0043025 1 25

C0018681 GO:0043178 1 25

C0018681 GO:0060393 2 25

C0018681 GO:0002377 2 25

C0018681 GO:0007163 2 25

C0018681 GO:0004550 1 25

C0018681 GO:0004551 2 25

C0018681 GO:0033558 1 25

C0018681 GO:0030206 1 25

C0018681 GO:0030204 1 25

C0018681 GO:0022600 1 25

C0018681 GO:0046006 1 25

C0018681 GO:0030345 1 25

C0018681 GO:0021915 1 25

C0018681 GO:0006929 3 25

C0018681 GO:0009062 1 25

C0018681 GO:0070169 2 25

C0018681 GO:0000786 1 25

C0018681 GO:0050808 1 25

C0018681 GO:0070207 1 25

C0018681 GO:0048246 1 25

C0018681 GO:0021700 1 25

C0018681 GO:0035004 1 25

C0018681 GO:0042625 2 25

C0018681 GO:0045058 1 25

C0018681 GO:0042627 1 25

C0018681 GO:0043547 1 25

C0018681 GO:0090100 1 25

C0018681 GO:0090101 1 25

C0018681 GO:0043548 3 25

C0018681 GO:0031672 1 25

C0018681 GO:0031674 2 25

C0018681 GO:0016571 1 25

C0018681 GO:0030183 1 25

C0018681 GO:0005522 2 25

C0018681 GO:0048641 2 25

C0018681 GO:0002200 1 25

C0018681 GO:0030879 1 25

C0018681 GO:0004129 1 25

C0018681 GO:0015909 1 25

C0018681 GO:0016303 1 25

C0018681 GO:0030371 1 25

C0018681 GO:0007422 1 25

C0018681 GO:0005138 2 25

C0018681 GO:0001776 1 25

C0018681 GO:0043900 1 25

C0018681 GO:0050810 2 25

C0018681 GO:0050811 2 25

C0018681 GO:0034713 1 25

C0018681 GO:0042308 3 25

C0018681 GO:0030278 1 25

C0018681 GO:0030279 3 25

C0018681 GO:0050818 1 25

C0018681 GO:0050819 2 25

C0018681 GO:0042301 1 25

C0018681 GO:0044275 3 25

C0018681 GO:0043410 1 25

C0018681 GO:0008091 2 25

C0018681 GO:0051607 1 25

C0018681 GO:0008093 2 25

C0018681 GO:0046906 2 25

C0018681 GO:0006213 1 25

C0018681 GO:0044447 1 25

C0018681 GO:0043046 1 25

C0018681 GO:0006957 1 25

C0018681 GO:0032770 1 25

C0018681 GO:0015280 1 25

C0018681 GO:0007519 3 25

C0018681 GO:0031016 2 25

C0018681 GO:0019200 1 25

C0018681 GO:0017022 1 25

C0018681 GO:0045580 1 25

C0018681 GO:0045429 3 25

C0018681 GO:0045428 1 25

C0018681 GO:0046677 2 25

C0018681 GO:0003229 1 25

C0018681 GO:0002526 1 25

C0018681 GO:0033138 1 25

C0018681 GO:0008543 2 25

C0018681 GO:0030018 1 25

C0018681 GO:0042273 1 25

C0018681 GO:0051324 1 25

C0018681 GO:0045346 1 25

C0018681 GO:0001656 1 25

C0018681 GO:0007250 2 25

C0018681 GO:0042274 1 25

C0018681 GO:0032374 1 25

C0018681 GO:0009116 1 25

C0018681 GO:0001709 2 25

C0018681 GO:0032371 1 25

C0018681 GO:0032370 3 25

C0018681 GO:0032373 3 25

C0018681 GO:0007259 1 25

C0018681 GO:0015377 3 25

C0018681 GO:0060562 2 25

C0018681 GO:0017157 1 25

C0018681 GO:0019209 2 25

C0018681 GO:0030595 1 25

C0018681 GO:0031330 2 25

C0018681 GO:0006720 1 25

C0018681 GO:0015459 1 25

C0018681 GO:0031333 2 25

C0018681 GO:0000217 2 25

C0018681 GO:0004861 2 25

C0018681 GO:0030128 1 25

C0018681 GO:0048385 1 25

C0018681 GO:0005593 4 25

C0018681 GO:0000188 1 25

C0018681 GO:0008484 1 25

C0018681 GO:0050777 1 25

C0018681 GO:0050771 1 25

C0018681 GO:0050679 1 25

C0018681 GO:0015884 1 25

C0018681 GO:0051250 1 25

C0018681 GO:0016675 1 25

C0018681 GO:0044272 2 25

C0018681 GO:0050670 1 25

C0018681 GO:0050671 1 25

C0018681 GO:0005024 2 25

C0018681 GO:0001816 1 25

C0018681 GO:0051828 1 25

C0018681 GO:0031907 1 25

C0018681 GO:0022839 2 25

C0018681 GO:0006007 1 25

C0018681 GO:0050909 1 25

C0018681 GO:0032421 1 25

C0018681 GO:0031575 1 25

C0018681 GO:0010675 1 25

C0018681 GO:0005160 3 25

C0018681 GO:0005161 2 25

C0018681 GO:0042113 1 25

C0018681 GO:0005796 1 25

C0018681 GO:0050654 1 25

C0018681 GO:0045682 1 25

C0018681 GO:0043506 2 25

C0018681 GO:0043500 1 25

C0018681 GO:0045768 1 25

C0018681 GO:0030832 2 25

C0018681 GO:0043462 1 25

C0018681 GO:0006109 1 25

C0018681 GO:0015074 2 25

C0018681 GO:0045766 2 25

C0018681 GO:0002573 2 25

C0018681 GO:0004467 1 25

C0018681 GO:0015695 2 25

C0018681 GO:0004879 1 25

C0018681 GO:0045604 1 25

C0018681 GO:0015026 1 25

C0018681 GO:0016229 1 25

C0018681 GO:0050851 1 25

C0018681 GO:0050852 1 25

C0018681 GO:0030330 1 25

C0018681 GO:0007584 1 25

C0018681 GO:0007585 2 25

C0018681 GO:0008198 1 25

C0018681 GO:0034220 2 25

C0018681 GO:0030501 2 25

C0018681 GO:0003785 2 25

C0018681 GO:0048407 2 25

C0018681 GO:0051646 1 25

C0018681 GO:0034599 1 25

C0018681 GO:0001530 2 25

C0018681 GO:0006312 1 25

C0018681 GO:0048278 1 25

C0018681 GO:0045123 1 25

C0018681 GO:0045124 2 25

C0018681 GO:0046457 1 25

C0018681 GO:0046456 1 25

C0018681 GO:0030900 1 25

C0018681 GO:0004890 1 25

C0018681 GO:0009712 1 25

C0018681 GO:0007090 1 25

C0018681 GO:0070555 1 25

C0018681 GO:0035065 1 25

C0018681 GO:0046637 1 25

C0018681 GO:0046634 1 25

C0018681 GO:0030427 2 25

C0018681 GO:0030426 2 25

C0018681 GO:0046631 2 25

C0018681 GO:0033500 1 25

C0018681 GO:0007292 1 25

C0018681 GO:0050926 2 25

C0018681 GO:0050927 2 25

C0018681 GO:0050920 1 25

C0018681 GO:0008272 1 25

C0018681 GO:0051893 1 25

C0018681 GO:0008375 1 25

C0018681 GO:0051897 3 25

C0018681 GO:0051896 1 25

C0018681 GO:0034762 1 25

C0018681 GO:0044259 1 25

C0018681 GO:0005640 1 25

C0018681 GO:0005523 1 25

C0018681 GO:0043043 1 25

C0018681 GO:0044419 1 25

C0018681 GO:0043325 1 25

C0018681 GO:0045834 1 25

C0018681 GO:0051705 1 25

C0018681 GO:0010896 2 25

C0018681 GO:0010921 2 25

C0018681 GO:0010898 2 25

C0018681 GO:0010875 3 25

C0018681 GO:0032393 2 25

C0018681 GO:0032420 1 25

C0018681 GO:0032934 2 25

C0018681 GO:0019722 1 25

C0018681 GO:0032781 2 25

C0018681 GO:0015300 1 25

C0018681 GO:0046579 1 25

C0018681 GO:0030166 1 25

C0018681 GO:0030048 1 25

C0018681 GO:0030165 1 25

C0018681 GO:0030041 1 25

C0018681 GO:0030514 1 25

C0018681 GO:0010874 2 25

C0018681 GO:0048872 2 25

C0018681 GO:0043648 1 25

C0018681 GO:0051216 1 25

C0018681 GO:0045416 1 25

C0018681 GO:0045414 1 25

C0018681 GO:0048871 2 25

C0018681 GO:0006040 1 25

C0018681 GO:0006041 1 25

C0018681 GO:0006044 2 25

C0018681 GO:0001822 1 25

C0018681 GO:0051353 2 25

C0018681 GO:0030199 1 25

C0018681 GO:0042177 1 25

C0018681 GO:0046849 1 25

C0018681 GO:0019439 2 25

C0018681 GO:0032731 1 25

C0018681 GO:0043034 2 25

C0018681 GO:0030194 1 25

C0018681 GO:0005089 2 25

C0018681 GO:0005088 2 25

C0018681 GO:0007632 2 25

C0018681 GO:0007635 1 25

C0018681 GO:0035023 1 25

C0018681 GO:0032649 1 25

C0018681 GO:0005086 2 25

C0018681 GO:0019934 1 25

C0018681 GO:0051453 1 25

C0018681 GO:0042744 1 25

C0018681 GO:0046320 1 25

C0018681 GO:0032496 1 25

C0018681 GO:0048488 2 25

C0018681 GO:0007062 1 25

C0018681 GO:0002286 1 25

C0018681 GO:0045649 1 25

C0018681 GO:0002285 1 25

C0018681 GO:0004428 1 25

C0018681 GO:0051457 2 25

C0018681 GO:0050699 1 25

C0018681 GO:0050700 1 25

C0018681 GO:0033017 1 25

C0018681 GO:0002366 1 25

C0018681 GO:0007271 1 25

C0018681 GO:0001754 1 25

C0018681 GO:0016763 1 25

C0018681 GO:0001841 2 25

C0018681 GO:0001843 2 25

C0018681 GO:0015145 2 25

C0018681 GO:0015144 1 25

C0018681 GO:0051131 1 25

C0018681 GO:0046545 1 25

C0018681 GO:0030890 1 25

C0018681 GO:0033764 1 25

C0018681 GO:0008013 1 25

C0018681 GO:0008154 1 25

C0018681 GO:0030193 1 25

C0018681 GO:0046626 2 25

C0018681 GO:0048255 1 25

C0018681 GO:0043256 1 25

C0018681 GO:0042632 3 25

C0018681 GO:0045165 1 25

C0018681 GO:0050885 1 25

C0018681 GO:0042734 1 25

C0018681 GO:0004683 1 25

C0018681 GO:0030863 1 25

C0018681 GO:0016445 1 25

C0018681 GO:0002715 1 25

C0018681 GO:0030864 1 25

C0018681 GO:0033261 2 25

C0018681 GO:0045638 1 25

C0018681 GO:0045730 2 25

C0018681 GO:0045737 2 25

C0018681 GO:0030195 2 25

C0018681 GO:0005795 1 25

C0018681 GO:0015662 2 25

C0018681 GO:0032732 1 25

C0018681 GO:0005537 1 25

C0018681 GO:0005884 2 25

C0018681 GO:0046718 1 25

C0018681 GO:0032376 3 25

C0018681 GO:0042752 1 25

C0018681 GO:0004114 1 25

C0018681 GO:0002819 1 25

C0018681 GO:0035148 2 25

C0018681 GO:0006941 1 25

C0018681 GO:0030260 1 25

C0018681 GO:0005903 1 25

C0018681 GO:0048029 2 25

C0018681 GO:0007266 2 25

C0018681 GO:0052126 1 25

C0018681 GO:0009798 1 25

C0018681 GO:0033280 2 25

C0018681 GO:0043087 1 25

C0018681 GO:0009124 2 25

C0018681 GO:0001540 1 25

C0018681 GO:0006342 1 25

C0018681 GO:0000041 2 25

C0018681 GO:0046782 1 25

C0018681 GO:0046467 1 25

C0018681 GO:0015297 1 25

C0018681 GO:0015296 1 25

C0018681 GO:0046165 1 25

C0018681 GO:0015299 1 25

C0018681 GO:0015298 1 25

C0018681 GO:0055088 1 25

C0018681 GO:0045621 1 25

C0018681 GO:0015807 4 25

C0018681 GO:0015800 1 25

C0018681 GO:0010830 1 25

C0018681 GO:0003995 1 25

C0018681 GO:0001894 1 25

C0018681 GO:0001893 2 25

C0018681 GO:0045598 2 25

C0018681 GO:0008635 1 25

C0018681 GO:0045622 2 25

C0018681 GO:0007568 2 25

C0018681 GO:0006081 1 25

C0018681 GO:0043603 1 25

C0018681 GO:0006805 1 25

C0018681 GO:0046329 1 25

C0018681 GO:0042476 2 25

C0018681 GO:0045296 2 25

C0018681 GO:0045862 1 25

C0018681 GO:0006821 1 25

C0018681 GO:0042269 1 25

C0018681 GO:0030099 1 25

C0018681 GO:0043370 2 25

C0018681 GO:0045861 3 25

C0018681 GO:0045749 1 25

C0018681 GO:0016459 2 25

C0018681 GO:0000185 2 25

C0018681 GO:0019201 1 25

C0018681 GO:0030021 1 25

C0018681 GO:0014910 1 25

C0018681 GO:0045582 1 25

C0018681 GO:0046326 1 25

C0018681 GO:0052192 1 25

C0018681 GO:0050829 1 25

C0018681 GO:0032862 1 25

C0018681 GO:0006879 1 25

C0018681 GO:0030169 1 25

C0018681 GO:0030132 1 25

C0018681 GO:0060228 1 25

C0018681 GO:0030134 1 25

C0018681 GO:0030641 1 25

C0018681 GO:0045684 1 25

C0018681 GO:0050661 1 25

C0018681 GO:0050660 1 25

C0018681 GO:0034765 1 25

C0018681 GO:0016641 1 25

C0018681 GO:0042516 1 25

C0018681 GO:0042517 1 25

C0018681 GO:0051262 1 25

C0018681 GO:0008526 1 25

C0018681 GO:0006721 2 25

C0018681 GO:0010466 2 25

C0018681 GO:0070412 1 25

C0018681 GO:0046504 1 25

C0018681 GO:0070410 1 25

C0018681 GO:0070411 1 25

C0018681 GO:0010745 1 25

C0018681 GO:0010741 1 25

C0018681 GO:0010743 1 25

C0018681 GO:0034101 2 25

C0018681 GO:0034103 2 25

C0018681 GO:0048662 1 25

C0018681 GO:0048660 1 25

C0018681 GO:0048741 1 25

C0018681 GO:0042104 1 25

C0018681 GO:0048742 2 25

C0018681 GO:0042100 1 25

C0018681 GO:0048747 1 25

C0018681 GO:0042102 1 25

C0018681 GO:0002449 2 25

C0018681 GO:0048041 1 25

C0018681 GO:0031214 3 25

C0018681 GO:0002440 3 25

C0018681 GO:0002443 1 25

C0018681 GO:0045807 1 25

C0018681 GO:0051004 1 25

C0018681 GO:0051180 1 25

C0018681 GO:0004806 1 25

C0018681 GO:0034637 2 25

C0018681 GO:0033344 1 25

C0018681 GO:0002250 2 25

C0018681 GO:0006970 1 25

C0018681 GO:0001937 1 25

C0018681 GO:0015036 1 25

C0002871 GO:0070665 1 10

C0002871 GO:0005786 1 10

C0002871 GO:0005788 1 10

C0002871 GO:0051044 1 10

C0002871 GO:0030228 2 10

C0002871 GO:0048638 1 10

C0002871 GO:0031970 1 10

C0002871 GO:0046887 1 10

C0002871 GO:0050681 1 10

C0002871 GO:0032846 1 10

C0002871 GO:0046631 2 10

C0002871 GO:0009299 1 10

C0002871 GO:0050926 1 10

C0002871 GO:0050808 1 10

C0002871 GO:0042558 1 10

C0002871 GO:0031057 1 10

C0002871 GO:0045165 2 10

C0002871 GO:0048260 1 10

C0002871 GO:0016863 2 10

C0002871 GO:0001894 1 10

C0002871 GO:0043489 1 10

C0002871 GO:0016860 1 10

C0002871 GO:0015085 2 10

C0002871 GO:0005072 1 10

C0002871 GO:0004364 1 10

C0002871 GO:0000002 1 10

C0002871 GO:0042605 1 10

C0002871 GO:0007213 1 10

C0002871 GO:0005770 1 10

C0002871 GO:0005544 1 10

C0002871 GO:0018210 2 10

C0002871 GO:0055117 1 10

C0002871 GO:0044419 1 10

C0002871 GO:0045833 1 10

C0002871 GO:0045839 2 10

C0002871 GO:0010875 2 10

C0002871 GO:0043120 1 10

C0002871 GO:0010522 1 10

C0002871 GO:0033032 1 10

C0002871 GO:0051057 1 10

C0002871 GO:0070228 1 10

C0002871 GO:0051055 1 10

C0002871 GO:0032934 1 10

C0002871 GO:0015718 1 10

C0002871 GO:0042439 1 10

C0002871 GO:0043966 1 10

C0002871 GO:0002757 1 10

C0002871 GO:0005159 1 10

C0002871 GO:0014003 1 10

C0002871 GO:0048771 1 10

C0002871 GO:0043536 1 10

C0002871 GO:0046330 1 10

C0002871 GO:0043534 1 10

C0002871 GO:0007173 2 10

C0002871 GO:0006477 1 10

C0002871 GO:0010975 1 10

C0002871 GO:0045912 1 10

C0002871 GO:0051701 1 10

C0002871 GO:0030514 1 10

C0002871 GO:0070875 1 10

C0002871 GO:0010874 1 10

C0002871 GO:0030510 1 10

C0002871 GO:0043648 1 10

C0002871 GO:0002429 1 10

C0002871 GO:0030509 1 10

C0002871 GO:0016812 1 10

C0002871 GO:0060395 1 10

C0002871 GO:0016055 1 10

C0002871 GO:0080010 1 10

C0002871 GO:0060393 1 10

C0002871 GO:0050927 1 10

C0002871 GO:0046849 1 10

C0002871 GO:0051784 2 10

C0002871 GO:0004806 1 10

C0002871 GO:0005388 1 10

C0002871 GO:0004709 1 10

C0002871 GO:0060416 1 10

C0002871 GO:0002702 1 10

C0002871 GO:0021537 1 10

C0002871 GO:0060263 1 10

C0002871 GO:0002700 1 10

C0002871 GO:0008252 1 10

C0002871 GO:0006929 1 10

C0002871 GO:0046658 1 10

C0002871 GO:0032946 1 10

C0002871 GO:0043393 1 10

C0002871 GO:0000786 1 10

C0002871 GO:0048002 1 10

C0002871 GO:0021915 1 10

C0002871 GO:0055102 1 10

C0002871 GO:0021700 1 10

C0002871 GO:0030071 1 10

C0002871 GO:0048488 3 10

C0002871 GO:0007062 2 10

C0002871 GO:0003205 1 10

C0002871 GO:0031577 2 10

C0002871 GO:0003206 1 10

C0002871 GO:0006563 1 10

C0002871 GO:0048660 1 10

C0002871 GO:0002285 2 10

C0002871 GO:0004428 1 10

C0002871 GO:0045646 1 10

C0002871 GO:0043548 1 10

C0002871 GO:0050700 2 10

C0002871 GO:0008088 1 10

C0002871 GO:0005391 1 10

C0002871 GO:0043028 1 10

C0002871 GO:0045429 1 10

C0002871 GO:0002366 1 10

C0002871 GO:0006687 1 10

C0002871 GO:0019894 1 10

C0002871 GO:0045725 1 10

C0002871 GO:0005520 1 10

C0002871 GO:0045727 1 10

C0002871 GO:0030879 1 10

C0002871 GO:0043027 1 10

C0002871 GO:0004551 1 10

C0002871 GO:0071174 2 10

C0002871 GO:0019883 1 10

C0002871 GO:0071173 2 10

C0002871 GO:0005071 2 10

C0002871 GO:0030371 1 10

C0002871 GO:0048500 1 10

C0002871 GO:0048709 1 10

C0002871 GO:0008013 1 10

C0002871 GO:0060070 2 10

C0002871 GO:0030276 1 10

C0002871 GO:0048255 1 10

C0002871 GO:0043256 1 10

C0002871 GO:0019882 1 10

C0002871 GO:0005930 1 10

C0002871 GO:0002263 1 10

C0002871 GO:0045851 1 10

C0002871 GO:0030675 1 10

C0002871 GO:0008091 1 10

C0002871 GO:0009952 1 10

C0002871 GO:0042734 1 10

C0002871 GO:0030863 1 10

C0002871 GO:0050650 1 10

C0002871 GO:0016830 1 10

C0002871 GO:0008417 1 10

C0002871 GO:0033261 1 10

C0002871 GO:0017080 2 10

C0002871 GO:0045737 1 10

C0002871 GO:0010677 1 10

C0002871 GO:0008301 1 10

C0002871 GO:0051452 1 10

C0002871 GO:0060192 1 10

C0002871 GO:0045841 2 10

C0002871 GO:0032376 2 10

C0002871 GO:0050810 1 10

C0002871 GO:0008028 1 10

C0002871 GO:0018212 1 10

C0002871 GO:0048145 1 10

C0002871 GO:0015695 1 10

C0002871 GO:0046579 1 10

C0002871 GO:0017022 2 10

C0002871 GO:0009312 1 10

C0002871 GO:0005901 1 10

C0002871 GO:0031228 1 10

C0002871 GO:0006942 1 10

C0002871 GO:0004859 1 10

C0002871 GO:0035113 1 10

C0002871 GO:0051453 1 10

C0002871 GO:0005871 1 10

C0002871 GO:0033280 2 10

C0002871 GO:0034284 1 10

C0002871 GO:0048385 1 10

C0002871 GO:0009124 2 10

C0002871 GO:0031225 1 10

C0002871 GO:0043168 1 10

C0002871 GO:0043560 1 10

C0002871 GO:0050885 2 10

C0002871 GO:0030546 1 10

C0002871 GO:0001709 1 10

C0002871 GO:0032370 2 10

C0002871 GO:0032373 2 10

C0002871 GO:0001530 1 10

C0002871 GO:0043325 1 10

C0002871 GO:0032273 1 10

C0002871 GO:0008034 2 10

C0002871 GO:0032984 1 10

C0002871 GO:0008329 1 10

C0002871 GO:0031330 1 10

C0002871 GO:0007272 1 10

C0002871 GO:0015929 1 10

C0002871 GO:0034637 1 10

C0002871 GO:0008308 1 10

C0002871 GO:0015807 1 10

C0002871 GO:0001654 1 10

C0002871 GO:0046870 1 10

C0002871 GO:0005593 1 10

C0002871 GO:0010595 1 10

C0002871 GO:0007131 1 10

C0002871 GO:0042562 1 10

C0002871 GO:0051983 2 10

C0002871 GO:0051881 1 10

C0002871 GO:0050679 1 10

C0002871 GO:0050671 1 10

C0002871 GO:0002237 1 10

C0002871 GO:0045862 1 10

C0002871 GO:0005518 1 10

C0002871 GO:0032364 1 10

C0002871 GO:0017046 1 10

C0002871 GO:0018107 1 10

C0002871 GO:0009895 1 10

C0002871 GO:0030326 1 10

C0002871 GO:0050770 1 10

C0002871 GO:0042177 1 10

C0002871 GO:0016338 1 10

C0002871 GO:0007098 1 10

C0002871 GO:0018108 1 10

C0002871 GO:0060021 1 10

C0002871 GO:0046112 1 10

C0002871 GO:0010948 1 10

C0002871 GO:0008210 1 10

C0002871 GO:0009108 1 10

C0002871 GO:0050654 1 10

C0002871 GO:0034623 1 10

C0002871 GO:0046890 1 10

C0002871 GO:0043506 1 10

C0002871 GO:0002724 2 10

C0002871 GO:0030169 1 10

C0002871 GO:0009749 1 10

C0002871 GO:0002720 1 10

C0002871 GO:0030641 1 10

C0002871 GO:0045768 1 10

C0002871 GO:0032355 1 10

C0002871 GO:0009068 1 10

C0002871 GO:0030520 1 10

C0002871 GO:0032210 1 10

C0002871 GO:0045767 1 10

C0002871 GO:0006081 1 10

C0002871 GO:0070412 1 10

C0002871 GO:0070410 3 10

C0002871 GO:0005758 1 10

C0002871 GO:0051353 1 10

C0002871 GO:0051897 1 10

C0002871 GO:0005697 1 10

C0002871 GO:0050851 1 10

C0002871 GO:0050852 1 10

C0002871 GO:0034451 1 10

C0002871 GO:0048662 1 10

C0002871 GO:0070120 1 10

C0002871 GO:0007584 1 10

C0002871 GO:0042100 1 10

C0002871 GO:0050690 1 10

C0002871 GO:0033483 1 10

C0002871 GO:0009112 2 10

C0002871 GO:0015175 1 10

C0002871 GO:0002718 3 10

C0002871 GO:0002286 2 10

C0002871 GO:0034185 1 10

C0002871 GO:0009746 1 10

C0002871 GO:0005605 2 10

C0002871 GO:0006493 1 10

C0002871 GO:0010594 1 10

C0002871 GO:0045123 1 10

C0002871 GO:0002793 1 10

C0002871 GO:0051923 1 10

C0002871 GO:0043535 1 10

C0002871 GO:0003707 1 10

C0002871 GO:0030900 1 10

C0002871 GO:0007094 2 10

C0002871 GO:0044403 1 10

C0002871 GO:0006413 1 10

C0002871 GO:0008366 1 10

C0004134 GO:0060192 1 2

C0004134 GO:0050808 1 2

C0004134 GO:0008028 1 2

C0004134 GO:0050700 1 2

C0004134 GO:0031970 1 2

C0004134 GO:0032846 1 2

C0004134 GO:0017022 1 2

C0004134 GO:0009299 1 2

C0004134 GO:0004859 1 2

C0004134 GO:0008088 1 2

C0004134 GO:0046890 1 2

C0004134 GO:0046112 1 2

C0004134 GO:0033280 1 2

C0004134 GO:0055102 1 2

C0004134 GO:0021700 1 2

C0004134 GO:0015085 1 2

C0004134 GO:0006563 1 2

C0004134 GO:0002285 1 2

C0004134 GO:0016863 1 2

C0004134 GO:0030546 1 2

C0004134 GO:0005544 1 2

C0004134 GO:0042558 1 2

C0004134 GO:0045833 1 2

C0004134 GO:0005758 1 2

C0004134 GO:0005520 1 2

C0004134 GO:0045727 1 2

C0004134 GO:0048145 1 2

C0004134 GO:0051055 1 2

C0004134 GO:0008034 1 2

C0004134 GO:0015718 1 2

C0004134 GO:0042100 1 2

C0004134 GO:0015929 1 2

C0004134 GO:0033483 1 2

C0004134 GO:0009112 1 2

C0004134 GO:0046330 1 2

C0004134 GO:0046887 1 2

C0004134 GO:0002286 1 2

C0004134 GO:0005930 1 2

C0004134 GO:0030169 1 2

C0004134 GO:0009746 1 2

C0004134 GO:0042734 1 2

C0004134 GO:0034284 1 2

C0004134 GO:0032364 1 2

C0004134 GO:0002793 1 2

C0004134 GO:0016830 1 2

C0004134 GO:0080010 1 2

C0004134 GO:0008417 1 2

C0004134 GO:0009749 1 2

C0004134 GO:0005388 1 2

C0004134 GO:0005518 1 2

C0004134 GO:0060416 1 2

C0007787 GO:0060192 1 1

C0007787 GO:0008028 1 1

C0007787 GO:0031970 1 1

C0007787 GO:0032846 1 1

C0007787 GO:0017022 1 1

C0007787 GO:0009299 1 1

C0007787 GO:0004859 1 1

C0007787 GO:0008088 1 1

C0007787 GO:0046890 1 1

C0007787 GO:0033280 1 1

C0007787 GO:0055102 1 1

C0007787 GO:0021700 1 1

C0007787 GO:0015085 1 1

C0007787 GO:0006563 1 1

C0007787 GO:0002285 1 1

C0007787 GO:0030546 1 1

C0007787 GO:0005544 1 1

C0007787 GO:0042558 1 1

C0007787 GO:0045833 1 1

C0007787 GO:0005758 1 1

C0007787 GO:0005520 1 1

C0007787 GO:0051055 1 1

C0007787 GO:0015718 1 1

C0007787 GO:0042100 1 1

C0007787 GO:0046330 1 1

C0007787 GO:0002286 1 1

C0007787 GO:0005930 1 1

C0007787 GO:0042734 1 1

C0007787 GO:0080010 1 1

C0007787 GO:0008417 1 1

C0007787 GO:0005388 1 1

C0007787 GO:0060416 1 1

C0009763 GO:0043954 1 4

C0009763 GO:0031970 1 4

C0009763 GO:0050681 1 4

C0009763 GO:0032846 1 4

C0009763 GO:0009299 1 4

C0009763 GO:0042558 1 4

C0009763 GO:0015085 2 4

C0009763 GO:0005072 1 4

C0009763 GO:0015082 1 4

C0009763 GO:0070665 1 4

C0009763 GO:0005544 1 4

C0009763 GO:0006266 1 4

C0009763 GO:0045833 1 4

C0009763 GO:0045839 1 4

C0009763 GO:0060541 1 4

C0009763 GO:0051055 1 4

C0009763 GO:0015718 1 4

C0009763 GO:0004029 1 4

C0009763 GO:0043966 1 4

C0009763 GO:0007176 1 4

C0009763 GO:0046330 1 4

C0009763 GO:0007173 1 4

C0009763 GO:0030514 1 4

C0009763 GO:0070875 1 4

C0009763 GO:0030510 1 4

C0009763 GO:0060395 1 4

C0009763 GO:0002274 1 4

C0009763 GO:0080010 1 4

C0009763 GO:0001910 1 4

C0009763 GO:0046849 1 4

C0009763 GO:0051784 1 4

C0009763 GO:0005388 2 4

C0009763 GO:0060416 1 4

C0009763 GO:0045494 1 4

C0009763 GO:0045841 1 4

C0009763 GO:0007635 1 4

C0009763 GO:0051103 1 4

C0009763 GO:0032946 1 4

C0009763 GO:0055102 1 4

C0009763 GO:0021700 1 4

C0009763 GO:0030071 1 4

C0009763 GO:0007062 1 4

C0009763 GO:0003205 1 4

C0009763 GO:0031577 1 4

C0009763 GO:0003206 1 4

C0009763 GO:0006563 1 4

C0009763 GO:0002285 1 4

C0009763 GO:0004428 1 4

C0009763 GO:0006885 1 4

C0009763 GO:0008088 2 4

C0009763 GO:0006687 1 4

C0009763 GO:0045725 1 4

C0009763 GO:0005520 1 4

C0009763 GO:0005254 1 4

C0009763 GO:0071174 1 4

C0009763 GO:0071173 1 4

C0009763 GO:0005071 1 4

C0009763 GO:0048709 1 4

C0009763 GO:0060070 1 4

C0009763 GO:0030276 1 4

C0009763 GO:0043256 1 4

C0009763 GO:0005930 1 4

C0009763 GO:0009952 1 4

C0009763 GO:0042734 1 4

C0009763 GO:0050650 1 4

C0009763 GO:0008417 1 4

C0009763 GO:0045730 1 4

C0009763 GO:0005247 1 4

C0009763 GO:0060192 1 4

C0009763 GO:0008028 1 4

C0009763 GO:0017022 1 4

C0009763 GO:0014003 1 4

C0009763 GO:0004859 1 4

C0009763 GO:0051453 1 4

C0009763 GO:0033280 1 4

C0009763 GO:0050885 1 4

C0009763 GO:0030546 1 4

C0009763 GO:0001709 1 4

C0009763 GO:0002224 1 4

C0009763 GO:0030004 1 4

C0009763 GO:0051983 1 4

C0009763 GO:0050679 1 4

C0009763 GO:0050671 1 4

C0009763 GO:0017144 1 4

C0009763 GO:0019200 1 4

C0009763 GO:0030324 1 4

C0009763 GO:0042058 1 4

C0009763 GO:0008210 1 4

C0009763 GO:0009108 1 4

C0009763 GO:0050654 1 4

C0009763 GO:0046890 1 4

C0009763 GO:0030641 1 4

C0009763 GO:0009068 1 4

C0009763 GO:0030216 1 4

C0009763 GO:0070412 1 4

C0009763 GO:0070410 1 4

C0009763 GO:0005758 1 4

C0009763 GO:0010469 1 4

C0009763 GO:0042100 1 4

C0009763 GO:0042102 1 4

C0009763 GO:0055067 1 4

C0009763 GO:0002286 1 4

C0009763 GO:0045646 1 4

C0009763 GO:0007094 1 4

C0015230 GO:0051043 1 12

C0015230 GO:0051044 1 12

C0015230 GO:0030228 1 12

C0015230 GO:0043954 1 12

C0015230 GO:0010677 1 12

C0015230 GO:0002703 1 12

C0015230 GO:0016863 2 12

C0015230 GO:0016860 1 12

C0015230 GO:0006306 1 12

C0015230 GO:0006305 1 12

C0015230 GO:0043205 1 12

C0015230 GO:0018210 1 12

C0015230 GO:0004715 1 12

C0015230 GO:0046504 1 12

C0015230 GO:0002822 1 12

C0015230 GO:0021510 1 12

C0015230 GO:0046330 1 12

C0015230 GO:0006477 1 12

C0015230 GO:0030291 1 12

C0015230 GO:0001843 1 12

C0015230 GO:0034284 1 12

C0015230 GO:0016812 1 12

C0015230 GO:0080010 1 12

C0015230 GO:0051784 1 12

C0015230 GO:0021537 1 12

C0015230 GO:0009895 2 12

C0015230 GO:0051224 1 12

C0015230 GO:0030071 1 12

C0015230 GO:0042558 1 12

C0015230 GO:0043028 1 12

C0015230 GO:0046850 1 12

C0015230 GO:0006110 1 12

C0015230 GO:0045912 1 12

C0015230 GO:0043025 1 12

C0015230 GO:0043027 1 12

C0015230 GO:0019882 1 12

C0015230 GO:0019883 1 12

C0015230 GO:0005071 1 12

C0015230 GO:0005138 2 12

C0015230 GO:0060606 1 12

C0015230 GO:0035303 1 12

C0015230 GO:0004437 1 12

C0015230 GO:0050715 1 12

C0015230 GO:0050714 1 12

C0015230 GO:0050650 1 12

C0015230 GO:0005436 1 12

C0015230 GO:0015074 1 12

C0015230 GO:0015179 1 12

C0015230 GO:0048638 1 12

C0015230 GO:0008028 1 12

C0015230 GO:0048145 1 12

C0015230 GO:0048634 1 12

C0015230 GO:0031228 1 12

C0015230 GO:0004693 1 12

C0015230 GO:0045078 1 12

C0015230 GO:0043489 1 12

C0015230 GO:0045072 1 12

C0015230 GO:0030705 1 12

C0015230 GO:0016459 1 12

C0015230 GO:0045622 1 12

C0015230 GO:0008286 1 12

C0015230 GO:0006879 1 12

C0015230 GO:0008329 1 12

C0015230 GO:0015929 1 12

C0015230 GO:0045211 1 12

C0015230 GO:0001654 1 12

C0015230 GO:0045216 1 12

C0015230 GO:0046887 1 12

C0015230 GO:0051983 1 12

C0015230 GO:0042269 1 12

C0015230 GO:0043120 1 12

C0015230 GO:0004190 2 12

C0015230 GO:0018107 1 12

C0015230 GO:0019047 1 12

C0015230 GO:0017048 2 12

C0015230 GO:0045884 1 12

C0015230 GO:0007270 1 12

C0015230 GO:0006096 1 12

C0015230 GO:0032350 1 12

C0015230 GO:0042354 1 12

C0015230 GO:0055092 1 12

C0015230 GO:0070668 1 12

C0015230 GO:0019213 1 12

C0015230 GO:0021915 1 12

C0015230 GO:0070120 1 12

C0015230 GO:0048593 1 12

C0015230 GO:0032856 1 12

C0015230 GO:0001838 1 12

C0015230 GO:0010595 1 12

C0015230 GO:0010594 2 12

C0015230 GO:0005044 1 12

C0015230 GO:0050995 1 12

C0015230 GO:0050994 1 12

C0015230 GO:0050654 1 12

C0015230 GO:0032321 1 12

C0015230 GO:0006413 1 12

C0015230 GO:0005149 1 12

C0015230 GO:0007612 1 12

C0015230 GO:0009299 1 12

C0015230 GO:0007162 1 12

C0015230 GO:0051879 1 12

C0015230 GO:0043524 1 12

C0015230 GO:0019915 1 12

C0015230 GO:0031343 1 12

C0015230 GO:0002573 1 12

C0015230 GO:0016597 1 12

C0015230 GO:0030742 1 12

C0015230 GO:0004407 1 12

C0015230 GO:0002263 1 12

C0015230 GO:0005544 1 12

C0015230 GO:0045667 1 12

C0015230 GO:0045661 1 12

C0015230 GO:0045669 1 12

C0015230 GO:0033032 1 12

C0015230 GO:0016202 1 12

C0015230 GO:0051055 1 12

C0015230 GO:0043966 1 12

C0015230 GO:0014020 1 12

C0015230 GO:0004859 1 12

C0015230 GO:0005388 1 12

C0015230 GO:0045841 1 12

C0015230 GO:0050820 1 12

C0015230 GO:0048002 1 12

C0015230 GO:0030286 1 12

C0015230 GO:0007530 1 12

C0015230 GO:0006563 1 12

C0015230 GO:0051180 1 12

C0015230 GO:0046915 1 12

C0015230 GO:0008088 2 12

C0015230 GO:0020037 1 12

C0015230 GO:0032768 1 12

C0015230 GO:0050796 1 12

C0015230 GO:0060070 1 12

C0015230 GO:0030069 1 12

C0015230 GO:0046660 1 12

C0015230 GO:0009953 1 12

C0015230 GO:0009952 1 12

C0015230 GO:0032364 1 12

C0015230 GO:0017080 1 12

C0015230 GO:0043010 1 12

C0015230 GO:0003785 1 12

C0015230 GO:0060192 1 12

C0015230 GO:0016790 1 12

C0015230 GO:0019956 1 12

C0015230 GO:0007043 1 12

C0015230 GO:0030546 1 12

C0015230 GO:0010563 1 12

C0015230 GO:0031397 1 12

C0015230 GO:0005355 1 12

C0015230 GO:0008034 1 12

C0015230 GO:0002237 1 12

C0015230 GO:0045619 2 12

C0015230 GO:0034405 1 12

C0015230 GO:0045616 1 12

C0015230 GO:0005518 1 12

C0015230 GO:0016409 1 12

C0015230 GO:0016331 1 12

C0015230 GO:0030323 1 12

C0015230 GO:0016338 2 12

C0015230 GO:0009108 1 12

C0015230 GO:0046890 1 12

C0015230 GO:0016538 1 12

C0015230 GO:0045936 1 12

C0015230 GO:0045089 1 12

C0015230 GO:0007266 1 12

C0015230 GO:0035295 1 12

C0015230 GO:0009746 1 12

C0015230 GO:0002793 1 12

C0015230 GO:0051923 1 12

C0015230 GO:0003705 1 12

C0015230 GO:0009749 1 12

C0015230 GO:0060538 2 12

C0015230 GO:0046697 1 12

C0015230 GO:0031970 2 12

C0015230 GO:0032846 1 12

C0015230 GO:0005788 1 12

C0015230 GO:0031058 1 12

C0015230 GO:0015085 2 12

C0015230 GO:0005072 1 12

C0015230 GO:0048864 1 12

C0015230 GO:0016627 1 12

C0015230 GO:0005770 1 12

C0015230 GO:0070001 2 12

C0015230 GO:0032722 1 12

C0015230 GO:0070228 1 12

C0015230 GO:0015718 1 12

C0015230 GO:0005159 1 12

C0015230 GO:0005158 1 12

C0015230 GO:0042129 1 12

C0015230 GO:0043536 1 12

C0015230 GO:0043535 2 12

C0015230 GO:0007173 1 12

C0015230 GO:0046631 1 12

C0015230 GO:0045725 1 12

C0015230 GO:0070875 1 12

C0015230 GO:0060395 1 12

C0015230 GO:0060393 2 12

C0015230 GO:0002377 1 12

C0015230 GO:0001910 1 12

C0015230 GO:0033558 1 12

C0015230 GO:0006929 1 12

C0015230 GO:0021700 2 12

C0015230 GO:0031577 1 12

C0015230 GO:0043548 1 12

C0015230 GO:0005522 1 12

C0015230 GO:0048641 1 12

C0015230 GO:0005520 1 12

C0015230 GO:0045727 1 12

C0015230 GO:0030879 1 12

C0015230 GO:0030276 1 12

C0015230 GO:0005930 2 12

C0015230 GO:0042301 1 12

C0015230 GO:0044275 1 12

C0015230 GO:0008091 2 12

C0015230 GO:0016830 1 12

C0015230 GO:0050810 1 12

C0015230 GO:0032770 1 12

C0015230 GO:0010921 1 12

C0015230 GO:0017022 2 12

C0015230 GO:0045580 2 12

C0015230 GO:0045429 1 12

C0015230 GO:0046677 1 12

C0015230 GO:0007250 1 12

C0015230 GO:0032374 1 12

C0015230 GO:0001709 1 12

C0015230 GO:0032371 1 12

C0015230 GO:0032370 3 12

C0015230 GO:0032373 3 12

C0015230 GO:0050819 1 12

C0015230 GO:0043325 2 12

C0015230 GO:0070688 1 12

C0015230 GO:0060562 1 12

C0015230 GO:0031330 1 12

C0015230 GO:0031333 1 12

C0015230 GO:0000217 1 12

C0015230 GO:0004861 1 12

C0015230 GO:0005593 1 12

C0015230 GO:0050671 1 12

C0015230 GO:0001818 1 12

C0015230 GO:0005160 1 12

C0015230 GO:0005161 1 12

C0015230 GO:0008417 1 12

C0015230 GO:0043506 2 12

C0015230 GO:0045768 1 12

C0015230 GO:0045767 1 12

C0015230 GO:0015695 1 12

C0015230 GO:0045604 1 12

C0015230 GO:0009112 1 12

C0015230 GO:0002715 1 12

C0015230 GO:0051646 1 12

C0015230 GO:0001530 1 12

C0015230 GO:0045123 1 12

C0015230 GO:0045124 1 12

C0015230 GO:0007094 1 12

C0015230 GO:0032376 3 12

C0015230 GO:0046637 1 12

C0015230 GO:0046634 1 12

C0015230 GO:0050681 1 12

C0015230 GO:0008585 1 12

C0015230 GO:0050926 1 12

C0015230 GO:0050927 1 12

C0015230 GO:0051893 1 12

C0015230 GO:0008375 1 12

C0015230 GO:0051897 1 12

C0015230 GO:0051896 1 12

C0015230 GO:0007519 2 12

C0015230 GO:0045833 1 12

C0015230 GO:0010896 1 12

C0015230 GO:0010898 1 12

C0015230 GO:0045839 1 12

C0015230 GO:0030510 1 12

C0015230 GO:0006760 1 12

C0015230 GO:0019827 1 12

C0015230 GO:0030048 1 12

C0015230 GO:0030514 1 12

C0015230 GO:0010874 2 12

C0015230 GO:0010875 3 12

C0015230 GO:0051353 2 12

C0015230 GO:0060416 1 12

C0015230 GO:0043034 2 12

C0015230 GO:0030194 1 12

C0015230 GO:0007632 1 12

C0015230 GO:0019934 1 12

C0015230 GO:0032496 1 12

C0015230 GO:0055102 1 12

C0015230 GO:0048488 1 12

C0015230 GO:0007062 1 12

C0015230 GO:0002286 2 12

C0015230 GO:0002285 2 12

C0015230 GO:0004428 1 12

C0015230 GO:0045646 1 12

C0015230 GO:0050700 2 12

C0015230 GO:0015149 1 12

C0015230 GO:0002366 1 12

C0015230 GO:0001841 1 12

C0015230 GO:0006688 1 12

C0015230 GO:0015145 1 12

C0015230 GO:0071174 1 12

C0015230 GO:0046545 1 12

C0015230 GO:0071173 1 12

C0015230 GO:0030890 1 12

C0015230 GO:0048255 1 12

C0015230 GO:0042632 1 12

C0015230 GO:0042102 1 12

C0015230 GO:0045165 1 12

C0015230 GO:0042734 1 12

C0015230 GO:0031645 1 12

C0015230 GO:0030864 1 12

C0015230 GO:0045730 1 12

C0015230 GO:0030195 1 12

C0015230 GO:0050808 1 12

C0015230 GO:0035148 1 12

C0015230 GO:0006941 1 12

C0015230 GO:0005903 1 12

C0015230 GO:0033280 2 12

C0015230 GO:0009124 1 12

C0015230 GO:0046782 1 12

C0015230 GO:0015807 3 12

C0015230 GO:0001894 1 12

C0015230 GO:0001893 1 12

C0015230 GO:0006081 1 12

C0015230 GO:0043603 1 12

C0015230 GO:0043370 1 12

C0015230 GO:0045861 1 12

C0015230 GO:0019200 1 12

C0015230 GO:0032862 1 12

C0015230 GO:0046112 1 12

C0015230 GO:0045682 1 12

C0015230 GO:0030169 1 12

C0015230 GO:0045684 1 12

C0015230 GO:0070412 1 12

C0015230 GO:0070410 1 12

C0015230 GO:0005758 1 12

C0015230 GO:0010741 1 12

C0015230 GO:0034103 1 12

C0015230 GO:0048662 1 12

C0015230 GO:0048660 1 12

C0015230 GO:0009247 1 12

C0015230 GO:0048742 1 12

C0015230 GO:0042100 1 12

C0015230 GO:0033483 1 12

C0015230 GO:0031214 2 12

C0015230 GO:0002440 1 12

C0015230 GO:0051181 1 12

C0015230 GO:0001937 1 12

C0018801 GO:0060192 1 2

C0018801 GO:0006633 1 2

C0018801 GO:0007632 1 2

C0018801 GO:0006636 1 2

C0018801 GO:0018107 1 2

C0018801 GO:0008028 1 2

C0018801 GO:0009636 1 2

C0018801 GO:0031970 1 2

C0018801 GO:0060173 1 2

C0018801 GO:0032846 1 2

C0018801 GO:0017022 1 2

C0018801 GO:0001822 1 2

C0018801 GO:0016917 1 2

C0018801 GO:0009299 1 2

C0018801 GO:0055072 1 2

C0018801 GO:0004859 1 2

C0018801 GO:0008088 1 2

C0018801 GO:0046890 1 2

C0018801 GO:0007270 1 2

C0018801 GO:0006879 1 2

C0018801 GO:0033280 1 2

C0018801 GO:0055102 1 2

C0018801 GO:0021700 1 2

C0018801 GO:0050431 1 2

C0018801 GO:0015085 1 2

C0018801 GO:0048261 1 2

C0018801 GO:0006563 1 2

C0018801 GO:0002285 1 2

C0018801 GO:0051224 1 2

C0018801 GO:0030546 1 2

C0018801 GO:0005544 1 2

C0018801 GO:0033500 1 2

C0018801 GO:0042558 1 2

C0018801 GO:0010745 1 2

C0018801 GO:0045833 1 2

C0018801 GO:0001754 1 2

C0018801 GO:0005758 1 2

C0018801 GO:0005520 1 2

C0018801 GO:0051055 1 2

C0018801 GO:0034103 1 2

C0018801 GO:0015718 1 2

C0018801 GO:0046850 1 2

C0018801 GO:0003073 1 2

C0018801 GO:0042100 2 2

C0018801 GO:0034637 1 2

C0018801 GO:0046330 1 2

C0018801 GO:0014069 1 2

C0018801 GO:0001655 1 2

C0018801 GO:0045912 1 2

C0018801 GO:0002286 1 2

C0018801 GO:0060021 1 2

C0018801 GO:0045124 1 2

C0018801 GO:0005930 1 2

C0018801 GO:0004435 1 2

C0018801 GO:0004434 1 2

C0018801 GO:0004629 1 2

C0018801 GO:0042734 1 2

C0018801 GO:0042593 1 2

C0018801 GO:0008272 1 2

C0018801 GO:0003746 1 2

C0018801 GO:0042274 1 2

C0018801 GO:0080010 1 2

C0018801 GO:0008417 1 2

C0018801 GO:0046530 1 2

C0018801 GO:0006693 1 2

C0018801 GO:0006692 1 2

C0018801 GO:0004890 1 2

C0018801 GO:0010677 1 2

C0018801 GO:0015175 1 2

C0018801 GO:0005388 1 2

C0018801 GO:0005518 1 2

C0018801 GO:0016445 1 2

C0018801 GO:0060416 1 2

C0018801 GO:0048736 1 2

C0018801 GO:0048029 1 2

C0032285 GO:0006633 1 4

C0032285 GO:0051044 1 4

C0032285 GO:0030228 1 4

C0032285 GO:0048638 1 4

C0032285 GO:0043954 2 4

C0032285 GO:0031970 1 4

C0032285 GO:0032846 1 4

C0032285 GO:0006477 1 4

C0032285 GO:0009299 1 4

C0032285 GO:0009164 1 4

C0032285 GO:0050927 1 4

C0032285 GO:0042558 1 4

C0032285 GO:0008375 1 4

C0032285 GO:0046631 1 4

C0032285 GO:0051897 1 4

C0032285 GO:0016860 1 4

C0032285 GO:0015085 2 4

C0032285 GO:0015082 1 4

C0032285 GO:0002263 1 4

C0032285 GO:0005544 1 4

C0032285 GO:0006266 1 4

C0032285 GO:0045833 1 4

C0032285 GO:0043120 1 4

C0032285 GO:0060541 1 4

C0032285 GO:0070228 1 4

C0032285 GO:0051055 1 4

C0032285 GO:0015718 1 4

C0032285 GO:0004029 1 4

C0032285 GO:0030216 1 4

C0032285 GO:0051059 1 4

C0032285 GO:0007176 1 4

C0032285 GO:0046330 1 4

C0032285 GO:0005788 1 4

C0032285 GO:0010874 1 4

C0032285 GO:0010875 1 4

C0032285 GO:0016812 1 4

C0032285 GO:0002274 1 4

C0032285 GO:0080010 1 4

C0032285 GO:0051353 1 4

C0032285 GO:0001910 1 4

C0032285 GO:0045821 1 4

C0032285 GO:0005388 2 4

C0032285 GO:0060416 1 4

C0032285 GO:0045494 2 4

C0032285 GO:0007635 1 4

C0032285 GO:0042398 1 4

C0032285 GO:0051103 1 4

C0032285 GO:0019883 1 4

C0032285 GO:0048002 1 4

C0032285 GO:0055102 1 4

C0032285 GO:0021700 1 4

C0032285 GO:0016863 1 4

C0032285 GO:0006563 1 4

C0032285 GO:0002285 2 4

C0032285 GO:0006885 2 4

C0032285 GO:0008088 2 4

C0032285 GO:0008643 1 4

C0032285 GO:0002366 1 4

C0032285 GO:0045912 1 4

C0032285 GO:0005520 1 4

C0032285 GO:0005254 1 4

C0032285 GO:0019882 1 4

C0032285 GO:0004675 1 4

C0032285 GO:0050810 1 4

C0032285 GO:0005930 1 4

C0032285 GO:0045165 1 4

C0032285 GO:0042734 2 4

C0032285 GO:0008417 1 4

C0032285 GO:0017080 1 4

C0032285 GO:0045730 1 4

C0032285 GO:0005247 1 4

C0032285 GO:0060192 1 4

C0032285 GO:0008021 1 4

C0032285 GO:0008028 1 4

C0032285 GO:0017022 1 4

C0032285 GO:0045429 1 4

C0032285 GO:0004859 1 4

C0032285 GO:0051453 2 4

C0032285 GO:0033280 1 4

C0032285 GO:0009124 1 4

C0032285 GO:0030546 1 4

C0032285 GO:0032376 1 4

C0032285 GO:0032370 1 4

C0032285 GO:0032373 1 4

C0032285 GO:0002286 2 4

C0032285 GO:0002224 1 4

C0032285 GO:0008329 1 4

C0032285 GO:0030004 1 4

C0032285 GO:0001654 1 4

C0032285 GO:0010677 1 4

C0032285 GO:0005593 1 4

C0032285 GO:0005024 1 4

C0032285 GO:0017144 1 4

C0032285 GO:0019200 1 4

C0032285 GO:0009187 1 4

C0032285 GO:0030324 1 4

C0032285 GO:0045927 1 4

C0032285 GO:0042119 1 4

C0032285 GO:0016338 1 4

C0032285 GO:0042058 1 4

C0032285 GO:0050926 1 4

C0032285 GO:0046890 1 4

C0032285 GO:0043507 1 4

C0032285 GO:0006090 1 4

C0032285 GO:0030641 2 4

C0032285 GO:0015758 1 4

C0032285 GO:0015695 1 4

C0032285 GO:0005758 1 4

C0032285 GO:0010469 1 4

C0032285 GO:0048662 1 4

C0032285 GO:0048660 1 4

C0032285 GO:0042100 1 4

C0032285 GO:0042102 1 4

C0032285 GO:0009110 1 4

C0032285 GO:0055067 1 4

C0032285 GO:0043627 1 4

C0032285 GO:0016049 1 4

C0032285 GO:0001530 1 4

C0032285 GO:0010595 1 4

C0032285 GO:0010594 1 4

C0032285 GO:0043536 1 4

C0032285 GO:0051923 1 4

C0032285 GO:0043535 1 4

C0032285 GO:0015749 1 4

C0032285 GO:0008645 1 4

C0032285 GO:0006413 1 4

C0038454 GO:0060192 1 1

C0038454 GO:0008028 1 1

C0038454 GO:0031970 1 1

C0038454 GO:0032846 1 1

C0038454 GO:0017022 1 1

C0038454 GO:0009299 1 1

C0038454 GO:0004859 1 1

C0038454 GO:0008088 1 1

C0038454 GO:0046890 1 1

C0038454 GO:0033280 1 1

C0038454 GO:0055102 1 1

C0038454 GO:0021700 1 1

C0038454 GO:0015085 1 1

C0038454 GO:0006563 1 1

C0038454 GO:0002285 1 1

C0038454 GO:0030546 1 1

C0038454 GO:0005544 1 1

C0038454 GO:0042558 1 1

C0038454 GO:0045833 1 1

C0038454 GO:0005758 1 1

C0038454 GO:0005520 1 1

C0038454 GO:0051055 1 1

C0038454 GO:0015718 1 1

C0038454 GO:0042100 1 1

C0038454 GO:0046330 1 1

C0038454 GO:0002286 1 1

C0038454 GO:0005930 1 1

C0038454 GO:0042734 1 1

C0038454 GO:0080010 1 1

C0038454 GO:0008417 1 1

C0038454 GO:0005388 1 1

C0038454 GO:0060416 1 1

C0039070 GO:0051044 1 6

C0039070 GO:0035065 1 6

C0039070 GO:0043954 1 6

C0039070 GO:0031970 1 6

C0039070 GO:0032846 1 6

C0039070 GO:0006477 1 6

C0039070 GO:0009299 1 6

C0039070 GO:0050926 1 6

C0039070 GO:0050927 1 6

C0039070 GO:0042558 1 6

C0039070 GO:0046631 1 6

C0039070 GO:0051897 1 6

C0039070 GO:0030228 1 6

C0039070 GO:0015085 2 6

C0039070 GO:0015082 1 6

C0039070 GO:0002263 1 6

C0039070 GO:0005544 1 6

C0039070 GO:0006266 1 6

C0039070 GO:0045833 1 6

C0039070 GO:0015695 1 6

C0039070 GO:0043120 1 6

C0039070 GO:0060541 2 6

C0039070 GO:0070228 1 6

C0039070 GO:0051055 1 6

C0039070 GO:0032655 1 6

C0039070 GO:0015718 1 6

C0039070 GO:0004029 1 6

C0039070 GO:0030216 1 6

C0039070 GO:0007176 1 6

C0039070 GO:0046330 1 6

C0039070 GO:0005788 1 6

C0039070 GO:0030041 1 6

C0039070 GO:0030295 1 6

C0039070 GO:0010874 1 6

C0039070 GO:0010875 1 6

C0039070 GO:0016812 1 6

C0039070 GO:0002274 1 6

C0039070 GO:0080010 1 6

C0039070 GO:0016814 1 6

C0039070 GO:0051353 1 6

C0039070 GO:0001910 1 6

C0039070 GO:0046849 1 6

C0039070 GO:0005388 2 6

C0039070 GO:0060416 1 6

C0039070 GO:0045494 1 6

C0039070 GO:0005089 1 6

C0039070 GO:0005088 1 6

C0039070 GO:0060263 1 6

C0039070 GO:0007635 1 6

C0039070 GO:0009898 1 6

C0039070 GO:0005086 1 6

C0039070 GO:0032946 1 6

C0039070 GO:0000578 1 6

C0039070 GO:0005929 1 6

C0039070 GO:0048002 1 6

C0039070 GO:0055102 1 6

C0039070 GO:0021700 1 6

C0039070 GO:0016863 1 6

C0039070 GO:0003205 1 6

C0039070 GO:0003206 1 6

C0039070 GO:0006563 1 6

C0039070 GO:0002285 2 6

C0039070 GO:0006885 1 6

C0039070 GO:0008088 2 6

C0039070 GO:0002366 1 6

C0039070 GO:0006112 1 6

C0039070 GO:0006687 1 6

C0039070 GO:0045912 2 6

C0039070 GO:0005520 1 6

C0039070 GO:0016860 1 6

C0039070 GO:0005254 1 6

C0039070 GO:0048705 1 6

C0039070 GO:0019882 1 6

C0039070 GO:0019883 1 6

C0039070 GO:0048709 1 6

C0039070 GO:0008154 1 6

C0039070 GO:0050810 1 6

C0039070 GO:0043256 1 6

C0039070 GO:0005930 1 6

C0039070 GO:0045165 1 6

C0039070 GO:0042734 2 6

C0039070 GO:0008417 1 6

C0039070 GO:0070567 1 6

C0039070 GO:0045730 1 6

C0039070 GO:0005247 1 6

C0039070 GO:0042834 1 6

C0039070 GO:0060192 1 6

C0039070 GO:0048638 1 6

C0039070 GO:0008028 1 6

C0039070 GO:0017022 1 6

C0039070 GO:0014003 1 6

C0039070 GO:0045429 1 6

C0039070 GO:0004859 1 6

C0039070 GO:0048029 1 6

C0039070 GO:0051453 1 6

C0039070 GO:0033280 1 6

C0039070 GO:0051457 1 6

C0039070 GO:0009124 1 6

C0039070 GO:0050885 1 6

C0039070 GO:0030546 1 6

C0039070 GO:0032376 1 6

C0039070 GO:0032370 1 6

C0039070 GO:0032373 1 6

C0039070 GO:0002224 1 6

C0039070 GO:0005506 1 6

C0039070 GO:0015296 1 6

C0039070 GO:0008329 1 6

C0039070 GO:0046326 1 6

C0039070 GO:0030004 1 6

C0039070 GO:0001654 1 6

C0039070 GO:0051103 1 6

C0039070 GO:0010677 2 6

C0039070 GO:0005593 1 6

C0039070 GO:0060021 1 6

C0039070 GO:0050679 1 6

C0039070 GO:0050671 1 6

C0039070 GO:0017144 1 6

C0039070 GO:0019200 1 6

C0039070 GO:0030323 1 6

C0039070 GO:0030324 2 6

C0039070 GO:0019209 1 6

C0039070 GO:0042058 1 6

C0039070 GO:0008210 1 6

C0039070 GO:0046890 1 6

C0039070 GO:0010828 1 6

C0039070 GO:0030641 1 6

C0039070 GO:0009068 1 6

C0039070 GO:0004181 1 6

C0039070 GO:0045766 1 6

C0039070 GO:0070665 1 6

C0039070 GO:0005758 1 6

C0039070 GO:0010469 1 6

C0039070 GO:0006700 1 6

C0039070 GO:0048662 1 6

C0039070 GO:0048660 1 6

C0039070 GO:0042100 1 6

C0039070 GO:0042102 1 6

C0039070 GO:0055067 1 6

C0039070 GO:0043627 1 6

C0039070 GO:0002286 2 6

C0039070 GO:0001530 1 6

C0039070 GO:0043190 1 6

C0039070 GO:0010595 1 6

C0039070 GO:0010594 1 6

C0039070 GO:0042345 1 6

C0039070 GO:0043536 1 6

C0039070 GO:0016338 1 6

C0039070 GO:0051923 1 6

C0039070 GO:0043535 1 6

C0039070 GO:0006413 1 6

C0039070 GO:0015036 1 6

C0042571 GO:0006633 1 9

C0042571 GO:0051043 1 9

C0042571 GO:0009593 1 9

C0042571 GO:0035065 1 9

C0042571 GO:0032434 1 9

C0042571 GO:0046637 1 9

C0042571 GO:0043954 2 9

C0042571 GO:0031970 1 9

C0042571 GO:0046634 1 9

C0042571 GO:0019228 1 9

C0042571 GO:0032846 1 9

C0042571 GO:0009299 1 9

C0042571 GO:0009164 1 9

C0042571 GO:0043524 1 9

C0042571 GO:0042558 1 9

C0042571 GO:0051702 1 9

C0042571 GO:0008375 1 9

C0042571 GO:0045981 1 9

C0042571 GO:0002709 1 9

C0042571 GO:0002708 1 9

C0042571 GO:0015085 2 9

C0042571 GO:0002705 1 9

C0042571 GO:0015082 1 9

C0042571 GO:0005540 1 9

C0042571 GO:0005544 1 9

C0042571 GO:0007217 1 9

C0042571 GO:0004716 1 9

C0042571 GO:0006266 1 9

C0042571 GO:0045833 1 9

C0042571 GO:0004712 1 9

C0042571 GO:0014020 1 9

C0042571 GO:0006942 1 9

C0042571 GO:0046870 1 9

C0042571 GO:0030858 1 9

C0042571 GO:0043120 1 9

C0042571 GO:0015002 1 9

C0042571 GO:0033032 1 9

C0042571 GO:0060541 2 9

C0042571 GO:0016202 1 9

C0042571 GO:0051055 2 9

C0042571 GO:0032655 1 9

C0042571 GO:0015718 1 9

C0042571 GO:0004029 1 9

C0042571 GO:0030216 1 9

C0042571 GO:0006688 1 9

C0042571 GO:0001841 1 9

C0042571 GO:0006769 1 9

C0042571 GO:0051059 1 9

C0042571 GO:0007281 1 9

C0042571 GO:0007176 1 9

C0042571 GO:0046330 1 9

C0042571 GO:0008366 1 9

C0042571 GO:0016894 1 9

C0042571 GO:0030041 1 9

C0042571 GO:0030291 1 9

C0042571 GO:0030295 1 9

C0042571 GO:0045580 2 9

C0042571 GO:0042326 1 9

C0042571 GO:0055092 1 9

C0042571 GO:0015145 1 9

C0042571 GO:0002274 1 9

C0042571 GO:0080010 1 9

C0042571 GO:0016814 1 9

C0042571 GO:0016566 1 9

C0042571 GO:0001910 1 9

C0042571 GO:0046849 1 9

C0042571 GO:0045821 1 9

C0042571 GO:0007043 1 9

C0042571 GO:0005388 2 9

C0042571 GO:0060416 2 9

C0042571 GO:0045494 2 9

C0042571 GO:0005089 1 9

C0042571 GO:0005088 1 9

C0042571 GO:0060263 1 9

C0042571 GO:0032410 1 9

C0042571 GO:0007635 1 9

C0042571 GO:0032412 1 9

C0042571 GO:0001843 1 9

C0042571 GO:0042398 1 9

C0042571 GO:0006458 1 9

C0042571 GO:0009898 1 9

C0042571 GO:0005086 1 9

C0042571 GO:0001763 1 9

C0042571 GO:0032946 1 9

C0042571 GO:0046496 1 9

C0042571 GO:0000578 1 9

C0042571 GO:0005929 1 9

C0042571 GO:0032496 1 9

C0042571 GO:0055102 1 9

C0042571 GO:0021700 1 9

C0042571 GO:0030178 1 9

C0042571 GO:0007272 1 9

C0042571 GO:0003205 1 9

C0042571 GO:0003206 1 9

C0042571 GO:0006563 1 9

C0042571 GO:0050709 1 9

C0042571 GO:0002285 1 9

C0042571 GO:0016045 1 9

C0042571 GO:0006885 4 9

C0042571 GO:0050700 1 9

C0042571 GO:0008088 3 9

C0042571 GO:0008643 1 9

C0042571 GO:0032862 1 9

C0042571 GO:0006112 1 9

C0042571 GO:0006687 1 9

C0042571 GO:0019218 1 9

C0042571 GO:0005522 1 9

C0042571 GO:0045912 1 9

C0042571 GO:0005520 2 9

C0042571 GO:0031346 1 9

C0042571 GO:0005254 1 9

C0042571 GO:0030879 1 9

C0042571 GO:0048705 1 9

C0042571 GO:0030149 1 9

C0042571 GO:0015833 1 9

C0042571 GO:0060260 1 9

C0042571 GO:0048709 1 9

C0042571 GO:0031985 1 9

C0042571 GO:0070167 1 9

C0042571 GO:0008154 1 9

C0042571 GO:0050810 1 9

C0042571 GO:0016877 1 9

C0042571 GO:0043370 1 9

C0042571 GO:0043256 1 9

C0042571 GO:0042632 1 9

C0042571 GO:0032489 1 9

C0042571 GO:0005930 1 9

C0042571 GO:0060606 1 9

C0042571 GO:0034765 1 9

C0042571 GO:0042734 3 9

C0042571 GO:0002711 1 9

C0042571 GO:0008417 1 9

C0042571 GO:0004708 1 9

C0042571 GO:0008206 1 9

C0042571 GO:0070567 1 9

C0042571 GO:0017080 1 9

C0042571 GO:0045730 1 9

C0042571 GO:0016676 1 9

C0042571 GO:0043034 1 9

C0042571 GO:0016675 1 9

C0042571 GO:0005247 1 9

C0042571 GO:0006752 1 9

C0042571 GO:0002699 1 9

C0042571 GO:0042834 1 9

C0042571 GO:0060192 1 9

C0042571 GO:0008021 1 9

C0042571 GO:0006637 1 9

C0042571 GO:0008028 1 9

C0042571 GO:0007519 1 9

C0042571 GO:0048145 1 9

C0042571 GO:0009311 1 9

C0042571 GO:0048634 1 9

C0042571 GO:0017022 1 9

C0042571 GO:0035148 1 9

C0042571 GO:0006941 1 9

C0042571 GO:0014003 1 9

C0042571 GO:0031228 1 9

C0042571 GO:0009187 1 9

C0042571 GO:0004859 1 9

C0042571 GO:0048029 1 9

C0042571 GO:0051453 2 9

C0042571 GO:0033280 1 9

C0042571 GO:0004690 1 9

C0042571 GO:0051457 1 9

C0042571 GO:0035295 1 9

C0042571 GO:0006220 1 9

C0042571 GO:0043112 1 9

C0042571 GO:0050885 1 9

C0042571 GO:0005583 1 9

C0042571 GO:0030546 1 9

C0042571 GO:0030810 1 9

C0042571 GO:0046887 1 9

C0042571 GO:0007162 1 9

C0042571 GO:0016459 1 9

C0042571 GO:0015166 1 9

C0042571 GO:0002224 1 9

C0042571 GO:0060562 1 9

C0042571 GO:0005506 1 9

C0042571 GO:0045622 1 9

C0042571 GO:0005501 1 9

C0042571 GO:0022614 1 9

C0042571 GO:0034599 1 9

C0042571 GO:0008286 1 9

C0042571 GO:0015296 1 9

C0042571 GO:0030317 1 9

C0042571 GO:0046326 2 9

C0042571 GO:0046466 1 9

C0042571 GO:0030004 1 9

C0042571 GO:0005796 1 9

C0042571 GO:0046883 1 9

C0042571 GO:0004864 1 9

C0042571 GO:0051103 1 9

C0042571 GO:0045216 1 9

C0042571 GO:0010677 1 9

C0042571 GO:0033293 1 9

C0042571 GO:0060021 2 9

C0042571 GO:0016903 1 9

C0042571 GO:0008093 1 9

C0042571 GO:0005507 1 9

C0042571 GO:0050679 1 9

C0042571 GO:0030804 1 9

C0042571 GO:0042542 1 9

C0042571 GO:0030801 1 9

C0042571 GO:0050671 1 9

C0042571 GO:0005024 1 9

C0042571 GO:0045940 1 9

C0042571 GO:0016528 1 9

C0042571 GO:0016529 1 9

C0042571 GO:0002237 1 9

C0042571 GO:0045619 1 9

C0042571 GO:0043088 1 9

C0042571 GO:0006282 1 9

C0042571 GO:0045616 1 9

C0042571 GO:0017144 1 9

C0042571 GO:0000185 1 9

C0042571 GO:0019200 1 9

C0042571 GO:0030323 1 9

C0042571 GO:0030324 2 9

C0042571 GO:0045927 1 9

C0042571 GO:0042119 1 9

C0042571 GO:0019840 1 9

C0042571 GO:0019209 1 9

C0042571 GO:0042169 1 9

C0042571 GO:0017048 1 9

C0042571 GO:0042058 1 9

C0042571 GO:0043506 1 9

C0042571 GO:0008210 1 9

C0042571 GO:0002822 1 9

C0042571 GO:0034623 1 9

C0042571 GO:0046890 2 9

C0042571 GO:0045445 1 9

C0042571 GO:0043507 1 9

C0042571 GO:0004691 1 9

C0042571 GO:0006090 1 9

C0042571 GO:0010828 2 9

C0042571 GO:0030641 2 9

C0042571 GO:0005790 1 9

C0042571 GO:0045768 1 9

C0042571 GO:0009068 1 9

C0042571 GO:0019362 1 9

C0042571 GO:0044447 1 9

C0042571 GO:0004181 1 9

C0042571 GO:0006937 1 9

C0042571 GO:0045766 1 9

C0042571 GO:0045767 1 9

C0042571 GO:0004675 1 9

C0042571 GO:0016538 1 9

C0042571 GO:0032321 1 9

C0042571 GO:0070665 1 9

C0042571 GO:0006244 1 9

C0042571 GO:0045604 1 9

C0042571 GO:0033189 1 9

C0042571 GO:0005758 1 9

C0042571 GO:0006929 1 9

C0042571 GO:0010469 1 9

C0042571 GO:0046716 1 9

C0042571 GO:0042552 1 9

C0042571 GO:0032526 1 9

C0042571 GO:0006700 1 9

C0042571 GO:0030902 1 9

C0042571 GO:0034384 1 9

C0042571 GO:0009247 1 9

C0042571 GO:0007585 1 9

C0042571 GO:0042100 1 9

C0042571 GO:0042102 1 9

C0042571 GO:0015758 1 9

C0042571 GO:0032856 1 9

C0042571 GO:0009110 1 9

C0042571 GO:0055067 2 9

C0042571 GO:0031214 1 9

C0042571 GO:0043627 2 9

C0042571 GO:0002286 1 9

C0042571 GO:0004033 1 9

C0042571 GO:0015149 1 9

C0042571 GO:0045739 1 9

C0042571 GO:0005858 1 9

C0042571 GO:0001838 1 9

C0042571 GO:0043190 1 9

C0042571 GO:0016620 1 9

C0042571 GO:0042345 1 9

C0042571 GO:0004861 1 9

C0042571 GO:0016338 1 9

C0042571 GO:0004129 1 9

C0042571 GO:0019212 1 9

C0042571 GO:0015665 1 9

C0042571 GO:0016049 1 9

C0042571 GO:0015749 1 9

C0042571 GO:0008645 1 9

C0042571 GO:0060538 1 9

C0042571 GO:0050750 1 9

C0042571 GO:0005161 1 9

C0042571 GO:0030166 1 9

C0042571 GO:0015036 1 9

C0086543 GO:0060192 1 1

C0086543 GO:0008028 1 1

C0086543 GO:0031970 1 1

C0086543 GO:0032846 1 1

C0086543 GO:0017022 1 1

C0086543 GO:0009299 1 1

C0086543 GO:0004859 1 1

C0086543 GO:0008088 1 1

C0086543 GO:0046890 1 1

C0086543 GO:0033280 1 1

C0086543 GO:0055102 1 1

C0086543 GO:0021700 1 1

C0086543 GO:0015085 1 1

C0086543 GO:0006563 1 1

C0086543 GO:0002285 1 1

C0086543 GO:0030546 1 1

C0086543 GO:0005544 1 1

C0086543 GO:0042558 1 1

C0086543 GO:0045833 1 1

C0086543 GO:0005758 1 1

C0086543 GO:0005520 1 1

C0086543 GO:0051055 1 1

C0086543 GO:0015718 1 1

C0086543 GO:0042100 1 1

C0086543 GO:0046330 1 1

C0086543 GO:0002286 1 1

C0086543 GO:0005930 1 1

C0086543 GO:0042734 1 1

C0086543 GO:0080010 1 1

C0086543 GO:0008417 1 1

C0086543 GO:0005388 1 1

C0086543 GO:0060416 1 1

C0003862 GO:0051043 1 14

C0003862 GO:0051044 2 14

C0003862 GO:0034358 1 14

C0003862 GO:0043954 2 14

C0003862 GO:0010677 1 14

C0003862 GO:0009164 1 14

C0003862 GO:0001505 1 14

C0003862 GO:0042036 1 14

C0003862 GO:0006909 1 14

C0003862 GO:0048261 1 14

C0003862 GO:0016863 3 14

C0003862 GO:0030228 1 14

C0003862 GO:0006306 2 14

C0003862 GO:0006305 2 14

C0003862 GO:0006304 1 14

C0003862 GO:0000002 1 14

C0003862 GO:0019992 1 14

C0003862 GO:0018210 2 14

C0003862 GO:0004716 1 14

C0003862 GO:0016459 2 14

C0003862 GO:0005795 1 14

C0003862 GO:0046504 1 14

C0003862 GO:0060541 3 14

C0003862 GO:0030595 1 14

C0003862 GO:0045851 1 14

C0003862 GO:0004029 1 14

C0003862 GO:0034311 1 14

C0003862 GO:0019229 1 14

C0003862 GO:0007281 1 14

C0003862 GO:0030299 1 14

C0003862 GO:0008360 1 14

C0003862 GO:0005788 1 14

C0003862 GO:0006836 1 14

C0003862 GO:0030291 1 14

C0003862 GO:0030295 2 14

C0003862 GO:0034284 1 14

C0003862 GO:0016812 1 14

C0003862 GO:0006953 1 14

C0003862 GO:0016811 1 14

C0003862 GO:0070633 1 14

C0003862 GO:0016814 1 14

C0003862 GO:0045821 1 14

C0003862 GO:0003995 1 14

C0003862 GO:0033261 1 14

C0003862 GO:0010888 1 14

C0003862 GO:0021537 1 14

C0003862 GO:0060263 1 14

C0003862 GO:0001953 1 14

C0003862 GO:0009898 1 14

C0003862 GO:0010869 1 14

C0003862 GO:0030170 1 14

C0003862 GO:0000578 1 14

C0003862 GO:0030501 1 14

C0003862 GO:0010862 1 14

C0003862 GO:0051224 1 14

C0003862 GO:0030073 1 14

C0003862 GO:0003205 2 14

C0003862 GO:0003206 2 14

C0003862 GO:0015491 1 14

C0003862 GO:0042551 1 14

C0003862 GO:0006885 3 14

C0003862 GO:0003208 1 14

C0003862 GO:0008643 1 14

C0003862 GO:0043028 2 14

C0003862 GO:0046850 2 14

C0003862 GO:0006112 1 14

C0003862 GO:0006110 1 14

C0003862 GO:0045912 1 14

C0003862 GO:0016860 2 14

C0003862 GO:0051879 1 14

C0003862 GO:0043027 2 14

C0003862 GO:0006303 1 14

C0003862 GO:0048705 1 14

C0003862 GO:0019882 3 14

C0003862 GO:0019883 1 14

C0003862 GO:0007623 1 14

C0003862 GO:0031256 1 14

C0003862 GO:0048709 1 14

C0003862 GO:0070167 1 14

C0003862 GO:0006584 1 14

C0003862 GO:0060606 1 14

C0003862 GO:0043205 1 14

C0003862 GO:0004437 1 14

C0003862 GO:0050716 1 14

C0003862 GO:0050714 2 14

C0003862 GO:0050718 1 14

C0003862 GO:0035097 1 14

C0003862 GO:0006690 1 14

C0003862 GO:0015179 1 14

C0003862 GO:0048407 2 14

C0003862 GO:0006026 1 14

C0003862 GO:0006027 1 14

C0003862 GO:0015172 1 14

C0003862 GO:0008021 1 14

C0003862 GO:0045923 1 14

C0003862 GO:0048145 1 14

C0003862 GO:0048634 1 14

C0003862 GO:0018108 1 14

C0003862 GO:0014003 1 14

C0003862 GO:0009410 1 14

C0003862 GO:0051453 3 14

C0003862 GO:0051452 1 14

C0003862 GO:0048385 1 14

C0003862 GO:0050431 1 14

C0003862 GO:0045078 1 14

C0003862 GO:0043489 1 14

C0003862 GO:0030810 1 14

C0003862 GO:0030705 1 14

C0003862 GO:0033238 1 14

C0003862 GO:0002224 1 14

C0003862 GO:0045622 1 14

C0003862 GO:0002221 1 14

C0003862 GO:0032677 1 14

C0003862 GO:0045732 1 14

C0003862 GO:0006879 1 14

C0003862 GO:0008329 1 14

C0003862 GO:0055008 1 14

C0003862 GO:0015929 1 14

C0003862 GO:0048872 1 14

C0003862 GO:0045211 1 14

C0003862 GO:0001654 2 14

C0003862 GO:0010675 1 14

C0003862 GO:0045216 1 14

C0003862 GO:0046887 1 14

C0003862 GO:0033293 1 14

C0003862 GO:0008656 1 14

C0003862 GO:0032722 1 14

C0003862 GO:0004190 4 14

C0003862 GO:0016525 1 14

C0003862 GO:0017046 1 14

C0003862 GO:0018107 1 14

C0003862 GO:0045927 1 14

C0003862 GO:0015813 1 14

C0003862 GO:0015810 1 14

C0003862 GO:0019048 1 14

C0003862 GO:0030035 1 14

C0003862 GO:0017048 1 14

C0003862 GO:0060021 1 14

C0003862 GO:0042593 1 14

C0003862 GO:0008210 1 14

C0003862 GO:0050906 1 14

C0003862 GO:0050905 1 14

C0003862 GO:0007270 2 14

C0003862 GO:0007271 1 14

C0003862 GO:0006090 1 14

C0003862 GO:0006096 1 14

C0003862 GO:0006094 1 14

C0003862 GO:0050909 1 14

C0003862 GO:0042359 1 14

C0003862 GO:0032350 1 14

C0003862 GO:0042354 1 14

C0003862 GO:0055092 2 14

C0003862 GO:0045639 1 14

C0003862 GO:0070665 1 14

C0003862 GO:0070668 1 14

C0003862 GO:0045736 1 14

C0003862 GO:0006700 1 14

C0003862 GO:0021915 1 14

C0003862 GO:0070120 1 14

C0003862 GO:0048593 1 14

C0003862 GO:0000271 1 14

C0003862 GO:0032856 1 14

C0003862 GO:0004969 1 14

C0003862 GO:0001838 1 14

C0003862 GO:0010595 1 14

C0003862 GO:0010594 3 14

C0003862 GO:0043536 1 14

C0003862 GO:0005044 1 14

C0003862 GO:0050995 1 14

C0003862 GO:0050994 1 14

C0003862 GO:0016986 1 14

C0003862 GO:0050996 1 14

C0003862 GO:0032321 1 14

C0003862 GO:0006413 1 14

C0003862 GO:0032148 1 14

C0003862 GO:0009593 1 14

C0003862 GO:0005149 1 14

C0003862 GO:0007618 1 14

C0003862 GO:0007612 2 14

C0003862 GO:0007611 1 14

C0003862 GO:0007162 1 14

C0003862 GO:0007163 1 14

C0003862 GO:0019915 2 14

C0003862 GO:0002573 1 14

C0003862 GO:0016597 1 14

C0003862 GO:0002263 2 14

C0003862 GO:0045667 1 14

C0003862 GO:0045661 1 14

C0003862 GO:0045669 1 14

C0003862 GO:0033032 1 14

C0003862 GO:0016202 1 14

C0003862 GO:0034364 1 14

C0003862 GO:0030216 1 14

C0003862 GO:0051119 1 14

C0003862 GO:0030218 1 14

C0003862 GO:0051117 1 14

C0003862 GO:0051059 1 14

C0003862 GO:0051058 1 14

C0003862 GO:0048278 1 14

C0003862 GO:0016894 1 14

C0003862 GO:0042994 1 14

C0003862 GO:0042992 2 14

C0003862 GO:0045621 1 14

C0003862 GO:0014020 1 14

C0003862 GO:0046530 1 14

C0003862 GO:0004707 1 14

C0003862 GO:0007044 1 14

C0003862 GO:0016291 1 14

C0003862 GO:0005388 2 14

C0003862 GO:0016445 1 14

C0003862 GO:0005905 1 14

C0003862 GO:0050820 1 14

C0003862 GO:0048002 2 14

C0003862 GO:0045445 1 14

C0003862 GO:0034708 1 14

C0003862 GO:0002761 1 14

C0003862 GO:0051187 1 14

C0003862 GO:0046915 2 14

C0003862 GO:0046425 1 14

C0003862 GO:0008088 2 14

C0003862 GO:0045749 1 14

C0003862 GO:0007416 1 14

C0003862 GO:0016755 1 14

C0003862 GO:0001948 1 14

C0003862 GO:0031941 1 14

C0003862 GO:0030235 1 14

C0003862 GO:0020037 2 14

C0003862 GO:0032768 1 14

C0003862 GO:0050796 1 14

C0003862 GO:0050795 1 14

C0003862 GO:0003231 1 14

C0003862 GO:0046660 1 14

C0003862 GO:0009953 1 14

C0003862 GO:0043666 1 14

C0003862 GO:0034220 1 14

C0003862 GO:0034382 1 14

C0003862 GO:0032364 1 14

C0003862 GO:0032365 1 14

C0003862 GO:0050892 1 14

C0003862 GO:0043154 1 14

C0003862 GO:0017080 2 14

C0003862 GO:0046823 2 14

C0003862 GO:0003746 1 14

C0003862 GO:0043010 1 14

C0003862 GO:0002698 1 14

C0003862 GO:0003785 2 14

C0003862 GO:0006633 1 14

C0003862 GO:0032107 1 14

C0003862 GO:0032104 1 14

C0003862 GO:0019894 1 14

C0003862 GO:0006637 1 14

C0003862 GO:0019956 1 14

C0003862 GO:0009311 1 14

C0003862 GO:0070279 1 14

C0003862 GO:0042401 1 14

C0003862 GO:0009895 2 14

C0003862 GO:0007043 1 14

C0003862 GO:0050766 1 14

C0003862 GO:0050764 1 14

C0003862 GO:0030201 1 14

C0003862 GO:0005035 1 14

C0003862 GO:0005031 1 14

C0003862 GO:0048568 1 14

C0003862 GO:0005355 1 14

C0003862 GO:0042509 1 14

C0003862 GO:0008034 1 14

C0003862 GO:0070303 1 14

C0003862 GO:0006929 3 14

C0003862 GO:0005868 1 14

C0003862 GO:0032587 1 14

C0003862 GO:0010906 1 14

C0003862 GO:0043470 1 14

C0003862 GO:0043471 1 14

C0003862 GO:0030804 1 14

C0003862 GO:0030175 1 14

C0003862 GO:0010596 1 14

C0003862 GO:0030801 1 14

C0003862 GO:0045072 1 14

C0003862 GO:0002237 1 14

C0003862 GO:0045619 2 14

C0003862 GO:0034405 2 14

C0003862 GO:0045616 1 14

C0003862 GO:0005518 1 14

C0003862 GO:0009083 1 14

C0003862 GO:0016331 1 14

C0003862 GO:0030323 2 14

C0003862 GO:0030324 3 14

C0003862 GO:0050868 1 14

C0003862 GO:0055010 1 14

C0003862 GO:0001843 1 14

C0003862 GO:0050866 2 14

C0003862 GO:0019842 1 14

C0003862 GO:0051147 1 14

C0003862 GO:0048041 1 14

C0003862 GO:0051149 1 14

C0003862 GO:0001523 1 14

C0003862 GO:0031532 2 14

C0003862 GO:0004181 1 14

C0003862 GO:0004675 2 14

C0003862 GO:0016538 1 14

C0003862 GO:0042787 1 14

C0003862 GO:0030934 1 14

C0003862 GO:0060415 1 14

C0003862 GO:0043588 1 14

C0003862 GO:0045931 1 14

C0003862 GO:0045930 1 14

C0003862 GO:0050699 1 14

C0003862 GO:0010812 1 14

C0003862 GO:0046626 1 14

C0003862 GO:0055062 1 14

C0003862 GO:0055061 1 14

C0003862 GO:0055067 1 14

C0003862 GO:0007269 1 14

C0003862 GO:0016505 1 14

C0003862 GO:0043627 2 14

C0003862 GO:0007266 1 14

C0003862 GO:0035295 1 14

C0003862 GO:0009746 1 14

C0003862 GO:0043190 1 14

C0003862 GO:0005605 1 14

C0003862 GO:0042345 1 14

C0003862 GO:0032205 1 14

C0003862 GO:0002793 1 14

C0003862 GO:0051923 1 14

C0003862 GO:0003705 1 14

C0003862 GO:0009749 1 14

C0003862 GO:0055072 1 14

C0003862 GO:0008645 1 14

C0003862 GO:0060538 3 14

C0003862 GO:0010832 1 14

C0003862 GO:0046697 1 14

C0003862 GO:0031970 1 14

C0003862 GO:0032387 2 14

C0003862 GO:0021510 1 14

C0003862 GO:0006477 1 14

C0003862 GO:0006476 1 14

C0003862 GO:0055081 1 14

C0003862 GO:0031058 1 14

C0003862 GO:0045981 1 14

C0003862 GO:0015085 1 14

C0003862 GO:0048864 1 14

C0003862 GO:0015082 2 14

C0003862 GO:0042531 1 14

C0003862 GO:0055085 1 14

C0003862 GO:0070001 4 14

C0003862 GO:0016101 1 14

C0003862 GO:0043120 2 14

C0003862 GO:0070228 2 14

C0003862 GO:0035272 1 14

C0003862 GO:0032655 2 14

C0003862 GO:0032652 1 14

C0003862 GO:0005159 1 14

C0003862 GO:0005158 1 14

C0003862 GO:0042129 1 14

C0003862 GO:0003007 1 14

C0003862 GO:0007176 1 14

C0003862 GO:0043535 3 14

C0003862 GO:0008585 1 14

C0003862 GO:0003001 1 14

C0003862 GO:0005523 1 14

C0003862 GO:0007598 1 14

C0003862 GO:0048732 1 14

C0003862 GO:0043025 1 14

C0003862 GO:0002274 1 14

C0003862 GO:0060393 2 14

C0003862 GO:0002377 2 14

C0003862 GO:0001910 1 14

C0003862 GO:0004550 1 14

C0003862 GO:0004551 1 14

C0003862 GO:0045494 2 14

C0003862 GO:0030203 1 14

C0003862 GO:0046006 1 14

C0003862 GO:0042398 1 14

C0003862 GO:0051103 1 14

C0003862 GO:0070169 1 14

C0003862 GO:0070207 1 14

C0003862 GO:0009068 1 14

C0003862 GO:0042625 1 14

C0003862 GO:0042627 1 14

C0003862 GO:0090100 1 14

C0003862 GO:0043548 2 14

C0003862 GO:0031674 1 14

C0003862 GO:0005522 1 14

C0003862 GO:0048641 1 14

C0003862 GO:0002200 1 14

C0003862 GO:0045727 1 14

C0003862 GO:0005254 1 14

C0003862 GO:0030879 1 14

C0003862 GO:0001776 1 14

C0003862 GO:0043900 1 14

C0003862 GO:0050810 1 14

C0003862 GO:0042308 2 14

C0003862 GO:0030279 1 14

C0003862 GO:0050819 2 14

C0003862 GO:0044275 2 14

C0003862 GO:0043410 1 14

C0003862 GO:0002757 1 14

C0003862 GO:0008091 2 14

C0003862 GO:0008093 1 14

C0003862 GO:0015300 1 14

C0003862 GO:0002758 1 14

C0003862 GO:0044447 1 14

C0003862 GO:0006739 1 14

C0003862 GO:0032770 1 14

C0003862 GO:0015280 1 14

C0003862 GO:0010921 2 14

C0003862 GO:0031016 1 14

C0003862 GO:0017022 1 14

C0003862 GO:0045580 1 14

C0003862 GO:0045429 2 14

C0003862 GO:0045428 1 14

C0003862 GO:0046677 2 14

C0003862 GO:0003229 1 14

C0003862 GO:0002526 1 14

C0003862 GO:0008543 1 14

C0003862 GO:0030018 1 14

C0003862 GO:0051324 1 14

C0003862 GO:0050885 3 14

C0003862 GO:0042274 2 14

C0003862 GO:0032374 1 14

C0003862 GO:0032376 3 14

C0003862 GO:0032371 1 14

C0003862 GO:0032370 3 14

C0003862 GO:0032373 3 14

C0003862 GO:0015377 1 14

C0003862 GO:0060562 1 14

C0003862 GO:0016338 2 14

C0003862 GO:0031330 2 14

C0003862 GO:0009247 1 14

C0003862 GO:0004861 1 14

C0003862 GO:0005593 2 14

C0003862 GO:0000217 2 14

C0003862 GO:0050777 1 14

C0003862 GO:0050679 2 14

C0003862 GO:0007422 1 14

C0003862 GO:0050670 1 14

C0003862 GO:0050671 2 14

C0003862 GO:0005024 2 14

C0003862 GO:0001816 1 14

C0003862 GO:0022839 1 14

C0003862 GO:0032421 1 14

C0003862 GO:0009187 1 14

C0003862 GO:0042119 2 14

C0003862 GO:0016830 1 14

C0003862 GO:0005160 2 14

C0003862 GO:0005161 3 14

C0003862 GO:0042058 1 14

C0003862 GO:0005436 1 14

C0003862 GO:0043506 2 14

C0003862 GO:0043507 1 14

C0003862 GO:0006109 1 14

C0003862 GO:0043500 1 14

C0003862 GO:0045768 1 14

C0003862 GO:0030832 1 14

C0003862 GO:0034366 1 14

C0003862 GO:0070567 1 14

C0003862 GO:0045766 2 14

C0003862 GO:0045767 2 14

C0003862 GO:0015758 1 14

C0003862 GO:0015695 1 14

C0003862 GO:0004879 1 14

C0003862 GO:0045604 1 14

C0003862 GO:0043531 1 14

C0003862 GO:0030330 1 14

C0003862 GO:0007585 1 14

C0003862 GO:0008198 1 14

C0003862 GO:0009112 1 14

C0003862 GO:0009110 1 14

C0003862 GO:0046580 1 14

C0003862 GO:0051646 1 14

C0003862 GO:0034599 1 14

C0003862 GO:0001530 1 14

C0003862 GO:0045123 1 14

C0003862 GO:0045124 2 14

C0003862 GO:0030900 1 14

C0003862 GO:0004890 1 14

C0003862 GO:0009712 1 14

C0003862 GO:0007090 1 14

C0003862 GO:0035065 1 14

C0003862 GO:0048638 1 14

C0003862 GO:0046637 1 14

C0003862 GO:0046634 1 14

C0003862 GO:0046631 2 14

C0003862 GO:0033500 1 14

C0003862 GO:0007292 1 14

C0003862 GO:0050926 1 14

C0003862 GO:0050927 1 14

C0003862 GO:0050920 1 14

C0003862 GO:0030345 1 14

C0003862 GO:0051893 1 14

C0003862 GO:0008375 1 14

C0003862 GO:0051897 2 14

C0003862 GO:0051701 1 14

C0003862 GO:0007519 3 14

C0003862 GO:0044419 1 14

C0003862 GO:0006266 1 14

C0003862 GO:0043325 1 14

C0003862 GO:0045834 1 14

C0003862 GO:0010896 2 14

C0003862 GO:0010898 2 14

C0003862 GO:0010875 3 14

C0003862 GO:0032393 1 14

C0003862 GO:0032420 1 14

C0003862 GO:0005796 1 14

C0003862 GO:0019827 1 14

C0003862 GO:0032781 1 14

C0003862 GO:0030048 1 14

C0003862 GO:0043178 1 14

C0003862 GO:0030041 1 14

C0003862 GO:0010874 2 14

C0003862 GO:0030169 2 14

C0003862 GO:0043648 1 14

C0003862 GO:0015485 1 14

C0003862 GO:0006040 1 14

C0003862 GO:0001822 1 14

C0003862 GO:0051353 2 14

C0003862 GO:0030199 1 14

C0003862 GO:0030198 1 14

C0003862 GO:0046849 1 14

C0003862 GO:0032731 1 14

C0003862 GO:0030195 2 14

C0003862 GO:0030194 1 14

C0003862 GO:0005089 2 14

C0003862 GO:0005088 2 14

C0003862 GO:0007632 2 14

C0003862 GO:0007635 1 14

C0003862 GO:0032642 1 14

C0003862 GO:0035023 1 14

C0003862 GO:0005086 1 14

C0003862 GO:0019934 1 14

C0003862 GO:0004693 1 14

C0003862 GO:0044403 1 14

C0003862 GO:0032946 1 14

C0003862 GO:0048488 1 14

C0003862 GO:0002286 2 14

C0003862 GO:0002285 2 14

C0003862 GO:0051457 1 14

C0003862 GO:0030183 1 14

C0003862 GO:0050700 2 14

C0003862 GO:0002366 2 14

C0003862 GO:0006687 3 14

C0003862 GO:0001754 1 14

C0003862 GO:0001841 1 14

C0003862 GO:0006688 1 14

C0003862 GO:0048477 1 14

C0003862 GO:0046545 1 14

C0003862 GO:0030890 1 14

C0003862 GO:0008154 1 14

C0003862 GO:0050690 1 14

C0003862 GO:0048255 1 14

C0003862 GO:0042476 1 14

C0003862 GO:0043256 2 14

C0003862 GO:0042632 2 14

C0003862 GO:0042102 2 14

C0003862 GO:0045165 1 14

C0003862 GO:0042734 2 14

C0003862 GO:0004683 1 14

C0003862 GO:0031645 1 14

C0003862 GO:0030864 1 14

C0003862 GO:0002218 1 14

C0003862 GO:0045638 1 14

C0003862 GO:0045730 3 14

C0003862 GO:0043034 2 14

C0003862 GO:0005247 1 14

C0003862 GO:0015662 1 14

C0003862 GO:0007158 1 14

C0003862 GO:0050808 2 14

C0003862 GO:0042752 1 14

C0003862 GO:0004114 1 14

C0003862 GO:0004112 1 14

C0003862 GO:0035148 1 14

C0003862 GO:0006941 1 14

C0003862 GO:0005903 1 14

C0003862 GO:0048029 2 14

C0003862 GO:0016909 1 14

C0003862 GO:0044241 1 14

C0003862 GO:0009124 1 14

C0003862 GO:0001540 1 14

C0003862 GO:0006342 1 14

C0003862 GO:0000041 1 14

C0003862 GO:0046782 1 14

C0003862 GO:0015297 1 14

C0003862 GO:0015296 1 14

C0003862 GO:0015299 2 14

C0003862 GO:0015298 2 14

C0003862 GO:0030004 1 14

C0003862 GO:0015807 4 14

C0003862 GO:0015800 1 14

C0003862 GO:0010830 1 14

C0003862 GO:0018958 1 14

C0003862 GO:0001894 1 14

C0003862 GO:0001893 1 14

C0003862 GO:0045598 1 14

C0003862 GO:0007568 1 14

C0003862 GO:0006081 1 14

C0003862 GO:0006805 1 14

C0003862 GO:0046329 1 14

C0003862 GO:0045778 1 14

C0003862 GO:0045296 2 14

C0003862 GO:0045862 1 14

C0003862 GO:0006821 1 14

C0003862 GO:0030099 1 14

C0003862 GO:0043370 1 14

C0003862 GO:0045861 1 14

C0003862 GO:0017144 1 14

C0003862 GO:0019200 2 14

C0003862 GO:0019201 1 14

C0003862 GO:0030021 1 14

C0003862 GO:0014910 1 14

C0003862 GO:0045582 1 14

C0003862 GO:0019209 2 14

C0003862 GO:0000188 1 14

C0003862 GO:0050829 1 14

C0003862 GO:0032862 1 14

C0003862 GO:0046112 1 14

C0003862 GO:0044236 1 14

C0003862 GO:0060228 1 14

C0003862 GO:0030641 3 14

C0003862 GO:0045684 1 14

C0003862 GO:0070555 1 14

C0003862 GO:0042516 1 14

C0003862 GO:0042517 1 14

C0003862 GO:0006721 1 14

C0003862 GO:0010466 1 14

C0003862 GO:0070411 1 14

C0003862 GO:0010745 1 14

C0003862 GO:0010469 1 14

C0003862 GO:0010741 1 14

C0003862 GO:0010743 1 14

C0003862 GO:0034101 1 14

C0003862 GO:0034103 2 14

C0003862 GO:0048662 1 14

C0003862 GO:0048660 1 14

C0003862 GO:0048741 1 14

C0003862 GO:0042104 1 14

C0003862 GO:0048742 1 14

C0003862 GO:0042100 1 14

C0003862 GO:0033483 1 14

C0003862 GO:0043523 1 14

C0003862 GO:0002449 1 14

C0003862 GO:0005884 1 14

C0003862 GO:0031214 1 14

C0003862 GO:0002440 2 14

C0003862 GO:0016049 1 14

C0003862 GO:0045807 1 14

C0003862 GO:0032994 1 14

C0003862 GO:0034637 1 14

C0003862 GO:0033344 1 14

C0003862 GO:0002250 1 14

C0003862 GO:0015749 1 14

C0003862 GO:0006970 1 14

C0003862 GO:0001937 1 14

C0003862 GO:0015036 1 14

C0003864 GO:0032107 1 2

C0003864 GO:0019200 1 2

C0003864 GO:0030324 1 2

C0003864 GO:0007635 1 2

C0003864 GO:0030299 1 2

C0003864 GO:0043954 1 2

C0003864 GO:0050866 1 2

C0003864 GO:0032104 1 2

C0003864 GO:0051103 1 2

C0003864 GO:0042058 1 2

C0003864 GO:0048002 1 2

C0003864 GO:0030801 1 2

C0003864 GO:0001505 1 2

C0003864 GO:0045296 1 2

C0003864 GO:0051453 1 2

C0003864 GO:0044241 1 2

C0003864 GO:0046631 1 2

C0003864 GO:0045981 1 2

C0003864 GO:0030641 1 2

C0003864 GO:0015297 1 2

C0003864 GO:0015085 1 2

C0003864 GO:0030810 1 2

C0003864 GO:0002286 1 2

C0003864 GO:0015082 1 2

C0003864 GO:0002285 1 2

C0003864 GO:0031532 1 2

C0003864 GO:0006885 1 2

C0003864 GO:0002263 1 2

C0003864 GO:0008088 1 2

C0003864 GO:0070001 1 2

C0003864 GO:0006266 1 2

C0003864 GO:0001910 1 2

C0003864 GO:0006687 1 2

C0003864 GO:0002224 1 2

C0003864 GO:0005254 1 2

C0003864 GO:0002221 1 2

C0003864 GO:0015491 1 2

C0003864 GO:0060541 1 2

C0003864 GO:0019882 1 2

C0003864 GO:0004029 1 2

C0003864 GO:0030216 1 2

C0003864 GO:0000271 1 2

C0003864 GO:0015299 1 2

C0003864 GO:0015298 1 2

C0003864 GO:0042102 1 2

C0003864 GO:0007176 1 2

C0003864 GO:0032148 1 2

C0003864 GO:0055067 1 2

C0003864 GO:0003001 1 2

C0003864 GO:0006836 1 2

C0003864 GO:0007269 1 2

C0003864 GO:0002757 1 2

C0003864 GO:0050885 1 2

C0003864 GO:0030004 1 2

C0003864 GO:0010469 1 2

C0003864 GO:0002274 1 2

C0003864 GO:0002758 1 2

C0003864 GO:0050892 1 2

C0003864 GO:0002218 1 2

C0003864 GO:0045730 1 2

C0003864 GO:0004190 1 2

C0003864 GO:0002366 1 2

C0003864 GO:0030804 1 2

C0003864 GO:0005247 1 2

C0003864 GO:0048407 1 2

C0003864 GO:0005388 1 2

C0003864 GO:0017144 1 2

C0003864 GO:0045494 1 2

C0003864 GO:0015300 1 2

C0006277 GO:0051043 1 5

C0006277 GO:0045216 1 5

C0006277 GO:0046637 1 5

C0006277 GO:0043954 1 5

C0006277 GO:0031970 1 5

C0006277 GO:0032387 1 5

C0006277 GO:0043256 1 5

C0006277 GO:0021510 1 5

C0006277 GO:0008585 1 5

C0006277 GO:0043525 1 5

C0006277 GO:0019915 1 5

C0006277 GO:0051893 1 5

C0006277 GO:0031058 1 5

C0006277 GO:0015085 1 5

C0006277 GO:0043205 1 5

C0006277 GO:0015082 1 5

C0006277 GO:0043206 1 5

C0006277 GO:0007519 2 5

C0006277 GO:0070001 1 5

C0006277 GO:0006266 1 5

C0006277 GO:0016459 1 5

C0006277 GO:0004712 1 5

C0006277 GO:0010896 1 5

C0006277 GO:0010721 1 5

C0006277 GO:0010898 1 5

C0006277 GO:0046504 1 5

C0006277 GO:0033032 1 5

C0006277 GO:0060541 1 5

C0006277 GO:0016202 1 5

C0006277 GO:0046634 1 5

C0006277 GO:0004029 1 5

C0006277 GO:0030216 1 5

C0006277 GO:0014020 1 5

C0006277 GO:0031960 1 5

C0006277 GO:0005159 1 5

C0006277 GO:0005158 1 5

C0006277 GO:0007176 1 5

C0006277 GO:0030291 1 5

C0006277 GO:0042992 1 5

C0006277 GO:0001841 1 5

C0006277 GO:0045580 1 5

C0006277 GO:0005254 1 5

C0006277 GO:0002274 1 5

C0006277 GO:0001910 1 5

C0006277 GO:0046849 1 5

C0006277 GO:0007043 1 5

C0006277 GO:0005388 1 5

C0006277 GO:0004708 1 5

C0006277 GO:0045494 1 5

C0006277 GO:0030194 1 5

C0006277 GO:0021537 2 5

C0006277 GO:0009895 1 5

C0006277 GO:0007635 1 5

C0006277 GO:0001843 1 5

C0006277 GO:0051103 1 5

C0006277 GO:0019934 1 5

C0006277 GO:0050820 1 5

C0006277 GO:0032946 1 5

C0006277 GO:0009068 1 5

C0006277 GO:0003205 1 5

C0006277 GO:0003206 1 5

C0006277 GO:0043547 1 5

C0006277 GO:0046915 1 5

C0006277 GO:0006885 1 5

C0006277 GO:0008088 1 5

C0006277 GO:0006687 1 5

C0006277 GO:0005522 1 5

C0006277 GO:0006688 1 5

C0006277 GO:0051879 1 5

C0006277 GO:0046545 1 5

C0006277 GO:0009880 1 5

C0006277 GO:0048709 1 5

C0006277 GO:0005138 1 5

C0006277 GO:0060606 1 5

C0006277 GO:0048255 1 5

C0006277 GO:0042308 1 5

C0006277 GO:0014003 1 5

C0006277 GO:0042632 1 5

C0006277 GO:0046660 1 5

C0006277 GO:0045616 1 5

C0006277 GO:0004437 1 5

C0006277 GO:0050885 1 5

C0006277 GO:0007423 1 5

C0006277 GO:0050714 1 5

C0006277 GO:0045730 1 5

C0006277 GO:0046823 1 5

C0006277 GO:0005247 1 5

C0006277 GO:0043010 1 5

C0006277 GO:0032107 1 5

C0006277 GO:0032104 1 5

C0006277 GO:0048634 1 5

C0006277 GO:0035148 1 5

C0006277 GO:0006941 1 5

C0006277 GO:0002440 1 5

C0006277 GO:0005903 1 5

C0006277 GO:0046677 1 5

C0006277 GO:0050768 1 5

C0006277 GO:0051453 1 5

C0006277 GO:0043087 1 5

C0006277 GO:0045078 1 5

C0006277 GO:0043489 1 5

C0006277 GO:0045072 1 5

C0006277 GO:0016055 1 5

C0006277 GO:0046782 1 5

C0006277 GO:0002224 1 5

C0006277 GO:0060562 1 5

C0006277 GO:0045622 1 5

C0006277 GO:0043034 1 5

C0006277 GO:0042169 1 5

C0006277 GO:0015296 1 5

C0006277 GO:0031330 1 5

C0006277 GO:0015459 1 5

C0006277 GO:0000217 1 5

C0006277 GO:0030004 1 5

C0006277 GO:0015807 1 5

C0006277 GO:0001654 1 5

C0006277 GO:0006929 1 5

C0006277 GO:0001656 1 5

C0006277 GO:0004693 1 5

C0006277 GO:0050679 1 5

C0006277 GO:0002460 1 5

C0006277 GO:0046906 1 5

C0006277 GO:0018105 1 5

C0006277 GO:0050671 1 5

C0006277 GO:0032862 1 5

C0006277 GO:0004190 1 5

C0006277 GO:0045619 1 5

C0006277 GO:0043370 1 5

C0006277 GO:0045861 1 5

C0006277 GO:0017144 1 5

C0006277 GO:0019200 1 5

C0006277 GO:0051384 1 5

C0006277 GO:0030324 1 5

C0006277 GO:0016338 1 5

C0006277 GO:0008286 1 5

C0006277 GO:0017048 1 5

C0006277 GO:0042058 1 5

C0006277 GO:0008210 1 5

C0006277 GO:0043506 2 5

C0006277 GO:0055092 1 5

C0006277 GO:0030641 1 5

C0006277 GO:0045768 1 5

C0006277 GO:0004861 1 5

C0006277 GO:0045767 1 5

C0006277 GO:0016538 1 5

C0006277 GO:0070665 1 5

C0006277 GO:0045604 1 5

C0006277 GO:0010469 1 5

C0006277 GO:0010741 1 5

C0006277 GO:0043588 1 5

C0006277 GO:0009247 1 5

C0006277 GO:0048592 1 5

C0006277 GO:0048593 1 5

C0006277 GO:0042102 1 5

C0006277 GO:0032856 1 5

C0006277 GO:0031575 1 5

C0006277 GO:0055067 1 5

C0006277 GO:0031214 1 5

C0006277 GO:0051646 1 5

C0006277 GO:0007266 1 5

C0006277 GO:0035295 1 5

C0006277 GO:0001838 1 5

C0006277 GO:0032318 1 5

C0006277 GO:0043197 1 5

C0006277 GO:0045211 1 5

C0006277 GO:0050995 1 5

C0006277 GO:0050994 1 5

C0006277 GO:0032320 1 5

C0006277 GO:0032321 1 5

C0006277 GO:0030900 1 5

C0006277 GO:0002250 1 5

C0006277 GO:0060538 2 5

C0006277 GO:0005161 1 5

C0006277 GO:0001937 1 5

C0009951 GO:0019200 1 2

C0009951 GO:0030324 1 2

C0009951 GO:0007635 1 2

C0009951 GO:0043954 1 2

C0009951 GO:0051103 1 2

C0009951 GO:0042058 1 2

C0009951 GO:0014003 1 2

C0009951 GO:0008210 1 2

C0009951 GO:0032946 1 2

C0009951 GO:0051453 1 2

C0009951 GO:0009068 1 2

C0009951 GO:0030641 1 2

C0009951 GO:0003205 1 2

C0009951 GO:0015085 1 2

C0009951 GO:0003206 1 2

C0009951 GO:0015082 1 2

C0009951 GO:0006885 1 2

C0009951 GO:0008088 1 2

C0009951 GO:0006266 1 2

C0009951 GO:0001910 1 2

C0009951 GO:0070665 1 2

C0009951 GO:0006687 1 2

C0009951 GO:0002224 1 2

C0009951 GO:0005254 1 2

C0009951 GO:0060541 1 2

C0009951 GO:0004029 1 2

C0009951 GO:0030216 1 2

C0009951 GO:0048709 1 2

C0009951 GO:0042102 1 2

C0009951 GO:0007176 1 2

C0009951 GO:0055067 1 2

C0009951 GO:0043256 1 2

C0009951 GO:0050679 1 2

C0009951 GO:0050885 1 2

C0009951 GO:0030004 1 2

C0009951 GO:0010469 1 2

C0009951 GO:0002274 1 2

C0009951 GO:0050671 1 2

C0009951 GO:0045730 1 2

C0009951 GO:0046849 1 2

C0009951 GO:0005247 1 2

C0009951 GO:0005388 1 2

C0009951 GO:0017144 1 2

C0009951 GO:0045494 1 2

C0013404 GO:0052192 1 9

C0013404 GO:0005788 1 9

C0013404 GO:0051044 1 9

C0013404 GO:0005149 1 9

C0013404 GO:0032770 1 9

C0013404 GO:0043954 1 9

C0013404 GO:0045429 1 9

C0013404 GO:0005518 1 9

C0013404 GO:0030427 1 9

C0013404 GO:0030426 1 9

C0013404 GO:0006477 1 9

C0013404 GO:0030509 1 9

C0013404 GO:0007162 1 9

C0013404 GO:0050808 1 9

C0013404 GO:0043524 1 9

C0013404 GO:0002702 1 9

C0013404 GO:0045165 1 9

C0013404 GO:0008375 1 9

C0013404 GO:0046718 1 9

C0013404 GO:0001654 1 9

C0013404 GO:0016863 2 9

C0013404 GO:0051896 1 9

C0013404 GO:0001894 1 9

C0013404 GO:0015085 3 9

C0013404 GO:0006305 1 9

C0013404 GO:0006304 1 9

C0013404 GO:0048864 1 9

C0013404 GO:0015082 1 9

C0013404 GO:0004407 1 9

C0013404 GO:0046631 1 9

C0013404 GO:0005770 1 9

C0013404 GO:0018212 1 9

C0013404 GO:0018210 2 9

C0013404 GO:0001893 1 9

C0013404 GO:0044419 1 9

C0013404 GO:0006266 1 9

C0013404 GO:0004715 1 9

C0013404 GO:0045661 1 9

C0013404 GO:0045839 1 9

C0013404 GO:0030169 1 9

C0013404 GO:0045669 1 9

C0013404 GO:0033032 1 9

C0013404 GO:0060541 1 9

C0013404 GO:0070228 1 9

C0013404 GO:0004029 1 9

C0013404 GO:0030216 1 9

C0013404 GO:0002720 1 9

C0013404 GO:0007612 1 9

C0013404 GO:0051059 1 9

C0013404 GO:0021510 1 9

C0013404 GO:0007176 1 9

C0013404 GO:0043535 2 9

C0013404 GO:0007173 1 9

C0013404 GO:0048638 1 9

C0013404 GO:0070668 1 9

C0013404 GO:0051701 1 9

C0013404 GO:0043536 1 9

C0013404 GO:0010874 2 9

C0013404 GO:0010875 3 9

C0013404 GO:0019827 1 9

C0013404 GO:0007270 1 9

C0013404 GO:0018209 1 9

C0013404 GO:0043603 1 9

C0013404 GO:0034284 1 9

C0013404 GO:0016860 1 9

C0013404 GO:0006041 1 9

C0013404 GO:0016055 1 9

C0013404 GO:0006044 1 9

C0013404 GO:0050926 1 9

C0013404 GO:0060393 1 9

C0013404 GO:0002377 1 9

C0013404 GO:0001910 1 9

C0013404 GO:0051784 1 9

C0013404 GO:0048660 1 9

C0013404 GO:0016812 1 9

C0013404 GO:0005388 1 9

C0013404 GO:0004709 2 9

C0013404 GO:0045494 1 9

C0013404 GO:0021537 1 9

C0013404 GO:0007632 1 9

C0013404 GO:0002274 1 9

C0013404 GO:0007635 1 9

C0013404 GO:0032768 1 9

C0013404 GO:0019882 1 9

C0013404 GO:0051103 1 9

C0013404 GO:0010906 1 9

C0013404 GO:0052126 1 9

C0013404 GO:0043025 1 9

C0013404 GO:0001764 1 9

C0013404 GO:0000786 1 9

C0013404 GO:0048002 1 9

C0013404 GO:0030286 1 9

C0013404 GO:0051353 2 9

C0013404 GO:0051224 1 9

C0013404 GO:0048488 2 9

C0013404 GO:0007062 2 9

C0013404 GO:0031577 1 9

C0013404 GO:0002286 1 9

C0013404 GO:0030890 1 9

C0013404 GO:0002285 1 9

C0013404 GO:0004428 1 9

C0013404 GO:0006885 1 9

C0013404 GO:0043548 1 9

C0013404 GO:0050700 2 9

C0013404 GO:0008088 2 9

C0013404 GO:0015149 1 9

C0013404 GO:0043028 1 9

C0013404 GO:0046850 1 9

C0013404 GO:0006110 2 9

C0013404 GO:0045912 1 9

C0013404 GO:0045727 1 9

C0013404 GO:0005254 1 9

C0013404 GO:0030879 1 9

C0013404 GO:0043027 1 9

C0013404 GO:0071174 1 9

C0013404 GO:0019883 1 9

C0013404 GO:0071173 1 9

C0013404 GO:0020037 1 9

C0013404 GO:0006306 1 9

C0013404 GO:0030371 1 9

C0013404 GO:0008286 1 9

C0013404 GO:0050796 1 9

C0013404 GO:0008013 2 9

C0013404 GO:0060070 2 9

C0013404 GO:0050810 1 9

C0013404 GO:0035303 1 9

C0013404 GO:0033483 1 9

C0013404 GO:0033558 1 9

C0013404 GO:0016597 1 9

C0013404 GO:0044275 1 9

C0013404 GO:0002263 1 9

C0013404 GO:0030675 1 9

C0013404 GO:0008091 2 9

C0013404 GO:0030863 1 9

C0013404 GO:0032364 1 9

C0013404 GO:0016830 1 9

C0013404 GO:0030864 1 9

C0013404 GO:0033261 1 9

C0013404 GO:0017080 2 9

C0013404 GO:0045730 2 9

C0013404 GO:0043034 1 9

C0013404 GO:0010677 1 9

C0013404 GO:0005247 1 9

C0013404 GO:0001709 1 9

C0013404 GO:0043010 1 9

C0013404 GO:0003785 1 9

C0013404 GO:0045841 1 9

C0013404 GO:0032376 3 9

C0013404 GO:0016790 1 9

C0013404 GO:0033613 1 9

C0013404 GO:0019956 1 9

C0013404 GO:0010921 1 9

C0013404 GO:0048145 1 9

C0013404 GO:0015695 1 9

C0013404 GO:0016331 1 9

C0013404 GO:0017022 1 9

C0013404 GO:0032370 3 9

C0013404 GO:0005901 1 9

C0013404 GO:0046112 1 9

C0013404 GO:0009895 1 9

C0013404 GO:0048662 1 9

C0013404 GO:0051453 1 9

C0013404 GO:0005871 1 9

C0013404 GO:0033280 1 9

C0013404 GO:0030705 1 9

C0013404 GO:0043087 1 9

C0013404 GO:0009124 1 9

C0013404 GO:0070001 1 9

C0013404 GO:0007127 1 9

C0013404 GO:0043560 1 9

C0013404 GO:0007250 1 9

C0013404 GO:0032374 1 9

C0013404 GO:0010563 1 9

C0013404 GO:0032371 1 9

C0013404 GO:0045667 1 9

C0013404 GO:0032373 3 9

C0013404 GO:0050819 1 9

C0013404 GO:0043325 1 9

C0013404 GO:0032273 1 9

C0013404 GO:0002224 1 9

C0013404 GO:0030260 1 9

C0013404 GO:0005355 1 9

C0013404 GO:0042169 1 9

C0013404 GO:0015145 1 9

C0013404 GO:0006879 1 9

C0013404 GO:0008329 1 9

C0013404 GO:0031333 1 9

C0013404 GO:0008517 1 9

C0013404 GO:0015929 1 9

C0013404 GO:0007163 1 9

C0013404 GO:0030004 1 9

C0013404 GO:0015807 2 9

C0013404 GO:0002440 1 9

C0013404 GO:0046887 1 9

C0013404 GO:0005593 1 9

C0013404 GO:0009953 1 9

C0013404 GO:0007131 2 9

C0013404 GO:0042562 1 9

C0013404 GO:0051983 1 9

C0013404 GO:0043470 1 9

C0013404 GO:0043471 1 9

C0013404 GO:0006081 1 9

C0013404 GO:0002460 1 9

C0013404 GO:0043120 1 9

C0013404 GO:0034103 1 9

C0013404 GO:0051828 1 9

C0013404 GO:0004190 1 9

C0013404 GO:0002366 1 9

C0013404 GO:0005044 1 9

C0013404 GO:0034405 1 9

C0013404 GO:0017144 1 9

C0013404 GO:0016409 1 9

C0013404 GO:0017046 1 9

C0013404 GO:0018107 1 9

C0013404 GO:0019200 2 9

C0013404 GO:0018105 1 9

C0013404 GO:0030324 1 9

C0013404 GO:0005160 1 9

C0013404 GO:0016338 1 9

C0013404 GO:0042113 1 9

C0013404 GO:0018108 1 9

C0013404 GO:0042058 1 9

C0013404 GO:0045884 1 9

C0013404 GO:0010948 1 9

C0013404 GO:0008034 1 9

C0013404 GO:0043506 1 9

C0013404 GO:0002724 2 9

C0013404 GO:0001530 1 9

C0013404 GO:0009749 1 9

C0013404 GO:0006096 1 9

C0013404 GO:0030641 1 9

C0013404 GO:0045684 1 9

C0013404 GO:0032355 2 9

C0013404 GO:0001727 1 9

C0013404 GO:0030520 1 9

C0013404 GO:0032350 1 9

C0013404 GO:0042354 1 9

C0013404 GO:0032210 1 9

C0013404 GO:0002573 1 9

C0013404 GO:0050927 1 9

C0013404 GO:0030323 1 9

C0013404 GO:0070412 1 9

C0013404 GO:0070410 2 9

C0013404 GO:0007098 1 9

C0013404 GO:0070491 1 9

C0013404 GO:0010469 1 9

C0013404 GO:0030195 1 9

C0013404 GO:0051897 1 9

C0013404 GO:0015179 1 9

C0013404 GO:0019213 1 9

C0013404 GO:0034451 1 9

C0013404 GO:0021915 1 9

C0013404 GO:0045088 1 9

C0013404 GO:0070120 1 9

C0013404 GO:0045936 1 9

C0013404 GO:0031513 1 9

C0013404 GO:0048593 1 9

C0013404 GO:0045089 1 9

C0013404 GO:0010595 1 9

C0013404 GO:0042102 1 9

C0013404 GO:0009112 1 9

C0013404 GO:0050660 1 9

C0013404 GO:0055067 1 9

C0013404 GO:0002718 2 9

C0013404 GO:0051806 1 9

C0013404 GO:0009746 1 9

C0013404 GO:0006493 1 9

C0013404 GO:0010594 2 9

C0013404 GO:0030048 1 9

C0013404 GO:0045123 1 9

C0013404 GO:0002793 1 9

C0013404 GO:0051923 1 9

C0013404 GO:0003705 1 9

C0013404 GO:0045124 1 9

C0013404 GO:0003707 1 9

C0013404 GO:0044409 1 9

C0013404 GO:0030900 1 9

C0013404 GO:0002250 1 9

C0013404 GO:0007094 1 9

C0013404 GO:0044403 1 9

C0013404 GO:0046697 1 9

C0013404 GO:0006413 1 9

C0013404 GO:0030228 1 9

C0015967 GO:0051044 1 15

C0015967 GO:0030228 2 15

C0015967 GO:0043954 2 15

C0015967 GO:0001505 1 15

C0015967 GO:0048260 1 15

C0015967 GO:0016863 2 15

C0015967 GO:0016860 1 15

C0015967 GO:0006306 1 15

C0015967 GO:0006305 1 15

C0015967 GO:0006304 2 15

C0015967 GO:0018212 1 15

C0015967 GO:0018210 2 15

C0015967 GO:0031513 1 15

C0015967 GO:0004715 1 15

C0015967 GO:0060541 1 15

C0015967 GO:0004029 1 15

C0015967 GO:0005788 1 15

C0015967 GO:0030291 1 15

C0015967 GO:0001843 1 15

C0015967 GO:0050679 1 15

C0015967 GO:0016812 1 15

C0015967 GO:0051784 2 15

C0015967 GO:0002702 1 15

C0015967 GO:0021537 1 15

C0015967 GO:0060263 1 15

C0015967 GO:0010906 1 15

C0015967 GO:0051224 1 15

C0015967 GO:0003205 1 15

C0015967 GO:0003206 1 15

C0015967 GO:0006885 1 15

C0015967 GO:0043028 1 15

C0015967 GO:0046850 1 15

C0015967 GO:0006110 2 15

C0015967 GO:0045912 1 15

C0015967 GO:0043025 1 15

C0015967 GO:0043027 1 15

C0015967 GO:0019882 1 15

C0015967 GO:0019883 1 15

C0015967 GO:0005071 1 15

C0015967 GO:0048709 1 15

C0015967 GO:0005138 1 15

C0015967 GO:0060606 1 15

C0015967 GO:0042439 1 15

C0015967 GO:0035303 2 15

C0015967 GO:0030675 2 15

C0015967 GO:0005436 1 15

C0015967 GO:0015074 1 15

C0015967 GO:0006693 1 15

C0015967 GO:0006692 1 15

C0015967 GO:0015179 1 15

C0015967 GO:0015175 1 15

C0015967 GO:0048638 1 15

C0015967 GO:0033613 1 15

C0015967 GO:0046579 1 15

C0015967 GO:0043256 1 15

C0015967 GO:0031228 2 15

C0015967 GO:0042177 1 15

C0015967 GO:0051453 1 15

C0015967 GO:0005871 1 15

C0015967 GO:0043241 1 15

C0015967 GO:0007127 1 15

C0015967 GO:0043560 1 15

C0015967 GO:0030705 1 15

C0015967 GO:0016459 1 15

C0015967 GO:0045740 1 15

C0015967 GO:0002224 1 15

C0015967 GO:0045622 1 15

C0015967 GO:0008286 2 15

C0015967 GO:0006879 1 15

C0015967 GO:0008329 1 15

C0015967 GO:0034637 1 15

C0015967 GO:0001654 1 15

C0015967 GO:0017048 1 15

C0015967 GO:0045216 1 15

C0015967 GO:0010677 1 15

C0015967 GO:0051983 1 15

C0015967 GO:0043120 1 15

C0015967 GO:0004190 1 15

C0015967 GO:0017046 1 15

C0015967 GO:0018107 1 15

C0015967 GO:0018105 1 15

C0015967 GO:0018108 1 15

C0015967 GO:0045884 1 15

C0015967 GO:0008210 1 15

C0015967 GO:0007270 1 15

C0015967 GO:0006096 1 15

C0015967 GO:0070491 1 15

C0015967 GO:0032355 2 15

C0015967 GO:0001727 1 15

C0015967 GO:0032350 1 15

C0015967 GO:0042354 1 15

C0015967 GO:0006527 1 15

C0015967 GO:0006525 1 15

C0015967 GO:0055092 1 15

C0015967 GO:0070665 1 15

C0015967 GO:0070668 1 15

C0015967 GO:0019213 1 15

C0015967 GO:0021915 1 15

C0015967 GO:0045088 1 15

C0015967 GO:0070120 1 15

C0015967 GO:0048593 1 15

C0015967 GO:0032856 1 15

C0015967 GO:0001838 1 15

C0015967 GO:0010595 1 15

C0015967 GO:0010594 2 15

C0015967 GO:0043536 1 15

C0015967 GO:0005044 1 15

C0015967 GO:0008517 1 15

C0015967 GO:0005041 1 15

C0015967 GO:0016986 1 15

C0015967 GO:0032321 1 15

C0015967 GO:0051806 1 15

C0015967 GO:0043112 1 15

C0015967 GO:0006413 1 15

C0015967 GO:0042133 1 15

C0015967 GO:0005149 1 15

C0015967 GO:0007612 1 15

C0015967 GO:0007162 1 15

C0015967 GO:0007163 1 15

C0015967 GO:0043524 1 15

C0015967 GO:0032210 1 15

C0015967 GO:0045767 1 15

C0015967 GO:0016597 1 15

C0015967 GO:0004407 1 15

C0015967 GO:0002263 1 15

C0015967 GO:0070875 1 15

C0015967 GO:0045667 1 15

C0015967 GO:0045661 1 15

C0015967 GO:0045669 1 15

C0015967 GO:0010522 1 15

C0015967 GO:0033032 2 15

C0015967 GO:0051057 1 15

C0015967 GO:0016202 1 15

C0015967 GO:0030216 1 15

C0015967 GO:0051059 1 15

C0015967 GO:0009074 1 15

C0015967 GO:0051701 1 15

C0015967 GO:0019827 1 15

C0015967 GO:0002429 1 15

C0015967 GO:0018209 1 15

C0015967 GO:0030260 1 15

C0015967 GO:0005388 1 15

C0015967 GO:0004709 2 15

C0015967 GO:0045841 2 15

C0015967 GO:0002274 1 15

C0015967 GO:0008252 1 15

C0015967 GO:0001764 2 15

C0015967 GO:0048002 1 15

C0015967 GO:0030286 1 15

C0015967 GO:0051181 1 15

C0015967 GO:0051180 1 15

C0015967 GO:0008088 2 15

C0015967 GO:0030148 1 15

C0015967 GO:0020037 1 15

C0015967 GO:0032768 1 15

C0015967 GO:0050796 1 15

C0015967 GO:0060070 2 15

C0015967 GO:0030069 1 15

C0015967 GO:0009953 1 15

C0015967 GO:0006895 1 15

C0015967 GO:0017080 2 15

C0015967 GO:0043010 1 15

C0015967 GO:0016790 1 15

C0015967 GO:0019956 1 15

C0015967 GO:0009312 1 15

C0015967 GO:0009895 1 15

C0015967 GO:0007043 1 15

C0015967 GO:0010563 1 15

C0015967 GO:0050768 1 15

C0015967 GO:0001530 1 15

C0015967 GO:0005355 1 15

C0015967 GO:0042169 2 15

C0015967 GO:0008034 1 15

C0015967 GO:0006929 1 15

C0015967 GO:0007131 2 15

C0015967 GO:0043470 1 15

C0015967 GO:0043471 1 15

C0015967 GO:0002460 1 15

C0015967 GO:0002237 1 15

C0015967 GO:0045619 2 15

C0015967 GO:0034405 1 15

C0015967 GO:0045616 1 15

C0015967 GO:0016409 1 15

C0015967 GO:0016331 1 15

C0015967 GO:0030323 1 15

C0015967 GO:0030324 1 15

C0015967 GO:0016338 2 15

C0015967 GO:0034623 1 15

C0015967 GO:0002724 2 15

C0015967 GO:0002720 1 15

C0015967 GO:0016538 1 15

C0015967 GO:0030934 1 15

C0015967 GO:0030900 1 15

C0015967 GO:0045936 1 15

C0015967 GO:0045089 2 15

C0015967 GO:0055067 1 15

C0015967 GO:0035295 1 15

C0015967 GO:0043624 1 15

C0015967 GO:0005605 1 15

C0015967 GO:0006493 1 15

C0015967 GO:0051923 1 15

C0015967 GO:0052192 1 15

C0015967 GO:0003707 1 15

C0015967 GO:0044409 1 15

C0015967 GO:0060538 1 15

C0015967 GO:0044403 1 15

C0015967 GO:0046697 1 15

C0015967 GO:0005782 1 15

C0015967 GO:0021510 1 15

C0015967 GO:0006477 1 15

C0015967 GO:0030509 1 15

C0015967 GO:0031057 1 15

C0015967 GO:0015085 3 15

C0015967 GO:0048864 1 15

C0015967 GO:0015082 1 15

C0015967 GO:0005770 2 15

C0015967 GO:0042562 1 15

C0015967 GO:0070001 1 15

C0015967 GO:0010721 1 15

C0015967 GO:0031907 1 15

C0015967 GO:0046870 1 15

C0015967 GO:0032722 1 15

C0015967 GO:0070228 1 15

C0015967 GO:0032153 1 15

C0015967 GO:0032155 1 15

C0015967 GO:0042129 1 15

C0015967 GO:0007176 1 15

C0015967 GO:0043535 2 15

C0015967 GO:0007173 1 15

C0015967 GO:0007172 1 15

C0015967 GO:0055117 1 15

C0015967 GO:0016055 1 15

C0015967 GO:0060393 1 15

C0015967 GO:0002377 1 15

C0015967 GO:0001910 1 15

C0015967 GO:0004551 1 15

C0015967 GO:0033558 1 15

C0015967 GO:0045494 1 15

C0015967 GO:0051103 1 15

C0015967 GO:0000786 2 15

C0015967 GO:0009068 1 15

C0015967 GO:0031577 2 15

C0015967 GO:0090101 1 15

C0015967 GO:0043548 2 15

C0015967 GO:0005522 1 15

C0015967 GO:0045725 1 15

C0015967 GO:0005254 1 15

C0015967 GO:0030879 1 15

C0015967 GO:0030371 1 15

C0015967 GO:0008301 1 15

C0015967 GO:0005930 1 15

C0015967 GO:0044275 1 15

C0015967 GO:0002757 1 15

C0015967 GO:0008091 2 15

C0015967 GO:0050810 1 15

C0015967 GO:0032770 1 15

C0015967 GO:0015280 1 15

C0015967 GO:0010921 1 15

C0015967 GO:0019200 2 15

C0015967 GO:0017022 1 15

C0015967 GO:0045580 2 15

C0015967 GO:0045429 1 15

C0015967 GO:0042572 1 15

C0015967 GO:0007250 1 15

C0015967 GO:0032374 1 15

C0015967 GO:0001709 2 15

C0015967 GO:0032371 1 15

C0015967 GO:0032370 3 15

C0015967 GO:0032373 3 15

C0015967 GO:0050819 1 15

C0015967 GO:0032273 1 15

C0015967 GO:0060562 1 15

C0015967 GO:0017157 1 15

C0015967 GO:0031333 1 15

C0015967 GO:0009247 1 15

C0015967 GO:0004861 1 15

C0015967 GO:0005593 1 15

C0015967 GO:0050771 1 15

C0015967 GO:0050770 2 15

C0015967 GO:0050671 2 15

C0015967 GO:0051828 1 15

C0015967 GO:0001818 1 15

C0015967 GO:0005160 1 15

C0015967 GO:0005161 1 15

C0015967 GO:0042113 1 15

C0015967 GO:0042058 1 15

C0015967 GO:0043506 2 15

C0015967 GO:0045768 1 15

C0015967 GO:0032984 1 15

C0015967 GO:0043467 1 15

C0015967 GO:0002573 2 15

C0015967 GO:0015695 1 15

C0015967 GO:0045604 1 15

C0015967 GO:0015026 1 15

C0015967 GO:0016229 1 15

C0015967 GO:0050851 1 15

C0015967 GO:0050852 1 15

C0015967 GO:0009112 1 15

C0015967 GO:0003785 1 15

C0015967 GO:0002718 2 15

C0015967 GO:0034185 1 15

C0015967 GO:0016877 1 15

C0015967 GO:0045123 1 15

C0015967 GO:0045124 1 15

C0015967 GO:0007098 1 15

C0015967 GO:0007094 2 15

C0015967 GO:0046637 1 15

C0015967 GO:0046634 1 15

C0015967 GO:0030427 1 15

C0015967 GO:0030426 1 15

C0015967 GO:0046631 3 15

C0015967 GO:0050926 2 15

C0015967 GO:0055072 1 15

C0015967 GO:0008375 1 15

C0015967 GO:0051897 1 15

C0015967 GO:0051896 1 15

C0015967 GO:0007519 1 15

C0015967 GO:0044419 1 15

C0015967 GO:0006266 1 15

C0015967 GO:0043325 2 15

C0015967 GO:0045839 2 15

C0015967 GO:0032934 2 15

C0015967 GO:0006760 1 15

C0015967 GO:0014020 1 15

C0015967 GO:0048634 1 15

C0015967 GO:0030048 1 15

C0015967 GO:0010975 2 15

C0015967 GO:0010874 2 15

C0015967 GO:0010875 3 15

C0015967 GO:0048871 1 15

C0015967 GO:0006041 1 15

C0015967 GO:0006044 1 15

C0015967 GO:0051353 2 15

C0015967 GO:0046849 1 15

C0015967 GO:0019439 1 15

C0015967 GO:0043034 2 15

C0015967 GO:0007632 1 15

C0015967 GO:0007635 1 15

C0015967 GO:0034451 1 15

C0015967 GO:0032946 1 15

C0015967 GO:0032496 1 15

C0015967 GO:0048488 3 15

C0015967 GO:0007062 2 15

C0015967 GO:0002286 1 15

C0015967 GO:0002285 1 15

C0015967 GO:0004428 1 15

C0015967 GO:0050700 1 15

C0015967 GO:0015149 1 15

C0015967 GO:0002366 1 15

C0015967 GO:0006687 1 15

C0015967 GO:0001841 1 15

C0015967 GO:0006688 1 15

C0015967 GO:0015145 1 15

C0015967 GO:0071174 2 15

C0015967 GO:0071173 2 15

C0015967 GO:0030890 1 15

C0015967 GO:0033764 1 15

C0015967 GO:0008013 2 15

C0015967 GO:0014003 1 15

C0015967 GO:0042632 1 15

C0015967 GO:0045165 1 15

C0015967 GO:0050885 1 15

C0015967 GO:0030863 1 15

C0015967 GO:0030864 1 15

C0015967 GO:0033261 2 15

C0015967 GO:0045730 2 15

C0015967 GO:0045737 1 15

C0015967 GO:0030195 1 15

C0015967 GO:0005247 1 15

C0015967 GO:0046718 1 15

C0015967 GO:0032376 3 15

C0015967 GO:0035148 1 15

C0015967 GO:0006941 1 15

C0015967 GO:0005901 1 15

C0015967 GO:0006942 1 15

C0015967 GO:0052126 1 15

C0015967 GO:0033280 1 15

C0015967 GO:0043087 1 15

C0015967 GO:0009124 2 15

C0015967 GO:0046467 1 15

C0015967 GO:0070688 1 15

C0015967 GO:0030004 1 15

C0015967 GO:0015807 2 15

C0015967 GO:0001894 1 15

C0015967 GO:0001893 1 15

C0015967 GO:0006081 1 15

C0015967 GO:0043603 1 15

C0015967 GO:0043370 1 15

C0015967 GO:0045861 1 15

C0015967 GO:0017144 1 15

C0015967 GO:0000185 1 15

C0015967 GO:0003705 2 15

C0015967 GO:0010948 1 15

C0015967 GO:0032862 1 15

C0015967 GO:0030641 1 15

C0015967 GO:0045684 1 15

C0015967 GO:0050661 1 15

C0015967 GO:0050660 1 15

C0015967 GO:0030520 1 15

C0015967 GO:0050927 2 15

C0015967 GO:0070412 1 15

C0015967 GO:0070410 3 15

C0015967 GO:0010469 1 15

C0015967 GO:0005697 1 15

C0015967 GO:0034103 1 15

C0015967 GO:0048662 1 15

C0015967 GO:0048660 1 15

C0015967 GO:0042102 2 15

C0015967 GO:0005884 1 15

C0015967 GO:0031214 1 15

C0015967 GO:0002440 1 15

C0015967 GO:0019047 1 15

C0015967 GO:0002250 1 15

C0020538 GO:0051043 1 11

C0020538 GO:0051044 1 11

C0020538 GO:0034358 1 11

C0020538 GO:0043954 2 11

C0020538 GO:0009164 1 11

C0020538 GO:0001508 1 11

C0020538 GO:0016863 1 11

C0020538 GO:0016860 1 11

C0020538 GO:0014031 1 11

C0020538 GO:0000002 1 11

C0020538 GO:0043206 1 11

C0020538 GO:0016459 1 11

C0020538 GO:0004712 1 11

C0020538 GO:0046504 1 11

C0020538 GO:0060541 3 11

C0020538 GO:0030595 1 11

C0020538 GO:0004029 1 11

C0020538 GO:0035272 1 11

C0020538 GO:0006477 1 11

C0020538 GO:0030291 1 11

C0020538 GO:0030295 2 11

C0020538 GO:0016812 1 11

C0020538 GO:0016814 1 11

C0020538 GO:0004629 1 11

C0020538 GO:0045821 1 11

C0020538 GO:0010889 1 11

C0020538 GO:0021537 2 11

C0020538 GO:0060263 1 11

C0020538 GO:0009898 2 11

C0020538 GO:0046658 1 11

C0020538 GO:0000578 1 11

C0020538 GO:0010862 1 11

C0020538 GO:0051224 1 11

C0020538 GO:0032318 1 11

C0020538 GO:0006885 2 11

C0020538 GO:0008643 1 11

C0020538 GO:0006112 1 11

C0020538 GO:0045912 2 11

C0020538 GO:0051879 1 11

C0020538 GO:0043027 1 11

C0020538 GO:0048705 1 11

C0020538 GO:0019882 1 11

C0020538 GO:0019883 1 11

C0020538 GO:0031256 1 11

C0020538 GO:0005138 1 11

C0020538 GO:0043410 1 11

C0020538 GO:0043205 1 11

C0020538 GO:0035303 1 11

C0020538 GO:0004437 1 11

C0020538 GO:0004435 1 11

C0020538 GO:0004434 1 11

C0020538 GO:0050714 2 11

C0020538 GO:0042605 1 11

C0020538 GO:0070567 1 11

C0020538 GO:0008021 1 11

C0020538 GO:0048146 1 11

C0020538 GO:0048634 2 11

C0020538 GO:0051453 2 11

C0020538 GO:0051457 1 11

C0020538 GO:0031225 1 11

C0020538 GO:0045078 1 11

C0020538 GO:0043489 1 11

C0020538 GO:0045072 1 11

C0020538 GO:0045740 1 11

C0020538 GO:0002224 1 11

C0020538 GO:0005506 2 11

C0020538 GO:0045622 1 11

C0020538 GO:0008286 1 11

C0020538 GO:0008329 1 11

C0020538 GO:0045211 1 11

C0020538 GO:0001654 3 11

C0020538 GO:0001656 1 11

C0020538 GO:0010677 2 11

C0020538 GO:0004190 1 11

C0020538 GO:0018105 1 11

C0020538 GO:0045927 1 11

C0020538 GO:0048762 1 11

C0020538 GO:0017048 1 11

C0020538 GO:0060021 1 11

C0020538 GO:0045445 1 11

C0020538 GO:0030425 1 11

C0020538 GO:0006090 1 11

C0020538 GO:0055092 1 11

C0020538 GO:0009820 1 11

C0020538 GO:0010828 1 11

C0020538 GO:0006700 1 11

C0020538 GO:0048592 1 11

C0020538 GO:0048593 1 11

C0020538 GO:0032856 1 11

C0020538 GO:0060485 1 11

C0020538 GO:0001838 1 11

C0020538 GO:0010595 1 11

C0020538 GO:0010594 1 11

C0020538 GO:0043536 1 11

C0020538 GO:0050995 1 11

C0020538 GO:0050994 1 11

C0020538 GO:0001837 1 11

C0020538 GO:0032320 1 11

C0020538 GO:0032321 1 11

C0020538 GO:0006413 1 11

C0020538 GO:0019362 1 11

C0020538 GO:0043525 1 11

C0020538 GO:0019915 1 11

C0020538 GO:0005540 1 11

C0020538 GO:0002263 1 11

C0020538 GO:0030323 1 11

C0020538 GO:0033032 1 11

C0020538 GO:0016202 2 11

C0020538 GO:0034364 1 11

C0020538 GO:0034366 1 11

C0020538 GO:0030217 1 11

C0020538 GO:0051059 1 11

C0020538 GO:0050804 1 11

C0020538 GO:0042992 1 11

C0020538 GO:0014020 1 11

C0020538 GO:0018209 1 11

C0020538 GO:0016566 1 11

C0020538 GO:0005388 1 11

C0020538 GO:0004708 1 11

C0020538 GO:0016055 1 11

C0020538 GO:0050821 1 11

C0020538 GO:0050820 1 11

C0020538 GO:0046496 1 11

C0020538 GO:0005929 2 11

C0020538 GO:0048002 1 11

C0020538 GO:0046915 1 11

C0020538 GO:0008088 1 11

C0020538 GO:0030148 1 11

C0020538 GO:0009880 2 11

C0020538 GO:0006584 1 11

C0020538 GO:0046660 1 11

C0020538 GO:0017080 1 11

C0020538 GO:0046823 1 11

C0020538 GO:0043010 2 11

C0020538 GO:0042834 1 11

C0020538 GO:0006633 1 11

C0020538 GO:0032107 1 11

C0020538 GO:0032104 1 11

C0020538 GO:0019894 1 11

C0020538 GO:0009895 1 11

C0020538 GO:0007043 1 11

C0020538 GO:0050768 2 11

C0020538 GO:0042509 1 11

C0020538 GO:0042169 1 11

C0020538 GO:0006929 2 11

C0020538 GO:0022612 1 11

C0020538 GO:0032587 1 11

C0020538 GO:0008093 1 11

C0020538 GO:0002460 1 11

C0020538 GO:0045619 1 11

C0020538 GO:0045616 1 11

C0020538 GO:0051384 1 11

C0020538 GO:0030324 3 11

C0020538 GO:0001843 1 11

C0020538 GO:0016339 1 11

C0020538 GO:0050866 1 11

C0020538 GO:0046890 1 11

C0020538 GO:0045216 2 11

C0020538 GO:0004181 1 11

C0020538 GO:0004675 1 11

C0020538 GO:0016538 1 11

C0020538 GO:0043588 1 11

C0020538 GO:0042581 1 11

C0020538 GO:0055067 1 11

C0020538 GO:0043627 2 11

C0020538 GO:0007266 1 11

C0020538 GO:0035295 1 11

C0020538 GO:0043190 1 11

C0020538 GO:0043197 1 11

C0020538 GO:0051923 1 11

C0020538 GO:0008645 1 11

C0020538 GO:0060538 2 11

C0020538 GO:0031970 1 11

C0020538 GO:0032387 1 11

C0020538 GO:0021510 1 11

C0020538 GO:0005788 1 11

C0020538 GO:0060326 1 11

C0020538 GO:0031058 1 11

C0020538 GO:0015085 1 11

C0020538 GO:0051205 1 11

C0020538 GO:0015082 1 11

C0020538 GO:0016338 2 11

C0020538 GO:0070001 1 11

C0020538 GO:0005024 1 11

C0020538 GO:0010721 2 11

C0020538 GO:0043124 1 11

C0020538 GO:0043120 1 11

C0020538 GO:0070228 1 11

C0020538 GO:0032655 1 11

C0020538 GO:0005159 1 11

C0020538 GO:0005158 1 11

C0020538 GO:0003007 1 11

C0020538 GO:0007176 1 11

C0020538 GO:0043535 1 11

C0020538 GO:0007172 1 11

C0020538 GO:0043025 1 11

C0020538 GO:0002274 1 11

C0020538 GO:0060393 1 11

C0020538 GO:0001910 1 11

C0020538 GO:0045494 3 11

C0020538 GO:0042398 1 11

C0020538 GO:0051103 1 11

C0020538 GO:0042625 1 11

C0020538 GO:0045058 1 11

C0020538 GO:0031575 1 11

C0020538 GO:0043547 1 11

C0020538 GO:0090101 1 11

C0020538 GO:0005522 1 11

C0020538 GO:0048641 1 11

C0020538 GO:0005254 1 11

C0020538 GO:0030374 1 11

C0020538 GO:0050810 1 11

C0020538 GO:0042308 1 11

C0020538 GO:0060606 1 11

C0020538 GO:0007423 1 11

C0020538 GO:0007422 1 11

C0020538 GO:0030159 1 11

C0020538 GO:0043043 1 11

C0020538 GO:0030275 1 11

C0020538 GO:0045580 1 11

C0020538 GO:0045429 2 11

C0020538 GO:0045428 1 11

C0020538 GO:0046677 1 11

C0020538 GO:0042274 1 11

C0020538 GO:0032376 1 11

C0020538 GO:0034311 1 11

C0020538 GO:0032370 1 11

C0020538 GO:0032373 1 11

C0020538 GO:0060562 1 11

C0020538 GO:0019209 2 11

C0020538 GO:0031330 1 11

C0020538 GO:0015459 1 11

C0020538 GO:0031333 1 11

C0020538 GO:0009247 1 11

C0020538 GO:0004861 1 11

C0020538 GO:0005593 1 11

C0020538 GO:0050771 1 11

C0020538 GO:0050770 1 11

C0020538 GO:0046906 1 11

C0020538 GO:0001950 1 11

C0020538 GO:0009187 1 11

C0020538 GO:0042119 3 11

C0020538 GO:0005161 2 11

C0020538 GO:0042058 1 11

C0020538 GO:0043506 2 11

C0020538 GO:0043507 1 11

C0020538 GO:0045768 1 11

C0020538 GO:0030216 1 11

C0020538 GO:0045766 1 11

C0020538 GO:0045767 1 11

C0020538 GO:0015758 1 11

C0020538 GO:0015695 1 11

C0020538 GO:0045604 1 11

C0020538 GO:0046425 1 11

C0020538 GO:0030235 1 11

C0020538 GO:0009110 1 11

C0020538 GO:0051646 1 11

C0020538 GO:0001530 1 11

C0020538 GO:0016877 1 11

C0020538 GO:0031526 1 11

C0020538 GO:0030900 1 11

C0020538 GO:0035065 1 11

C0020538 GO:0048638 1 11

C0020538 GO:0046637 1 11

C0020538 GO:0046634 1 11

C0020538 GO:0008585 1 11

C0020538 GO:0007292 1 11

C0020538 GO:0050926 1 11

C0020538 GO:0055072 1 11

C0020538 GO:0008272 1 11

C0020538 GO:0051893 1 11

C0020538 GO:0008375 1 11

C0020538 GO:0046631 1 11

C0020538 GO:0051897 1 11

C0020538 GO:0015280 1 11

C0020538 GO:0007519 2 11

C0020538 GO:0006266 1 11

C0020538 GO:0045833 1 11

C0020538 GO:0045834 1 11

C0020538 GO:0010896 1 11

C0020538 GO:0010898 2 11

C0020538 GO:0031960 1 11

C0020538 GO:0006769 1 11

C0020538 GO:0010975 1 11

C0020538 GO:0030041 1 11

C0020538 GO:0042345 2 11

C0020538 GO:0010874 1 11

C0020538 GO:0010875 1 11

C0020538 GO:0051353 1 11

C0020538 GO:0043034 1 11

C0020538 GO:0030194 1 11

C0020538 GO:0005089 1 11

C0020538 GO:0005088 1 11

C0020538 GO:0007635 1 11

C0020538 GO:0032642 1 11

C0020538 GO:0005086 1 11

C0020538 GO:0019934 1 11

C0020538 GO:0004693 1 11

C0020538 GO:0048488 1 11

C0020538 GO:0002286 1 11

C0020538 GO:0002285 1 11

C0020538 GO:0002366 1 11

C0020538 GO:0001841 1 11

C0020538 GO:0006688 1 11

C0020538 GO:0051131 1 11

C0020538 GO:0046545 1 11

C0020538 GO:0008154 2 11

C0020538 GO:0048255 1 11

C0020538 GO:0042632 1 11

C0020538 GO:0005844 1 11

C0020538 GO:0045165 1 11

C0020538 GO:0042734 2 11

C0020538 GO:0033261 1 11

C0020538 GO:0045730 1 11

C0020538 GO:0005247 1 11

C0020538 GO:0015662 1 11

C0020538 GO:0009712 1 11

C0020538 GO:0035148 1 11

C0020538 GO:0006941 1 11

C0020538 GO:0005903 1 11

C0020538 GO:0048029 2 11

C0020538 GO:0043087 1 11

C0020538 GO:0009124 1 11

C0020538 GO:0046782 1 11

C0020538 GO:0046467 1 11

C0020538 GO:0015296 2 11

C0020538 GO:0035035 1 11

C0020538 GO:0030004 2 11

C0020538 GO:0015807 1 11

C0020538 GO:0018958 1 11

C0020538 GO:0007569 1 11

C0020538 GO:0045296 1 11

C0020538 GO:0043370 1 11

C0020538 GO:0045861 2 11

C0020538 GO:0017144 1 11

C0020538 GO:0019200 1 11

C0020538 GO:0046326 1 11

C0020538 GO:0032862 1 11

C0020538 GO:0030641 2 11

C0020538 GO:0050660 1 11

C0020538 GO:0008443 1 11

C0020538 GO:0050927 1 11

C0020538 GO:0010469 1 11

C0020538 GO:0010741 1 11

C0020538 GO:0048662 1 11

C0020538 GO:0048660 1 11

C0020538 GO:0000217 1 11

C0020538 GO:0048742 1 11

C0020538 GO:0042102 1 11

C0020538 GO:0005884 1 11

C0020538 GO:0031214 1 11

C0020538 GO:0002440 1 11

C0020538 GO:0016049 1 11

C0020538 GO:0002443 1 11

C0020538 GO:0032994 1 11

C0020538 GO:0002250 1 11

C0020538 GO:0015749 1 11

C0020538 GO:0030228 1 11

C0020538 GO:0001937 1 11

C0020538 GO:0015036 1 11

C0020649 GO:0046637 1 8

C0020649 GO:0043954 1 8

C0020649 GO:0010677 2 8

C0020649 GO:0004629 1 8

C0020649 GO:0006476 1 8

C0020649 GO:0016917 1 8

C0020649 GO:0002705 1 8

C0020649 GO:0055072 1 8

C0020649 GO:0003015 1 8

C0020649 GO:0008272 1 8

C0020649 GO:0016864 1 8

C0020649 GO:0016863 1 8

C0020649 GO:0016862 1 8

C0020649 GO:0002709 1 8

C0020649 GO:0002708 1 8

C0020649 GO:0015085 1 8

C0020649 GO:0015082 1 8

C0020649 GO:0005540 1 8

C0020649 GO:0007519 1 8

C0020649 GO:0001822 1 8

C0020649 GO:0006266 1 8

C0020649 GO:0016459 1 8

C0020649 GO:0004890 1 8

C0020649 GO:0033032 1 8

C0020649 GO:0060541 1 8

C0020649 GO:0016202 1 8

C0020649 GO:0046634 1 8

C0020649 GO:0004029 1 8

C0020649 GO:0030216 1 8

C0020649 GO:0014020 2 8

C0020649 GO:0002824 1 8

C0020649 GO:0001841 2 8

C0020649 GO:0034637 1 8

C0020649 GO:0007176 1 8

C0020649 GO:0035272 1 8

C0020649 GO:0030291 1 8

C0020649 GO:0001843 2 8

C0020649 GO:0030169 1 8

C0020649 GO:0045580 1 8

C0020649 GO:0007270 1 8

C0020649 GO:0055092 1 8

C0020649 GO:0034284 1 8

C0020649 GO:0016860 1 8

C0020649 GO:0002274 1 8

C0020649 GO:0046530 1 8

C0020649 GO:0001910 1 8

C0020649 GO:0003995 1 8

C0020649 GO:0005388 1 8

C0020649 GO:0016445 1 8

C0020649 GO:0045494 1 8

C0020649 GO:0007632 1 8

C0020649 GO:0007635 1 8

C0020649 GO:0060173 1 8

C0020649 GO:0051103 1 8

C0020649 GO:0046326 1 8

C0020649 GO:0048246 1 8

C0020649 GO:0009068 1 8

C0020649 GO:0051224 1 8

C0020649 GO:0003073 1 8

C0020649 GO:0048261 1 8

C0020649 GO:0002764 1 8

C0020649 GO:0006885 1 8

C0020649 GO:0031672 1 8

C0020649 GO:0050700 1 8

C0020649 GO:0008088 1 8

C0020649 GO:0046850 1 8

C0020649 GO:0001754 1 8

C0020649 GO:0005522 1 8

C0020649 GO:0045912 2 8

C0020649 GO:0006688 1 8

C0020649 GO:0045727 1 8

C0020649 GO:0005254 1 8

C0020649 GO:0060606 2 8

C0020649 GO:0045862 1 8

C0020649 GO:0042632 1 8

C0020649 GO:0042102 1 8

C0020649 GO:0004435 1 8

C0020649 GO:0017144 1 8

C0020649 GO:0008093 1 8

C0020649 GO:0002711 1 8

C0020649 GO:0031645 1 8

C0020649 GO:0032364 1 8

C0020649 GO:0016830 1 8

C0020649 GO:0070567 1 8

C0020649 GO:0006693 1 8

C0020649 GO:0045730 1 8

C0020649 GO:0043034 1 8

C0020649 GO:0005247 1 8

C0020649 GO:0015175 1 8

C0020649 GO:0006752 1 8

C0020649 GO:0002699 1 8

C0020649 GO:0042834 1 8

C0020649 GO:0003785 1 8

C0020649 GO:0006633 1 8

C0020649 GO:0050808 1 8

C0020649 GO:0006636 1 8

C0020649 GO:0048145 1 8

C0020649 GO:0048634 1 8

C0020649 GO:0035148 2 8

C0020649 GO:0006941 1 8

C0020649 GO:0045582 1 8

C0020649 GO:0006942 1 8

C0020649 GO:0007043 1 8

C0020649 GO:0048029 1 8

C0020649 GO:0051453 1 8

C0020649 GO:0051457 1 8

C0020649 GO:0005123 1 8

C0020649 GO:0042274 1 8

C0020649 GO:0032376 1 8

C0020649 GO:0032370 1 8

C0020649 GO:0032373 1 8

C0020649 GO:0050431 1 8

C0020649 GO:0003756 1 8

C0020649 GO:0034341 1 8

C0020649 GO:0002224 1 8

C0020649 GO:0060562 1 8

C0020649 GO:0005506 1 8

C0020649 GO:0045622 1 8

C0020649 GO:0017157 1 8

C0020649 GO:0001838 2 8

C0020649 GO:0008034 1 8

C0020649 GO:0006879 1 8

C0020649 GO:0009247 1 8

C0020649 GO:0015929 1 8

C0020649 GO:0008320 1 8

C0020649 GO:0030004 1 8

C0020649 GO:0004434 1 8

C0020649 GO:0005913 1 8

C0020649 GO:0006929 1 8

C0020649 GO:0045216 1 8

C0020649 GO:0046887 1 8

C0020649 GO:0046889 1 8

C0020649 GO:0060021 2 8

C0020649 GO:0042593 1 8

C0020649 GO:0060047 1 8

C0020649 GO:0005024 1 8

C0020649 GO:0045619 1 8

C0020649 GO:0006004 1 8

C0020649 GO:0043370 1 8

C0020649 GO:0045616 1 8

C0020649 GO:0005518 2 8

C0020649 GO:0007229 1 8

C0020649 GO:0048736 1 8

C0020649 GO:0018107 1 8

C0020649 GO:0019200 1 8

C0020649 GO:0030324 1 8

C0020649 GO:0042177 1 8

C0020649 GO:0005929 1 8

C0020649 GO:0017048 1 8

C0020649 GO:0042058 1 8

C0020649 GO:0043506 1 8

C0020649 GO:0032862 1 8

C0020649 GO:0046112 1 8

C0020649 GO:0010828 1 8

C0020649 GO:0030641 1 8

C0020649 GO:0045768 1 8

C0020649 GO:0045732 1 8

C0020649 GO:0009636 1 8

C0020649 GO:0033500 1 8

C0020649 GO:0045767 1 8

C0020649 GO:0004675 1 8

C0020649 GO:0016538 1 8

C0020649 GO:0006692 1 8

C0020649 GO:0045604 1 8

C0020649 GO:0010745 1 8

C0020649 GO:0010469 1 8

C0020649 GO:0005112 1 8

C0020649 GO:0048531 1 8

C0020649 GO:0034103 1 8

C0020649 GO:0031334 1 8

C0020649 GO:0042100 1 8

C0020649 GO:0033483 1 8

C0020649 GO:0009112 1 8

C0020649 GO:0032856 1 8

C0020649 GO:0007159 1 8

C0020649 GO:0055067 1 8

C0020649 GO:0031214 1 8

C0020649 GO:0035295 1 8

C0020649 GO:0003746 1 8

C0020649 GO:0009746 1 8

C0020649 GO:0016338 1 8

C0020649 GO:0004861 1 8

C0020649 GO:0002793 1 8

C0020649 GO:0045124 1 8

C0020649 GO:0009749 1 8

C0020649 GO:0032321 1 8

C0020649 GO:0060538 1 8

C0020649 GO:0014069 1 8

C0020649 GO:0005161 1 8

C0020649 GO:0001655 1 8

C0030554 GO:0051043 1 8

C0030554 GO:0051044 1 8

C0030554 GO:0045216 1 8

C0030554 GO:0008374 1 8

C0030554 GO:0032107 1 8

C0030554 GO:0046637 1 8

C0030554 GO:0043954 2 8

C0030554 GO:0031970 1 8

C0030554 GO:0032387 1 8

C0030554 GO:0021510 1 8

C0030554 GO:0008585 1 8

C0030554 GO:0002286 1 8

C0030554 GO:0009164 1 8

C0030554 GO:0050927 1 8

C0030554 GO:0002703 1 8

C0030554 GO:0043525 1 8

C0030554 GO:0034199 1 8

C0030554 GO:0019915 1 8

C0030554 GO:0051893 1 8

C0030554 GO:0008375 1 8

C0030554 GO:0046631 1 8

C0030554 GO:0016597 1 8

C0030554 GO:0030228 1 8

C0030554 GO:0015085 1 8

C0030554 GO:0043205 1 8

C0030554 GO:0015082 1 8

C0030554 GO:0043206 1 8

C0030554 GO:0002263 1 8

C0030554 GO:0007519 2 8

C0030554 GO:0045616 1 8

C0030554 GO:0030295 1 8

C0030554 GO:0070001 1 8

C0030554 GO:0006266 1 8

C0030554 GO:0016459 1 8

C0030554 GO:0004712 1 8

C0030554 GO:0010874 1 8

C0030554 GO:0010896 1 8

C0030554 GO:0010721 1 8

C0030554 GO:0010898 1 8

C0030554 GO:0046504 1 8

C0030554 GO:0051897 1 8

C0030554 GO:0010875 1 8

C0030554 GO:0043120 1 8

C0030554 GO:0033032 1 8

C0030554 GO:0060541 1 8

C0030554 GO:0016202 1 8

C0030554 GO:0005977 1 8

C0030554 GO:0004029 1 8

C0030554 GO:0030216 1 8

C0030554 GO:0014020 1 8

C0030554 GO:0031960 1 8

C0030554 GO:0005159 1 8

C0030554 GO:0005158 1 8

C0030554 GO:0002822 1 8

C0030554 GO:0051059 1 8

C0030554 GO:0048634 1 8

C0030554 GO:0007281 1 8

C0030554 GO:0007176 1 8

C0030554 GO:0008170 1 8

C0030554 GO:0032373 1 8

C0030554 GO:0006477 1 8

C0030554 GO:0010975 1 8

C0030554 GO:0007286 1 8

C0030554 GO:0030291 1 8

C0030554 GO:0042992 1 8

C0030554 GO:0030512 1 8

C0030554 GO:0030296 1 8

C0030554 GO:0030169 1 8

C0030554 GO:0045580 1 8

C0030554 GO:0031343 1 8

C0030554 GO:0016812 1 8

C0030554 GO:0051879 1 8

C0030554 GO:0002274 1 8

C0030554 GO:0050926 1 8

C0030554 GO:0051353 1 8

C0030554 GO:0045682 1 8

C0030554 GO:0001910 2 8

C0030554 GO:0045821 1 8

C0030554 GO:0007043 1 8

C0030554 GO:0005388 1 8

C0030554 GO:0042269 1 8

C0030554 GO:0004708 1 8

C0030554 GO:0045494 2 8

C0030554 GO:0030194 1 8

C0030554 GO:0021537 2 8

C0030554 GO:0019320 1 8

C0030554 GO:0009895 1 8

C0030554 GO:0019209 1 8

C0030554 GO:0007635 1 8

C0030554 GO:0001843 1 8

C0030554 GO:0042398 1 8

C0030554 GO:0030345 1 8

C0030554 GO:0051103 1 8

C0030554 GO:0019934 1 8

C0030554 GO:0050820 1 8

C0030554 GO:0019883 1 8

C0030554 GO:0031058 1 8

C0030554 GO:0048002 1 8

C0030554 GO:0021700 1 8

C0030554 GO:0016863 1 8

C0030554 GO:0007530 1 8

C0030554 GO:0031575 1 8

C0030554 GO:0016049 1 8

C0030554 GO:0043547 1 8

C0030554 GO:0046915 1 8

C0030554 GO:0006885 2 8

C0030554 GO:0008088 1 8

C0030554 GO:0008643 1 8

C0030554 GO:0019047 1 8

C0030554 GO:0002366 1 8

C0030554 GO:0005522 1 8

C0030554 GO:0045912 1 8

C0030554 GO:0006688 1 8

C0030554 GO:0016860 1 8

C0030554 GO:0005254 1 8

C0030554 GO:0030879 1 8

C0030554 GO:0002688 1 8

C0030554 GO:0019882 1 8

C0030554 GO:0004675 1 8

C0030554 GO:0046545 1 8

C0030554 GO:0009880 1 8

C0030554 GO:0032769 1 8

C0030554 GO:0005138 2 8

C0030554 GO:0060606 1 8

C0030554 GO:0050810 1 8

C0030554 GO:0048255 1 8

C0030554 GO:0042308 1 8

C0030554 GO:0050715 1 8

C0030554 GO:0042632 1 8

C0030554 GO:0030069 1 8

C0030554 GO:0046660 1 8

C0030554 GO:0005930 1 8

C0030554 GO:0042301 1 8

C0030554 GO:0004437 1 8

C0030554 GO:0006007 1 8

C0030554 GO:0007250 1 8

C0030554 GO:0042734 1 8

C0030554 GO:0050714 1 8

C0030554 GO:0031645 1 8

C0030554 GO:0050770 1 8

C0030554 GO:0017080 1 8

C0030554 GO:0045730 2 8

C0030554 GO:0048742 1 8

C0030554 GO:0046823 1 8

C0030554 GO:0005247 1 8

C0030554 GO:0051384 1 8

C0030554 GO:0002715 1 8

C0030554 GO:0006633 1 8

C0030554 GO:0008021 1 8

C0030554 GO:0032104 1 8

C0030554 GO:0005788 1 8

C0030554 GO:0044042 1 8

C0030554 GO:0035148 1 8

C0030554 GO:0006941 1 8

C0030554 GO:0043627 1 8

C0030554 GO:0045429 1 8

C0030554 GO:0005903 1 8

C0030554 GO:0046677 1 8

C0030554 GO:0050768 1 8

C0030554 GO:0051453 2 8

C0030554 GO:0048641 1 8

C0030554 GO:0043087 1 8

C0030554 GO:0009124 1 8

C0030554 GO:0050868 1 8

C0030554 GO:0045078 1 8

C0030554 GO:0043489 1 8

C0030554 GO:0001841 1 8

C0030554 GO:0032376 1 8

C0030554 GO:0048638 1 8

C0030554 GO:0032370 1 8

C0030554 GO:0045072 1 8

C0030554 GO:0016055 1 8

C0030554 GO:0046782 1 8

C0030554 GO:0002224 1 8

C0030554 GO:0060562 1 8

C0030554 GO:0045622 1 8

C0030554 GO:0043034 1 8

C0030554 GO:0044275 1 8

C0030554 GO:0042169 1 8

C0030554 GO:0015296 1 8

C0030554 GO:0043535 1 8

C0030554 GO:0006073 1 8

C0030554 GO:0008329 1 8

C0030554 GO:0031330 1 8

C0030554 GO:0015459 1 8

C0030554 GO:0000217 1 8

C0030554 GO:0045211 1 8

C0030554 GO:0015807 1 8

C0030554 GO:0002440 1 8

C0030554 GO:0006929 1 8

C0030554 GO:0001656 1 8

C0030554 GO:0010677 1 8

C0030554 GO:0005593 1 8

C0030554 GO:0006413 1 8

C0030554 GO:0004693 1 8

C0030554 GO:0010594 1 8

C0030554 GO:0043536 1 8

C0030554 GO:0007423 1 8

C0030554 GO:0002460 1 8

C0030554 GO:0031397 1 8

C0030554 GO:0046906 1 8

C0030554 GO:0018105 1 8

C0030554 GO:0005024 1 8

C0030554 GO:0032862 1 8

C0030554 GO:0004190 1 8

C0030554 GO:0045619 1 8

C0030554 GO:0043370 1 8

C0030554 GO:0045861 1 8

C0030554 GO:0017144 1 8

C0030554 GO:0019200 1 8

C0030554 GO:0009187 1 8

C0030554 GO:0030324 1 8

C0030554 GO:0045927 1 8

C0030554 GO:0042119 1 8

C0030554 GO:0046365 1 8

C0030554 GO:0051923 1 8

C0030554 GO:0050866 1 8

C0030554 GO:0008286 1 8

C0030554 GO:0017048 2 8

C0030554 GO:0042058 1 8

C0030554 GO:0051181 1 8

C0030554 GO:0070228 1 8

C0030554 GO:0043506 2 8

C0030554 GO:0043507 1 8

C0030554 GO:0006090 1 8

C0030554 GO:0055092 1 8

C0030554 GO:0030641 2 8

C0030554 GO:0045165 1 8

C0030554 GO:0045768 1 8

C0030554 GO:0015074 1 8

C0030554 GO:0004861 1 8

C0030554 GO:0045767 1 8

C0030554 GO:0015758 1 8

C0030554 GO:0016538 1 8

C0030554 GO:0015695 1 8

C0030554 GO:0008543 1 8

C0030554 GO:0045604 1 8

C0030554 GO:0010469 1 8

C0030554 GO:0010741 1 8

C0030554 GO:0043588 1 8

C0030554 GO:0002690 1 8

C0030554 GO:0046634 1 8

C0030554 GO:0048662 1 8

C0030554 GO:0048660 1 8

C0030554 GO:0009247 1 8

C0030554 GO:0048592 1 8

C0030554 GO:0048593 1 8

C0030554 GO:0030021 1 8

C0030554 GO:0042102 1 8

C0030554 GO:0002285 1 8

C0030554 GO:0032856 1 8

C0030554 GO:0009110 1 8

C0030554 GO:0055067 1 8

C0030554 GO:0031214 2 8

C0030554 GO:0051646 1 8

C0030554 GO:0007266 1 8

C0030554 GO:0035295 1 8

C0030554 GO:0001530 1 8

C0030554 GO:0001838 1 8

C0030554 GO:0010595 1 8

C0030554 GO:0032318 1 8

C0030554 GO:0043197 1 8

C0030554 GO:0030004 1 8

C0030554 GO:0043010 1 8

C0030554 GO:0016338 2 8

C0030554 GO:0050995 1 8

C0030554 GO:0050994 1 8

C0030554 GO:0032320 1 8

C0030554 GO:0032321 1 8

C0030554 GO:0030900 1 8

C0030554 GO:0002250 1 8

C0030554 GO:0015749 1 8

C0030554 GO:0008645 1 8

C0030554 GO:0060538 2 8

C0030554 GO:0001654 2 8

C0030554 GO:0005161 1 8

C0030554 GO:0001937 1 8

C0031350 GO:0042133 1 4

C0031350 GO:0051044 1 4

C0031350 GO:0030228 1 4

C0031350 GO:0043954 1 4

C0031350 GO:0030427 1 4

C0031350 GO:0030426 1 4

C0031350 GO:0006477 1 4

C0031350 GO:0007292 1 4

C0031350 GO:0050926 1 4

C0031350 GO:0050927 1 4

C0031350 GO:0016863 1 4

C0031350 GO:0016860 1 4

C0031350 GO:0015085 1 4

C0031350 GO:0015082 1 4

C0031350 GO:0043046 1 4

C0031350 GO:0002263 1 4

C0031350 GO:0018212 1 4

C0031350 GO:0006266 1 4

C0031350 GO:0043120 1 4

C0031350 GO:0060541 1 4

C0031350 GO:0070228 1 4

C0031350 GO:0004029 1 4

C0031350 GO:0030216 1 4

C0031350 GO:0007176 1 4

C0031350 GO:0043535 1 4

C0031350 GO:0046631 1 4

C0031350 GO:0010874 1 4

C0031350 GO:0010875 1 4

C0031350 GO:0018209 1 4

C0031350 GO:0016812 1 4

C0031350 GO:0006041 1 4

C0031350 GO:0002274 1 4

C0031350 GO:0006044 1 4

C0031350 GO:0051353 1 4

C0031350 GO:0001910 1 4

C0031350 GO:0005388 1 4

C0031350 GO:0045494 1 4

C0031350 GO:0032732 1 4

C0031350 GO:0007635 1 4

C0031350 GO:0051103 1 4

C0031350 GO:0008484 1 4

C0031350 GO:0048002 1 4

C0031350 GO:0031575 1 4

C0031350 GO:0002286 1 4

C0031350 GO:0045649 1 4

C0031350 GO:0002285 1 4

C0031350 GO:0006885 1 4

C0031350 GO:0008088 1 4

C0031350 GO:0002366 1 4

C0031350 GO:0045912 1 4

C0031350 GO:0005254 1 4

C0031350 GO:0019882 1 4

C0031350 GO:0019883 1 4

C0031350 GO:0009880 1 4

C0031350 GO:0050810 1 4

C0031350 GO:0030279 1 4

C0031350 GO:0045165 1 4

C0031350 GO:0008093 1 4

C0031350 GO:0045730 1 4

C0031350 GO:0005247 1 4

C0031350 GO:0048638 1 4

C0031350 GO:0033613 1 4

C0031350 GO:0005788 1 4

C0031350 GO:0045429 1 4

C0031350 GO:0051453 1 4

C0031350 GO:0042169 1 4

C0031350 GO:0009124 1 4

C0031350 GO:0032376 1 4

C0031350 GO:0032370 1 4

C0031350 GO:0032373 1 4

C0031350 GO:0002224 1 4

C0031350 GO:0008286 1 4

C0031350 GO:0008329 1 4

C0031350 GO:0030004 1 4

C0031350 GO:0001654 1 4

C0031350 GO:0010677 1 4

C0031350 GO:0005593 2 4

C0031350 GO:0002460 1 4

C0031350 GO:0017144 1 4

C0031350 GO:0019200 1 4

C0031350 GO:0018105 1 4

C0031350 GO:0030324 1 4

C0031350 GO:0005160 1 4

C0031350 GO:0016338 1 4

C0031350 GO:0042113 1 4

C0031350 GO:0018108 1 4

C0031350 GO:0042058 1 4

C0031350 GO:0051147 1 4

C0031350 GO:0030641 1 4

C0031350 GO:0015695 1 4

C0031350 GO:0010469 1 4

C0031350 GO:0051897 1 4

C0031350 GO:0048662 1 4

C0031350 GO:0045088 1 4

C0031350 GO:0048660 1 4

C0031350 GO:0042102 1 4

C0031350 GO:0055067 1 4

C0031350 GO:0002440 1 4

C0031350 GO:0007266 1 4

C0031350 GO:0001530 1 4

C0031350 GO:0010595 1 4

C0031350 GO:0010594 1 4

C0031350 GO:0043536 1 4

C0031350 GO:0051923 1 4

C0031350 GO:0002250 1 4

C0031350 GO:0006413 1 4

C0037199 GO:0042834 1 4

C0037199 GO:0006909 1 4

C0037199 GO:0007598 1 4

C0037199 GO:0043954 1 4

C0037199 GO:0042036 1 4

C0037199 GO:0051044 1 4

C0037199 GO:0048261 1 4

C0037199 GO:0016863 1 4

C0037199 GO:0016860 1 4

C0037199 GO:0006306 1 4

C0037199 GO:0006305 1 4

C0037199 GO:0006304 1 4

C0037199 GO:0006303 1 4

C0037199 GO:0019992 1 4

C0037199 GO:0018210 1 4

C0037199 GO:0004716 1 4

C0037199 GO:0016459 1 4

C0037199 GO:0005795 1 4

C0037199 GO:0016101 1 4

C0037199 GO:0060541 3 4

C0037199 GO:0030595 1 4

C0037199 GO:0045851 1 4

C0037199 GO:0004029 1 4

C0037199 GO:0007281 1 4

C0037199 GO:0008360 1 4

C0037199 GO:0001754 1 4

C0037199 GO:0030295 2 4

C0037199 GO:0016811 1 4

C0037199 GO:0070633 1 4

C0037199 GO:0016814 1 4

C0037199 GO:0003995 1 4

C0037199 GO:0010888 1 4

C0037199 GO:0060263 1 4

C0037199 GO:0048568 1 4

C0037199 GO:0009898 1 4

C0037199 GO:0010869 1 4

C0037199 GO:0030170 1 4

C0037199 GO:0000578 1 4

C0037199 GO:0010862 1 4

C0037199 GO:0030073 1 4

C0037199 GO:0003205 1 4

C0037199 GO:0003206 1 4

C0037199 GO:0042551 1 4

C0037199 GO:0006885 2 4

C0037199 GO:0003208 1 4

C0037199 GO:0043028 1 4

C0037199 GO:0046850 1 4

C0037199 GO:0006112 1 4

C0037199 GO:0045912 1 4

C0037199 GO:0048705 1 4

C0037199 GO:0019882 1 4

C0037199 GO:0070169 1 4

C0037199 GO:0070207 1 4

C0037199 GO:0031256 1 4

C0037199 GO:0070167 1 4

C0037199 GO:0043410 1 4

C0037199 GO:0002526 1 4

C0037199 GO:0050714 1 4

C0037199 GO:0035097 1 4

C0037199 GO:0070567 2 4

C0037199 GO:0048407 1 4

C0037199 GO:0006026 1 4

C0037199 GO:0006027 1 4

C0037199 GO:0015172 1 4

C0037199 GO:0001822 1 4

C0037199 GO:0009410 1 4

C0037199 GO:0051453 2 4

C0037199 GO:0051452 1 4

C0037199 GO:0051457 2 4

C0037199 GO:0050431 1 4

C0037199 GO:0045621 1 4

C0037199 GO:0005506 1 4

C0037199 GO:0032677 1 4

C0037199 GO:0055008 1 4

C0037199 GO:0048872 1 4

C0037199 GO:0034637 1 4

C0037199 GO:0006970 1 4

C0037199 GO:0010675 1 4

C0037199 GO:0010677 1 4

C0037199 GO:0033293 1 4

C0037199 GO:0008656 1 4

C0037199 GO:0043120 1 4

C0037199 GO:0004190 1 4

C0037199 GO:0016525 1 4

C0037199 GO:0017046 1 4

C0037199 GO:0045923 1 4

C0037199 GO:0015813 1 4

C0037199 GO:0015810 1 4

C0037199 GO:0019048 1 4

C0037199 GO:0030035 1 4

C0037199 GO:0018108 1 4

C0037199 GO:0060021 1 4

C0037199 GO:0042593 1 4

C0037199 GO:0050906 1 4

C0037199 GO:0050905 1 4

C0037199 GO:0045445 1 4

C0037199 GO:0007271 1 4

C0037199 GO:0050909 1 4

C0037199 GO:0010828 1 4

C0037199 GO:0006094 1 4

C0037199 GO:0042359 1 4

C0037199 GO:0033261 1 4

C0037199 GO:0055092 1 4

C0037199 GO:0045639 1 4

C0037199 GO:0045736 1 4

C0037199 GO:0006700 1 4

C0037199 GO:0004969 1 4

C0037199 GO:0042509 1 4

C0037199 GO:0010594 1 4

C0037199 GO:0010596 1 4

C0037199 GO:0043535 1 4

C0037199 GO:0016986 1 4

C0037199 GO:0050996 1 4

C0037199 GO:0035272 1 4

C0037199 GO:0009593 1 4

C0037199 GO:0007618 1 4

C0037199 GO:0007612 1 4

C0037199 GO:0007611 1 4

C0037199 GO:0007163 1 4

C0037199 GO:0019915 1 4

C0037199 GO:0045667 1 4

C0037199 GO:0045669 1 4

C0037199 GO:0030216 1 4

C0037199 GO:0051119 1 4

C0037199 GO:0030218 1 4

C0037199 GO:0051117 1 4

C0037199 GO:0048278 1 4

C0037199 GO:0016894 1 4

C0037199 GO:0051701 1 4

C0037199 GO:0042992 1 4

C0037199 GO:0046530 1 4

C0037199 GO:0004707 1 4

C0037199 GO:0016291 1 4

C0037199 GO:0005388 2 4

C0037199 GO:0016445 1 4

C0037199 GO:0005905 1 4

C0037199 GO:0006805 1 4

C0037199 GO:0005929 1 4

C0037199 GO:0050829 1 4

C0037199 GO:0034708 1 4

C0037199 GO:0002761 1 4

C0037199 GO:0051187 1 4

C0037199 GO:0046915 1 4

C0037199 GO:0030330 1 4

C0037199 GO:0008088 1 4

C0037199 GO:0045749 1 4

C0037199 GO:0004550 1 4

C0037199 GO:0050766 1 4

C0037199 GO:0031941 1 4

C0037199 GO:0007585 1 4

C0037199 GO:0020037 1 4

C0037199 GO:0006584 1 4

C0037199 GO:0050795 1 4

C0037199 GO:0003231 1 4

C0037199 GO:0043666 1 4

C0037199 GO:0034382 1 4

C0037199 GO:0032365 1 4

C0037199 GO:0043154 1 4

C0037199 GO:0046823 1 4

C0037199 GO:0003746 1 4

C0037199 GO:0006109 1 4

C0037199 GO:0002698 1 4

C0037199 GO:0006739 1 4

C0037199 GO:0006637 1 4

C0037199 GO:0009311 1 4

C0037199 GO:0070279 1 4

C0037199 GO:0042401 1 4

C0037199 GO:0007044 1 4

C0037199 GO:0001948 1 4

C0037199 GO:0050764 1 4

C0037199 GO:0005035 1 4

C0037199 GO:0005031 1 4

C0037199 GO:0070303 1 4

C0037199 GO:0006929 1 4

C0037199 GO:0005868 1 4

C0037199 GO:0032587 1 4

C0037199 GO:0010906 1 4

C0037199 GO:0043470 1 4

C0037199 GO:0043471 1 4

C0037199 GO:0030175 1 4

C0037199 GO:0045619 1 4

C0037199 GO:0034405 1 4

C0037199 GO:0048732 1 4

C0037199 GO:0009083 1 4

C0037199 GO:0030323 1 4

C0037199 GO:0030324 3 4

C0037199 GO:0050868 1 4

C0037199 GO:0055010 1 4

C0037199 GO:0045124 1 4

C0037199 GO:0051147 1 4

C0037199 GO:0048041 1 4

C0037199 GO:0051149 1 4

C0037199 GO:0001523 1 4

C0037199 GO:0031532 1 4

C0037199 GO:0004181 1 4

C0037199 GO:0004675 1 4

C0037199 GO:0030934 1 4

C0037199 GO:0043588 1 4

C0037199 GO:0045931 1 4

C0037199 GO:0045930 1 4

C0037199 GO:0050699 1 4

C0037199 GO:0046626 1 4

C0037199 GO:0055062 1 4

C0037199 GO:0055061 1 4

C0037199 GO:0055067 1 4

C0037199 GO:0043627 1 4

C0037199 GO:0016909 1 4

C0037199 GO:0043190 1 4

C0037199 GO:0005605 1 4

C0037199 GO:0042345 1 4

C0037199 GO:0030004 1 4

C0037199 GO:0032205 1 4

C0037199 GO:0044236 1 4

C0037199 GO:0055072 1 4

C0037199 GO:0060538 1 4

C0037199 GO:0010832 1 4

C0037199 GO:0045807 1 4

C0037199 GO:0032387 1 4

C0037199 GO:0019229 1 4

C0037199 GO:0006476 1 4

C0037199 GO:0055081 1 4

C0037199 GO:0030501 1 4

C0037199 GO:0015085 1 4

C0037199 GO:0015082 2 4

C0037199 GO:0042531 1 4

C0037199 GO:0070001 1 4

C0037199 GO:0032722 1 4

C0037199 GO:0070228 1 4

C0037199 GO:0032655 2 4

C0037199 GO:0032652 1 4

C0037199 GO:0003007 1 4

C0037199 GO:0007176 1 4

C0037199 GO:0042752 1 4

C0037199 GO:0043531 1 4

C0037199 GO:0048641 1 4

C0037199 GO:0002274 1 4

C0037199 GO:0060393 1 4

C0037199 GO:0002377 1 4

C0037199 GO:0001910 1 4

C0037199 GO:0016755 1 4

C0037199 GO:0004551 1 4

C0037199 GO:0045494 1 4

C0037199 GO:0030203 1 4

C0037199 GO:0030201 1 4

C0037199 GO:0051103 1 4

C0037199 GO:0007623 1 4

C0037199 GO:0042625 1 4

C0037199 GO:0042627 1 4

C0037199 GO:0090100 1 4

C0037199 GO:0043548 1 4

C0037199 GO:0031674 1 4

C0037199 GO:0005523 1 4

C0037199 GO:0002200 1 4

C0037199 GO:0005254 1 4

C0037199 GO:0006953 1 4

C0037199 GO:0043900 1 4

C0037199 GO:0042308 1 4

C0037199 GO:0030279 1 4

C0037199 GO:0050819 1 4

C0037199 GO:0044275 1 4

C0037199 GO:0008093 1 4

C0037199 GO:0001776 1 4

C0037199 GO:0044447 1 4

C0037199 GO:0010921 1 4

C0037199 GO:0045429 1 4

C0037199 GO:0045428 1 4

C0037199 GO:0046677 1 4

C0037199 GO:0003229 1 4

C0037199 GO:0035023 1 4

C0037199 GO:0008543 1 4

C0037199 GO:0030018 1 4

C0037199 GO:0051324 1 4

C0037199 GO:0050885 1 4

C0037199 GO:0042274 1 4

C0037199 GO:0009712 1 4

C0037199 GO:0015377 1 4

C0037199 GO:0019209 2 4

C0037199 GO:0031330 1 4

C0037199 GO:0000217 1 4

C0037199 GO:0005593 1 4

C0037199 GO:0050777 1 4

C0037199 GO:0050679 1 4

C0037199 GO:0007422 1 4

C0037199 GO:0005024 1 4

C0037199 GO:0001816 1 4

C0037199 GO:0022839 1 4

C0037199 GO:0032393 1 4

C0037199 GO:0005160 1 4

C0037199 GO:0005161 1 4

C0037199 GO:0042058 1 4

C0037199 GO:0005436 1 4

C0037199 GO:0043500 1 4

C0037199 GO:0030832 1 4

C0037199 GO:0006690 1 4

C0037199 GO:0045766 1 4

C0037199 GO:0045767 1 4

C0037199 GO:0004879 1 4

C0037199 GO:0046425 1 4

C0037199 GO:0030235 1 4

C0037199 GO:0008198 1 4

C0037199 GO:0034220 1 4

C0037199 GO:0003785 1 4

C0037199 GO:0034599 1 4

C0037199 GO:0019842 1 4

C0037199 GO:0030900 1 4

C0037199 GO:0004890 1 4

C0037199 GO:0016505 1 4

C0037199 GO:0007090 1 4

C0037199 GO:0035065 1 4

C0037199 GO:0033500 1 4

C0037199 GO:0006821 1 4

C0037199 GO:0050920 1 4

C0037199 GO:0030345 1 4

C0037199 GO:0051897 1 4

C0037199 GO:0007519 1 4

C0037199 GO:0044419 1 4

C0037199 GO:0006266 1 4

C0037199 GO:0045834 1 4

C0037199 GO:0010896 1 4

C0037199 GO:0010898 1 4

C0037199 GO:0032421 1 4

C0037199 GO:0032420 1 4

C0037199 GO:0005796 1 4

C0037199 GO:0032781 1 4

C0037199 GO:0030041 1 4

C0037199 GO:0030169 1 4

C0037199 GO:0043648 1 4

C0037199 GO:0007270 1 4

C0037199 GO:0015485 1 4

C0037199 GO:0006040 1 4

C0037199 GO:0045582 1 4

C0037199 GO:0030199 1 4

C0037199 GO:0030198 1 4

C0037199 GO:0060415 1 4

C0037199 GO:0005089 2 4

C0037199 GO:0005088 2 4

C0037199 GO:0007632 1 4

C0037199 GO:0007635 1 4

C0037199 GO:0005086 1 4

C0037199 GO:0030183 1 4

C0037199 GO:0006687 1 4

C0037199 GO:0048477 1 4

C0037199 GO:0008154 1 4

C0037199 GO:0050690 1 4

C0037199 GO:0043256 1 4

C0037199 GO:0042632 1 4

C0037199 GO:0042734 1 4

C0037199 GO:0004683 1 4

C0037199 GO:0031645 1 4

C0037199 GO:0045732 1 4

C0037199 GO:0045638 1 4

C0037199 GO:0045730 2 4

C0037199 GO:0030195 1 4

C0037199 GO:0005247 1 4

C0037199 GO:0015662 1 4

C0037199 GO:0007158 1 4

C0037199 GO:0050808 1 4

C0037199 GO:0004114 1 4

C0037199 GO:0004112 1 4

C0037199 GO:0034311 1 4

C0037199 GO:0048029 2 4

C0037199 GO:0007416 1 4

C0037199 GO:0001540 1 4

C0037199 GO:0006342 1 4

C0037199 GO:0000041 1 4

C0037199 GO:0015296 1 4

C0037199 GO:0015299 1 4

C0037199 GO:0015298 1 4

C0037199 GO:0002224 1 4

C0037199 GO:0015807 1 4

C0037199 GO:0015800 1 4

C0037199 GO:0010830 1 4

C0037199 GO:0018958 1 4

C0037199 GO:0055085 1 4

C0037199 GO:0045598 1 4

C0037199 GO:0007568 1 4

C0037199 GO:0043178 1 4

C0037199 GO:0042476 1 4

C0037199 GO:0045296 1 4

C0037199 GO:0030099 1 4

C0037199 GO:0045862 1 4

C0037199 GO:0017144 1 4

C0037199 GO:0019200 1 4

C0037199 GO:0019201 1 4

C0037199 GO:0030021 1 4

C0037199 GO:0014910 1 4

C0037199 GO:0046326 1 4

C0037199 GO:0000188 1 4

C0037199 GO:0046329 1 4

C0037199 GO:0060228 1 4

C0037199 GO:0030641 2 4

C0037199 GO:0070555 1 4

C0037199 GO:0042516 1 4

C0037199 GO:0042517 1 4

C0037199 GO:0006721 1 4

C0037199 GO:0010466 1 4

C0037199 GO:0070411 1 4

C0037199 GO:0010745 1 4

C0037199 GO:0010469 1 4

C0037199 GO:0010743 1 4

C0037199 GO:0034101 1 4

C0037199 GO:0034103 1 4

C0037199 GO:0048741 1 4

C0037199 GO:0048742 1 4

C0037199 GO:0042100 1 4

C0037199 GO:0042102 1 4

C0037199 GO:0043523 1 4

C0037199 GO:0002449 1 4

C0037199 GO:0005884 1 4

C0037199 GO:0044403 1 4

C0037199 GO:0002440 1 4

C0037199 GO:0045778 1 4

C0037199 GO:0033344 1 4

C0037199 GO:0002250 1 4

C0037199 GO:0015036 1 4

C0085593 GO:0051043 1 4

C0085593 GO:0035065 1 4

C0085593 GO:0043954 1 4

C0085593 GO:0031970 1 4

C0085593 GO:0008585 1 4

C0085593 GO:0031057 1 4

C0085593 GO:0019915 1 4

C0085593 GO:0051893 1 4

C0085593 GO:0031058 1 4

C0085593 GO:0048260 1 4

C0085593 GO:0030228 1 4

C0085593 GO:0015085 1 4

C0085593 GO:0043205 1 4

C0085593 GO:0015082 1 4

C0085593 GO:0007519 1 4

C0085593 GO:0055117 1 4

C0085593 GO:0070001 1 4

C0085593 GO:0006266 1 4

C0085593 GO:0010896 1 4

C0085593 GO:0010898 1 4

C0085593 GO:0046504 1 4

C0085593 GO:0010522 1 4

C0085593 GO:0060541 2 4

C0085593 GO:0032655 1 4

C0085593 GO:0004029 1 4

C0085593 GO:0030216 1 4

C0085593 GO:0005159 1 4

C0085593 GO:0005158 1 4

C0085593 GO:0034637 1 4

C0085593 GO:0007176 1 4

C0085593 GO:0046631 1 4

C0085593 GO:0010975 1 4

C0085593 GO:0030041 1 4

C0085593 GO:0030295 1 4

C0085593 GO:0002429 1 4

C0085593 GO:0005254 1 4

C0085593 GO:0002274 1 4

C0085593 GO:0016814 1 4

C0085593 GO:0001910 1 4

C0085593 GO:0004551 1 4

C0085593 GO:0005388 1 4

C0085593 GO:0045494 1 4

C0085593 GO:0030194 1 4

C0085593 GO:0005089 1 4

C0085593 GO:0005088 1 4

C0085593 GO:0060263 2 4

C0085593 GO:0007635 1 4

C0085593 GO:0008252 1 4

C0085593 GO:0051103 1 4

C0085593 GO:0005086 1 4

C0085593 GO:0019934 1 4

C0085593 GO:0050820 1 4

C0085593 GO:0000578 1 4

C0085593 GO:0000786 1 4

C0085593 GO:0046915 1 4

C0085593 GO:0006942 1 4

C0085593 GO:0006885 1 4

C0085593 GO:0043548 1 4

C0085593 GO:0008088 1 4

C0085593 GO:0006112 1 4

C0085593 GO:0051879 1 4

C0085593 GO:0048705 1 4

C0085593 GO:0046545 1 4

C0085593 GO:0005071 1 4

C0085593 GO:0008154 1 4

C0085593 GO:0008301 1 4

C0085593 GO:0048255 1 4

C0085593 GO:0046660 1 4

C0085593 GO:0004437 1 4

C0085593 GO:0002757 1 4

C0085593 GO:0042734 1 4

C0085593 GO:0050714 1 4

C0085593 GO:0033261 1 4

C0085593 GO:0045730 1 4

C0085593 GO:0045737 1 4

C0085593 GO:0005247 1 4

C0085593 GO:0042439 1 4

C0085593 GO:0008034 1 4

C0085593 GO:0021537 1 4

C0085593 GO:0046579 1 4

C0085593 GO:0009312 1 4

C0085593 GO:0043627 1 4

C0085593 GO:0031228 1 4

C0085593 GO:0009895 1 4

C0085593 GO:0046677 1 4

C0085593 GO:0048029 1 4

C0085593 GO:0051453 1 4

C0085593 GO:0009124 1 4

C0085593 GO:0045078 1 4

C0085593 GO:0043489 1 4

C0085593 GO:0045072 1 4

C0085593 GO:0046782 1 4

C0085593 GO:0002224 1 4

C0085593 GO:0015296 1 4

C0085593 GO:0031330 1 4

C0085593 GO:0000217 1 4

C0085593 GO:0030004 1 4

C0085593 GO:0015807 1 4

C0085593 GO:0002440 1 4

C0085593 GO:0009898 1 4

C0085593 GO:0046870 1 4

C0085593 GO:0060021 1 4

C0085593 GO:0004693 1 4

C0085593 GO:0050770 1 4

C0085593 GO:0004190 1 4

C0085593 GO:0005903 1 4

C0085593 GO:0045861 1 4

C0085593 GO:0017144 1 4

C0085593 GO:0051057 1 4

C0085593 GO:0019200 1 4

C0085593 GO:0030323 1 4

C0085593 GO:0030324 2 4

C0085593 GO:0019209 1 4

C0085593 GO:0042058 1 4

C0085593 GO:0032934 1 4

C0085593 GO:0034623 1 4

C0085593 GO:0030641 1 4

C0085593 GO:0032984 1 4

C0085593 GO:0004181 1 4

C0085593 GO:0045766 1 4

C0085593 GO:0070410 1 4

C0085593 GO:0010469 1 4

C0085593 GO:0010741 1 4

C0085593 GO:0005697 1 4

C0085593 GO:0050851 1 4

C0085593 GO:0050852 1 4

C0085593 GO:0006700 1 4

C0085593 GO:0042102 1 4

C0085593 GO:0009112 1 4

C0085593 GO:0055067 1 4

C0085593 GO:0015175 1 4

C0085593 GO:0051646 1 4

C0085593 GO:0007266 1 4

C0085593 GO:0034185 1 4

C0085593 GO:0043190 1 4

C0085593 GO:0005605 1 4

C0085593 GO:0042345 1 4

C0085593 GO:0045211 1 4

C0085593 GO:0050995 1 4

C0085593 GO:0050994 1 4

C0085593 GO:0060538 1 4

C0085593 GO:0001937 1 4

C0085593 GO:0015036 1 4

C0085649 GO:0051044 1 3

C0085649 GO:0030228 1 3

C0085649 GO:0048638 1 3

C0085649 GO:0043954 1 3

C0085649 GO:0032387 1 3

C0085649 GO:0021510 1 3

C0085649 GO:0006477 1 3

C0085649 GO:0050926 1 3

C0085649 GO:0050927 1 3

C0085649 GO:0043525 1 3

C0085649 GO:0046631 1 3

C0085649 GO:0051897 1 3

C0085649 GO:0016860 1 3

C0085649 GO:0015085 1 3

C0085649 GO:0015082 1 3

C0085649 GO:0043206 1 3

C0085649 GO:0002263 1 3

C0085649 GO:0006266 1 3

C0085649 GO:0004712 1 3

C0085649 GO:0010721 1 3

C0085649 GO:0043120 1 3

C0085649 GO:0060541 1 3

C0085649 GO:0070228 1 3

C0085649 GO:0004029 1 3

C0085649 GO:0030216 1 3

C0085649 GO:0031960 1 3

C0085649 GO:0007176 1 3

C0085649 GO:0043535 1 3

C0085649 GO:0005788 1 3

C0085649 GO:0042992 1 3

C0085649 GO:0010874 1 3

C0085649 GO:0010875 1 3

C0085649 GO:0016812 1 3

C0085649 GO:0002274 1 3

C0085649 GO:0051353 1 3

C0085649 GO:0001910 1 3

C0085649 GO:0005388 1 3

C0085649 GO:0004708 1 3

C0085649 GO:0045494 1 3

C0085649 GO:0021537 1 3

C0085649 GO:0016055 1 3

C0085649 GO:0007635 1 3

C0085649 GO:0051103 1 3

C0085649 GO:0048002 1 3

C0085649 GO:0016863 1 3

C0085649 GO:0031575 1 3

C0085649 GO:0002286 1 3

C0085649 GO:0043547 1 3

C0085649 GO:0002285 1 3

C0085649 GO:0006885 1 3

C0085649 GO:0008088 1 3

C0085649 GO:0002366 1 3

C0085649 GO:0045912 1 3

C0085649 GO:0005254 1 3

C0085649 GO:0019882 1 3

C0085649 GO:0019883 1 3

C0085649 GO:0009880 1 3

C0085649 GO:0005138 1 3

C0085649 GO:0050810 1 3

C0085649 GO:0042308 1 3

C0085649 GO:0045165 1 3

C0085649 GO:0007423 1 3

C0085649 GO:0046906 1 3

C0085649 GO:0045730 1 3

C0085649 GO:0046823 1 3

C0085649 GO:0005247 1 3

C0085649 GO:0043010 1 3

C0085649 GO:0032107 1 3

C0085649 GO:0032104 1 3

C0085649 GO:0045429 1 3

C0085649 GO:0018105 1 3

C0085649 GO:0032373 1 3

C0085649 GO:0051453 1 3

C0085649 GO:0043087 1 3

C0085649 GO:0009124 1 3

C0085649 GO:0032376 1 3

C0085649 GO:0032370 1 3

C0085649 GO:0050768 1 3

C0085649 GO:0002224 1 3

C0085649 GO:0042169 1 3

C0085649 GO:0015296 1 3

C0085649 GO:0008329 1 3

C0085649 GO:0015459 1 3

C0085649 GO:0030004 1 3

C0085649 GO:0001654 2 3

C0085649 GO:0001656 1 3

C0085649 GO:0010677 1 3

C0085649 GO:0005593 1 3

C0085649 GO:0002460 1 3

C0085649 GO:0017144 1 3

C0085649 GO:0019200 1 3

C0085649 GO:0051384 1 3

C0085649 GO:0030324 1 3

C0085649 GO:0016338 1 3

C0085649 GO:0008286 1 3

C0085649 GO:0042058 1 3

C0085649 GO:0043506 1 3

C0085649 GO:0030641 1 3

C0085649 GO:0015695 1 3

C0085649 GO:0010469 1 3

C0085649 GO:0043588 1 3

C0085649 GO:0048662 1 3

C0085649 GO:0048660 1 3

C0085649 GO:0048592 1 3

C0085649 GO:0048593 1 3

C0085649 GO:0042102 1 3

C0085649 GO:0032318 1 3

C0085649 GO:0055067 1 3

C0085649 GO:0001530 1 3

C0085649 GO:0010595 1 3

C0085649 GO:0010594 1 3

C0085649 GO:0043197 1 3

C0085649 GO:0043536 1 3

C0085649 GO:0051923 1 3

C0085649 GO:0032320 1 3

C0085649 GO:0030900 1 3

C0085649 GO:0002250 1 3

C0085649 GO:0006413 1 3

C0231218 GO:0042133 1 9

C0231218 GO:0005782 1 9

C0231218 GO:0035065 1 9

C0231218 GO:0043954 2 9

C0231218 GO:0009187 1 9

C0231218 GO:0046631 2 9

C0231218 GO:0009074 1 9

C0231218 GO:0009164 1 9

C0231218 GO:0055072 1 9

C0231218 GO:0001505 1 9

C0231218 GO:0031057 1 9

C0231218 GO:0008375 1 9

C0231218 GO:0048260 1 9

C0231218 GO:0045841 1 9

C0231218 GO:0016863 1 9

C0231218 GO:0030228 1 9

C0231218 GO:0015085 1 9

C0231218 GO:0006304 1 9

C0231218 GO:0045839 1 9

C0231218 GO:0015082 1 9

C0231218 GO:0055117 1 9

C0231218 GO:0006266 1 9

C0231218 GO:0010721 1 9

C0231218 GO:0031907 1 9

C0231218 GO:0046870 1 9

C0231218 GO:0050927 1 9

C0231218 GO:0010522 1 9

C0231218 GO:0060541 2 9

C0231218 GO:0032934 2 9

C0231218 GO:0004029 1 9

C0231218 GO:0030216 1 9

C0231218 GO:0032153 1 9

C0231218 GO:0032155 1 9

C0231218 GO:0051059 1 9

C0231218 GO:0046579 1 9

C0231218 GO:0007176 1 9

C0231218 GO:0007172 1 9

C0231218 GO:0010975 2 9

C0231218 GO:0045725 1 9

C0231218 GO:0030041 1 9

C0231218 GO:0030295 1 9

C0231218 GO:0050770 2 9

C0231218 GO:0002429 1 9

C0231218 GO:0048871 1 9

C0231218 GO:0010469 1 9

C0231218 GO:0002274 1 9

C0231218 GO:0050926 1 9

C0231218 GO:0016814 1 9

C0231218 GO:0001910 1 9

C0231218 GO:0046849 1 9

C0231218 GO:0045821 1 9

C0231218 GO:0051784 1 9

C0231218 GO:0019439 1 9

C0231218 GO:0005388 1 9

C0231218 GO:0033261 1 9

C0231218 GO:0045494 2 9

C0231218 GO:0005089 1 9

C0231218 GO:0005088 1 9

C0231218 GO:0015175 1 9

C0231218 GO:0060263 2 9

C0231218 GO:0007635 1 9

C0231218 GO:0042398 1 9

C0231218 GO:0008252 1 9

C0231218 GO:0051103 1 9

C0231218 GO:0005086 1 9

C0231218 GO:0032946 1 9

C0231218 GO:0000578 1 9

C0231218 GO:0005929 1 9

C0231218 GO:0009068 1 9

C0231218 GO:0048488 1 9

C0231218 GO:0003205 1 9

C0231218 GO:0031577 1 9

C0231218 GO:0003206 1 9

C0231218 GO:0016049 1 9

C0231218 GO:0090101 1 9

C0231218 GO:0006942 1 9

C0231218 GO:0006885 2 9

C0231218 GO:0043548 1 9

C0231218 GO:0008088 1 9

C0231218 GO:0008643 1 9

C0231218 GO:0006112 1 9

C0231218 GO:0006687 1 9

C0231218 GO:0045912 1 9

C0231218 GO:0005254 1 9

C0231218 GO:0004551 1 9

C0231218 GO:0048705 1 9

C0231218 GO:0071174 1 9

C0231218 GO:0071173 1 9

C0231218 GO:0005071 1 9

C0231218 GO:0048709 1 9

C0231218 GO:0033764 1 9

C0231218 GO:0070875 1 9

C0231218 GO:0008154 1 9

C0231218 GO:0008301 1 9

C0231218 GO:0043256 1 9

C0231218 GO:0030069 1 9

C0231218 GO:0002757 1 9

C0231218 GO:0042734 2 9

C0231218 GO:0009112 1 9

C0231218 GO:0015074 1 9

C0231218 GO:0017080 1 9

C0231218 GO:0045730 1 9

C0231218 GO:0045737 1 9

C0231218 GO:0005247 1 9

C0231218 GO:0042439 1 9

C0231218 GO:0008034 1 9

C0231218 GO:0042834 1 9

C0231218 GO:0005884 1 9

C0231218 GO:0006633 1 9

C0231218 GO:0008021 1 9

C0231218 GO:0015280 1 9

C0231218 GO:0006895 1 9

C0231218 GO:0000185 1 9

C0231218 GO:0009312 1 9

C0231218 GO:0014003 1 9

C0231218 GO:0031228 1 9

C0231218 GO:0042177 1 9

C0231218 GO:0048029 1 9

C0231218 GO:0051453 2 9

C0231218 GO:0042572 1 9

C0231218 GO:0043241 1 9

C0231218 GO:0035303 1 9

C0231218 GO:0009124 1 9

C0231218 GO:0000786 1 9

C0231218 GO:0050885 1 9

C0231218 GO:0001709 1 9

C0231218 GO:0050768 1 9

C0231218 GO:0045740 1 9

C0231218 GO:0002224 1 9

C0231218 GO:0046467 1 9

C0231218 GO:0005506 1 9

C0231218 GO:0017157 1 9

C0231218 GO:0042169 1 9

C0231218 GO:0015296 1 9

C0231218 GO:0051457 1 9

C0231218 GO:0046326 1 9

C0231218 GO:0030004 1 9

C0231218 GO:0030675 1 9

C0231218 GO:0009898 1 9

C0231218 GO:0010677 1 9

C0231218 GO:0060021 1 9

C0231218 GO:0050771 1 9

C0231218 GO:0050679 1 9

C0231218 GO:0050671 1 9

C0231218 GO:0005024 1 9

C0231218 GO:0008210 1 9

C0231218 GO:0001764 1 9

C0231218 GO:0045861 1 9

C0231218 GO:0017144 1 9

C0231218 GO:0051057 1 9

C0231218 GO:0019200 1 9

C0231218 GO:0030323 1 9

C0231218 GO:0030324 2 9

C0231218 GO:0045927 1 9

C0231218 GO:0042119 1 9

C0231218 GO:0005041 1 9

C0231218 GO:0019209 1 9

C0231218 GO:0042058 1 9

C0231218 GO:0032655 1 9

C0231218 GO:0034623 1 9

C0231218 GO:0043507 1 9

C0231218 GO:0006090 1 9

C0231218 GO:0010828 1 9

C0231218 GO:0030641 2 9

C0231218 GO:0050661 1 9

C0231218 GO:0043467 1 9

C0231218 GO:0070567 1 9

C0231218 GO:0004181 1 9

C0231218 GO:0006527 1 9

C0231218 GO:0006693 1 9

C0231218 GO:0006525 1 9

C0231218 GO:0002573 1 9

C0231218 GO:0004675 1 9

C0231218 GO:0006692 1 9

C0231218 GO:0070665 1 9

C0231218 GO:0030934 1 9

C0231218 GO:0070410 1 9

C0231218 GO:0015026 1 9

C0231218 GO:0005697 1 9

C0231218 GO:0016229 1 9

C0231218 GO:0050851 1 9

C0231218 GO:0050852 1 9

C0231218 GO:0006700 1 9

C0231218 GO:0032984 1 9

C0231218 GO:0042102 1 9

C0231218 GO:0015758 1 9

C0231218 GO:0009110 1 9

C0231218 GO:0055067 1 9

C0231218 GO:0045766 1 9

C0231218 GO:0043627 2 9

C0231218 GO:0043624 1 9

C0231218 GO:0034185 1 9

C0231218 GO:0016877 1 9

C0231218 GO:0043190 1 9

C0231218 GO:0005605 1 9

C0231218 GO:0042345 1 9

C0231218 GO:0034637 1 9

C0231218 GO:0019047 1 9

C0231218 GO:0003705 1 9

C0231218 GO:0016986 1 9

C0231218 GO:0030148 1 9

C0231218 GO:0015749 1 9

C0231218 GO:0008645 1 9

C0231218 GO:0007094 1 9

C0231218 GO:0043112 1 9

C0231218 GO:0015036 1 9

C0700590 GO:0051043 1 4

C0700590 GO:0005149 1 4

C0700590 GO:0043954 1 4

C0700590 GO:0031970 1 4

C0700590 GO:0007612 1 4

C0700590 GO:0021510 1 4

C0700590 GO:0008585 1 4

C0700590 GO:0007162 1 4

C0700590 GO:0019915 1 4

C0700590 GO:0051893 1 4

C0700590 GO:0031058 1 4

C0700590 GO:0002573 1 4

C0700590 GO:0016597 1 4

C0700590 GO:0015085 1 4

C0700590 GO:0006305 1 4

C0700590 GO:0043205 1 4

C0700590 GO:0015082 1 4

C0700590 GO:0030195 1 4

C0700590 GO:0007519 1 4

C0700590 GO:0070001 2 4

C0700590 GO:0006266 1 4

C0700590 GO:0045661 1 4

C0700590 GO:0010896 1 4

C0700590 GO:0010898 1 4

C0700590 GO:0046504 1 4

C0700590 GO:0060541 1 4

C0700590 GO:0004029 1 4

C0700590 GO:0030216 1 4

C0700590 GO:0005159 1 4

C0700590 GO:0005158 1 4

C0700590 GO:0007176 1 4

C0700590 GO:0043535 1 4

C0700590 GO:0010874 1 4

C0700590 GO:0010875 1 4

C0700590 GO:0019827 1 4

C0700590 GO:0005254 1 4

C0700590 GO:0002274 1 4

C0700590 GO:0051353 1 4

C0700590 GO:0002377 1 4

C0700590 GO:0001910 1 4

C0700590 GO:0030048 1 4

C0700590 GO:0005388 1 4

C0700590 GO:0045494 1 4

C0700590 GO:0030194 1 4

C0700590 GO:0021537 1 4

C0700590 GO:0007632 1 4

C0700590 GO:0009895 2 4

C0700590 GO:0007635 1 4

C0700590 GO:0051103 1 4

C0700590 GO:0019934 1 4

C0700590 GO:0050820 1 4

C0700590 GO:0043025 1 4

C0700590 GO:0046326 1 4

C0700590 GO:0051224 1 4

C0700590 GO:0030890 1 4

C0700590 GO:0046915 1 4

C0700590 GO:0006885 1 4

C0700590 GO:0043548 1 4

C0700590 GO:0003705 1 4

C0700590 GO:0008088 2 4

C0700590 GO:0046850 1 4

C0700590 GO:0006110 1 4

C0700590 GO:0045912 1 4

C0700590 GO:0051879 1 4

C0700590 GO:0046545 1 4

C0700590 GO:0020037 1 4

C0700590 GO:0006306 1 4

C0700590 GO:0032768 1 4

C0700590 GO:0050796 1 4

C0700590 GO:0048255 1 4

C0700590 GO:0048864 1 4

C0700590 GO:0046660 1 4

C0700590 GO:0050819 1 4

C0700590 GO:0004437 1 4

C0700590 GO:0009953 1 4

C0700590 GO:0050714 1 4

C0700590 GO:0030864 1 4

C0700590 GO:0070567 1 4

C0700590 GO:0045730 2 4

C0700590 GO:0043034 1 4

C0700590 GO:0005247 1 4

C0700590 GO:0043010 1 4

C0700590 GO:0042834 1 4

C0700590 GO:0003785 1 4

C0700590 GO:0032770 1 4

C0700590 GO:0019956 1 4

C0700590 GO:0010921 1 4

C0700590 GO:0016331 1 4

C0700590 GO:0051646 1 4

C0700590 GO:0005903 1 4

C0700590 GO:0046677 1 4

C0700590 GO:0032373 1 4

C0700590 GO:0051453 1 4

C0700590 GO:0051457 1 4

C0700590 GO:0045078 1 4

C0700590 GO:0043489 1 4

C0700590 GO:0032374 1 4

C0700590 GO:0032376 1 4

C0700590 GO:0032371 1 4

C0700590 GO:0032370 1 4

C0700590 GO:0045072 1 4

C0700590 GO:0030705 1 4

C0700590 GO:0046782 1 4

C0700590 GO:0002224 1 4

C0700590 GO:0005506 1 4

C0700590 GO:0044275 1 4

C0700590 GO:0006879 1 4

C0700590 GO:0031330 1 4

C0700590 GO:0000217 1 4

C0700590 GO:0045211 1 4

C0700590 GO:0015807 2 4

C0700590 GO:0010677 1 4

C0700590 GO:0008091 1 4

C0700590 GO:0001894 1 4

C0700590 GO:0004693 1 4

C0700590 GO:0004190 2 4

C0700590 GO:0034405 1 4

C0700590 GO:0045861 1 4

C0700590 GO:0017144 1 4

C0700590 GO:0019200 2 4

C0700590 GO:0030323 1 4

C0700590 GO:0030324 1 4

C0700590 GO:0005160 1 4

C0700590 GO:0005929 1 4

C0700590 GO:0042058 1 4

C0700590 GO:0007270 1 4

C0700590 GO:0010828 1 4

C0700590 GO:0030641 1 4

C0700590 GO:0045684 1 4

C0700590 GO:0032350 1 4

C0700590 GO:0042354 1 4

C0700590 GO:0006096 1 4

C0700590 GO:0070668 1 4

C0700590 GO:0010469 1 4

C0700590 GO:0010741 1 4

C0700590 GO:0015179 1 4

C0700590 GO:0034103 1 4

C0700590 GO:0021915 1 4

C0700590 GO:0048593 1 4

C0700590 GO:0042102 1 4

C0700590 GO:0055067 1 4

C0700590 GO:0002440 1 4

C0700590 GO:0007266 1 4

C0700590 GO:0010594 1 4

C0700590 GO:0030004 1 4

C0700590 GO:0005044 1 4

C0700590 GO:0050995 1 4

C0700590 GO:0050994 1 4

C0700590 GO:0045124 1 4

C0700590 GO:0060538 1 4

C0700590 GO:0001937 1 4

C0016382 GO:0032148 1 6

C0016382 GO:0005149 2 6

C0016382 GO:0050840 1 6

C0016382 GO:0046637 1 6

C0016382 GO:0046634 1 6

C0016382 GO:0007612 1 6

C0016382 GO:0021510 2 6

C0016382 GO:0046631 1 6

C0016382 GO:0016615 1 6

C0016382 GO:0007162 1 6

C0016382 GO:0050920 1 6

C0016382 GO:0001505 1 6

C0016382 GO:0031343 1 6

C0016382 GO:0048261 1 6

C0016382 GO:0016597 1 6

C0016382 GO:0045981 1 6

C0016382 GO:0006306 1 6

C0016382 GO:0006305 1 6

C0016382 GO:0048864 1 6

C0016382 GO:0005834 1 6

C0016382 GO:0002263 1 6

C0016382 GO:0005544 1 6

C0016382 GO:0070001 3 6

C0016382 GO:0002685 1 6

C0016382 GO:0045661 1 6

C0016382 GO:0010896 1 6

C0016382 GO:0010898 1 6

C0016382 GO:0032722 1 6

C0016382 GO:0033032 1 6

C0016382 GO:0016202 1 6

C0016382 GO:0032655 1 6

C0016382 GO:0014020 1 6

C0016382 GO:0032781 1 6

C0016382 GO:0005313 1 6

C0016382 GO:0001841 1 6

C0016382 GO:0002821 1 6

C0016382 GO:0030166 1 6

C0016382 GO:0035270 1 6

C0016382 GO:0046330 1 6

C0016382 GO:0006477 1 6

C0016382 GO:0003001 1 6

C0016382 GO:0006836 1 6

C0016382 GO:0030291 1 6

C0016382 GO:0043536 1 6

C0016382 GO:0001843 1 6

C0016382 GO:0010874 1 6

C0016382 GO:0010875 1 6

C0016382 GO:0019827 1 6

C0016382 GO:0055092 1 6

C0016382 GO:0034284 1 6

C0016382 GO:0006044 1 6

C0016382 GO:0005902 1 6

C0016382 GO:0051353 2 6

C0016382 GO:0016566 1 6

C0016382 GO:0002377 2 6

C0016382 GO:0004629 1 6

C0016382 GO:0030048 1 6

C0016382 GO:0043030 1 6

C0016382 GO:0043034 2 6

C0016382 GO:0050921 1 6

C0016382 GO:0045840 1 6

C0016382 GO:0007632 1 6

C0016382 GO:0009895 1 6

C0016382 GO:0045767 1 6

C0016382 GO:0032411 1 6

C0016382 GO:0004035 1 6

C0016382 GO:0032642 1 6

C0016382 GO:0000428 1 6

C0016382 GO:0032648 1 6

C0016382 GO:0006929 1 6

C0016382 GO:0048634 1 6

C0016382 GO:0005929 1 6

C0016382 GO:0048002 1 6

C0016382 GO:0010862 1 6

C0016382 GO:0051224 2 6

C0016382 GO:0045086 1 6

C0016382 GO:0035088 1 6

C0016382 GO:0002286 1 6

C0016382 GO:0051180 1 6

C0016382 GO:0015491 1 6

C0016382 GO:0002285 1 6

C0016382 GO:0043548 1 6

C0016382 GO:0009309 1 6

C0016382 GO:0005858 1 6

C0016382 GO:0002687 1 6

C0016382 GO:0002366 1 6

C0016382 GO:0006687 1 6

C0016382 GO:0006110 1 6

C0016382 GO:0005522 1 6

C0016382 GO:0006688 1 6

C0016382 GO:0043025 1 6

C0016382 GO:0008088 1 6

C0016382 GO:0019882 1 6

C0016382 GO:0048593 1 6

C0016382 GO:0051785 1 6

C0016382 GO:0020037 1 6

C0016382 GO:0055029 1 6

C0016382 GO:0032768 1 6

C0016382 GO:0035250 1 6

C0016382 GO:0019319 1 6

C0016382 GO:0050795 1 6

C0016382 GO:0050796 1 6

C0016382 GO:0060606 1 6

C0016382 GO:0030274 1 6

C0016382 GO:0042632 1 6

C0016382 GO:0030069 1 6

C0016382 GO:0050819 1 6

C0016382 GO:0042301 1 6

C0016382 GO:0044275 1 6

C0016382 GO:0008652 1 6

C0016382 GO:0002757 1 6

C0016382 GO:0009953 1 6

C0016382 GO:0008656 1 6

C0016382 GO:0015300 1 6

C0016382 GO:0002758 2 6

C0016382 GO:0050892 1 6

C0016382 GO:0030864 1 6

C0016382 GO:0030890 1 6

C0016382 GO:0002218 2 6

C0016382 GO:0045730 1 6

C0016382 GO:0015179 1 6

C0016382 GO:0048407 1 6

C0016382 GO:0003746 1 6

C0016382 GO:0043010 1 6

C0016382 GO:0015172 1 6

C0016382 GO:0003785 2 6

C0016382 GO:0019897 1 6

C0016382 GO:0032107 1 6

C0016382 GO:0032104 1 6

C0016382 GO:0030880 1 6

C0016382 GO:0019956 1 6

C0016382 GO:0010921 1 6

C0016382 GO:0015074 1 6

C0016382 GO:0032770 1 6

C0016382 GO:0019200 1 6

C0016382 GO:0035148 1 6

C0016382 GO:0006941 1 6

C0016382 GO:0006940 1 6

C0016382 GO:0031228 1 6

C0016382 GO:0019047 1 6

C0016382 GO:0007043 1 6

C0016382 GO:0032373 1 6

C0016382 GO:0044241 1 6

C0016382 GO:0008235 1 6

C0016382 GO:0035295 1 6

C0016382 GO:0043168 1 6

C0016382 GO:0050764 1 6

C0016382 GO:0030201 1 6

C0016382 GO:0050885 1 6

C0016382 GO:0032374 1 6

C0016382 GO:0045076 1 6

C0016382 GO:0032376 1 6

C0016382 GO:0032371 1 6

C0016382 GO:0032370 1 6

C0016382 GO:0030810 1 6

C0016382 GO:0030705 1 6

C0016382 GO:0016459 1 6

C0016382 GO:0022829 1 6

C0016382 GO:0070688 1 6

C0016382 GO:0002224 2 6

C0016382 GO:0060562 1 6

C0016382 GO:0045622 1 6

C0016382 GO:0002221 2 6

C0016382 GO:0001838 1 6

C0016382 GO:0015297 1 6

C0016382 GO:0016338 1 6

C0016382 GO:0006879 1 6

C0016382 GO:0046850 1 6

C0016382 GO:0005844 1 6

C0016382 GO:0015299 1 6

C0016382 GO:0015298 1 6

C0016382 GO:0045580 1 6

C0016382 GO:0043270 1 6

C0016382 GO:0004861 1 6

C0016382 GO:0046928 1 6

C0016382 GO:0045216 1 6

C0016382 GO:0008091 1 6

C0016382 GO:0001894 1 6

C0016382 GO:0010594 2 6

C0016382 GO:0051092 1 6

C0016382 GO:0030804 1 6

C0016382 GO:0051928 1 6

C0016382 GO:0030801 1 6

C0016382 GO:0045296 1 6

C0016382 GO:0003725 1 6

C0016382 GO:0004190 3 6

C0016382 GO:0045619 1 6

C0016382 GO:0032479 1 6

C0016382 GO:0034405 1 6

C0016382 GO:0043370 1 6

C0016382 GO:0045616 1 6

C0016382 GO:0005104 1 6

C0016382 GO:0016331 1 6

C0016382 GO:0030323 1 6

C0016382 GO:0042119 1 6

C0016382 GO:0046364 1 6

C0016382 GO:0005160 1 6

C0016382 GO:0050866 1 6

C0016382 GO:0017048 1 6

C0016382 GO:0003705 1 6

C0016382 GO:0043506 1 6

C0016382 GO:0032862 1 6

C0016382 GO:0007270 1 6

C0016382 GO:0009749 1 6

C0016382 GO:0006096 1 6

C0016382 GO:0045684 1 6

C0016382 GO:0045768 1 6

C0016382 GO:0044447 1 6

C0016382 GO:0031532 1 6

C0016382 GO:0042516 1 6

C0016382 GO:0002573 1 6

C0016382 GO:0016538 1 6

C0016382 GO:0032321 1 6

C0016382 GO:0045604 1 6

C0016382 GO:0070668 1 6

C0016382 GO:0031307 1 6

C0016382 GO:0010469 1 6

C0016382 GO:0030195 1 6

C0016382 GO:0034103 1 6

C0016382 GO:0021915 1 6

C0016382 GO:0048741 1 6

C0016382 GO:0009247 1 6

C0016382 GO:0034612 1 6

C0016382 GO:0007585 1 6

C0016382 GO:0000271 1 6

C0016382 GO:0042100 1 6

C0016382 GO:0016504 1 6

C0016382 GO:0032856 1 6

C0016382 GO:0016903 1 6

C0016382 GO:0046580 1 6

C0016382 GO:0007269 1 6

C0016382 GO:0031214 1 6

C0016382 GO:0002440 1 6

C0016382 GO:0034599 1 6

C0016382 GO:0051058 1 6

C0016382 GO:0007519 1 6

C0016382 GO:0009746 1 6

C0016382 GO:0007281 1 6

C0016382 GO:0000178 1 6

C0016382 GO:0010595 1 6

C0016382 GO:0051881 1 6

C0016382 GO:0040017 1 6

C0016382 GO:0030299 1 6

C0016382 GO:0005044 1 6

C0016382 GO:0032350 1 6

C0016382 GO:0051923 1 6

C0016382 GO:0043535 1 6

C0016382 GO:0045124 1 6

C0016382 GO:0031526 1 6

C0016382 GO:0042354 1 6

C0016382 GO:0015807 1 6

C0016382 GO:0002250 1 6

C0016382 GO:0060538 1 6

C0016382 GO:0016505 2 6

C0016382 GO:0008290 1 6

C0016382 GO:0005161 1 6

C0016382 GO:0048520 1 6

C0020040 GO:0033238 2 6

C0020040 GO:0032148 2 6

C0020040 GO:0050688 1 6

C0020040 GO:0035270 1 6

C0020040 GO:0050840 2 6

C0020040 GO:0000086 1 6

C0020040 GO:0022410 1 6

C0020040 GO:0050681 1 6

C0020040 GO:0021510 1 6

C0020040 GO:0046631 1 6

C0020040 GO:0010660 1 6

C0020040 GO:0031050 1 6

C0020040 GO:0043449 1 6

C0020040 GO:0050920 2 6

C0020040 GO:0001505 1 6

C0020040 GO:0060326 1 6

C0020040 GO:0031341 1 6

C0020040 GO:0046718 1 6

C0020040 GO:0046883 1 6

C0020040 GO:0016863 1 6

C0020040 GO:0045981 1 6

C0020040 GO:0005149 2 6

C0020040 GO:0005071 1 6

C0020040 GO:0005072 1 6

C0020040 GO:0030742 1 6

C0020040 GO:0043206 1 6

C0020040 GO:0005540 1 6

C0020040 GO:0042308 1 6

C0020040 GO:0031016 1 6

C0020040 GO:0002263 1 6

C0020040 GO:0005544 1 6

C0020040 GO:0050727 1 6

C0020040 GO:0009071 1 6

C0020040 GO:0070001 2 6

C0020040 GO:0046697 1 6

C0020040 GO:0048512 1 6

C0020040 GO:0002685 2 6

C0020040 GO:0030656 1 6

C0020040 GO:0006073 1 6

C0020040 GO:0010898 1 6

C0020040 GO:0045839 1 6

C0020040 GO:0004089 1 6

C0020040 GO:0032722 1 6

C0020040 GO:0051954 1 6

C0020040 GO:0002683 1 6

C0020040 GO:0030595 1 6

C0020040 GO:0032655 2 6

C0020040 GO:0043020 1 6

C0020040 GO:0043966 1 6

C0020040 GO:0051119 1 6

C0020040 GO:0032429 1 6

C0020040 GO:0032781 2 6

C0020040 GO:0005313 1 6

C0020040 GO:0015696 1 6

C0020040 GO:0050777 1 6

C0020040 GO:0007603 1 6

C0020040 GO:0002821 1 6

C0020040 GO:0030166 1 6

C0020040 GO:0030299 1 6

C0020040 GO:0046330 1 6

C0020040 GO:0045187 1 6

C0020040 GO:0007173 1 6

C0020040 GO:0006477 1 6

C0020040 GO:0003001 1 6

C0020040 GO:0006836 1 6

C0020040 GO:0030041 1 6

C0020040 GO:0042994 1 6

C0020040 GO:0030514 1 6

C0020040 GO:0043536 2 6

C0020040 GO:0070875 1 6

C0020040 GO:0030511 1 6

C0020040 GO:0030510 1 6

C0020040 GO:0006570 1 6

C0020040 GO:0005501 1 6

C0020040 GO:0031343 2 6

C0020040 GO:0050716 1 6

C0020040 GO:0001893 1 6

C0020040 GO:0034284 1 6

C0020040 GO:0060395 2 6

C0020040 GO:0045652 1 6

C0020040 GO:0010578 1 6

C0020040 GO:0007205 1 6

C0020040 GO:0005902 1 6

C0020040 GO:0051353 2 6

C0020040 GO:0010887 1 6

C0020040 GO:0002377 1 6

C0020040 GO:0048260 1 6

C0020040 GO:0045821 1 6

C0020040 GO:0051784 1 6

C0020040 GO:0030193 1 6

C0020040 GO:0043030 2 6

C0020040 GO:0032731 1 6

C0020040 GO:0030195 1 6

C0020040 GO:0050921 2 6

C0020040 GO:0045841 1 6

C0020040 GO:0000428 1 6

C0020040 GO:0007632 1 6

C0020040 GO:0034374 1 6

C0020040 GO:0004035 2 6

C0020040 GO:0032642 1 6

C0020040 GO:0006044 1 6

C0020040 GO:0008272 1 6

C0020040 GO:0071174 1 6

C0020040 GO:0080010 1 6

C0020040 GO:0046321 1 6

C0020040 GO:0046320 1 6

C0020040 GO:0042745 1 6

C0020040 GO:0042742 1 6

C0020040 GO:0005929 1 6

C0020040 GO:0048002 1 6

C0020040 GO:0005272 1 6

C0020040 GO:0032496 1 6

C0020040 GO:0010862 1 6

C0020040 GO:0030071 1 6

C0020040 GO:0034708 1 6

C0020040 GO:0007062 1 6

C0020040 GO:0042992 1 6

C0020040 GO:0031577 1 6

C0020040 GO:0002286 2 6

C0020040 GO:0051180 1 6

C0020040 GO:0015491 1 6

C0020040 GO:0002285 2 6

C0020040 GO:0004428 1 6

C0020040 GO:0045646 1 6

C0020040 GO:0050906 1 6

C0020040 GO:0019229 1 6

C0020040 GO:0005858 1 6

C0020040 GO:0005859 1 6

C0020040 GO:0070513 1 6

C0020040 GO:0002687 1 6

C0020040 GO:0002366 1 6

C0020040 GO:0006687 1 6

C0020040 GO:0045725 1 6

C0020040 GO:0045727 1 6

C0020040 GO:0010812 1 6

C0020040 GO:0007185 1 6

C0020040 GO:0035150 1 6

C0020040 GO:0050766 1 6

C0020040 GO:0019882 1 6

C0020040 GO:0007623 1 6

C0020040 GO:0071173 1 6

C0020040 GO:0055029 1 6

C0020040 GO:0005161 1 6

C0020040 GO:0035250 2 6

C0020040 GO:0042129 1 6

C0020040 GO:0019319 1 6

C0020040 GO:0050795 2 6

C0020040 GO:0060070 1 6

C0020040 GO:0032387 1 6

C0020040 GO:0030276 1 6

C0020040 GO:0030898 1 6

C0020040 GO:0030274 1 6

C0020040 GO:0046006 1 6

C0020040 GO:0050670 1 6

C0020040 GO:0050728 1 6

C0020040 GO:0050818 1 6

C0020040 GO:0010851 1 6

C0020040 GO:0042301 1 6

C0020040 GO:0044042 1 6

C0020040 GO:0002757 1 6

C0020040 GO:0009952 1 6

C0020040 GO:0032368 1 6

C0020040 GO:0015300 1 6

C0020040 GO:0050650 1 6

C0020040 GO:0050718 1 6

C0020040 GO:0002758 2 6

C0020040 GO:0010712 1 6

C0020040 GO:0050654 1 6

C0020040 GO:0006691 1 6

C0020040 GO:0002218 2 6

C0020040 GO:0006693 1 6

C0020040 GO:0016460 1 6

C0020040 GO:0046823 1 6

C0020040 GO:0010553 1 6

C0020040 GO:0042787 1 6

C0020040 GO:0048407 2 6

C0020040 GO:0006752 1 6

C0020040 GO:0030194 1 6

C0020040 GO:0015172 1 6

C0020040 GO:0005884 1 6

C0020040 GO:0032107 1 6

C0020040 GO:0032104 1 6

C0020040 GO:0042752 1 6

C0020040 GO:0030880 1 6

C0020040 GO:0021536 1 6

C0020040 GO:0007622 1 6

C0020040 GO:0032770 1 6

C0020040 GO:0051004 1 6

C0020040 GO:0030260 1 6

C0020040 GO:0031228 1 6

C0020040 GO:0018149 1 6

C0020040 GO:0007189 1 6

C0020040 GO:0052126 1 6

C0020040 GO:0044241 1 6

C0020040 GO:0051457 1 6

C0020040 GO:0019840 1 6

C0020040 GO:0044246 1 6

C0020040 GO:0043168 1 6

C0020040 GO:0050764 2 6

C0020040 GO:0050880 1 6

C0020040 GO:0030201 2 6

C0020040 GO:0050885 1 6

C0020040 GO:0005583 1 6

C0020040 GO:0034391 1 6

C0020040 GO:0032091 1 6

C0020040 GO:0007602 1 6

C0020040 GO:0001709 1 6

C0020040 GO:0030810 1 6

C0020040 GO:0007259 1 6

C0020040 GO:0051917 1 6

C0020040 GO:0004715 1 6

C0020040 GO:0022829 1 6

C0020040 GO:0032845 1 6

C0020040 GO:0002224 1 6

C0020040 GO:0002237 1 6

C0020040 GO:0002221 2 6

C0020040 GO:0001953 1 6

C0020040 GO:0005355 1 6

C0020040 GO:0043498 1 6

C0020040 GO:0015297 1 6

C0020040 GO:0008034 1 6

C0020040 GO:0010896 1 6

C0020040 GO:0008329 1 6

C0020040 GO:0015459 1 6

C0020040 GO:0035097 1 6

C0020040 GO:0015299 2 6

C0020040 GO:0015298 1 6

C0020040 GO:0032648 1 6

C0020040 GO:0043270 1 6

C0020040 GO:0046928 1 6

C0020040 GO:0004864 1 6

C0020040 GO:0014068 1 6

C0020040 GO:0006692 1 6

C0020040 GO:0004890 1 6

C0020040 GO:0042562 1 6

C0020040 GO:0051983 1 6

C0020040 GO:0010594 2 6

C0020040 GO:0051092 1 6

C0020040 GO:0043470 1 6

C0020040 GO:0043471 1 6

C0020040 GO:0010579 1 6

C0020040 GO:0050820 1 6

C0020040 GO:0030804 1 6

C0020040 GO:0046580 2 6

C0020040 GO:0051928 1 6

C0020040 GO:0030801 1 6

C0020040 GO:0050671 1 6

C0020040 GO:0045296 1 6

C0020040 GO:0015385 1 6

C0020040 GO:0001816 1 6

C0020040 GO:0051828 1 6

C0020040 GO:0002020 1 6

C0020040 GO:0004190 2 6

C0020040 GO:0032479 1 6

C0020040 GO:0009084 1 6

C0020040 GO:0005518 1 6

C0020040 GO:0030426 1 6

C0020040 GO:0048385 1 6

C0020040 GO:0030249 1 6

C0020040 GO:0051384 1 6

C0020040 GO:0042119 1 6

C0020040 GO:0042116 1 6

C0020040 GO:0046364 1 6

C0020040 GO:0045582 1 6

C0020040 GO:0050866 1 6

C0020040 GO:0010744 1 6

C0020040 GO:0050829 1 6

C0020040 GO:0051018 1 6

C0020040 GO:0009108 1 6

C0020040 GO:0050892 1 6

C0020040 GO:0034235 1 6

C0020040 GO:0030425 1 6

C0020040 GO:0009749 1 6

C0020040 GO:0004972 1 6

C0020040 GO:0044447 1 6

C0020040 GO:0031532 1 6

C0020040 GO:0051739 1 6

C0020040 GO:0042516 1 6

C0020040 GO:0032430 1 6

C0020040 GO:0045766 1 6

C0020040 GO:0005513 1 6

C0020040 GO:0070412 1 6

C0020040 GO:0042749 1 6

C0020040 GO:0070410 1 6

C0020040 GO:0031960 1 6

C0020040 GO:0033189 1 6

C0020040 GO:0031307 1 6

C0020040 GO:0070918 1 6

C0020040 GO:0010596 1 6

C0020040 GO:0010743 1 6

C0020040 GO:0045931 1 6

C0020040 GO:0032526 1 6

C0020040 GO:0019212 1 6

C0020040 GO:0048662 1 6

C0020040 GO:0007200 1 6

C0020040 GO:0048660 1 6

C0020040 GO:0090068 1 6

C0020040 GO:0048741 1 6

C0020040 GO:0042104 1 6

C0020040 GO:0034612 1 6

C0020040 GO:0007585 1 6

C0020040 GO:0000271 1 6

C0020040 GO:0042100 2 6

C0020040 GO:0042102 1 6

C0020040 GO:0002449 1 6

C0020040 GO:0016903 1 6

C0020040 GO:0042026 1 6

C0020040 GO:0031047 1 6

C0020040 GO:0007269 1 6

C0020040 GO:0002440 1 6

C0020040 GO:0034599 1 6

C0020040 GO:0051058 2 6

C0020040 GO:0005451 1 6

C0020040 GO:0003746 1 6

C0020040 GO:0009746 1 6

C0020040 GO:0000178 2 6

C0020040 GO:0010595 2 6

C0020040 GO:0051881 1 6

C0020040 GO:0040017 2 6

C0020040 GO:0042476 1 6

C0020040 GO:0031998 1 6

C0020040 GO:0035195 1 6

C0020040 GO:0035196 1 6

C0020040 GO:0045648 1 6

C0020040 GO:0051923 1 6

C0020040 GO:0052192 1 6

C0020040 GO:0031526 1 6

C0020040 GO:0050819 1 6

C0020040 GO:0005577 1 6

C0020040 GO:0044409 1 6

C0020040 GO:0002250 2 6

C0020040 GO:0007094 1 6

C0020040 GO:0016505 1 6

C0020040 GO:0051806 1 6

C0020040 GO:0008290 1 6

C0020040 GO:0006940 1 6

C0020040 GO:0048520 2 6

C0151825 GO:0032107 1 2

C0151825 GO:0032104 1 2

C0151825 GO:0050808 1 2

C0151825 GO:0007269 1 2

C0151825 GO:0030801 1 2

C0151825 GO:0048145 1 2

C0151825 GO:0050866 1 2

C0151825 GO:0015297 1 2

C0151825 GO:0046631 1 2

C0151825 GO:0046112 1 2

C0151825 GO:0001505 1 2

C0151825 GO:0045296 1 2

C0151825 GO:0048002 1 2

C0151825 GO:0044241 1 2

C0151825 GO:0009749 1 2

C0151825 GO:0016863 1 2

C0151825 GO:0045981 1 2

C0151825 GO:0002286 1 2

C0151825 GO:0050885 1 2

C0151825 GO:0002285 1 2

C0151825 GO:0031532 1 2

C0151825 GO:0002263 1 2

C0151825 GO:0050700 1 2

C0151825 GO:0016830 1 2

C0151825 GO:0030810 1 2

C0151825 GO:0070001 1 2

C0151825 GO:0009112 1 2

C0151825 GO:0002366 1 2

C0151825 GO:0006687 1 2

C0151825 GO:0045727 1 2

C0151825 GO:0002221 1 2

C0151825 GO:0019882 1 2

C0151825 GO:0008034 1 2

C0151825 GO:0000271 1 2

C0151825 GO:0015299 1 2

C0151825 GO:0015298 1 2

C0151825 GO:0015929 1 2

C0151825 GO:0033483 1 2

C0151825 GO:0030299 1 2

C0151825 GO:0032148 1 2

C0151825 GO:0003001 1 2

C0151825 GO:0006836 1 2

C0151825 GO:0030169 1 2

C0151825 GO:0009746 1 2

C0151825 GO:0002757 1 2

C0151825 GO:0015491 1 2

C0151825 GO:0015300 1 2

C0151825 GO:0030804 1 2

C0151825 GO:0032364 1 2

C0151825 GO:0034284 1 2

C0151825 GO:0002758 1 2

C0151825 GO:0050892 1 2

C0151825 GO:0002793 1 2

C0151825 GO:0002218 1 2

C0151825 GO:0046887 1 2

C0151825 GO:0004190 1 2

C0151825 GO:0048407 1 2

C0151825 GO:0005518 1 2

C0030552 GO:0032148 1 3

C0030552 GO:0003001 1 3

C0030552 GO:0007286 1 3

C0030552 GO:0046631 1 3

C0030552 GO:0002703 1 3

C0030552 GO:0001505 1 3

C0030552 GO:0034199 1 3

C0030552 GO:0030345 1 3

C0030552 GO:0008374 1 3

C0030552 GO:0031343 1 3

C0030552 GO:0016597 1 3

C0030552 GO:0045981 1 3

C0030552 GO:0002263 1 3

C0030552 GO:0030295 1 3

C0030552 GO:0070001 1 3

C0030552 GO:0006073 1 3

C0030552 GO:0002822 1 3

C0030552 GO:0007281 1 3

C0030552 GO:0030299 1 3

C0030552 GO:0008170 1 3

C0030552 GO:0010975 1 3

C0030552 GO:0006836 1 3

C0030552 GO:0030512 1 3

C0030552 GO:0030296 1 3

C0030552 GO:0030169 1 3

C0030552 GO:0001910 1 3

C0030552 GO:0019320 1 3

C0030552 GO:0048002 1 3

C0030552 GO:0021700 1 3

C0030552 GO:0007530 1 3

C0030552 GO:0002286 1 3

C0030552 GO:0015491 1 3

C0030552 GO:0002285 1 3

C0030552 GO:0002366 1 3

C0030552 GO:0006687 1 3

C0030552 GO:0048641 1 3

C0030552 GO:0030879 1 3

C0030552 GO:0002688 1 3

C0030552 GO:0019882 1 3

C0030552 GO:0032769 1 3

C0030552 GO:0005138 1 3

C0030552 GO:0030069 1 3

C0030552 GO:0005930 1 3

C0030552 GO:0042301 1 3

C0030552 GO:0044042 1 3

C0030552 GO:0002757 1 3

C0030552 GO:0050885 1 3

C0030552 GO:0050715 1 3

C0030552 GO:0015300 1 3

C0030552 GO:0031645 1 3

C0030552 GO:0002758 1 3

C0030552 GO:0050892 1 3

C0030552 GO:0002218 1 3

C0030552 GO:0045730 1 3

C0030552 GO:0002690 1 3

C0030552 GO:0048407 1 3

C0030552 GO:0032107 1 3

C0030552 GO:0032104 1 3

C0030552 GO:0044241 1 3

C0030552 GO:0008543 1 3

C0030552 GO:0007250 1 3

C0030552 GO:0030810 1 3

C0030552 GO:0031397 1 3

C0030552 GO:0002221 1 3

C0030552 GO:0044275 1 3

C0030552 GO:0015297 1 3

C0030552 GO:0019209 1 3

C0030552 GO:0015299 1 3

C0030552 GO:0015298 1 3

C0030552 GO:0042269 1 3

C0030552 GO:0050770 1 3

C0030552 GO:0030804 1 3

C0030552 GO:0030801 1 3

C0030552 GO:0045296 1 3

C0030552 GO:0004190 1 3

C0030552 GO:0006007 1 3

C0030552 GO:0019047 1 3

C0030552 GO:0050868 1 3

C0030552 GO:0046365 1 3

C0030552 GO:0050866 2 3

C0030552 GO:0017048 1 3

C0030552 GO:0045682 1 3

C0030552 GO:0015074 1 3

C0030552 GO:0031532 1 3

C0030552 GO:0048742 1 3

C0030552 GO:0000271 1 3

C0030552 GO:0030021 1 3

C0030552 GO:0002715 1 3

C0030552 GO:0007269 1 3

C0030552 GO:0031214 1 3

C0030552 GO:0051181 1 3

C0030552 GO:0005977 1 3

C0021311 GO:0005786 1 8

C0021311 GO:0006775 1 8

C0021311 GO:0050681 1 8

C0021311 GO:0030425 1 8

C0021311 GO:0048385 1 8

C0021311 GO:0050926 2 8

C0021311 GO:0015645 1 8

C0021311 GO:0043524 1 8

C0021311 GO:0002702 2 8

C0021311 GO:0008272 1 8

C0021311 GO:0001508 1 8

C0021311 GO:0008375 1 8

C0021311 GO:0046718 1 8

C0021311 GO:0010906 1 8

C0021311 GO:0000786 1 8

C0021311 GO:0051896 1 8

C0021311 GO:0051453 1 8

C0021311 GO:0001894 1 8

C0021311 GO:0005071 1 8

C0021311 GO:0005072 1 8

C0021311 GO:0051205 1 8

C0021311 GO:0000002 1 8

C0021311 GO:0033613 1 8

C0021311 GO:0042605 1 8

C0021311 GO:0005540 1 8

C0021311 GO:0004407 1 8

C0021311 GO:0043043 1 8

C0021311 GO:0030295 1 8

C0021311 GO:0045667 1 8

C0021311 GO:0004715 1 8

C0021311 GO:0045834 1 8

C0021311 GO:0010898 1 8

C0021311 GO:0045839 2 8

C0021311 GO:0009190 1 8

C0021311 GO:0045669 1 8

C0021311 GO:0006171 1 8

C0021311 GO:0060541 1 8

C0021311 GO:0016202 1 8

C0021311 GO:0030595 1 8

C0021311 GO:0004364 1 8

C0021311 GO:0043966 1 8

C0021311 GO:0030217 1 8

C0021311 GO:0030218 1 8

C0021311 GO:0006304 2 8

C0021311 GO:0006769 1 8

C0021311 GO:0051059 1 8

C0021311 GO:0003007 1 8

C0021311 GO:0001516 1 8

C0021311 GO:0046965 1 8

C0021311 GO:0035272 1 8

C0021311 GO:0007173 1 8

C0021311 GO:0048641 1 8

C0021311 GO:0018209 1 8

C0021311 GO:0030514 1 8

C0021311 GO:0070875 2 8

C0021311 GO:0030510 1 8

C0021311 GO:0042088 1 8

C0021311 GO:0060395 1 8

C0021311 GO:0060393 1 8

C0021311 GO:0016566 1 8

C0021311 GO:0004629 1 8

C0021311 GO:0051784 2 8

C0021311 GO:0019439 1 8

C0021311 GO:0033558 1 8

C0021311 GO:0033261 2 8

C0021311 GO:0004709 1 8

C0021311 GO:0010466 1 8

C0021311 GO:0045494 1 8

C0021311 GO:0004033 1 8

C0021311 GO:0030206 1 8

C0021311 GO:0009895 1 8

C0021311 GO:0043087 1 8

C0021311 GO:0060326 1 8

C0021311 GO:0021915 1 8

C0021311 GO:0009898 1 8

C0021311 GO:0050821 1 8

C0021311 GO:0046658 2 8

C0021311 GO:0043025 1 8

C0021311 GO:0001764 2 8

C0021311 GO:0046496 1 8

C0021311 GO:0005929 1 8

C0021311 GO:0030286 1 8

C0021311 GO:0010862 1 8

C0021311 GO:0051224 1 8

C0021311 GO:0030071 1 8

C0021311 GO:0048488 2 8

C0021311 GO:0007062 2 8

C0021311 GO:0031577 2 8

C0021311 GO:0045058 2 8

C0021311 GO:0004428 2 8

C0021311 GO:0045646 1 8

C0021311 GO:0046425 1 8

C0021311 GO:0015149 1 8

C0021311 GO:0009880 1 8

C0021311 GO:0006110 1 8

C0021311 GO:0045725 2 8

C0021311 GO:0015145 1 8

C0021311 GO:0051131 1 8

C0021311 GO:0071174 2 8

C0021311 GO:0030235 1 8

C0021311 GO:0015909 1 8

C0021311 GO:0071173 2 8

C0021311 GO:0015085 1 8

C0021311 GO:0050714 1 8

C0021311 GO:0031256 1 8

C0021311 GO:0030371 1 8

C0021311 GO:0048500 1 8

C0021311 GO:0006584 1 8

C0021311 GO:0008013 1 8

C0021311 GO:0030374 1 8

C0021311 GO:0008154 1 8

C0021311 GO:0060070 2 8

C0021311 GO:0014031 1 8

C0021311 GO:0030276 1 8

C0021311 GO:0048255 1 8

C0021311 GO:0030275 1 8

C0021311 GO:0035303 1 8

C0021311 GO:0043489 1 8

C0021311 GO:0043410 1 8

C0021311 GO:0004435 1 8

C0021311 GO:0004434 1 8

C0021311 GO:0009952 1 8

C0021311 GO:0008093 1 8

C0021311 GO:0046906 1 8

C0021311 GO:0016409 1 8

C0021311 GO:0050650 1 8

C0021311 GO:0050654 1 8

C0021311 GO:0030663 1 8

C0021311 GO:0043154 1 8

C0021311 GO:0006693 1 8

C0021311 GO:0006692 1 8

C0021311 GO:0045737 1 8

C0021311 GO:0010889 1 8

C0021311 GO:0043393 1 8

C0021311 GO:0006518 1 8

C0021311 GO:0043010 1 8

C0021311 GO:0045841 2 8

C0021311 GO:0001709 3 8

C0021311 GO:0030204 1 8

C0021311 GO:0016790 1 8

C0021311 GO:0030159 1 8

C0021311 GO:0048146 1 8

C0021311 GO:0008206 1 8

C0021311 GO:0048634 1 8

C0021311 GO:0050804 1 8

C0021311 GO:0034311 1 8

C0021311 GO:0030260 1 8

C0021311 GO:0045429 1 8

C0021311 GO:0045428 1 8

C0021311 GO:0048029 1 8

C0021311 GO:0016909 1 8

C0021311 GO:0052126 1 8

C0021311 GO:0051452 1 8

C0021311 GO:0033280 2 8

C0021311 GO:0031513 1 8

C0021311 GO:0008543 1 8

C0021311 GO:0009124 1 8

C0021311 GO:0031225 2 8

C0021311 GO:0051806 1 8

C0021311 GO:0007127 1 8

C0021311 GO:0007250 1 8

C0021311 GO:0016709 1 8

C0021311 GO:0045076 1 8

C0021311 GO:0010563 1 8

C0021311 GO:0008308 1 8

C0021311 GO:0045833 1 8

C0021311 GO:0042572 1 8

C0021311 GO:0002020 1 8

C0021311 GO:0022612 1 8

C0021311 GO:0005506 1 8

C0021311 GO:0043534 1 8

C0021311 GO:0005355 1 8

C0021311 GO:0042169 1 8

C0021311 GO:0042119 1 8

C0021311 GO:0045740 1 8

C0021311 GO:0005844 1 8

C0021311 GO:0006721 1 8

C0021311 GO:0006720 1 8

C0021311 GO:0031333 2 8

C0021311 GO:0045086 1 8

C0021311 GO:0035035 1 8

C0021311 GO:0007163 1 8

C0021311 GO:0043124 1 8

C0021311 GO:0030675 1 8

C0021311 GO:0006929 1 8

C0021311 GO:0045216 1 8

C0021311 GO:0016101 1 8

C0021311 GO:0018958 1 8

C0021311 GO:0007131 1 8

C0021311 GO:0030126 1 8

C0021311 GO:0001893 1 8

C0021311 GO:0051983 1 8

C0021311 GO:0051881 1 8

C0021311 GO:0042625 1 8

C0021311 GO:0043470 1 8

C0021311 GO:0043471 1 8

C0021311 GO:0050771 1 8

C0021311 GO:0007569 1 8

C0021311 GO:0043603 1 8

C0021311 GO:0015884 1 8

C0021311 GO:0007422 1 8

C0021311 GO:0045296 1 8

C0021311 GO:0001950 1 8

C0021311 GO:0051828 1 8

C0021311 GO:0002237 1 8

C0021311 GO:0045861 1 8

C0021311 GO:0004745 1 8

C0021311 GO:0005391 1 8

C0021311 GO:0030324 1 8

C0021311 GO:0030326 1 8

C0021311 GO:0048762 1 8

C0021311 GO:0042177 1 8

C0021311 GO:0016339 1 8

C0021311 GO:0019209 1 8

C0021311 GO:0018108 1 8

C0021311 GO:0003705 1 8

C0021311 GO:0045884 1 8

C0021311 GO:0045851 1 8

C0021311 GO:0009108 1 8

C0021311 GO:0046890 1 8

C0021311 GO:0045445 1 8

C0021311 GO:0002724 2 8

C0021311 GO:0001837 1 8

C0021311 GO:0001523 1 8

C0021311 GO:0030641 1 8

C0021311 GO:0070491 1 8

C0021311 GO:0045768 1 8

C0021311 GO:0032355 1 8

C0021311 GO:0050660 2 8

C0021311 GO:0008443 1 8

C0021311 GO:0043467 1 8

C0021311 GO:0046456 1 8

C0021311 GO:0002720 2 8

C0021311 GO:0050927 2 8

C0021311 GO:0031330 1 8

C0021311 GO:0004467 1 8

C0021311 GO:0070412 2 8

C0021311 GO:0030137 1 8

C0021311 GO:0070410 2 8

C0021311 GO:0005159 1 8

C0021311 GO:0019213 1 8

C0021311 GO:0045089 1 8

C0021311 GO:0032587 1 8

C0021311 GO:0045936 1 8

C0021311 GO:0007584 1 8

C0021311 GO:0048742 1 8

C0021311 GO:0050690 1 8

C0021311 GO:0042581 1 8

C0021311 GO:0006895 1 8

C0021311 GO:0018212 1 8

C0021311 GO:0001727 1 8

C0021311 GO:0035113 1 8

C0021311 GO:0002718 2 8

C0021311 GO:0060485 1 8

C0021311 GO:0002443 1 8

C0021311 GO:0015662 1 8

C0021311 GO:0042509 1 8

C0021311 GO:0005605 1 8

C0021311 GO:0009820 1 8

C0021311 GO:0042345 1 8

C0021311 GO:0030004 1 8

C0021311 GO:0008517 2 8

C0021311 GO:0005041 1 8

C0021311 GO:0052192 1 8

C0021311 GO:0031526 1 8

C0021311 GO:0046457 1 8

C0021311 GO:0016801 1 8

C0021311 GO:0044409 1 8

C0021311 GO:0045767 1 8

C0021311 GO:0007094 2 8

C0021311 GO:0009712 1 8

C0021311 GO:0046697 1 8

C0021311 GO:0019362 1 8

C0013604 GO:0060249 1 5

C0013604 GO:0000086 1 5

C0013604 GO:0043954 1 5

C0013604 GO:0048771 1 5

C0013604 GO:0000080 1 5

C0013604 GO:0009164 1 5

C0013604 GO:0043523 1 5

C0013604 GO:0043524 1 5

C0013604 GO:0008375 1 5

C0013604 GO:0004675 1 5

C0013604 GO:0006304 1 5

C0013604 GO:0019992 1 5

C0013604 GO:0018210 1 5

C0013604 GO:0043325 1 5

C0013604 GO:0045839 1 5

C0013604 GO:0051059 1 5

C0013604 GO:0007281 1 5

C0013604 GO:0045725 1 5

C0013604 GO:0070875 1 5

C0013604 GO:0009072 1 5

C0013604 GO:0010875 1 5

C0013604 GO:0050926 1 5

C0013604 GO:0060393 1 5

C0013604 GO:0050927 1 5

C0013604 GO:0046849 1 5

C0013604 GO:0045821 1 5

C0013604 GO:0051784 1 5

C0013604 GO:0045494 1 5

C0013604 GO:0045841 1 5

C0013604 GO:0042398 1 5

C0013604 GO:0001764 1 5

C0013604 GO:0005929 1 5

C0013604 GO:0048488 1 5

C0013604 GO:0031577 1 5

C0013604 GO:0016049 1 5

C0013604 GO:0008645 1 5

C0013604 GO:0050700 1 5

C0013604 GO:0008643 1 5

C0013604 GO:0043028 1 5

C0013604 GO:0045912 1 5

C0013604 GO:0004143 1 5

C0013604 GO:0030879 1 5

C0013604 GO:0043027 1 5

C0013604 GO:0071174 1 5

C0013604 GO:0071173 1 5

C0013604 GO:0005138 1 5

C0013604 GO:0030675 1 5

C0013604 GO:0008091 1 5

C0013604 GO:0042734 1 5

C0013604 GO:0050714 1 5

C0013604 GO:0070567 1 5

C0013604 GO:0017080 2 5

C0013604 GO:0006692 1 5

C0013604 GO:0042834 1 5

C0013604 GO:0006633 1 5

C0013604 GO:0008021 1 5

C0013604 GO:0001709 1 5

C0013604 GO:0017022 1 5

C0013604 GO:0051453 1 5

C0013604 GO:0009798 1 5

C0013604 GO:0042572 1 5

C0013604 GO:0050769 1 5

C0013604 GO:0051457 1 5

C0013604 GO:0032376 1 5

C0013604 GO:0032370 1 5

C0013604 GO:0032373 1 5

C0013604 GO:0007259 1 5

C0013604 GO:0015377 1 5

C0013604 GO:0005506 1 5

C0013604 GO:0042169 1 5

C0013604 GO:0046165 1 5

C0013604 GO:0005217 1 5

C0013604 GO:0015807 1 5

C0013604 GO:0010677 1 5

C0013604 GO:0006081 1 5

C0013604 GO:0005024 1 5

C0013604 GO:0005518 1 5

C0013604 GO:0018107 1 5

C0013604 GO:0009187 1 5

C0013604 GO:0045927 1 5

C0013604 GO:0042119 1 5

C0013604 GO:0046326 1 5

C0013604 GO:0043506 1 5

C0013604 GO:0051147 1 5

C0013604 GO:0007270 1 5

C0013604 GO:0043507 1 5

C0013604 GO:0006090 1 5

C0013604 GO:0010828 1 5

C0013604 GO:0030641 1 5

C0013604 GO:0006094 1 5

C0013604 GO:0043467 1 5

C0013604 GO:0006693 1 5

C0013604 GO:0015758 1 5

C0013604 GO:0015459 1 5

C0013604 GO:0070120 1 5

C0013604 GO:0006895 1 5

C0013604 GO:0009110 1 5

C0013604 GO:0043627 1 5

C0013604 GO:0002443 1 5

C0013604 GO:0045123 1 5

C0013604 GO:0005041 1 5

C0013604 GO:0003705 1 5

C0013604 GO:0015749 1 5

C0013604 GO:0006885 1 5

C0013604 GO:0007094 1 5

C0012833 GO:0051043 2 12

C0012833 GO:0051044 1 12

C0012833 GO:0030228 1 12

C0012833 GO:0043954 1 12

C0012833 GO:0046887 1 12

C0012833 GO:0009164 1 12

C0012833 GO:0002703 1 12

C0012833 GO:0034199 1 12

C0012833 GO:0016863 2 12

C0012833 GO:0016860 1 12

C0012833 GO:0006306 1 12

C0012833 GO:0006305 1 12

C0012833 GO:0043205 1 12

C0012833 GO:0004716 1 12

C0012833 GO:0033238 1 12

C0012833 GO:0004712 1 12

C0012833 GO:0046504 1 12

C0012833 GO:0030858 1 12

C0012833 GO:0006284 1 12

C0012833 GO:0006688 1 12

C0012833 GO:0002822 2 12

C0012833 GO:0007281 2 12

C0012833 GO:0006477 1 12

C0012833 GO:0007286 1 12

C0012833 GO:0030291 1 12

C0012833 GO:0030295 1 12

C0012833 GO:0030296 1 12

C0012833 GO:0042326 1 12

C0012833 GO:0016812 1 12

C0012833 GO:0070633 1 12

C0012833 GO:0045821 1 12

C0012833 GO:0004622 1 12

C0012833 GO:0021537 1 12

C0012833 GO:0019320 1 12

C0012833 GO:0009895 2 12

C0012833 GO:0032410 1 12

C0012833 GO:0032412 1 12

C0012833 GO:0001953 2 12

C0012833 GO:0006458 1 12

C0012833 GO:0001952 1 12

C0012833 GO:0005337 1 12

C0012833 GO:0051224 1 12

C0012833 GO:0030178 1 12

C0012833 GO:0042552 1 12

C0012833 GO:0006942 1 12

C0012833 GO:0008645 1 12

C0012833 GO:0008643 1 12

C0012833 GO:0046850 1 12

C0012833 GO:0006110 1 12

C0012833 GO:0045912 2 12

C0012833 GO:0043025 1 12

C0012833 GO:0002688 1 12

C0012833 GO:0019882 1 12

C0012833 GO:0019883 1 12

C0012833 GO:0070167 1 12

C0012833 GO:0050796 1 12

C0012833 GO:0060606 1 12

C0012833 GO:0004437 1 12

C0012833 GO:0050716 1 12

C0012833 GO:0050715 2 12

C0012833 GO:0050714 1 12

C0012833 GO:0050718 1 12

C0012833 GO:0070567 1 12

C0012833 GO:0015179 1 12

C0012833 GO:0006752 1 12

C0012833 GO:0048638 1 12

C0012833 GO:0048145 1 12

C0012833 GO:0048634 1 12

C0012833 GO:0016620 1 12

C0012833 GO:0031228 1 12

C0012833 GO:0051453 1 12

C0012833 GO:0004691 1 12

C0012833 GO:0004690 1 12

C0012833 GO:0051457 1 12

C0012833 GO:0045078 1 12

C0012833 GO:0043489 1 12

C0012833 GO:0045072 1 12

C0012833 GO:0030705 1 12

C0012833 GO:0016459 1 12

C0012833 GO:0033273 1 12

C0012833 GO:0005506 1 12

C0012833 GO:0045622 1 12

C0012833 GO:0005501 1 12

C0012833 GO:0008286 1 12

C0012833 GO:0006879 1 12

C0012833 GO:0008329 1 12

C0012833 GO:0030317 1 12

C0012833 GO:0045211 1 12

C0012833 GO:0046883 1 12

C0012833 GO:0001654 1 12

C0012833 GO:0045216 1 12

C0012833 GO:0010677 2 12

C0012833 GO:0033293 1 12

C0012833 GO:0004190 2 12

C0012833 GO:0019047 1 12

C0012833 GO:0045927 1 12

C0012833 GO:0030035 1 12

C0012833 GO:0017048 2 12

C0012833 GO:0060021 1 12

C0012833 GO:0007270 1 12

C0012833 GO:0007272 1 12

C0012833 GO:0006090 1 12

C0012833 GO:0055092 2 12

C0012833 GO:0032350 1 12

C0012833 GO:0042354 1 12

C0012833 GO:0010828 2 12

C0012833 GO:0070668 1 12

C0012833 GO:0046823 1 12

C0012833 GO:0032526 1 12

C0012833 GO:0019212 1 12

C0012833 GO:0021915 1 12

C0012833 GO:0019218 1 12

C0012833 GO:0048593 1 12

C0012833 GO:0032856 1 12

C0012833 GO:0015662 1 12

C0012833 GO:0050750 1 12

C0012833 GO:0001838 1 12

C0012833 GO:0010595 1 12

C0012833 GO:0010594 2 12

C0012833 GO:0005044 1 12

C0012833 GO:0050995 1 12

C0012833 GO:0050994 1 12

C0012833 GO:0032321 1 12

C0012833 GO:0043112 1 12

C0012833 GO:0006413 1 12

C0012833 GO:0008366 1 12

C0012833 GO:0009593 2 12

C0012833 GO:0005149 1 12

C0012833 GO:0007612 1 12

C0012833 GO:0007162 2 12

C0012833 GO:0043524 1 12

C0012833 GO:0019915 1 12

C0012833 GO:0031343 2 12

C0012833 GO:0045767 1 12

C0012833 GO:0016597 2 12

C0012833 GO:0031346 1 12

C0012833 GO:0042446 1 12

C0012833 GO:0005540 1 12

C0012833 GO:0002263 1 12

C0012833 GO:0045661 1 12

C0012833 GO:0006096 1 12

C0012833 GO:0033032 1 12

C0012833 GO:0051057 1 12

C0012833 GO:0016202 1 12

C0012833 GO:0051055 1 12

C0012833 GO:0051119 1 12

C0012833 GO:0030218 1 12

C0012833 GO:0051059 1 12

C0012833 GO:0051058 1 12

C0012833 GO:0008170 1 12

C0012833 GO:0016894 1 12

C0012833 GO:0051702 2 12

C0012833 GO:0042994 1 12

C0012833 GO:0042992 1 12

C0012833 GO:0014020 1 12

C0012833 GO:0016566 1 12

C0012833 GO:0016565 1 12

C0012833 GO:0016291 1 12

C0012833 GO:0004708 1 12

C0012833 GO:0046716 1 12

C0012833 GO:0015858 1 12

C0012833 GO:0050820 1 12

C0012833 GO:0001763 1 12

C0012833 GO:0046496 1 12

C0012833 GO:0005929 1 12

C0012833 GO:0048002 1 12

C0012833 GO:0030286 1 12

C0012833 GO:0007530 1 12

C0012833 GO:0051181 1 12

C0012833 GO:0046915 1 12

C0012833 GO:0042098 1 12

C0012833 GO:0008088 1 12

C0012833 GO:0030149 1 12

C0012833 GO:0020037 1 12

C0012833 GO:0032768 1 12

C0012833 GO:0032769 1 12

C0012833 GO:0006584 1 12

C0012833 GO:0030069 1 12

C0012833 GO:0046660 1 12

C0012833 GO:0009953 1 12

C0012833 GO:0042269 1 12

C0012833 GO:0034384 1 12

C0012833 GO:0017080 1 12

C0012833 GO:0002690 1 12

C0012833 GO:0043010 1 12

C0012833 GO:0042834 1 12

C0012833 GO:0003785 1 12

C0012833 GO:0006633 1 12

C0012833 GO:0060260 1 12

C0012833 GO:0006637 1 12

C0012833 GO:0019956 1 12

C0012833 GO:0009311 1 12

C0012833 GO:0060263 1 12

C0012833 GO:0007043 1 12

C0012833 GO:0005583 2 12

C0012833 GO:0015166 1 12

C0012833 GO:0031397 1 12

C0012833 GO:0005355 2 12

C0012833 GO:0042169 1 12

C0012833 GO:0009062 1 12

C0012833 GO:0030804 1 12

C0012833 GO:0030801 1 12

C0012833 GO:0002237 2 12

C0012833 GO:0045619 1 12

C0012833 GO:0034405 1 12

C0012833 GO:0045616 1 12

C0012833 GO:0016331 1 12

C0012833 GO:0030323 1 12

C0012833 GO:0050868 1 12

C0012833 GO:0046365 1 12

C0012833 GO:0050866 1 12

C0012833 GO:0034623 1 12

C0012833 GO:0046890 1 12

C0012833 GO:0004675 1 12

C0012833 GO:0016538 1 12

C0012833 GO:0042787 1 12

C0012833 GO:0033189 1 12

C0012833 GO:0010810 1 12

C0012833 GO:0010812 2 12

C0012833 GO:0030021 1 12

C0012833 GO:0016903 1 12

C0012833 GO:0055067 1 12

C0012833 GO:0043627 1 12

C0012833 GO:0007266 1 12

C0012833 GO:0035295 1 12

C0012833 GO:0005977 1 12

C0012833 GO:0051923 1 12

C0012833 GO:0003705 1 12

C0012833 GO:0006885 2 12

C0012833 GO:0060538 2 12

C0012833 GO:0046697 1 12

C0012833 GO:0019362 1 12

C0012833 GO:0031970 1 12

C0012833 GO:0032387 1 12

C0012833 GO:0019228 1 12

C0012833 GO:0021510 1 12

C0012833 GO:0005788 1 12

C0012833 GO:0031058 1 12

C0012833 GO:0045981 1 12

C0012833 GO:0048864 1 12

C0012833 GO:0016338 2 12

C0012833 GO:0007217 1 12

C0012833 GO:0070001 2 12

C0012833 GO:0006073 1 12

C0012833 GO:0046870 1 12

C0012833 GO:0043120 2 12

C0012833 GO:0070228 1 12

C0012833 GO:0005159 1 12

C0012833 GO:0005158 1 12

C0012833 GO:0042129 1 12

C0012833 GO:0043536 1 12

C0012833 GO:0043535 2 12

C0012833 GO:0046631 1 12

C0012833 GO:0034235 1 12

C0012833 GO:0051879 1 12

C0012833 GO:0002377 1 12

C0012833 GO:0001910 1 12

C0012833 GO:0004551 1 12

C0012833 GO:0045494 1 12

C0012833 GO:0022600 1 12

C0012833 GO:0046006 1 12

C0012833 GO:0042398 1 12

C0012833 GO:0006929 1 12

C0012833 GO:0051893 1 12

C0012833 GO:0034765 2 12

C0012833 GO:0021700 1 12

C0012833 GO:0042625 1 12

C0012833 GO:0043548 1 12

C0012833 GO:0005858 1 12

C0012833 GO:0005522 1 12

C0012833 GO:0048641 1 12

C0012833 GO:0005520 1 12

C0012833 GO:0030879 2 12

C0012833 GO:0005138 1 12

C0012833 GO:0048730 1 12

C0012833 GO:0050810 3 12

C0012833 GO:0042308 1 12

C0012833 GO:0030278 1 12

C0012833 GO:0050819 1 12

C0012833 GO:0042301 1 12

C0012833 GO:0044275 2 12

C0012833 GO:0008091 1 12

C0012833 GO:0008093 1 12

C0012833 GO:0008206 1 12

C0012833 GO:0044447 1 12

C0012833 GO:0032770 1 12

C0012833 GO:0010921 1 12

C0012833 GO:0031016 1 12

C0012833 GO:0000185 1 12

C0012833 GO:0045580 2 12

C0012833 GO:0045429 1 12

C0012833 GO:0045428 1 12

C0012833 GO:0046677 1 12

C0012833 GO:0008543 1 12

C0012833 GO:0007250 1 12

C0012833 GO:0032374 1 12

C0012833 GO:0009116 1 12

C0012833 GO:0032376 2 12

C0012833 GO:0032371 1 12

C0012833 GO:0032370 2 12

C0012833 GO:0032373 2 12

C0012833 GO:0005930 1 12

C0012833 GO:0060562 1 12

C0012833 GO:0044042 1 12

C0012833 GO:0019209 1 12

C0012833 GO:0031330 1 12

C0012833 GO:0000217 1 12

C0012833 GO:0004861 1 12

C0012833 GO:0004864 1 12

C0012833 GO:0005593 1 12

C0012833 GO:0050770 1 12

C0012833 GO:0050670 1 12

C0012833 GO:0050671 1 12

C0012833 GO:0005024 1 12

C0012833 GO:0006007 1 12

C0012833 GO:0009187 1 12

C0012833 GO:0042119 1 12

C0012833 GO:0042116 1 12

C0012833 GO:0005160 1 12

C0012833 GO:0005161 1 12

C0012833 GO:0043506 1 12

C0012833 GO:0043507 1 12

C0012833 GO:0045768 1 12

C0012833 GO:0015074 1 12

C0012833 GO:0006937 1 12

C0012833 GO:0031638 1 12

C0012833 GO:0045766 1 12

C0012833 GO:0002573 1 12

C0012833 GO:0015758 1 12

C0012833 GO:0015695 2 12

C0012833 GO:0045604 1 12

C0012833 GO:0007584 1 12

C0012833 GO:0007585 1 12

C0012833 GO:0009110 1 12

C0012833 GO:0046580 1 12

C0012833 GO:0051646 1 12

C0012833 GO:0034599 1 12

C0012833 GO:0001530 2 12

C0012833 GO:0006941 1 12

C0012833 GO:0016877 1 12

C0012833 GO:0019840 1 12

C0012833 GO:0045124 1 12

C0012833 GO:0030902 1 12

C0012833 GO:0008021 1 12

C0012833 GO:0046637 1 12

C0012833 GO:0046634 1 12

C0012833 GO:0008585 1 12

C0012833 GO:0050926 1 12

C0012833 GO:0050927 1 12

C0012833 GO:0030345 1 12

C0012833 GO:0008374 2 12

C0012833 GO:0008375 1 12

C0012833 GO:0051897 1 12

C0012833 GO:0031985 1 12

C0012833 GO:0007519 2 12

C0012833 GO:0010896 1 12

C0012833 GO:0010898 1 12

C0012833 GO:0030510 1 12

C0012833 GO:0032393 1 12

C0012833 GO:0005796 1 12

C0012833 GO:0005790 1 12

C0012833 GO:0019827 1 12

C0012833 GO:0006769 1 12

C0012833 GO:0046579 1 12

C0012833 GO:0030166 1 12

C0012833 GO:0030048 1 12

C0012833 GO:0010975 1 12

C0012833 GO:0030512 1 12

C0012833 GO:0010874 2 12

C0012833 GO:0010875 2 12

C0012833 GO:0048872 1 12

C0012833 GO:0015833 1 12

C0012833 GO:0051353 2 12

C0012833 GO:0032731 1 12

C0012833 GO:0060416 1 12

C0012833 GO:0030195 1 12

C0012833 GO:0030194 1 12

C0012833 GO:0007632 1 12

C0012833 GO:0019934 1 12

C0012833 GO:0004693 1 12

C0012833 GO:0032496 1 12

C0012833 GO:0002286 1 12

C0012833 GO:0050709 1 12

C0012833 GO:0002285 1 12

C0012833 GO:0016045 1 12

C0012833 GO:0048385 1 12

C0012833 GO:0050700 1 12

C0012833 GO:0015149 1 12

C0012833 GO:0002366 1 12

C0012833 GO:0042104 1 12

C0012833 GO:0001841 1 12

C0012833 GO:0001843 1 12

C0012833 GO:0015145 1 12

C0012833 GO:0046545 1 12

C0012833 GO:0030890 1 12

C0012833 GO:0048255 1 12

C0012833 GO:0042632 2 12

C0012833 GO:0045165 1 12

C0012833 GO:0042734 1 12

C0012833 GO:0031645 1 12

C0012833 GO:0002715 1 12

C0012833 GO:0030864 1 12

C0012833 GO:0045730 2 12

C0012833 GO:0043034 2 12

C0012833 GO:0045739 1 12

C0012833 GO:0015665 1 12

C0012833 GO:0009712 1 12

C0012833 GO:0004115 1 12

C0012833 GO:0035148 1 12

C0012833 GO:0034311 1 12

C0012833 GO:0005903 1 12

C0012833 GO:0030810 1 12

C0012833 GO:0009124 1 12

C0012833 GO:0000041 1 12

C0012833 GO:0046782 1 12

C0012833 GO:0046466 1 12

C0012833 GO:0022614 1 12

C0012833 GO:0015807 2 12

C0012833 GO:0001894 1 12

C0012833 GO:0001893 1 12

C0012833 GO:0005507 1 12

C0012833 GO:0045940 1 12

C0012833 GO:0043370 1 12

C0012833 GO:0045861 1 12

C0012833 GO:0019200 1 12

C0012833 GO:0046326 2 12

C0012833 GO:0032862 1 12

C0012833 GO:0045682 1 12

C0012833 GO:0030169 1 12

C0012833 GO:0030641 1 12

C0012833 GO:0045684 2 12

C0012833 GO:0010741 1 12

C0012833 GO:0034101 1 12

C0012833 GO:0034103 1 12

C0012833 GO:0048662 1 12

C0012833 GO:0048660 1 12

C0012833 GO:0009247 1 12

C0012833 GO:0048742 1 12

C0012833 GO:0042102 1 12

C0012833 GO:0002449 1 12

C0012833 GO:0031214 2 12

C0012833 GO:0002440 1 12

C0012833 GO:0016049 1 12

C0012833 GO:0051004 1 12

C0012833 GO:0015749 1 12

C0012833 GO:0001937 1 12

C0002170 GO:0070665 1 8

C0002170 GO:0005786 1 8

C0002170 GO:0030228 1 8

C0002170 GO:0032770 1 8

C0002170 GO:0050681 1 8

C0002170 GO:0021510 1 8

C0002170 GO:0046631 1 8

C0002170 GO:0016331 1 8

C0002170 GO:0030509 1 8

C0002170 GO:0007162 1 8

C0002170 GO:0007163 1 8

C0002170 GO:0002702 2 8

C0002170 GO:0031057 1 8

C0002170 GO:0048260 1 8

C0002170 GO:0045767 1 8

C0002170 GO:0016597 1 8

C0002170 GO:0001894 2 8

C0002170 GO:0051453 1 8

C0002170 GO:0005149 1 8

C0002170 GO:0015085 1 8

C0002170 GO:0005072 1 8

C0002170 GO:0006304 2 8

C0002170 GO:0048864 1 8

C0002170 GO:0005770 1 8

C0002170 GO:0018212 1 8

C0002170 GO:0018210 1 8

C0002170 GO:0055117 1 8

C0002170 GO:0044419 1 8

C0002170 GO:0045661 1 8

C0002170 GO:0045839 3 8

C0002170 GO:0010875 1 8

C0002170 GO:0051452 1 8

C0002170 GO:0010522 1 8

C0002170 GO:0033032 1 8

C0002170 GO:0051057 1 8

C0002170 GO:0032934 1 8

C0002170 GO:0043966 1 8

C0002170 GO:0002720 2 8

C0002170 GO:0007612 1 8

C0002170 GO:0005159 1 8

C0002170 GO:0014003 1 8

C0002170 GO:0051059 1 8

C0002170 GO:0030048 1 8

C0002170 GO:0043535 1 8

C0002170 GO:0043534 1 8

C0002170 GO:0007173 2 8

C0002170 GO:0010975 1 8

C0002170 GO:0070668 1 8

C0002170 GO:0051701 1 8

C0002170 GO:0030514 1 8

C0002170 GO:0070875 2 8

C0002170 GO:0010874 1 8

C0002170 GO:0030510 1 8

C0002170 GO:0019827 1 8

C0002170 GO:0002429 1 8

C0002170 GO:0006306 1 8

C0002170 GO:0030260 1 8

C0002170 GO:0060395 1 8

C0002170 GO:0016055 1 8

C0002170 GO:0050926 1 8

C0002170 GO:0051353 1 8

C0002170 GO:0002377 1 8

C0002170 GO:0042177 1 8

C0002170 GO:0046849 1 8

C0002170 GO:0051784 3 8

C0002170 GO:0004709 2 8

C0002170 GO:0043034 1 8

C0002170 GO:0021537 1 8

C0002170 GO:0007632 1 8

C0002170 GO:0060263 1 8

C0002170 GO:0008252 1 8

C0002170 GO:0006929 1 8

C0002170 GO:0010906 1 8

C0002170 GO:0046658 1 8

C0002170 GO:0043489 1 8

C0002170 GO:0034103 1 8

C0002170 GO:0032946 1 8

C0002170 GO:0000786 2 8

C0002170 GO:0045089 1 8

C0002170 GO:0009068 1 8

C0002170 GO:0051224 1 8

C0002170 GO:0030071 1 8

C0002170 GO:0048488 2 8

C0002170 GO:0007062 3 8

C0002170 GO:0003205 1 8

C0002170 GO:0031577 3 8

C0002170 GO:0003206 1 8

C0002170 GO:0030890 1 8

C0002170 GO:0004428 2 8

C0002170 GO:0045646 1 8

C0002170 GO:0043548 2 8

C0002170 GO:0008088 1 8

C0002170 GO:0005391 1 8

C0002170 GO:0046850 1 8

C0002170 GO:0006687 1 8

C0002170 GO:0006110 2 8

C0002170 GO:0045725 2 8

C0002170 GO:0043025 1 8

C0002170 GO:0032768 1 8

C0002170 GO:0004551 1 8

C0002170 GO:0071174 3 8

C0002170 GO:0071173 3 8

C0002170 GO:0020037 1 8

C0002170 GO:0005071 2 8

C0002170 GO:0030371 1 8

C0002170 GO:0048500 1 8

C0002170 GO:0048709 1 8

C0002170 GO:0006305 1 8

C0002170 GO:0008013 2 8

C0002170 GO:0050796 1 8

C0002170 GO:0060070 3 8

C0002170 GO:0004364 1 8

C0002170 GO:0030276 1 8

C0002170 GO:0048255 1 8

C0002170 GO:0043256 1 8

C0002170 GO:0000002 1 8

C0002170 GO:0050819 1 8

C0002170 GO:0044275 1 8

C0002170 GO:0045851 1 8

C0002170 GO:0030675 2 8

C0002170 GO:0042439 1 8

C0002170 GO:0009952 1 8

C0002170 GO:0006895 1 8

C0002170 GO:0030863 1 8

C0002170 GO:0050650 1 8

C0002170 GO:0050654 1 8

C0002170 GO:0030864 1 8

C0002170 GO:0033261 2 8

C0002170 GO:0017080 1 8

C0002170 GO:0045730 1 8

C0002170 GO:0045737 1 8

C0002170 GO:0015179 1 8

C0002170 GO:0008301 1 8

C0002170 GO:0043010 1 8

C0002170 GO:0045841 3 8

C0002170 GO:0032376 1 8

C0002170 GO:0033613 1 8

C0002170 GO:0006692 1 8

C0002170 GO:0019956 1 8

C0002170 GO:0010921 1 8

C0002170 GO:0035113 1 8

C0002170 GO:0046579 1 8

C0002170 GO:0009312 1 8

C0002170 GO:0005901 1 8

C0002170 GO:0031228 1 8

C0002170 GO:0006942 1 8

C0002170 GO:0043393 1 8

C0002170 GO:0052126 1 8

C0002170 GO:0005871 1 8

C0002170 GO:0033280 1 8

C0002170 GO:0031513 1 8

C0002170 GO:0043087 1 8

C0002170 GO:0009124 1 8

C0002170 GO:0031225 1 8

C0002170 GO:0070001 1 8

C0002170 GO:0007127 1 8

C0002170 GO:0043560 1 8

C0002170 GO:0008091 1 8

C0002170 GO:0050885 1 8

C0002170 GO:0032374 1 8

C0002170 GO:0001709 3 8

C0002170 GO:0032371 1 8

C0002170 GO:0032370 1 8

C0002170 GO:0032373 1 8

C0002170 GO:0030705 1 8

C0002170 GO:0032273 1 8

C0002170 GO:0002237 1 8

C0002170 GO:0042572 1 8

C0002170 GO:0019200 1 8

C0002170 GO:0042169 1 8

C0002170 GO:0008034 1 8

C0002170 GO:0006879 1 8

C0002170 GO:0031330 1 8

C0002170 GO:0034637 1 8

C0002170 GO:0008308 1 8

C0002170 GO:0002757 1 8

C0002170 GO:0048385 1 8

C0002170 GO:0046870 1 8

C0002170 GO:0009953 1 8

C0002170 GO:0007131 2 8

C0002170 GO:0042562 1 8

C0002170 GO:0051983 2 8

C0002170 GO:0051881 1 8

C0002170 GO:0043470 1 8

C0002170 GO:0043471 1 8

C0002170 GO:0050679 1 8

C0002170 GO:0043467 1 8

C0002170 GO:0050671 1 8

C0002170 GO:0051828 1 8

C0002170 GO:0004190 1 8

C0002170 GO:0046718 1 8

C0002170 GO:0034405 1 8

C0002170 GO:0001764 2 8

C0002170 GO:0017046 1 8

C0002170 GO:0009895 2 8

C0002170 GO:0030323 1 8

C0002170 GO:0007250 1 8

C0002170 GO:0030326 1 8

C0002170 GO:0050770 1 8

C0002170 GO:0005160 1 8

C0002170 GO:0018108 1 8

C0002170 GO:0052192 1 8

C0002170 GO:0010948 1 8

C0002170 GO:0008210 1 8

C0002170 GO:0009108 1 8

C0002170 GO:0034623 1 8

C0002170 GO:0007270 1 8

C0002170 GO:0002724 3 8

C0002170 GO:0006096 1 8

C0002170 GO:0030641 1 8

C0002170 GO:0045684 1 8

C0002170 GO:0045768 1 8

C0002170 GO:0032355 2 8

C0002170 GO:0001727 1 8

C0002170 GO:0030520 1 8

C0002170 GO:0032350 1 8

C0002170 GO:0042354 1 8

C0002170 GO:0006693 1 8

C0002170 GO:0032210 1 8

C0002170 GO:0002573 1 8

C0002170 GO:0050927 1 8

C0002170 GO:0044409 1 8

C0002170 GO:0070412 2 8

C0002170 GO:0070410 4 8

C0002170 GO:0007098 1 8

C0002170 GO:0070491 1 8

C0002170 GO:0030195 1 8

C0002170 GO:0005697 1 8

C0002170 GO:0050851 1 8

C0002170 GO:0050852 1 8

C0002170 GO:0034451 1 8

C0002170 GO:0021915 2 8

C0002170 GO:0007584 1 8

C0002170 GO:0048593 1 8

C0002170 GO:0032984 1 8

C0002170 GO:0050690 1 8

C0002170 GO:0009112 1 8

C0002170 GO:0050660 1 8

C0002170 GO:0003785 1 8

C0002170 GO:0015175 1 8

C0002170 GO:0002718 3 8

C0002170 GO:0034185 1 8

C0002170 GO:0005605 2 8

C0002170 GO:0006493 1 8

C0002170 GO:0010594 1 8

C0002170 GO:0005044 1 8

C0002170 GO:0008517 1 8

C0002170 GO:0005041 1 8

C0002170 GO:0003705 2 8

C0002170 GO:0045124 1 8

C0002170 GO:0003707 1 8

C0002170 GO:0015807 1 8

C0002170 GO:0030900 1 8

C0002170 GO:0007094 3 8

C0002170 GO:0044403 1 8

C0002170 GO:0051806 1 8

C0003123 GO:0033613 1 10

C0003123 GO:0042133 1 10

C0003123 GO:0005782 1 10

C0003123 GO:0005149 1 10

C0003123 GO:0008021 1 10

C0003123 GO:0043954 1 10

C0003123 GO:0032387 1 10

C0003123 GO:0030427 1 10

C0003123 GO:0030426 1 10

C0003123 GO:0046631 1 10

C0003123 GO:0030509 1 10

C0003123 GO:0009164 1 10

C0003123 GO:0007163 1 10

C0003123 GO:0032210 1 10

C0003123 GO:0008375 1 10

C0003123 GO:0046718 1 10

C0003123 GO:0016863 2 10

C0003123 GO:0001894 1 10

C0003123 GO:0015085 1 10

C0003123 GO:0006305 1 10

C0003123 GO:0006304 2 10

C0003123 GO:0048864 1 10

C0003123 GO:0043010 2 10

C0003123 GO:0043206 1 10

C0003123 GO:0005770 1 10

C0003123 GO:0018212 1 10

C0003123 GO:0018210 1 10

C0003123 GO:0031513 1 10

C0003123 GO:0044419 1 10

C0003123 GO:0004712 1 10

C0003123 GO:0010721 1 10

C0003123 GO:0031907 1 10

C0003123 GO:0045839 2 10

C0003123 GO:0010875 1 10

C0003123 GO:0030323 1 10

C0003123 GO:0050926 1 10

C0003123 GO:0019200 1 10

C0003123 GO:0033032 1 10

C0003123 GO:0032934 1 10

C0003123 GO:0002720 1 10

C0003123 GO:0031960 1 10

C0003123 GO:0032153 1 10

C0003123 GO:0032155 1 10

C0003123 GO:0043470 1 10

C0003123 GO:0051059 2 10

C0003123 GO:0021510 2 10

C0003123 GO:0030048 1 10

C0003123 GO:0043535 1 10

C0003123 GO:0007173 1 10

C0003123 GO:0030260 1 10

C0003123 GO:0070668 1 10

C0003123 GO:0009074 1 10

C0003123 GO:0051701 1 10

C0003123 GO:0042992 1 10

C0003123 GO:0070875 1 10

C0003123 GO:0010874 1 10

C0003123 GO:0030169 1 10

C0003123 GO:0019827 1 10

C0003123 GO:0007270 1 10

C0003123 GO:0018209 1 10

C0003123 GO:0048871 1 10

C0003123 GO:0034284 1 10

C0003123 GO:0006041 1 10

C0003123 GO:0016055 2 10

C0003123 GO:0006044 1 10

C0003123 GO:0007162 1 10

C0003123 GO:0051353 1 10

C0003123 GO:0001654 1 10

C0003123 GO:0032350 1 10

C0003123 GO:0046887 1 10

C0003123 GO:0043627 1 10

C0003123 GO:0045821 1 10

C0003123 GO:0051784 2 10

C0003123 GO:0019439 1 10

C0003123 GO:0033261 1 10

C0003123 GO:0004709 2 10

C0003123 GO:0004708 1 10

C0003123 GO:0045494 1 10

C0003123 GO:0001505 1 10

C0003123 GO:0021537 2 10

C0003123 GO:0007632 1 10

C0003123 GO:0009895 1 10

C0003123 GO:0043087 2 10

C0003123 GO:0042398 1 10

C0003123 GO:0010906 1 10

C0003123 GO:0052126 1 10

C0003123 GO:0034103 1 10

C0003123 GO:0001764 2 10

C0003123 GO:0051457 1 10

C0003123 GO:0005929 1 10

C0003123 GO:0045089 1 10

C0003123 GO:0051224 1 10

C0003123 GO:0048488 2 10

C0003123 GO:0007062 2 10

C0003123 GO:0031577 2 10

C0003123 GO:0031575 1 10

C0003123 GO:0016049 1 10

C0003123 GO:0043547 1 10

C0003123 GO:0002377 1 10

C0003123 GO:0004428 1 10

C0003123 GO:0008645 1 10

C0003123 GO:0043548 1 10

C0003123 GO:0050700 1 10

C0003123 GO:0008088 1 10

C0003123 GO:0008643 1 10

C0003123 GO:0009880 1 10

C0003123 GO:0046850 1 10

C0003123 GO:0006110 2 10

C0003123 GO:0045912 1 10

C0003123 GO:0045727 1 10

C0003123 GO:0043025 1 10

C0003123 GO:0032768 1 10

C0003123 GO:0071174 2 10

C0003123 GO:0071173 2 10

C0003123 GO:0020037 1 10

C0003123 GO:0006306 1 10

C0003123 GO:0030371 1 10

C0003123 GO:0033764 1 10

C0003123 GO:0008013 2 10

C0003123 GO:0005138 1 10

C0003123 GO:0060070 2 10

C0003123 GO:0006895 1 10

C0003123 GO:0042308 1 10

C0003123 GO:0045725 1 10

C0003123 GO:0030069 1 10

C0003123 GO:0050796 1 10

C0003123 GO:0050819 1 10

C0003123 GO:0016597 1 10

C0003123 GO:0044275 1 10

C0003123 GO:0030675 2 10

C0003123 GO:0009953 1 10

C0003123 GO:0042734 1 10

C0003123 GO:0046906 1 10

C0003123 GO:0030863 1 10

C0003123 GO:0032364 1 10

C0003123 GO:0016830 1 10

C0003123 GO:0030864 1 10

C0003123 GO:0030890 1 10

C0003123 GO:0015074 1 10

C0003123 GO:0017080 2 10

C0003123 GO:0045730 1 10

C0003123 GO:0046823 1 10

C0003123 GO:0001709 2 10

C0003123 GO:0032107 1 10

C0003123 GO:0042834 1 10

C0003123 GO:0006633 1 10

C0003123 GO:0045841 2 10

C0003123 GO:0032104 1 10

C0003123 GO:0050808 1 10

C0003123 GO:0002702 1 10

C0003123 GO:0015280 1 10

C0003123 GO:0019956 1 10

C0003123 GO:0043525 1 10

C0003123 GO:0048145 1 10

C0003123 GO:0032770 1 10

C0003123 GO:0016331 1 10

C0003123 GO:0005901 1 10

C0003123 GO:0009112 1 10

C0003123 GO:0042177 1 10

C0003123 GO:0032373 1 10

C0003123 GO:0051453 1 10

C0003123 GO:0005871 1 10

C0003123 GO:0042572 1 10

C0003123 GO:0043241 1 10

C0003123 GO:0000786 1 10

C0003123 GO:0070001 1 10

C0003123 GO:0007127 1 10

C0003123 GO:0043560 1 10

C0003123 GO:0007250 1 10

C0003123 GO:0009749 1 10

C0003123 GO:0032374 1 10

C0003123 GO:0032376 1 10

C0003123 GO:0032371 1 10

C0003123 GO:0032370 1 10

C0003123 GO:0050768 1 10

C0003123 GO:0030705 1 10

C0003123 GO:0050927 1 10

C0003123 GO:0050660 1 10

C0003123 GO:0045740 1 10

C0003123 GO:0032273 1 10

C0003123 GO:0045661 1 10

C0003123 GO:0005506 1 10

C0003123 GO:0010921 1 10

C0003123 GO:0043034 1 10

C0003123 GO:0007612 1 10

C0003123 GO:0017157 1 10

C0003123 GO:0042169 3 10

C0003123 GO:0015296 1 10

C0003123 GO:0006879 1 10

C0003123 GO:0015459 1 10

C0003123 GO:0008517 1 10

C0003123 GO:0015929 1 10

C0003123 GO:0042113 1 10

C0003123 GO:0015807 1 10

C0003123 GO:0002440 1 10

C0003123 GO:0001656 1 10

C0003123 GO:0010677 1 10

C0003123 GO:0008091 1 10

C0003123 GO:0007131 2 10

C0003123 GO:0042562 1 10

C0003123 GO:0051983 1 10

C0003123 GO:0010594 1 10

C0003123 GO:0006096 1 10

C0003123 GO:0009187 1 10

C0003123 GO:0043471 1 10

C0003123 GO:0007423 1 10

C0003123 GO:0002460 2 10

C0003123 GO:0043467 1 10

C0003123 GO:0005024 1 10

C0003123 GO:0051828 1 10

C0003123 GO:0004190 1 10

C0003123 GO:0005044 1 10

C0003123 GO:0034405 1 10

C0003123 GO:0045861 1 10

C0003123 GO:0005518 1 10

C0003123 GO:0017046 1 10

C0003123 GO:0000185 1 10

C0003123 GO:0018105 2 10

C0003123 GO:0045927 1 10

C0003123 GO:0042119 1 10

C0003123 GO:0005041 1 10

C0003123 GO:0005160 1 10

C0003123 GO:0046326 1 10

C0003123 GO:0008286 2 10

C0003123 GO:0018108 1 10

C0003123 GO:0052192 1 10

C0003123 GO:0046112 1 10

C0003123 GO:0010948 1 10

C0003123 GO:0008034 1 10

C0003123 GO:0045124 1 10

C0003123 GO:0043507 1 10

C0003123 GO:0043506 1 10

C0003123 GO:0002724 2 10

C0003123 GO:0006090 1 10

C0003123 GO:0010828 1 10

C0003123 GO:0030641 1 10

C0003123 GO:0045684 1 10

C0003123 GO:0032355 2 10

C0003123 GO:0001727 1 10

C0003123 GO:0030520 1 10

C0003123 GO:0070567 1 10

C0003123 GO:0042354 1 10

C0003123 GO:0006527 1 10

C0003123 GO:0006693 1 10

C0003123 GO:0006525 1 10

C0003123 GO:0002573 2 10

C0003123 GO:0004675 1 10

C0003123 GO:0006692 1 10

C0003123 GO:0070412 1 10

C0003123 GO:0030934 1 10

C0003123 GO:0070410 2 10

C0003123 GO:0007098 1 10

C0003123 GO:0070491 1 10

C0003123 GO:0015026 1 10

C0003123 GO:0030195 1 10

C0003123 GO:0043588 1 10

C0003123 GO:0015179 1 10

C0003123 GO:0016229 1 10

C0003123 GO:0034451 1 10

C0003123 GO:0021915 1 10

C0003123 GO:0045088 1 10

C0003123 GO:0048592 1 10

C0003123 GO:0048593 2 10

C0003123 GO:0050661 1 10

C0003123 GO:0033483 1 10

C0003123 GO:0015758 1 10

C0003123 GO:0009110 1 10

C0003123 GO:0003785 1 10

C0003123 GO:0002718 2 10

C0003123 GO:0043624 1 10

C0003123 GO:0009746 1 10

C0003123 GO:0006493 1 10

C0003123 GO:0032318 1 10

C0003123 GO:0043197 1 10

C0003123 GO:0051384 1 10

C0003123 GO:0002793 1 10

C0003123 GO:0019047 1 10

C0003123 GO:0003705 2 10

C0003123 GO:0016986 1 10

C0003123 GO:0003707 1 10

C0003123 GO:0032320 1 10

C0003123 GO:0044409 1 10

C0003123 GO:0030900 2 10

C0003123 GO:0002250 2 10

C0003123 GO:0015749 1 10

C0003123 GO:0006885 1 10

C0003123 GO:0007094 2 10

C0003123 GO:0044403 1 10

C0003123 GO:0051806 1 10

C0003123 GO:0043112 1 10

C0004093 GO:0051043 1 16

C0004093 GO:0051044 1 16

C0004093 GO:0030228 2 16

C0004093 GO:0043954 2 16

C0004093 GO:0010677 2 16

C0004093 GO:0009164 1 16

C0004093 GO:0048260 1 16

C0004093 GO:0016863 2 16

C0004093 GO:0016860 1 16

C0004093 GO:0006306 1 16

C0004093 GO:0006305 1 16

C0004093 GO:0006304 1 16

C0004093 GO:0043205 1 16

C0004093 GO:0018212 1 16

C0004093 GO:0018210 2 16

C0004093 GO:0004715 1 16

C0004093 GO:0046504 1 16

C0004093 GO:0005788 1 16

C0004093 GO:0042326 1 16

C0004093 GO:0034284 1 16

C0004093 GO:0016812 1 16

C0004093 GO:0045821 1 16

C0004093 GO:0051784 3 16

C0004093 GO:0021537 3 16

C0004093 GO:0009895 2 16

C0004093 GO:0051224 1 16

C0004093 GO:0030071 1 16

C0004093 GO:0006885 1 16

C0004093 GO:0008643 1 16

C0004093 GO:0043028 1 16

C0004093 GO:0046850 1 16

C0004093 GO:0006110 1 16

C0004093 GO:0045912 2 16

C0004093 GO:0043025 1 16

C0004093 GO:0043027 1 16

C0004093 GO:0019882 1 16

C0004093 GO:0019883 1 16

C0004093 GO:0005071 2 16

C0004093 GO:0005138 1 16

C0004093 GO:0042439 1 16

C0004093 GO:0035303 2 16

C0004093 GO:0004437 1 16

C0004093 GO:0030675 2 16

C0004093 GO:0050714 1 16

C0004093 GO:0050650 1 16

C0004093 GO:0050654 1 16

C0004093 GO:0070567 1 16

C0004093 GO:0006693 1 16

C0004093 GO:0006692 1 16

C0004093 GO:0015179 1 16

C0004093 GO:0015175 1 16

C0004093 GO:0008021 1 16

C0004093 GO:0050810 1 16

C0004093 GO:0048145 1 16

C0004093 GO:0033619 1 16

C0004093 GO:0046579 1 16

C0004093 GO:0031228 2 16

C0004093 GO:0004693 1 16

C0004093 GO:0031498 1 16

C0004093 GO:0051457 1 16

C0004093 GO:0045078 1 16

C0004093 GO:0043489 1 16

C0004093 GO:0045072 1 16

C0004093 GO:0030705 1 16

C0004093 GO:0005506 1 16

C0004093 GO:0008286 2 16

C0004093 GO:0006879 1 16

C0004093 GO:0008329 1 16

C0004093 GO:0015929 1 16

C0004093 GO:0045211 1 16

C0004093 GO:0001654 1 16

C0004093 GO:0046887 1 16

C0004093 GO:0046888 1 16

C0004093 GO:0034329 1 16

C0004093 GO:0051983 2 16

C0004093 GO:0006337 1 16

C0004093 GO:0004190 2 16

C0004093 GO:0007229 1 16

C0004093 GO:0017046 1 16

C0004093 GO:0018107 1 16

C0004093 GO:0018105 1 16

C0004093 GO:0045927 1 16

C0004093 GO:0018108 1 16

C0004093 GO:0045884 1 16

C0004093 GO:0007270 1 16

C0004093 GO:0006090 1 16

C0004093 GO:0010828 1 16

C0004093 GO:0032355 1 16

C0004093 GO:0032350 1 16

C0004093 GO:0042354 1 16

C0004093 GO:0032210 1 16

C0004093 GO:0006096 1 16

C0004093 GO:0070668 1 16

C0004093 GO:0019213 1 16

C0004093 GO:0045089 1 16

C0004093 GO:0045088 1 16

C0004093 GO:0070120 1 16

C0004093 GO:0048593 1 16

C0004093 GO:0010595 1 16

C0004093 GO:0010594 2 16

C0004093 GO:0005044 1 16

C0004093 GO:0050995 1 16

C0004093 GO:0050994 2 16

C0004093 GO:0006413 1 16

C0004093 GO:0007172 1 16

C0004093 GO:0005149 1 16

C0004093 GO:0007612 1 16

C0004093 GO:0007162 1 16

C0004093 GO:0051879 1 16

C0004093 GO:0043524 1 16

C0004093 GO:0019915 1 16

C0004093 GO:0016597 1 16

C0004093 GO:0045785 1 16

C0004093 GO:0004407 1 16

C0004093 GO:0002263 2 16

C0004093 GO:0055117 1 16

C0004093 GO:0045667 1 16

C0004093 GO:0045661 1 16

C0004093 GO:0045669 1 16

C0004093 GO:0010522 1 16

C0004093 GO:0033032 1 16

C0004093 GO:0051057 1 16

C0004093 GO:0043966 1 16

C0004093 GO:0051059 2 16

C0004093 GO:0051701 1 16

C0004093 GO:0019827 1 16

C0004093 GO:0002429 1 16

C0004093 GO:0018209 1 16

C0004093 GO:0045109 1 16

C0004093 GO:0006942 1 16

C0004093 GO:0004709 1 16

C0004093 GO:0045841 3 16

C0004093 GO:0008252 1 16

C0004093 GO:0050820 1 16

C0004093 GO:0001764 1 16

C0004093 GO:0005929 1 16

C0004093 GO:0048002 1 16

C0004093 GO:0030286 1 16

C0004093 GO:0051181 1 16

C0004093 GO:0051180 1 16

C0004093 GO:0046915 1 16

C0004093 GO:0008088 1 16

C0004093 GO:0030148 1 16

C0004093 GO:0020037 1 16

C0004093 GO:0032768 1 16

C0004093 GO:0050796 1 16

C0004093 GO:0060070 2 16

C0004093 GO:0046660 1 16

C0004093 GO:0009953 1 16

C0004093 GO:0009952 1 16

C0004093 GO:0006895 1 16

C0004093 GO:0032364 1 16

C0004093 GO:0017080 3 16

C0004093 GO:0043010 1 16

C0004093 GO:0042834 1 16

C0004093 GO:0006633 1 16

C0004093 GO:0016790 1 16

C0004093 GO:0019956 1 16

C0004093 GO:0009312 1 16

C0004093 GO:0002718 1 16

C0004093 GO:0060263 1 16

C0004093 GO:0010563 2 16

C0004093 GO:0001709 2 16

C0004093 GO:0050768 1 16

C0004093 GO:0001530 1 16

C0004093 GO:0010769 1 16

C0004093 GO:0005355 1 16

C0004093 GO:0042169 2 16

C0004093 GO:0008034 3 16

C0004093 GO:0007131 1 16

C0004093 GO:0002460 1 16

C0004093 GO:0002237 1 16

C0004093 GO:0045619 1 16

C0004093 GO:0034405 1 16

C0004093 GO:0005518 1 16

C0004093 GO:0016409 1 16

C0004093 GO:0004177 1 16

C0004093 GO:0016331 1 16

C0004093 GO:0030323 1 16

C0004093 GO:0050864 1 16

C0004093 GO:0016338 1 16

C0004093 GO:0009108 1 16

C0004093 GO:0034623 1 16

C0004093 GO:0002724 1 16

C0004093 GO:0004675 1 16

C0004093 GO:0030900 1 16

C0004093 GO:0045936 2 16

C0004093 GO:0043627 1 16

C0004093 GO:0007266 1 16

C0004093 GO:0009746 1 16

C0004093 GO:0005605 1 16

C0004093 GO:0006493 1 16

C0004093 GO:0002793 1 16

C0004093 GO:0051923 1 16

C0004093 GO:0003705 2 16

C0004093 GO:0009749 1 16

C0004093 GO:0008645 1 16

C0004093 GO:0060538 1 16

C0004093 GO:0044403 1 16

C0004093 GO:0046697 1 16

C0004093 GO:0031970 1 16

C0004093 GO:0021510 1 16

C0004093 GO:0006477 1 16

C0004093 GO:0030509 1 16

C0004093 GO:0031057 1 16

C0004093 GO:0031058 1 16

C0004093 GO:0015085 2 16

C0004093 GO:0005072 1 16

C0004093 GO:0048864 1 16

C0004093 GO:0005770 2 16

C0004093 GO:0042562 1 16

C0004093 GO:0070001 2 16

C0004093 GO:0010721 1 16

C0004093 GO:0046870 1 16

C0004093 GO:0032722 1 16

C0004093 GO:0070228 1 16

C0004093 GO:0030427 1 16

C0004093 GO:0005159 1 16

C0004093 GO:0005158 1 16

C0004093 GO:0042129 1 16

C0004093 GO:0043536 1 16

C0004093 GO:0043535 2 16

C0004093 GO:0007173 2 16

C0004093 GO:0046631 3 16

C0004093 GO:0070875 2 16

C0004093 GO:0060395 1 16

C0004093 GO:0016055 1 16

C0004093 GO:0060393 1 16

C0004093 GO:0002377 1 16

C0004093 GO:0050927 2 16

C0004093 GO:0004551 1 16

C0004093 GO:0033558 1 16

C0004093 GO:0045494 1 16

C0004093 GO:0042398 1 16

C0004093 GO:0021915 1 16

C0004093 GO:0000786 1 16

C0004093 GO:0031577 3 16

C0004093 GO:0090101 1 16

C0004093 GO:0043548 2 16

C0004093 GO:0046520 1 16

C0004093 GO:0045725 2 16

C0004093 GO:0045727 2 16

C0004093 GO:0030879 1 16

C0004093 GO:0030371 1 16

C0004093 GO:0030276 1 16

C0004093 GO:0005930 1 16

C0004093 GO:0044275 1 16

C0004093 GO:0002757 1 16

C0004093 GO:0008091 2 16

C0004093 GO:0016830 1 16

C0004093 GO:0043120 1 16

C0004093 GO:0008301 1 16

C0004093 GO:0032770 1 16

C0004093 GO:0010921 1 16

C0004093 GO:0017022 1 16

C0004093 GO:0045580 1 16

C0004093 GO:0045429 1 16

C0004093 GO:0046677 1 16

C0004093 GO:0042572 1 16

C0004093 GO:0032374 1 16

C0004093 GO:0032376 3 16

C0004093 GO:0032371 1 16

C0004093 GO:0032370 3 16

C0004093 GO:0032373 3 16

C0004093 GO:0050819 1 16

C0004093 GO:0032273 1 16

C0004093 GO:0048332 1 16

C0004093 GO:0031330 1 16

C0004093 GO:0031333 1 16

C0004093 GO:0000217 1 16

C0004093 GO:0005593 1 16

C0004093 GO:0050771 1 16

C0004093 GO:0050770 2 16

C0004093 GO:0050671 1 16

C0004093 GO:0005024 1 16

C0004093 GO:0001818 1 16

C0004093 GO:0009187 1 16

C0004093 GO:0042119 1 16

C0004093 GO:0005160 1 16

C0004093 GO:0042113 1 16

C0004093 GO:0005436 1 16

C0004093 GO:0043506 1 16

C0004093 GO:0043507 1 16

C0004093 GO:0032986 1 16

C0004093 GO:0032984 1 16

C0004093 GO:0043467 1 16

C0004093 GO:0045766 1 16

C0004093 GO:0002573 1 16

C0004093 GO:0015758 1 16

C0004093 GO:0015695 1 16

C0004093 GO:0050851 1 16

C0004093 GO:0050852 1 16

C0004093 GO:0009112 2 16

C0004093 GO:0009110 1 16

C0004093 GO:0003785 1 16

C0004093 GO:0051646 1 16

C0004093 GO:0034185 1 16

C0004093 GO:0016877 1 16

C0004093 GO:0045123 1 16

C0004093 GO:0045124 1 16

C0004093 GO:0007098 1 16

C0004093 GO:0007094 3 16

C0004093 GO:0048638 1 16

C0004093 GO:0050681 1 16

C0004093 GO:0030426 1 16

C0004093 GO:0008585 1 16

C0004093 GO:0050926 2 16

C0004093 GO:0055072 1 16

C0004093 GO:0051893 1 16

C0004093 GO:0008375 2 16

C0004093 GO:0051897 1 16

C0004093 GO:0051896 1 16

C0004093 GO:0007519 1 16

C0004093 GO:0044419 1 16

C0004093 GO:0043325 2 16

C0004093 GO:0010896 1 16

C0004093 GO:0010898 1 16

C0004093 GO:0045839 3 16

C0004093 GO:0030510 1 16

C0004093 GO:0032934 1 16

C0004093 GO:0006760 1 16

C0004093 GO:0030048 1 16

C0004093 GO:0010975 2 16

C0004093 GO:0030514 1 16

C0004093 GO:0010874 2 16

C0004093 GO:0010875 3 16

C0004093 GO:0006041 1 16

C0004093 GO:0006044 1 16

C0004093 GO:0051353 2 16

C0004093 GO:0043034 1 16

C0004093 GO:0030194 1 16

C0004093 GO:0007632 1 16

C0004093 GO:0019934 1 16

C0004093 GO:0051453 1 16

C0004093 GO:0034103 1 16

C0004093 GO:0005871 1 16

C0004093 GO:0032496 1 16

C0004093 GO:0048488 2 16

C0004093 GO:0007062 2 16

C0004093 GO:0016049 1 16

C0004093 GO:0002285 1 16

C0004093 GO:0004428 1 16

C0004093 GO:0045646 1 16

C0004093 GO:0050700 2 16

C0004093 GO:0015149 1 16

C0004093 GO:0002366 2 16

C0004093 GO:0015145 1 16

C0004093 GO:0071174 3 16

C0004093 GO:0046545 1 16

C0004093 GO:0071173 3 16

C0004093 GO:0030890 1 16

C0004093 GO:0008013 1 16

C0004093 GO:0048255 1 16

C0004093 GO:0043560 1 16

C0004093 GO:0042102 1 16

C0004093 GO:0045165 1 16

C0004093 GO:0042734 1 16

C0004093 GO:0030863 1 16

C0004093 GO:0030864 1 16

C0004093 GO:0033261 1 16

C0004093 GO:0045730 1 16

C0004093 GO:0045737 1 16

C0004093 GO:0030195 1 16

C0004093 GO:0005041 1 16

C0004093 GO:0050808 1 16

C0004093 GO:0005901 1 16

C0004093 GO:0005903 1 16

C0004093 GO:0033280 1 16

C0004093 GO:0009124 2 16

C0004093 GO:0046782 1 16

C0004093 GO:0046467 1 16

C0004093 GO:0070688 1 16

C0004093 GO:0015807 3 16

C0004093 GO:0001894 1 16

C0004093 GO:0001893 1 16

C0004093 GO:0006081 1 16

C0004093 GO:0043603 1 16

C0004093 GO:0045861 1 16

C0004093 GO:0019200 1 16

C0004093 GO:0046326 1 16

C0004093 GO:0010948 1 16

C0004093 GO:0046112 1 16

C0004093 GO:0030169 1 16

C0004093 GO:0003707 1 16

C0004093 GO:0030641 1 16

C0004093 GO:0045684 1 16

C0004093 GO:0030520 1 16

C0004093 GO:0070412 1 16

C0004093 GO:0070410 3 16

C0004093 GO:0010741 1 16

C0004093 GO:0005697 1 16

C0004093 GO:0034451 1 16

C0004093 GO:0048662 1 16

C0004093 GO:0048660 1 16

C0004093 GO:0033483 1 16

C0004093 GO:0005884 1 16

C0004093 GO:0002440 2 16

C0004093 GO:0002286 1 16

C0004093 GO:0034637 1 16

C0004093 GO:0002250 1 16

C0004093 GO:0015749 1 16

C0004093 GO:0001937 1 16

C0009806 GO:0051043 1 9

C0009806 GO:0051044 1 9

C0009806 GO:0030228 2 9

C0009806 GO:0032107 1 9

C0009806 GO:0004864 1 9

C0009806 GO:0046027 1 9

C0009806 GO:0032387 2 9

C0009806 GO:0019228 1 9

C0009806 GO:0021510 1 9

C0009806 GO:0006477 1 9

C0009806 GO:0048385 1 9

C0009806 GO:0030509 1 9

C0009806 GO:0050926 1 9

C0009806 GO:0050927 1 9

C0009806 GO:0043524 1 9

C0009806 GO:0043525 1 9

C0009806 GO:0002703 1 9

C0009806 GO:0015695 1 9

C0009806 GO:0009593 1 9

C0009806 GO:0046631 1 9

C0009806 GO:0016863 2 9

C0009806 GO:0046131 1 9

C0009806 GO:0016860 1 9

C0009806 GO:0015085 1 9

C0009806 GO:0031985 1 9

C0009806 GO:0043206 1 9

C0009806 GO:0005540 1 9

C0009806 GO:0005770 1 9

C0009806 GO:0016338 1 9

C0009806 GO:0007217 1 9

C0009806 GO:0018210 1 9

C0009806 GO:0001893 1 9

C0009806 GO:0044419 1 9

C0009806 GO:0033238 1 9

C0009806 GO:0004712 2 9

C0009806 GO:0010721 1 9

C0009806 GO:0006942 1 9

C0009806 GO:0045839 1 9

C0009806 GO:0051897 1 9

C0009806 GO:0010875 1 9

C0009806 GO:0043120 2 9

C0009806 GO:0033032 1 9

C0009806 GO:0070228 1 9

C0009806 GO:0051055 1 9

C0009806 GO:0005796 1 9

C0009806 GO:0015697 1 9

C0009806 GO:0006937 1 9

C0009806 GO:0031960 1 9

C0009806 GO:0006769 1 9

C0009806 GO:0042129 1 9

C0009806 GO:0051058 1 9

C0009806 GO:0007281 1 9

C0009806 GO:0001516 1 9

C0009806 GO:0043535 1 9

C0009806 GO:0007173 1 9

C0009806 GO:0005788 1 9

C0009806 GO:0016894 1 9

C0009806 GO:0048641 1 9

C0009806 GO:0051702 1 9

C0009806 GO:0051701 1 9

C0009806 GO:0042992 2 9

C0009806 GO:0010874 1 9

C0009806 GO:0030169 1 9

C0009806 GO:0031343 1 9

C0009806 GO:0042326 1 9

C0009806 GO:0042994 1 9

C0009806 GO:0034284 1 9

C0009806 GO:0016812 1 9

C0009806 GO:0016055 2 9

C0009806 GO:0045429 1 9

C0009806 GO:0051353 1 9

C0009806 GO:0016566 1 9

C0009806 GO:0046887 2 9

C0009806 GO:0051784 1 9

C0009806 GO:0005507 1 9

C0009806 GO:0004709 1 9

C0009806 GO:0004708 2 9

C0009806 GO:0021537 2 9

C0009806 GO:0050715 1 9

C0009806 GO:0032410 1 9

C0009806 GO:0046006 1 9

C0009806 GO:0045739 1 9

C0009806 GO:0001953 1 9

C0009806 GO:0006458 1 9

C0009806 GO:0019882 1 9

C0009806 GO:0046716 1 9

C0009806 GO:0001763 1 9

C0009806 GO:0046496 1 9

C0009806 GO:0005929 1 9

C0009806 GO:0048002 1 9

C0009806 GO:0032496 1 9

C0009806 GO:0002822 2 9

C0009806 GO:0021700 1 9

C0009806 GO:0030178 1 9

C0009806 GO:0048488 1 9

C0009806 GO:0007062 1 9

C0009806 GO:0031577 1 9

C0009806 GO:0031575 1 9

C0009806 GO:0002286 1 9

C0009806 GO:0043547 1 9

C0009806 GO:0002285 1 9

C0009806 GO:0016045 1 9

C0009806 GO:0043087 1 9

C0009806 GO:0031228 1 9

C0009806 GO:0050700 2 9

C0009806 GO:0005858 1 9

C0009806 GO:0045981 1 9

C0009806 GO:0015149 1 9

C0009806 GO:0019047 1 9

C0009806 GO:0009880 1 9

C0009806 GO:0002366 1 9

C0009806 GO:0019218 1 9

C0009806 GO:0045912 2 9

C0009806 GO:0005520 1 9

C0009806 GO:0045727 2 9

C0009806 GO:0015145 1 9

C0009806 GO:0010812 1 9

C0009806 GO:0030149 1 9

C0009806 GO:0071174 1 9

C0009806 GO:0019883 1 9

C0009806 GO:0071173 1 9

C0009806 GO:0030371 1 9

C0009806 GO:0005138 2 9

C0009806 GO:0008013 1 9

C0009806 GO:0070167 1 9

C0009806 GO:0060070 1 9

C0009806 GO:0030858 1 9

C0009806 GO:0002263 1 9

C0009806 GO:0046870 1 9

C0009806 GO:0016877 1 9

C0009806 GO:0042308 2 9

C0009806 GO:0030069 1 9

C0009806 GO:0042102 1 9

C0009806 GO:0042301 1 9

C0009806 GO:0002715 1 9

C0009806 GO:0005844 1 9

C0009806 GO:0045165 2 9

C0009806 GO:0034765 1 9

C0009806 GO:0050716 1 9

C0009806 GO:0007423 1 9

C0009806 GO:0046906 1 9

C0009806 GO:0030863 1 9

C0009806 GO:0032731 1 9

C0009806 GO:0032364 1 9

C0009806 GO:0050718 1 9

C0009806 GO:0016830 1 9

C0009806 GO:0006213 1 9

C0009806 GO:0060416 1 9

C0009806 GO:0008206 1 9

C0009806 GO:0070567 1 9

C0009806 GO:0017080 1 9

C0009806 GO:0016903 1 9

C0009806 GO:0048742 1 9

C0009806 GO:0046823 2 9

C0009806 GO:0031346 1 9

C0009806 GO:0050810 2 9

C0009806 GO:0006752 1 9

C0009806 GO:0043010 1 9

C0009806 GO:0042834 1 9

C0009806 GO:0055067 1 9

C0009806 GO:0045841 1 9

C0009806 GO:0032104 1 9

C0009806 GO:0050808 1 9

C0009806 GO:0006637 1 9

C0009806 GO:0010921 1 9

C0009806 GO:0048145 2 9

C0009806 GO:0031016 1 9

C0009806 GO:0009311 1 9

C0009806 GO:0016620 1 9

C0009806 GO:0045580 1 9

C0009806 GO:0005901 2 9

C0009806 GO:0046112 1 9

C0009806 GO:0018105 1 9

C0009806 GO:0032373 1 9

C0009806 GO:0005871 1 9

C0009806 GO:0004691 1 9

C0009806 GO:0004690 1 9

C0009806 GO:0051457 1 9

C0009806 GO:0034599 1 9

C0009806 GO:0009124 1 9

C0009806 GO:0001948 1 9

C0009806 GO:0004716 1 9

C0009806 GO:0043560 1 9

C0009806 GO:0032412 1 9

C0009806 GO:0007250 1 9

C0009806 GO:0005583 1 9

C0009806 GO:0032376 1 9

C0009806 GO:0048638 1 9

C0009806 GO:0032370 1 9

C0009806 GO:0050768 1 9

C0009806 GO:0007162 1 9

C0009806 GO:0060260 1 9

C0009806 GO:0015833 1 9

C0009806 GO:0015166 1 9

C0009806 GO:0032273 1 9

C0009806 GO:0046466 1 9

C0009806 GO:0031397 1 9

C0009806 GO:0005506 1 9

C0009806 GO:0050750 1 9

C0009806 GO:0005501 1 9

C0009806 GO:0022614 1 9

C0009806 GO:0005355 1 9

C0009806 GO:0042169 2 9

C0009806 GO:0015296 1 9

C0009806 GO:0035239 1 9

C0009806 GO:0008329 1 9

C0009806 GO:0015459 1 9

C0009806 GO:0015929 1 9

C0009806 GO:0030675 1 9

C0009806 GO:0001654 2 9

C0009806 GO:0001656 1 9

C0009806 GO:0010677 2 9

C0009806 GO:0005593 1 9

C0009806 GO:0010595 1 9

C0009806 GO:0007131 1 9

C0009806 GO:0042562 1 9

C0009806 GO:0051983 1 9

C0009806 GO:0042269 1 9

C0009806 GO:0015665 1 9

C0009806 GO:0008093 1 9

C0009806 GO:0002460 1 9

C0009806 GO:0030804 1 9

C0009806 GO:0050670 1 9

C0009806 GO:0050671 1 9

C0009806 GO:0045940 1 9

C0009806 GO:0002237 2 9

C0009806 GO:0031645 1 9

C0009806 GO:0005518 1 9

C0009806 GO:0019840 1 9

C0009806 GO:0007585 1 9

C0009806 GO:0017046 1 9

C0009806 GO:0000185 1 9

C0009806 GO:0051384 1 9

C0009806 GO:0014910 1 9

C0009806 GO:0046326 2 9

C0009806 GO:0015074 1 9

C0009806 GO:0008286 2 9

C0009806 GO:0017048 1 9

C0009806 GO:0060021 1 9

C0009806 GO:0022407 1 9

C0009806 GO:0010948 1 9

C0009806 GO:0008034 1 9

C0009806 GO:0034384 1 9

C0009806 GO:0034623 1 9

C0009806 GO:0046890 1 9

C0009806 GO:0043506 1 9

C0009806 GO:0002724 1 9

C0009806 GO:0007272 1 9

C0009806 GO:0009749 1 9

C0009806 GO:0010828 2 9

C0009806 GO:0005790 1 9

C0009806 GO:0030801 1 9

C0009806 GO:0032355 1 9

C0009806 GO:0001910 1 9

C0009806 GO:0019362 1 9

C0009806 GO:0030520 1 9

C0009806 GO:0044447 1 9

C0009806 GO:0045682 1 9

C0009806 GO:0032210 1 9

C0009806 GO:0030810 1 9

C0009806 GO:0042787 1 9

C0009806 GO:0030879 1 9

C0009806 GO:0070410 1 9

C0009806 GO:0033189 1 9

C0009806 GO:0007098 1 9

C0009806 GO:0007530 1 9

C0009806 GO:0030317 1 9

C0009806 GO:0043588 1 9

C0009806 GO:0032526 1 9

C0009806 GO:0034451 1 9

C0009806 GO:0048662 2 9

C0009806 GO:0048660 1 9

C0009806 GO:0042104 1 9

C0009806 GO:0048592 1 9

C0009806 GO:0048593 1 9

C0009806 GO:0033483 1 9

C0009806 GO:0009112 1 9

C0009806 GO:0032318 1 9

C0009806 GO:0031214 1 9

C0009806 GO:0046580 1 9

C0009806 GO:0045766 1 9

C0009806 GO:0002718 1 9

C0009806 GO:0042552 1 9

C0009806 GO:0035295 1 9

C0009806 GO:0033293 1 9

C0009806 GO:0001530 1 9

C0009806 GO:0050709 1 9

C0009806 GO:0009746 1 9

C0009806 GO:0030166 1 9

C0009806 GO:0006493 1 9

C0009806 GO:0010594 1 9

C0009806 GO:0043197 1 9

C0009806 GO:0043536 1 9

C0009806 GO:0002793 1 9

C0009806 GO:0051923 1 9

C0009806 GO:0019212 1 9

C0009806 GO:0003707 1 9

C0009806 GO:0046457 1 9

C0009806 GO:0032320 1 9

C0009806 GO:0046883 1 9

C0009806 GO:0030900 2 9

C0009806 GO:0002250 1 9

C0009806 GO:0030902 1 9

C0009806 GO:0006885 1 9

C0009806 GO:0007094 1 9

C0009806 GO:0044403 1 9

C0009806 GO:0046697 1 9

C0009806 GO:0043112 1 9

C0009806 GO:0006413 1 9

C0009806 GO:0008366 1 9

C0023530 GO:0005786 1 6

C0023530 GO:0006775 1 6

C0023530 GO:0009593 1 6

C0023530 GO:0030228 1 6

C0023530 GO:0043954 1 6

C0023530 GO:0010677 1 6

C0023530 GO:0050681 1 6

C0023530 GO:0046631 1 6

C0023530 GO:0030509 1 6

C0023530 GO:0002702 1 6

C0023530 GO:0031057 1 6

C0023530 GO:0016868 1 6

C0023530 GO:0034765 1 6

C0023530 GO:0048260 1 6

C0023530 GO:0001894 1 6

C0023530 GO:0034762 1 6

C0023530 GO:0015085 1 6

C0023530 GO:0005072 1 6

C0023530 GO:0004364 1 6

C0023530 GO:0000002 1 6

C0023530 GO:0005770 2 6

C0023530 GO:0018212 1 6

C0023530 GO:0018210 1 6

C0023530 GO:0055117 1 6

C0023530 GO:0044419 1 6

C0023530 GO:0043325 1 6

C0023530 GO:0004712 1 6

C0023530 GO:0045839 2 6

C0023530 GO:0032722 1 6

C0023530 GO:0010522 1 6

C0023530 GO:0033032 2 6

C0023530 GO:0051057 1 6

C0023530 GO:0032934 1 6

C0023530 GO:0006760 1 6

C0023530 GO:0043966 1 6

C0023530 GO:0005159 1 6

C0023530 GO:0042129 1 6

C0023530 GO:0043534 1 6

C0023530 GO:0007173 2 6

C0023530 GO:0007172 1 6

C0023530 GO:0010975 1 6

C0023530 GO:0045912 1 6

C0023530 GO:0051701 1 6

C0023530 GO:0030514 1 6

C0023530 GO:0070875 1 6

C0023530 GO:0030510 1 6

C0023530 GO:0002429 2 6

C0023530 GO:0032965 1 6

C0023530 GO:0060395 1 6

C0023530 GO:0016055 1 6

C0023530 GO:0005902 1 6

C0023530 GO:0042177 1 6

C0023530 GO:0004551 1 6

C0023530 GO:0051784 2 6

C0023530 GO:0004709 1 6

C0023530 GO:0004708 1 6

C0023530 GO:0006268 1 6

C0023530 GO:0021537 1 6

C0023530 GO:0060263 1 6

C0023530 GO:0002700 1 6

C0023530 GO:0008252 1 6

C0023530 GO:0006929 1 6

C0023530 GO:0046658 1 6

C0023530 GO:0046324 1 6

C0023530 GO:0000786 1 6

C0023530 GO:0021915 1 6

C0023530 GO:0032496 1 6

C0023530 GO:0030071 1 6

C0023530 GO:0048488 2 6

C0023530 GO:0007062 2 6

C0023530 GO:0031577 2 6

C0023530 GO:0051181 1 6

C0023530 GO:0051180 1 6

C0023530 GO:0042551 1 6

C0023530 GO:0004428 1 6

C0023530 GO:0045646 1 6

C0023530 GO:0043548 1 6

C0023530 GO:0005391 1 6

C0023530 GO:0045725 1 6

C0023530 GO:0051131 1 6

C0023530 GO:0071174 2 6

C0023530 GO:0071173 2 6

C0023530 GO:0005071 2 6

C0023530 GO:0030371 1 6

C0023530 GO:0048500 1 6

C0023530 GO:0005138 1 6

C0023530 GO:0008013 1 6

C0023530 GO:0060070 2 6

C0023530 GO:0030276 1 6

C0023530 GO:0048255 1 6

C0023530 GO:0005930 1 6

C0023530 GO:0030675 1 6

C0023530 GO:0042439 1 6

C0023530 GO:0009952 1 6

C0023530 GO:0030863 1 6

C0023530 GO:0050650 1 6

C0023530 GO:0032365 1 6

C0023530 GO:0050654 1 6

C0023530 GO:0033261 1 6

C0023530 GO:0017080 1 6

C0023530 GO:0045737 1 6

C0023530 GO:0008301 1 6

C0023530 GO:0015269 1 6

C0023530 GO:0045841 2 6

C0023530 GO:0035113 1 6

C0023530 GO:0046579 1 6

C0023530 GO:0009312 1 6

C0023530 GO:0045580 1 6

C0023530 GO:0005901 1 6

C0023530 GO:0031228 2 6

C0023530 GO:0006942 1 6

C0023530 GO:0043393 1 6

C0023530 GO:0051453 1 6

C0023530 GO:0005871 1 6

C0023530 GO:0033280 1 6

C0023530 GO:0048385 1 6

C0023530 GO:0009124 1 6

C0023530 GO:0031225 1 6

C0023530 GO:0043560 1 6

C0023530 GO:0043489 1 6

C0023530 GO:0001709 1 6

C0023530 GO:0008308 1 6

C0023530 GO:0015370 1 6

C0023530 GO:0045742 1 6

C0023530 GO:0032273 1 6

C0023530 GO:0010828 1 6

C0023530 GO:0022612 1 6

C0023530 GO:0008286 1 6

C0023530 GO:0008034 1 6

C0023530 GO:0031330 1 6

C0023530 GO:0070688 1 6

C0023530 GO:0034637 1 6

C0023530 GO:0002757 1 6

C0023530 GO:0046870 1 6

C0023530 GO:0007131 1 6

C0023530 GO:0042562 1 6

C0023530 GO:0051983 2 6

C0023530 GO:0050770 1 6

C0023530 GO:0043603 1 6

C0023530 GO:0045669 1 6

C0023530 GO:0050671 1 6

C0023530 GO:0031985 1 6

C0023530 GO:0002237 2 6

C0023530 GO:0045619 1 6

C0023530 GO:0022839 1 6

C0023530 GO:0001818 1 6

C0023530 GO:0017046 1 6

C0023530 GO:0018107 1 6

C0023530 GO:0009895 1 6

C0023530 GO:0030326 1 6

C0023530 GO:0051452 1 6

C0023530 GO:0046326 1 6

C0023530 GO:0019751 1 6

C0023530 GO:0018108 1 6

C0023530 GO:0010948 1 6

C0023530 GO:0045851 1 6

C0023530 GO:0009108 1 6

C0023530 GO:0005436 1 6

C0023530 GO:0034623 1 6

C0023530 GO:0045445 1 6

C0023530 GO:0002724 2 6

C0023530 GO:0002720 1 6

C0023530 GO:0030641 1 6

C0023530 GO:0045768 1 6

C0023530 GO:0032355 1 6

C0023530 GO:0030520 1 6

C0023530 GO:0032210 1 6

C0023530 GO:0045767 1 6

C0023530 GO:0070412 1 6

C0023530 GO:0070410 3 6

C0023530 GO:0007098 1 6

C0023530 GO:0005697 1 6

C0023530 GO:0050851 2 6

C0023530 GO:0050852 2 6

C0023530 GO:0034451 2 6

C0023530 GO:0045089 1 6

C0023530 GO:0007584 1 6

C0023530 GO:0032984 1 6

C0023530 GO:0050690 1 6

C0023530 GO:0042102 1 6

C0023530 GO:0009112 1 6

C0023530 GO:0015175 1 6

C0023530 GO:0002718 2 6

C0023530 GO:0034185 1 6

C0023530 GO:0005605 2 6

C0023530 GO:0006493 1 6

C0023530 GO:0051881 1 6

C0023530 GO:0010827 1 6

C0023530 GO:0003707 1 6

C0023530 GO:0030900 1 6

C0023530 GO:0007094 2 6

C0023530 GO:0044403 1 6

C0027947 GO:0005786 1 8

C0027947 GO:0030228 1 8

C0027947 GO:0032770 1 8

C0027947 GO:0050681 1 8

C0027947 GO:0021510 1 8

C0027947 GO:0046631 1 8

C0027947 GO:0016331 1 8

C0027947 GO:0030509 1 8

C0027947 GO:0007162 1 8

C0027947 GO:0007163 1 8

C0027947 GO:0002702 2 8

C0027947 GO:0031057 2 8

C0027947 GO:0048260 1 8

C0027947 GO:0045767 1 8

C0027947 GO:0016597 1 8

C0027947 GO:0001894 2 8

C0027947 GO:0051453 1 8

C0027947 GO:0005149 1 8

C0027947 GO:0015085 1 8

C0027947 GO:0005072 1 8

C0027947 GO:0006304 1 8

C0027947 GO:0048864 1 8

C0027947 GO:0005770 1 8

C0027947 GO:0018212 1 8

C0027947 GO:0018210 2 8

C0027947 GO:0055117 1 8

C0027947 GO:0044419 1 8

C0027947 GO:0001824 1 8

C0027947 GO:0043325 1 8

C0027947 GO:0045661 1 8

C0027947 GO:0045839 2 8

C0027947 GO:0030510 1 8

C0027947 GO:0051452 1 8

C0027947 GO:0010522 1 8

C0027947 GO:0033032 1 8

C0027947 GO:0051057 1 8

C0027947 GO:0032934 1 8

C0027947 GO:0042439 1 8

C0027947 GO:0043966 2 8

C0027947 GO:0002757 1 8

C0027947 GO:0002720 2 8

C0027947 GO:0007612 1 8

C0027947 GO:0005159 1 8

C0027947 GO:0000090 1 8

C0027947 GO:0051059 1 8

C0027947 GO:0051058 1 8

C0027947 GO:0030048 1 8

C0027947 GO:0043535 1 8

C0027947 GO:0043534 1 8

C0027947 GO:0007173 2 8

C0027947 GO:0010975 1 8

C0027947 GO:0070668 1 8

C0027947 GO:0051701 1 8

C0027947 GO:0030514 1 8

C0027947 GO:0070875 1 8

C0027947 GO:0010874 1 8

C0027947 GO:0010875 2 8

C0027947 GO:0019827 1 8

C0027947 GO:0002429 1 8

C0027947 GO:0006306 1 8

C0027947 GO:0030260 1 8

C0027947 GO:0060395 1 8

C0027947 GO:0016055 1 8

C0027947 GO:0060393 1 8

C0027947 GO:0002377 1 8

C0027947 GO:0042177 1 8

C0027947 GO:0004551 1 8

C0027947 GO:0051784 2 8

C0027947 GO:0035065 1 8

C0027947 GO:0004709 2 8

C0027947 GO:0043034 1 8

C0027947 GO:0019439 1 8

C0027947 GO:0021537 1 8

C0027947 GO:0007632 1 8

C0027947 GO:0060263 1 8

C0027947 GO:0032768 1 8

C0027947 GO:0008252 1 8

C0027947 GO:0006929 1 8

C0027947 GO:0010906 1 8

C0027947 GO:0046658 1 8

C0027947 GO:0043489 1 8

C0027947 GO:0034103 1 8

C0027947 GO:0001764 1 8

C0027947 GO:0000786 2 8

C0027947 GO:0043425 1 8

C0027947 GO:0045089 1 8

C0027947 GO:0051353 1 8

C0027947 GO:0051224 1 8

C0027947 GO:0030071 1 8

C0027947 GO:0048488 3 8

C0027947 GO:0007062 3 8

C0027947 GO:0031577 2 8

C0027947 GO:0030890 1 8

C0027947 GO:0004428 2 8

C0027947 GO:0045646 1 8

C0027947 GO:0043548 2 8

C0027947 GO:0050700 1 8

C0027947 GO:0008088 1 8

C0027947 GO:0005391 1 8

C0027947 GO:0043028 1 8

C0027947 GO:0046850 1 8

C0027947 GO:0006110 2 8

C0027947 GO:0045725 1 8

C0027947 GO:0043025 1 8

C0027947 GO:0030879 1 8

C0027947 GO:0043027 1 8

C0027947 GO:0071174 2 8

C0027947 GO:0071173 2 8

C0027947 GO:0020037 1 8

C0027947 GO:0005071 2 8

C0027947 GO:0030371 1 8

C0027947 GO:0048500 1 8

C0027947 GO:0006305 1 8

C0027947 GO:0008013 2 8

C0027947 GO:0050796 1 8

C0027947 GO:0060070 3 8

C0027947 GO:0004364 1 8

C0027947 GO:0030276 1 8

C0027947 GO:0048255 1 8

C0027947 GO:0000002 1 8

C0027947 GO:0050819 1 8

C0027947 GO:0004029 1 8

C0027947 GO:0044275 1 8

C0027947 GO:0030675 1 8

C0027947 GO:0008091 2 8

C0027947 GO:0009952 1 8

C0027947 GO:0030863 1 8

C0027947 GO:0043983 1 8

C0027947 GO:0050650 1 8

C0027947 GO:0043981 1 8

C0027947 GO:0044241 1 8

C0027947 GO:0050654 1 8

C0027947 GO:0030864 1 8

C0027947 GO:0033261 2 8

C0027947 GO:0017080 2 8

C0027947 GO:0045730 1 8

C0027947 GO:0045737 1 8

C0027947 GO:0015179 1 8

C0027947 GO:0016565 1 8

C0027947 GO:0008301 1 8

C0027947 GO:0043010 1 8

C0027947 GO:0046580 1 8

C0027947 GO:0045841 2 8

C0027947 GO:0032376 2 8

C0027947 GO:0033613 1 8

C0027947 GO:0019956 1 8

C0027947 GO:0010921 1 8

C0027947 GO:0035113 1 8

C0027947 GO:0046579 1 8

C0027947 GO:0017022 1 8

C0027947 GO:0009312 1 8

C0027947 GO:0005901 1 8

C0027947 GO:0031228 1 8

C0027947 GO:0006942 1 8

C0027947 GO:0043393 1 8

C0027947 GO:0052126 1 8

C0027947 GO:0005871 1 8

C0027947 GO:0033280 1 8

C0027947 GO:0031513 1 8

C0027947 GO:0043087 1 8

C0027947 GO:0009124 1 8

C0027947 GO:0031225 1 8

C0027947 GO:0070001 1 8

C0027947 GO:0007127 1 8

C0027947 GO:0043560 1 8

C0027947 GO:0007250 1 8

C0027947 GO:0051322 1 8

C0027947 GO:0032374 1 8

C0027947 GO:0001709 2 8

C0027947 GO:0032371 1 8

C0027947 GO:0032370 2 8

C0027947 GO:0032373 2 8

C0027947 GO:0030705 1 8

C0027947 GO:0045740 1 8

C0027947 GO:0032273 1 8

C0027947 GO:0008385 1 8

C0027947 GO:0002237 1 8

C0027947 GO:0019200 1 8

C0027947 GO:0008034 1 8

C0027947 GO:0006879 1 8

C0027947 GO:0031330 1 8

C0027947 GO:0034637 1 8

C0027947 GO:0008308 1 8

C0027947 GO:0015807 2 8

C0027947 GO:0048385 1 8

C0027947 GO:0046870 1 8

C0027947 GO:0050840 1 8

C0027947 GO:0009953 1 8

C0027947 GO:0007131 2 8

C0027947 GO:0046426 1 8

C0027947 GO:0042562 1 8

C0027947 GO:0051983 2 8

C0027947 GO:0051881 1 8

C0027947 GO:0043470 1 8

C0027947 GO:0043471 1 8

C0027947 GO:0006081 1 8

C0027947 GO:0043982 1 8

C0027947 GO:0051828 1 8

C0027947 GO:0004190 1 8

C0027947 GO:0046718 1 8

C0027947 GO:0005044 1 8

C0027947 GO:0034405 1 8

C0027947 GO:0017046 1 8

C0027947 GO:0018107 1 8

C0027947 GO:0009895 2 8

C0027947 GO:0030323 1 8

C0027947 GO:0030326 1 8

C0027947 GO:0050770 1 8

C0027947 GO:0005160 1 8

C0027947 GO:0001933 1 8

C0027947 GO:0018108 1 8

C0027947 GO:0052192 1 8

C0027947 GO:0007270 1 8

C0027947 GO:0010948 1 8

C0027947 GO:0045851 1 8

C0027947 GO:0009108 1 8

C0027947 GO:0043984 1 8

C0027947 GO:0034623 2 8

C0027947 GO:0043506 1 8

C0027947 GO:0002724 3 8

C0027947 GO:0006096 1 8

C0027947 GO:0030641 1 8

C0027947 GO:0045684 1 8

C0027947 GO:0045768 1 8

C0027947 GO:0032355 2 8

C0027947 GO:0001727 1 8

C0027947 GO:0030520 2 8

C0027947 GO:0032350 1 8

C0027947 GO:0042354 1 8

C0027947 GO:0032210 1 8

C0027947 GO:0002573 1 8

C0027947 GO:0070412 2 8

C0027947 GO:0070410 4 8

C0027947 GO:0007098 1 8

C0027947 GO:0070491 2 8

C0027947 GO:0030195 1 8

C0027947 GO:0005697 1 8

C0027947 GO:0050850 1 8

C0027947 GO:0050851 1 8

C0027947 GO:0050852 1 8

C0027947 GO:0034451 1 8

C0027947 GO:0021915 2 8

C0027947 GO:0070120 1 8

C0027947 GO:0007584 1 8

C0027947 GO:0048593 1 8

C0027947 GO:0032984 1 8

C0027947 GO:0050690 1 8

C0027947 GO:0009112 1 8

C0027947 GO:0050660 1 8

C0027947 GO:0003785 1 8

C0027947 GO:0015175 1 8

C0027947 GO:0002718 3 8

C0027947 GO:0034185 1 8

C0027947 GO:0043547 1 8

C0027947 GO:0005605 2 8

C0027947 GO:0006493 1 8

C0027947 GO:0010594 1 8

C0027947 GO:0045123 1 8

C0027947 GO:0008517 1 8

C0027947 GO:0003705 1 8

C0027947 GO:0045124 1 8

C0027947 GO:0003707 1 8

C0027947 GO:0032320 1 8

C0027947 GO:0044409 1 8

C0027947 GO:0030900 1 8

C0027947 GO:0007094 2 8

C0027947 GO:0044403 1 8

C0027947 GO:0051806 1 8

C0027947 GO:0008170 1 8

C0030193 GO:0051044 1 7

C0030193 GO:0035065 1 7

C0030193 GO:0048638 1 7

C0030193 GO:0046637 1 7

C0030193 GO:0032387 1 7

C0030193 GO:0006477 1 7

C0030193 GO:0030509 1 7

C0030193 GO:0050926 2 7

C0030193 GO:0050927 2 7

C0030193 GO:0045766 2 7

C0030193 GO:0015695 1 7

C0030193 GO:0046631 1 7

C0030193 GO:0016863 2 7

C0030193 GO:0042994 1 7

C0030193 GO:0030228 1 7

C0030193 GO:0015085 1 7

C0030193 GO:0006304 1 7

C0030193 GO:0005770 1 7

C0030193 GO:0007519 1 7

C0030193 GO:0018210 1 7

C0030193 GO:0001893 1 7

C0030193 GO:0044419 1 7

C0030193 GO:0033238 1 7

C0030193 GO:0070875 1 7

C0030193 GO:0045839 2 7

C0030193 GO:0010875 1 7

C0030193 GO:0043120 1 7

C0030193 GO:0033032 2 7

C0030193 GO:0060541 1 7

C0030193 GO:0016202 1 7

C0030193 GO:0032655 1 7

C0030193 GO:0014020 1 7

C0030193 GO:0042129 1 7

C0030193 GO:0051058 1 7

C0030193 GO:0001841 1 7

C0030193 GO:0043535 1 7

C0030193 GO:0007173 1 7

C0030193 GO:0005788 1 7

C0030193 GO:0045725 1 7

C0030193 GO:0030041 1 7

C0030193 GO:0030291 1 7

C0030193 GO:0051701 1 7

C0030193 GO:0042992 1 7

C0030193 GO:0030295 1 7

C0030193 GO:0010874 1 7

C0030193 GO:0030169 1 7

C0030193 GO:0045580 1 7

C0030193 GO:0034284 1 7

C0030193 GO:0016860 1 7

C0030193 GO:0016055 1 7

C0030193 GO:0016814 1 7

C0030193 GO:0051353 1 7

C0030193 GO:0016812 1 7

C0030193 GO:0051784 2 7

C0030193 GO:0004709 1 7

C0030193 GO:0043034 1 7

C0030193 GO:0021537 1 7

C0030193 GO:0005088 1 7

C0030193 GO:0060263 1 7

C0030193 GO:0046006 1 7

C0030193 GO:0001843 1 7

C0030193 GO:0001953 1 7

C0030193 GO:0019882 1 7

C0030193 GO:0009898 1 7

C0030193 GO:0005086 1 7

C0030193 GO:0006700 1 7

C0030193 GO:0001764 1 7

C0030193 GO:0000578 1 7

C0030193 GO:0048002 1 7

C0030193 GO:0048488 1 7

C0030193 GO:0007062 1 7

C0030193 GO:0031577 2 7

C0030193 GO:0002286 1 7

C0030193 GO:0002285 1 7

C0030193 GO:0051897 1 7

C0030193 GO:0050700 1 7

C0030193 GO:0002366 1 7

C0030193 GO:0006112 1 7

C0030193 GO:0005522 1 7

C0030193 GO:0045912 1 7

C0030193 GO:0006688 1 7

C0030193 GO:0045727 1 7

C0030193 GO:0048705 1 7

C0030193 GO:0071174 2 7

C0030193 GO:0019883 1 7

C0030193 GO:0071173 2 7

C0030193 GO:0030371 1 7

C0030193 GO:0008013 1 7

C0030193 GO:0008154 1 7

C0030193 GO:0060070 1 7

C0030193 GO:0002263 1 7

C0030193 GO:0001709 1 7

C0030193 GO:0042308 1 7

C0030193 GO:0042632 1 7

C0030193 GO:0042102 1 7

C0030193 GO:0045616 1 7

C0030193 GO:0060606 1 7

C0030193 GO:0030675 2 7

C0030193 GO:0050716 1 7

C0030193 GO:0042734 1 7

C0030193 GO:0030863 1 7

C0030193 GO:0032731 1 7

C0030193 GO:0032364 1 7

C0030193 GO:0050718 1 7

C0030193 GO:0016830 1 7

C0030193 GO:0017080 1 7

C0030193 GO:0006692 1 7

C0030193 GO:0046823 1 7

C0030193 GO:0050810 1 7

C0030193 GO:0045841 2 7

C0030193 GO:0050808 1 7

C0030193 GO:0048145 1 7

C0030193 GO:0031016 1 7

C0030193 GO:0048634 1 7

C0030193 GO:0035148 1 7

C0030193 GO:0006941 1 7

C0030193 GO:0005901 1 7

C0030193 GO:0045429 1 7

C0030193 GO:0007043 1 7

C0030193 GO:0048029 1 7

C0030193 GO:0005871 1 7

C0030193 GO:0042572 1 7

C0030193 GO:0048385 1 7

C0030193 GO:0009124 1 7

C0030193 GO:0043560 1 7

C0030193 GO:0032376 1 7

C0030193 GO:0032370 1 7

C0030193 GO:0032373 1 7

C0030193 GO:0016459 1 7

C0030193 GO:0032273 1 7

C0030193 GO:0060562 1 7

C0030193 GO:0045622 1 7

C0030193 GO:0005355 1 7

C0030193 GO:0001838 1 7

C0030193 GO:0042169 1 7

C0030193 GO:0008034 1 7

C0030193 GO:0002718 1 7

C0030193 GO:0008329 1 7

C0030193 GO:0009247 1 7

C0030193 GO:0015929 1 7

C0030193 GO:0004861 1 7

C0030193 GO:0045165 1 7

C0030193 GO:0001654 1 7

C0030193 GO:0006929 1 7

C0030193 GO:0045216 1 7

C0030193 GO:0046887 1 7

C0030193 GO:0005593 1 7

C0030193 GO:0010595 1 7

C0030193 GO:0007131 1 7

C0030193 GO:0042562 1 7

C0030193 GO:0051983 1 7

C0030193 GO:0006895 1 7

C0030193 GO:0043467 1 7

C0030193 GO:0050670 1 7

C0030193 GO:0050671 1 7

C0030193 GO:0032862 1 7

C0030193 GO:0002237 1 7

C0030193 GO:0045619 1 7

C0030193 GO:0043370 1 7

C0030193 GO:0005089 1 7

C0030193 GO:0005518 1 7

C0030193 GO:0017046 1 7

C0030193 GO:0030323 1 7

C0030193 GO:0030324 1 7

C0030193 GO:0070228 1 7

C0030193 GO:0060538 1 7

C0030193 GO:0005041 1 7

C0030193 GO:0019209 1 7

C0030193 GO:0017048 1 7

C0030193 GO:0060021 1 7

C0030193 GO:0043506 1 7

C0030193 GO:0010948 1 7

C0030193 GO:0015296 1 7

C0030193 GO:0016338 2 7

C0030193 GO:0046112 1 7

C0030193 GO:0002724 1 7

C0030193 GO:0009749 1 7

C0030193 GO:0055092 1 7

C0030193 GO:0045768 1 7

C0030193 GO:0032355 1 7

C0030193 GO:0030520 1 7

C0030193 GO:0004181 1 7

C0030193 GO:0006693 1 7

C0030193 GO:0032210 1 7

C0030193 GO:0045767 1 7

C0030193 GO:0016538 1 7

C0030193 GO:0042787 1 7

C0030193 GO:0070410 1 7

C0030193 GO:0045604 1 7

C0030193 GO:0007098 1 7

C0030193 GO:0046634 1 7

C0030193 GO:0010677 1 7

C0030193 GO:0034451 1 7

C0030193 GO:0048662 1 7

C0030193 GO:0048660 1 7

C0030193 GO:0042104 1 7

C0030193 GO:0010812 1 7

C0030193 GO:0033483 1 7

C0030193 GO:0009112 1 7

C0030193 GO:0032856 1 7

C0030193 GO:0046580 1 7

C0030193 GO:0031214 1 7

C0030193 GO:0043627 1 7

C0030193 GO:0035295 1 7

C0030193 GO:0001530 1 7

C0030193 GO:0009746 1 7

C0030193 GO:0043190 1 7

C0030193 GO:0006493 1 7

C0030193 GO:0010594 1 7

C0030193 GO:0042345 1 7

C0030193 GO:0043536 1 7

C0030193 GO:0006413 1 7

C0030193 GO:0002793 1 7

C0030193 GO:0051923 1 7

C0030193 GO:0003705 1 7

C0030193 GO:0003707 1 7

C0030193 GO:0032321 1 7

C0030193 GO:0030900 1 7

C0030193 GO:0007094 2 7

C0030193 GO:0044403 1 7

C0030193 GO:0046697 1 7

C0030193 GO:0005161 1 7

C0030193 GO:0015036 1 7

C0002453 GO:0005149 1 6

C0002453 GO:0050840 1 6

C0002453 GO:0046637 1 6

C0002453 GO:0046634 1 6

C0002453 GO:0050681 1 6

C0002453 GO:0021510 1 6

C0002453 GO:0006477 1 6

C0002453 GO:0050926 1 6

C0002453 GO:0050927 1 6

C0002453 GO:0050920 1 6

C0002453 GO:0050921 1 6

C0002453 GO:0031343 1 6

C0002453 GO:0016863 1 6

C0002453 GO:0005071 1 6

C0002453 GO:0005072 1 6

C0002453 GO:0006304 1 6

C0002453 GO:0070665 1 6

C0002453 GO:0005544 1 6

C0002453 GO:0070001 1 6

C0002453 GO:0016459 1 6

C0002453 GO:0010896 1 6

C0002453 GO:0010898 1 6

C0002453 GO:0045839 2 6

C0002453 GO:0030169 1 6

C0002453 GO:0032722 1 6

C0002453 GO:0033032 1 6

C0002453 GO:0016202 1 6

C0002453 GO:0032655 1 6

C0002453 GO:0043966 1 6

C0002453 GO:0014020 1 6

C0002453 GO:0032781 1 6

C0002453 GO:0005313 1 6

C0002453 GO:0001841 1 6

C0002453 GO:0002821 1 6

C0002453 GO:0030166 1 6

C0002453 GO:0035270 1 6

C0002453 GO:0046330 1 6

C0002453 GO:0007173 1 6

C0002453 GO:0030291 1 6

C0002453 GO:0030514 1 6

C0002453 GO:0043536 1 6

C0002453 GO:0070875 2 6

C0002453 GO:0030510 1 6

C0002453 GO:0045580 1 6

C0002453 GO:0034284 2 6

C0002453 GO:0060395 1 6

C0002453 GO:0006044 1 6

C0002453 GO:0005902 1 6

C0002453 GO:0051353 1 6

C0002453 GO:0002377 1 6

C0002453 GO:0046849 1 6

C0002453 GO:0051784 2 6

C0002453 GO:0043030 1 6

C0002453 GO:0043034 1 6

C0002453 GO:0045841 2 6

C0002453 GO:0004035 1 6

C0002453 GO:0032642 1 6

C0002453 GO:0032648 1 6

C0002453 GO:0006929 1 6

C0002453 GO:0032946 1 6

C0002453 GO:0005929 1 6

C0002453 GO:0009068 1 6

C0002453 GO:0030071 1 6

C0002453 GO:0007062 1 6

C0002453 GO:0003205 1 6

C0002453 GO:0031577 2 6

C0002453 GO:0003206 1 6

C0002453 GO:0051180 1 6

C0002453 GO:0004428 1 6

C0002453 GO:0045646 1 6

C0002453 GO:0050700 1 6

C0002453 GO:0005858 1 6

C0002453 GO:0002687 1 6

C0002453 GO:0002685 1 6

C0002453 GO:0006687 1 6

C0002453 GO:0005522 1 6

C0002453 GO:0045725 2 6

C0002453 GO:0006688 1 6

C0002453 GO:0045727 1 6

C0002453 GO:0071174 2 6

C0002453 GO:0071173 2 6

C0002453 GO:0055029 1 6

C0002453 GO:0005161 1 6

C0002453 GO:0035250 1 6

C0002453 GO:0048709 1 6

C0002453 GO:0019319 1 6

C0002453 GO:0050795 1 6

C0002453 GO:0060606 1 6

C0002453 GO:0060070 1 6

C0002453 GO:0030276 1 6

C0002453 GO:0030274 1 6

C0002453 GO:0014003 1 6

C0002453 GO:0042632 1 6

C0002453 GO:0042301 1 6

C0002453 GO:0030675 1 6

C0002453 GO:0009952 1 6

C0002453 GO:0006895 1 6

C0002453 GO:0050650 1 6

C0002453 GO:0002758 1 6

C0002453 GO:0050654 1 6

C0002453 GO:0002218 1 6

C0002453 GO:0006693 1 6

C0002453 GO:0006692 1 6

C0002453 GO:0060538 1 6

C0002453 GO:0003746 1 6

C0002453 GO:0005041 1 6

C0002453 GO:0015172 1 6

C0002453 GO:0050808 1 6

C0002453 GO:0030880 1 6

C0002453 GO:0007519 1 6

C0002453 GO:0048145 1 6

C0002453 GO:0048634 1 6

C0002453 GO:0035148 1 6

C0002453 GO:0006941 1 6

C0002453 GO:0043256 1 6

C0002453 GO:0031228 1 6

C0002453 GO:0007043 1 6

C0002453 GO:0042572 1 6

C0002453 GO:0035295 1 6

C0002453 GO:0043168 1 6

C0002453 GO:0050764 1 6

C0002453 GO:0030201 1 6

C0002453 GO:0050885 1 6

C0002453 GO:0001709 2 6

C0002453 GO:0001843 1 6

C0002453 GO:0022829 1 6

C0002453 GO:0002224 1 6

C0002453 GO:0060562 1 6

C0002453 GO:0045622 1 6

C0002453 GO:0002221 1 6

C0002453 GO:0001838 1 6

C0002453 GO:0042169 1 6

C0002453 GO:0008034 1 6

C0002453 GO:0009247 1 6

C0002453 GO:0015929 1 6

C0002453 GO:0043270 1 6

C0002453 GO:0004861 1 6

C0002453 GO:0046928 1 6

C0002453 GO:0045216 1 6

C0002453 GO:0046887 1 6

C0002453 GO:0051983 1 6

C0002453 GO:0010594 1 6

C0002453 GO:0051092 1 6

C0002453 GO:0050679 1 6

C0002453 GO:0000428 1 6

C0002453 GO:0050671 1 6

C0002453 GO:0032862 1 6

C0002453 GO:0004190 1 6

C0002453 GO:0045619 1 6

C0002453 GO:0032479 1 6

C0002453 GO:0043370 1 6

C0002453 GO:0045616 1 6

C0002453 GO:0005518 1 6

C0002453 GO:0032364 1 6

C0002453 GO:0042119 1 6

C0002453 GO:0046364 1 6

C0002453 GO:0016338 1 6

C0002453 GO:0017048 1 6

C0002453 GO:0043506 1 6

C0002453 GO:0008210 1 6

C0002453 GO:0009108 1 6

C0002453 GO:0046112 1 6

C0002453 GO:0009749 2 6

C0002453 GO:0055092 1 6

C0002453 GO:0045768 1 6

C0002453 GO:0010862 1 6

C0002453 GO:0043467 1 6

C0002453 GO:0044447 1 6

C0002453 GO:0042516 1 6

C0002453 GO:0016830 1 6

C0002453 GO:0045767 1 6

C0002453 GO:0016538 1 6

C0002453 GO:0001764 1 6

C0002453 GO:0070412 1 6

C0002453 GO:0070410 1 6

C0002453 GO:0045604 1 6

C0002453 GO:0031307 1 6

C0002453 GO:0048741 1 6

C0002453 GO:0034612 1 6

C0002453 GO:0007585 1 6

C0002453 GO:0042100 1 6

C0002453 GO:0033483 1 6

C0002453 GO:0009112 1 6

C0002453 GO:0032856 1 6

C0002453 GO:0016903 1 6

C0002453 GO:0046580 1 6

C0002453 GO:0031214 1 6

C0002453 GO:0002440 1 6

C0002453 GO:0034599 1 6

C0002453 GO:0051058 1 6

C0002453 GO:0009746 2 6

C0002453 GO:0000178 1 6

C0002453 GO:0010595 1 6

C0002453 GO:0051881 1 6

C0002453 GO:0040017 1 6

C0002453 GO:0051928 1 6

C0002453 GO:0002793 1 6

C0002453 GO:0051923 1 6

C0002453 GO:0003705 1 6

C0002453 GO:0031526 1 6

C0002453 GO:0032321 1 6

C0002453 GO:0002250 1 6

C0002453 GO:0007094 2 6

C0002453 GO:0016505 1 6

C0002453 GO:0008290 1 6

C0002453 GO:0006940 1 6

C0002453 GO:0048520 1 6

C0040034 GO:0005786 1 4

C0040034 GO:0030276 1 4

C0040034 GO:0030228 1 4

C0040034 GO:0050681 1 4

C0040034 GO:0046631 1 4

C0040034 GO:0009895 1 4

C0040034 GO:0002702 1 4

C0040034 GO:0031057 1 4

C0040034 GO:0048260 1 4

C0040034 GO:0005071 2 4

C0040034 GO:0005072 1 4

C0040034 GO:0004364 1 4

C0040034 GO:0000002 1 4

C0040034 GO:0018212 1 4

C0040034 GO:0018210 1 4

C0040034 GO:0070875 1 4

C0040034 GO:0043325 1 4

C0040034 GO:0046870 1 4

C0040034 GO:0010875 1 4

C0040034 GO:0010522 1 4

C0040034 GO:0051057 1 4

C0040034 GO:0032934 1 4

C0040034 GO:0043966 1 4

C0040034 GO:0005159 1 4

C0040034 GO:0043534 1 4

C0040034 GO:0007173 1 4

C0040034 GO:0010975 1 4

C0040034 GO:0030514 1 4

C0040034 GO:0055117 1 4

C0040034 GO:0006081 1 4

C0040034 GO:0002429 1 4

C0040034 GO:0060395 1 4

C0040034 GO:0060393 1 4

C0040034 GO:0042177 1 4

C0040034 GO:0004551 1 4

C0040034 GO:0051784 1 4

C0040034 GO:0045841 1 4

C0040034 GO:0060263 1 4

C0040034 GO:0008252 1 4

C0040034 GO:0006929 1 4

C0040034 GO:0046658 1 4

C0040034 GO:0000786 1 4

C0040034 GO:0030071 1 4

C0040034 GO:0048488 2 4

C0040034 GO:0007062 1 4

C0040034 GO:0031577 1 4

C0040034 GO:0004428 1 4

C0040034 GO:0045646 1 4

C0040034 GO:0043548 1 4

C0040034 GO:0050700 1 4

C0040034 GO:0005391 1 4

C0040034 GO:0043028 1 4

C0040034 GO:0045725 1 4

C0040034 GO:0030879 1 4

C0040034 GO:0043027 1 4

C0040034 GO:0071174 1 4

C0040034 GO:0071173 1 4

C0040034 GO:0048500 1 4

C0040034 GO:0060070 1 4

C0040034 GO:0042439 1 4

C0040034 GO:0048255 1 4

C0040034 GO:0008308 1 4

C0040034 GO:0002757 1 4

C0040034 GO:0008091 1 4

C0040034 GO:0009952 1 4

C0040034 GO:0030510 1 4

C0040034 GO:0050654 1 4

C0040034 GO:0033261 1 4

C0040034 GO:0017080 1 4

C0040034 GO:0045737 1 4

C0040034 GO:0008301 1 4

C0040034 GO:0001709 1 4

C0040034 GO:0035113 1 4

C0040034 GO:0046579 1 4

C0040034 GO:0017022 1 4

C0040034 GO:0009312 1 4

C0040034 GO:0031228 1 4

C0040034 GO:0006942 1 4

C0040034 GO:0043393 1 4

C0040034 GO:0051453 1 4

C0040034 GO:0051452 1 4

C0040034 GO:0033280 1 4

C0040034 GO:0048385 1 4

C0040034 GO:0009124 1 4

C0040034 GO:0031225 1 4

C0040034 GO:0043489 1 4

C0040034 GO:0032376 1 4

C0040034 GO:0032370 1 4

C0040034 GO:0032373 1 4

C0040034 GO:0008034 1 4

C0040034 GO:0031330 1 4

C0040034 GO:0034637 1 4

C0040034 GO:0015807 1 4

C0040034 GO:0045839 1 4

C0040034 GO:0001894 1 4

C0040034 GO:0051983 1 4

C0040034 GO:0050770 1 4

C0040034 GO:0002237 1 4

C0040034 GO:0050650 1 4

C0040034 GO:0018107 1 4

C0040034 GO:0030326 1 4

C0040034 GO:0018108 1 4

C0040034 GO:0045851 1 4

C0040034 GO:0009108 1 4

C0040034 GO:0034623 1 4

C0040034 GO:0043506 1 4

C0040034 GO:0002724 1 4

C0040034 GO:0002720 1 4

C0040034 GO:0030641 1 4

C0040034 GO:0045768 1 4

C0040034 GO:0032984 1 4

C0040034 GO:0045767 1 4

C0040034 GO:0070412 1 4

C0040034 GO:0070410 2 4

C0040034 GO:0005697 1 4

C0040034 GO:0050851 1 4

C0040034 GO:0050852 1 4

C0040034 GO:0021915 1 4

C0040034 GO:0070120 1 4

C0040034 GO:0007584 1 4

C0040034 GO:0050690 1 4

C0040034 GO:0009112 1 4

C0040034 GO:0015175 1 4

C0040034 GO:0002718 1 4

C0040034 GO:0034185 1 4

C0040034 GO:0005605 2 4

C0040034 GO:0051881 1 4

C0040034 GO:0045123 1 4

C0040034 GO:0007094 1 4

C0022568 GO:0045841 1 1

C0022568 GO:0050681 1 1

C0022568 GO:0009108 1 1

C0022568 GO:0030071 1 1

C0022568 GO:0007062 1 1

C0022568 GO:0005071 1 1

C0022568 GO:0005072 1 1

C0022568 GO:0004428 1 1

C0022568 GO:0045646 1 1

C0022568 GO:0001709 1 1

C0022568 GO:0070412 1 1

C0022568 GO:0070410 1 1

C0022568 GO:0045839 1 1

C0022568 GO:0071174 1 1

C0022568 GO:0071173 1 1

C0022568 GO:0043966 1 1

C0022568 GO:0031577 1 1

C0022568 GO:0060070 1 1

C0022568 GO:0007173 1 1

C0022568 GO:0045725 1 1

C0022568 GO:0030514 1 1

C0022568 GO:0070875 1 1

C0022568 GO:0051983 1 1

C0022568 GO:0030510 1 1

C0022568 GO:0009952 1 1

C0022568 GO:0060395 1 1

C0022568 GO:0050650 1 1

C0022568 GO:0050654 1 1

C0022568 GO:0051784 1 1

C0022568 GO:0007094 1 1

C0022568 GO:0030276 1 1

C0010200 GO:0051044 1 12

C0010200 GO:0045216 1 12

C0010200 GO:0010677 2 12

C0010200 GO:0002702 1 12

C0010200 GO:0016863 2 12

C0010200 GO:0016860 1 12

C0010200 GO:0006306 1 12

C0010200 GO:0006305 1 12

C0010200 GO:0006304 1 12

C0010200 GO:0043206 1 12

C0010200 GO:0018212 1 12

C0010200 GO:0018210 1 12

C0010200 GO:0031513 1 12

C0010200 GO:0004715 1 12

C0010200 GO:0004712 1 12

C0010200 GO:0060541 1 12

C0010200 GO:0006688 1 12

C0010200 GO:0006477 1 12

C0010200 GO:0030291 1 12

C0010200 GO:0030295 1 12

C0010200 GO:0034284 1 12

C0010200 GO:0016812 1 12

C0010200 GO:0016814 1 12

C0010200 GO:0021537 1 12

C0010200 GO:0009895 1 12

C0010200 GO:0009898 1 12

C0010200 GO:0010906 1 12

C0010200 GO:0000578 1 12

C0010200 GO:0051224 1 12

C0010200 GO:0003205 1 12

C0010200 GO:0003206 1 12

C0010200 GO:0043028 1 12

C0010200 GO:0046850 1 12

C0010200 GO:0006112 1 12

C0010200 GO:0006110 2 12

C0010200 GO:0045912 2 12

C0010200 GO:0043025 1 12

C0010200 GO:0043027 1 12

C0010200 GO:0048705 1 12

C0010200 GO:0019882 1 12

C0010200 GO:0019883 1 12

C0010200 GO:0048709 1 12

C0010200 GO:0005138 1 12

C0010200 GO:0060606 1 12

C0010200 GO:0035303 1 12

C0010200 GO:0070567 1 12

C0010200 GO:0015179 1 12

C0010200 GO:0048638 1 12

C0010200 GO:0033613 1 12

C0010200 GO:0048145 1 12

C0010200 GO:0048634 1 12

C0010200 GO:0014003 1 12

C0010200 GO:0051457 1 12

C0010200 GO:0007127 1 12

C0010200 GO:0030705 1 12

C0010200 GO:0016459 1 12

C0010200 GO:0005506 1 12

C0010200 GO:0045622 1 12

C0010200 GO:0008286 2 12

C0010200 GO:0006879 1 12

C0010200 GO:0008329 1 12

C0010200 GO:0015929 1 12

C0010200 GO:0001654 2 12

C0010200 GO:0018108 1 12

C0010200 GO:0001656 1 12

C0010200 GO:0046887 1 12

C0010200 GO:0004190 1 12

C0010200 GO:0018107 1 12

C0010200 GO:0018105 2 12

C0010200 GO:0017048 1 12

C0010200 GO:0060021 1 12

C0010200 GO:0045884 1 12

C0010200 GO:0008210 1 12

C0010200 GO:0007270 1 12

C0010200 GO:0055092 1 12

C0010200 GO:0070491 1 12

C0010200 GO:0032355 1 12

C0010200 GO:0001727 1 12

C0010200 GO:0032350 1 12

C0010200 GO:0042354 1 12

C0010200 GO:0010828 1 12

C0010200 GO:0070665 1 12

C0010200 GO:0070668 1 12

C0010200 GO:0019213 1 12

C0010200 GO:0006700 1 12

C0010200 GO:0021915 1 12

C0010200 GO:0045088 1 12

C0010200 GO:0070120 1 12

C0010200 GO:0048592 1 12

C0010200 GO:0048593 2 12

C0010200 GO:0032856 1 12

C0010200 GO:0001838 1 12

C0010200 GO:0010595 1 12

C0010200 GO:0010594 2 12

C0010200 GO:0005044 1 12

C0010200 GO:0008517 1 12

C0010200 GO:0032320 1 12

C0010200 GO:0032321 1 12

C0010200 GO:0051806 1 12

C0010200 GO:0006413 1 12

C0010200 GO:0005149 1 12

C0010200 GO:0007612 1 12

C0010200 GO:0007162 1 12

C0010200 GO:0007163 1 12

C0010200 GO:0043524 1 12

C0010200 GO:0043525 1 12

C0010200 GO:0045767 1 12

C0010200 GO:0016597 1 12

C0010200 GO:0004407 1 12

C0010200 GO:0002263 1 12

C0010200 GO:0045667 1 12

C0010200 GO:0045661 1 12

C0010200 GO:0006096 1 12

C0010200 GO:0045669 1 12

C0010200 GO:0033032 1 12

C0010200 GO:0016202 1 12

C0010200 GO:0051059 1 12

C0010200 GO:0042992 1 12

C0010200 GO:0014020 1 12

C0010200 GO:0018209 1 12

C0010200 GO:0004709 1 12

C0010200 GO:0004708 1 12

C0010200 GO:0046718 1 12

C0010200 GO:0001764 1 12

C0010200 GO:0005929 1 12

C0010200 GO:0048002 1 12

C0010200 GO:0030286 1 12

C0010200 GO:0008088 1 12

C0010200 GO:0009880 1 12

C0010200 GO:0020037 1 12

C0010200 GO:0032768 1 12

C0010200 GO:0050796 1 12

C0010200 GO:0060070 1 12

C0010200 GO:0009953 1 12

C0010200 GO:0032364 1 12

C0010200 GO:0017080 1 12

C0010200 GO:0046823 1 12

C0010200 GO:0043010 2 12

C0010200 GO:0042834 1 12

C0010200 GO:0032107 1 12

C0010200 GO:0032104 1 12

C0010200 GO:0016790 1 12

C0010200 GO:0019956 1 12

C0010200 GO:0060263 1 12

C0010200 GO:0007043 1 12

C0010200 GO:0010563 1 12

C0010200 GO:0050768 1 12

C0010200 GO:0005355 1 12

C0010200 GO:0042169 2 12

C0010200 GO:0008034 1 12

C0010200 GO:0007131 1 12

C0010200 GO:0043470 1 12

C0010200 GO:0043471 1 12

C0010200 GO:0002460 2 12

C0010200 GO:0045619 1 12

C0010200 GO:0034405 1 12

C0010200 GO:0045616 1 12

C0010200 GO:0005518 1 12

C0010200 GO:0016409 1 12

C0010200 GO:0016331 1 12

C0010200 GO:0051384 1 12

C0010200 GO:0030324 1 12

C0010200 GO:0016338 2 12

C0010200 GO:0002724 1 12

C0010200 GO:0002720 1 12

C0010200 GO:0004181 1 12

C0010200 GO:0016538 1 12

C0010200 GO:0043588 1 12

C0010200 GO:0045936 1 12

C0010200 GO:0045089 1 12

C0010200 GO:0032318 1 12

C0010200 GO:0043627 1 12

C0010200 GO:0035295 1 12

C0010200 GO:0009746 1 12

C0010200 GO:0043190 1 12

C0010200 GO:0043197 1 12

C0010200 GO:0002793 1 12

C0010200 GO:0051923 1 12

C0010200 GO:0052192 1 12

C0010200 GO:0009749 1 12

C0010200 GO:0044409 1 12

C0010200 GO:0060538 1 12

C0010200 GO:0046697 1 12

C0010200 GO:0032387 1 12

C0010200 GO:0021510 2 12

C0010200 GO:0005788 1 12

C0010200 GO:0015085 1 12

C0010200 GO:0048864 1 12

C0010200 GO:0070001 1 12

C0010200 GO:0010721 1 12

C0010200 GO:0043120 1 12

C0010200 GO:0070228 1 12

C0010200 GO:0032655 1 12

C0010200 GO:0043536 1 12

C0010200 GO:0043535 2 12

C0010200 GO:0016055 1 12

C0010200 GO:0060393 1 12

C0010200 GO:0002377 1 12

C0010200 GO:0033558 1 12

C0010200 GO:0006929 1 12

C0010200 GO:0000786 1 12

C0010200 GO:0009068 1 12

C0010200 GO:0031575 1 12

C0010200 GO:0043547 1 12

C0010200 GO:0043548 1 12

C0010200 GO:0005522 1 12

C0010200 GO:0045727 1 12

C0010200 GO:0030879 1 12

C0010200 GO:0050810 1 12

C0010200 GO:0042308 1 12

C0010200 GO:0050819 1 12

C0010200 GO:0044275 1 12

C0010200 GO:0008091 2 12

C0010200 GO:0007423 1 12

C0010200 GO:0046906 1 12

C0010200 GO:0016830 1 12

C0010200 GO:0032770 1 12

C0010200 GO:0010921 1 12

C0010200 GO:0017022 1 12

C0010200 GO:0045580 1 12

C0010200 GO:0045429 1 12

C0010200 GO:0030323 2 12

C0010200 GO:0007250 1 12

C0010200 GO:0032374 1 12

C0010200 GO:0001709 1 12

C0010200 GO:0032371 1 12

C0010200 GO:0032370 3 12

C0010200 GO:0032373 3 12

C0010200 GO:0060562 1 12

C0010200 GO:0019209 1 12

C0010200 GO:0015459 1 12

C0010200 GO:0031333 1 12

C0010200 GO:0009247 1 12

C0010200 GO:0004861 1 12

C0010200 GO:0005593 1 12

C0010200 GO:0050679 1 12

C0010200 GO:0050671 1 12

C0010200 GO:0051828 1 12

C0010200 GO:0005160 1 12

C0010200 GO:0005161 1 12

C0010200 GO:0042113 1 12

C0010200 GO:0043506 3 12

C0010200 GO:0045768 1 12

C0010200 GO:0045766 1 12

C0010200 GO:0002573 1 12

C0010200 GO:0015695 1 12

C0010200 GO:0045604 1 12

C0010200 GO:0009112 1 12

C0010200 GO:0003785 1 12

C0010200 GO:0002718 1 12

C0010200 GO:0001530 1 12

C0010200 GO:0045123 1 12

C0010200 GO:0045124 1 12

C0010200 GO:0030900 1 12

C0010200 GO:0032376 3 12

C0010200 GO:0035065 1 12

C0010200 GO:0046637 1 12

C0010200 GO:0046634 1 12

C0010200 GO:0030427 1 12

C0010200 GO:0030426 1 12

C0010200 GO:0046631 1 12

C0010200 GO:0050926 1 12

C0010200 GO:0050927 1 12

C0010200 GO:0008375 1 12

C0010200 GO:0051897 1 12

C0010200 GO:0051896 1 12

C0010200 GO:0007519 1 12

C0010200 GO:0043325 1 12

C0010200 GO:0010875 3 12

C0010200 GO:0019827 1 12

C0010200 GO:0031960 1 12

C0010200 GO:0030048 1 12

C0010200 GO:0030041 1 12

C0010200 GO:0042345 1 12

C0010200 GO:0010874 2 12

C0010200 GO:0030169 1 12

C0010200 GO:0006041 1 12

C0010200 GO:0006044 1 12

C0010200 GO:0051353 2 12

C0010200 GO:0046849 1 12

C0010200 GO:0043034 2 12

C0010200 GO:0005089 1 12

C0010200 GO:0005088 1 12

C0010200 GO:0007632 1 12

C0010200 GO:0005086 1 12

C0010200 GO:0032946 1 12

C0010200 GO:0048488 1 12

C0010200 GO:0007062 1 12

C0010200 GO:0002286 1 12

C0010200 GO:0002285 1 12

C0010200 GO:0004428 1 12

C0010200 GO:0050700 2 12

C0010200 GO:0015149 1 12

C0010200 GO:0002366 1 12

C0010200 GO:0006687 1 12

C0010200 GO:0001841 1 12

C0010200 GO:0001843 1 12

C0010200 GO:0015145 1 12

C0010200 GO:0030890 1 12

C0010200 GO:0008013 1 12

C0010200 GO:0008154 1 12

C0010200 GO:0043256 1 12

C0010200 GO:0042632 1 12

C0010200 GO:0045165 1 12

C0010200 GO:0050885 1 12

C0010200 GO:0042734 1 12

C0010200 GO:0030864 1 12

C0010200 GO:0033261 1 12

C0010200 GO:0045730 1 12

C0010200 GO:0030195 1 12

C0010200 GO:0050808 1 12

C0010200 GO:0035148 1 12

C0010200 GO:0006941 1 12

C0010200 GO:0030260 1 12

C0010200 GO:0048029 1 12

C0010200 GO:0052126 1 12

C0010200 GO:0033280 1 12

C0010200 GO:0043087 2 12

C0010200 GO:0009124 1 12

C0010200 GO:0015296 2 12

C0010200 GO:0015807 2 12

C0010200 GO:0001894 1 12

C0010200 GO:0001893 1 12

C0010200 GO:0006081 1 12

C0010200 GO:0043603 1 12

C0010200 GO:0043370 1 12

C0010200 GO:0019200 1 12

C0010200 GO:0046326 1 12

C0010200 GO:0003705 1 12

C0010200 GO:0032862 1 12

C0010200 GO:0046112 1 12

C0010200 GO:0045684 1 12

C0010200 GO:0050660 1 12

C0010200 GO:0070412 1 12

C0010200 GO:0070410 1 12

C0010200 GO:0034103 1 12

C0010200 GO:0048662 1 12

C0010200 GO:0048660 1 12

C0010200 GO:0033483 1 12

C0010200 GO:0031214 1 12

C0010200 GO:0002440 1 12

C0010200 GO:0002250 2 12

C0010200 GO:0030228 1 12

C0010200 GO:0015036 1 12

C0033774 GO:0051043 1 7

C0033774 GO:0052126 1 7

C0033774 GO:0035065 1 7

C0033774 GO:0032107 1 7

C0033774 GO:0046637 1 7

C0033774 GO:0031970 1 7

C0033774 GO:0032387 1 7

C0033774 GO:0007612 2 7

C0033774 GO:0021510 2 7

C0033774 GO:0008585 1 7

C0033774 GO:0007292 1 7

C0033774 GO:0007162 1 7

C0033774 GO:0043524 1 7

C0033774 GO:0043525 1 7

C0033774 GO:0019915 1 7

C0033774 GO:0051893 1 7

C0033774 GO:0031058 1 7

C0033774 GO:0046718 1 7

C0033774 GO:0045767 1 7

C0033774 GO:0016597 1 7

C0033774 GO:0034358 1 7

C0033774 GO:0045216 1 7

C0033774 GO:0006306 1 7

C0033774 GO:0006305 1 7

C0033774 GO:0000002 1 7

C0033774 GO:0043010 2 7

C0033774 GO:0043206 1 7

C0033774 GO:0042992 1 7

C0033774 GO:0007519 2 7

C0033774 GO:0045616 1 7

C0033774 GO:0070001 2 7

C0033774 GO:0033238 1 7

C0033774 GO:0004712 1 7

C0033774 GO:0010896 1 7

C0033774 GO:0010721 1 7

C0033774 GO:0010898 1 7

C0033774 GO:0046504 1 7

C0033774 GO:0010875 1 7

C0033774 GO:0033032 1 7

C0033774 GO:0060541 1 7

C0033774 GO:0016202 1 7

C0033774 GO:0032655 1 7

C0033774 GO:0034364 1 7

C0033774 GO:0034366 1 7

C0033774 GO:0014020 1 7

C0033774 GO:0031960 1 7

C0033774 GO:0005159 1 7

C0033774 GO:0005158 1 7

C0033774 GO:0002821 1 7

C0033774 GO:0030048 1 7

C0033774 GO:0043535 1 7

C0033774 GO:0032373 1 7

C0033774 GO:0009953 1 7

C0033774 GO:0070668 1 7

C0033774 GO:0030041 1 7

C0033774 GO:0030291 1 7

C0033774 GO:0007598 1 7

C0033774 GO:0030295 1 7

C0033774 GO:0010874 1 7

C0033774 GO:0030169 1 7

C0033774 GO:0019827 1 7

C0033774 GO:0055092 1 7

C0033774 GO:0030260 1 7

C0033774 GO:0043025 1 7

C0033774 GO:0016055 1 7

C0033774 GO:0005149 1 7

C0033774 GO:0016814 1 7

C0033774 GO:0051353 1 7

C0033774 GO:0002377 1 7

C0033774 GO:0030262 1 7

C0033774 GO:0007043 1 7

C0033774 GO:0004708 1 7

C0033774 GO:0043034 2 7

C0033774 GO:0030194 1 7

C0033774 GO:0005089 1 7

C0033774 GO:0005088 1 7

C0033774 GO:0007632 1 7

C0033774 GO:0060263 1 7

C0033774 GO:0032642 1 7

C0033774 GO:0050768 1 7

C0033774 GO:0009898 1 7

C0033774 GO:0005086 1 7

C0033774 GO:0019934 1 7

C0033774 GO:0050820 1 7

C0033774 GO:0034103 1 7

C0033774 GO:0002440 1 7

C0033774 GO:0000578 1 7

C0033774 GO:0051224 1 7

C0033774 GO:0031575 1 7

C0033774 GO:0043547 1 7

C0033774 GO:0046915 1 7

C0033774 GO:0006885 1 7

C0033774 GO:0043548 1 7

C0033774 GO:0003705 1 7

C0033774 GO:0008088 1 7

C0033774 GO:0046850 1 7

C0033774 GO:0006112 1 7

C0033774 GO:0006110 1 7

C0033774 GO:0005522 1 7

C0033774 GO:0006688 1 7

C0033774 GO:0051879 1 7

C0033774 GO:0043027 1 7

C0033774 GO:0048705 1 7

C0033774 GO:0046545 1 7

C0033774 GO:0009880 1 7

C0033774 GO:0020037 1 7

C0033774 GO:0032768 1 7

C0033774 GO:0005138 1 7

C0033774 GO:0050796 1 7

C0033774 GO:0008154 1 7

C0033774 GO:0034405 1 7

C0033774 GO:0048255 1 7

C0033774 GO:0042308 1 7

C0033774 GO:0043205 1 7

C0033774 GO:0046660 1 7

C0033774 GO:0050819 1 7

C0033774 GO:0004437 1 7

C0033774 GO:0060606 1 7

C0033774 GO:0009895 2 7

C0033774 GO:0042734 2 7

C0033774 GO:0050714 1 7

C0033774 GO:0030864 1 7

C0033774 GO:0030890 1 7

C0033774 GO:0045732 1 7

C0033774 GO:0045730 1 7

C0033774 GO:0046823 1 7

C0033774 GO:0051384 1 7

C0033774 GO:0015665 1 7

C0033774 GO:0021537 2 7

C0033774 GO:0032104 1 7

C0033774 GO:0019894 1 7

C0033774 GO:0015280 1 7

C0033774 GO:0021536 1 7

C0033774 GO:0019956 1 7

C0033774 GO:0010921 1 7

C0033774 GO:0032770 1 7

C0033774 GO:0019200 1 7

C0033774 GO:0035148 1 7

C0033774 GO:0006941 1 7

C0033774 GO:0043627 1 7

C0033774 GO:0009410 1 7

C0033774 GO:0005903 1 7

C0033774 GO:0046677 1 7

C0033774 GO:0048029 1 7

C0033774 GO:0042632 1 7

C0033774 GO:0004693 1 7

C0033774 GO:0030195 1 7

C0033774 GO:0043087 1 7

C0033774 GO:0045078 1 7

C0033774 GO:0043489 1 7

C0033774 GO:0042274 1 7

C0033774 GO:0032374 1 7

C0033774 GO:0052192 1 7

C0033774 GO:0032371 1 7

C0033774 GO:0032370 1 7

C0033774 GO:0045072 1 7

C0033774 GO:0030705 1 7

C0033774 GO:0016459 1 7

C0033774 GO:0046782 1 7

C0033774 GO:0045661 1 7

C0033774 GO:0045622 1 7

C0033774 GO:0015166 1 7

C0033774 GO:0044275 1 7

C0033774 GO:0008286 2 7

C0033774 GO:0050866 1 7

C0033774 GO:0002718 1 7

C0033774 GO:0043190 1 7

C0033774 GO:0031330 1 7

C0033774 GO:0031331 1 7

C0033774 GO:0015459 1 7

C0033774 GO:0000217 1 7

C0033774 GO:0017134 1 7

C0033774 GO:0045211 1 7

C0033774 GO:0015807 2 7

C0033774 GO:0001654 2 7

C0033774 GO:0006929 2 7

C0033774 GO:0001656 1 7

C0033774 GO:0060562 1 7

C0033774 GO:0008091 2 7

C0033774 GO:0001894 1 7

C0033774 GO:0048634 1 7

C0033774 GO:0007423 1 7

C0033774 GO:0002460 1 7

C0033774 GO:0046906 1 7

C0033774 GO:0018105 1 7

C0033774 GO:0043370 1 7

C0033774 GO:0051828 1 7

C0033774 GO:0032862 1 7

C0033774 GO:0004190 2 7

C0033774 GO:0045619 1 7

C0033774 GO:0005044 1 7

C0033774 GO:0006004 1 7

C0033774 GO:0045862 1 7

C0033774 GO:0045861 1 7

C0033774 GO:0016331 1 7

C0033774 GO:0030323 2 7

C0033774 GO:0030324 1 7

C0033774 GO:0042119 1 7

C0033774 GO:0001843 1 7

C0033774 GO:0005160 1 7

C0033774 GO:0019209 2 7

C0033774 GO:0042169 1 7

C0033774 GO:0017048 1 7

C0033774 GO:0060021 1 7

C0033774 GO:0043506 2 7

C0033774 GO:0015296 2 7

C0033774 GO:0009109 1 7

C0033774 GO:0007270 2 7

C0033774 GO:0006879 1 7

C0033774 GO:0006096 1 7

C0033774 GO:0030137 1 7

C0033774 GO:0048864 1 7

C0033774 GO:0043197 1 7

C0033774 GO:0032984 1 7

C0033774 GO:0001841 1 7

C0033774 GO:0032350 1 7

C0033774 GO:0042354 2 7

C0033774 GO:0004181 1 7

C0033774 GO:0045766 1 7

C0033774 GO:0002573 1 7

C0033774 GO:0016538 1 7

C0033774 GO:0033344 1 7

C0033774 GO:0045604 1 7

C0033774 GO:0030900 1 7

C0033774 GO:0045684 1 7

C0033774 GO:0010741 1 7

C0033774 GO:0043588 2 7

C0033774 GO:0015179 1 7

C0033774 GO:0046634 1 7

C0033774 GO:0045768 1 7

C0033774 GO:0045580 1 7

C0033774 GO:0006700 1 7

C0033774 GO:0021915 1 7

C0033774 GO:0009247 1 7

C0033774 GO:0048592 1 7

C0033774 GO:0048593 2 7

C0033774 GO:0032321 1 7

C0033774 GO:0032856 1 7

C0033774 GO:0032318 1 7

C0033774 GO:0003785 1 7

C0033774 GO:0031214 1 7

C0033774 GO:0051646 1 7

C0033774 GO:0007266 1 7

C0033774 GO:0035295 1 7

C0033774 GO:0001838 1 7

C0033774 GO:0032994 1 7

C0033774 GO:0010594 1 7

C0033774 GO:0042345 1 7

C0033774 GO:0004861 1 7

C0033774 GO:0045123 1 7

C0033774 GO:0016338 1 7

C0033774 GO:0050995 1 7

C0033774 GO:0050994 1 7

C0033774 GO:0045124 1 7

C0033774 GO:0032320 1 7

C0033774 GO:0044409 1 7

C0033774 GO:0002824 1 7

C0033774 GO:0002250 1 7

C0033774 GO:0060538 2 7

C0033774 GO:0032376 1 7

C0033774 GO:0051806 1 7

C0033774 GO:0005161 2 7

C0033774 GO:0001937 1 7

C0033774 GO:0015036 1 7

C0035455 GO:0051043 1 6

C0035455 GO:0051044 1 6

C0035455 GO:0035065 1 6

C0035455 GO:0048638 1 6

C0035455 GO:0031970 1 6

C0035455 GO:0010677 1 6

C0035455 GO:0030427 1 6

C0035455 GO:0030426 1 6

C0035455 GO:0008585 1 6

C0035455 GO:0050926 1 6

C0035455 GO:0050927 1 6

C0035455 GO:0043524 1 6

C0035455 GO:0019915 1 6

C0035455 GO:0051893 1 6

C0035455 GO:0031058 1 6

C0035455 GO:0046631 1 6

C0035455 GO:0016863 2 6

C0035455 GO:0051896 1 6

C0035455 GO:0030228 1 6

C0035455 GO:0015085 1 6

C0035455 GO:0043205 1 6

C0035455 GO:0004407 1 6

C0035455 GO:0002263 1 6

C0035455 GO:0007519 1 6

C0035455 GO:0070001 1 6

C0035455 GO:0045667 1 6

C0035455 GO:0004715 1 6

C0035455 GO:0010896 1 6

C0035455 GO:0010898 1 6

C0035455 GO:0046504 1 6

C0035455 GO:0010875 1 6

C0035455 GO:0030323 1 6

C0035455 GO:0060541 1 6

C0035455 GO:0070228 1 6

C0035455 GO:0032655 1 6

C0035455 GO:0005159 1 6

C0035455 GO:0005158 1 6

C0035455 GO:0043536 1 6

C0035455 GO:0043535 1 6

C0035455 GO:0032373 1 6

C0035455 GO:0006477 1 6

C0035455 GO:0030041 1 6

C0035455 GO:0030295 1 6

C0035455 GO:0010874 1 6

C0035455 GO:0030169 1 6

C0035455 GO:0018209 1 6

C0035455 GO:0043603 1 6

C0035455 GO:0034284 1 6

C0035455 GO:0016860 1 6

C0035455 GO:0006041 1 6

C0035455 GO:0006044 1 6

C0035455 GO:0016814 1 6

C0035455 GO:0051353 1 6

C0035455 GO:0016812 1 6

C0035455 GO:0005903 1 6

C0035455 GO:0043627 1 6

C0035455 GO:0033558 1 6

C0035455 GO:0030194 1 6

C0035455 GO:0005089 1 6

C0035455 GO:0005088 1 6

C0035455 GO:0009895 1 6

C0035455 GO:0009898 1 6

C0035455 GO:0005086 1 6

C0035455 GO:0019934 1 6

C0035455 GO:0050820 1 6

C0035455 GO:0000578 1 6

C0035455 GO:0008375 1 6

C0035455 GO:0048002 1 6

C0035455 GO:0030286 1 6

C0035455 GO:0015145 1 6

C0035455 GO:0002286 1 6

C0035455 GO:0046915 1 6

C0035455 GO:0051897 1 6

C0035455 GO:0050700 1 6

C0035455 GO:0015149 1 6

C0035455 GO:0002366 1 6

C0035455 GO:0006112 1 6

C0035455 GO:0045912 1 6

C0035455 GO:0045727 1 6

C0035455 GO:0051879 1 6

C0035455 GO:0005788 1 6

C0035455 GO:0048705 1 6

C0035455 GO:0019882 1 6

C0035455 GO:0019883 1 6

C0035455 GO:0046545 1 6

C0035455 GO:0008154 1 6

C0035455 GO:0050810 1 6

C0035455 GO:0048255 1 6

C0035455 GO:0035303 1 6

C0035455 GO:0046660 1 6

C0035455 GO:0004437 1 6

C0035455 GO:0045165 1 6

C0035455 GO:0042734 1 6

C0035455 GO:0050714 1 6

C0035455 GO:0032364 1 6

C0035455 GO:0016830 1 6

C0035455 GO:0043120 1 6

C0035455 GO:0021537 1 6

C0035455 GO:0050808 1 6

C0035455 GO:0016790 1 6

C0035455 GO:0018212 1 6

C0035455 GO:0048145 1 6

C0035455 GO:0002440 2 6

C0035455 GO:0045429 1 6

C0035455 GO:0060263 1 6

C0035455 GO:0046677 1 6

C0035455 GO:0048029 1 6

C0035455 GO:0004693 1 6

C0035455 GO:0033280 1 6

C0035455 GO:0009124 1 6

C0035455 GO:0045078 1 6

C0035455 GO:0043489 1 6

C0035455 GO:0010563 1 6

C0035455 GO:0032370 1 6

C0035455 GO:0045072 1 6

C0035455 GO:0046782 1 6

C0035455 GO:0005355 1 6

C0035455 GO:0008286 1 6

C0035455 GO:0015296 1 6

C0035455 GO:0008329 1 6

C0035455 GO:0031330 1 6

C0035455 GO:0031333 1 6

C0035455 GO:0000217 1 6

C0035455 GO:0015929 1 6

C0035455 GO:0042113 1 6

C0035455 GO:0045211 1 6

C0035455 GO:0015807 1 6

C0035455 GO:0001654 1 6

C0035455 GO:0002285 1 6

C0035455 GO:0046887 1 6

C0035455 GO:0005593 1 6

C0035455 GO:0001893 1 6

C0035455 GO:0002460 1 6

C0035455 GO:0045669 1 6

C0035455 GO:0004190 1 6

C0035455 GO:0045861 1 6

C0035455 GO:0005518 1 6

C0035455 GO:0016409 1 6

C0035455 GO:0018105 1 6

C0035455 GO:0030324 1 6

C0035455 GO:0051923 1 6

C0035455 GO:0019209 1 6

C0035455 GO:0042169 1 6

C0035455 GO:0018108 1 6

C0035455 GO:0060021 1 6

C0035455 GO:0045884 1 6

C0035455 GO:0008034 1 6

C0035455 GO:0016338 1 6

C0035455 GO:0046112 1 6

C0035455 GO:0004181 1 6

C0035455 GO:0045766 1 6

C0035455 GO:0015695 1 6

C0035455 GO:0010741 1 6

C0035455 GO:0019213 1 6

C0035455 GO:0006700 1 6

C0035455 GO:0048662 1 6

C0035455 GO:0045088 1 6

C0035455 GO:0048660 1 6

C0035455 GO:0045936 1 6

C0035455 GO:0033483 1 6

C0035455 GO:0009112 1 6

C0035455 GO:0051646 1 6

C0035455 GO:0007266 1 6

C0035455 GO:0001530 1 6

C0035455 GO:0009746 1 6

C0035455 GO:0043190 1 6

C0035455 GO:0010595 1 6

C0035455 GO:0010594 1 6

C0035455 GO:0042345 1 6

C0035455 GO:0002793 1 6

C0035455 GO:0050995 1 6

C0035455 GO:0050994 1 6

C0035455 GO:0009749 1 6

C0035455 GO:0002250 1 6

C0035455 GO:0060538 1 6

C0035455 GO:0032376 1 6

C0035455 GO:0046697 1 6

C0035455 GO:0006413 1 6

C0035455 GO:0001937 1 6

C0035455 GO:0015036 1 6

C0039231 GO:0051043 1 8

C0039231 GO:0009593 1 8

C0039231 GO:0035065 1 8

C0039231 GO:0046637 1 8

C0039231 GO:0010677 1 8

C0039231 GO:0019228 1 8

C0039231 GO:0007162 1 8

C0039231 GO:0043524 1 8

C0039231 GO:0003015 1 8

C0039231 GO:0034199 1 8

C0039231 GO:0030345 1 8

C0039231 GO:0008374 1 8

C0039231 GO:0034765 1 8

C0039231 GO:0031343 1 8

C0039231 GO:0016864 1 8

C0039231 GO:0016863 1 8

C0039231 GO:0045981 1 8

C0039231 GO:0031346 1 8

C0039231 GO:0031985 1 8

C0039231 GO:0002703 1 8

C0039231 GO:0005540 1 8

C0039231 GO:0007519 1 8

C0039231 GO:0007217 1 8

C0039231 GO:0030295 2 8

C0039231 GO:0004716 1 8

C0039231 GO:0050715 1 8

C0039231 GO:0016459 1 8

C0039231 GO:0004712 1 8

C0039231 GO:0006073 1 8

C0039231 GO:0031397 1 8

C0039231 GO:0046870 1 8

C0039231 GO:0030858 1 8

C0039231 GO:0043120 1 8

C0039231 GO:0033032 1 8

C0039231 GO:0060541 1 8

C0039231 GO:0016202 1 8

C0039231 GO:0051055 1 8

C0039231 GO:0032655 1 8

C0039231 GO:0005790 1 8

C0039231 GO:0014020 2 8

C0039231 GO:0006769 1 8

C0039231 GO:0007281 2 8

C0039231 GO:0001841 2 8

C0039231 GO:0008170 1 8

C0039231 GO:0032373 1 8

C0039231 GO:0008366 1 8

C0039231 GO:0010975 1 8

C0039231 GO:0007286 1 8

C0039231 GO:0030041 1 8

C0039231 GO:0030291 1 8

C0039231 GO:0030512 1 8

C0039231 GO:0030296 1 8

C0039231 GO:0030169 2 8

C0039231 GO:0045580 2 8

C0039231 GO:0042326 1 8

C0039231 GO:0055092 1 8

C0039231 GO:0034284 1 8

C0039231 GO:0016860 1 8

C0039231 GO:0003785 1 8

C0039231 GO:0015833 1 8

C0039231 GO:0016814 1 8

C0039231 GO:0016566 1 8

C0039231 GO:0001910 1 8

C0039231 GO:0003995 1 8

C0039231 GO:0005507 1 8

C0039231 GO:0004708 1 8

C0039231 GO:0043034 1 8

C0039231 GO:0005089 1 8

C0039231 GO:0005088 1 8

C0039231 GO:0060263 1 8

C0039231 GO:0032410 1 8

C0039231 GO:0032412 1 8

C0039231 GO:0001843 2 8

C0039231 GO:0045739 1 8

C0039231 GO:0006458 1 8

C0039231 GO:0009898 1 8

C0039231 GO:0005086 1 8

C0039231 GO:0001763 1 8

C0039231 GO:0046496 1 8

C0039231 GO:0000578 1 8

C0039231 GO:0005929 1 8

C0039231 GO:0031334 1 8

C0039231 GO:0032496 1 8

C0039231 GO:0002822 2 8

C0039231 GO:0021700 1 8

C0039231 GO:0030178 1 8

C0039231 GO:0007530 1 8

C0039231 GO:0001838 2 8

C0039231 GO:0051181 1 8

C0039231 GO:0050709 1 8

C0039231 GO:0016045 1 8

C0039231 GO:0008543 1 8

C0039231 GO:0031672 1 8

C0039231 GO:0050700 2 8

C0039231 GO:0005858 1 8

C0039231 GO:0016862 1 8

C0039231 GO:0015149 1 8

C0039231 GO:0019047 1 8

C0039231 GO:0006112 1 8

C0039231 GO:0019218 1 8

C0039231 GO:0005522 1 8

C0039231 GO:0045912 1 8

C0039231 GO:0006688 1 8

C0039231 GO:0045727 1 8

C0039231 GO:0015145 1 8

C0039231 GO:0030879 2 8

C0039231 GO:0002688 1 8

C0039231 GO:0048705 1 8

C0039231 GO:0030149 1 8

C0039231 GO:0016894 1 8

C0039231 GO:0032769 1 8

C0039231 GO:0070167 1 8

C0039231 GO:0005138 1 8

C0039231 GO:0008154 1 8

C0039231 GO:0042177 1 8

C0039231 GO:0050810 1 8

C0039231 GO:0016877 1 8

C0039231 GO:0042632 1 8

C0039231 GO:0030069 1 8

C0039231 GO:0005930 1 8

C0039231 GO:0016597 1 8

C0039231 GO:0044042 1 8

C0039231 GO:0060606 2 8

C0039231 GO:0005518 1 8

C0039231 GO:0042269 1 8

C0039231 GO:0042734 1 8

C0039231 GO:0031645 1 8

C0039231 GO:0032364 1 8

C0039231 GO:0016830 1 8

C0039231 GO:0034384 1 8

C0039231 GO:0060416 1 8

C0039231 GO:0008206 1 8

C0039231 GO:0070567 1 8

C0039231 GO:0045730 1 8

C0039231 GO:0048742 1 8

C0039231 GO:0002690 1 8

C0039231 GO:0008034 1 8

C0039231 GO:0042834 1 8

C0039231 GO:0002715 1 8

C0039231 GO:0060260 1 8

C0039231 GO:0050808 1 8

C0039231 GO:0006637 1 8

C0039231 GO:0051702 1 8

C0039231 GO:0019320 1 8

C0039231 GO:0048145 2 8

C0039231 GO:0015074 1 8

C0039231 GO:0009311 1 8

C0039231 GO:0048634 1 8

C0039231 GO:0035148 2 8

C0039231 GO:0006941 1 8

C0039231 GO:0031228 1 8

C0039231 GO:0006942 2 8

C0039231 GO:0007043 1 8

C0039231 GO:0048029 1 8

C0039231 GO:0048641 1 8

C0039231 GO:0004691 1 8

C0039231 GO:0004690 1 8

C0039231 GO:0051457 1 8

C0039231 GO:0034599 1 8

C0039231 GO:0003756 1 8

C0039231 GO:0007250 1 8

C0039231 GO:0005583 1 8

C0039231 GO:0032376 1 8

C0039231 GO:0032370 1 8

C0039231 GO:0030810 1 8

C0039231 GO:0015166 1 8

C0039231 GO:0005520 1 8

C0039231 GO:0046466 1 8

C0039231 GO:0060562 1 8

C0039231 GO:0005506 1 8

C0039231 GO:0045622 1 8

C0039231 GO:0005501 1 8

C0039231 GO:0022614 1 8

C0039231 GO:0044275 1 8

C0039231 GO:0008286 1 8

C0039231 GO:0050866 1 8

C0039231 GO:0030317 1 8

C0039231 GO:0046326 2 8

C0039231 GO:0015929 1 8

C0039231 GO:0004861 1 8

C0039231 GO:0005796 1 8

C0039231 GO:0046883 1 8

C0039231 GO:0004864 1 8

C0039231 GO:0006929 1 8

C0039231 GO:0045216 1 8

C0039231 GO:0046887 2 8

C0039231 GO:0046889 1 8

C0039231 GO:0033293 1 8

C0039231 GO:0008093 2 8

C0039231 GO:0015665 1 8

C0039231 GO:0042345 1 8

C0039231 GO:0060047 1 8

C0039231 GO:0030804 1 8

C0039231 GO:0030801 1 8

C0039231 GO:0016620 1 8

C0039231 GO:0045940 1 8

C0039231 GO:0032862 1 8

C0039231 GO:0002237 1 8

C0039231 GO:0045619 1 8

C0039231 GO:0048246 1 8

C0039231 GO:0043370 1 8

C0039231 GO:0045616 1 8

C0039231 GO:0006007 1 8

C0039231 GO:0007229 1 8

C0039231 GO:0000185 1 8

C0039231 GO:0030323 1 8

C0039231 GO:0030324 1 8

C0039231 GO:0050868 1 8

C0039231 GO:0050770 1 8

C0039231 GO:0046365 1 8

C0039231 GO:0019209 2 8

C0039231 GO:0042169 1 8

C0039231 GO:0017048 2 8

C0039231 GO:0060021 3 8

C0039231 GO:0043506 1 8

C0039231 GO:0015296 1 8

C0039231 GO:0016338 1 8

C0039231 GO:0034623 1 8

C0039231 GO:0046890 1 8

C0039231 GO:0046112 1 8

C0039231 GO:0045682 1 8

C0039231 GO:0007272 1 8

C0039231 GO:0010828 2 8

C0039231 GO:0045768 1 8

C0039231 GO:0009068 1 8

C0039231 GO:0019362 1 8

C0039231 GO:0044447 1 8

C0039231 GO:0004181 1 8

C0039231 GO:0006937 1 8

C0039231 GO:0045766 1 8

C0039231 GO:0045767 1 8

C0039231 GO:0042301 1 8

C0039231 GO:0016538 1 8

C0039231 GO:0030166 1 8

C0039231 GO:0045604 1 8

C0039231 GO:0033189 1 8

C0039231 GO:0046716 1 8

C0039231 GO:0048531 1 8

C0039231 GO:0046634 1 8

C0039231 GO:0032526 1 8

C0039231 GO:0006700 1 8

C0039231 GO:0009247 1 8

C0039231 GO:0007585 1 8

C0039231 GO:0030021 1 8

C0039231 GO:0033483 1 8

C0039231 GO:0009112 1 8

C0039231 GO:0032856 1 8

C0039231 GO:0016903 1 8

C0039231 GO:0055067 1 8

C0039231 GO:0031214 2 8

C0039231 GO:0043627 1 8

C0039231 GO:0042552 1 8

C0039231 GO:0035295 1 8

C0039231 GO:0006752 1 8

C0039231 GO:0050750 1 8

C0039231 GO:0009746 1 8

C0039231 GO:0043190 1 8

C0039231 GO:0005977 1 8

C0039231 GO:0002793 1 8

C0039231 GO:0019840 1 8

C0039231 GO:0019212 1 8

C0039231 GO:0009749 1 8

C0039231 GO:0032321 1 8

C0039231 GO:0030902 1 8

C0039231 GO:0006885 1 8

C0039231 GO:0060538 1 8

C0039231 GO:0043112 1 8

C0039231 GO:0005161 1 8

C0039231 GO:0015036 1 8

C0043094 GO:0051043 2 8

C0043094 GO:0051044 1 8

C0043094 GO:0035065 1 8

C0043094 GO:0048638 1 8

C0043094 GO:0046637 1 8

C0043094 GO:0031970 1 8

C0043094 GO:0046634 1 8

C0043094 GO:0019228 1 8

C0043094 GO:0008585 1 8

C0043094 GO:0050926 1 8

C0043094 GO:0050927 1 8

C0043094 GO:0043524 1 8

C0043094 GO:0019915 1 8

C0043094 GO:0051893 1 8

C0043094 GO:0031058 1 8

C0043094 GO:0046631 1 8

C0043094 GO:0046883 1 8

C0043094 GO:0051897 1 8

C0043094 GO:0045981 1 8

C0043094 GO:0030228 1 8

C0043094 GO:0031985 1 8

C0043094 GO:0043205 1 8

C0043094 GO:0005540 1 8

C0043094 GO:0002263 1 8

C0043094 GO:0007519 2 8

C0043094 GO:0007217 1 8

C0043094 GO:0018210 1 8

C0043094 GO:0070001 1 8

C0043094 GO:0043325 1 8

C0043094 GO:0004712 1 8

C0043094 GO:0010896 1 8

C0043094 GO:0004864 1 8

C0043094 GO:0010898 1 8

C0043094 GO:0046504 1 8

C0043094 GO:0030858 1 8

C0043094 GO:0043120 2 8

C0043094 GO:0048145 1 8

C0043094 GO:0033032 1 8

C0043094 GO:0060541 1 8

C0043094 GO:0016202 1 8

C0043094 GO:0051055 1 8

C0043094 GO:0032655 1 8

C0043094 GO:0005790 1 8

C0043094 GO:0014020 1 8

C0043094 GO:0005159 1 8

C0043094 GO:0005158 1 8

C0043094 GO:0006769 1 8

C0043094 GO:0007281 1 8

C0043094 GO:0001841 1 8

C0043094 GO:0043535 1 8

C0043094 GO:0032373 2 8

C0043094 GO:0006477 1 8

C0043094 GO:0016894 1 8

C0043094 GO:0030041 1 8

C0043094 GO:0030291 1 8

C0043094 GO:0043536 1 8

C0043094 GO:0030295 1 8

C0043094 GO:0010874 1 8

C0043094 GO:0010875 2 8

C0043094 GO:0045580 2 8

C0043094 GO:0042326 1 8

C0043094 GO:0055092 1 8

C0043094 GO:0016812 1 8

C0043094 GO:0015145 1 8

C0043094 GO:0007162 1 8

C0043094 GO:0016814 1 8

C0043094 GO:0060393 1 8

C0043094 GO:0016566 1 8

C0043094 GO:0005903 1 8

C0043094 GO:0043627 1 8

C0043094 GO:0046849 1 8

C0043094 GO:0007043 1 8

C0043094 GO:0004708 1 8

C0043094 GO:0043034 1 8

C0043094 GO:0030194 1 8

C0043094 GO:0005089 1 8

C0043094 GO:0005088 1 8

C0043094 GO:0060263 1 8

C0043094 GO:0032410 1 8

C0043094 GO:0032412 1 8

C0043094 GO:0001843 1 8

C0043094 GO:0045739 1 8

C0043094 GO:0006458 1 8

C0043094 GO:0032526 1 8

C0043094 GO:0009898 1 8

C0043094 GO:0005086 1 8

C0043094 GO:0019934 1 8

C0043094 GO:0050820 1 8

C0043094 GO:0001763 1 8

C0043094 GO:0032946 1 8

C0043094 GO:0046496 1 8

C0043094 GO:0000578 1 8

C0043094 GO:0005929 1 8

C0043094 GO:0048002 1 8

C0043094 GO:0032496 1 8

C0043094 GO:0009068 1 8

C0043094 GO:0030178 1 8

C0043094 GO:0048488 1 8

C0043094 GO:0003205 1 8

C0043094 GO:0003206 1 8

C0043094 GO:0002286 1 8

C0043094 GO:0048660 1 8

C0043094 GO:0046915 1 8

C0043094 GO:0016045 1 8

C0043094 GO:0016863 1 8

C0043094 GO:0031228 1 8

C0043094 GO:0006942 1 8

C0043094 GO:0050700 2 8

C0043094 GO:0005858 1 8

C0043094 GO:0015149 1 8

C0043094 GO:0043028 1 8

C0043094 GO:0002366 1 8

C0043094 GO:0006112 1 8

C0043094 GO:0006687 1 8

C0043094 GO:0019218 1 8

C0043094 GO:0005522 1 8

C0043094 GO:0045912 2 8

C0043094 GO:0006688 1 8

C0043094 GO:0016860 1 8

C0043094 GO:0051879 1 8

C0043094 GO:0030879 2 8

C0043094 GO:0043027 1 8

C0043094 GO:0048705 1 8

C0043094 GO:0030149 1 8

C0043094 GO:0019882 1 8

C0043094 GO:0019883 1 8

C0043094 GO:0046545 1 8

C0043094 GO:0015833 1 8

C0043094 GO:0048709 1 8

C0043094 GO:0070167 1 8

C0043094 GO:0008154 1 8

C0043094 GO:0050810 2 8

C0043094 GO:0046870 1 8

C0043094 GO:0048255 1 8

C0043094 GO:0043256 1 8

C0043094 GO:0042632 1 8

C0043094 GO:0046660 1 8

C0043094 GO:0045616 1 8

C0043094 GO:0004437 1 8

C0043094 GO:0060606 1 8

C0043094 GO:0045165 1 8

C0043094 GO:0008091 1 8

C0043094 GO:0050885 1 8

C0043094 GO:0042734 1 8

C0043094 GO:0050714 1 8

C0043094 GO:0034384 1 8

C0043094 GO:0060416 1 8

C0043094 GO:0008206 1 8

C0043094 GO:0070567 1 8

C0043094 GO:0017080 1 8

C0043094 GO:0060260 1 8

C0043094 GO:0031346 1 8

C0043094 GO:0006752 1 8

C0043094 GO:0042834 1 8

C0043094 GO:0021537 1 8

C0043094 GO:0006637 1 8

C0043094 GO:0051702 1 8

C0043094 GO:0005788 1 8

C0043094 GO:0015695 1 8

C0043094 GO:0009311 1 8

C0043094 GO:0048634 1 8

C0043094 GO:0017022 1 8

C0043094 GO:0035148 1 8

C0043094 GO:0006941 1 8

C0043094 GO:0014003 1 8

C0043094 GO:0045429 1 8

C0043094 GO:0009895 1 8

C0043094 GO:0046677 1 8

C0043094 GO:0048029 1 8

C0043094 GO:0004693 1 8

C0043094 GO:0004691 1 8

C0043094 GO:0004690 1 8

C0043094 GO:0051457 1 8

C0043094 GO:0034599 1 8

C0043094 GO:0009124 1 8

C0043094 GO:0004716 1 8

C0043094 GO:0019362 1 8

C0043094 GO:0045078 1 8

C0043094 GO:0043489 1 8

C0043094 GO:0005583 1 8

C0043094 GO:0002237 1 8

C0043094 GO:0032376 2 8

C0043094 GO:0032370 2 8

C0043094 GO:0045072 1 8

C0043094 GO:0046887 1 8

C0043094 GO:0016459 1 8

C0043094 GO:0046782 1 8

C0043094 GO:0005520 1 8

C0043094 GO:0046466 1 8

C0043094 GO:0060562 1 8

C0043094 GO:0005506 1 8

C0043094 GO:0045622 1 8

C0043094 GO:0005501 1 8

C0043094 GO:0008366 1 8

C0043094 GO:0015166 1 8

C0043094 GO:0008286 1 8

C0043094 GO:0015296 1 8

C0043094 GO:0016877 1 8

C0043094 GO:0008329 1 8

C0043094 GO:0031330 1 8

C0043094 GO:0030317 1 8

C0043094 GO:0046326 2 8

C0043094 GO:0045211 1 8

C0043094 GO:0005796 1 8

C0043094 GO:0015807 2 8

C0043094 GO:0002440 1 8

C0043094 GO:0006929 1 8

C0043094 GO:0045216 1 8

C0043094 GO:0010677 2 8

C0043094 GO:0005593 1 8

C0043094 GO:0033293 1 8

C0043094 GO:0006413 1 8

C0043094 GO:0008093 1 8

C0043094 GO:0005507 1 8

C0043094 GO:0050679 1 8

C0043094 GO:0030804 1 8

C0043094 GO:0030801 1 8

C0043094 GO:0050671 1 8

C0043094 GO:0045940 1 8

C0043094 GO:0032862 1 8

C0043094 GO:0004190 1 8

C0043094 GO:0045619 1 8

C0043094 GO:0043370 1 8

C0043094 GO:0045861 1 8

C0043094 GO:0022614 1 8

C0043094 GO:0019840 1 8

C0043094 GO:0018107 1 8

C0043094 GO:0000185 1 8

C0043094 GO:0030323 1 8

C0043094 GO:0030324 1 8

C0043094 GO:0070228 1 8

C0043094 GO:0051923 1 8

C0043094 GO:0019209 1 8

C0043094 GO:0002285 1 8

C0043094 GO:0017048 1 8

C0043094 GO:0060021 2 8

C0043094 GO:0008210 1 8

C0043094 GO:0002822 1 8

C0043094 GO:0034623 1 8

C0043094 GO:0006081 1 8

C0043094 GO:0043506 2 8

C0043094 GO:0007272 1 8

C0043094 GO:0042169 1 8

C0043094 GO:0010828 2 8

C0043094 GO:0045768 1 8

C0043094 GO:0046890 1 8

C0043094 GO:0034765 1 8

C0043094 GO:0044447 1 8

C0043094 GO:0004181 1 8

C0043094 GO:0006937 1 8

C0043094 GO:0045766 1 8

C0043094 GO:0045767 1 8

C0043094 GO:0016538 1 8

C0043094 GO:0030810 1 8

C0043094 GO:0070665 1 8

C0043094 GO:0030166 1 8

C0043094 GO:0045604 1 8

C0043094 GO:0033189 1 8

C0043094 GO:0051353 1 8

C0043094 GO:0010741 1 8

C0043094 GO:0046716 1 8

C0043094 GO:0042552 1 8

C0043094 GO:0009247 1 8

C0043094 GO:0006700 1 8

C0043094 GO:0048662 1 8

C0043094 GO:0030902 1 8

C0043094 GO:0070120 1 8

C0043094 GO:0000217 1 8

C0043094 GO:0007585 1 8

C0043094 GO:0009593 1 8

C0043094 GO:0032856 1 8

C0043094 GO:0016903 1 8

C0043094 GO:0055067 1 8

C0043094 GO:0031214 1 8

C0043094 GO:0051646 1 8

C0043094 GO:0007266 1 8

C0043094 GO:0035295 1 8

C0043094 GO:0001530 1 8

C0043094 GO:0050709 1 8

C0043094 GO:0001838 1 8

C0043094 GO:0043190 1 8

C0043094 GO:0016620 1 8

C0043094 GO:0010595 1 8

C0043094 GO:0010594 1 8

C0043094 GO:0042345 1 8

C0043094 GO:0004861 1 8

C0043094 GO:0045123 1 8

C0043094 GO:0016338 2 8

C0043094 GO:0050995 1 8

C0043094 GO:0050994 1 8

C0043094 GO:0019212 1 8

C0043094 GO:0015665 1 8

C0043094 GO:0032321 1 8

C0043094 GO:0050750 1 8

C0043094 GO:0006885 1 8

C0043094 GO:0060538 2 8

C0043094 GO:0001654 1 8

C0043094 GO:0043112 1 8

C0043094 GO:0005161 1 8

C0043094 GO:0001937 1 8

C0043094 GO:0015036 1 8

C0043096 GO:0051043 1 7

C0043096 GO:0051044 1 7

C0043096 GO:0035065 1 7

C0043096 GO:0050840 1 7

C0043096 GO:0046637 1 7

C0043096 GO:0031970 1 7

C0043096 GO:0046634 1 7

C0043096 GO:0007612 1 7

C0043096 GO:0021510 2 7

C0043096 GO:0008585 1 7

C0043096 GO:0007162 1 7

C0043096 GO:0050927 1 7

C0043096 GO:0050920 1 7

C0043096 GO:0050921 1 7

C0043096 GO:0019915 1 7

C0043096 GO:0051893 1 7

C0043096 GO:0031058 1 7

C0043096 GO:0031343 1 7

C0043096 GO:0002573 1 7

C0043096 GO:0016597 1 7

C0043096 GO:0005149 2 7

C0043096 GO:0006306 1 7

C0043096 GO:0006305 1 7

C0043096 GO:0043205 1 7

C0043096 GO:0030195 1 7

C0043096 GO:0046631 1 7

C0043096 GO:0002263 1 7

C0043096 GO:0007519 2 7

C0043096 GO:0045616 1 7

C0043096 GO:0070001 3 7

C0043096 GO:0046850 1 7

C0043096 GO:0045661 1 7

C0043096 GO:0010896 2 7

C0043096 GO:0010921 1 7

C0043096 GO:0010898 2 7

C0043096 GO:0046504 1 7

C0043096 GO:0032722 1 7

C0043096 GO:0033032 1 7

C0043096 GO:0060541 1 7

C0043096 GO:0016202 1 7

C0043096 GO:0032655 2 7

C0043096 GO:0014020 1 7

C0043096 GO:0032781 1 7

C0043096 GO:0005159 1 7

C0043096 GO:0005158 1 7

C0043096 GO:0002821 1 7

C0043096 GO:0030166 1 7

C0043096 GO:0035270 1 7

C0043096 GO:0046330 1 7

C0043096 GO:0032373 2 7

C0043096 GO:0006477 2 7

C0043096 GO:0030041 1 7

C0043096 GO:0030291 1 7

C0043096 GO:0043536 2 7

C0043096 GO:0030295 1 7

C0043096 GO:0010874 2 7

C0043096 GO:0010875 2 7

C0043096 GO:0019827 1 7

C0043096 GO:0055092 1 7

C0043096 GO:0034284 1 7

C0043096 GO:0016812 1 7

C0043096 GO:0043025 1 7

C0043096 GO:0006044 1 7

C0043096 GO:0005902 1 7

C0043096 GO:0016814 1 7

C0043096 GO:0051353 3 7

C0043096 GO:0002377 2 7

C0043096 GO:0032350 1 7

C0043096 GO:0005903 1 7

C0043096 GO:0043627 1 7

C0043096 GO:0030048 1 7

C0043096 GO:0007043 1 7

C0043096 GO:0043030 1 7

C0043096 GO:0043034 2 7

C0043096 GO:0030194 1 7

C0043096 GO:0005089 1 7

C0043096 GO:0005088 1 7

C0043096 GO:0007632 1 7

C0043096 GO:0060263 1 7

C0043096 GO:0045767 1 7

C0043096 GO:0004035 1 7

C0043096 GO:0032642 1 7

C0043096 GO:0032648 1 7

C0043096 GO:0009898 1 7

C0043096 GO:0005086 1 7

C0043096 GO:0019934 1 7

C0043096 GO:0050820 1 7

C0043096 GO:0034103 1 7

C0043096 GO:0048634 1 7

C0043096 GO:0000578 1 7

C0043096 GO:0005929 2 7

C0043096 GO:0048002 1 7

C0043096 GO:0048662 1 7

C0043096 GO:0010862 1 7

C0043096 GO:0051224 1 7

C0043096 GO:0016863 1 7

C0043096 GO:0001838 1 7

C0043096 GO:0002286 1 7

C0043096 GO:0051180 1 7

C0043096 GO:0046915 1 7

C0043096 GO:0030228 1 7

C0043096 GO:0043548 1 7

C0043096 GO:0003705 1 7

C0043096 GO:0005858 1 7

C0043096 GO:0002687 1 7

C0043096 GO:0045429 1 7

C0043096 GO:0002685 1 7

C0043096 GO:0006112 1 7

C0043096 GO:0006110 1 7

C0043096 GO:0005522 1 7

C0043096 GO:0045912 2 7

C0043096 GO:0006688 1 7

C0043096 GO:0016860 1 7

C0043096 GO:0051879 1 7

C0043096 GO:0008088 1 7

C0043096 GO:0048705 1 7

C0043096 GO:0019882 1 7

C0043096 GO:0048593 1 7

C0043096 GO:0046545 1 7

C0043096 GO:0020037 1 7

C0043096 GO:0055029 1 7

C0043096 GO:0032768 1 7

C0043096 GO:0035250 1 7

C0043096 GO:0019319 1 7

C0043096 GO:0050795 1 7

C0043096 GO:0050796 1 7

C0043096 GO:0008154 1 7

C0043096 GO:0050810 1 7

C0043096 GO:0048255 1 7

C0043096 GO:0048864 1 7

C0043096 GO:0046660 1 7

C0043096 GO:0050819 1 7

C0043096 GO:0042301 1 7

C0043096 GO:0004437 1 7

C0043096 GO:0060606 1 7

C0043096 GO:0045165 1 7

C0043096 GO:0009953 1 7

C0043096 GO:0042734 1 7

C0043096 GO:0050714 1 7

C0043096 GO:0002758 1 7

C0043096 GO:0030864 1 7

C0043096 GO:0030890 1 7

C0043096 GO:0070567 1 7

C0043096 GO:0045730 1 7

C0043096 GO:0015179 1 7

C0043096 GO:0004861 1 7

C0043096 GO:0003746 1 7

C0043096 GO:0043010 1 7

C0043096 GO:0042834 1 7

C0043096 GO:0015172 1 7

C0043096 GO:0003785 1 7

C0043096 GO:0021537 1 7

C0043096 GO:0005313 1 7

C0043096 GO:0030880 1 7

C0043096 GO:0019956 1 7

C0043096 GO:0005544 1 7

C0043096 GO:0005788 1 7

C0043096 GO:0032770 1 7

C0043096 GO:0019200 1 7

C0043096 GO:0048638 1 7

C0043096 GO:0035148 1 7

C0043096 GO:0006941 1 7

C0043096 GO:0002440 2 7

C0043096 GO:0031228 1 7

C0043096 GO:0009895 2 7

C0043096 GO:0046677 1 7

C0043096 GO:0048029 1 7

C0043096 GO:0042632 1 7

C0043096 GO:0004693 1 7

C0043096 GO:0051457 1 7

C0043096 GO:0035295 1 7

C0043096 GO:0009124 1 7

C0043096 GO:0043168 1 7

C0043096 GO:0050764 1 7

C0043096 GO:0045078 1 7

C0043096 GO:0030201 1 7

C0043096 GO:0043489 1 7

C0043096 GO:0032374 1 7

C0043096 GO:0032376 2 7

C0043096 GO:0032371 1 7

C0043096 GO:0032370 2 7

C0043096 GO:0045072 1 7

C0043096 GO:0030705 1 7

C0043096 GO:0001843 1 7

C0043096 GO:0016459 1 7

C0043096 GO:0002366 1 7

C0043096 GO:0046782 1 7

C0043096 GO:0002224 1 7

C0043096 GO:0060562 1 7

C0043096 GO:0005506 1 7

C0043096 GO:0045622 1 7

C0043096 GO:0002221 1 7

C0043096 GO:0022829 1 7

C0043096 GO:0044275 1 7

C0043096 GO:0015296 1 7

C0043096 GO:0043535 2 7

C0043096 GO:0008329 1 7

C0043096 GO:0031330 1 7

C0043096 GO:0000217 1 7

C0043096 GO:0045580 1 7

C0043096 GO:0043270 1 7

C0043096 GO:0045211 1 7

C0043096 GO:0015807 2 7

C0043096 GO:0001654 1 7

C0043096 GO:0006929 1 7

C0043096 GO:0045216 1 7

C0043096 GO:0010677 2 7

C0043096 GO:0005593 1 7

C0043096 GO:0008091 1 7

C0043096 GO:0001894 1 7

C0043096 GO:0005161 1 7

C0043096 GO:0006413 1 7

C0043096 GO:0010594 3 7

C0043096 GO:0051092 1 7

C0043096 GO:0040017 1 7

C0043096 GO:0043120 1 7

C0043096 GO:0000428 1 7

C0043096 GO:0019883 1 7

C0043096 GO:0004190 3 7

C0043096 GO:0045619 1 7

C0043096 GO:0032479 1 7

C0043096 GO:0034405 1 7

C0043096 GO:0043370 1 7

C0043096 GO:0045861 1 7

C0043096 GO:0016331 1 7

C0043096 GO:0030323 2 7

C0043096 GO:0030324 1 7

C0043096 GO:0042119 1 7

C0043096 GO:0046364 1 7

C0043096 GO:0005160 1 7

C0043096 GO:0019209 1 7

C0043096 GO:0002285 1 7

C0043096 GO:0017048 1 7

C0043096 GO:0060021 1 7

C0043096 GO:0043506 1 7

C0043096 GO:0050926 1 7

C0043096 GO:0032862 1 7

C0043096 GO:0016338 2 7

C0043096 GO:0070228 1 7

C0043096 GO:0007270 1 7

C0043096 GO:0006879 1 7

C0043096 GO:0030274 1 7

C0043096 GO:0009749 1 7

C0043096 GO:0010828 1 7

C0043096 GO:0045684 1 7

C0043096 GO:0045768 1 7

C0043096 GO:0001841 1 7

C0043096 GO:0002218 1 7

C0043096 GO:0042354 1 7

C0043096 GO:0004181 1 7

C0043096 GO:0042516 1 7

C0043096 GO:0045766 1 7

C0043096 GO:0006096 1 7

C0043096 GO:0016538 1 7

C0043096 GO:0032321 1 7

C0043096 GO:0015695 1 7

C0043096 GO:0045604 1 7

C0043096 GO:0051923 2 7

C0043096 GO:0070668 1 7

C0043096 GO:0031307 1 7

C0043096 GO:0010741 1 7

C0043096 GO:0051897 1 7

C0043096 GO:0009247 1 7

C0043096 GO:0006700 1 7

C0043096 GO:0021915 1 7

C0043096 GO:0048660 1 7

C0043096 GO:0048741 1 7

C0043096 GO:0046326 1 7

C0043096 GO:0034612 1 7

C0043096 GO:0007585 1 7

C0043096 GO:0042100 1 7

C0043096 GO:0032856 1 7

C0043096 GO:0016903 1 7

C0043096 GO:0046580 1 7

C0043096 GO:0031214 1 7

C0043096 GO:0051646 1 7

C0043096 GO:0007266 1 7

C0043096 GO:0034599 1 7

C0043096 GO:0051058 1 7

C0043096 GO:0001530 1 7

C0043096 GO:0009746 1 7

C0043096 GO:0043190 1 7

C0043096 GO:0000178 1 7

C0043096 GO:0010595 2 7

C0043096 GO:0051881 1 7

C0043096 GO:0042345 1 7

C0043096 GO:0051928 1 7

C0043096 GO:0005044 1 7

C0043096 GO:0044447 1 7

C0043096 GO:0050995 1 7

C0043096 GO:0050994 1 7

C0043096 GO:0045124 1 7

C0043096 GO:0031526 1 7

C0043096 GO:0046928 1 7

C0043096 GO:0002250 1 7

C0043096 GO:0060538 2 7

C0043096 GO:0016505 1 7

C0043096 GO:0008290 1 7

C0043096 GO:0006940 1 7

C0043096 GO:0048520 1 7

C0043096 GO:0001937 1 7

C0043096 GO:0015036 1 7

C0235198 GO:0035065 1 3

C0235198 GO:0046637 1 3

C0235198 GO:0046634 1 3

C0235198 GO:0007612 1 3

C0235198 GO:0021510 1 3

C0235198 GO:0007162 1 3

C0235198 GO:0045767 1 3

C0235198 GO:0016597 1 3

C0235198 GO:0005149 1 3

C0235198 GO:0006306 1 3

C0235198 GO:0006305 1 3

C0235198 GO:0048864 1 3

C0235198 GO:0007519 1 3

C0235198 GO:0070001 1 3

C0235198 GO:0016459 1 3

C0235198 GO:0045661 1 3

C0235198 GO:0033032 1 3

C0235198 GO:0060541 1 3

C0235198 GO:0016202 1 3

C0235198 GO:0032655 1 3

C0235198 GO:0014020 1 3

C0235198 GO:0001841 1 3

C0235198 GO:0048634 1 3

C0235198 GO:0030048 1 3

C0235198 GO:0043535 1 3

C0235198 GO:0030041 1 3

C0235198 GO:0030291 1 3

C0235198 GO:0030295 1 3

C0235198 GO:0010874 1 3

C0235198 GO:0010875 1 3

C0235198 GO:0019827 1 3

C0235198 GO:0055092 1 3

C0235198 GO:0016814 1 3

C0235198 GO:0051353 1 3

C0235198 GO:0002377 1 3

C0235198 GO:0043034 2 3

C0235198 GO:0005089 1 3

C0235198 GO:0005088 1 3

C0235198 GO:0007632 1 3

C0235198 GO:0060263 1 3

C0235198 GO:0001843 1 3

C0235198 GO:0009898 1 3

C0235198 GO:0005086 1 3

C0235198 GO:0034103 1 3

C0235198 GO:0000578 1 3

C0235198 GO:0051224 1 3

C0235198 GO:0020037 1 3

C0235198 GO:0043548 1 3

C0235198 GO:0008088 1 3

C0235198 GO:0046850 1 3

C0235198 GO:0006112 1 3

C0235198 GO:0006110 1 3

C0235198 GO:0005522 1 3

C0235198 GO:0006688 1 3

C0235198 GO:0043025 1 3

C0235198 GO:0048705 1 3

C0235198 GO:0030890 1 3

C0235198 GO:0032768 1 3

C0235198 GO:0050796 1 3

C0235198 GO:0008154 1 3

C0235198 GO:0042632 1 3

C0235198 GO:0050819 1 3

C0235198 GO:0044275 1 3

C0235198 GO:0060606 1 3

C0235198 GO:0008091 1 3

C0235198 GO:0042734 1 3

C0235198 GO:0030864 1 3

C0235198 GO:0045730 1 3

C0235198 GO:0015179 1 3

C0235198 GO:0043010 1 3

C0235198 GO:0032770 1 3

C0235198 GO:0019956 1 3

C0235198 GO:0010921 1 3

C0235198 GO:0019200 1 3

C0235198 GO:0035148 1 3

C0235198 GO:0006941 1 3

C0235198 GO:0009895 1 3

C0235198 GO:0007043 1 3

C0235198 GO:0048029 1 3

C0235198 GO:0032374 1 3

C0235198 GO:0032376 1 3

C0235198 GO:0032371 1 3

C0235198 GO:0032370 1 3

C0235198 GO:0032373 1 3

C0235198 GO:0030705 1 3

C0235198 GO:0060562 1 3

C0235198 GO:0045622 1 3

C0235198 GO:0015296 1 3

C0235198 GO:0006879 1 3

C0235198 GO:0009247 1 3

C0235198 GO:0045580 1 3

C0235198 GO:0004861 1 3

C0235198 GO:0015807 1 3

C0235198 GO:0006929 1 3

C0235198 GO:0045216 1 3

C0235198 GO:0009953 1 3

C0235198 GO:0001894 1 3

C0235198 GO:0004190 1 3

C0235198 GO:0045619 1 3

C0235198 GO:0034405 1 3

C0235198 GO:0043370 1 3

C0235198 GO:0045616 1 3

C0235198 GO:0016331 1 3

C0235198 GO:0030323 2 3

C0235198 GO:0030324 1 3

C0235198 GO:0005160 1 3

C0235198 GO:0019209 1 3

C0235198 GO:0017048 1 3

C0235198 GO:0060021 1 3

C0235198 GO:0043506 1 3

C0235198 GO:0032862 1 3

C0235198 GO:0007270 1 3

C0235198 GO:0006096 1 3

C0235198 GO:0045684 1 3

C0235198 GO:0045768 1 3

C0235198 GO:0032350 1 3

C0235198 GO:0042354 1 3

C0235198 GO:0004181 1 3

C0235198 GO:0045766 1 3

C0235198 GO:0002573 1 3

C0235198 GO:0016538 1 3

C0235198 GO:0045604 1 3

C0235198 GO:0070668 1 3

C0235198 GO:0030195 1 3

C0235198 GO:0006700 1 3

C0235198 GO:0021915 1 3

C0235198 GO:0048593 1 3

C0235198 GO:0032856 1 3

C0235198 GO:0003785 1 3

C0235198 GO:0031214 1 3

C0235198 GO:0043627 1 3

C0235198 GO:0035295 1 3

C0235198 GO:0001838 1 3

C0235198 GO:0043190 1 3

C0235198 GO:0010594 1 3

C0235198 GO:0042345 1 3

C0235198 GO:0005044 1 3

C0235198 GO:0016338 1 3

C0235198 GO:0003705 1 3

C0235198 GO:0045124 1 3

C0235198 GO:0032321 1 3

C0235198 GO:0060538 1 3

C0235198 GO:0005161 1 3

C0235198 GO:0015036 1 3

C0040264 GO:0051043 1 6

C0040264 GO:0035065 1 6

C0040264 GO:0032107 1 6

C0040264 GO:0046637 1 6

C0040264 GO:0031970 1 6

C0040264 GO:0032387 1 6

C0040264 GO:0021510 1 6

C0040264 GO:0008585 1 6

C0040264 GO:0002703 1 6

C0040264 GO:0043525 1 6

C0040264 GO:0019915 1 6

C0040264 GO:0051893 1 6

C0040264 GO:0031058 1 6

C0040264 GO:0031343 1 6

C0040264 GO:0043205 1 6

C0040264 GO:0043206 1 6

C0040264 GO:0007519 2 6

C0040264 GO:0070001 1 6

C0040264 GO:0050715 1 6

C0040264 GO:0016459 1 6

C0040264 GO:0004712 1 6

C0040264 GO:0010896 1 6

C0040264 GO:0010721 1 6

C0040264 GO:0010898 1 6

C0040264 GO:0046504 1 6

C0040264 GO:0033032 1 6

C0040264 GO:0060541 1 6

C0040264 GO:0016202 1 6

C0040264 GO:0032655 1 6

C0040264 GO:0014020 1 6

C0040264 GO:0031960 1 6

C0040264 GO:0005159 1 6

C0040264 GO:0005158 1 6

C0040264 GO:0002822 1 6

C0040264 GO:0001841 1 6

C0040264 GO:0030041 1 6

C0040264 GO:0030291 1 6

C0040264 GO:0042992 1 6

C0040264 GO:0030295 1 6

C0040264 GO:0045580 1 6

C0040264 GO:0016055 1 6

C0040264 GO:0016814 1 6

C0040264 GO:0001910 1 6

C0040264 GO:0043627 1 6

C0040264 GO:0046849 1 6

C0040264 GO:0007043 1 6

C0040264 GO:0004708 1 6

C0040264 GO:0043034 1 6

C0040264 GO:0030194 1 6

C0040264 GO:0005089 1 6

C0040264 GO:0005088 1 6

C0040264 GO:0060263 1 6

C0040264 GO:0001843 1 6

C0040264 GO:0009898 1 6

C0040264 GO:0005086 1 6

C0040264 GO:0019934 1 6

C0040264 GO:0050820 1 6

C0040264 GO:0032946 1 6

C0040264 GO:0000578 1 6

C0040264 GO:0009068 1 6

C0040264 GO:0021700 1 6

C0040264 GO:0007530 1 6

C0040264 GO:0003205 1 6

C0040264 GO:0003206 1 6

C0040264 GO:0043547 1 6

C0040264 GO:0046915 1 6

C0040264 GO:0019047 1 6

C0040264 GO:0006112 1 6

C0040264 GO:0006687 1 6

C0040264 GO:0005522 1 6

C0040264 GO:0048641 1 6

C0040264 GO:0006688 1 6

C0040264 GO:0051879 1 6

C0040264 GO:0048705 1 6

C0040264 GO:0048742 1 6

C0040264 GO:0046545 1 6

C0040264 GO:0009880 1 6

C0040264 GO:0048709 1 6

C0040264 GO:0005138 2 6

C0040264 GO:0008154 1 6

C0040264 GO:0048255 1 6

C0040264 GO:0042308 1 6

C0040264 GO:0043256 1 6

C0040264 GO:0042632 1 6

C0040264 GO:0030069 1 6

C0040264 GO:0046660 1 6

C0040264 GO:0045616 1 6

C0040264 GO:0042301 1 6

C0040264 GO:0004437 1 6

C0040264 GO:0007423 1 6

C0040264 GO:0007250 1 6

C0040264 GO:0009895 1 6

C0040264 GO:0050885 1 6

C0040264 GO:0042734 1 6

C0040264 GO:0050714 1 6

C0040264 GO:0031645 1 6

C0040264 GO:0015074 1 6

C0040264 GO:0046823 1 6

C0040264 GO:0051384 1 6

C0040264 GO:0021537 2 6

C0040264 GO:0032104 1 6

C0040264 GO:0050768 1 6

C0040264 GO:0048634 1 6

C0040264 GO:0035148 1 6

C0040264 GO:0006941 1 6

C0040264 GO:0014003 1 6

C0040264 GO:0005903 1 6

C0040264 GO:0046677 1 6

C0040264 GO:0048029 1 6

C0040264 GO:0004693 1 6

C0040264 GO:0043087 1 6

C0040264 GO:0045078 1 6

C0040264 GO:0043489 1 6

C0040264 GO:0045072 1 6

C0040264 GO:0046782 1 6

C0040264 GO:0060562 1 6

C0040264 GO:0045622 1 6

C0040264 GO:0042169 1 6

C0040264 GO:0015296 2 6

C0040264 GO:0031330 1 6

C0040264 GO:0015459 1 6

C0040264 GO:0000217 1 6

C0040264 GO:0045211 1 6

C0040264 GO:0015807 1 6

C0040264 GO:0002440 1 6

C0040264 GO:0006929 1 6

C0040264 GO:0001656 1 6

C0040264 GO:0031397 1 6

C0040264 GO:0043197 1 6

C0040264 GO:0042269 1 6

C0040264 GO:0050679 1 6

C0040264 GO:0002460 1 6

C0040264 GO:0046906 1 6

C0040264 GO:0018105 1 6

C0040264 GO:0050671 1 6

C0040264 GO:0032862 1 6

C0040264 GO:0004190 1 6

C0040264 GO:0045619 1 6

C0040264 GO:0043370 1 6

C0040264 GO:0045861 1 6

C0040264 GO:0030323 1 6

C0040264 GO:0030324 1 6

C0040264 GO:0019209 1 6

C0040264 GO:0008286 1 6

C0040264 GO:0017048 2 6

C0040264 GO:0060021 1 6

C0040264 GO:0008210 1 6

C0040264 GO:0043506 2 6

C0040264 GO:0045682 1 6

C0040264 GO:0055092 1 6

C0040264 GO:0045768 1 6

C0040264 GO:0045216 1 6

C0040264 GO:0060606 1 6

C0040264 GO:0004181 1 6

C0040264 GO:0045766 1 6

C0040264 GO:0045767 1 6

C0040264 GO:0016538 1 6

C0040264 GO:0070665 1 6

C0040264 GO:0045604 1 6

C0040264 GO:0010741 1 6

C0040264 GO:0043588 1 6

C0040264 GO:0046634 1 6

C0040264 GO:0006700 1 6

C0040264 GO:0009247 1 6

C0040264 GO:0048592 1 6

C0040264 GO:0048593 1 6

C0040264 GO:0032856 1 6

C0040264 GO:0031575 1 6

C0040264 GO:0002715 1 6

C0040264 GO:0031214 2 6

C0040264 GO:0051646 1 6

C0040264 GO:0007266 1 6

C0040264 GO:0035295 1 6

C0040264 GO:0001838 1 6

C0040264 GO:0043190 1 6

C0040264 GO:0032318 1 6

C0040264 GO:0042345 1 6

C0040264 GO:0004861 1 6

C0040264 GO:0043010 1 6

C0040264 GO:0016338 1 6

C0040264 GO:0050995 1 6

C0040264 GO:0050994 1 6

C0040264 GO:0032320 1 6

C0040264 GO:0032321 1 6

C0040264 GO:0030900 1 6

C0040264 GO:0002250 1 6

C0040264 GO:0060538 2 6

C0040264 GO:0001654 1 6

C0040264 GO:0005161 1 6

C0040264 GO:0001937 1 6

C0040264 GO:0015036 1 6

C0002622 GO:0051043 1 6

C0002622 GO:0051044 1 6

C0002622 GO:0035065 1 6

C0002622 GO:0048638 1 6

C0002622 GO:0046637 1 6

C0002622 GO:0031970 1 6

C0002622 GO:0032387 1 6

C0002622 GO:0021510 1 6

C0002622 GO:0008585 1 6

C0002622 GO:0050926 1 6

C0002622 GO:0050927 1 6

C0002622 GO:0043525 1 6

C0002622 GO:0019915 1 6

C0002622 GO:0051893 1 6

C0002622 GO:0031058 1 6

C0002622 GO:0046631 1 6

C0002622 GO:0051897 1 6

C0002622 GO:0030228 1 6

C0002622 GO:0043205 1 6

C0002622 GO:0043206 1 6

C0002622 GO:0002263 1 6

C0002622 GO:0007519 2 6

C0002622 GO:0070001 1 6

C0002622 GO:0016459 1 6

C0002622 GO:0004712 1 6

C0002622 GO:0010896 1 6

C0002622 GO:0010721 1 6

C0002622 GO:0010898 1 6

C0002622 GO:0046504 1 6

C0002622 GO:0043120 1 6

C0002622 GO:0033032 1 6

C0002622 GO:0060541 1 6

C0002622 GO:0016202 1 6

C0002622 GO:0032655 1 6

C0002622 GO:0014020 1 6

C0002622 GO:0031960 1 6

C0002622 GO:0005159 1 6

C0002622 GO:0005158 1 6

C0002622 GO:0001841 1 6

C0002622 GO:0043535 1 6

C0002622 GO:0032373 1 6

C0002622 GO:0006477 1 6

C0002622 GO:0030041 1 6

C0002622 GO:0030291 1 6

C0002622 GO:0042992 1 6

C0002622 GO:0030295 1 6

C0002622 GO:0010874 1 6

C0002622 GO:0010875 1 6

C0002622 GO:0045580 1 6

C0002622 GO:0055092 1 6

C0002622 GO:0016812 1 6

C0002622 GO:0016055 1 6

C0002622 GO:0016814 1 6

C0002622 GO:0051353 1 6

C0002622 GO:0005903 1 6

C0002622 GO:0043627 1 6

C0002622 GO:0007043 1 6

C0002622 GO:0004708 1 6

C0002622 GO:0043034 1 6

C0002622 GO:0030194 1 6

C0002622 GO:0005089 1 6

C0002622 GO:0005088 1 6

C0002622 GO:0060263 1 6

C0002622 GO:0001843 1 6

C0002622 GO:0009898 1 6

C0002622 GO:0005086 1 6

C0002622 GO:0019934 1 6

C0002622 GO:0050820 1 6

C0002622 GO:0000578 1 6

C0002622 GO:0005929 1 6

C0002622 GO:0048002 1 6

C0002622 GO:0016863 1 6

C0002622 GO:0031575 1 6

C0002622 GO:0002286 1 6

C0002622 GO:0043547 1 6

C0002622 GO:0046915 1 6

C0002622 GO:0043087 1 6

C0002622 GO:0002366 1 6

C0002622 GO:0006112 1 6

C0002622 GO:0005522 1 6

C0002622 GO:0045912 2 6

C0002622 GO:0006688 1 6

C0002622 GO:0016860 1 6

C0002622 GO:0051879 1 6

C0002622 GO:0048705 1 6

C0002622 GO:0019882 1 6

C0002622 GO:0019883 1 6

C0002622 GO:0046545 1 6

C0002622 GO:0009880 1 6

C0002622 GO:0005138 1 6

C0002622 GO:0008154 1 6

C0002622 GO:0050810 1 6

C0002622 GO:0048255 1 6

C0002622 GO:0042308 1 6

C0002622 GO:0042632 1 6

C0002622 GO:0046660 1 6

C0002622 GO:0045616 1 6

C0002622 GO:0004437 1 6

C0002622 GO:0060606 1 6

C0002622 GO:0045165 1 6

C0002622 GO:0042734 1 6

C0002622 GO:0050714 1 6

C0002622 GO:0070567 1 6

C0002622 GO:0046823 1 6

C0002622 GO:0051384 1 6

C0002622 GO:0042834 1 6

C0002622 GO:0032107 1 6

C0002622 GO:0032104 1 6

C0002622 GO:0050768 1 6

C0002622 GO:0005788 1 6

C0002622 GO:0048634 1 6

C0002622 GO:0035148 1 6

C0002622 GO:0006941 1 6

C0002622 GO:0002440 1 6

C0002622 GO:0045429 1 6

C0002622 GO:0009895 1 6

C0002622 GO:0046677 1 6

C0002622 GO:0048029 1 6

C0002622 GO:0004693 1 6

C0002622 GO:0051457 1 6

C0002622 GO:0009124 1 6

C0002622 GO:0045078 1 6

C0002622 GO:0043489 1 6

C0002622 GO:0032376 1 6

C0002622 GO:0032370 1 6

C0002622 GO:0045072 1 6

C0002622 GO:0046782 1 6

C0002622 GO:0060562 1 6

C0002622 GO:0005506 1 6

C0002622 GO:0045622 1 6

C0002622 GO:0042169 1 6

C0002622 GO:0015296 2 6

C0002622 GO:0008329 1 6

C0002622 GO:0031330 1 6

C0002622 GO:0021537 2 6

C0002622 GO:0000217 1 6

C0002622 GO:0045211 1 6

C0002622 GO:0015807 1 6

C0002622 GO:0001654 2 6

C0002622 GO:0006929 1 6

C0002622 GO:0001656 1 6

C0002622 GO:0010677 2 6

C0002622 GO:0005593 1 6

C0002622 GO:0043197 1 6

C0002622 GO:0006413 1 6

C0002622 GO:0043536 1 6

C0002622 GO:0007423 1 6

C0002622 GO:0002460 1 6

C0002622 GO:0046906 1 6

C0002622 GO:0018105 1 6

C0002622 GO:0004190 1 6

C0002622 GO:0045619 1 6

C0002622 GO:0043370 1 6

C0002622 GO:0045861 1 6

C0002622 GO:0030323 1 6

C0002622 GO:0030324 1 6

C0002622 GO:0070228 1 6

C0002622 GO:0051923 1 6

C0002622 GO:0019209 1 6

C0002622 GO:0008286 1 6

C0002622 GO:0017048 1 6

C0002622 GO:0060021 1 6

C0002622 GO:0032862 1 6

C0002622 GO:0043506 2 6

C0002622 GO:0015459 1 6

C0002622 GO:0010828 1 6

C0002622 GO:0045768 1 6

C0002622 GO:0045216 1 6

C0002622 GO:0004181 1 6

C0002622 GO:0045766 1 6

C0002622 GO:0045767 1 6

C0002622 GO:0016538 1 6

C0002622 GO:0015695 1 6

C0002622 GO:0045604 1 6

C0002622 GO:0010741 1 6

C0002622 GO:0043588 1 6

C0002622 GO:0046634 1 6

C0002622 GO:0009247 1 6

C0002622 GO:0006700 1 6

C0002622 GO:0048662 1 6

C0002622 GO:0048660 1 6

C0002622 GO:0046326 1 6

C0002622 GO:0048592 1 6

C0002622 GO:0048593 1 6

C0002622 GO:0002285 1 6

C0002622 GO:0032856 1 6

C0002622 GO:0032318 1 6

C0002622 GO:0031214 1 6

C0002622 GO:0051646 1 6

C0002622 GO:0007266 1 6

C0002622 GO:0035295 1 6

C0002622 GO:0001530 1 6

C0002622 GO:0001838 1 6

C0002622 GO:0043190 1 6

C0002622 GO:0010595 1 6

C0002622 GO:0010594 1 6

C0002622 GO:0042345 1 6

C0002622 GO:0004861 1 6

C0002622 GO:0043010 1 6

C0002622 GO:0016338 2 6

C0002622 GO:0050995 1 6

C0002622 GO:0050994 1 6

C0002622 GO:0032320 1 6

C0002622 GO:0032321 1 6

C0002622 GO:0030900 1 6

C0002622 GO:0002250 1 6

C0002622 GO:0060538 2 6

C0002622 GO:0005161 1 6

C0002622 GO:0001937 1 6

C0002622 GO:0015036 1 6

C0013144 GO:0005786 1 17

C0013144 GO:0051043 3 17

C0013144 GO:0051044 1 17

C0013144 GO:0030228 1 17

C0013144 GO:0043954 3 17

C0013144 GO:0010677 2 17

C0013144 GO:0009164 1 17

C0013144 GO:0001503 1 17

C0013144 GO:0002703 1 17

C0013144 GO:0034199 1 17

C0013144 GO:0016863 3 17

C0013144 GO:0016860 1 17

C0013144 GO:0043205 1 17

C0013144 GO:0004716 1 17

C0013144 GO:0033238 1 17

C0013144 GO:0030856 1 17

C0013144 GO:0046504 1 17

C0013144 GO:0030858 1 17

C0013144 GO:0060541 1 17

C0013144 GO:0006284 1 17

C0013144 GO:0002824 1 17

C0013144 GO:0002822 4 17

C0013144 GO:0007281 2 17

C0013144 GO:0006477 1 17

C0013144 GO:0007286 1 17

C0013144 GO:0030295 2 17

C0013144 GO:0030296 1 17

C0013144 GO:0042326 1 17

C0013144 GO:0034284 1 17

C0013144 GO:0016812 1 17

C0013144 GO:0070633 1 17

C0013144 GO:0016814 1 17

C0013144 GO:0045821 1 17

C0013144 GO:0004622 2 17

C0013144 GO:0060260 1 17

C0013144 GO:0019320 1 17

C0013144 GO:0009895 1 17

C0013144 GO:0032410 1 17

C0013144 GO:0032412 1 17

C0013144 GO:0050670 1 17

C0013144 GO:0006458 2 17

C0013144 GO:0009898 1 17

C0013144 GO:0000302 1 17

C0013144 GO:0000578 2 17

C0013144 GO:0030178 2 17

C0013144 GO:0042552 1 17

C0013144 GO:0006885 2 17

C0013144 GO:0008643 1 17

C0013144 GO:0043028 1 17

C0013144 GO:0006112 1 17

C0013144 GO:0045912 2 17

C0013144 GO:0051879 1 17

C0013144 GO:0002688 1 17

C0013144 GO:0048705 1 17

C0013144 GO:0019882 1 17

C0013144 GO:0019883 1 17

C0013144 GO:0005138 2 17

C0013144 GO:0004437 1 17

C0013144 GO:0050716 1 17

C0013144 GO:0050715 2 17

C0013144 GO:0050714 1 17

C0013144 GO:0050718 1 17

C0013144 GO:0005436 1 17

C0013144 GO:0070567 1 17

C0013144 GO:0006693 1 17

C0013144 GO:0006692 1 17

C0013144 GO:0006750 1 17

C0013144 GO:0015175 1 17

C0013144 GO:0006752 1 17

C0013144 GO:0048638 1 17

C0013144 GO:0033613 1 17

C0013144 GO:0007498 1 17

C0013144 GO:0048145 2 17

C0013144 GO:0033209 1 17

C0013144 GO:0046579 1 17

C0013144 GO:0008144 1 17

C0013144 GO:0031228 2 17

C0013144 GO:0042177 1 17

C0013144 GO:0004693 1 17

C0013144 GO:0004691 1 17

C0013144 GO:0004690 1 17

C0013144 GO:0051457 1 17

C0013144 GO:0045078 1 17

C0013144 GO:0043489 1 17

C0013144 GO:0045072 1 17

C0013144 GO:0030705 1 17

C0013144 GO:0033273 1 17

C0013144 GO:0004712 1 17

C0013144 GO:0005506 1 17

C0013144 GO:0005507 1 17

C0013144 GO:0005501 1 17

C0013144 GO:0008366 1 17

C0013144 GO:0008286 2 17

C0013144 GO:0008329 1 17

C0013144 GO:0030317 1 17

C0013144 GO:0015929 1 17

C0013144 GO:0045211 1 17

C0013144 GO:0046883 2 17

C0013144 GO:0001654 1 17

C0013144 GO:0046887 3 17

C0013144 GO:0033293 1 17

C0013144 GO:0060348 1 17

C0013144 GO:0043120 2 17

C0013144 GO:0004190 1 17

C0013144 GO:0019047 1 17

C0013144 GO:0045927 1 17

C0013144 GO:0030031 1 17

C0013144 GO:0030035 1 17

C0013144 GO:0017048 1 17

C0013144 GO:0060021 2 17

C0013144 GO:0022406 1 17

C0013144 GO:0045445 1 17

C0013144 GO:0007272 1 17

C0013144 GO:0006090 1 17

C0013144 GO:0010828 2 17

C0013144 GO:0009820 1 17

C0013144 GO:0055092 1 17

C0013144 GO:0046823 1 17

C0013144 GO:0032526 1 17

C0013144 GO:0006700 1 17

C0013144 GO:0045089 1 17

C0013144 GO:0019218 1 17

C0013144 GO:0015662 1 17

C0013144 GO:0050750 1 17

C0013144 GO:0010595 1 17

C0013144 GO:0010594 1 17

C0013144 GO:0006518 1 17

C0013144 GO:0050995 1 17

C0013144 GO:0050994 1 17

C0013144 GO:0051806 1 17

C0013144 GO:0043112 1 17

C0013144 GO:0006413 2 17

C0013144 GO:0007172 1 17

C0013144 GO:0009593 2 17

C0013144 GO:0048771 1 17

C0013144 GO:0007162 1 17

C0013144 GO:0007163 1 17

C0013144 GO:0043524 1 17

C0013144 GO:0043525 1 17

C0013144 GO:0019915 1 17

C0013144 GO:0031343 2 17

C0013144 GO:0016597 1 17

C0013144 GO:0031346 1 17

C0013144 GO:0042446 1 17

C0013144 GO:0005540 1 17

C0013144 GO:0002263 2 17

C0013144 GO:0033032 1 17

C0013144 GO:0051057 1 17

C0013144 GO:0051055 1 17

C0013144 GO:0033143 1 17

C0013144 GO:0006937 1 17

C0013144 GO:0030218 2 17

C0013144 GO:0051059 2 17

C0013144 GO:0051058 1 17

C0013144 GO:0008170 1 17

C0013144 GO:0016894 1 17

C0013144 GO:0051702 2 17

C0013144 GO:0042994 1 17

C0013144 GO:0042992 1 17

C0013144 GO:0016566 1 17

C0013144 GO:0006942 1 17

C0013144 GO:0016291 1 17

C0013144 GO:0004708 1 17

C0013144 GO:0046718 1 17

C0013144 GO:0046716 1 17

C0013144 GO:0050820 1 17

C0013144 GO:0001763 1 17

C0013144 GO:0046496 2 17

C0013144 GO:0046326 2 17

C0013144 GO:0048002 1 17

C0013144 GO:0030286 1 17

C0013144 GO:0007530 1 17

C0013144 GO:0051181 2 17

C0013144 GO:0051180 1 17

C0013144 GO:0046915 1 17

C0013144 GO:0001910 1 17

C0013144 GO:0044246 2 17

C0013144 GO:0030149 1 17

C0013144 GO:0009880 1 17

C0013144 GO:0032769 1 17

C0013144 GO:0017015 2 17

C0013144 GO:0030069 1 17

C0013144 GO:0046660 1 17

C0013144 GO:0042269 1 17

C0013144 GO:0032364 1 17

C0013144 GO:0016709 1 17

C0013144 GO:0010712 2 17

C0013144 GO:0034384 1 17

C0013144 GO:0006513 1 17

C0013144 GO:0017080 1 17

C0013144 GO:0002690 1 17

C0013144 GO:0003746 1 17

C0013144 GO:0042834 1 17

C0013144 GO:0009116 1 17

C0013144 GO:0006633 1 17

C0013144 GO:0021537 1 17

C0013144 GO:0006637 1 17

C0013144 GO:0006635 1 17

C0013144 GO:0009311 1 17

C0013144 GO:0060263 1 17

C0013144 GO:0001948 1 17

C0013144 GO:0005583 2 17

C0013144 GO:0015166 1 17

C0013144 GO:0006749 1 17

C0013144 GO:0031397 1 17

C0013144 GO:0005355 1 17

C0013144 GO:0042169 1 17

C0013144 GO:0008034 1 17

C0013144 GO:0032587 1 17

C0013144 GO:0009062 1 17

C0013144 GO:0030804 1 17

C0013144 GO:0030801 1 17

C0013144 GO:0002237 6 17

C0013144 GO:0045619 1 17

C0013144 GO:0005518 1 17

C0013144 GO:0030323 1 17

C0013144 GO:0030324 1 17

C0013144 GO:0050868 1 17

C0013144 GO:0046365 1 17

C0013144 GO:0050866 1 17

C0013144 GO:0016234 1 17

C0013144 GO:0034623 1 17

C0013144 GO:0046890 1 17

C0013144 GO:0004181 1 17

C0013144 GO:0004675 1 17

C0013144 GO:0042787 1 17

C0013144 GO:0033189 1 17

C0013144 GO:0010812 2 17

C0013144 GO:0030021 1 17

C0013144 GO:0016903 1 17

C0013144 GO:0055067 1 17

C0013144 GO:0043627 3 17

C0013144 GO:0007266 1 17

C0013144 GO:0009746 1 17

C0013144 GO:0043190 1 17

C0013144 GO:0042345 3 17

C0013144 GO:0002791 1 17

C0013144 GO:0002793 1 17

C0013144 GO:0051923 1 17

C0013144 GO:0052192 1 17

C0013144 GO:0009749 1 17

C0013144 GO:0044409 1 17

C0013144 GO:0008645 1 17

C0013144 GO:0060538 1 17

C0013144 GO:0046697 1 17

C0013144 GO:0019362 2 17

C0013144 GO:0090087 1 17

C0013144 GO:0000242 1 17

C0013144 GO:0031970 1 17

C0013144 GO:0032387 1 17

C0013144 GO:0019228 1 17

C0013144 GO:0005788 1 17

C0013144 GO:0031058 1 17

C0013144 GO:0045981 1 17

C0013144 GO:0005770 1 17

C0013144 GO:0016338 1 17

C0013144 GO:0007217 2 17

C0013144 GO:0070001 1 17

C0013144 GO:0006073 1 17

C0013144 GO:0046870 1 17

C0013144 GO:0032722 2 17

C0013144 GO:0070228 1 17

C0013144 GO:0032655 1 17

C0013144 GO:0005159 1 17

C0013144 GO:0005158 1 17

C0013144 GO:0042129 2 17

C0013144 GO:0043536 1 17

C0013144 GO:0043535 1 17

C0013144 GO:0046631 1 17

C0013144 GO:0008630 1 17

C0013144 GO:0055072 1 17

C0013144 GO:0004551 1 17

C0013144 GO:0045494 2 17

C0013144 GO:0003015 1 17

C0013144 GO:0046006 1 17

C0013144 GO:0042398 1 17

C0013144 GO:0006929 1 17

C0013144 GO:0051893 1 17

C0013144 GO:0070169 1 17

C0013144 GO:0034765 1 17

C0013144 GO:0031334 1 17

C0013144 GO:0021700 1 17

C0013144 GO:0042625 1 17

C0013144 GO:0031672 1 17

C0013144 GO:0005858 1 17

C0013144 GO:0048641 1 17

C0013144 GO:0005520 1 17

C0013144 GO:0045727 1 17

C0013144 GO:0030879 2 17

C0013144 GO:0070167 1 17

C0013144 GO:0050810 3 17

C0013144 GO:0042308 1 17

C0013144 GO:0030278 1 17

C0013144 GO:0005930 2 17

C0013144 GO:0042301 1 17

C0013144 GO:0044275 1 17

C0013144 GO:0008093 1 17

C0013144 GO:0044272 1 17

C0013144 GO:0016830 1 17

C0013144 GO:0044447 1 17

C0013144 GO:0031016 1 17

C0013144 GO:0045580 2 17

C0013144 GO:0045429 2 17

C0013144 GO:0060047 1 17

C0013144 GO:0046677 1 17

C0013144 GO:0035023 1 17

C0013144 GO:0008543 1 17

C0013144 GO:0007250 1 17

C0013144 GO:0032376 1 17

C0013144 GO:0032370 1 17

C0013144 GO:0032373 1 17

C0013144 GO:0070688 1 17

C0013144 GO:0044042 1 17

C0013144 GO:0019209 2 17

C0013144 GO:0031330 1 17

C0013144 GO:0000217 1 17

C0013144 GO:0004864 1 17

C0013144 GO:0005593 1 17

C0013144 GO:0050770 1 17

C0013144 GO:0001953 2 17

C0013144 GO:0050671 2 17

C0013144 GO:0005024 1 17

C0013144 GO:0051828 1 17

C0013144 GO:0006007 1 17

C0013144 GO:0001818 3 17

C0013144 GO:0009187 1 17

C0013144 GO:0042119 1 17

C0013144 GO:0034235 1 17

C0013144 GO:0043507 1 17

C0013144 GO:0005790 1 17

C0013144 GO:0045768 1 17

C0013144 GO:0015074 1 17

C0013144 GO:0045766 2 17

C0013144 GO:0015758 1 17

C0013144 GO:0015695 2 17

C0013144 GO:0007584 1 17

C0013144 GO:0007585 1 17

C0013144 GO:0009112 1 17

C0013144 GO:0009110 1 17

C0013144 GO:0042026 1 17

C0013144 GO:0046580 1 17

C0013144 GO:0051646 1 17

C0013144 GO:0034599 1 17

C0013144 GO:0001530 2 17

C0013144 GO:0016877 1 17

C0013144 GO:0016620 1 17

C0013144 GO:0019840 1 17

C0013144 GO:0030902 1 17

C0013144 GO:0016504 1 17

C0013144 GO:0016505 1 17

C0013144 GO:0035065 1 17

C0013144 GO:0008021 1 17

C0013144 GO:0030427 1 17

C0013144 GO:0008585 1 17

C0013144 GO:0050926 1 17

C0013144 GO:0050927 1 17

C0013144 GO:0030345 1 17

C0013144 GO:0008374 3 17

C0013144 GO:0008375 1 17

C0013144 GO:0051897 1 17

C0013144 GO:0008373 1 17

C0013144 GO:0031985 1 17

C0013144 GO:0007519 1 17

C0013144 GO:0043325 1 17

C0013144 GO:0010896 1 17

C0013144 GO:0010898 1 17

C0013144 GO:0030169 2 17

C0013144 GO:0032393 2 17

C0013144 GO:0005796 1 17

C0013144 GO:0006760 1 17

C0013144 GO:0006769 2 17

C0013144 GO:0030166 1 17

C0013144 GO:0030165 1 17

C0013144 GO:0010975 1 17

C0013144 GO:0030041 1 17

C0013144 GO:0030512 1 17

C0013144 GO:0010874 1 17

C0013144 GO:0010875 1 17

C0013144 GO:0045414 1 17

C0013144 GO:0015833 1 17

C0013144 GO:0051353 1 17

C0013144 GO:0060048 1 17

C0013144 GO:0048500 1 17

C0013144 GO:0032731 1 17

C0013144 GO:0060416 1 17

C0013144 GO:0030194 1 17

C0013144 GO:0005089 1 17

C0013144 GO:0005088 1 17

C0013144 GO:0045454 1 17

C0013144 GO:0005086 1 17

C0013144 GO:0019934 1 17

C0013144 GO:0051453 1 17

C0013144 GO:0032496 3 17

C0013144 GO:0002286 1 17

C0013144 GO:0050709 1 17

C0013144 GO:0002285 1 17

C0013144 GO:0016045 1 17

C0013144 GO:0048385 1 17

C0013144 GO:0050700 2 17

C0013144 GO:0015149 1 17

C0013144 GO:0002366 2 17

C0013144 GO:0015145 1 17

C0013144 GO:0046545 1 17

C0013144 GO:0008154 1 17

C0013144 GO:0048255 1 17

C0013144 GO:0042632 1 17

C0013144 GO:0043254 1 17

C0013144 GO:0042102 2 17

C0013144 GO:0045165 1 17

C0013144 GO:0042734 2 17

C0013144 GO:0031645 1 17

C0013144 GO:0002715 1 17

C0013144 GO:0016461 1 17

C0013144 GO:0045730 2 17

C0013144 GO:0045739 1 17

C0013144 GO:0015665 1 17

C0013144 GO:0050808 1 17

C0013144 GO:0002819 1 17

C0013144 GO:0051004 1 17

C0013144 GO:0030260 1 17

C0013144 GO:0005903 1 17

C0013144 GO:0048029 1 17

C0013144 GO:0052126 1 17

C0013144 GO:0043087 1 17

C0013144 GO:0009124 1 17

C0013144 GO:0000041 1 17

C0013144 GO:0046782 1 17

C0013144 GO:0046466 1 17

C0013144 GO:0022614 1 17

C0013144 GO:0015296 1 17

C0013144 GO:0015807 2 17

C0013144 GO:0008206 1 17

C0013144 GO:0001893 1 17

C0013144 GO:0045598 1 17

C0013144 GO:0008635 1 17

C0013144 GO:0005977 2 17

C0013144 GO:0045940 1 17

C0013144 GO:0045861 2 17

C0013144 GO:0000185 1 17

C0013144 GO:0005929 1 17

C0013144 GO:0046112 1 17

C0013144 GO:0045682 1 17

C0013144 GO:0030641 1 17

C0013144 GO:0050660 1 17

C0013144 GO:0030810 1 17

C0013144 GO:0010741 1 17

C0013144 GO:0048662 1 17

C0013144 GO:0048660 1 17

C0013144 GO:0042104 1 17

C0013144 GO:0048742 1 17

C0013144 GO:0033483 1 17

C0013144 GO:0002449 1 17

C0013144 GO:0031214 2 17

C0013144 GO:0002440 1 17

C0013144 GO:0016049 1 17

C0013144 GO:0002764 1 17

C0013144 GO:0019212 1 17

C0013144 GO:0015749 1 17

C0013144 GO:0001937 1 17

C0013144 GO:0015036 1 17

C0043352 GO:0051044 1 7

C0043352 GO:0035065 1 7

C0043352 GO:0048771 1 7

C0043352 GO:0006477 1 7

C0043352 GO:0002706 1 7

C0043352 GO:0050926 1 7

C0043352 GO:0050927 1 7

C0043352 GO:0002703 2 7

C0043352 GO:0043525 1 7

C0043352 GO:0034199 1 7

C0043352 GO:0030345 1 7

C0043352 GO:0008374 1 7

C0043352 GO:0034765 1 7

C0043352 GO:0046631 1 7

C0043352 GO:0016597 1 7

C0043352 GO:0034762 1 7

C0043352 GO:0030228 1 7

C0043352 GO:0002263 1 7

C0043352 GO:0016338 1 7

C0043352 GO:0030295 2 7

C0043352 GO:0050715 1 7

C0043352 GO:0030856 1 7

C0043352 GO:0010874 1 7

C0043352 GO:0006073 1 7

C0043352 GO:0010875 1 7

C0043352 GO:0043120 1 7

C0043352 GO:0015002 1 7

C0043352 GO:0060541 1 7

C0043352 GO:0070228 2 7

C0043352 GO:0032655 1 7

C0043352 GO:0002822 1 7

C0043352 GO:0007281 1 7

C0043352 GO:0043536 1 7

C0043352 GO:0008170 1 7

C0043352 GO:0007172 1 7

C0043352 GO:0010975 1 7

C0043352 GO:0007286 1 7

C0043352 GO:0030041 1 7

C0043352 GO:0030514 1 7

C0043352 GO:0042992 1 7

C0043352 GO:0030512 1 7

C0043352 GO:0030296 1 7

C0043352 GO:0030169 1 7

C0043352 GO:0031343 1 7

C0043352 GO:0016812 1 7

C0043352 GO:0016814 1 7

C0043352 GO:0051353 1 7

C0043352 GO:0001910 1 7

C0043352 GO:0034329 1 7

C0043352 GO:0005089 1 7

C0043352 GO:0005088 1 7

C0043352 GO:0060263 1 7

C0043352 GO:0032412 1 7

C0043352 GO:0009898 1 7

C0043352 GO:0005086 1 7

C0043352 GO:0046320 1 7

C0043352 GO:0000578 1 7

C0043352 GO:0046326 1 7

C0043352 GO:0048002 1 7

C0043352 GO:0021700 1 7

C0043352 GO:0016863 1 7

C0043352 GO:0035004 1 7

C0043352 GO:0007530 1 7

C0043352 GO:0051181 1 7

C0043352 GO:0043547 1 7

C0043352 GO:0002285 1 7

C0043352 GO:0004129 1 7

C0043352 GO:0051457 1 7

C0043352 GO:0002366 1 7

C0043352 GO:0006112 1 7

C0043352 GO:0009124 1 7

C0043352 GO:0006110 1 7

C0043352 GO:0045912 2 7

C0043352 GO:0016860 2 7

C0043352 GO:0030879 1 7

C0043352 GO:0002688 1 7

C0043352 GO:0048705 1 7

C0043352 GO:0019882 1 7

C0043352 GO:0019883 1 7

C0043352 GO:0016303 1 7

C0043352 GO:0032769 1 7

C0043352 GO:0005929 1 7

C0043352 GO:0005138 1 7

C0043352 GO:0008154 1 7

C0043352 GO:0050810 1 7

C0043352 GO:0034713 1 7

C0043352 GO:0042308 1 7

C0043352 GO:0030069 1 7

C0043352 GO:0005930 1 7

C0043352 GO:0042301 1 7

C0043352 GO:0044042 1 7

C0043352 GO:0045165 1 7

C0043352 GO:0042269 1 7

C0043352 GO:0042734 1 7

C0043352 GO:0015300 1 7

C0043352 GO:0022898 1 7

C0043352 GO:0030510 1 7

C0043352 GO:0005640 1 7

C0043352 GO:0010712 1 7

C0043352 GO:0070567 1 7

C0043352 GO:0045730 1 7

C0043352 GO:0016676 1 7

C0043352 GO:0002690 1 7

C0043352 GO:0016675 1 7

C0043352 GO:0042834 1 7

C0043352 GO:0048638 1 7

C0043352 GO:0019320 1 7

C0043352 GO:0005788 1 7

C0043352 GO:0008144 1 7

C0043352 GO:0045429 1 7

C0043352 GO:0019047 1 7

C0043352 GO:0048029 1 7

C0043352 GO:0035023 1 7

C0043352 GO:0048641 1 7

C0043352 GO:0043087 1 7

C0043352 GO:0044246 1 7

C0043352 GO:0007250 1 7

C0043352 GO:0032376 1 7

C0043352 GO:0032370 1 7

C0043352 GO:0032373 1 7

C0043352 GO:0032409 1 7

C0043352 GO:0031397 1 7

C0043352 GO:0005506 1 7

C0043352 GO:0044275 1 7

C0043352 GO:0015297 1 7

C0043352 GO:0050866 1 7

C0043352 GO:0008329 1 7

C0043352 GO:0001654 1 7

C0043352 GO:0001656 1 7

C0043352 GO:0010677 2 7

C0043352 GO:0005593 1 7

C0043352 GO:0042562 1 7

C0043352 GO:0015144 1 7

C0043352 GO:0043470 1 7

C0043352 GO:0043471 1 7

C0043352 GO:0042345 1 7

C0043352 GO:0008633 1 7

C0043352 GO:0031645 1 7

C0043352 GO:0006007 1 7

C0043352 GO:0030323 1 7

C0043352 GO:0030324 1 7

C0043352 GO:0050868 1 7

C0043352 GO:0050770 1 7

C0043352 GO:0046365 1 7

C0043352 GO:0019209 2 7

C0043352 GO:0017048 1 7

C0043352 GO:0060021 1 7

C0043352 GO:0015296 1 7

C0043352 GO:0001889 1 7

C0043352 GO:0045682 1 7

C0043352 GO:0010828 1 7

C0043352 GO:0045768 1 7

C0043352 GO:0015074 1 7

C0043352 GO:0004181 1 7

C0043352 GO:0045766 1 7

C0043352 GO:0030134 1 7

C0043352 GO:0015695 1 7

C0043352 GO:0008543 1 7

C0043352 GO:0051897 1 7

C0043352 GO:0046823 1 7

C0043352 GO:0006700 1 7

C0043352 GO:0048662 1 7

C0043352 GO:0048660 1 7

C0043352 GO:0048593 1 7

C0043352 GO:0030021 1 7

C0043352 GO:0002715 1 7

C0043352 GO:0031214 1 7

C0043352 GO:0043627 1 7

C0043352 GO:0002286 1 7

C0043352 GO:0001530 1 7

C0043352 GO:0043190 1 7

C0043352 GO:0043409 1 7

C0043352 GO:0010595 1 7

C0043352 GO:0010594 1 7

C0043352 GO:0005977 2 7

C0043352 GO:0090101 1 7

C0043352 GO:0051923 1 7

C0043352 GO:0043535 1 7

C0043352 GO:0032320 1 7

C0043352 GO:0006413 1 7

C0043352 GO:0048742 1 7

C0043352 GO:0015036 1 7

C0021400 GO:0051044 2 6

C0021400 GO:0034358 1 6

C0021400 GO:0042036 2 6

C0021400 GO:0006909 1 6

C0021400 GO:0048261 1 6

C0021400 GO:0016863 2 6

C0021400 GO:0007598 1 6

C0021400 GO:0006306 2 6

C0021400 GO:0006305 2 6

C0021400 GO:0006304 1 6

C0021400 GO:0000002 1 6

C0021400 GO:0019992 1 6

C0021400 GO:0018210 1 6

C0021400 GO:0005833 1 6

C0021400 GO:0004716 1 6

C0021400 GO:0016459 2 6

C0021400 GO:0016101 1 6

C0021400 GO:0060541 1 6

C0021400 GO:0030595 1 6

C0021400 GO:0045851 1 6

C0021400 GO:0004029 1 6

C0021400 GO:0035176 1 6

C0021400 GO:0019229 1 6

C0021400 GO:0007281 1 6

C0021400 GO:0008360 1 6

C0021400 GO:0005788 1 6

C0021400 GO:0030291 1 6

C0021400 GO:0006688 1 6

C0021400 GO:0016812 1 6

C0021400 GO:0006953 1 6

C0021400 GO:0016811 1 6

C0021400 GO:0070633 1 6

C0021400 GO:0003995 1 6

C0021400 GO:0005436 1 6

C0021400 GO:0033261 1 6

C0021400 GO:0010888 1 6

C0021400 GO:0009895 1 6

C0021400 GO:0048568 1 6

C0021400 GO:0010869 1 6

C0021400 GO:0030170 1 6

C0021400 GO:0010862 1 6

C0021400 GO:0051224 1 6

C0021400 GO:0030073 1 6

C0021400 GO:0003205 1 6

C0021400 GO:0003206 1 6

C0021400 GO:0042551 1 6

C0021400 GO:0006885 1 6

C0021400 GO:0003208 1 6

C0021400 GO:0043028 1 6

C0021400 GO:0046850 2 6

C0021400 GO:0006110 1 6

C0021400 GO:0045912 1 6

C0021400 GO:0016860 2 6

C0021400 GO:0043025 1 6

C0021400 GO:0043027 1 6

C0021400 GO:0006303 1 6

C0021400 GO:0019882 2 6

C0021400 GO:0070169 2 6

C0021400 GO:0007623 1 6

C0021400 GO:0009306 1 6

C0021400 GO:0031256 1 6

C0021400 GO:0070167 1 6

C0021400 GO:0006584 1 6

C0021400 GO:0060606 1 6

C0021400 GO:0002526 1 6

C0021400 GO:0050714 1 6

C0021400 GO:0050650 1 6

C0021400 GO:0050654 1 6

C0021400 GO:0006690 1 6

C0021400 GO:0006029 1 6

C0021400 GO:0015179 1 6

C0021400 GO:0048407 1 6

C0021400 GO:0006026 1 6

C0021400 GO:0006027 1 6

C0021400 GO:0015172 1 6

C0021400 GO:0048638 1 6

C0021400 GO:0048634 1 6

C0021400 GO:0018108 1 6

C0021400 GO:0009410 1 6

C0021400 GO:0051453 1 6

C0021400 GO:0051452 1 6

C0021400 GO:0051457 1 6

C0021400 GO:0050431 1 6

C0021400 GO:0030705 1 6

C0021400 GO:0045621 1 6

C0021400 GO:0045622 1 6

C0021400 GO:0032677 1 6

C0021400 GO:0006879 1 6

C0021400 GO:0008329 1 6

C0021400 GO:0055008 1 6

C0021400 GO:0048872 1 6

C0021400 GO:0034637 1 6

C0021400 GO:0001654 2 6

C0021400 GO:0010675 1 6

C0021400 GO:0045216 1 6

C0021400 GO:0010677 1 6

C0021400 GO:0033293 1 6

C0021400 GO:0009952 1 6

C0021400 GO:0032722 1 6

C0021400 GO:0004190 2 6

C0021400 GO:0016525 1 6

C0021400 GO:0017046 1 6

C0021400 GO:0045923 1 6

C0021400 GO:0015813 1 6

C0021400 GO:0015810 1 6

C0021400 GO:0019048 1 6

C0021400 GO:0030035 1 6

C0021400 GO:0017048 1 6

C0021400 GO:0042593 1 6

C0021400 GO:0050906 1 6

C0021400 GO:0050905 1 6

C0021400 GO:0007270 2 6

C0021400 GO:0007271 1 6

C0021400 GO:0050909 1 6

C0021400 GO:0055092 2 6

C0021400 GO:0006094 1 6

C0021400 GO:0042359 1 6

C0021400 GO:0032350 1 6

C0021400 GO:0042354 1 6

C0021400 GO:0006096 2 6

C0021400 GO:0045639 1 6

C0021400 GO:0070668 1 6

C0021400 GO:0045736 1 6

C0021400 GO:0021915 1 6

C0021400 GO:0048593 1 6

C0021400 GO:0032856 1 6

C0021400 GO:0004969 1 6

C0021400 GO:0042509 1 6

C0021400 GO:0010595 1 6

C0021400 GO:0010594 3 6

C0021400 GO:0010596 1 6

C0021400 GO:0006109 1 6

C0021400 GO:0042752 1 6

C0021400 GO:0016986 1 6

C0021400 GO:0015269 1 6

C0021400 GO:0035272 1 6

C0021400 GO:0006413 1 6

C0021400 GO:0009593 1 6

C0021400 GO:0005149 1 6

C0021400 GO:0007618 1 6

C0021400 GO:0007612 2 6

C0021400 GO:0007611 1 6

C0021400 GO:0007162 1 6

C0021400 GO:0007163 1 6

C0021400 GO:0019915 1 6

C0021400 GO:0002573 1 6

C0021400 GO:0016597 1 6

C0021400 GO:0002263 1 6

C0021400 GO:0030295 1 6

C0021400 GO:0045667 1 6

C0021400 GO:0045661 1 6

C0021400 GO:0045669 1 6

C0021400 GO:0033032 1 6

C0021400 GO:0016202 1 6

C0021400 GO:0034364 1 6

C0021400 GO:0034366 1 6

C0021400 GO:0051119 1 6

C0021400 GO:0030218 1 6

C0021400 GO:0051117 1 6

C0021400 GO:0048278 1 6

C0021400 GO:0016894 1 6

C0021400 GO:0051701 1 6

C0021400 GO:0042992 1 6

C0021400 GO:0051705 1 6

C0021400 GO:0014020 1 6

C0021400 GO:0045109 1 6

C0021400 GO:0046530 1 6

C0021400 GO:0004707 1 6

C0021400 GO:0016291 1 6

C0021400 GO:0005388 1 6

C0021400 GO:0016445 1 6

C0021400 GO:0005905 1 6

C0021400 GO:0006805 1 6

C0021400 GO:0048002 1 6

C0021400 GO:0034708 1 6

C0021400 GO:0002761 1 6

C0021400 GO:0051184 1 6

C0021400 GO:0051187 1 6

C0021400 GO:0051183 1 6

C0021400 GO:0046915 1 6

C0021400 GO:0030228 1 6

C0021400 GO:0030330 1 6

C0021400 GO:0008088 1 6

C0021400 GO:0007416 1 6

C0021400 GO:0019894 1 6

C0021400 GO:0016755 1 6

C0021400 GO:0004143 1 6

C0021400 GO:0007185 1 6

C0021400 GO:0030148 1 6

C0021400 GO:0001948 1 6

C0021400 GO:0031941 1 6

C0021400 GO:0030235 1 6

C0021400 GO:0020037 2 6

C0021400 GO:0032768 1 6

C0021400 GO:0050796 1 6

C0021400 GO:0050795 1 6

C0021400 GO:0003231 1 6

C0021400 GO:0009953 1 6

C0021400 GO:0008656 1 6

C0021400 GO:0034382 1 6

C0021400 GO:0032365 1 6

C0021400 GO:0043154 1 6

C0021400 GO:0046823 1 6

C0021400 GO:0003746 1 6

C0021400 GO:0043010 1 6

C0021400 GO:0002698 1 6

C0021400 GO:0006739 1 6

C0021400 GO:0006637 1 6

C0021400 GO:0019956 1 6

C0021400 GO:0009311 1 6

C0021400 GO:0070279 1 6

C0021400 GO:0042401 1 6

C0021400 GO:0007044 1 6

C0021400 GO:0007043 1 6

C0021400 GO:0050766 1 6

C0021400 GO:0050764 1 6

C0021400 GO:0005035 1 6

C0021400 GO:0005031 1 6

C0021400 GO:0001838 1 6

C0021400 GO:0070303 1 6

C0021400 GO:0019209 1 6

C0021400 GO:0070304 1 6

C0021400 GO:0005868 1 6

C0021400 GO:0032587 1 6

C0021400 GO:0010906 1 6

C0021400 GO:0043470 1 6

C0021400 GO:0043471 1 6

C0021400 GO:0030175 1 6

C0021400 GO:0045619 2 6

C0021400 GO:0005044 1 6

C0021400 GO:0034405 2 6

C0021400 GO:0045616 1 6

C0021400 GO:0009083 1 6

C0021400 GO:0016331 1 6

C0021400 GO:0030323 1 6

C0021400 GO:0033138 1 6

C0021400 GO:0050868 1 6

C0021400 GO:0055010 1 6

C0021400 GO:0050866 1 6

C0021400 GO:0019842 1 6

C0021400 GO:0051147 1 6

C0021400 GO:0048041 1 6

C0021400 GO:0051149 1 6

C0021400 GO:0050996 1 6

C0021400 GO:0001523 1 6

C0021400 GO:0031532 1 6

C0021400 GO:0004675 1 6

C0021400 GO:0016538 1 6

C0021400 GO:0032321 1 6

C0021400 GO:0030934 1 6

C0021400 GO:0060415 1 6

C0021400 GO:0043588 1 6

C0021400 GO:0045931 1 6

C0021400 GO:0045930 1 6

C0021400 GO:0050699 1 6

C0021400 GO:0046626 1 6

C0021400 GO:0055062 1 6

C0021400 GO:0045471 1 6

C0021400 GO:0055061 1 6

C0021400 GO:0016909 1 6

C0021400 GO:0035295 1 6

C0021400 GO:0005605 1 6

C0021400 GO:0032205 1 6

C0021400 GO:0051923 1 6

C0021400 GO:0003705 1 6

C0021400 GO:0044236 1 6

C0021400 GO:0055072 1 6

C0021400 GO:0060538 2 6

C0021400 GO:0010832 1 6

C0021400 GO:0045807 1 6

C0021400 GO:0032387 1 6

C0021400 GO:0021510 1 6

C0021400 GO:0006477 1 6

C0021400 GO:0006476 1 6

C0021400 GO:0055081 1 6

C0021400 GO:0030501 2 6

C0021400 GO:0048864 1 6

C0021400 GO:0015082 1 6

C0021400 GO:0042531 1 6

C0021400 GO:0070001 2 6

C0021400 GO:0043120 2 6

C0021400 GO:0070228 2 6

C0021400 GO:0032655 1 6

C0021400 GO:0032652 1 6

C0021400 GO:0005154 1 6

C0021400 GO:0000096 1 6

C0021400 GO:0003007 2 6

C0021400 GO:0043536 1 6

C0021400 GO:0043535 3 6

C0021400 GO:0043531 1 6

C0021400 GO:0005523 1 6

C0021400 GO:0048732 1 6

C0021400 GO:0060393 1 6

C0021400 GO:0002377 2 6

C0021400 GO:0043523 1 6

C0021400 GO:0004550 1 6

C0021400 GO:0004551 1 6

C0021400 GO:0030206 1 6

C0021400 GO:0030204 1 6

C0021400 GO:0030203 1 6

C0021400 GO:0030201 1 6

C0021400 GO:0006929 3 6

C0021400 GO:0019883 1 6

C0021400 GO:0050808 1 6

C0021400 GO:0070207 1 6

C0021400 GO:0048246 1 6

C0021400 GO:0042625 1 6

C0021400 GO:0042627 1 6

C0021400 GO:0090100 1 6

C0021400 GO:0043548 3 6

C0021400 GO:0031674 1 6

C0021400 GO:0005522 1 6

C0021400 GO:0048641 1 6

C0021400 GO:0002200 1 6

C0021400 GO:0001776 1 6

C0021400 GO:0043900 1 6

C0021400 GO:0050810 1 6

C0021400 GO:0006957 1 6

C0021400 GO:0030275 1 6

C0021400 GO:0030279 1 6

C0021400 GO:0050819 2 6

C0021400 GO:0044275 3 6

C0021400 GO:0043410 1 6

C0021400 GO:0008091 1 6

C0021400 GO:0008093 1 6

C0021400 GO:0044272 1 6

C0021400 GO:0044447 1 6

C0021400 GO:0032770 1 6

C0021400 GO:0015280 1 6

C0021400 GO:0010921 2 6

C0021400 GO:0042308 1 6

C0021400 GO:0045580 1 6

C0021400 GO:0045429 2 6

C0021400 GO:0045428 1 6

C0021400 GO:0046677 1 6

C0021400 GO:0003229 1 6

C0021400 GO:0035023 1 6

C0021400 GO:0030324 1 6

C0021400 GO:0008543 1 6

C0021400 GO:0030018 1 6

C0021400 GO:0051324 1 6

C0021400 GO:0045346 1 6

C0021400 GO:0050885 1 6

C0021400 GO:0042274 2 6

C0021400 GO:0032374 1 6

C0021400 GO:0032376 2 6

C0021400 GO:0032371 1 6

C0021400 GO:0032370 2 6

C0021400 GO:0032373 2 6

C0021400 GO:0015377 1 6

C0021400 GO:0060562 1 6

C0021400 GO:0016338 2 6

C0021400 GO:0006721 1 6

C0021400 GO:0009247 1 6

C0021400 GO:0004861 1 6

C0021400 GO:0005593 2 6

C0021400 GO:0050777 1 6

C0021400 GO:0050679 1 6

C0021400 GO:0007422 1 6

C0021400 GO:0005024 1 6

C0021400 GO:0001816 1 6

C0021400 GO:0022839 2 6

C0021400 GO:0006007 1 6

C0021400 GO:0032421 1 6

C0021400 GO:0042119 1 6

C0021400 GO:0005160 2 6

C0021400 GO:0005161 3 6

C0021400 GO:0035097 1 6

C0021400 GO:0043506 1 6

C0021400 GO:0043500 1 6

C0021400 GO:0045768 1 6

C0021400 GO:0030832 1 6

C0021400 GO:0070567 1 6

C0021400 GO:0045767 2 6

C0021400 GO:0043666 1 6

C0021400 GO:0015695 1 6

C0021400 GO:0004879 1 6

C0021400 GO:0045604 1 6

C0021400 GO:0046425 1 6

C0021400 GO:0007585 1 6

C0021400 GO:0008198 1 6

C0021400 GO:0034220 1 6

C0021400 GO:0003785 2 6

C0021400 GO:0034599 1 6

C0021400 GO:0001530 1 6

C0021400 GO:0006312 1 6

C0021400 GO:0045124 2 6

C0021400 GO:0030900 1 6

C0021400 GO:0004890 1 6

C0021400 GO:0016505 1 6

C0021400 GO:0007090 1 6

C0021400 GO:0046637 1 6

C0021400 GO:0046634 1 6

C0021400 GO:0046631 1 6

C0021400 GO:0033500 1 6

C0021400 GO:0007292 1 6

C0021400 GO:0050926 1 6

C0021400 GO:0050927 1 6

C0021400 GO:0050920 1 6

C0021400 GO:0030345 1 6

C0021400 GO:0051897 2 6

C0021400 GO:0007519 2 6

C0021400 GO:0044419 1 6

C0021400 GO:0045834 1 6

C0021400 GO:0010896 1 6

C0021400 GO:0010898 1 6

C0021400 GO:0030169 1 6

C0021400 GO:0032393 1 6

C0021400 GO:0032420 1 6

C0021400 GO:0005796 1 6

C0021400 GO:0019827 1 6

C0021400 GO:0032781 1 6

C0021400 GO:0030166 1 6

C0021400 GO:0030048 1 6

C0021400 GO:0010874 2 6

C0021400 GO:0010875 2 6

C0021400 GO:0043648 1 6

C0021400 GO:0015485 1 6

C0021400 GO:0048871 1 6

C0021400 GO:0006040 1 6

C0021400 GO:0001822 1 6

C0021400 GO:0051353 2 6

C0021400 GO:0030199 1 6

C0021400 GO:0030198 1 6

C0021400 GO:0030195 2 6

C0021400 GO:0005089 1 6

C0021400 GO:0005088 1 6

C0021400 GO:0007632 2 6

C0021400 GO:0032642 1 6

C0021400 GO:0032649 1 6

C0021400 GO:0005086 1 6

C0021400 GO:0044403 1 6

C0021400 GO:0034311 1 6

C0021400 GO:0002286 1 6

C0021400 GO:0002285 1 6

C0021400 GO:0030183 1 6

C0021400 GO:0002366 1 6

C0021400 GO:0006687 1 6

C0021400 GO:0001754 1 6

C0021400 GO:0001841 1 6

C0021400 GO:0001843 1 6

C0021400 GO:0048477 1 6

C0021400 GO:0030890 1 6

C0021400 GO:0050690 1 6

C0021400 GO:0043256 1 6

C0021400 GO:0042632 2 6

C0021400 GO:0045165 1 6

C0021400 GO:0004683 1 6

C0021400 GO:0031645 1 6

C0021400 GO:0030864 1 6

C0021400 GO:0045732 1 6

C0021400 GO:0045638 1 6

C0021400 GO:0045730 2 6

C0021400 GO:0043034 2 6

C0021400 GO:0005795 1 6

C0021400 GO:0015662 1 6

C0021400 GO:0007158 1 6

C0021400 GO:0009712 1 6

C0021400 GO:0004114 1 6

C0021400 GO:0004112 1 6

C0021400 GO:0035148 1 6

C0021400 GO:0006941 1 6

C0021400 GO:0048029 1 6

C0021400 GO:0009124 1 6

C0021400 GO:0001540 1 6

C0021400 GO:0006342 1 6

C0021400 GO:0000041 1 6

C0021400 GO:0046467 1 6

C0021400 GO:0015299 1 6

C0021400 GO:0015298 1 6

C0021400 GO:0015807 2 6

C0021400 GO:0015800 1 6

C0021400 GO:0010830 1 6

C0021400 GO:0018958 1 6

C0021400 GO:0001894 1 6

C0021400 GO:0055085 1 6

C0021400 GO:0045598 1 6

C0021400 GO:0007568 1 6

C0021400 GO:0043178 1 6

C0021400 GO:0046329 1 6

C0021400 GO:0042476 1 6

C0021400 GO:0045296 1 6

C0021400 GO:0045862 1 6

C0021400 GO:0006821 1 6

C0021400 GO:0030099 1 6

C0021400 GO:0043370 1 6

C0021400 GO:0045749 1 6

C0021400 GO:0019200 1 6

C0021400 GO:0019201 1 6

C0021400 GO:0030021 1 6

C0021400 GO:0014910 1 6

C0021400 GO:0045582 1 6

C0021400 GO:0045445 1 6

C0021400 GO:0000188 1 6

C0021400 GO:0050829 1 6

C0021400 GO:0032862 1 6

C0021400 GO:0060228 1 6

C0021400 GO:0030641 1 6

C0021400 GO:0045684 1 6

C0021400 GO:0070555 1 6

C0021400 GO:0042516 1 6

C0021400 GO:0042517 1 6

C0021400 GO:0031330 1 6

C0021400 GO:0010466 1 6

C0021400 GO:0070411 1 6

C0021400 GO:0010745 1 6

C0021400 GO:0010743 1 6

C0021400 GO:0034101 1 6

C0021400 GO:0034103 2 6

C0021400 GO:0048662 1 6

C0021400 GO:0048660 1 6

C0021400 GO:0048741 1 6

C0021400 GO:0000217 1 6

C0021400 GO:0048742 1 6

C0021400 GO:0042100 1 6

C0021400 GO:0002449 1 6

C0021400 GO:0005884 1 6

C0021400 GO:0031214 1 6

C0021400 GO:0002440 1 6

C0021400 GO:0032994 1 6

C0021400 GO:0045778 2 6

C0021400 GO:0033344 1 6

C0021400 GO:0002250 1 6

C0021400 GO:0006970 1 6

C1527304 GO:0046718 1 2

C1527304 GO:0019894 1 2

C1527304 GO:0034358 1 2

C1527304 GO:0033613 1 2

C1527304 GO:0042119 1 2

C1527304 GO:0005161 1 2

C1527304 GO:0006929 1 2

C1527304 GO:0010906 1 2

C1527304 GO:0030260 1 2

C1527304 GO:0007163 1 2

C1527304 GO:0001764 1 2

C1527304 GO:0002702 1 2

C1527304 GO:0000786 1 2

C1527304 GO:0052126 1 2

C1527304 GO:0002724 1 2

C1527304 GO:0043087 1 2

C1527304 GO:0002720 1 2

C1527304 GO:0070491 1 2

C1527304 GO:0032355 1 2

C1527304 GO:0006304 1 2

C1527304 GO:0000002 1 2

C1527304 GO:0015280 1 2

C1527304 GO:0007250 1 2

C1527304 GO:0042274 1 2

C1527304 GO:0004428 1 2

C1527304 GO:0032642 1 2

C1527304 GO:0031513 1 2

C1527304 GO:0070412 1 2

C1527304 GO:0070410 1 2

C1527304 GO:0007062 1 2

C1527304 GO:0043027 1 2

C1527304 GO:0050866 1 2

C1527304 GO:0034364 1 2

C1527304 GO:0006110 1 2

C1527304 GO:0034366 1 2

C1527304 GO:0032994 1 2

C1527304 GO:0045089 1 2

C1527304 GO:0008013 1 2

C1527304 GO:0060070 1 2

C1527304 GO:0001727 1 2

C1527304 GO:0001654 1 2

C1527304 GO:0051059 1 2

C1527304 GO:0002718 1 2

C1527304 GO:0007131 1 2

C1527304 GO:0050660 1 2

C1527304 GO:0007292 1 2

C1527304 GO:0043470 1 2

C1527304 GO:0043471 1 2

C1527304 GO:0007127 1 2

C1527304 GO:0008517 1 2

C1527304 GO:0052192 1 2

C1527304 GO:0033261 1 2

C1527304 GO:0051828 1 2

C1527304 GO:0044409 1 2

C1527304 GO:0001709 1 2

C1527304 GO:0051806 1 2

C1527304 GO:0004709 1 2

C0018524 GO:0005593 1 2

C0018524 GO:0048638 1 2

C0018524 GO:0032376 1 2

C0018524 GO:0051044 1 2

C0018524 GO:0030228 1 2

C0018524 GO:0045429 1 2

C0018524 GO:0010677 1 2

C0018524 GO:0006477 1 2

C0018524 GO:0043256 1 2

C0018524 GO:0050926 1 2

C0018524 GO:0008210 1 2

C0018524 GO:0032946 1 2

C0018524 GO:0048002 1 2

C0018524 GO:0046631 1 2

C0018524 GO:0009068 1 2

C0018524 GO:0051897 1 2

C0018524 GO:0009124 1 2

C0018524 GO:0016860 1 2

C0018524 GO:0003205 1 2

C0018524 GO:0003206 1 2

C0018524 GO:0002286 1 2

C0018524 GO:0048660 1 2

C0018524 GO:0050885 1 2

C0018524 GO:0002285 1 2

C0018524 GO:0016863 1 2

C0018524 GO:0002263 1 2

C0018524 GO:0016338 1 2

C0018524 GO:0032370 1 2

C0018524 GO:0032373 1 2

C0018524 GO:0002366 1 2

C0018524 GO:0015695 1 2

C0018524 GO:0006687 1 2

C0018524 GO:0070665 1 2

C0018524 GO:0043120 1 2

C0018524 GO:0070228 1 2

C0018524 GO:0019882 1 2

C0018524 GO:0019883 1 2

C0018524 GO:0048662 1 2

C0018524 GO:0008329 1 2

C0018524 GO:0048709 1 2

C0018524 GO:0043536 1 2

C0018524 GO:0043535 1 2

C0018524 GO:0001654 1 2

C0018524 GO:0005788 1 2

C0018524 GO:0045912 1 2

C0018524 GO:0014003 1 2

C0018524 GO:0001530 1 2

C0018524 GO:0010874 1 2

C0018524 GO:0010875 1 2

C0018524 GO:0045165 1 2

C0018524 GO:0010595 1 2

C0018524 GO:0050679 1 2

C0018524 GO:0016812 1 2

C0018524 GO:0010594 1 2

C0018524 GO:0051923 1 2

C0018524 GO:0050671 1 2

C0018524 GO:0051353 1 2

C0018524 GO:0050927 1 2

C0018524 GO:0046849 1 2

C0018524 GO:0050810 1 2

C0018524 GO:0006413 1 2

C0021116 GO:0032148 1 3

C0021116 GO:0051043 1 3

C0021116 GO:0050688 1 3

C0021116 GO:0031970 1 3

C0021116 GO:0008585 1 3

C0021116 GO:0031050 1 3

C0021116 GO:0019915 1 3

C0021116 GO:0051893 1 3

C0021116 GO:0031058 1 3

C0021116 GO:0016863 1 3

C0021116 GO:0030742 1 3

C0021116 GO:0007519 1 3

C0021116 GO:0070001 1 3

C0021116 GO:0006073 1 3

C0021116 GO:0010898 1 3

C0021116 GO:0046504 1 3

C0021116 GO:0005159 1 3

C0021116 GO:0005158 1 3

C0021116 GO:0014003 1 3

C0021116 GO:0030041 1 3

C0021116 GO:0007205 1 3

C0021116 GO:0010887 1 3

C0021116 GO:0046849 1 3

C0021116 GO:0030194 1 3

C0021116 GO:0046718 1 3

C0021116 GO:0009895 1 3

C0021116 GO:0046321 1 3

C0021116 GO:0050820 1 3

C0021116 GO:0032946 1 3

C0021116 GO:0009068 1 3

C0021116 GO:0070918 1 3

C0021116 GO:0003205 1 3

C0021116 GO:0003206 1 3

C0021116 GO:0046915 1 3

C0021116 GO:0006687 1 3

C0021116 GO:0051879 1 3

C0021116 GO:0046545 1 3

C0021116 GO:0048709 1 3

C0021116 GO:0048255 1 3

C0021116 GO:0043256 1 3

C0021116 GO:0043205 1 3

C0021116 GO:0046660 1 3

C0021116 GO:0004437 1 3

C0021116 GO:0050885 1 3

C0021116 GO:0032368 1 3

C0021116 GO:0050714 1 3

C0021116 GO:0010553 1 3

C0021116 GO:0006752 1 3

C0021116 GO:0005884 1 3

C0021116 GO:0021537 1 3

C0021116 GO:0030260 1 3

C0021116 GO:0005903 1 3

C0021116 GO:0046677 1 3

C0021116 GO:0052126 1 3

C0021116 GO:0051457 1 3

C0021116 GO:0045078 1 3

C0021116 GO:0043489 1 3

C0021116 GO:0045072 1 3

C0021116 GO:0046782 1 3

C0021116 GO:0044042 1 3

C0021116 GO:0008034 1 3

C0021116 GO:0010896 1 3

C0021116 GO:0031330 1 3

C0021116 GO:0000217 1 3

C0021116 GO:0045211 1 3

C0021116 GO:0015807 1 3

C0021116 GO:0002440 1 3

C0021116 GO:0004693 1 3

C0021116 GO:0019934 1 3

C0021116 GO:0050679 1 3

C0021116 GO:0046320 1 3

C0021116 GO:0050671 1 3

C0021116 GO:0051828 1 3

C0021116 GO:0004190 1 3

C0021116 GO:0045861 1 3

C0021116 GO:0050994 1 3

C0021116 GO:0008210 1 3

C0021116 GO:0034235 1 3

C0021116 GO:0070665 1 3

C0021116 GO:0033189 1 3

C0021116 GO:0010741 1 3

C0021116 GO:0032526 1 3

C0021116 GO:0031047 1 3

C0021116 GO:0051646 1 3

C0021116 GO:0007266 1 3

C0021116 GO:0042476 1 3

C0021116 GO:0031998 1 3

C0021116 GO:0035195 1 3

C0021116 GO:0035196 1 3

C0021116 GO:0050995 1 3

C0021116 GO:0052192 1 3

C0021116 GO:0044409 1 3

C0021116 GO:0060538 1 3

C0021116 GO:0051806 1 3

C0021116 GO:0001937 1 3

C0033975 GO:0005593 1 3

C0033975 GO:0048638 1 3

C0033975 GO:0050808 1 3

C0033975 GO:0051044 1 3

C0033975 GO:0030228 1 3

C0033975 GO:0051923 1 3

C0033975 GO:0048145 1 3

C0033975 GO:0010677 1 3

C0033975 GO:0051353 1 3

C0033975 GO:0006477 1 3

C0033975 GO:0010594 1 3

C0033975 GO:0043256 1 3

C0033975 GO:0050926 1 3

C0033975 GO:0008210 1 3

C0033975 GO:0032946 1 3

C0033975 GO:0050810 1 3

C0033975 GO:0010875 1 3

C0033975 GO:0046112 1 3

C0033975 GO:0046631 1 3

C0033975 GO:0009068 1 3

C0033975 GO:0016863 2 3

C0033975 GO:0009124 1 3

C0033975 GO:0016860 1 3

C0033975 GO:0003205 1 3

C0033975 GO:0045912 1 3

C0033975 GO:0003206 1 3

C0033975 GO:0002286 1 3

C0033975 GO:0048660 1 3

C0033975 GO:0050885 1 3

C0033975 GO:0002285 1 3

C0033975 GO:0051897 1 3

C0033975 GO:0048002 1 3

C0033975 GO:0002263 1 3

C0033975 GO:0050700 1 3

C0033975 GO:0032370 1 3

C0033975 GO:0032373 1 3

C0033975 GO:0045429 1 3

C0033975 GO:0002366 1 3

C0033975 GO:0070665 1 3

C0033975 GO:0006687 1 3

C0033975 GO:0015695 1 3

C0033975 GO:0045727 1 3

C0033975 GO:0043120 1 3

C0033975 GO:0016338 1 3

C0033975 GO:0070228 1 3

C0033975 GO:0019882 1 3

C0033975 GO:0008034 1 3

C0033975 GO:0048662 1 3

C0033975 GO:0008329 1 3

C0033975 GO:0048709 1 3

C0033975 GO:0015929 1 3

C0033975 GO:0033483 1 3

C0033975 GO:0009112 1 3

C0033975 GO:0043535 1 3

C0033975 GO:0001654 1 3

C0033975 GO:0005788 1 3

C0033975 GO:0046887 1 3

C0033975 GO:0014003 1 3

C0033975 GO:0043536 1 3

C0033975 GO:0001530 1 3

C0033975 GO:0010874 1 3

C0033975 GO:0030169 1 3

C0033975 GO:0009746 1 3

C0033975 GO:0045165 1 3

C0033975 GO:0010595 1 3

C0033975 GO:0050679 1 3

C0033975 GO:0034284 1 3

C0033975 GO:0016812 1 3

C0033975 GO:0032364 1 3

C0033975 GO:0002793 1 3

C0033975 GO:0016830 1 3

C0033975 GO:0050671 1 3

C0033975 GO:0009749 1 3

C0033975 GO:0019883 1 3

C0033975 GO:0050927 1 3

C0033975 GO:0046849 1 3

C0033975 GO:0032376 1 3

C0033975 GO:0005518 1 3

C0033975 GO:0006413 1 3

C0038663 GO:0016459 1 2

C0038663 GO:0046637 1 2

C0038663 GO:0046634 1 2

C0038663 GO:0048634 1 2

C0038663 GO:0006929 1 2

C0038663 GO:0035148 1 2

C0038663 GO:0006941 1 2

C0038663 GO:0043256 1 2

C0038663 GO:0008210 1 2

C0038663 GO:0032946 1 2

C0038663 GO:0030291 1 2

C0038663 GO:0043506 1 2

C0038663 GO:0009068 1 2

C0038663 GO:0055092 1 2

C0038663 GO:0003205 1 2

C0038663 GO:0003206 1 2

C0038663 GO:0050885 1 2

C0038663 GO:0032321 1 2

C0038663 GO:0007519 1 2

C0038663 GO:0045767 1 2

C0038663 GO:0016538 1 2

C0038663 GO:0045768 1 2

C0038663 GO:0070665 1 2

C0038663 GO:0006687 1 2

C0038663 GO:0045604 1 2

C0038663 GO:0005522 1 2

C0038663 GO:0060562 1 2

C0038663 GO:0006688 1 2

C0038663 GO:0045622 1 2

C0038663 GO:0045619 1 2

C0038663 GO:0016202 1 2

C0038663 GO:0016338 1 2

C0038663 GO:0014020 1 2

C0038663 GO:0009247 1 2

C0038663 GO:0048709 1 2

C0038663 GO:0060606 1 2

C0038663 GO:0001841 1 2

C0038663 GO:0032856 1 2

C0038663 GO:0017048 1 2

C0038663 GO:0045216 1 2

C0038663 GO:0031214 1 2

C0038663 GO:0014003 1 2

C0038663 GO:0042632 1 2

C0038663 GO:0035295 1 2

C0038663 GO:0001843 1 2

C0038663 GO:0001838 1 2

C0038663 GO:0045580 1 2

C0038663 GO:0050679 1 2

C0038663 GO:0004861 1 2

C0038663 GO:0050671 1 2

C0038663 GO:0043370 1 2

C0038663 GO:0032862 1 2

C0038663 GO:0046849 1 2

C0038663 GO:0060538 1 2

C0038663 GO:0007043 1 2

C0038663 GO:0045616 1 2

C0038663 GO:0033032 1 2

C0038663 GO:0005161 1 2

C0038663 GO:0043034 1 2

C0011175 GO:0003205 1 1

C0011175 GO:0003206 1 1

C0011175 GO:0050679 1 1

C0011175 GO:0050885 1 1

C0011175 GO:0048709 1 1

C0011175 GO:0050671 1 1

C0011175 GO:0014003 1 1

C0011175 GO:0008210 1 1

C0011175 GO:0032946 1 1

C0011175 GO:0006687 1 1

C0011175 GO:0043256 1 1

C0011175 GO:0070665 1 1

C0011175 GO:0009068 1 1

C0011175 GO:0046849 1 1

C0013428 GO:0050808 1 2

C0013428 GO:0048145 1 2

C0013428 GO:0043256 1 2

C0013428 GO:0008210 1 2

C0013428 GO:0032946 1 2

C0013428 GO:0046112 1 2

C0013428 GO:0009068 1 2

C0013428 GO:0016863 1 2

C0013428 GO:0003205 1 2

C0013428 GO:0003206 1 2

C0013428 GO:0050885 1 2

C0013428 GO:0050700 1 2

C0013428 GO:0070665 1 2

C0013428 GO:0006687 1 2

C0013428 GO:0045727 1 2

C0013428 GO:0008034 1 2

C0013428 GO:0048709 1 2

C0013428 GO:0015929 1 2

C0013428 GO:0033483 1 2

C0013428 GO:0009112 1 2

C0013428 GO:0046887 1 2

C0013428 GO:0014003 1 2

C0013428 GO:0030169 1 2

C0013428 GO:0009746 1 2

C0013428 GO:0050679 1 2

C0013428 GO:0034284 1 2

C0013428 GO:0032364 1 2

C0013428 GO:0002793 1 2

C0013428 GO:0016830 1 2

C0013428 GO:0050671 1 2

C0013428 GO:0009749 1 2

C0013428 GO:0046849 1 2

C0013428 GO:0005518 1 2

C0019112 GO:0003205 1 1

C0019112 GO:0003206 1 1

C0019112 GO:0050679 1 1

C0019112 GO:0050885 1 1

C0019112 GO:0048709 1 1

C0019112 GO:0050671 1 1

C0019112 GO:0014003 1 1

C0019112 GO:0008210 1 1

C0019112 GO:0032946 1 1

C0019112 GO:0006687 1 1

C0019112 GO:0043256 1 1

C0019112 GO:0070665 1 1

C0019112 GO:0009068 1 1

C0019112 GO:0046849 1 1

C0022650 GO:0003205 1 1

C0022650 GO:0003206 1 1

C0022650 GO:0050679 1 1

C0022650 GO:0050885 1 1

C0022650 GO:0048709 1 1

C0022650 GO:0050671 1 1

C0022650 GO:0014003 1 1

C0022650 GO:0008210 1 1

C0022650 GO:0032946 1 1

C0022650 GO:0006687 1 1

C0022650 GO:0043256 1 1

C0022650 GO:0070665 1 1

C0022650 GO:0009068 1 1

C0022650 GO:0046849 1 1

C0042109 GO:0003205 1 1

C0042109 GO:0003206 1 1

C0042109 GO:0050679 1 1

C0042109 GO:0050885 1 1

C0042109 GO:0048709 1 1

C0042109 GO:0050671 1 1

C0042109 GO:0014003 1 1

C0042109 GO:0008210 1 1

C0042109 GO:0032946 1 1

C0042109 GO:0006687 1 1

C0042109 GO:0043256 1 1

C0042109 GO:0070665 1 1

C0042109 GO:0009068 1 1

C0042109 GO:0046849 1 1

C0151786 GO:0003205 1 1

C0151786 GO:0003206 1 1

C0151786 GO:0050679 1 1

C0151786 GO:0050885 1 1

C0151786 GO:0048709 1 1

C0151786 GO:0050671 1 1

C0151786 GO:0014003 1 1

C0151786 GO:0008210 1 1

C0151786 GO:0032946 1 1

C0151786 GO:0006687 1 1

C0151786 GO:0043256 1 1

C0151786 GO:0070665 1 1

C0151786 GO:0009068 1 1

C0151786 GO:0046849 1 1

C0702166 GO:0021537 1 3

C0702166 GO:0032107 1 3

C0702166 GO:0032104 1 3

C0702166 GO:0051384 1 3

C0702166 GO:0032387 1 3

C0702166 GO:0008286 1 3

C0702166 GO:0043256 1 3

C0702166 GO:0008210 1 3

C0702166 GO:0032946 1 3

C0702166 GO:0043525 1 3

C0702166 GO:0043506 1 3

C0702166 GO:0009068 1 3

C0702166 GO:0043087 1 3

C0702166 GO:0003205 1 3

C0702166 GO:0003206 1 3

C0702166 GO:0030742 1 3

C0702166 GO:0043547 1 3

C0702166 GO:0050885 1 3

C0702166 GO:0043206 1 3

C0702166 GO:0016627 1 3

C0702166 GO:0050768 1 3

C0702166 GO:0070665 1 3

C0702166 GO:0006687 1 3

C0702166 GO:0010721 1 3

C0702166 GO:0004712 1 3

C0702166 GO:0015459 1 3

C0702166 GO:0043588 1 3

C0702166 GO:0046823 1 3

C0702166 GO:0042169 1 3

C0702166 GO:0015296 1 3

C0702166 GO:0009880 1 3

C0702166 GO:0031960 1 3

C0702166 GO:0048592 1 3

C0702166 GO:0048593 1 3

C0702166 GO:0048709 1 3

C0702166 GO:0005138 1 3

C0702166 GO:0021510 1 3

C0702166 GO:0031575 1 3

C0702166 GO:0001654 1 3

C0702166 GO:0002460 1 3

C0702166 GO:0001656 1 3

C0702166 GO:0042308 1 3

C0702166 GO:0014003 1 3

C0702166 GO:0043197 1 3

C0702166 GO:0042992 1 3

C0702166 GO:0050679 1 3

C0702166 GO:0007423 1 3

C0702166 GO:0046906 1 3

C0702166 GO:0016055 1 3

C0702166 GO:0018105 1 3

C0702166 GO:0050671 1 3

C0702166 GO:0060393 1 3

C0702166 GO:0032318 1 3

C0702166 GO:0032320 1 3

C0702166 GO:0030900 1 3

C0702166 GO:0046849 1 3

C0702166 GO:0002250 1 3

C0702166 GO:0043010 1 3

C0702166 GO:0004708 1 3

C0011124 GO:0046718 1 2

C0011124 GO:0032148 1 2

C0011124 GO:0050688 1 2

C0011124 GO:0043256 1 2

C0011124 GO:0052192 1 2

C0011124 GO:0046321 1 2

C0011124 GO:0031050 1 2

C0011124 GO:0008210 1 2

C0011124 GO:0032946 1 2

C0011124 GO:0034235 1 2

C0011124 GO:0052126 1 2

C0011124 GO:0009068 1 2

C0011124 GO:0016863 1 2

C0011124 GO:0070918 1 2
[truncated: 272,527 more chars]
